# Genome-Wide Identification of Histone Modifiers and Their Expression Patterns during Fruit Abscission in Litchi

**DOI:** 10.3389/fpls.2017.00639

**Published:** 2017-04-27

**Authors:** Manjun Peng, Peiyuan Ying, Xuncheng Liu, Caiqin Li, Rui Xia, Jianguo Li, Minglei Zhao

**Affiliations:** ^1^State Key Laboratory for Conservation and Utilization of Subtropical Agro-Bioresources, China Litchi Research Center, South China Agricultural UniversityGuangzhou, China; ^2^Guangdong Litchi Engineering Research Center, College of Horticulture, South China Agricultural UniversityGuangzhou, China; ^3^Key Laboratory of Plant Resources Conservation and Sustainable Utilization, South China Botanical Garden, Chinese Academy of SciencesGuangzhou, China

**Keywords:** litchi, fruit abscission, histone modifiers, gene expression, HDAC activity

## Abstract

Modifications to histones, including acetylation and methylation processes, play crucial roles in the regulation of gene expression in plant development as well as in stress responses. However, limited information on the enzymes catalyzing histone acetylation and methylation in non-model plants is currently available. In this study, several histone modifier (HM) types, including six histone acetyltransferases (HATs), 11 histone deacetylases (HDACs), 48 histone methyltransferases (HMTs), and 22 histone demethylases (HDMs), are identified in litchi (*Litchi chinensis* Sonn. cv. Feizixiao) based on similarities in their sequences to homologs in *Arabidopsis* (*A. thaliana*), tomato (*Solanum lycopersicum*), and rice (*Oryza sativa*). Phylogenetic analyses reveal that HM enzymes can be grouped into four HAT, two HDAC, two HMT, and two HDM subfamilies, respectively, while further expression profile analyses demonstrate that 17 HMs were significantly altered during fruit abscission in two field treatments. Analyses reveal that these genes exhibit four distinct patterns of expression in response to fruit abscission, while an *in vitro* assay was used to confirm the HDAC activity of LcHDA2, LcHDA6, and LcSRT2. Our findings are the first in-depth analysis of HMs in the litchi genome, and imply that some are likely to play important roles in fruit abscission in this commercially important plant.

## Introduction

Eukaryote genomic DNA (gDNA) is tightly compacted into a complex structure known as chromatin. The basic chromatin unit is the nucleosome which comprises ca. 146 base pairs of DNA wrapped onto a histone octamer that itself contains two examples of histone 2A, histone 2B, histone 3, and histone 4 (Luger et al., 1997). Each histone contains a structured globular domain and an unstructured amino-terminal tail that extends from the core nucleosome; these tails provide sites for a variety of post-translational modifications, including acetylation, methylation, phosphorylation, ubiquitination, and ADP-ribosylation (Berger, 2007).

The acetylation state of histones in the ε-amino group of conserved lysine residues is reversibly regulated by HATs and HDACs, which mainly target the H3 lysine (K) residues 9, 14, 18, and 23, as well as the H4 lysine (K) residues 5, 8, 12, 16, and 20 (Fuchs et al., 2006). Thus, based on their domain composition, plant HATs can be classified into one of four groups: (i) HAGs with an acetyltransf_1 domain (PF00583) (AT1) which includes GCN5-, ELP3-, and HAT1-like acetyltransferases; (ii) HAMs that include a MOZ-YBF2/SAS3-SAS2-TIP60 domain (MYSTs); (iii) HACs that are similar to the p300/CREB-binding protein; and (iv) HAFs related to the TATA-binding protein-associated factor 1 (Pandey et al., 2002). On the basis of their sequence similarity and cofactor dependencies, HDACs in all eukaryotes can also be divided into three families: reduced potassium dependence 3/histone deacetylase 1 (RPD3/HDA1); silent information regulator 2 (SIR2); and plant-specific histone deacetylase 2 (HD2) (Pandey et al., 2002). Specifically, nicotine adenine dinucleotide (NAD) is required as a cofactor by the SIR2 family (Haigis and Guarente, 2006), while members of the RPD3/HDA1 family need a Zn^2+^ cofactor for deacetylase activity (Yang and Seto, 2007).

Histone methylation is very complicated as it not only occurs within different residues (i.e., lysine and arginine) and at distinct sites, but also differs in terms of the number of methyl groups that are involved. Thus, different active and silent chromatin states can be characterized by different combinations of histone methylation modification patterns. Methylation of the histones H3K9, H3K27, H3K79, and H4K20, for example, is associated with gene silencing, while this process in H3K4 and H3K36 is related to gene activation (Zhang and Reinberg, 2001; Lachner and Jenuwein, 2002; Liu et al., 2010). Similar to acetylation, histone methylation is also a reversible process catalyzed by both HMTs and HDMs (Klose and Zhang, 2007); however, depending on different methylated residues, HMTs comprise both PRMT-, and SET-domain group (SDG)-like types which catalyze histone arginine methylation and lysine methylation, respectively (Liu et al., 2010). SDG-like HMTs have a conserved “suppressor of variegation, enhancer of zeste, and trithorax in *Drosophila*” (SET) domain which mediates methyltransferase catalytic activity. Based on the amino acid sequence conservation of SET domains, SDG-like HMTs are classified into seven groups (Ng et al., 2007): Class I comprises EZD, SANT (SM00717), CXC (PF03638), and SET (PF00856) domains, capable of transferring methyl groups to H3K27; Class II comprises N-terminal AWS (SM00570), SET, and post-SET (SM00508) domains, responsible for the methylation of H3K4 and/or H3K36; Class III comprises another group of SET genes responsible for the active mark H3K4me1/2/3, containing N-terminal PWWP (PF00855), FYRN (PF05964), and FYRC (PF05965), as well as two PHD, SET, and post-SET domains; Class IV is plant-specific, responsible for mono-methylation on H3K27, including N-terminal PHD and C-terminal SET domains; Class V is the largest SET group, comprising N-terminal SRA-YDG (PF02182) or WIYLD (PF10440), as well as pre-SET, SET, and post-SET domains; and Class VI and VII comprise genes that include interrupted SET or SET-related domains with unclear functions (Aiese et al., 2013; Gu et al., 2016).

Similarly, there are two types of HDMs: histone lysine demethylase 1 (KDM1), also known as lysine-specific demethylase 1 (LSD1), and Jumonji C (JmjC) domain-containing proteins. Previous work has shown that both KDM1/LSD1 and JmjC-type demethylases can remove methyl groups from methylated lysine residues (Shi et al., 2004; Tsukada et al., 2006); KDM1/LSD1-type HDMs require flavin adenine dinucleotide (FAD) as a cofactor to act on di- and mono-methylated lysines, while the demethylase activity of JmjC domain-containing proteins toward tri-, di-, and mono-methylated lysines are dependent on the presence of Fe (II) and α-ketoglutarate (αKG) cofactors (Klose and Zhang, 2007). Furthermore, JmjC proteins also have been found to demethylate arginine H3R2 and H4R3 in animal cells and H4R3 in *Arabidopsis* (Chang et al., 2007; Cho et al., 2012).

Organ abscission in plants is a programmed developmental process that facilitates the shedding of no longer necessary, infected, damaged, or senescent organs. This process can affect both vegetative and reproductive organs via cell wall dissolution in predetermined positions, referred to as abscission zones (AZs) that are often related to stress or senescence (Estornell et al., 2013). During abscission, the regulatory effects of plant hormones are key as they mediate plant organ responses to stress (Peleg and Blumwald, 2011; Estornell et al., 2013; Smékalová et al., 2014). Generally, ethylene and abscisic acid (ABA) act as abscission-accelerating signals (Sipes and Einset, 1983; Taylor and Whitelaw, 2001; Dal Cin et al., 2007), while auxin and gibberellins (GA) are thought to be abscission inhibitors (Bencheikh et al., 1997; Taylor and Whitelaw, 2001; Aziz, 2003). Recently, a large number of genes have been identified in tomato and litchi via RNA-seq that are differentially regulated during organ abscission and are involved in ethylene, auxin, ABA, and GA biosynthesis, as well as transport, metabolism, and signaling pathways. The presence of these genes further confirms that hormones are of particular importance to organ abscission as they are the effector molecules (Li et al., 2015a,b; Sundaresan et al., 2016).

HMs have attracted considerable research attention over recent decades as they are thought to be important regulators controlling gene transcription. An increasing number of these molecules have been identified and characterized in plants, and they are now known to play essential roles in a variety of growth and development processes as well as in stress responses via their interactions with other HMs and transcription factors (Thorstensen et al., 2011; Liu et al., 2014). In contrast, the role of HMs in perennial woody plants has so far received limited attention. One important example, litchi, is a perennial horticultural fruit tree that originates from southern China but has been widely cultivated in Southeast Asia because of its delicious and nutritious fruits. Litchi trees commonly fall victim to massive fruitlet abscission that causes low yields and heavy economic losses; however, whether or not HMs are involved in this abscission in litchi remains unknown.

The aim of this study was to address the functional relevance of HMs in litchi organ abscission. To do this, we used bioinformatics to identify a large number of HMs in the litchi genome and present a comprehensive overview of the structure, phylogeny, and composition of the classical HM families. We also investigated the expression of these enzymes during fruit abscission using two field treatments. The results of this study enable bioinformatic characterization of the complete set of litchi HMs, as well as their gene expression profiles during fruit abscission. Our results also facilitate the functional characterization of epigenetic regulators in this economically important fruit crop.

## Materials and methods

### Data collection and HM domain identification

Initially, HM protein sequences from *A. thaliana* (https://www.arabidopsis.org/index.jsp), *O. sativa* (http://www.ricedata.cn/gene/), and *S. lycopersicum* (https://solgenomics.net/) were retrieved, and domains typical to each enzyme family were extracted from multiple alignments. These were then used to search for putative HM proteins within the litchi genome (http://litchidb.genomics.cn/page/species/index.jsp). Next, the SET domain- and JmjC domain-containing protein sequences of *Fragaria vesca* (Gu et al., 2016), *Citrus sinensis*, and *Vitis vinifera* were downloaded from Phytozome (version 10.3; http://phytozome.jgi.doe.gov/pz/portal.html). Duplicates were removed from all acquired sequences and recognizable domains were analyzed using BLAST-based NCBI conserved domain searches (https://www.ncbi.nlm.nih.gov/Structure/cdd/wrpsb.cgi). The presence of these domains was then verified using the HMMER-based Simple Modular Architecture Research Tool database (SMART; http://smart.embl-heidelberg.de/) and as well as the Pfam software program (http://pfam.xfam.org/search). All of the HM protein sequences generated in this study are listed in the File S1 that accompanies this paper.

### Sequence alignment and phylogenetic analysis

Litchi HM proteins identified in this study were aligned with proteins from *A. thaliana, O. sativa, S. lycopersicum, F. vesca, C. sinensis*, and *V. vinifera* using the default option in the software program ClustalW (Thompson et al., 1994). Then, to further study the evolutionary relationship of these HMs, a series of phylogenetic trees were constructed using a maximum likelihood (ML) approach in the software program MEGA 5.2 (Tamura et al., 2011), and with the following parameters: Poisson correction, pairwise deletion and bootstrap analysis with 1,000 replicates.

### Plant materials and treatments

Three 9-year-old litchi trees that had been grown in an orchard at South China Agricultural University (Guangzhou, China) were randomly selected, and 30 fruit bearing shoots with similar diameter (about 5–8 mm) located in different directions on each tree were tagged. Ten of these shoots were then treated with a girdling bark ring about 0.5 cm in width; cambium was removed from the branch base prior to defoliation of all leaves above the girdle 35 days after anthesis via girdling plus defoliation (GPD) treatment. A further 10 shoots were then dipped in 250 mg L^−1^ ethephon solution containing 0.05% Tween-80 surfactant for 1 min (ETH treatment), while the remaining untreated shoots were used as controls. Samples were collected on the day of treatment as well as 1, 2, 3, and 4 days after treatment; the AZ was excised by cutting 2 mm around each side of the abscission fracture plane. Following separation, all tissues were rapidly frozen in liquid nitrogen and stored at −80°C for future analysis with each tree treated as a biological replicate.

### Quantitative real-time (RT-PCR) analysis

The first strand cDNA synthesis was generated using 2 μg total RNA isolated from litchi AZ tissues according to the manufacturer's instructions of TransScript One-Step gDNA Removal and cDNA Synthesis SuperMix Kit (TransGen, Beijing). Hundred nanograms of synthesized cDNA was used as a template to perform quantitative RT-PCR analysis. PCR reactions were performed in the total volume of 20 μL, with 0.5 μL for each primer (10 mm, final concentration 100 nm) and 10 μL for SYBR Green PCR Supermix (Bio-Rad) on a ABI7500 Real-Time PCR System (Applied Biosystems). The PCR program included an initial denaturation step at 94°C for 3 min, followed by 40 cycles of 5 s at 94°C and 1 min at 60°C. Each sample was quantified at least triplicate and normalized using *EF-1a* as an internal control for litchi (Zhong et al., 2011). The gene-specific primer pairs for quantitative Real-Time PCR are listed in Table S1. All PCR reactions were normalized using Ct value corresponding to the reference gene. The relative expression levels of the target gene were calculated with formula 2^−ddCt^ (Livak and Schmittgen, 2001). Values represented the average of three biological replicates.

### *In vitro* HDAC assay

In vitro HDAC activity was measured using the Epigenase HDAC Activity/Inhibition Direct Assay Kit (Fluorimetric, Epigentek; catalog no. P-4035) according to the manufacturer's instructions. Briefly, the full-length coding sequence of *LcHDA2, LcHDA6* and *LcSRT2*, were cloned into pET32a(+) (Novagen) and transformed into *Escherichia coli* strain BL21(DE3). The gene-specific primer pairs are listed in Table S1. The recombinant proteins were affinity purified using His60 Ni Superflow Resin (TransGen, Beijing). Then, 5 μg of purified proteins per well was incubated with 50 ng of substrate for 90 min at room temperature. The HDAC-deacetylated products can be recognized with a specific antibody. The ratio or amount of deacetylated products, which is proportional to the enzyme activity, can then be fluorometrically measured by reading the fluorescence in a fluorescent microplate reader (FLx800, BioTek) at 355_ex_/460_em_ and data acquired using Gen5™ Analysis Software 2.06.10. The HDAC inhibitor TSA was used to demonstrate the specificity of deacetylation activities. That the activity of the HDAC enzyme is proportional to the OD intensity was calculated with formula: HDAC Activity (RFU/min/μg) = (Sample RFU − Blank RFU)/(Protein Amount (μg)^*^ × min^**^).

## Results

### Litchi HATs

**Figure 1:**
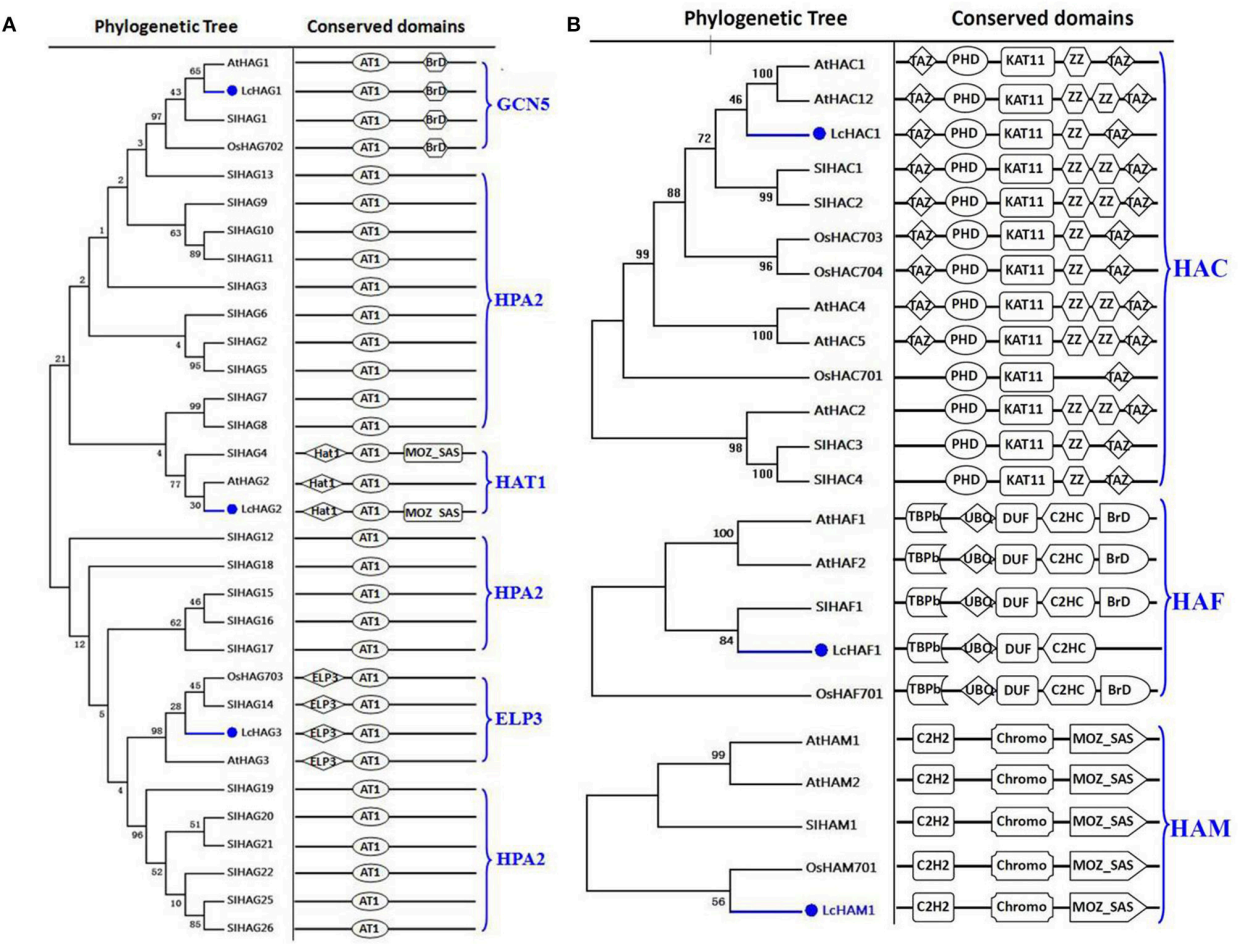
**Maximum likelihood phylogenetic trees and schematic diagrams for domain composition of HAT proteins predicted from ***Litchi chinensis (Lc), Arabidopsis thaliana (At), Oryza sativa (Os)***, and ***Solanum lycopersicon (Sl)***. (A)** Phylogenetic tree and schematic diagrams for domain composition of HAG group. AT1 (PF00583) and C-terminal BrD (PF00439) are conserved domains of GCN5-like members; N-terminal ELP (IPR006638), and C-terminal AT1 are domains of ELP3-like; N-terminal Hat1_N (PF10394) and C-terminal AT1 are motifs of HAT1-like members while the only AT1 domain is of HPA2-like proteins. **(B)** Phylogenetic trees and schematic diagrams for domain composition of HAC, HAF, and HAM groups. KAT11 (PF08214), PHD-finger (PF00628), and TAZ (PF02135) are conserved domains of HAC proteins. N-terminal kinase (PF09247) (TBPb), ubiquitin, UBQ (PF00240), zinc-finger C2HC (PF01530), and C-terminal bromo BrD (PF00439) are conserved domains of HAFs. N-terminal Chromo (PF00385), C2H2 (PF00096), and C-terminal MOZ_SAS (PF01853) domains are typical of HAMs. The phylogenetic tree was constructed based on the amino acids sequences with 1,000 bootstrapping replicates.

On the basis of homology searching, we identified six HATs in the litchi genome in this study. In order to hypothesize the phylogenic history of these HATs, we compared them with orthologs in *Arabidopsis*, rice, and tomato, noting the presence of four distinct classes: HAGs, HACs, HAFs, and HAM (Figures 1A,B). Results show that the litchi genome encodes three proteins that belong in the HAG group; of these, we found LcHAG1 to be closely related to the GCN5 clade while LcHAG3 is closely related to the ELP3 clade. Although previous work has shown that HAT1 clade proteins usually comprise an AT1 domain as well as a MOZ_SAS motif (PF01853), in this case, the combination of these domains was not seen in AtHAG2 and has thus far only been reported in tomato (SlHAG4) (Aiese et al., 2013). The results of this study show that LcHAG2 also comprises these two domains and thus can be grouped within the HAT1 clade. In addition, we found no evidence for HAGs in litchi that are related to the HPA2 clade; this lineage comprises just the AT1 domain and is regarded as specific to fungi. Only one protein (LcHAC1) belonging to the HAC class was identified in litchi. Our data show (Figure 1B) that LcHAC1 is characterized by TAZ (PF02135), PHD (PF00628), KAT11 (PF08214), ZZ, and TAZ domains (Pandey et al., 2002), while the litchi genome comprises just one HAM protein (LcHAM1) consisting of the same conserved domains, specifically an N-terminal Chromo (PF00385), C2H2 (PF00096), and a C-terminal MOZ_SAS (PF01853), as previously reported in *Arabidopsis*, rice, and tomato HAMs (Latrasse et al., 2008). Data also demonstrate that just one litchi HAF protein (LcHAF1) can be assigned to the HAF class (Figure 1B).

### Litchi HDACs

**Figure 2:**
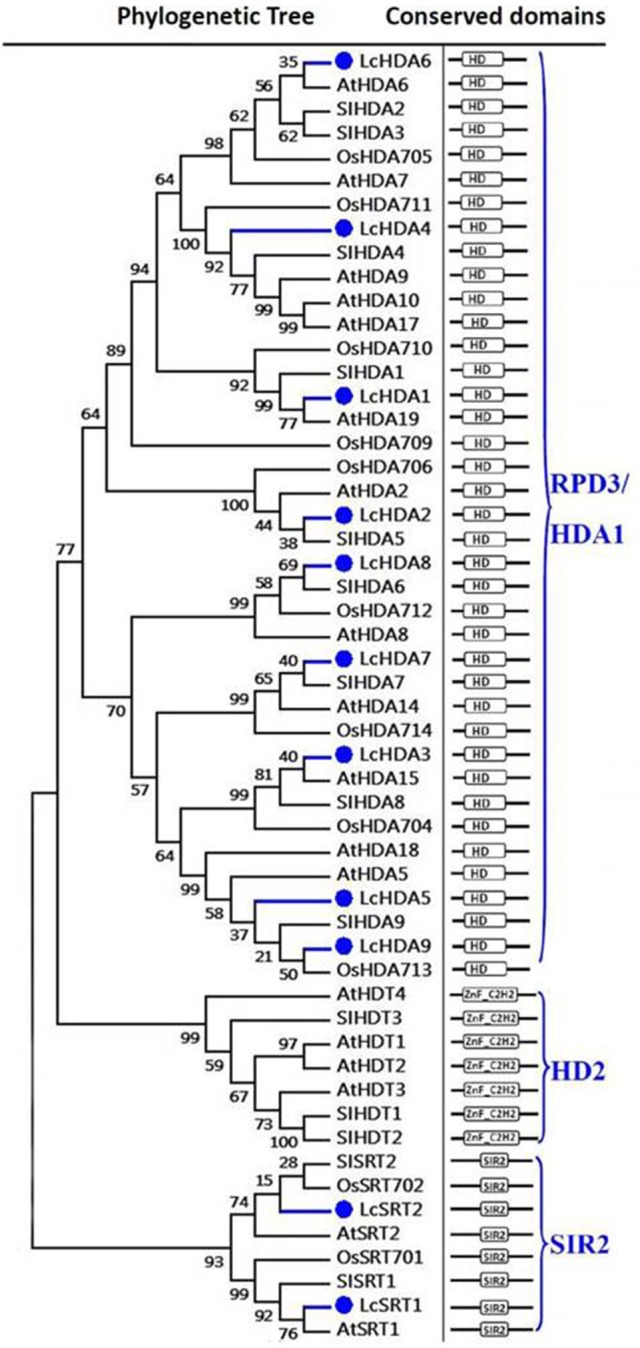
**Maximum likelihood phylogenetic trees and schematic diagrams for domain composition of HDAC proteins predicted from ***Litchi chinensis (Lc), Arabidopsis thaliana (At), Oryza sativa (Os)***, and ***Solanum lycopersicon (Sl)*****. The HDAC proteins can been divided into three groups based on their conserved domain composition. The hist_deacetyl domain, HD (PF00850) is the conserved domain of RPD3/HDA1 group. C-terminal zinc finger domain in addition to the predicted HD2 domain is the typical of HD2 group. The SIR2 group is characterized by an SIR2 domain (PF02146). The phylogenetic tree was constructed based on the amino acids sequences with 1,000 bootstrapping replicates.

Our results show that the litchi genome encodes 11 proteins that exhibit similarities with the HDAC family. As discussed above (Figure 2), plant HDACs can be classified into three subgroups (Alinsug et al., 2009); results show that nine HDACs in litchi (i.e., LcHDA1 to LcHDA9) belong to the RPD3/HDA1 subgroup characterized by a Hist_deacetyl domain (i.e., HD, PF00850). Although these HD2-type proteins are plant-specific and were first discovered in maize (Lusser et al., 1997), our results suggest that no HD2-like HDAC member is found in the litchi genome. In contrast, we identified two HDACs that can be classified within the SIR2 family, LcSRT1, and LcSRT2, which are characterized by the presence of an SIR2 domain (PF02146) (Pandey et al., 2002).

### Litchi HMTs

**Figure 3:**
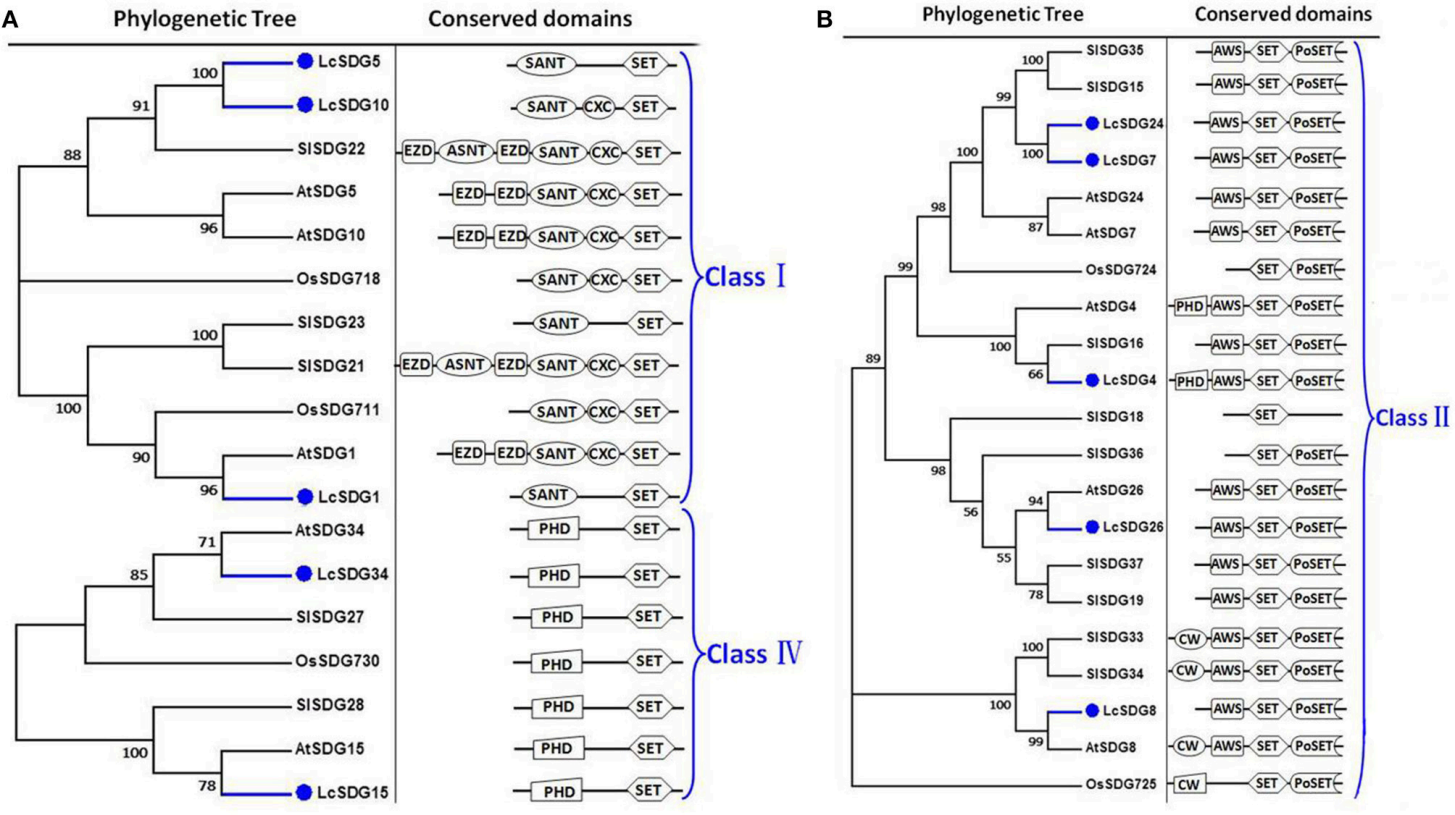
**Maximum likelihood phylogenetic trees and schematic diagrams for domain composition of class I, class II, and class IV HMT proteins predicted from ***Litchi chinensis (Lc), Arabidopsis thaliana (At)***, ***Oryza sativa (Os)***, and ***Solanum lycopersicon (Sl)***. (A)** Phylogenetic trees and schematic diagrams for domain composition of class I and class IV HMT proteins. Two EZD, SANT (SM00717), CXC (PF03638), and SET (PF00856) are conserved domains of Class I. N-terminal PHD and C-terminal SET are conserved domains of Class IV. **(B)** Phylogenetic tree and schematic diagrams for domain composition of class II HMT proteins. N-terminal AWS (SM00570), SET and Post-SET (SM00508) are conserved domains of Class II. The phylogenetic tree was constructed based on the amino acids sequences with 1,000 bootstrapping replicates.

**Figure 4:**
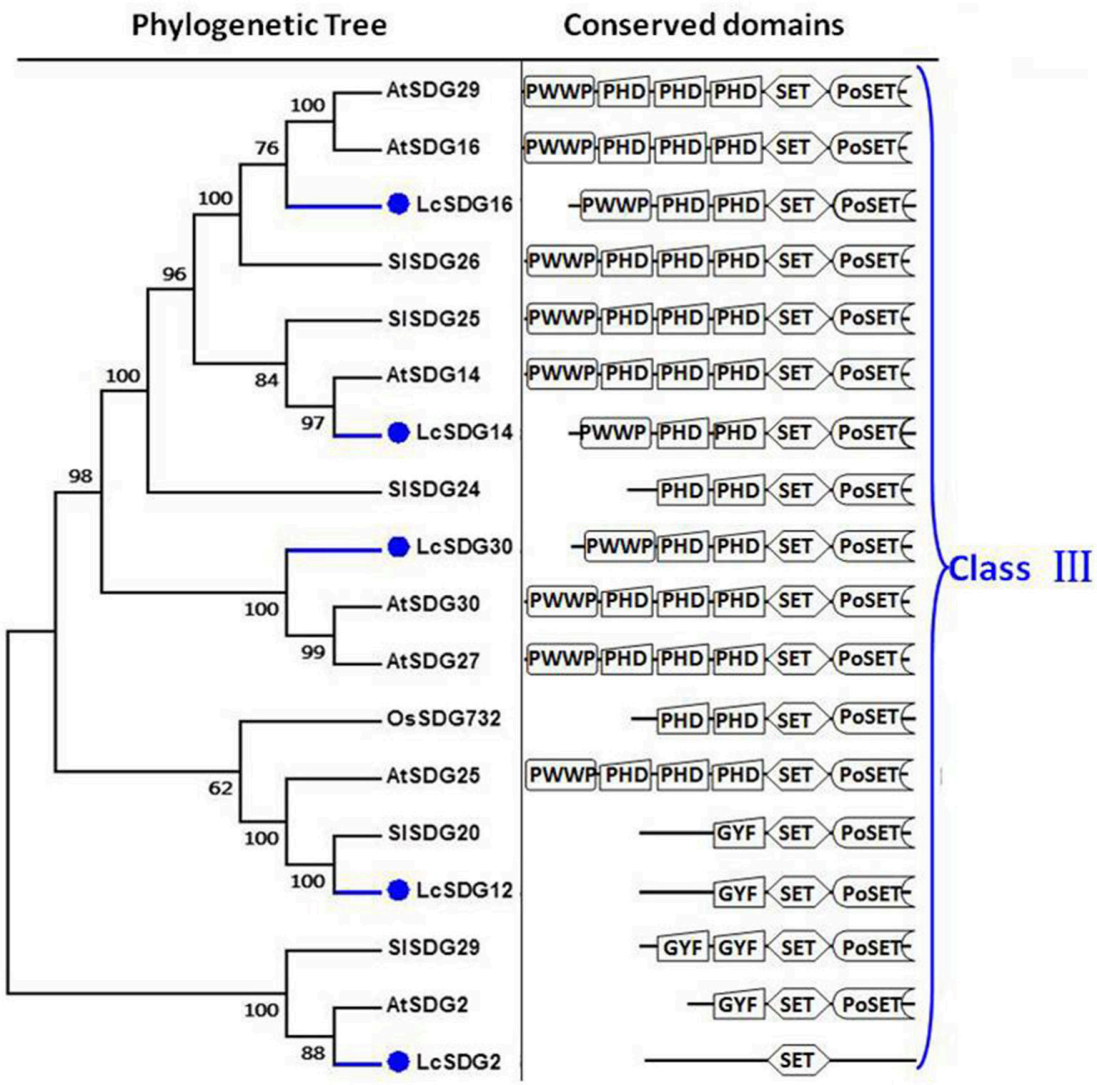
**Maximum likelihood phylogenetic trees and schematic diagrams for domain composition of class III HMT proteins predicted from ***Litchi chinensis (Lc), Arabidopsis thaliana (At), Oryza sativa (Os)***, and ***Solanum lycopersicon (Sl)*****. N-terminal PWWP (PF00855), two PHD, SET, and Post-SET are conserved domains of Class III. Some Class III proteins have N-terminal GYF (PF02213) but lack the PWWP (PF00855), two PHD domains. The phylogenetic tree was constructed based on the amino acids sequences with 1,000 bootstrapping replicates.

**Figure 5:**
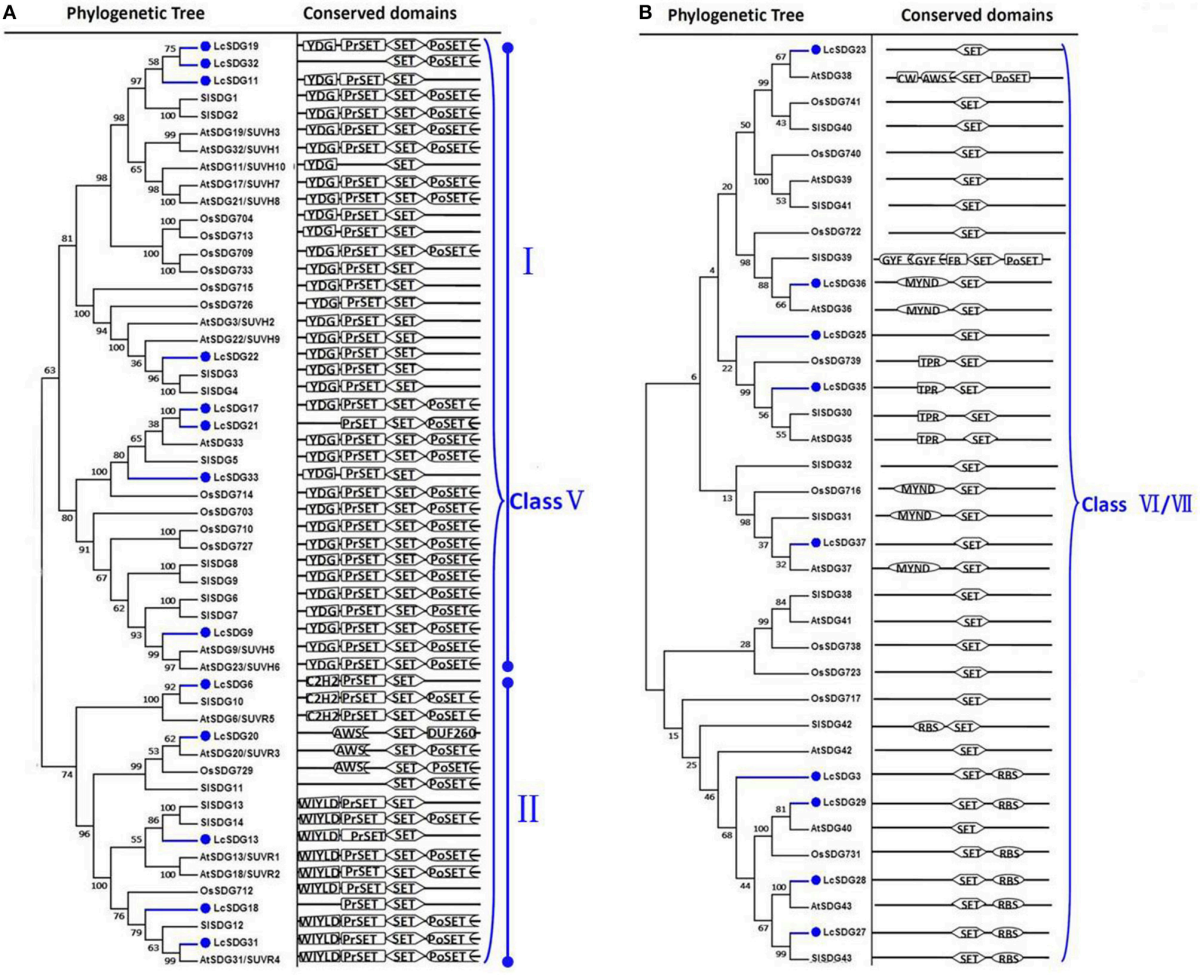
**Maximum likelihood phylogenetic trees and schematic diagrams for domain composition of class V and class VI/VII HMT proteins predicted from ***Litchi chinensis (Lc), Arabidopsis thaliana (At), Oryza sativa (Os)***, and ***Solanum lycopersicon (Sl)***. (A)** Phylogenetic tree and schematic diagrams for domain composition of class V which are further divided into two groups. N-terminal YDG (PF02182), Pre-SET, SET, and Post-SET are conserved domains of the group I of Class V; N-terminal WIYLD (PF10440), or C2H2 (PF00096) or absence of domain, Pre-SET, SET, and Post-SET are conserved domains of the group II of Class V. **(B)** Phylogenetic tree and schematic diagrams for domain composition of class VI/VII HMT proteins. Some members of these two classed comprised N-terminal AWS, or GYF, or TPR, or MYND domain, while some members included C-terminal RBS domain. The phylogenetic tree was constructed based on the amino acids sequences with 1,000 bootstrapping replicates.

As a result of the genome-wide classification applied in this study, we identified 37 SDG proteins in litchi that belong to seven classes (Springer et al., 2003). Of these, three (i.e., LcSDG1, LcSDG5, and LcSDG10) are grouped in class I, which is characterized by variable domains. Results show that although LcSDG1 clusters closely with AtSDG1, including EZD (two), SANT, CXC (PF03638), and SET domains, LcSDG1 contained just SET (PF00856) and SANT (SM00717) domains (Figure 3A). At the same time, we identified five proteins (i.e., LcSDG4, LcSDG7, LcSDG8, LcSDG24, and LcSDG26) that can be classified into class II, characterized by the presence of N-terminal AWS (SM00570), SET, and post-SET (SM00508) domains (Figure 3B). Five proteins (i.e., LcSDG2, LcSDG12, LcSDG14, LcSDG16, and LcSDG30) were also identified that belong to class III; in addition to SET and post-SET domains, most members of this class possess PWWP (PF00855) and PHD (PF00628) domains (Figure 4). Interestingly, results show that the class III protein LcSDG2 contains just the SET domain, while two proteins (LcSDG34 and LcSDG15) classified into class IV exhibit the same domain architecture (including the presence of PHD and SET domains) as their orthologs in *Arabidopsis*, rice, and tomato (Figure 3A). Results also reveal that 13 litchi SDGs can be classified into class V, and can be further divided into two main clades (Springer et al., 2003). Of these, eight proteins (i.e., LcSDG9, LcSDG11, LcSDG17, LcSDG19, LcSDG21, LcSDG22, LcSDG32, and LcSDG33) can be grouped within the first of these two clades; two of these proteins (i.e., LcSDG21 and LcSDG32) lack N-terminal YDG (PF02182) and PrSET domains, while three (i.e., LcSDG11, LcSDG22, and LcSDG33) are missing the C-terminal PoSET domain (Figure 5A). Results also show that five proteins can be classified within the second class V clade, characterized by N-terminal WIYLD (PF10440), C2H2 (PF00096), or AWS (SM00570) domains. Interestingly, we note that an a N-terminal DUF260 (PF03195) domain replaces the PoSET domain in LcSDG20 (Figure 5A), while nine proteins (i.e., LcSDG3, LcSDG23, LcSDG25, LcSDG27, LcSDG28, LcSDG29, LcSDG35, LcSDG36, and LcSDG37) are clustered within classes VI and VII. Data show that some members of these classes possess N-terminal AWS, GYF, TPR, or MYND domains, while others have C-terminal RBS domains (Figure 5B). A total of 11 litchi PRMTs were identified in this study (i.e., LcPRMT1 to SlPRMT11) (Figure S1).

As discussed above, conserved domains in LcSDG2 and LcSDG20 can be distinguished from their orthologs in other plants within the same group (Figures 4, 5A). We therefore further analyzed LcSDG2 and LcSDG20 phylogenetically using SET proteins from *F. vesca, C. sinensis*, and *V. vinifera* to test whether or not the domain structures of these two proteins are unique to litchi. Results show (Figure S2) that VvSET3, CsSET18, and FvSET19, the latter of which also contains just the SET domain, were grouped together with LcSDG2 and AtSDG2. This suggests that some class III SDG proteins which just contain the SET domain are also found in other plants. Indeed, CsSET26, FvSET17, FvSET11, and VvSET5, all of which lack a DUF260 domain, also cluster with LcSDG20 and AtSDG20; this suggests that the class V member LcSDG20, which includes a DUF260 domain, may perform a specific function in litchi (Figure S2).

### Litchi HDMs

**Figure 6:**
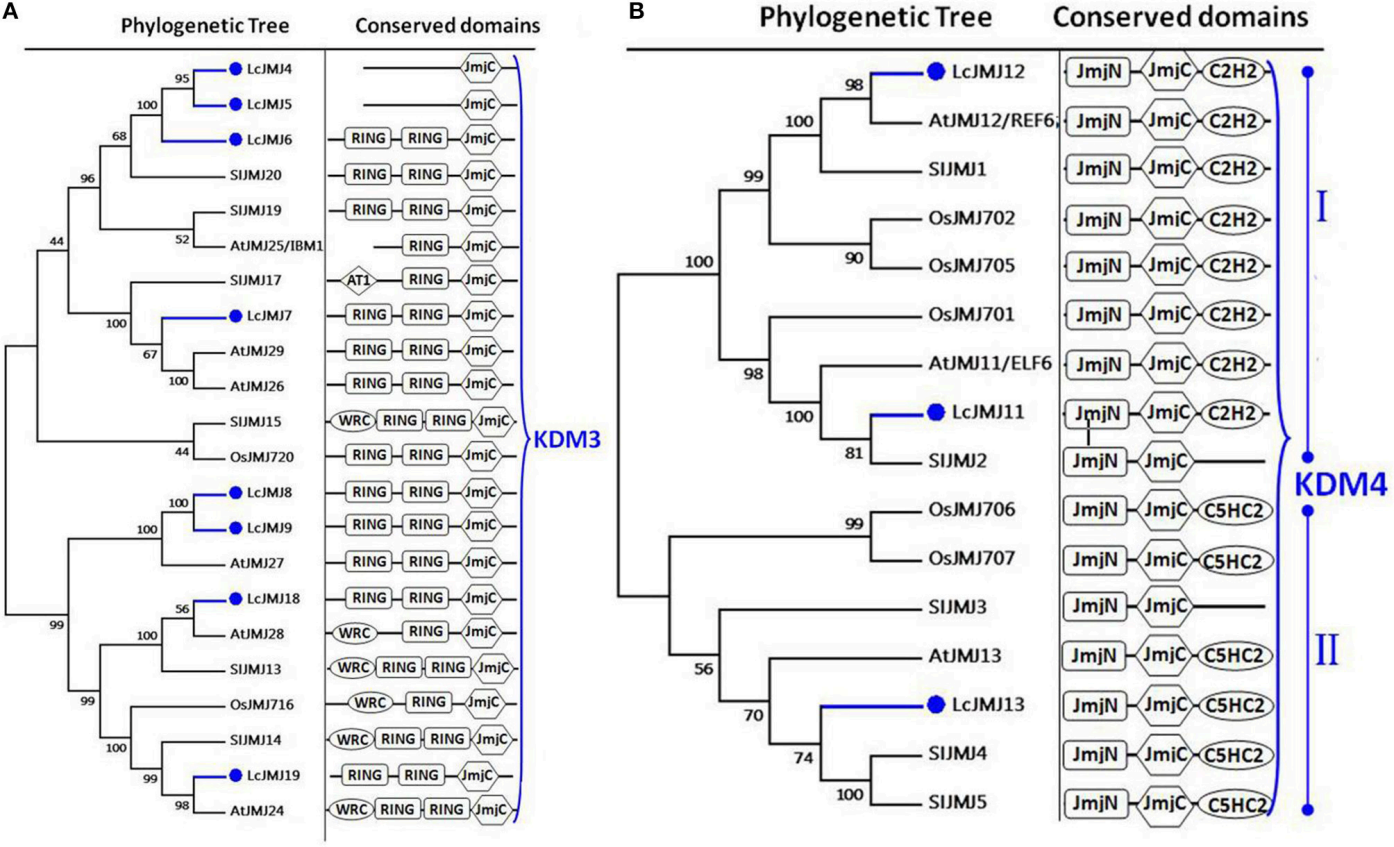
**Maximum likelihood phylogenetic trees and schematic diagrams for domain composition of KDM3 class and KDM4 class HDM proteins predicted from ***Litchi chinensis (Lc), Arabidopsis thaliana (At), Oryza sativa (Os)*** and ***Solanum lycopersicon (Sl)***. (A)** Phylogenetic tree and schematic diagrams for domain composition of KDM3 class HDM proteins. RING-finger (IPR001841) and JmjC (PF02373) are conserved domains of KDM3 class. **(B)** Phylogenetic tree and schematic diagrams for domain composition of KDM4 class proteins which are further divided into two groups. N-terminal JmjN (PF02375) and JmjC, and C-terminal C5HC2 (PF02928) (subgroup I) or C2H2 (PF00096) (subgroup II) domains are conserved domains of KDM4 proteins. The phylogenetic tree was constructed based on the amino acids sequences with 1,000 bootstrapping replicates.

We identified 20 litchi proteins that belong to the JMJ family of HDMs. Indeed, consistent with previous reports, the proteins that combined with members of the JMJ family in *Arabidopsis*, rice, and tomato were grouped into five classes (Lu et al., 2008). Eight proteins (i.e., LcJMJ4 to LcJMJ9, LcJMJ18, and LcJMJ19) were assigned to the KDM3 class, while two members (i.e., LcJMJ4 and LcJMJ5) that lack the N-terminal RING finger (IPR001841) domain were classified within the typical JMJ-only class (Figure 6A). At the same time, our results split the members of the KDM4 class into two main clades comprising two (i.e., LcJMJ11 and LcJMJ12) and one (LcJMJ13) litchi proteins, respectively. Members of the first clade possess the N-terminal C2HC2-domain while members of the second have the N-terminal C5HC2 domain (Lu et al., 2008; Figure 6B).

**Figure 7:**
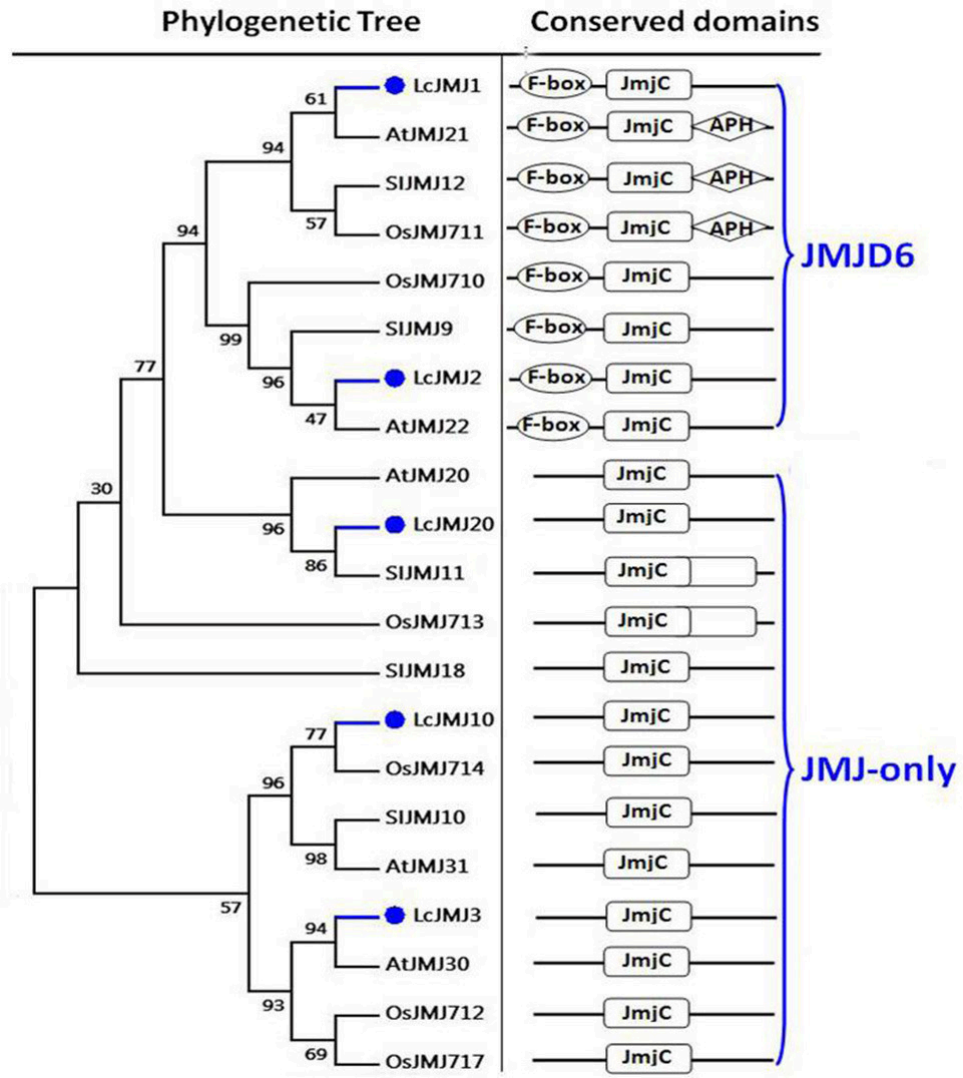
**Maximum likelihood phylogenetic trees and schematic diagrams for domain composition of JMJD6 class and JMJ-only class HDM proteins predicted from ***Litchi chinensis (Lc), Arabidopsis thaliana (At), Oryza sativa (Os)***, and ***Solanum lycopersicon (Sl)*****. N-terminal F-box (PF00646), and C-terminal JmjC are conserved domains of JMJD6 class; JmjC domain is the conserved domain of the JMJs-only class demethylases. The phylogenetic tree was constructed based on the amino acids sequences with 1,000 bootstrapping replicates.

Results show that members of the KDM5 class can also be divided into two main clades, with three proteins (i.e., LcJMJ14–LcJMJ16) grouped within the first, and one (LcJMJ17) within the second (Figure S3). In contrast to orthologs grouped within the first clade, results show that the C5HC2 (PF02928) domain was replaced with the PLU-1 (PF08429) domain in LcJMJ17 when compared to orthologs in the second clade. At the same time, two (i.e., LcJMJ1 and LcJMJ2) and three proteins (i.e., LcJMJ3, LcJMJ10, and LcJMJ20), respectively, were found to be related to the JMJD6 and JMJ-only classes, with the former characterized by a N-terminal F-box (PF00646) domain to the exclusion of the JmjC domain. Results show that members of the JMJ-only class just possess the JmjC domain (Figure 7; Lu et al., 2008). Furthermore, we found two members that belong to KDM1/LSD1-type HDMs (Figure S4).

As discussed above, the existence of different LcJMJ4/5 and LcJMJ17 domains compared with orthologs from other plants within the same clade encouraged us to further investigate these three LcJMJs. Thus, we analyzed LcJMJ4/5 and LcJMJ17 phylogenetically (Figure S5) alongside JmjC-containing proteins from *F. vesca, C. sinensis*, and *V. vinifera*. The results of this study show that LcJMJ4/5 clusters with homologs that either do (i.e., CsJmjC13, VvJmjC9, and FvJmjC21) or do not (i.e., CsJmjC7, FvJmjC12, and CsJmjC14) possess an N-terminal RING finger domain. This outcome suggests that some KDM3 class members did not acquire the N-terminal RING finger domain during their evolution. At the same time, LcJMJ17 clusters with CsJmjC8, FvJmjC7, and VvJmjC6, which all possess the C5HC2 domain. This result further suggests that the KDM5 class gene LcJMJ17 may not have acquired the C5HC2 domain during litchi evolution.

### HM expression profiles during litchi fruit abscission

**Figure 8:**
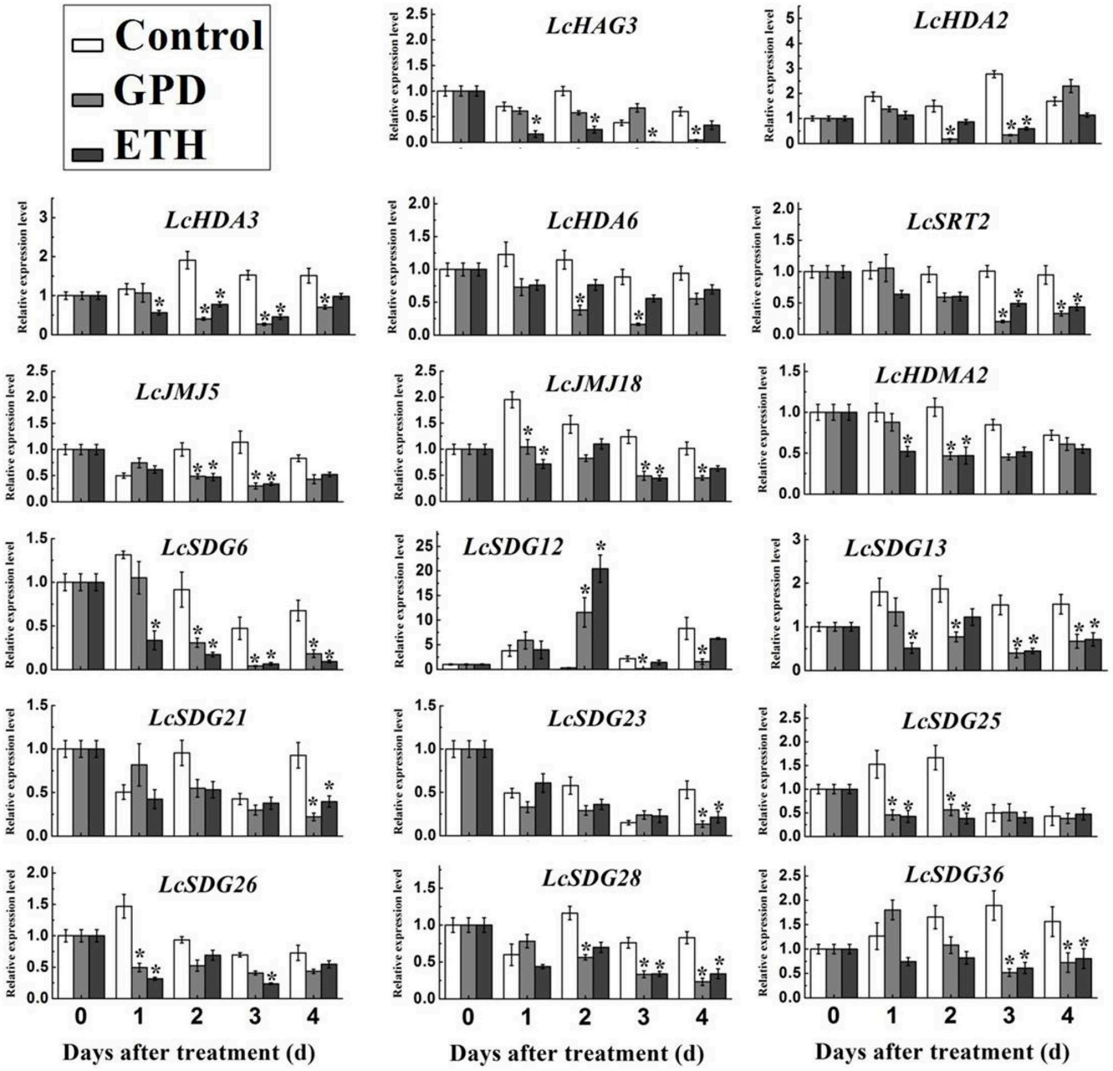
**Expression level of HMs in AZ cells during fruit abscission in litchi**. GPD indicated Girdling Plus Defoliation (GPD) treatment and ETH indicated ethephon (ETH) treatment. qRT-PCR analysis was used. *LcEF-1a* was used as an internal control. Data shown are means ± SD. One-way ANOVA (Tukey-Kramer test) analysis was performed, and statistically significant differences (*P* < 0.05) were indicated by asterisks.

The occurrence of fruit abscission on a massive scale is a limiting factor for the litchi industry; previous studies have shown that a range of genes are associated with this process (Li et al., 2015a,b). Because the process of histone modification is known to play a crucial role in the regulation of gene expression, we investigated the expression profiles of HM genes during the abscission of litchi fruitlets. Thus, fruit-bearing shoots were subjected to one of two abscission-inducing treatments: GPD or ETH (Li et al., 2015a,b). Results show that transcription of 14 out of 87 litchi HM genes could not be detected; these were therefore omitted from this investigation. However, as shown in Figure 8 and Figure S6, 17 HM genes exhibited significantly altered patterns of expression when treated with either GPD or ETH, compared with the control. Identified HM genes include one HAT (i.e., *LcHAG3*), four HDACs (i.e., *LcHDA2, LcHDA3, LcHDA6*, and *LcSRT2*), nine HMTs (i.e., *LcSDG6, LcSDG12, LcSDG13, LcSDG21, LcSDG23, LcSDG25, LcSDG26, LcSDG28*, and *LcSDG36*), and three HDMs (i.e., *LcHDM2, LcJMJ15*, and *LcJMJ18*). The results of our qRT-PCR analysis reveal that the expression of *LsSDG12* was significantly induced on the second day following GPD and EHT treatments, while the other 16 HM genes were significantly repressed as the result of treatments (Figure 8). These data suggest that a number of HM genes may be involved in litchi fruit abscission.

**Figure 9:**
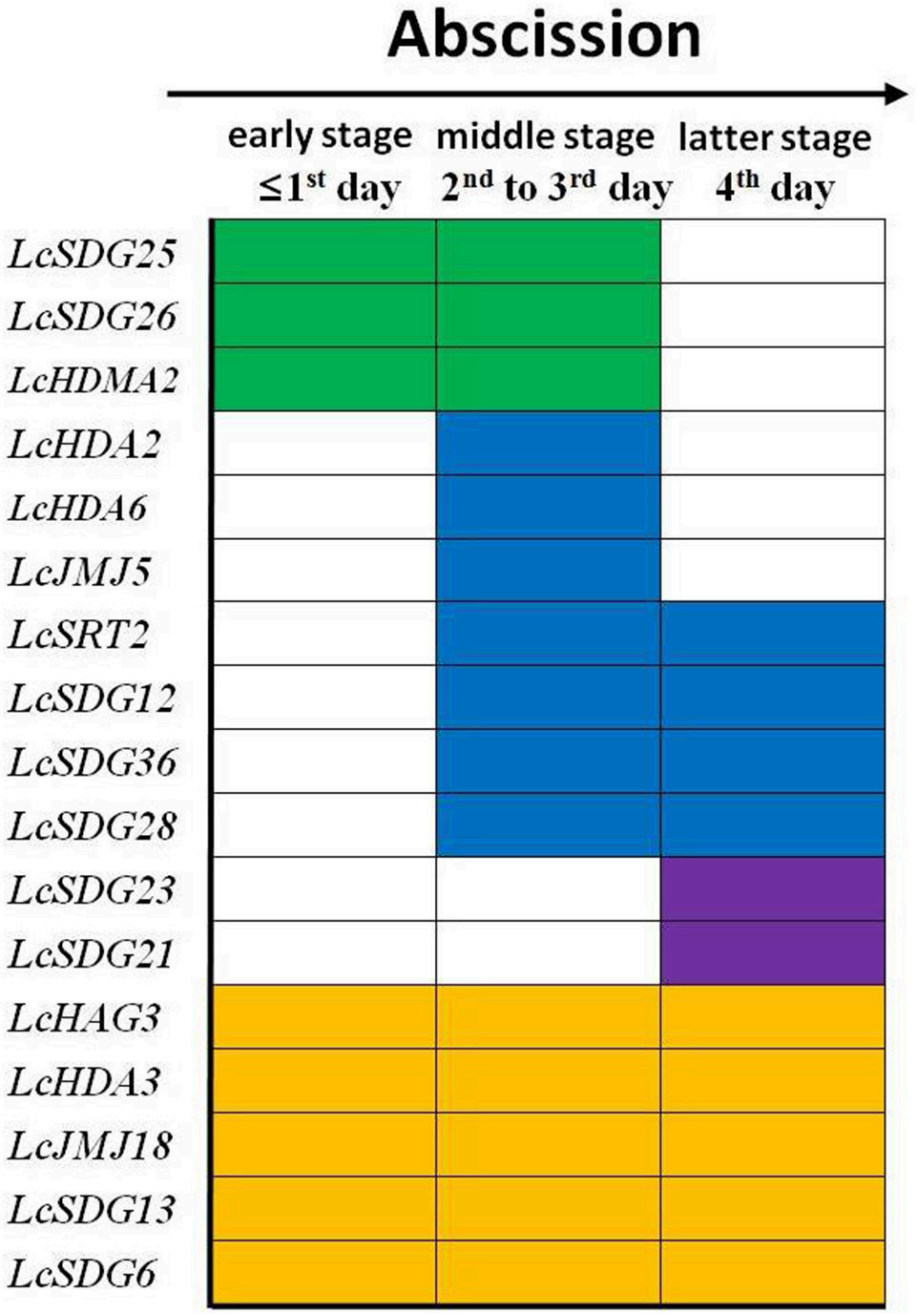
**Patterns of differently expressed HMs in response to fruit abscission in litchi**. The early stage responsive genes were shaded in green (from 1st to 3rd day); the middle stage responsive genes were shaded in blue (from 2nd to 3rd or to 4th day); the latter stage responsive genes were shaded in purple (4th day); the full stage responsive genes were shaded in yellow (from 1st to 4th day).

Further interrogation of these results show that the 17 HM genes identified in this study can be classified into four groups (Figure 9). These comprise a set of early-stage response genes, including *LcSDG25* and *LcSDG26*, that are expressed within 1 day of treatment. This result suggests that these two genes may play a role in the early stages of litchi fruit abscission. The second group comprises middle-stage response genes that are expressed from the second day after treatment and includes *LcHDA2, LcHDA6, LcHDM2, LcSDG12, LcJMJ15, LcSRT2, LcSDG36*, and *LcSDG28*. This result suggests that these genes may function in the middle and latter stages of fruit abscission in litchi. The third group we identified comprises later-stage response genes that are expressed from the fourth day following treatment and includes *LcSDG21* and *LcSDG23*. This result suggests that these genes may be more specific to the later stages of fruit abscission. Finally, the fourth group we identified comprises a set of full-stage response genes that are expressed over the period between the first day and fourth day following treatment and includes *LcHAG3, LcHDA3, LcJMJ18, LcSDG13*, and *LcSDG6*. This result suggests that these genes may be required over the whole of the fruit abscission process. We therefore propose that the 17 members of this set of HMs play a range of diverse roles in regulating litchi fruit abscission.

### An *in vitro* assay of HDAC activity

**Figure 10:**
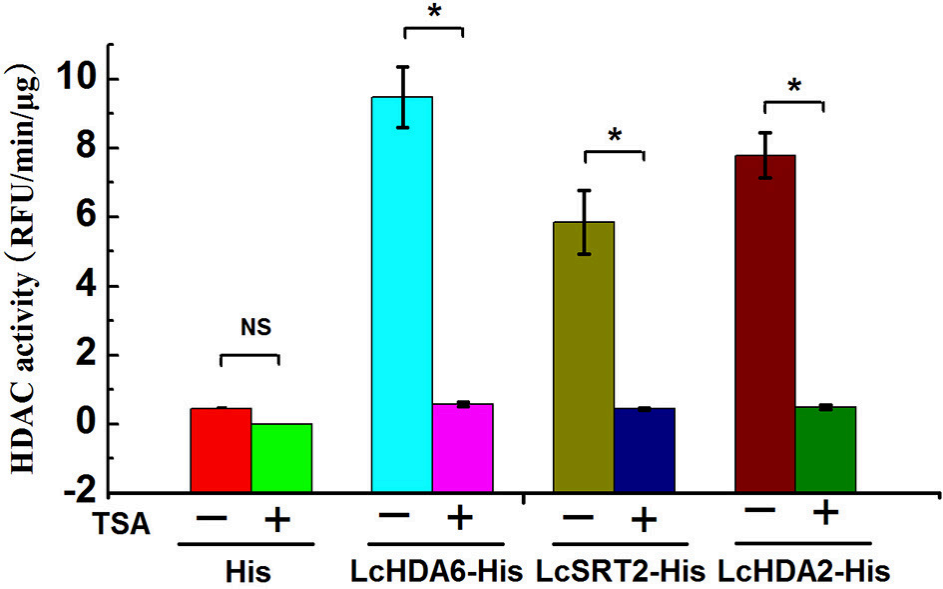
**In ***vitro*** assay of HDAC activity of LcHDA2, LcHDA6, and LcSRT2**. His protein alone was used as control, TSA was added to samples to demonstrate the specificity of deacetylase activities. − and + represent the absence or presence of TSA, respectively. The HDAC activity was expressed by relative fluorescent intensity using a fluorescence microplate reader at 355_EX_/460_EM_. Data shown are means ± SD. One-way ANOVA (Tukey-Kramer test) analysis was performed, and statistically significant differences (*P* < 0.05) were indicated by asterisks.

In order to test whether or not differentially regulated LcHDACs exhibit HDAC activity during fruit abscission, we purified the His-tagged LcHDA2, LcHDA6, and LcSRT2 proteins that are expressed in *E. coli* strain BL21(DE3) and measured their HDAC activity using fluorometric assays. We used His protein as a control for this analysis and added the HDAC inhibitor trichostatin A (TSA) to demonstrate the specificity of deacetylase activities. The results of this analysis show that LcHDA2, LcHDA6, and LcSRT2 proteins all exhibit strong HDAC activity compared to the control and were significantly inhibited by TSA (Figure 10).

## Discussion

### Identification of litchi HMs

Previous work has shown that HMs play critical roles in a number of growth and development processes by regulating gene expression. Thus, an increasing amount of attention has been focused on the identification and characterization of these modifiers in various plant species, including *Arabidopsis*, rice, and tomato. Because sequence-based searches have proved effective for the identification of candidate genes in new plant genomes (Aiese et al., 2013; Gu et al., 2016), we systematically identified 87 HMs within the litchi genome, including six HATs, 11 HDACs, 48 HMTs, and 22 HDMs. Notably, just six HATs were identified in the litchi genome, while 12 and 32 are present in *Arabidopsis* and tomato, respectively. This result might indicate that a lower level of HAT gene duplication took place during litchi evolution.

### The possible roles of litchi HMs

Although histone modifications are thought to play important roles in a variety of growth and development processes and stress responses, the functions of HMs in litchi remain unknown. A number of previous studies have demonstrated that ortholog analysis is a viable approach for predicting the unknown functions of comparable genes in different species that have evolved as the result of speciation events. Because they are derived from a single gene in the last common ancestor of two or more species, orthologs frequently share the same functions in newly evolved taxa (Das et al., 2016). Thus, in order to predict the potential biological roles of litchi HMs, we reviewed the known examples of these enzymes that have been functionally characterized in other plants and identified the closest orthologs to litchi HMs based on phylogenetic analysis (Table 1). For example, AtHAG1 is an important regulator that performs essential roles in a number of developmental processes, including meristem function, cell differentiation, leaf and floral organ ogenesis, and responses to light and cold (Benhamed et al., 2006; Kornet and Scheres, 2009; Servet et al., 2010). Our phylogenetic analysis demonstrates that LcHAG1 is the closest homolog of AtHAG1 (Figure 1A), and suggests that the two orthologs likely performed the same function. Previous work has shown that AtHAG3 is involved in transcription elongation, cell proliferation, leaf axis development, seedling growth, and plant responses to UV-B (Nelissen et al., 2005, 2010; Kojima et al., 2011; Fina and Casati, 2015). Interestingly, our results suggest that LcHAG3 in litchi appears to be closely related to AtHAG3 (Figure 1A), suggesting that the two likely perform the same functions. As a member of the HAC family of HATs, LcHAC1 also shares sequence similarity with AtHAC1 and AtHAC12, both of which are linked to flowering (Deng et al., 2007; Han et al., 2007; Li et al., 2014). Similarly, LcHAF1 is the closest homolog of AtHAF2, known to be involved in responses to light (Benhamed et al., 2006). Our results show that LcHAM1 is the closest homolog of AtHAM1/2, which acts redundantly in male and female gametophyte development as well as in flowering (Latrasse et al., 2008; Xiao et al., 2013). This suggests that LcHAM1 likely performs the same control function in the floral transition.

**Table 1:** **Histone midifiers functionally characterized in plants and their closest orthologs in litchi**.

**HMs**	**Gene name**	**Biological role**	**Close homology in litchi**	**References**
HATs	AtHAG1	Pleiotrpic effects on development, responses to environmental conditions	LcHAG1	Benhamed et al., 2006; Kornet and Scheres, 2009; Servet et al., 2010
	AtHAG3	Transcription elongation, cell proliferation, leaf axis development, seedling, and root growth and UV-B responses	LcHAG3	Nelissen et al., 2005, 2010; Kojima et al., 2011; Fina and Casati, 2015
	AtHAC1	Flowering; fertility; ethylene signaling	LcHAC1	Deng et al., 2007; Han et al., 2007; Li et al., 2014
	AtHAC12	Flowering	LcHAC1	Han et al., 2007
	AtHAF2	Light response	LcHAF1	Benhamed et al., 2006
	AtHAM1/2	Flowering; gametophyte development	LcHAM1	Latrasse et al., 2008; Xiao et al., 2013
HDACs	AtHDA6	Flowering; seed maturation; circadian transcription; leaf development; embryonic properties;chromatin silencing; jasmonate and ethylene signaling; abiotic stress response; freezing tolerance	LcHDA6	Probst et al., 2004; Tanaka et al., 2008; Wu et al., 2008; Chen and Wu, 2010; Gu et al., 2011; To et al., 2011a,b; Yu et al., 2011; Zhu et al., 2011; Liu et al., 2012; Luo et al., 2012a,b; Wang L. et al., 2013; Wang Z. et al., 2013
	AtHDA19	Circadian transcription; floral organ identity; seed dormancy; seed maturation; abiotic stress response; embryonic properties; pathogen response	LcHDA1	Zhou et al., 2005, 2013; Tanaka et al., 2008; Chen and Wu, 2010; Krogan et al., 2012; Wang L. et al., 2013; Wang Z. et al., 2013
	AtHDA15	Chlorophyll biosynthesis and photosynthesis	LcHDA3	Liu et al., 2013
	AtHDA5	Flowering	LcHDA5	Luo et al., 2015
	AtSRT1/2	Mitochondrial energy metabolism; basal defense	LcSRT1/2	Wang et al., 2010; Konig et al., 2014
	OsSRT701	Cell death and transposon repression	LcSRT1/2	Zhong et al., 2013
HMTs	AtSDG4	Pollen tube growth	LcSDG4	Cartagena et al., 2008
	AtSDG26	Flowering	LcSDG26	Xu et al., 2008
	AtSDG8	Flowering; shoot branching; ovule and anther development; defense response; repression of the embryonic program	LcSDG8	Dong et al., 2008; Xu et al., 2008; Cazzonelli et al., 2009; Grini et al., 2009; Berr et al., 2010; Tang et al., 2012
	AtSDG27	Root development; dehydration stress responses	LcSDG30	Ding et al., 2011a,b; Napsucialy-Mendivil et al., 2014
	AtSDG2	Flowering; sporophyte, and gametophyte development	LcSDG2	Berr et al., 2010; Guo et al., 2010; Yun et al., 2012
	OsSDG714	Retrotransposon repression; macro trichome development	LcSDG33	Ding et al., 2007; Qin et al., 2010
	AtPRMT3	Ribosome biogenesis	LcPRMT3	Hang et al., 2014
HDMs	AtHDMA2/3	Flowering; seed dormancy	LcHDMA1/2	Jiang et al., 2007; Zhao et al., 2015
	AtJMJ20/22	Seed germination	LcJMJ2/20	Cho et al., 2012
	AtJMJ30	Flowering	LcJMJ3	Gan et al., 2014
	AtJMJ24	RNA silencing; proteasomal degradation	LcJMJ19	Deng et al., 2015, 2016
	AtJMJ11/12	Flowering; BR response	LcJMJ11/12	Noh et al., 2004; Yu et al., 2008

Indeed, out of all known plant HDACs, AtHDA6 has been functionally characterized to the greatest extent. Previous research has shown that this HDAC performs a number of essential roles in flowering, seed maturation, circadian transcription, leaf development, embryonic function, gene silencing, jasmonate (JA) and ethylene signaling, abiotic stress responses, and tolerance to freezing (Probst et al., 2004; Tanaka et al., 2008; Wu et al., 2008; Chen and Wu, 2010; Gu et al., 2011; To et al., 2011a,b; Yu et al., 2011; Zhu et al., 2011; Liu et al., 2012; Luo et al., 2012a,b; Wang Z. et al., 2013). Because phylogenetic analysis shows that LcHDA6 is the closest homolog of AtHDA6, the two likely perform the same functions. In addition to AtHDA6, AtHDA19 has also been well-characterized in *Arabidopsis* and is known to play a critical role in circadian transcription, the identity of floral organs, seed dormancy and maturation, embryonic functions, and abiotic and biotic stress responses (Zhou et al., 2005, 2013; Tanaka et al., 2008; Chen and Wu, 2010; Krogan et al., 2012; Wang L. et al., 2013; Wang Z. et al., 2013). The results of this study show that LcHDA1 is the closest ortholog to AtHDA19 (Figure 2). AtHDA15 has recently been shown to be a key component involved in photomorphogenesis as it directly represses chlorophyll biosynthesis and photosynthetic genes via association with phytochrome-interacting-factor 3 (PIF3) in etiolated *Arabidopsis* seedlings (Liu et al., 2013). Because our phylogenetic analysis suggests that LcHDA3 is the closest homolog of AtHDA15, the former is likely to also play a role in light signaling pathways.

HMT functions have been well-characterized in a number of model plants. For example, previous research has shown that AtSDG8 performs a number of key roles in growth and development processes, including flowering, shoot branching, ovule and anther development, and repression of the embryonic program. At the same time, AtSDG8 also functions as an important regulator against pathogen attacks (Dong et al., 2008; Xu et al., 2008; Cazzonelli et al., 2009; Grini et al., 2009; Berr et al., 2010; Tang et al., 2012). Because our results show that LcSDG8 is the closest homolog of AtSDG8, it likely performs the same functions as its counterpart in *Arabidopsis*. The biological roles of other SDG members in *Arabidopsis* and rice alongside their closest homologs in litchi are summarized in Table 1. Out of these HDMs, several, including AtHDMA2/3, AtJMJ30, and AtJMJ11/12, share the function of regulating flowering time (Noh et al., 2004; Jiang et al., 2007; Gan et al., 2014). However, whether or not their closest homologs in litchi have a similar role will require further research.

### Regulation of litchi fruit abscission by HMs

No research has been published to date on the role of HMs in organ abscission. In this study, we show that 17 HMs are significantly altered during fruit abscission under two fruit promoting treatments (Figure 8). At this point, however, we are unable to speculate as to which of these HMs have the closest relationship to fruit abscission as research on expression patterns represents just a starting point in this field and not enough information is currently available to determine the HMs involved in fruit abscission. A good deal of evidence has nevertheless been presented that hormones are effectors of abscission and that the balance between auxin and ethylene is of particular importance. We have also observed an ethylene emission peak before fruit abscission in litchi in our earlier work, and have demonstrated that fruit abscission is accelerated following ethylene treatment. In addition, with the exception of genes related to ethylene, those involved in auxin, ABA, and JA metabolism and signaling are also known to be differentially regulated during litchi fruit abscission (Li et al., 2015a,b). Thus, it is also possible that if HMs are capable of regulating these hormones then they are also likely to be involved in litchi fruit abscission. In *Arabidopsis*, for example, HDA6 is required as part of the JA response, as well as for senescence and flowering.

Similarly, expression of the JA-responsive genes, *PDF1.2, VSP2, JIN1*, and *ERF1*, were also down-regulated in the *Arabidopsis* HDA6 mutant, *axe1–5*, as well as in HDA6-RNA interfering (HDA6-RNAi) plants (Wu et al., 2008). We also know that the ethylene-stabilized transcription factors ETHYLENE IN-SENSITIVE 3 (EIN3) and its closest homolog EIN3-LIKE 1 (EIL1) integrate ethylene and JA signaling in the regulation of gene expression, root development, and necrotrophic pathogen defense. As part of this process, HDA6 has been shown to be a corepressor that down-regulates EIN3/EIL1-dependent transcription (Zhu et al., 2011). Furthermore, HDA6 is involved in regulating the ABA signal pathway as HDA6 mutant plants have phenotypes that are more sensitive to ABA (Chen et al., 2010). Our phylogenetic analysis shows that LcHDA6 is the closest homolog of AtHDA6, which also suggests that LcHDA6 is a likely candidate for the regulation of fruit abscission in litchi, possibly via the mediation of ethylene and the ABA response in the same way as AtHDA6 (Figure 2).

In *Arabidopsis* amongst other plants, only SDG8 has been shown to play a key role in regulating responses to pathogen attacks via mediation of ethylene response factor ERF1 transcription (Berr et al., 2010). Although AtSDG8 does not appear to have a close relationship with litchi SDG genes which are differentially regulated during abscission, given that of nine of the 17 HMs identified in this study are SDG genes, it can be hypothesized that histone methylation is likely to play a role in regulating fruit abscission in litchi.

In conclusion, here we provided an overview of the HMs encoded in the litchi genome and make a number of predictions for their likely roles based on phylogenetic analyses. We also screened 17 HMs which may act as candidate genes involved in fruit abscission. Further studies, including biological experiments, will be required to confirm the functions of these genes as well as to explore the mechanisms that underlie responses to fruit abscission in litchi.

## Author contributions

MZ, JL, and MP contributed to designing the experiments. MZ, MP, PY, and CL performed the experiments, collected, and analyzed the data. MZ, JL, MP, XL, and RX contributed to data interpretation and preparation of the manuscript. All authors reviewed the manuscript.

### Conflict of interest statement

The authors declare that the research was conducted in the absence of any commercial or financial relationships that could be construed as a potential conflict of interest.

## Supplementary material

Figure S1 **Maximum likelihood phylogenetic tree of PRMT type HMT proteins predicted from ***Litchi chinensis*** (***Lc***), ***Arabidopsis thaliana*** (***At***), ***Oryza sativa*** (***Os***), and ***Solanum lycopersicon*** (***Sl***)**. The phylogenetic tree was constructed based on the amino acids sequences with 1,000 bootstrapping replicates.

Figure S2 **Maximum likelihood phylogenetic tree of SET domain-containing HMT proteins predicted from ***Litchi chinensis*** (***Lc***), ***Fragaria vesca*** (***Fa***), ***Citrus sinensis*** (***Cs***), and ***Vitis vinifera*** (***Vv***)**. The phylogenetic tree was constructed based on the amino acids sequences with 1,000 bootstrapping replicates.

Figure S3 **Maximum likelihood phylogenetic tree of KDM5 class HDM proteins predicted from ***Litchi chinensis*** (***Lc***), ***Arabidopsis thaliana*** (***At***), ***Oryza sativa*** (***Os***), and ***Solanum lycopersicon*** (***Sl***)**. JmjN, BRIGHT/ARID (PF01388), JmjC, C5HC2, FYRN (PF05964), and FYRC (PF05965) are conserved domains of the subgroup I of KDM5; JmjN, BRT (PF01388), PHD (PF00628), JmJC, and C5HC2 are conserved domains of the subgroup II of class KDM5. The phylogenetic tree was constructed based on the amino acids sequences with 1,000 bootstrapping replicates.

Figure S4 **Maximum likelihood phylogenetic tree and schematic diagrams for domain composition of HDMA type HDM proteins predicted from ***Litchi chinensis*** (***Lc***), ***Arabidopsis thaliana*** (***At***), ***Oryza sativa*** (***Os***), and ***Solanum lycopersicon*** (***Sl***)**. N-terminal SWIRM (PF04433), NADB8, and C-terminal Amino_Oxidase domain (AOD) (PF01593) are conserved domains of HDMA type HDM proteins.The phylogenetic tree was constructed based on the amino acids sequences with 1,000 bootstrapping replicates.

Figure S5 **Maximum likelihood phylogenetic tree of JmjC domain-containing proteins predicted from ***Litchi chinensis*** (***Lc***), ***Fragaria vesca*** (***Fa***), ***Citrus sinensis*** (***Cs***), and ***Vitis vinifera*** (***Vv***)**. The phylogenetic tree was constructed based on the amino acids sequences with 1,000 bootstrapping replicates.

Figure S6 **Expression level of HMs in AZ cells during fruit abscission in litchi**. GPD indicated Girdling Plus Defoliation (GPD) treatment and ETH indicated ethephon (ETH) treatment. qRT-PCR analysis was used. *LcEF-1a* was used as an internal control. Data shown are means ± SD. One-way ANOVA (Tukey-Kramer test) analysis was performed, and statistically significant differences (*P* < 0.05) were indicated by asterisks.

File S1 **Protein sequences used in this study**.

Table S1 **Primers used in this study**.
