# Supplementary figures and images for: Genome-Wide Identification of Histone Modifiers and Their Expression Patterns during Fruit Abscission in Litchi
Source: Front Plant Sci. 2017 Apr 27;8:639. doi: 10.3389/fpls.2017.00639 (PMC5406457; doi:10.3389/fpls.2017.00639)

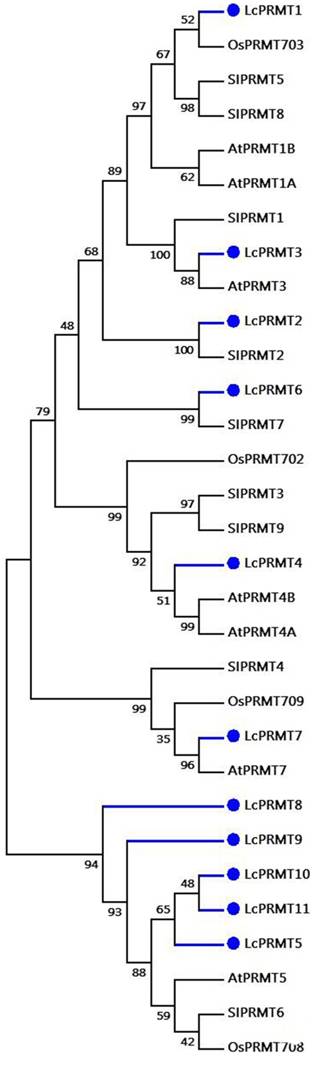

Supplement: Figure S1 — Maximum likelihood phylogenetic tree of PRMT type HMT proteins predicted from Litchi chinensis (Lc), Arabidopsis thaliana (At), Oryza sativa (Os), and Solanum lycopersicon (Sl). The phylogenetic tree was constructed based on the amino acids sequences with 1,000 bootstrapping replicates. [file Image1.JPEG]

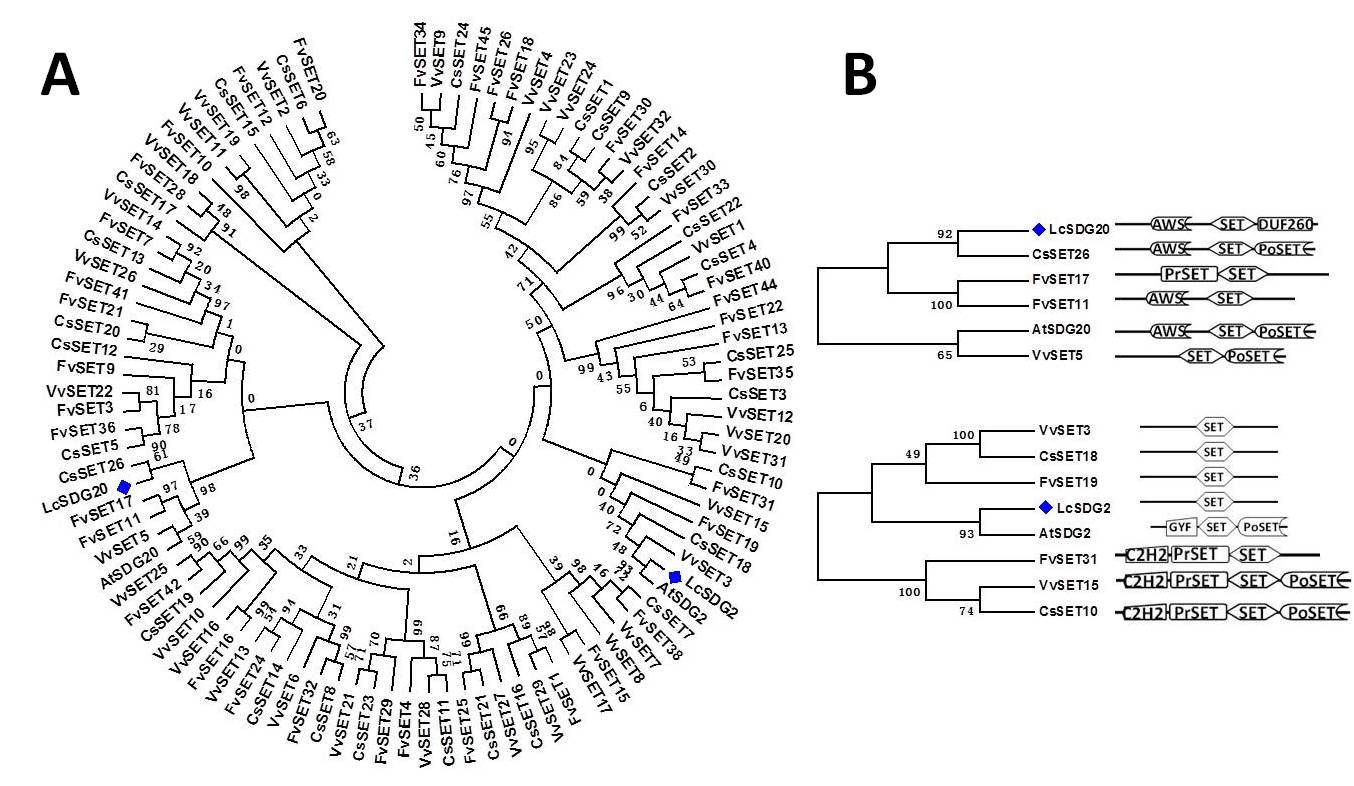

Supplement: Figure S2 — Maximum likelihood phylogenetic tree of SET domain-containing HMT proteins predicted from Litchi chinensis (Lc), Fragaria vesca (Fa), Citrus sinensis (Cs), and Vitis vinifera (Vv). The phylogenetic tree was constructed based on the amino acids sequences with 1,000 bootstrapping replicates. [file Image2.JPEG]

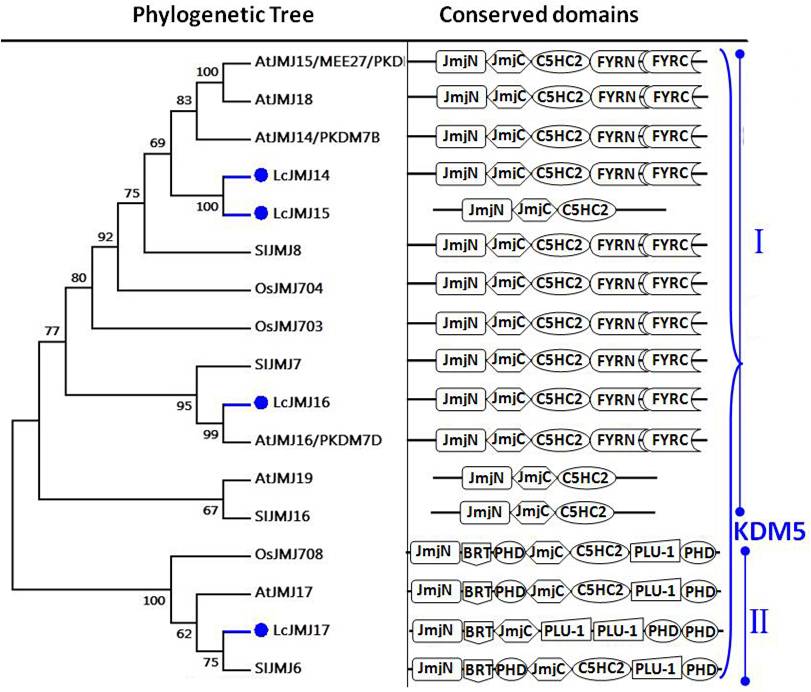

Supplement: Figure S3 — Maximum likelihood phylogenetic tree of KDM5 class HDM proteins predicted from Litchi chinensis (Lc), Arabidopsis thaliana (At), Oryza sativa (Os), and Solanum lycopersicon (Sl). JmjN, BRIGHT/ARID (PF01388), JmjC, C5HC2, FYRN (PF05964), and FYRC (PF05965) are conserved domains of the subgroup I of KDM5; JmjN, BRT (PF01388), PHD (PF00628), JmJC, and C5HC2 are conserved domains of the subgroup II of class KDM5. The phylogenetic tree was constructed based on the amino acids sequences with 1,000 bootstrapping replicates. [file Image3.JPEG]

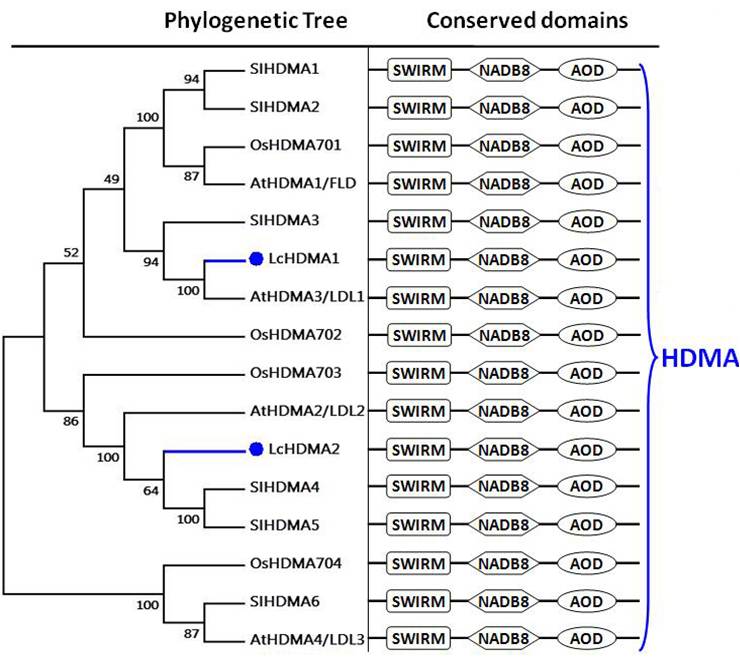

Supplement: Figure S4 — Maximum likelihood phylogenetic tree and schematic diagrams for domain composition of HDMA type HDM proteins predicted from Litchi chinensis (Lc), Arabidopsis thaliana (At), Oryza sativa (Os), and Solanum lycopersicon (Sl). N-terminal SWIRM (PF04433), NADB8, and C-terminal Amino_Oxidase domain (AOD) (PF01593) are conserved domains of HDMA type HDM proteins.The phylogenetic tree was constructed based on the amino acids sequences with 1,000 bootstrapping replicates. [file Image4.JPEG]

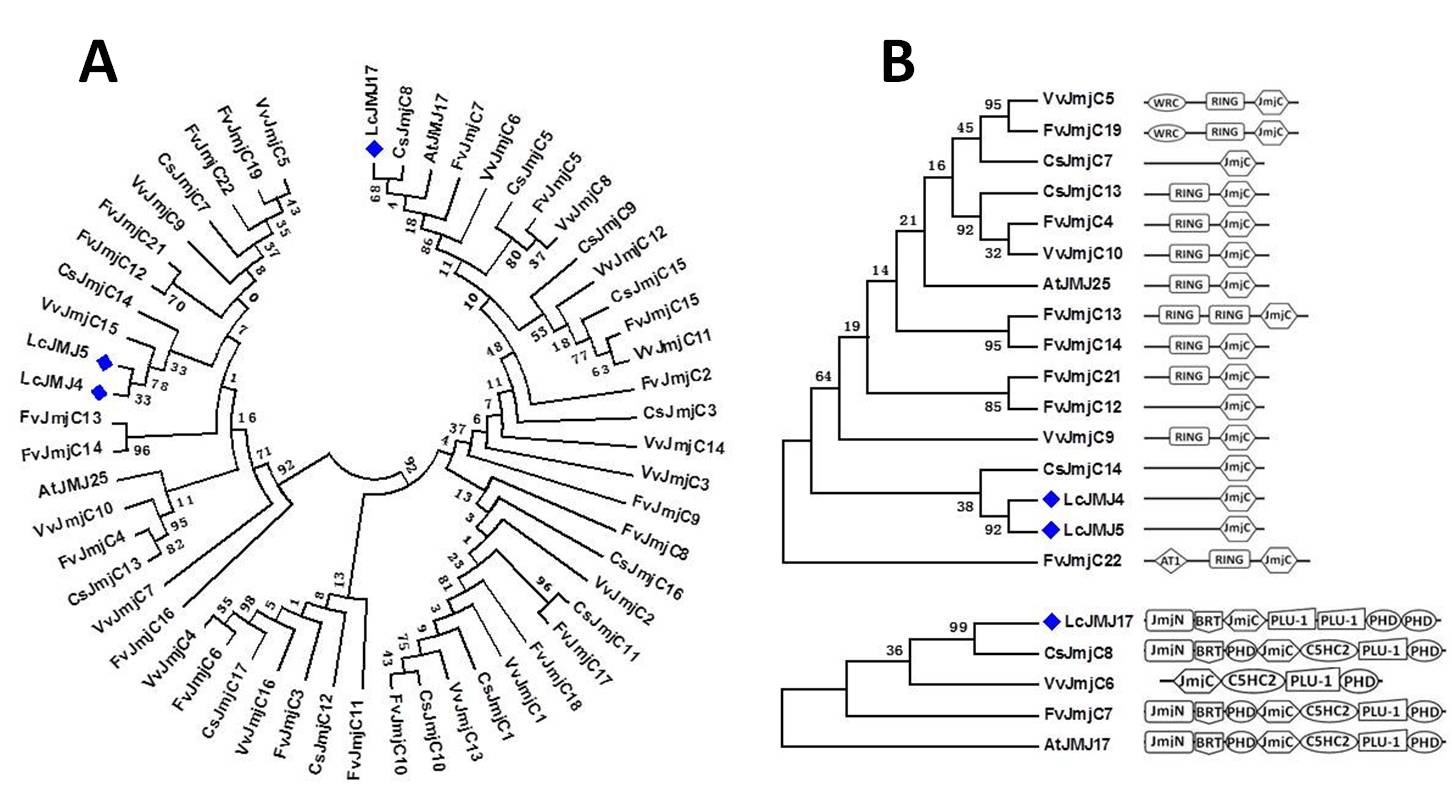

Supplement: Figure S5 — Maximum likelihood phylogenetic tree of JmjC domain-containing proteins predicted from Litchi chinensis (Lc), Fragaria vesca (Fa), Citrus sinensis (Cs), and Vitis vinifera (Vv). The phylogenetic tree was constructed based on the amino acids sequences with 1,000 bootstrapping replicates. [file Image5.JPEG]

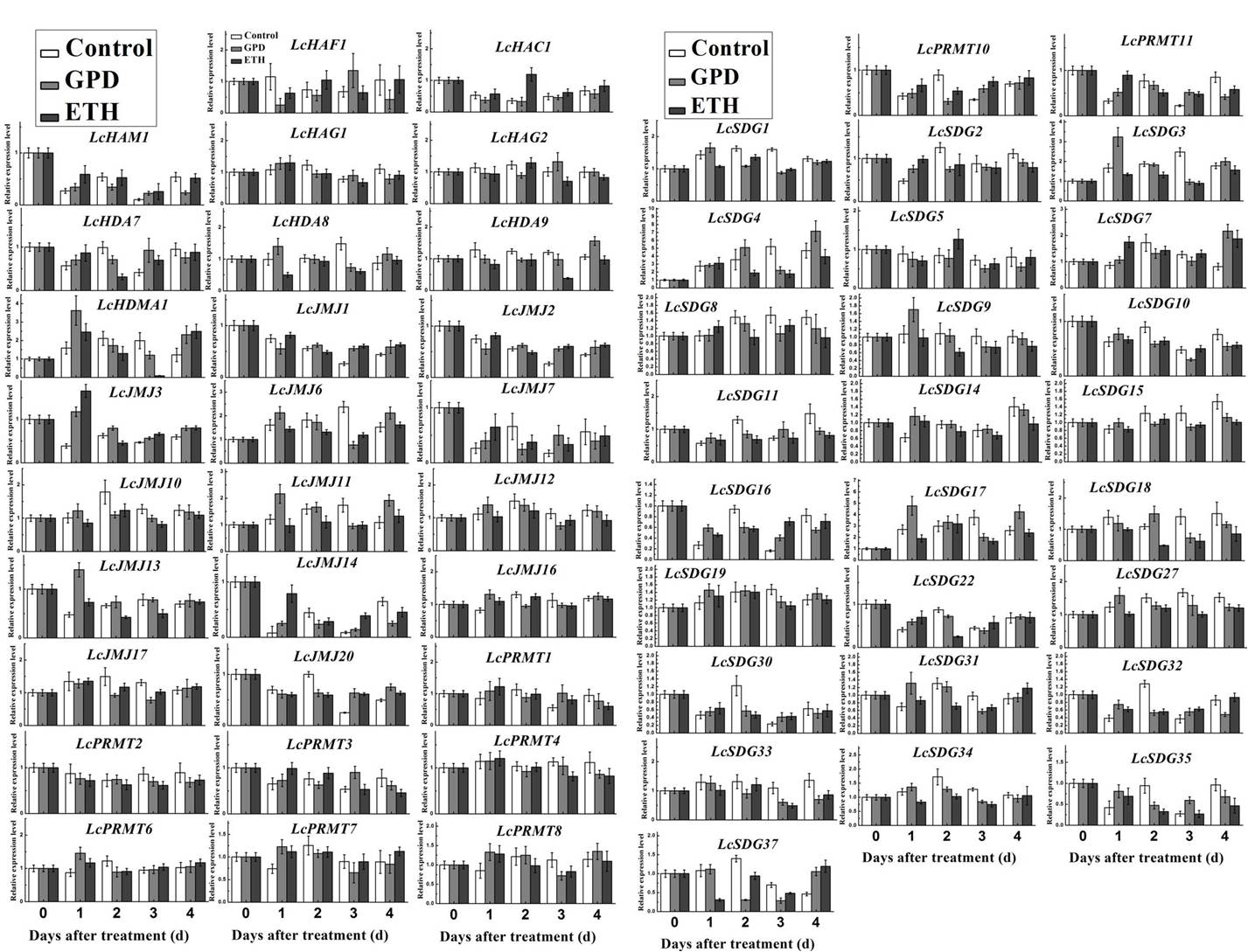

Supplement: Figure S6 — Expression level of HMs in AZ cells during fruit abscission in litchi. GPD indicated Girdling Plus Defoliation (GPD) treatment and ETH indicated ethephon (ETH) treatment. qRT-PCR analysis was used. LcEF-1a was used as an internal control. Data shown are means ± SD. One-way ANOVA (Tukey-Kramer test) analysis was performed, and statistically significant differences (P < 0.05) were indicated by asterisks. [file Image6.JPEG]
