# Supplementary material for: Genome-Wide Identification of Histone Modifiers and Their Expression Patterns during Fruit Abscission in Litchi
Source: Front Plant Sci. 2017 Apr 27;8:639. doi: 10.3389/fpls.2017.00639 (PMC5406457; doi:10.3389/fpls.2017.00639)
Supplement: File S1 — Protein sequences used in this study. [file Table2.DOCX]

>LcHAC1

MNVQAHMTGGQVPNPQQNGNALPPNQLHNLVGGVAPPAAPPPVLQHNMPNMDNDLSRARAVICERIYQILSRRQTQPADEATKMKFRDISKRLEEGLFKTAHTKEDYMNMDTLEPRLSSLIKGRSGNNHNQRHQQLVNSTSAVGTMIPTPGMTHSGNSPMMVASSMDSSMITTGGGNTIAPATVNTGSLLSTGGIQSGSFNRSDGSLPNGYQQSAANYSIGSGGNMSSMGAQRVTSQMIPTPGFNNNNTSSNSNQPYMNLESSNNSGGFSTVESTMVSQPQQQKQHVGGQNSRILHNLGSHVGIRSGLQHKYGFSNGALNGGLGMIGNNLQLANESGTSEGYLTATPYTHSPKPLQQQFDQHQRLMAGDGYAMGNADSFGTGNFYGGSMTNSQNLSSVNMQSMPMSKTTSLLIGNQSNLHGAPQAAHVKPPSIDQSEKVNFHPSLTSRDGLLQSHQPQQFQQQPHQFQQQQQYVQHQRQQQKQQSQQQQHLLNSDAFGRPQLMADMGGQVKSEPGIEQHNNAMHSQVSEQFQLPETQNQFQQSSSEDRSRGAQHLPVSSGQHDLCSSLTQISQPMQQMLHPHQMVADSHSTFSVGAQSESLLQGQWQPQSQERTHMTGNMPHEQHIQEDFRQRIAVQGEAQCNNLSSEGSIVGQPVGPRSTVEPPNPRGASCRSSNGNRDRQFRNQQRWLLFLRHARRCQAPEGKCQDVNCITVQKLWRHMDSCNSPSCAYPRCRHSKILIHHHKHCRDPCCPVCVFVKNYLQQQKERVRLNSDSHLLSPVSGSCKSYPTGDVSGGVISKTPSAVETSGDIQPSLKRMKIEQSAQSIAPESESSAVSASAISEPHISQDVQHQDYQHGKIIMPVKSEFMDVKMEVPLSSGQGSPPNNEMNDNMDDVSNQKADEPIVYDDPAALAKQENIKVEKENELAKQENVTQPAENAAGTKSGKPKIKGVSLTELFTPEQVKEHITGLRQWVGQSKAKAEKNQAMEHSMSENSCQLCAVEKLTFEPPPIYCTPCGARIKRNAMYYTMGAGDTRNYFCIPCYNEARGDIIVVDGSPTPKARLEKKKNDEETEEWWVQCDKCEAWQHQICALFNGRRNDGGQAEYTCPNCYIEEVDRGERKPLPQSAVLGAKDLPRTILSDHIEQRLFRRLKYERQERARNQGKSYDEVPGAESLVIRVVSSVDKKLEVKPRFLEIFQDENYPTEFPYKSKVVLLFQKIEGVEVCLFGMYVQEFGSECQFPNQRRVYLSYLDSVKYFRPEIKTVTGEALRTFVYHEILIGYLEYCKLRGFTSCYIWACPPLNGEDYILYCHPEIQKTPKSDKLREWYLSMLRKAARENIVVDLTNLYDHFFVTTGECRAKVTAARLPYFDGDYWPGAAEDLIYQIRQEEDGKKQNKKGLTKETITKRALKASGQTDLSGNASKDLLLMHKLGETICPMKEDFIMVHLQHACTHCCILMVSGTCHMCRQCKNFQLCDKCFEAEKKREDRERHPVNSREVHPLEEVPITDVPADTKDKDEILESEFFDTRQAFLSLCQGNHYQYDTLRRAKHSSMMILYHLHNPTAPAFVTTCNICHLDIETGQGWRCEVCPDYDVCNSCYQKDGGIDHPHKLTNHPSNADRDAQNKEARQLRVLQLRKMLDLLVHASQCRSPHCQYPNCRKVKGLFRHGIQCKTRASGGCVLCKKMWYLLQLHARACKESECHVPRCRDLKEHLRRLQQQSDSRRRAAVMEMMRQRAAEVAGNAG

>AtHAC1

MNVQAHMSGQVSNQGTMSQQNGNSQMQNLVGGGSAPATGAGLGPSRVSPVDNDILKLRQAMRIRIFNILQQKQPSPADEASKAKYMDVARRLEEGLFKIANTKEDYVNPSTLEPRLASLIKGRQLNNYNQRHANSSSVGTMIPTPGLQHSGGNPNLMITSSGDATMAGSNNITTSAMNTGNLLNSGGMLGGNLSNGYQHSSSNFGLGSGGNMSSMSSQRNTGQMMPTPGFVNSSTNNNSNNGQSYLSVEASNNSGGFSTAPMMVPQTQQQQLRQDIGGQNSRMLQNHGSQMGVGLRPGMQQKLSNVSNSSINGGVGMNAKSVDSGTSYTNPIRNSQQAYDNLQRSGMQGDGYGTNNSDPFGSGNLYGAVTSVGMMTNTQNANTASFQAVSRTSSSLSHQQQQFQQQPNRFQQQPNQFHQQQQQFLHQQQLKQQSQQQQRFISHDAFGQNNVASDMVTHVKHEPGMENPSESIHSQTPEQFQLSQFQNQYQNNAEDRHAGSQILPVTSQSDMCTSVPQNSQQIQQMLHPHSMASDSVNGFSNLSVGVKTESGMRGHWQSQSQEHTQMSNSMSNERHIQEDFRQRMSGTDEAQPNNMSGGSIIGQNRVSTTSESLNPQNPTATTCRNGNGNRDPRFKNQQKWLLFLRHARHCKAPEGKCPDRNCVTVQKLWKHMDSCAAPQCSYPRCLPTKTLINHHRSCKEPNCPVCIPVKAYLQQQANARSLARLKNETDAARSVNGGGISSDAVQTSAGAKSCTSPGADISGHLQPSLKRLKVEQSSQPVDVETESCKSSVVSVTEAQSSQYAERKDHKHSDVRAPSKYFEVKAEVSDFSVQTRPGFKDTKIGIAENIPKQRPVSQPDKQDLSDVSPMQETTKVEKEPESLKKENLAESTEHTSKSGKPEIKGVSLTELFTPEQVREHIRGLRQWVGQSKAKAEKNQAMEHSMSENSCQLCAVEKLTFEPPPIYCTPCGARIKRNAMYYTVGAGDTRHYFCIPCYNESRGDTILAEGTPMPKARLEKKKNDEETEEWWVQCDKCEAWQHQICALFNGRRNDGGQAEYTCPYCFIAEVEQSKRKPLPQSAVLGAKDLPRTILSDHIEQRLFKRLKQERTERARAQGKSYDEIPTAESLVIRVVSSVDKKLEVKPRFLEIFREDSYPTEFAYKSKVVLLFQKIEGVEVCLFGMYVQEFGSECAFPNQRRVYLSYLDSVKYFRPEVRSYNGEALRTFVYHEILIGYLEYCKLRGFTSCYIWACPPLKGEDYILYCHPEIQKTPKSDKLREWYLAMLRKASKEGIVAETINLYDHFFMQTGECRAKVTAARLPYFDGDYWPGAAEDLIYQMSQEEDGRKGNKKGMLKKTITKRALKASGQTDLSGNASKDLLLMHRLGETIHPMKEDFIMVHLQPSCTHCCILMVSGNRWVCSQCKHFQICDKCYEAEQRREDRERHPVNFKDKHALYPVEIMDIPADTRDKDEILESEFFDTRQAFLSLCQGNHYQYDTLRRAKHSSMMVLYHLHNPTAPAFVTTCNACHLDIETGQGWRCEVCPDYDVCNACFSRDGGVNHPHKLTNHPSLADQNAQNKEARQLRVLQLRKMLDLLVHASQCRSAHCQYPNCRKVKGLFRHGINCKVRASGGCVLCKKMWYLLQLHARACKESECHVPRCRDLKEHLRRLQQQSDSRRRAAVMEMMRQRAAEVAGGSGINMRALNQGCHFPEEEEREQCQPSSQMTRVLRDSGLMGFGWLIF

>AtHAC2

MAPPRKRTRDLMPKFLNTESFDEFNQRLNNLPAESNVTSDEDAQFLESRKCQSKRWRKEEPLKLNLRSPWNVLCSPESISSAKFIVEKTCLIPVPSFEEAATNARRCLNTSSIPGSSGSASETNSGSDITKQDFKNDSPSDSKKVQGSSTSKSAKPKVIKVYSFVDLVTTTKKGNIQTEESSLNHEKKLGTVVDIVEPMKCDERSKEVQGSSTSKSAKPKVIKVYSFADVVTTTKKGNIQTEESSLNHEKKLGTVVDIIEPMKCDERSKEVQGSSTSKSAKLKVIKVYSFADVVTTTKKGNIQTEESSLNHEKKLGTVVDIVEPMKCDERSKEVQGSSTSKSEKPKVIKVYSFADVVTTTKKGNIQTEESSLNHEKKLGTVVDIVEPMKCDEGTKCEVTTTNKGKIHTEERSLNHEKKLGTVVDIVEPMKCDEGSKCEVTTTNKGNTQTEERSLNHEKKLGIGVDIVEPMKCDEGTKCEVTTTNKGKIQTEERSLNYEKKLGIGVDIVEPMKCDEENKCEVNADTFDVVIVEPMKCNKVTKCEVNVDTTGVNIVEPMKCNEVTKCEVNVDTIGVDIVEPMKCNEESKCEVNADTMSLQKRSKRAVSLVERFTEEEIKLHIMSLKKPSTQSAVEGMCDLKEEEESCQLCDDGTLLFPPQPLYCLLCSRRIDDRSFYYTPGEEELSNAQHQICSPCHSRCKTKFPLCGVFIDKHKMLKRSNFDNADTEEWVQCESCEKWQHQICGLYNKLKDEDKTAEYICPTCLLEECQSINNMALVDYTDSGAKDLPETVLSYFLEQRLFKRLKEERYQTAKATGKSINDVPEPEGLTLRVVFSADRTLTVNKQFASLLHKENFPSEFPYRSKVILLFQKVHGVDICIFALFVQEFGSECSQPNQRSTYIFYLDSVKYFKPERVTFAGEALRTFVYHEVLIGYLEYCKLRGFTTSYIWACPPKIGQDYIMYSHPKTQQTPDTKKLRKWYVSMLQKAAEQRVVMNVTNLYDRFFDSTEEYMTAARLPYFEGSFWSNRAEIMIQDIEREGNNELQKKVKLLSRRKVKTMSYKTTGDVDVDDVKNILLMEKLEKEVFPNKKDLMVVELNYSCTRCSKAVLSGLRWFCEKCKNLHLCESCYDAGQELPGEHIYKRMDKEKHQLSKVQVNGVLFSTTEDNDIIQENDMFESRQAFLAFSQKHNYNFHTLRHAKHSSMMILHHLHTSNKHHCSQNSSSLTCTACKKDVSTTIYFPCLLCPDYRACTGCYTKNRTLRHLHIFPTLPSANRAPSRTVMVLEILNAISHALLCQHKTTKSCSYPKCHEVKALFTHNVQCKIRKKGTRCNTCYKLWQTIRIHVYHCQDLNCPVPQCRDRKEVLIRKV

>AtHAC4

MNNNKEVPQNSVAVSSSSSAPITVISPQQDANNFIKKRRTALRNRIYAIVRHKQKQHQIFLDKKNQQQQQRVDDATQRALLEKQDQQCIAATRMIEEELLKSSRSFEEYFDLRTFDARVRTILQQLGTMLSQRRAAAAAMNNGEAQCITSTRAVHTSISVSNSFQCGRSLVPINCTTATAGAFSIGPDMQTHHSTGANHQMVEVNRPNMNQITCGISSPLITGFNGNCVPVSANIPMTSQDLFNATHFSTLPQPFLQPPPDQSHMHRYSMSNVASFGQSNPYPCGVVMSSGSMAVAQNSIPWNNPNPMQGLDPTVTSYHSNLQPMQQTPLPKRQLHHPLWNTNFQSAPNNRDNLPQVSQQLSNHGSRQHRGQHSQNLYPGQLQNQDRLLPNLTQQAMALAAPVMHVPSKQVNEDCGQTSSNTVLRWIPFMFHARHCKAKKDKCASKFCFQARKIVKHIDCCKVPNCKYRYCLGTRMWLDHFKQCKSISCRTCVAVREYMEKNKYTIVPLRRAKCSSASSKCQPKKSSKSRQAYKKGGAEAPSVDADLQRSIKRPKLHRPSQNITPETKSISVTGCGVVCKPHSLMNMQEKDGLQSLKVEAMPMDIDVPGASEIPVTRELVKHVAEDTPKGNNCGGFAMVEKTSCLLAQGKSKCMNEMSAPKEENVKQSVEVVDASKMEISSLVELFTPEQVKEHIRSLRQWVGQSKTKAEKNKAMGCSMSVNSCQLCAVEWLVFEPVPIYCSPCGIRIKKNALHYSIAVGESRHYVCAPCYNEAREKLVFLDGTSIPKTRLQKKKNDEQVPEGWVQCDKCEAWQHIICALFNSRRNHGESTKYTCPSCYIQEVEQRERRPLPLSAVPGATSLPVTSLSKHLEERLFKKLKEERQERARLQGKTYEEVPGAESLTVRVVASVDKVLEVKERFLELFREENYPSEFPYKSKAIFLFQKIENVEVCLFGMFVQEFGTDSGPPNERRTFRTVSGEALRTFVYHEILIGYLDYCKKRGFTSCYIWACPPLKGDDYILYCHPEIQKTPKTDKLREWYLAMLRKASKEDVVVECTNLYNHFFVQSGECRANVTAARLPYFDGDYWPSAAEDLLRQMNQEDDGETKLHRKGLTKKVISKRALKAVGQLDLSLNASKDRLMMQKLGETICPMKEDFIMVHLQHCCKHCTTLMVSGNRWVCNHCKNFQICDKCYEVEQNRINIERHPINQKEKHALFPVAIKDVPTKIEDKDNNLESEFFHNRQAFLNLCQGNNYQYETLRRAKHSSMMILYHLHNPTAPAFATVCTICQQEVENSQGWHCEVCPGYDVCSACYSKDSINHSHKLTSRSSSTDSTVVQQNGQASQSYQVKLEKLKKLLVHAATCRSTQCQYQGCRKSKMLFRHCIDCTTGDCPICKGLWSLLKLHARNCRDSKCTVPKCSGLRAISRRKQQQADKRRRAAVMEMMRERAAEATRTG

>AtHAC5

MAQGQNRTVLQSGQMHNSVVASASSVSSQPNMVNGISQDTLLLRQEMLNRTYAWLQQRQPSKTDDASKAKLSEVAKRLESAMWRTATSKEDYLDFRSFDVRVESTLKQLLSQRRANPSSSVSTMVQTPGVSHGWGQSYTATPMVDTSKFNSSNNLSDATTETGRLLPTNRMYRGTINNGRQELSAVGQMIPTPGFDNSANADVYQSHRNEEYSGDGGKLLATGSDFGNPSQLQKQRPTGSNDLMGYNLDHQLGGGFRSNIHQNTSGMTSIPLNAGVGMSGNNVHLANVPRSSEGVLSSTHFSTFSQPSQQPVEQLQVSHVNRYSMSNSGTFVSGNLYGVQTSSGSIETAVDMNSMSLNSMRRVDTSFGSQSGLQNNPLLKPHLRHQFENGNFQSSSNSKENLAQVSHRPLERQFNQQAHYGQYHQQELLMNNDAYRQSQPASNLVSQVKNEPRVEYYNEAFQMQAINKVEPSKPQNQYKQNTVKDEYVGAQSAPVSSSQLKMSPSFPPQTHQTQQVSQWKDSSSLSAGVQPVSGLGQWHSSSQNLTPISKNSNEEREHFGVRFHKQHEGTNNSSSVRESTNCLTVAPSGTLDVPHLPVGINVLSKQLNGDCGLSYKNQRRWLLFLLHVRKCNAAEDNCESKYCFTAKTLLKHINCCKAPACAYQYCHQTRQLIHHYKHCGDEACPVCVFVKNFKEKQKEKFTFLQRAEPSSASLNHGPKESFESMRTSSERDSEAPFVVDDLQPSPKRQKVEKPSQFAYPDTQGNPATISAGVSQAHFSMGLQEKDRLPSDVCKPVRSNVPMNADSSDSSRRLVPVSRELEKPVCKDTHMGRHGVKSALDGESLRLSKQEKPKRMNEISAPKEENAEQSLGVVSASNCGKSKIKGVSLIELFTPEQVEEHIRGLRQWVGQSKTKAEKNKAMGLSMSENSCQLCAVERLAFEPTPIYCTPCGARVKRNAMHYTVVAGESRHYVCIPCYNEARANTVSVDGTPVPKSRFEKKKNDEEVEESWVQCDKCQAWQHQICALFNGRRNHGQAEYTCPNCYIQEVEQGERKPVSQNVILGAKSLPASTLSNHLEQRLFKKLKQERQERARLQGKSYEEVPGADSLVIRVVASVDKILEVKPRFLDIFREDNYSSEFPYKSKAILLFQKIEGVEVCLFGMYVQEFGTDSASPNQRRVYLSYLDSVKYFRPDVRTVSGEALRTFVYHEILIGYLDYCKKRGFSSCYIWACPPLKGEDYILYCHPEIQKTPKTDKLREWYLAMLKKASKEKVVVECTNFYDHFFVQSGECRAKVTAARLPYFDGDYWPGAAEDLIDQMSQEEDGKKSNRKLMPKKVISKRALKAVGQLDLSVNASKDLLLMHKLGEIILPMKEDFIMVHLQHCCKHCCTLMVSGNRWVCNQCKNFQICDKCHEVEENRVEKEKHPVNQKEKHVLYPVAIDNIPTEIKDNDDILESEFFDTRQAFLSLCQGNHYQYDTLRRAKHSSMMILYHLHNPTVPAFAMACAICQQELETAQGWRCEVCPDYDVCNACYSKGINHPHSIISRPSATDSVVQNTQTNQIQNAQLREVLLHVMTCCTAQCQYPRCRVIKGLIRHGLVCKTRGCIACKKMWSLFRLHSRNCRDPQCKVPKCRELRAHFSRKQQQADSRRRAAVMEMVRQRAADTTASTPE

>AtHAC12

MNVQAHMSGQRSGQVPNQGTVPQNNGNSQMQNLVGSNGAATAVTGAGAATGSGTGVRPSRNIVGAMDHDIMKLRQYMQTLVFNMLQQRQPSPADAASKAKYMDVARRLEEGLFKMAVTKEDYMNRSTLESRITSLIKGRQINNYNQRHANSSSVGTMIPTPGLSQTAGNPNLMVTSSVDATIVGNTNITSTALNTGNPLIAGGMHGGNMSNGYQHSSRNFSLGSGGSMTSMGAQRSTAQMIPTPGFVNSVTNNNSGGFSAEPTIVPQSQQQQQRQHTGGQNSHMLSNHMAAGVRPDMQSKPSGAANSSVNGDVGANEKIVDSGSSYTNASKKLQQGNFSLLSFCPDDLISGQHIESTFHISGEGYSTTNPDPFDGAITSAGTGTKAHNINTASFQPVSRVNSSLSHQQQFQQPPNRFQQQPNQIQQQQQQFLNQRKLKQQTPQQHRLISNDGLGKTQVDSDMVTKVKCEPGMENKSQAPQSQASERFQLSQLQNQYQNSGEDCQADAQLLPVESQSDICTSLPQNSQQIQQMMHPQNIGSDSSNSFSNLAVGVKSESSPQGQWPSKSQENTLMSNAISSGKHIQEDFRQRITGMDEAQPNNLTEGSVIGQNHTSTISESHNLQNSIGTTCRYGNVSHDPKFKNQQRWLLFLRHARSCKPPGGRCQDQNCVTVQKLWSHMDNCADPQCLYPRCRHTKALIGHYKNCKDPRCPVCVPVKTYQQQANVRALARLKNESSAVGSVNRSVVSNDSLSANAGAVSGTPRCADTLDNLQPSLKRLKVEQSFQPVVPKTESCKSSIVSTTEADLSQDAERKDHRPLKSETMEVKVEIPDNSVQAGFGIKETKSEPFENVPKPKPVSEPGKHGLSGDSPKQENIKMKKEPGWPKKEPGCPKKEELVESPELTSKSRKPKIKGVSLTELFTPEQVREHIRGLRQWVGQSKAKAEKNQAMENSMSENSCQLCAVEKLTFEPPPIYCTPCGARIKRNAMYYTVGGGETRHYFCIPCYNESRGDTILAEGTSMPKAKLEKKKNDEEIEESWVQCDKCQAWQHQICALFNGRRNDGGQAEYTCPYCYVIDVEQNERKPLLQSAVLGAKDLPRTILSDHIEQRLFKRLKQERTERARVQGTSYDEIPTVESLVVRVVSSVDKKLEVKSRFLEIFREDNFPTEFPYKSKVVLLFQKIEGVEVCLFGMYVQEFGSECSNPNQRRVYLSYLDSVKYFRPDIKSANGEALRTFVYHEILIGYLEYCKLRGFTSCYIWACPPLKGEDYILYCHPEIQKTPKSDKLREWYLAMLRKAAKEGIVAETTNLYDHFFLQTGECRAKVTAARLPYFDGDYWPGAAEDIISQMSQEDDGRKGNKKGILKKPITKRALKASGQSDFSGNASKDLLLMHKLGETIHPMKEDFIMVHLQHSCTHCCTLMVTGNRWVCSQCKDFQLCDGCYEAEQKREDRERHPVNQKDKHNIFPVEIADIPTDTKDRDEILESEFFDTRQAFLSLCQGNHYQYDTLRRAKHSSMMVLYHLHNPTAPAFVTTCNVCHLDIESGLGWRCEVCPDYDVCNACYKKEGCINHPHKLTTHPSLADQNAQNKEARQLRVLQLRKMLDLLVHASQCRSPVCLYPNCRKVKGLFRHGLRCKVRASGGCVLCKKMWYLLQLHARACKESECDVPRCGDLKEHLRRLQQQSDSRRRAAVMEMMRQRAAEVAGTSG

>OsHAC701

MMAKTLQGTQQQYAASGFPTQQYPTSGWTQSAAEILQLDNMDQDTSVVRNIIHRKIVEYLNERKEFCNFDLSFLMEIGKCIDRHLFEKADSKIKYMDLETLRTRLNAIVNSASFRGSMFHWSASAASSKLNSQQLPVMEVPIYHDRVTPGPNNLPSCAYNVSSTQGYNQYENCMGAANFAHSLADKPKQMPERLANTIFTSCASTLPKCSPSIDVLHIGHIKEHFSGDAYQNDSSQPSTSGSSSSLSAVWDQTTCSSAMRTLPMDSFSTVNGQNLSTNNKSLYPTTGQGPLLQQYIECEMKQETWSRSLEQSDQSNITTGNRDLYHAQIHPYINGEHKRDRCIQMKEKLGHTSDHEGFSREKSSNLSNHFMHHQQGFMTNYGACSPVSKTVDRAEQTSNSTVSKPTSPASDGSSGKHYPAKRLKVDVPHLVHVNEMEASKEQQPAANETYASAETVQSEVTNSPTKSPCCTSLGDNIACTDNVHGMDMVRLSGSAVQTEEEFRRENSDIEMKDAKVDLLDQTLSGDSLRARKRRGASVLYALTSEELKDHLCTLNHDTSQSKVPTEELLSVEGLPDQNTCNLCGMERLLFEPPPRFCALCFKIINSTGSYYVEVENGNDKSSICGRCHHLSSAKAKYQKRFSYAETDAEAEWWVQCDKCKAWQHQICALFNPKIVDPEAEYTCAKCFLKEKDNEDVDSLEPSTILGARELPRTRLSDHIEQRLSERLVQERQQRAIASGKSVDEVPGVEGLTVRVVSSADRTLQVQPRFKDFFKKEQYPGEFPYKSKAILLFQKNEGVDVCLFAMYVQEYGSACPSPNQRHVYLAYIDSVKYFRPEIKSASGEALRTFVYHEILIGYLDFCKKRGFVSCSIWTCPSTKRDDYVLYCHPTIQKMPKSDKLRSWYQNLVKKAVKEGVVVERNTLYDFFLQPTNECKTNISAAWLPYCDNDFWPGEAERLLEKKDDDTSQKKETQLGRLLRVAKRDDRKGNLEDILLVHKLGERLRTMKEDFLMLCLQQFCKHCHHPIVSGSSWVCTSCKNFFLCERCYAEELNTPLKDRHPATTKQKHAFERIEEEPLPETDDVDPTMESKYFDSRIDFLKHCQDNQYQFDTLRRAKHSTMMILYHLHDSTCSSCHRAMDQCLAWRCLVCLGCNFCDSCYKQDGESLHIHKLRQKKDHHVLQKYTLQDYLEGLVHASRCFDRSCTSKLCLTLKKLFFHGVRCHTRARGGGGCHMCVFMWKLLFTHSLLCDNADCSAPRCRDIKAYIADRSMTDLSIS

>OsHAC703

MNVGQAAHLSGQMSGQAPQTNQVGGSGVGGADGLPQQMQDVVGLGGLDTQFLLMRNTMRDRIFEYIGRKQSSTDWRRRLPELAKRLEEILYRKFLNKADYLNMMRGPVEPQLQFAIKTLSAQNQQNQQNQQMPRQMASSSGYGTMIPTPGITQSATGNSRMPYVTDNTGLPSSGATMVPQGANTGSMSNGYQHLTTSVPLNSTTSSIPSTMGPVGIQRQVTHMIPTPGFNNQQNVPVNPDFSNGAGYFNGEPTVTSQMQQQKQFPSNQNSHQIQHIGGHSNSGMHSNMLENSSAYGLSDGHVNGGMGVHGSNMQLTNRSAASEAYINISTYGNSPKPVQQQFNQHPPQRIPTPVDISGSGNFYNTGSSALTAANNHSMGATNLPSRSRMNSMLHTNQLNMQSIQPQPQIKTEVLDQPEKMNFQSSQLTHEQLIRQQHSMQQHQMQPSSQFVQNQYHLNQQQPNSQHQQSILRSNSLKQPQLSSSHSMQLSEQGALPHTELISSQATEHADIPIYQGQYQQRSAHDNVKGGQVFGHLSSSQNFHSNASHDSQQLLPTNQQLDDSSNDVSYVLKGSQPEQMHQAQWRPQTMEKAPVTNDSSLEKQIQADLCQRTMSQDGAQQPFSSDWRLPGCTVTPADPALPKLPSGGLEQAAGNIYYFRQMKWLLLLFHAKSCLTPVGSCKFHRCFQVQELVKHFENCKRKDCSYRDCRRSRMVTEHYKACVDLQCPVCSNAKKLLQRSAELASKQKPPEPRKIAQQNTAQRIMNGVEGDIMDIDLVSDEIFDSQPSVPKRLKMQPVSPSTAEREVSMPSNAGLILQETHSELPDQNNKVGQLKMDVKIDPRPLQKPAKIGYGTDGNVPTARHNVAPGGSNEIKTHVKQEIMPIDKETSETAPEVKNEANDSTDITVSKSGKPKIKGVSMTELFTPEQIQEHINSLRLWVGQSKAKAEKNQLMGHNENENSCQLCKVEKLTFEPPPIYCSPCGARIKRNAPYYTVGTGDTRHFFCIPCYNESRGDTIEVEGQNFLKARFEKKRNDEETEEWWVQCDKCECWQHQICALFNGRRNDGGQAEYTCPNCYVEEVKRGLRMPLPQSAVLGAKDLPRTVLSDHIEDRLFKRLKQERQDRAAQERKSIEEVPGAEGLVVRVVSSVDKKLEVKPRFLEIFQEDNYPTEFPYKSKAVLLFQKIEGVEVCLFGMYVQEFGAECSYPNQRRVYLSYLDSVKYFRPEIRTVSGEALRTFVYHEILIGYLEYCKQRGFTSCYIWACPPLKGEDYILYCHPEIQKTPKSDKLREWYLSMLRKATKEEIVVELTNLYDHFFITMGECKAKVTASRLPYFDGDYWPGAAEDMINQLRQEEDDRKQQKKGKTKKIITKRALKAAGHTDLSGNASKDAMLMHKLGETIYPMKEDFIMVHLQYSCSHCCTLMVSGKRWVCHQCRSFYICDKCYDAEQQLEDRERHPSNSRDTHTLHPVDIVGLPKDTKDRDDILESEFFDTRQAFLSLCQGNHYQYDTLRRAKHSSMMVLYHLHNPTAPAFVTTCNVCCHDIETGQGWRCEVCPDFDLRKMLDLLVHASTCRSGSCQYPNCRKVKGLFRHGMQCKTRASGGCVLCKKMWYMLQLHARACRDSGCNVPRCRDLKEHLRRLQQQSDSRRRAAVNEMMRQRAAEVAANE

>OsHAC704

MKQGQGAHLSGQRIGHHPTAQMNPGDGDGNGRHQVASGHASADPELMNLRIRMTNRLIWELLSREPKLQTRPRKLVSDLAKRFEAVIYKKNPNKAAYYSILNGEIFPHLQHALSTHMAQHQQGQQMLQQLTSSSSYGTTIPIPDVVQNASGNTRALYEMDNTSGPMSNGHHHFSANFPLHSTTKGASLEMSAVSMQEGKITHMIPTPGSSNQQSLPGNFHYSTGTGYLNGKSNVMAQMQEQQAPFASKINCCPVQRDLGGYAGSGVHSDILNNSSPYGVSEAHMIDGMGLHRSNVQVINRTVVPETFINPSPYGISPNKPLQRHVNPSTRSTPTPADIAASTSFNGTGSSALSTTSYLDMTTVNSLPKSRMDSGLIMSQPTIQSFQTEYYIQTEGLDLQEKISLEQLHQQVNQLHLIQPHSQYAQNQCSLKLQQQNSLHHLVMSRGNVLTQCHLGSDHAEKLLDKRNQLHSELVSSQINEHVGLTNLQGHYEQTQYHDNYKKGQMSASSQNLGIPAPHDLLPPQQQFDDGSYRLSCFLKETYTKPLQPHCKSKPMKEVIMTSLLSGKIQDGFCQKKMARDREHHPIISGWHSAGCAATSFGSEEVMENTKQYHAQARWLLFLFHAKSCTSPPGSCKSSYCDRVRELVVHLTDCQIKDCSYRHCRESKMVSDHYKNCINEHCHVCCKAKEMLRRSSELAHKQNPAEPILITQHNMNQRSADRVHGDRMDIDQAVETFDDQPPAAKRPKLQLVSPDASENVPVCQKNPGFMLQEAHPRQLDQNKKMVPDQEVDVGLDIRHPQVTLVSCHGSDEKIGAAQNTVIPGALNKIHCHVQQETVVADKESVTVVDVKKKTGSVDVTISKTGKPKVKGVSLMELFTPEQIHEHINSLRQWIGQWVQCDKCECWQHQICALFNARRNDVEEAEYTCFKCYIEEFKRGLRMPLPESVVRGAKDLPRTLLSDHIEERLFKRLREERQERANKLKTSLDEVPGADGLVVRVVSSVDKKLEVKPHFFKILQEDNYPAEFPYKSKAILLFQKIEGVEVCLFGMYVQEYGAECKFPNQRRVYLSYLDSVKYFRPDIETVSGQALRTYVYHEILIGYLEYYKQRGFTSCYIWACPPVKGEDYILYCHPEIQKTPKSDKLRQWYLSMLQKAIKENIVVELTNLYDQFFVTAKECKIKVSAARLPYFDGDYWPGAAEDIINQLQLEGDGKLLKKGRVNKIITKRALKAAGHTDLSGNASKEAMLMQKLGEIICPIKDDLIMVHLQYSCSHCCTFMVSGRRWVCNECKSFYICDRCYNAEQRLEEKERHPSNSKCLHILHPVEIVGVSEDTKDRDIILENEIFDTRQAFLSFCQGYHYQYDTLRRAKHSTMMMLYHLHNPTGPAFVATCNVCNCDIENGQGWDFKSFERKQNQLSESRRMASVNERVRQRVAEVTRHE

>SlHAC1

MNLQHMSGQISGQVPNQSGTSLPGLPQQSGNPLTMQMQNPVVHSNVLNMEPDFSRARIFISNKIYDYLMQRQQSHEKPPKKVMDIVKRLEEGLFKSASSKEEYLNQATLENRLHVLIKSLRMNNQNQRFPRVNSSGSIGTMIPTPGMTQSANSALIGTSSVDSSMAAGSTIASSAGSFLPMANVSSSGCLTNGYQQPTSNFLVSSGGNNLVPSMSGQRMTSQMIPTPGFNASGGANLNSNTNTQSSLNLDSTNSIAALPSVDSMNVSQPLQQKQHVAAQNSRILHTVGSHVGGGIRSGFQNRSYGQSTGPLNGGGLGMIGNNLHLVNGSAPEGYISATTYGNSPKSLPQHFDQQHQPLMQGDRYGISHADTSGSGNLCLPVSSVGMVMNNQKPGAVALQSISRTNSPLITNQSNLTASGQMPNVKVQPADQSTKMNYQSQHSLGDNHLSSYQHQHSQQPPQQFQEQHQLVQPQLQQKLQNQQHQTLSRSNAFAQAQAPSDIGIQVKSEHGNHDEAQHSRVNAEQFQFSDMDQFQPNSIEDHSKDAVFQGQWYSKSQDGSQIPGSFSDKQNVQEELYLRTSRKEEAYPNNLCTERSPIGQPVGNGAVATNNASSSICRFNHLPRERQYFNQQKWLLFLTHARGCSAPEGKCAEKNCIKAQKLVKHMERCSTFECQYPRCPATRDLINHYRRCRDLNCPVCIPVRKFVRAQQKVARPGCNSDMPSSANGTCRSYGTGEIASRLTAKQGSVPVQTEDLQYSVKRPKIEQPSQSLIVETENCFMSVTASESHVTQNAQPIEQHGNAVAMKSEITDAMMEIPAKAVLVSPRSIDIRNDNLDGSCIRKSDGDSVVSSNAACLVKQENVKTEKDIVQPKQENMSAPSESTSGSKSGKPTIKGVSMTELFTPEQVREHIIGLRRWVGQTKAKAEKNQAMEHSMSENSCQLCAVEKLNFEPPPIYCTPCGARIKRNAMYYTIGTGDTRHYFCIPCYNEARGDTINVDGTTIPKARMEKKKNDEETEEWWVQCDKCEAWQHQICALFNGRRNDGGQAEYTCPNCYIIEVERGERKPLPQSAVLGAKDLPRTCLSDHIEVRLDRVLKDDRQKRAEREGKSYDEVPGAEGLVVRVVSSVDKKLEVKSRFLEVFQEENYPLEFPYKSKVLLLFQKIEGVEVCLFGMYVQEFGSECAQPNHRRVYLSYLDSVKYFRPEIKAASGEALRTYVYHEILIGYLEYCKKRGFSSCYIWACPPLKGEDYILYCHPEIQKTPKSDKLREWYLSMLRKAVKEKIVVDLTNLFDHFFTTTGECKAKITAARLPYFDGDYWPGAAEDMIFQLQQEEDGRKHHKKGAMKKTISKRALKASGQSDLSGNATKDILLMHKLGETISPMKEDFIMVHLQHACTHCCILMVSGNRWVCKQCKNFQLCDKCYEVEQKLEARERHPLYHKDIHMLYPTEIDDVPADTKDPDEILESEFFDTRQAFLSLCQGNHYQYDTLRRAKHSSMMVLYHLHNPTAPAFVTTCNICHLDIETGQGWRCETCPDYDVCNACYQKDGGVDHPHKLTHHPSIAERDAQNKEARQQRVLQLRKMLDLLVHASQCRSSHCQYPNCRKVKGLFRHGIQCKVRASGGCVLCKKMWYLLQLHARACKVSECHVPRCRDLKEHLRRLQQQADSRRRAAVMEMMRQRAAEVANSAG

>SlHAC2

MNFQQMSGQISAQVPNQSGTSLPGLPQQNGNPSPMQMQSPSVHRTIPNMEFELVKVRRTISRRIYEYLIRRHQQQQQIQEAQHQRIVDLVKRLEESLFKSASTKEEYMDLSTLENRLLSVIKRLPRNNHSQQFSHINSSASIGTMIPTPGMPRSLNASLVGTSSVDSSVTAGSTLTSSAVNSGNFVRTTNFPSGSMHGPLANGYQQSTSNFSINSGGNNLVPSMGGQRITSQMIPTPGFSNSDKSNNNTSAQSHINLESSNGGAAFSGVDSVTVSQTPQQKQNVSGQNSCILHTLGSHMGGGIRSELQNRSYGQSTAPLNEGLGMTGNSLQHLNGPGTSEGYTSATMHVDSPKSLPQHFDEHQRPEMQGTVISSQSLSAVALQSMSKTNSPLMSNTSNLAASQQMPNAKVQPVVQLEKMNLQSQYYLGDAHLSSHQPQQFQHQHKFAQHLSQQKLQSQQQQLVLSSSAVGGQLPSNPDTQVKLEPENHDEALQNQFQQKTVGEQSKGAVLQGERYPKSQDGSQIPGSFFEPNAQEELRQRTSTQEEAQPNNLSTGGSLASQSVANRIVETNNSSSAMRRSGNVPRERQYVNQQRWLLFLIHARRCSAPEGKCPETNCIHAQKLLRHMERCSKFDCRYLRCPETKVLINHYRQCKNVNCPVCIPVKKFMQTQHKVFGRPGYISDMTNSLNGICRTYDAVETASKLAGNLSPMAVKTPEDLQPSLKRMKIEPSSQPHILEIENFVPVSACESNVLQDTQFVEQNDAVVMKSEVTEVKMEAFANAVQVGPGSTDIAKNNLDDKYTQRPASDSLASSTPGCLVKEENVNTEKDIDLPKQENTSVPSESTSGSKSGKPKIKGVSMMELFTPEQVREHIKGLRQWIGQSKAKAEKNQAMEHSMSENSCQLCAVEKLNFEPPPIYCTPCGARIKRNAMYYTIGAGDTRHYFCIPCYNEARGDTIVVDGTSVPKARMEKKRNDEETEEWWVQCDKCEAWQHQICALFNGRRNDGGQAEYTCPNCYIAEVERGERKPLPQSAVLVAKDLPQTTLSDHIEKRLANSLREEREKRAKREGKGYDEVPGAEGLVVRIVSSVDKKLEVKPRFLEIFQEENYPLEFPYKSKVLLLFQRIEGVEVCLFGMYVQEFGSECAQPNHRRVYLSYLDSVKYFRPEIKTVTGEALRTFVYHEILIGYLEYCKKRGFTSCYIWACPPLKGEDYILYCHPEIQKTPKSDKLREWYLSMLRKAKEQNIVVELTNLYNHFFTSTGECKAKVTAARLPYFDGDYWPGAAEDMIYQLQQEEDGRKQPKKGTTRKTITKRALKASGQFDLSGNTSKDLLLMQKLGETISPMKEDFIMVHLQHACTHCCGLMVSGNRWECKQCENFQLCDKCYEIEQKLEDRERHPINQKDKHTLYQCKIKEVPHDTKDEDEILESEFFDTRQAFLSLCQGNHYQYDTLRRAKHSSMMVLYHLHNPTAPAFVTTCNICYLDIEAGQGWRCEVCADYDVCNACYQKDGGIDHPHKLTKHPSLADRDAQNKEARQLRVSQLKKMLELLVHASQCRFPHCPYVNCRKVKGLFRHGIQCKIRVSGGCVLCKKMWYLLQLHARACKVSECHVPRCRDLKEHLRRMQQQADSRRRAAVMEMMRQRTAEVAGGSG

>SlHAC3

MIGNTIKSFMCKRNYGIRSYINEADLPPSKRKRFENLPVRSKKNKTEMNNDLTEPKLLGVSLMDYFSIEQLKNHIRNLRSTETMSERSVTNNVCQLCSMDSLDFVPTPIHCSSCYKCIKRNLIYYWAVDESDRRHCYCNNCFRKCSDDDNVLGKNKFQKAKNNYRNEEPWVQCDKCECWQHQVCGLYNANEDLEGQAKYICPFCRLKEIEAGEHKPLPVSIGAQHLPRTMLSDHIEQRLFRRLELDRNERAKYDDEVPAAADLTVRVVLSVNRNLKLNQQFLDIFQNDEYPPEFQYKSKLILLFQKIGGVDVCLFGMYVQEFGSECASPNRRCVYISYLDSINYFTPDVRTVKGETLRTFVYHEILIGYMDYCKKRGFTACYLWACPSLKGEDYIFYCHPKSQKTPKPEKLRLWYKSMLRKASEEGIVVNHTNLYDQFFGPSTRNSAAHLPYFNGDYWSGAAEEIIRNIEKENRADKAKKLMTKSSLKAIGNDNVYADATKDILVMQKLGQTILPVKKEFIVVNLRFVCINCQQVIVSERRWSCKQCKSFHICNKCVALQKTHRSSSGDEHLLSEIVMEEDDIPVSTEDQDAIIENDIFENRHSVLSFCEKNHYQFDSLRRAKHSSMMILYHLYKNIHLSFEKEHFEGHGALKVKLMGALVHASQCCATLSNHCSFSGCIKVRQLFQHVSRCSHRVAGGCALCRKIWSLLHWHSQTCRDTSCIVPRCKDIKKHGVAYS

>SlHAC4

MGFQFSLQEECLESQMNLNRVNVGVPFCSGGTYLFQEASDLLQLNRTGSQQREKQDDNQIESTNDQTRVPVDGMIGNRIKSFMWKRNLDMGKQVFNNLADLSSQSSADAVCKQDHVQGTPSREYQNLFQQEACYPQAWQDEALCCSSNLQSVHHDHLTASCMTDSLLQTELSANKPGELGEVLSSVGVSYVSKEIADSPFSSVTVCSEGEWNANKEPYEDGLTYSDNGFLSISESVSKSPIKSQYSDEVEFNLRNCGRYLETNQNIHHDNVPALHGLSVDSSHLNRRPSESEVNWFSKDYTLPTAVSYQLPNSRSYGYLVKQDATMNNVSRQRSTLSSVLQPFPEQLPSIQQCDFHQLHTGSSNGSDCDVHPIGSANLSNSDNLSLHDILALYINYDSVVVDAGRIRIPFLNYLHLTVCNVKRCWCDWSSALISHFKNCQYAGCGMCKPVRELHPKDVKESVKNMVAILHDEECSGFRSSINEAVLPPSKRKRMENLPVLECWSSNADSGNLQSPAAGHLSLRQFVESPICSKKNKTEISNEIASCVEDQNTAGESCNIANIDILHVANGSFSSTELTNDCGLQKTVHTCTSGTDYNEIDSSSHMSLDRSSFLPIEPTDDQQQELQSASKYDQTTSSARINLTEPKADYQMEMRSEDPKRLGISLTDYFTIEQLKDHIHNLSQYNQGSTGNMTVLPISENVCQLCGTDRLVFVPTPVYCSSCCKCIKRNLVYYWAVDEAGGRHCFCTKCFRKSCGDDVSSQGLSINKNKFQKAKNNDQNEESWVQCDKCEGWQHQVCALYNAKKDFEGQAKYICPFCCLKEIEAGEHVPLPVSIGAQDLPRTMLSDHIEQRLFRRLKLERNERAKLSGQDADEVPGAADLIVRVVLSVNRNLKVKQQFLDLCHNEGYPPEFQYKSKVILLFQKIGGVDICLFGMYVQEFGSECAPPNRRCVYISYLDSVKYFKPDIETVKGEALRTFVYHEILIGYMDYCRKRGFTTCYLWACPPIKGEDYILYCHPESQKTPKPEKLRSWYWSMLRKASEEDIVVNYTNLYDHFFVPSTRNSARISAAHLPYFDGDYWSGAAEDIVRNIEKESRGDSQNKVKKLMTKSTLKAIGHDNLSADATKDILVMQKLGQTILPVKEDFIIVNLHVVCANCQQAILSEGQWSCKQCRNFHICRRCLALKDNLSEHKTHTSSSGEEHLLSEVVVNDIPASTEDQDAIIENDFFENRHSFLSFCEKNHYQFDSLRRAKHSSMMILYHLNKNIHLSKTDSGFGKVQFEGQRPLKVKLMDILVHASQCRATSSNPCSYSGCLKMRKLFQHASRCSVRVPGGCALCRKIWSLLHWHSQTCQDISCLVPRCKDIKKHVARRNPLLQRGERG

>AtHAF1

MAESNGKGSHNETSSDDDDEYEDNSRGFNLGFIFGNVDNSGDLDADYLDEDAKEHLSALADKLGSSLPDINLLAKSERTASDPAEQDYDRKAEDAVDYEDIDEEYDGPEVQVVSEEDHLLPKKEYFSTAVALGSLKSRASVFDDEDYDEEEEQEEEQAPVEKSLETEKREPVVLKEDKALEYEEEASILDKEDHMDTEDVQEEEVDELLEGTLDDKGATPLPTLYVEDGMVILQFSEIFAIHEPPQKRDRRENRYVTCRDKYKSMDISELVEDDEEVLLKSHGRIDTHVEQADLIQLDVPFPIREGLQLVKASTIGGITPESREFTKLGRDSCIMGELLKQDFIDDNSSLCQSQLSMQVFPLDQHEWERRIIWEHSPEISGNSGEIFEPGLEPEGMLVKGTNSETEQESLNVVNSRVQVQADNNMFVPFSANLLESFGSRGSQSTNESTNKSRHHPQLLRLESQWDENHLSGNDEAGVKKIKRLEKDALGRFSRLVLRERDLGDEAWLDSIIWDSEKELSRSKLIFDLQDEQMVFEIFDNEESKNLQLHAGAMIVSRSSKSKDETFQEGCESNSGWQFNLSNDKFYMNGKSSQQLQANTNKSSVHSLRVFHSVPAIKLQTMKSKLSNKDIANFHRPKALWYPHDNELAIKQQGKLPTRGSMKIIVKSLGGKGSKLHVGIEESVSSLRAKASRKLDFKETEAVKMFYKGKELDDEKSLAAQNVQPNSLVHLIRTKVHLWPWAQKLPGENKSLRPPGAFKKKSDLSTKDGHVFLMEYCEERPLMLSNAGMGANLCTYYQKSSPEDQRGNLLRNQSDTLGNVMILEPGDKSPFLGEIHAGCSQSSVETNMYKAPIFPQRLQSTDYLLVRSPKGKLSLRRIDKIVVVGQQEPRMEVMSPGSKNLQTYLVNRMLVYVYREFFKRGGGEHPIAADELSFLFSNLTDAIIKKNMKIIACWKRDKNGQSYWTKKDSLLEPPESELKKLVAPEHVCSYESMLAGLYRLKHLGITRFTLPASISNALAQLPDEAIALAAASHIERELQITPWNLSSNFVACTNQDRANIERLEITGVGDPSGRGLGFSYVRAAPKAPAAAGHMKKKAAAGRGAPTVTGTDADLRRLSMEAAREVLIKFNVPDEIIAKQTRWHRIAMIRKLSSEQAASGVKVDPTTIGKYARGQRMSFLQMQQQAREKCQEIWDRQLLSLSAFDGDENESENEANSDLDSFAGDLENLLDAEEGGEGEESNISKNDKLDGVKGLKMRRRPSQVETDEEIEDEATEYAELCRLLMQDEDQKKKKKKMKGVGEGMGSYPPPRPNIALQSGEPVRKANAMDKKPIAIQPDASFLVNESTIKDNRNVDSIIKTPKGKQVKENSNSLGQLKKVKILNENLKVFKEKKSARENFVCGACGQHGHMRTNKHCPRYRENTESQPEGIDMDKSAGKPSSSEPSGLPKLKPIKNSKAAPKSAMKTSVDEALKGDKLSSKTGGLPLKFRYGIPAGDLSDKPVSEAPGSSEQAVVSDIDTGIKSTSKISKLKISSKAKPKESKGESERRSHSLMPTFSRERGESESHKPSVSGQPLSSTERNQAASSRHTISIPRPSLSMDTDQAESRRPHLVIRPPTEREQPQKKLVIKRSKEMNDHDMSSLEESPRFESRKTKRMAELAGFQRQQSFRLSENSLERRPKEDRVWWEEEEISTGRHREVRARRDYDDMSVSEEPNEIAEIRRYEEVIRSEREEEERQKAKKKKKKKKLQPEIVEGYLEDYPPRKNDRRLSERGRNVRSRYVSDFERDGAEYAPQPKRRKKGEVGLANILERIVDTLRLKEEVSRLFLKPVSKKEAPDYLDIVENPMDLSTIRDKVRKIEYRNREQFRHDVWQIKYNAHLYNDGRNPGIPPLADQLLEICDYLLDDYEDQLKEAEKGIDPND

>AtHAF2

MICRVDYGSNDEEYDGPELQVVTEEDHLLPKREYLSAAFALSGLNSRASVFDDEDYDEQGGQEKEHVPVEKSFDSEEREPVVLKEEKPVKHEKEASILGNKNQMDTGDVQEELVVGLSEATLDEKRVTPLPTLYLEDDGMVILQFSEIFAIQEPQKKRQKREIRCITYRDKYISMDISELIEDDEEVLLKSHGRIDTHGKKTDQIQLDVPLPIRERSQLVKSGIVRDTTSESREFTKLGRDSCIMGELLKQDLKDDNSSLCQSQLTMEVFPLDQQEWEHLILWEISPQFSANCCEGFKSGLESAGIMVQVRASNSVTEQESLNVMNSGGQTQGDNNNMLEPFFVNPLESFGSRGSQSTNESTNKSRHHPQLLRLESQWDEDHYRENGDAGRENLKQLNSDARGRLSGLALQDRDMWDESWLDSIIWESDKDLSRSKLIFDLQDEQMIFEVPNNKERKYLQLHAGSRIVSRSSKSKDGSFQEGCGSNSGWQFNISNDKFYMNGKSAQKLQGNAKKSTVHSLRVFHSAPAIKLQTMKIKLSNKERANFHRPKALWYPHDNELAIKQQKILPTQGSMTIVVKSLGGKGSLLTVGREESVSSLKAKASRKLDFKETEAVKMFYMGKELEDEKSLAEQNVQPNSLVHLLRTKVHLWPWAQKLPGENKSLRPPGAFKKKSDLSNQDGHVFLMEYCEERPLMLSNAGMGANLCTYYQKSSPEDQHGNLLRNQSDTLGSVIILEHGNKSPFLGEVHGGCSQSSVETNMYKAPVFPHRLQSTDYLLVRSAKGKLSLRRINKIVAVGQQEPRMEIMSPASKNLHAYLVNRMMAYVYREFKHRDRIAADELSFSFSNISDATVRKYMQVCSDLERDANGKACWSKKRKFDKIPLGLNTLVAPEDVCSYESMLAGLFRLKHLGITRFTLPASISTALAQLPDERIAAASHIARELQITPWNLSSSFVTCATQGRENIERLEITGVGDPSGRGLGFSYVRVAPKSSAASEHKKKKAAACRGVPTVTGTDADPRRLSMEAAREVLLKFNVPDEIIAKQTQRHRTAMIRKISSEQAASGGKVGPTTVGMFSRSQRMSFLQLQQQAREMCHEIWDRQRLSLSACDDDGNESENEANSDLDSFVGDLEDLLDAEDGGEGEESNKSMNEKLDGVKGLKMRRWPSQVEKDEEIEDEAAEYVELCRLLMQDENDKKKKKLKDVGEGIGSFPPPRSNFEPFIDKKYIATEPDASFLIVNESTVKHTKNVDKATSKSPKDKQVKEIGTPICQMKKILKENQKVFMGKKTARANFVCGACGQHGHMKTNKHCPKYRRNTESQPESMDMKKSTGKPSSSDLSGEVWLTPIDNKKPAPKSATKISVNEATKVGDSTSKTPGSSDVAAVSEIDSGTKLTSRKLKISSKAKPKASKVESDSPFHSLMPAYSRERGESELHNPSVSGQLLPSTETDQAASSRYTTSVPQPSLSIDKDQAESCRPHRVIWPPTGKEHSQKKLVIKRLKEITDHDSGSLEETPQFESRKTKRMAELADFQRQQRLRLSENFLDWGPKDDRKWRKEQDISTELHREGKVRRAYDDSTVSEERSEIAESRRYREVIRSEREEEKRRKAKQKKKLQRGILENYPPRRNDGISSESGQNINSLCVSDFERNRTEYAPQPKRRKKGQVGLANILESIVDTLRVKEVNVSYLFLKPVTKKEAPNYLEIVKCPMDLSTIRDKVRRMEYRDRQQFRHDVWQIKFNAHLYNDGRNLSIPPLADELLVKCDRLLDEYRDELKEAEKGIVDSSDSLR

>OsHAF701

MGDGRRDNTTSAADDDDDDYDGGGNHGMGNVDDSGDDADYDDAKHAADKGSKDIDIKSAATDSDYDAKADAVDYDIDYDGVAATDHSKKDYSSNAVYASVNSKVSVDNYDDNDNDSDNIVNCTSASADMASNDNAVKMSSSSSSAKMVASKTATSVCIDGSVIKSIGAVRKAKMDRHKRVNKITNTDIVDVRSTINSAKHIKTNDNVSDSDSTSDVARKDSCSMKDKDITAVSVDYHNWNDIVWGNSTTAICTSCAISKSDDHNDAGYVSGCWDVNKHSSSVMADGHTIDSTSYRSNSYSRKTANNSDNNITVKIDTTRHNKSNKGSWDNIVWDSDVKKIDKDDHMIDKNGDHRSHARAMIVTRMKTSAVNVDHNNAIASGRNISNDKYSNRKMSARSHAKKRATMGKVHSVAKTMKKSIKIANHRKAKWYHNKTARGDCSHGMTAIVMTGGKGVKVNATSVKSKASKKKSKIKCSGKDDISAMNVRNSIHVVRTIHWKARGNKRGARKKSDSVKDGHVMYCRANAGMAARCTYYKTSSDTATSRSNSDGGTMAIDADKSGNIRSGSHSCTNMYRAVHKVATTDYVRSKGMSRRIDKYAVGHMVSGTKNMNYINRIVYVYRRARKGIIIRADIITAIVRKRKHCADRKGKGHYIRDRISRRTNVCCYSMAGYRKHGIKTVGASAMNDAIAAAAHIRITSWNTSNVACTNDKNIRITGVGDSGRGGSYVRVTKAVSNSTHKKKSAAAKGTTVTGTDADRRSMDAARKGVIDKTRWHRIAMVRKSSAASGVTMDIVSKARGRMSTKKCIWDRISSAMDGNNGSDTANSDDSAGDNDADDDVGNTDIRSDKMDGMRGKMRRCHTSINIDDVAAAVKSDSDMKRKKVTTNYSTMYNGNKMKGKAGMIKSSVYAGATKSIRAKVNAGSSKRTKTGDANDDIIVKRKNIGKDGKKRGARGDTVCGACGGHMRTNKCKYGDTSMDVNSIRSHDIVSNAIKTSNKRVAKVSSATGSIKAKVVKKCGAKSDRNMSISASVSDKRMMDATDSKSTGKVNKIKISNKIKYDDYDTKSVVIRAVKDRKKIIIKKVGDRTRSGRKTRKIVSSKRDRDDNGSGIINSSHDRGWGVGKRSKGIMSSSWRARRRIARIYDARRDKAKKKNKKKKKHRDDDDRYKNDRRVRGRAAKRRTADMTYTAKRHRGGVSNIKIVDHRTMSCSRKVTKKADYDIIRMDGTIRDKVRKMYKNRDRHDVAIANAHTYNNRHHIADCDYSADVDDAYAID

>SlHAF1

MFGNVDYSGDLDVDYLDEDAKEHLAALADKLGPSLTEIDLSVKSPQESADAAEQDYDEKAEDAVDYEDIDEQYEGPEVQTVTEEDLLLPKRDYFSTEISLTTLENRDSVFDDENYDEDDNEEKEQEVVEKAAEVQSTPVKGEYNNEAEVISLGNKVPEEVISMDAPEFSEDLQEEEPLALEEPVDSQSSLPLPVLCVEDGEAILKFSEIFALHKPRKKAEKRERRCSVPKDKYKAMHTLDIVEEDEVKLLRGSYEEFPWLRMTHVHHDSALTMLDIEPGTVQGTDDLKPTIEKKDPCCSAEPMKENLSMDLCADWSSPICPEFYPFDQQDWEDRIIWDNSPPLSDNTAESCEISEPDYEALTDKQLDVEAESQSLQSEKEIEPHEKGHSSFFSCSVSVEPFGSKQPSGHLDFSLSEGRYHPQLLRLESRLNSDKQKSTDTPKDGDTDEILSSDALKRFTKLTLQNRDILEESWVDNIIWEPDQPFPKPKLIYDLQDEQMLFEVLHNRDDQQLMLHAGAMITTGLVKPSSGDSAELYGLSGLSGRFNIANDKYYLNRKSTQQLKSHSKKRTAHGLKVLHSIPALKLQTMKAKLSNKDIANFHRPRALWHPHDNEVVLKEQRKLPTQGPMKIILKSLGGKGSKLHVAAEETISSLKSKASKKLDFKLSEPVKIIYCGKELEDDKSLSAQNVPPNSVLHLVRTRIHLLPRAQKLPGENKSLRPPGAFKKKSDLSAKDGHVFLMEYCEERPLLLGNVGMGARLCTYYQKLSPNDQQGTLMRNGNTGLGSVLTLDPSDKSPFLGDIKPGCSQSSLETNMYRAPIFQQKVSSTDYLLVRSTKGKLSIRRIDRIDVVGQQEPHMEVTSPGSKGVQTYIMNRLLVYMYREFRAIEKRGSRPSIRADELSAQFPSLSEAFLRKRLKHCADLQRRSNGQFQWVMRFNFRIPSEEELRRLVSPESVCAYESMQAGLYRLKRLGITRLTHPTGLSAAMNQLPDEAIALAAASHIERELQITPWNLSSNFVACTNQDRENIERLEITGVGDPSGRGLGFSYVRTTPKAPIPNAISKKKTVVAKGSTVTGTDADLRRLSMEAAREVLLKFNVPEEQIAKLTRWHRIAMIRKLSSEQAASGVKVDPTTISKYARGQRMSFLQLQQQTREKCQEIWDRQVQNLSAVDGEENESDSEVNSDLDSFAGDLENLLDAEDFEDGEEGSHEPKHDNADGVKGLKMRRRPFQAQVEEEIEDEAAEAAELCRMLMDDDEADRKKKKKDKAMGEQIGFMPDIRYRFSTESTDRGKKPQIFAKPSIKSNGLNVLDFIGDQKELQAEGFATKRTPSSKVKPKKKFDILDSGLFNKKVKILGEGIKPMKEKKSARDSFVCGACGQLGHMRTNKNCPKYGEDVEARAESTDLEKTTGKSMGSIDILDQSQIFSKKIQKSGTKNLMVDVHEDDNSSSKAKVLKVKCASTDKLPDKPTPATSLNSDIPVTSDAEIGTLPPPIKFNKIKFSNKMRAEDDSNEAYKPSILVRPPMETAESHRSKKIVIKQLKDSTSVDEGFLDGSSGMEYRKTKKINELSYMGQQEREYLYEETLGRKKMDDKRLWEEEERRRIAVRQREERAKIYERQKALEEQEKLAAIESYQDAIRREREEEERLKEKKKKKKKTEIRDDYLDDFLPRRNDRRIPDRDRSVKRRQTFESGRHAKEHAPPTKRRRGGEVGLSNILEEIVDTLKNNVNVSYLFLKPVTRKEAPDYHKYVKRPMDLSTIKEKARKLEYKNRGQFRHDVAQITINAHLYNDGRNPGIPPLADQLLEICDYLLEENESILAEAESAI

>LcHAF1

MIVKEAMLPYLLRIDNVGVGRRRRDKLIERAVTISYTCKMSGYDSGSASKDGHDEDDEEEFEDASGGNRLLGFMFGNVDNAGDLDVDYLDEDAKEHLAALADKLGSSLTDIDLSVKSPQTSADAIEQDYDEKAEDAVDYEDIDEQYEGPEIQAASEEDYLLPKKEFFSAEVSLTTLKPVTSIFDDENYDEEEEYEKEHESVDKENECEMEHEVVDKENEVSTVSLSGEEESERGVTVLQGEKSIEDEQQVGSLGTEEDMAVEGDDINQEAADILNGPFDDQVSTPLPVLYVEDGSVILRFSEIFGIREPLKKRGKGDPRYYTHRDKYNIMDFSNLVEEDEEAYLKGSVKCISSIQAHKVQHDNSFLSDDSSEFTKFGVVQDAHPIFDEQRKDSCLSAEPMKEDFMENLSMGWQPMLSPKFFPLDQNDWEEKILWDNSPAVSGNSLESCEIAETETETDTGFIRETELEAGQHNVLSMTPDEKNHNIVMQNTPVLVDPFESRNSLEKTDHVSTESRCHPQLLRLESRLDLHSPNNEDVRKDTVAVELCKSDAVKQFSKLTLQNRDMVEGSWLDNIIWEPEETVGKPKLILDLQDEQMLFEILDDKDSKHLQLHAGAMIVTQSVKPRSVDSFEPPGRKYQSDWQFNIANDKFYMNGKISQQLQSNSNKRTAHGIRVHHSAPALKLQTMKLKLSNKDIAYFHRPKALWYPHDNEVAVKEQGKLPTQGPMKIIVKSLGGKGSKLHVDAEETVSSIKTKASKKLDFKPSETVKLFYLGKELEDHKSLAGQNVQPNSLLHLVRTKVYLLPRAQKLPGENKSLRPPGAFKKKSDLSVKDGHVFLMEYCEERPLLLSNAGMGANLCTYYQKSAPSDQASSMLCNGNSSLGSFLTLEHGDKSPFLGDIKAGCIQSSLETNMYRAPVFPHKVASTDFLLVRSAKGKISIRRIDKIAVVGQQEPLMEVMSPGSKNLQTYSINRLLVYVYREFSAAGKRGMPPCIGVDELATQFPNLSEAIIRKKMKECAFLRRDAKGKQVWSMKRTFNIPAEGDLRKLVSPEHVCAYESMLAGLYRLKHLGITQLTLPASISSAMSQLPDEAIALAAASHIERELLISPWNLSSNFVACTNQDRENIERLEITGVGDPSGRGLGFSYVRSASKAPVSNAMVKKKAAAGRGGSTVTGTDADLRRLSMEAAREVLLKFNVPEEMIAKQTRWHRIAMIRKLSSEQATSGVKVDPATISKYARGQRMSFLQLQQQTREKCQEIWDRQIQSLSAVDDDDENGSDSEANSDLDSFAGDLENLLDAEEFEEGEESNYESKHDKADGVKGLKMRRRPSLAQAEEEIEDEAAEAAELCRLLMDDDEAEQKKKKKKTKAVAEEAGFASAMQSIPGIEIVERVKKPNKIAKQISTFQPIGSYMTNENFRDPKEENLTTKRNVSGKVKPMKKNGVAQVGQLKKLKILGDNVKMFKEKKSSRESFVCGACGQFGHMRTNKNCPKYGADPETQLEIADSEKASGKSNSLDPSSQSQQKKLKKKLISKSATKITVVEAPEDETPSTKAKGLPVKFRCGSTNKPSDTLAQASIQE

>AtHAG1

MDSHSSHLNAANRSRSSQTPSPSHSASASVTSSLHKRKLAATTAANAAASEDHAPPSSSFPPSSFSADTRDGALTSNDELESISARGADTDSDPDESEDIVVDDDEDEFAPEQDQDSSIRTFTAARLDSSSGVNGSSRNTKLKTESSTVKLESSDGGKDGGSSVVGTGVSGTVGGSSISGLVPKDESVKVLAENFQTSGAYIAREEALKREEQAGRLKFVCYSNDSIDEHMMCLIGLKNIFARQLPNMPKEYIVRLLMDRKHKSVMVLRGNLVVGGITYRPYHSQKFGEIAFCAITADEQVKGYGTRLMNHLKQHARDVDGLTHFLTYADNNAVGYFVKQGFTKEIYLEKDVWHGFIKDYDGGLLMECKIDPKLPYTDLSSMIRQQRKAIDERIRELSNCQNVYPKIEFLKNEAGIPRKIIKVEEIRGLREAGWTPDQWGHTRFKLFNGSADMVTNQKQLNALMRALLKTMQDHADAWPFKEPVDSRDVPDYYDIIKDPIDLKVIAKRVESEQYYVTLDMFVADARRMFNNCRTYNSPDTIYYKCATRLETHFHSKVQAGLQSGAKSQ

>AtHAG2

MVQKQQASAGPGTEPKKRRRVGFSPADTGVEANECIKIYLVSSKEEVDSSDISSVKPVDLNDFFDGDGKIYGYQGLKINVWINSISLHSYADITYQSTINGDKGITDLKSALQNIFAETIVDTKDEFLQTFSTQRDFIRNMVSNGEVMHAGATDGSSKNAEVVPSDPQVIRMEIGSPNAGLLYSRLVPLVLLFVDGSNPIDVTDPDWHLYLLIQKKEEKEDPLYRIVGFTAIYKFYRYPDRLRMRLSQILVLPSFQGKGLGSYLMEVVNNVAITENVYDLTVEEPSEKFQHIRTCIDINRLRSFDPIKPDIDSAVQTLTKGKLSKKAQIPRFTPPLNAIEKVRESLKINKKQFLKCWEILIYLALDPIDKYMEDYTSVITNHVRTDILGKDIETPKKQVVDVPSSFEPEASFVVFKSVNGEEANTNVQVDENKPDQEQQLKQLVEERIREIKLVAEKVSKSGQTLKV

>AtHAG3

MATAVVMNGELKKQPRPGKGGYQGRGLTEEEARVRAISEIVSTMIERSHRNENVDLNAIKTAACRKYGLARAPKLVEMIAALPDSERETLLPKLRAKPVRTASGIAVVAVMSKPHRCPHIATTGNICVYCPGGPDSDFEYSTQSYTGYEPTSMRAIRARYNPYVQARSRIDQLKRLGHSVDKVEFILMGGTFMSLPAEYRDFFIRNLHDALSGHTSANVEEAVAYSEHSATKCIGMTIETRPDYCLGPHLRQMLIYGCTRLEIGVQSTYEDVARDTNRGHTVAAVADCFCLAKDAGFKVVAHMMPDLPNVGVERDMESFKEFFESPSFRADGLKIYPTLVIRGTGLYELWKTGRYRNYPPEQLVDIVARILSMVPPWTRVYRVQRDIPMPLVTSGVEKGNLRELALARMDDLGLKCRDVRTREAGIQDIHHKIKPEQVELVRRDYTANEGWETFLSYEDTRQDILVGLLRLRKCGKNVTCPELMGKCSVVRELHVYGTAVPVHGRDADKLQHQGYGTLLMEEAERIARREHRSNKIGVISGVGTRHYYRKLGYELEGPYMVKHLL

>OsHAG702

MDGLAAPSPSHSGATSGGGASHRKRKLPPSSLSDATADEDDDTTAPSSPSTSPSSPSRPSSPSSSHSDDDDDDSLHTFTAARLDGAPPSSSGRPPKPESSTVSAAAAAAAAAAAPKPDSASAAAGDGKEDPKGLFTDNIQTSGAYSAREEGLKREEEAGRLKFLCYSNDGVDEHMIWLVGLKNIFARQLPNMPKEYIVRLVMDRTHKSMMVIRNNIVVGGITYRPYTSQKFGEIAFCAITADEQVKGYGTRLMNHLKQHARDADGLTHFLTYADNNAVGYFVKQGFTKEITLDKERWQGYIKDYDGGILMECRIDQKLPYVDLATMIRRQRQAIDEKIRELSNCHIVYSGIDFQKKEAGIPRRTMKPEDIQGLREAGWTPDQWGHSKSRSAFSPDYSTYRQQLTNLMRSLLKNMNEHPDAWPFKEPVDSRDVPDYYDIIKDPIDLKTMSKRVESEQYYVTLEMFVADMKRMFSNAKTYNSPDTIYYKCASRLESFFSNKVASQLAQASTKN

>OsHAG703

MATAVAAAGGGGGGEQPRRRKPAPGRGGVVLPAGLSEEEARVRAIAEIVSAMGELSRRGEDVDLNALKSAACRRYGLARAPKLVEMIAAVPEADRAALLPRLRAKPVRTASGIAVVAVMSKPHRCPHIATTGNICVYCPGGPDSDFEYSTQSYTGYEPTSMRAIRARYNPYVQARSRIDQLKRLGHSVDKVEFILMGGTFMSLPADYRDYFIRNLHDALSGHTSANVEEAVCYSEHGAVKCIGMTIETRPDYCLGPHLRQMLSYGCTRLEIGVQSTYEDVARDTNRGHTVAAVADCFCLAKDAGFKVVAHMMPDLPNVGVERDLESFREFFENPAFRADGLKIYPTLVIRGTGLYELWKTGRYRNYPPELLVDIVARILSMVPPWTRVYRVQRDIPMPLVTSGVEKGNLRELALARMEDLGLKCRDVRTREAGIQDIHHKIRPDEVELVRRDYAANEGWETFLSYEDTQQDILIGLLRLRKCGRNVTCPELVGRCSIVRELHVYGTAVPVHGRDADKLQHQGYGTLLMEEAERIARKEHRSKKIAVISGVGTRHYYRKLGYELEGPYMVKCLV

>OsHAG704 MALKQKGTDAAADPKKRRRVGFSGIDAGVEANECMKVFIARNPDEAGSANSTSLQPFDLNHFFGEDGKIYGYKNLKINVWISAISFHAYADISFEETSDGGKGITDLKPVLQNIFGENLVEKDEFLKTFSKECEYLSNVVTDGNVIKHDASIDEDSAVEIVRVELQGAAAFLYCRLVPLILLLVEGSTPIDITEHGWEMLLVVKKSAQASSSSNFLVLGFAAVHHFYHYPESTRLRISQILVLPPYQGEGHGLRLLETINSISESENIYDVTIEDPSDYLQYIRSSIDCLRLLTFDPIKPALCSMVSSLKDTNLSKRTSSLKMVPPSDLAETVRQKLKINKKQFLRCWEILIYLNLDAEDRKSMDNFRACIYDRIKGEILGTSTGPNGKRLVQMPSNFDEETCFAVYWTQDGGDADDQTVEQQPEDLKTQEQQLNEVVDSQMEEIVEIAKNVTSRGKDKLSVSCSV

>SlHAG1

MDASHLTAPGRSRSSQSPSPSHSASASATSSIHKRKMASDDHAPPFPSSFSDTRDGALTSNDDLESISARGGGADSDSEDDSEEVGDDDEEDYDASSIRNFTASRLETSVAMVPSGRNTKIKSDNSVKIESSEIAKEVGTGCSGNVAPPTATGSVSGAVVKDESMKNIFTENLQTSGAYTAREESLKREEESGRLKFVCVSNDGVDEHMIWLVGLKNIFARQLPNMPKEYIVRLVMDRNHKSVMVIRRNLVVGGITYRPYTSQKFGEIAFCAITADEQVKGYGTRLMNHLKQHARDVDGLTHFLTYADNNAVGYFVKQGFTKEIYLEKERWQGYIKDYDGGILMECKIDPKLPYTDLSTMIRRQRQAIDEKIRELSNCHIVYPGIDFQKVCTFISEQSSILDID

>SlHAG2

MLLHFNPISTPFTLKPTVHHRNLTVFSQIQPIPIPFTTTNLSISDESLRSKGFNLHSTITDLNLDHLNSVFVAVGFPRRDTEKIQIALENTDSLLWIQYEKTKRPVAFARATGDGVFNAIIWDVVVDPNFQGIGLGKAVMERLVTKLLRKGITNIALYSEPRVLGFYRPLGFVADPDGIRGMVYSRRKNKK

>SlHAG3

MIICSLRAMMQSENSVEDSLHIRKLEVTDKEKGFIELLRQLTVCDSVSDERFKERFEEIAKYGDDHCICVIEDVRLGKIVATGSVFIEKKFVRNCGKAGHIEDVVVDSSARGMQLGKRIVEYLAHHAHSMGCYKVILDCTEENRPFYEKCGFKKKEIQMVKYFV

>SlHAG4

MGTKHHSSSDPISEPKKRRRVGFSKTDAGIEANDCITIYIVSSAEEVDSPNSFCLEPIDLNQFFEDDGRIFGYQGLKITILVSLISFHAYADISFESSSDGGRGITNVKSALENIFGESLVDEKDAFLKTFSTESQYVRSVVSNAETLQHKVSKNGCSTESNCSDVEVFRIAGSPVGQLYSRLVPLVLLLVDGSNPIDVLDPGWEIYLLVQADKLLGFAAVYRFYRYPGSTRMRLGQILVMPPYQRKGYGRFLLEVLNRVAVSENVYDLTIEEPEDSLQHVRLCIDVERLLVFDPVQQSLESVVSHLKQEKLLEKSYMCKYAPPLSAVEDVRKTLKINKKQFTQCWDILIYLRLAPIDKYMEIYQAIVSHRVKAEVLGKDSEGAGKQVIDVPTEHDQEMSFVMFKSRNGQSSSSIETADNQSIVEEQLQKLVDQRMKQIKLIAEKVSSTKAAAMKAEVKFRHEKKESL

>SlHAG5

MQTLHLVSTSTVASSSSSSSLPTIVSLNCCRCQPSNQLPFPNSNLGFLKVKRQPKVSNLKASFWDSIRSGFGKNNIIQVIDTPSSEEEEEEPLPEEFVLVEKTQPDGTVEQIIFSSGGDVDVYDLQDLCDKVGWPRRPLSKLAAALKNSYIVATLHSRKFSSGEEGSGEKKLIGMARATSDHAFNATIWDVLVDPSYQGQGLGKVLIEKLIRTLLQRDIGNISLFADSKVVEFYRNLGFEPDPEGIKGMFWYPMY

>SlHAG6

MEAEKSAESNKKSKNNRDKKMTRKEILEKKKKVQEIIKAAVSQKDLFSSFPTFRHYKKKDLSVYLDSGCGDSLSSPVKQYIQNLLKVNMEASYGTEWPVEEKVKRREMVSSEARYIFVYEISNVDDMLKLSKKERCNADGMDDKGHLVAFVHYRFTIEEEIPVLYVYELQLEHRVQGKGLGKFLMVLIELIAQKSKMSAMVLTVQKANMIAMNFYRNKLRYLISTMSPSQMGLQTNYELLCKSFDYEAKDGLQEGEDK

>SlHAG7

MMAVNEQVRIVIREFNVNKDCRQVEEVERRCEVGPSGKLSLFTDLLGDPVCRVRHSPAYLMLVAEIVVGEESRGIVGMIRGCIKSVTCGKRLWRNNHDFPKLPHQQHLPVFTKLSYILGLRVSPHHRRKGIGLKLVRKMEEWFRENGAEYSYIATENDNQASVKLFTHKCGYAKFRTPSILVQPVFAHRVKISKSVTIFKLTPTEAETLYRHKFSTTEFFPKDMDSILNNKLNLGTFLAVPKGTFTCHWPGINEFLTNPPESWAVLSVWNCKDVFKLEVRGASRMTKGLAKTTRLMDRAFPWLKVPSVPEVFRPFGLHFMYGLGGEGPLSVKLTKALCDLAHNLAAESGCEVVVTEVASCEPLKLGIPHWRKLSCAEDLWCIKRLGEDYSDGSVGDWTKSRPGLSIFVDPREF

>SlHAG8

MSLNISQENLSAYDNTNKKTEIVVRQYDEEKDKLAVEELEGQCDFGQRGQPSLFTDLMGDPISRIRNLPLHVMLVAEYGNNGEIVGVIRGCIKTVTRGNKGSTPCPVYVKIAYILGLRVSSHHRRLGIGTKLVENLEEWSKINGAKYAYMATDSNNEASIKLFVSKCNYAKFRTPSVLVQPVHAHYKPVASDIAIVRVSPQLSESFYRRIFANSEFFPKDIDHILDNKLNFGTFMAVPKKTLFNWDPKSGSFPQTFAILSVWNTKEVYKLQVRGVSSLKYACCLGTRVLDSWMPWLRVPSIPNIFKMFGFYLLYGIHMEGKDGPRLMKSLYAFAHNMGRNDKECRLLVSEVGFDDPVKEAIPRWNKFSWGDLWCMKKLDVDELLSKEDDNWMESQVSNSSSVIFVDPRDF

>SlHAG9

MAEIVELQRNSANWVKVVDDIVRIEKKIFPKHESLARSFDEELKKKNSGLLYTQFGEEIAGYVMYSWPSSLSACITKLAVKENYRGQGLGEKLLKVAIEKCRTRNVHRVSLHVDPTRAAAMQLYKKLRFQVDTLVEGYYSSDRNAYRMYLDFDME

>SlHAG10

MGVGREVAISLDGVRDKNMMQLKKINTAIFPVRYNDKYYTDAIASGDFTRLAYYSDICVGSIACRIEKKESGAVRVYIMTLGVLAPYRGLGTGTKLLNHVLDLCAKQNVTDIYLHVHTINEEAINFYKKFGFEVTDKISNYYTNITPPDCFVLTKVITQTKKQTA

>SlHAG11

MGAGREMLISLDNVRDKNMMQLKKINTALFPIRYNDKYYSDALASADFTKLAYYSDICVGSIACRLEKKEDGAVCVYIMTLGVLAPYRGLGIGTKLLNHVLDLSTKQNSREIYLHVQTSNEDAINFYKKFGFEVTDTIKNYYINITPPDCYVLTKFISQTKK

>SlHAG12

MLNLKLSHNPLIQYRPIRTSDLEVLEKIHGDLFPIRYESEFFQNVVNGRDIVSWGAVDRNRPNEQSDELIGFVTARTVLAKDSDIEEFLRYDSSRSEQTLIYILTLGVADSYRNLGIASSLIREVIKYASNIPTCRAVYLHVISYNNPAIYLYKKMSFQCVRRLSAFYFINDQHYDAYLFIYYVNGGRSPCSPLELIKLIVTGAKSGYRMVAAKLWRKEDRKPSKWSKCKESGSLLPTKHNKRMPTEGALSQFV

>SlHAG13

MAILFTPFSYSFFPKKLNTNFSAINGYTYNFSNRYPFHSSVLCSSQISQQPQICSQTPSPILIDKSFLCVSEAKSENELWAASCLRVRTFYGFHHEILNIEDHTKYLTEREFEALTERIAGKRVGFGRVSCINATLPFSEVSNVAYDLSTSCKFSHDNADLVVVGTLDVNQCIRLPDEITGMKPKGIGADFARGYLSNVCVAGELQRNGLGYALICKAKTVAKDMGISDLYVHVAIDNEPAKKLYIKCGFVQENEEPAWQARFLDRPRRLLLWTDLSNSSDVSM

>SlHAG14

MAAAAVAVAETRKLPRPGRGGVVSLGLTEEEARVRAITEIVNNMVELSRKGKDVDLNALKSAACRKYGLSRAPKLVEMIAALPDSERETLLPKLRAKPVRTASGIAVVAVMSKPHRCPHIATTGNICVYCPGGPDSDFEYSTQSYTGYEPTSMRAIRARYNPYVQARSRIDQLKRLGHSVDKVEFILMGGTFMSLPAEYRDYFTRNLHDALSGHTSANVEEAVAYSEHGATKCIGMTIETRPDYCLGPHLRQMLSYGCTRLEIGVQSTYEDVARDTNRGHTVAAVADCFCLAKDAGFKVVAHMMPDLPNVGVERDLESFKEFFESPSFRTDGLKIYPTLVIRGTGLYELWKTGRYRNYPPEQLVDIVARILSMVPPWTRVYRVQRDIPMPLVTSGVEKGNLRELALARMDDLGLKCRDVRTREAGIQDIHNKIRPEEVELVRRDYTANEGWETFLSYEDTRQDILVGLLRLRKCGRNVTCPELTGRCSIVRELHVYGTAVPVHGRDTDKLQHQGYGTLLMEEAERIARREHRSTKIAVISGVGTRHYYRKLGYELEGPYMVKNLV

>SlHAG15

MAEKISFSTQIFSRVRLATESDVSHLHKLMYQMAKYHNYTKVFNATKASITTNLFKSDNPCPPFYSVTAFILEVSSDSFPQNTLKNPIFDPILKIVNLDSPILDSDLELFRTSNNISSILSEVDNKPLSSDNNVLVAGYVLFYPCYSGYFEKPGFFIENMFVRECYRKCGFGKMLFSAVASQAIKLGFTKVDWLTVGWNENAIDFYLGMGAYIMQDVKRFRLCGDGLDAFATNDQDGGDMV

>SlHAG16

MAAAAPPPPPSPTPDVISEDLLPTGYNVFSRIRLATIVDVPHIHKLIHQMAVFERLTHLFSATESSLSSTLFPENSPPPFTSFTVFLLEVSQTPFPLIDQNYPNFTPIHKTVNLDLPITDPQAESFRSCGNDVVVAGFVLFFPNYSTFLAKPGFYIEDIFVRECYRRKGFGKMLLSAVAAQAAKMGYGRVEWVVLDWNVNAIKFYEEMGAQVMQEWRVCRLTGDALQAFANV

>SlHAG17

MEYSTSSLINSFEPKQKHTTLFNHQISARIRLATELDIPNLHKLVTQMAEYHGLSQIFTTTEASLYNNLFKSQNPPFHSPTALILEISPNPFPHTTTTTTNFVPIIKNNYNFNTLETLDHELESYRSKIIGHDDVYVAGHVLVYPSYNGFFEKPGLFLDQMFVRKCYRGMKFGKLLFSTVAMQAEKMGMGMVDWLVADWNEETINFYEKMGAHYIPDYRLCKLYGDQLQAFGKKSDYEPRIYRE

>SlHAG18

MALISPISSSNGKTFNQKLFSRIRVAIECDVPHIYKLMQQLFVYHDITHLLKSNESSIASGLFNSKYPHLSPVTTLLIEVSTDPFPSCANDKNNSKATNFIESLLSMKELDLNMPLVDEESEQFRVVKHDQHDVFIAGYVMFYPCFSSFFENPVFHMENFFIRECYRRKGFGKWLFSTMALEVARMGFSSIDLYASDWNESTLDFYKHMGATISDDFRVLSLAGKALEAYEDDSN

>SlHAG19

MSTIDNNPLTITEKVYVRVRLANENDIHHIYKLFYQIHEYHNYTHLYKATESSLCDLLFNKTNPSPLFYGPSILLLEVSPTPFLDIDSKNEKFKPVLKEFDLRSNVVDKEADEFKSNSMNDNDKNDVYIAGYSFFYANYSCFYDKAGIYFESLYFRESYRKLGMGRLLFGTVASIAANNGFSSVEGIVAVWNKKSYDFYVDMGVEIFEEFRYGKMVGDALQKYKDI

>SlHAG20

MAPAPSETITTDVSSENNNVTITGKIYTRVRLATKSDLSHIYKLFYQIHEYHNFTHLYKATESSLANLLFKENPLPLFYGPSVLLLEVSPTPFDEPKNTTDEGFKPILTTFDLKFPVVEGEVEEFRSKYDDKSDAYIAGYAFFYANYSCFNDKPGFYFESLYFRESYRKLGMGKLLFGTVASIAANNGFVSVEGIIAVWNKKSYDFYINMGVEVFDEFRYGKLHGENLQKYAHN

>SlHAG21

MASSLSETITTDASSENNNVTITGKIYTRLRLATKSDLSHIYQLFYQIHAYHNNTHLYKATESSLANLLFKENPLPLFYGPSVLLLEVSPTPFNEPTNEGFKPVLTTFDLKFPVVEGQVEEFRSKYDDKSDVYIAGYAFFYVNYSCFSDKPGFYFESLYFRESYRKLGMGSLLFGTVASIAANNGFVSVEGIVAVWNKKSYDFYVNMGVEIFDEFRYGKLHGENLQKYANDKEKNDGGN

>SlHAG22

MTPSLQQPIPSESITSDASSDVTITGKIYTRVRLATKSNLSHIYKLFYQIHEYHNYIHLYRASESSLANLLFKENPLPLFYGPSVLLLEVSQTPFKEPTNEGFKPVLTTFDLKFPVVEGEVEEFRSKYDDKSDAYIAGYAFFYANYSCFNDKPGLYLESLYLRESYRNLGMGKLLFGTVASIAANNGFVSVEEIVAVWNKKAYDLYINMGLEIFDEFRYGKLHGENIKMYADKNVGN

>SlHAG25

MAPALQQSITSDASSDVTITGKIYTRVRLATKSDLSHIYKLFYQIHVYHNYTHLYKATESSLANLLFKENPLPLFYGPSVLLLEVSPTPFDEPKNTTDEGFKPVLTTFDLKFPVVEGEVEEFRSKYDDKSDAYIAGYAFFYANYSCFNDKPGFYFESLYFRESYRKLGMGKLLFGTVSSIAADNGFVSVDGIVAVWNKKSYDFYINMGVEIFDEFRYGKLHGENLQKYAHNKGEIEEETC

>SlHAG26

MAPTSQQPTPSPSLSDSLTTDASSDVTITGKIYTRVRLATKSDLSHIYKLFYQIHEYHNFTHLYKATESSLEGLLFKENPLPLFYGPSVLLLEVSPTPFNEPTNQAFKPVLTTFDLKFPVVEGQVEEFRSKHDDKSDAYIAGYAFFYANYSCFNDKPGFYFESLYFRESYRKLGMGKLLFGTVSSIAANNGFVSVDGIIAVWNKKSYDFYINMGVEIFDEFRYGKLHGENLQKYAHNKGKTEEETC

>LcHAG1

MRTFTAARLETNNSVGSGRNTKLKTENPTVKLENATVKLENADSGKEGGLAGSAAVGVTTGTGGSVPGIVVKEDAVKIFTDNMQASGAYSAREESLKREEEAGRLKFVCLSNDGIDEHMVWLIGLKNIFARQLPNMPKEYIVRLVMDRSHKSVMVIRCNLVVGGITYRPYASQRFGEIAFCAITADEQVKGYGTRLMNHLKQHARDVDGLTHFLTYADNNAVGYFIKQGFTKEIYLEKERWHGCIKDYDGGILMECKIDPKLPYTDLSTMIRRQRQAIDEKIRELSNCHIVYPGIDFQKKEAGIPKKIIKGEDIPGLREAGWTPDQWGHSRFRALNPSADSASNQKFLTTFMRTLLKASS

>LcHAG2

MGQKQQPTADPVTEPKKRRRVGFSAIDAGVDANDCIQIYLASSKEEVGAPDCFSLVPVDLNSFFGEDGKIYGYQGLKITIWVSIVSFNAYADITFQSKSDGGKGITDLKSSLQSIFAETLVENKDDFLQTFSTENDFIRSAVSSGEVLQHQASNGHISHCNSHLEAAGSDLEVVRMVVDNFATGHLYSRLIPLVLLLVDGSNPIDVTDPRWELYLLIQKKKDQQGDIQHRLLGFTAVYRFFHYPDSTRMRLSQILILSSYQRKGYGSYLVEVLSNVAIAENVHDFTVEEPLDSFQHVRTCVDIKHLLAFEPIQNAVKSAVLQLKQGKLSKKIHAPRFVPPADAVEDARKTLKINKKQFLQCWEILIYLGLDSVDKYMEDYVAIVSNRDGEPSSVQMDGNQMNQEEQLQQLVDERIKEIKLIARKVSPHHA

>LcHAG3

MATAVLSDTKKLPRPGRGGFEAHGLTEEEARVRAIAEIVNSMIDLSRKNQTVDLNAIKSAACRKYGLARAPKLVEMIAALPDTERESLLPKLRAKPVRTASGIAVVAVMSKPHRCPHIATTGNICVYCPGGPDSDFEYSTQSYTGYEPTSMRAIRARYNPYVQARSRIDQLKRLGHSVDKVEFILMGGTFMSLPADYRDYFIRNLHDALSGHTSANVEEAVTYSEHSATKCIGMTIETRPDYCLGPHLRQMLLYGCTRLEIGVQSTYEDVARDTNRGHTVAAVADCFCLAKDAGFKVVAHMMPDLPNVGVERDLESFKEFFESPSFRADGLKIYPTLVIRGTGLYELWKTGRYRNYPPEQLVDIVARILAMVPPWTRVYRVQRDIPMPLVTSGVEKGNLRELALARMDDLGLKCRDVRTREAGIQDIHHKIRPEEVELVRRDYTANEGWETFLSYEDTCQDILVGLLRLRKCGRNVTCPELIGKCSIVRELHVYGTAVPVHGRDADKLQHQGYGTLLMEEAEQIARREHRSTKIAIISGVGTRHYYRKLGYELEGPYMVKYLV

>OsHAM701

MGSMEASTAPENGTAAAAAAAASTACNGAGGGGGAAAASNGGGVERRLRSSAASASWASHLPLEVGTRVMCRWRDQKLHPVKVIERRKSSTSSSPADYEYYVHYTEFNRRLDEWVKLEQLDLETVETDVDEKVEDKATSLKMTRHQKRKIDETHVEQGHEELDAASLREHEEFTKVKNIAKIELGRYEIDTWYFSPFPPEYNDSPKLFFCEFCLNFMKRKEQLQRHMKKCDLKHPPGDEIYRSGTLSMFEVDGKKNKVYGQNLCYLAKLFLDHKTLYYDVDLFLFYVLCECDDRGCHMVGYFSKEKHSEESYNLACILTLPPYQRKGYGKFLIAFSYELSKKEGKVGTPERPLSDLGLLSYRGYWTRVLLEILKKHKSNISIKELSDMTAIKADDILSTLQSLDLIQYRKGQHVICADPKVLDRHLKAAGRGGLEVDVSKLIWTPYKEQG

>AtHAM1

MGSSADTETAMIIATPASNHNNPATNGGDANQNHTSGAILALTNSESDASKKRRMGVLPLEVGTRVMCQWRDGKYHPVKVIERRKNYNGGHNDYEYYVHYTEFNRRLDEWIKLEQLDLDSVECALDEKVEDKVTSLKMTRHQKRKIDETHVEGHEELDAASLREHEEFTKVKNIATIELGKYEIETWYFSPFPPEYNDCVKLFFCEFCLSFMKRKEQLQRHMRKCDLKHPPGDEIYRSSTLSMFEVDGKKNKVYAQNLCYLAKLFLDHKTLYYDVDLFLFYILCECDDRGCHMVGYFSKEKHSEEAYNLACILTLPPYQRKGYGKFLIAFSYELSKKEGKVGTPERPLSDLGLVSYRGYWTRILLDILKKHKGNISIKELSDMTAIKAEDILSTLQSLELIQYRKGQHVICADPKVLDRHLKAAGRGGLDVDVSKMIWTPYKEQS

>AtHAM2

MGSSANTETNGNAPPPSSNQKPPATNGVDGSHPPPPPLTPDQAIIESDPSKKRKMGMLPLEVGTRVMCRWRDGKHHPVKVIERRRIHNGGQNDYEYYVHYTEFNRRLDEWTQLDQLDLDSVECAVDEKVEDKVTSLKMTRHQKRKIDETHIEGHEELDAASLREHEEFTKVKNISTIELGKYEIETWYFSPFPPEYNDCVKLFFCEFCLNFMKRKEQLQRHMRKCDLKHPPGDEIYRSGTLSMFEVDGKKNKVYAQNLCYLAKLFLDHKTLYYDVDLFLFYVLCECDDRGCHMVGYFSKEKHSEEAYNLACILTLPSYQRKGYGKFLIAFSYELSKKEGKVGTPERPLSDLGLLSYRGYWTRVLLEILKKHKGNISIKELSDVTAIKAEDILSTLQSLELIQYRKGQHVICADPKVLDRHLKAAGRGGLDVDASKLIWTPYKDQS

>SlHAM1

MGSIDTSTRAESGSDPIPTPNGTTNGLHAGEDSVQTPAVDSTGASAENESLRKRRGSGVLPLEVGTRVMCRWRDGKYHPVKVIERRKFPCGGVNDYEYYVHYTEFNRRLDEWVKLEQLDLNSVETVVDEKVEDKVTSLKMTRHQKRKIDETHVEGHEELDAASLREHEEFTKVKNIATIELGRYEIETWYFSPFPPEYNDCSKLFFCEFCLNFMKRKEQLQRHMRKCDLKHPPGDEIYRSGTLSMFEVDGKKNKVYGQNLCYLAKLFLDHKTLYYDVDLFLFYVLCECDDRGCHMVGYFSKEKHSEESYNLACILTLPPYQRKGYGKFLIAFSYELSKKEGKVGTPERPLSDLGMLSYRGYWTRVLLDILKKHKGNISIKELSDMTAIKAEDILSTLQGLELIQYRKGQHVICADPKVLDRHLKAAGRGGLEVDVSKLIWTPYKEQN

>LcHAM1

MGSLETPTSTENNGTTPGDGGDQKPPVSANGAAPPRLDSTESEASKKRKASLLPLEVGTRVMCRWRDGKYHP

VKVIERRKMHTAGPNDYEYYVHYTEFNRRLDEWVKLEQLDLDSVETVVDEKVEDKVTSLKMTRHQKRKIDETH

VEGHEELDAASLREHEEFTKVKNIATIELGRYEIETWYFSPFPPEYNDCSKLYFCEFCLNFMKRKEQLQRHMRKC

DLKHPPGDEIYRSGTLSMFEVDGKKNKVYGQNLCYLAKLFLDHKTLYYDVDLFLFYVLCECDDRGCHMVGYFSK

EKHSEESYNLACILTLPPYQRKGYGKFLIAFSYELSKKEGKVGTPERPLSDLGLLSYRGYWTRVLLDILKKHKGNISIK

ELSDMTAIKAEDILNTLQSLELIQYRKGQHVICADPKVLDRHLKAAGRGGLEVDVSKLIWTPYKEQG

>LcHDA2

MSLVITESTRKLGKSALYLLLTFVTVKASRLLYRYLRSLTMSSSSSSSSATDTETIRRNRILSSKLYFDIPRSKVPLIYSSAYDISFLGIEKLHPFDSSKWGRICQFLYLDGVLDKKSIVEPLEASKEDLLVVHSKTYLDSLKSSPNVAMIVEVPPVALVPNCLVQKNVLYPFRKQVGGTILAAKLANERGWAINVGGGFHHCSADKGGGFCAYADISLCIHYAFVQLNISRVMIIDLDAHQGNGHEKDFANDRRVYTLDMYNPGIYPFDYEAKQYIDQKVEVVSGTTTDEYMQKLDEALEVAKRKYDPELVIYNAGTDILDGDPLGRLKISPDGIAIRDEKVFRFARLRKIPLVMLTSGGYMKSSARIIANSMINLSRKGLIDMGS

>LcHDA9

MEVKSGETAAPIGRRRVGLLYDERMCRHQTPDGDYHPENPNRIKAIWNKLHAARIPQRCVVLSGKEVEDKHVLSVHSKSHVNLIRNISSKQFGSRRNKIASKINSIYFNEGSSESACLAAGSVIELAERVAKGELNSGFAIVRPPGHHAEKDEAMGFCLYNNVAIAASFLLNEKPELGIKKILIVDWDVHHGNGTQKMFWKDSRVLFFSVHRHEFGTFYPATDDGSYTMIGEGPGAGYNINVPWENGRCGDADYLAVWDHVLVPVAKEFNPDMIIISAGFDAGLEFFPFVVGLC

>LcHDA3

MLMDDKKEKKRQKNFFFFFSDYSTGTYSQTVIDLVESLHVIFEHNMGTESLQPSHFTNGEAENSLHKESFQIPHQDSKRRHGNSVSDGTVTASNENCSEGYLVGLSSPNATDDVDTNSQLGVVVKKARVQREMTVQDMYNQESFDDDDDEDDSDWEPMQKHVEVMKWFCTNCTMANLDDVVHCDICGEHKESGIVRHGFFASPSSPDGLSDVGSEIKDRYKDLHSGISQTNSSTAIGFDERMLLHSEVEMKSHPHPERPDRLQAIATSLATAGIFPGRCYPIASREITREELKMIHSSEQIEAVELTRDMHASYFTPDTYANEHSACAARLAAGLCADLASAIYSGRARNGFALVRPPGHHAGVRQSMGFCLHNNAAVAALAAQAAGAKKVLIVDWDVHHGNGTQEIFDQNKSVLYISLHRHEGGRFYPGTGAANEVGTEGAEGYCVNVPWSRGGVGDNDYVFAFQNVVLPIASEFAPDFTIISAGFDAARGDPLGCCDTLAALTLEVRRVAGERQPDLTGDRMATTGGDAGDDSRRQGGRNLDGRDPREPDLGRRDPQDPLNLCDPQGDPRPMPPDLHTELVKILECKKEYKQIVIPVYYQIDLSDVRNQTECFAAALRKHEECCGKVEKWRKALIEATNLCGLDTRSFK

>LcHDA6

MEERTEGASLPSGPDGKKRRVSYFYEPTIGDYYYGQSHPMKPHRIRMAHNLVVHYALHRRMEINRPFPAGPSDIRRFHTDEYVDFLASVRPESTADPSFSRHLKRFNVGEDCPVFDGLFGFCQASAGGSIGAAVKLNRGDADIAINWAGGLHHAKKSEASGFCYVNDIVLGILELLKVHRRVLYVDIDVHHGDGVEEAFYTTDRVMTVSFHKFGDFFPGTGHIKDVGVGQGKYYALNVPLNDGLDDDSFQGLFRPIIQKVMEVYQPDAVVLQCGADSLSGDRLGCFNLSVKGHADCLRFLRSFNVPLMVLGGGGYTIRNVARCWCYETAVAVGVEPDNQLPFNEYYEYFGPDYTLHVEPCNMENLNSSKDMEKIRSILLEQLSRLSHAPSVQFQTTPPTTEVPEEPEEDMEERPKPRIWNGDDYESDPDEDEKPRIRTSFVDQPVVNVHMRDVEDVKDVKDVKVKTEEHQSS

>LcHDA1

MDTGGNSLPSGADGVKRKVCYFYDPEVGNYYYGQGHPMKPHRIRMTHALLAHYGLLQHMQVLKPFPARDRDLCRFHADDYVSFLRSITPETQQDQLRQLKRFNVGEDCPVFDGLYSFCQTYAGGSVGGAVKLNHGLCDIAINWAGGLHHAKKCEASGFCYVNDIVLAILELLKQHERVLYVDIDIHHGDGVEEAFYTTDRVMTVSFHKFGDYFPGTGDVRDIGYGKGKYYSLNVPLDDGIDDESYHYLFKPIMGKVMEAFRPGAVVLQCGADSLSGDRLGCFNLSIKGHAECVKFMRSFNVPLLLVGGGGYTIRNVARCWCYETGVALGMEVDDKMPQHEYYEYFGPDYTLHVAPSNMENKNSRQMLEEIRNKLLDYLSKLQHAPSVQFQERPPDTELPEADEDHEDGDERWDPDSDMDVDDERKPIPSRVKRELIEPEPKDSDGHKGTAEHPRGFDTNTDEAASTKILDMSSMSIEEPSVKVEQENMAKASDQMYPKWIKSFQAGTQFPSPP

>LcHDA8

MAASPAERIAVFWDDGMLSHDTGKGVFDTGIDPGFLDVLENHPENPDRVKNMLSILKRGPISSHISWHPGRPALLSELLSFHTPEYINELVEADKAGGKMVCAGTFLNSGSWDAALLAAGTSLSAMKHILDGHGKLAYALVRPPGHHAQPTQADGYCFLNNAGLAVQLALDSGCGKVVVVDIDVHYGNGTAEGFYRSDKVLTVSLHMNHGSWGPSHPQRGTVDELGEAEGFGYNLNIPLPNGTGDKGYVYAMTTLVVPAVQKFEPNMIVLVVGQDSSAFDPNGRQCLTMEGYREIGRIVRDLAEMYTDGRVLIVQEGGYHVAYSAYCLHATLEGVLNLPLPLLSDPIADYPEDEAFAVKVIESIKQYQKETIPFLKST

>LcHDA4

MRSKDRISYFYDDFVSFGLCKVCDAGDVGSVYFGPNHPMKPHRLCMTHHLVLSYELHNKMEIYRPHKAYPVELAQFHSADYVDFLHRITPDTQHLFSHELTKYNLGEDCPVFDNLFEFCQIYAGGTIDAARKLNNQLCDIAINWAGGLHHAKKCEASGFCYINDLVLGILELLKHHARVLYIDIDVHHGDGVEEAFYFTDRVMTVSFHKFGDMFFPGTGDVKEIGEREGKFYAINVPLKDGIDDTSFTRLFKTIISKVVETYIPGAIVLQCGADSLAGDRLGCFNLSIDGHAECVRFVKKFNLPLLVTGGGGYTKENVARCWTVETGVLLDTELPNEIPDNEYIKYFAPECSLRIPSGHIENLNSKSYLSTIKMQVLENLRCIQHAPSVQMQEVPPDFYIPDFDEDEQNPDERIDQHTQDKQIQRDDEYYEGDNDNDHMDVSSNPMAAFEAVAQLISCRPLCFDACVNTSNKRFRRIQFYNGSRNRFSVRRWNSRRCNCQRGESVGGLSADDGRLQTSFLVNGQSNVQNIELDEKLNNRNGDLVANRLDSVEAKSVEDEARDLLRESIVCYCGNPVGTIAANDPNDSIILNYDHVFIRDFIPSGIAFLLMGEYDIVRNFILHTLQLQSWEKTMDCHSPGQGLMPASFKVRTVPLDGDDTATEEILDPDFGEAAIGRVAPVDSGLWWIILLRAYGKCSGDLTVQERIDVQTGIKMILKLCLADGFDMFPTLLVTDGSCMIDRRMGIHGHPLEIQALFYSALLSAREMLAPEDGSADLIRALNNRLVALSFHIREYYWIDMKKINEIYRYKTEEYSYDAVNKFNIYPDQIPPWLVEFMPNRGGYLIGNLQPAHMDFRFFSLGNLWSIVSSLATPDQSHAILDLVEAKWAELVADMPFKICYPALEGQEWRIITGSDPKNTPWSYHNGGSWPTLLWQLTVACIKMGRSEIAEKAVKLAERRIARDKWPEYYDTKRGRFIGKQARLFQTWSIAGYLVAKLLLADPNAAKILVNEEDSELQNAFSCMISANPRRKRGRKQTFIV

>LcHDA7

MRVGKQLHVCKSVSMELQTFGSSCLLGSVHLYLLRNPRVLGHSHSTKHPTFTRKSNGGGSILCTYNQNLQPSDARVIFSVAPAMGHNQESHPESHFRVPAIVSALENMQLTPTFRGSEIIELQNFKPASVDDIASVHTRAYISGLEKAMDQASQRGVILIEGSGPTYATPTTFQESLVAAGAGISIVDSVVAASKSRPDPPVGFALIRPPGHHAIPKGPMGFCVFGNVAIAARYAQRVHGLKRVFIIDFDVHHGNGTNDAFYDDPDIFFLSTHQDGSYPGTGKFDEVGHGDGEGTTLNLPLPGGSGDIAMRTVFDEVIVPCAQTFKPDIILDGSYPGTGKFDEVGHGDGEGTTLNLPLPGGSGDIAMRTVFDEVIVPCAQTFKPDIILVSAGYDGHVLDPLASLQFTTGTYYMLASNIKQLAKDLCGGRCVFYLEGGYNLKSLSYSVADSFRAFLGEPSMASEFDDPAILYEEPSTKVKQAIQRIKHIHSL

>LcSRT2

MLLWVLDIVESGMRGWQSLSGGRRIIFYLGSVKSVQTSCRISVPGASLGNVEKEPPKFLRDKKVVPDADPPSTEDVDQLYQFFDQSTKLIVLTGAGISTECGIPDYRSPNGAYSSGFKPITHQEFVRSSRARRRYWARSYAGWKRFIAAQPSSAHFALASLEKAGRIEFMITQNVDRLHHRAGSNPLELHGTVYTVGCLDCGFSIGRDLFQDQVKALNPKWAAAIESLDYGSPGSDKSFGMKQRPDGDIEIDEKFWEEEFHIPTCQKCNGVVKPNVVFFGDNVPKDSADKAMEAAKQCDAFLILGSSLMTKSAYRLVRAAHEAGASTAIVNIGSTRADDFVPLKINARLGEILSRVLNVGSLSVPPLQ

>LcSRT1

MSLGYAEKLSFIEDVGNVGMAEFFDTPKALQEKKHIDRFPEIGFLSNVDHIVVNGAETLEQWMFDIDQLAKLIEKSKHLVVFTGAGISTSCGIPDFRGPKGIWTLQREGKPLPEASLPFHRAMPGVTHMALVGLEKAGILKFVISQNVDGLHLRSGIPREKLAELHGNSFMEACPSCGTEYMRDFEVETIGLKETSRRCSDTKCGAKLRDTVLDWEDALPPKEMNPAEKHCRMADVVLCLGTSLQITPACNLPLKSLRGGGKIVIVNLQKTPKDKKASLVIHGLVDKVIAGVMDLLSLRIPPYVRIDRFQIIVTQSLSADKKFVNWTLRVGSVHGPNAPVPFIKSIEVSFAETQKYKAANLDRQPFQLKRRTITNEAFDITLKLNFSDGCGCLSTQIDIPFDFKVSTKGFDLDKEAIFQKLRDTAVHDLRCGQNAVIEKQAISSPKSDVTTYAIVTNIKTFESQLQSNGDLKWPKDSVNGTETSRKRSNSRKRKSRP

>LcHDA5

MLGTAINDPLGGCRVTPYGYTILLKKLMDFAQGKILLALEGGYNLDSISNSVLACMEVLLEDKPINGSSKAYPFESTWRVIQAVRHKLSAFWPTLADELPTKLTNQKAPPPHMISNSDSDDEDDETPHVSKNLVAAVQDVIEPLLKLKVEDGHAQLVSKSIPWGLEAANVEIWYATFGSNMWKSRFLCYIEGGQVEGMKRACSGSMDRNPPKEILWKTFPHRLFFGLESTRTWGPGGVAFLNPESNSEDRTYMCLYRITLEQFNDVLHQENYGYDMSSPLFDMTDLNSVTTKRSISLEALKKGWYHNVVYLGKECDIPILTMTCSLKDVESFKSGKHPIRAPSTKYADTLIKGLVEGQQLSEEEAKAYIQEASTKPL

>SlHDA1

MDVGGNSLASGADGKKRKVSYFYDPEVGNYYYGQGHPMKPHRMRMTHALLAHYGLLQHMHVLKPNPARDKDLCRFHADDYIAFLRSVTPETQQDQLRQLKRFNVGEDCPVFDGLYSFCQTYAGGSVGGAVKLNHGHCDIAVNWAGGLHHAKKCEASGFCYVNDIVLAILELLKVHERVLYVDIDIHHGDGVEEAFYTTDRVMTVSFHKFGDYFPGTGDVRDIGYGSGKYYSLNVPLDDGIDDESYQSLFKPIMGKVMEVFKPGAVVLQCGADSLSGDRLGCFNLSIKGHAECVKYMRSFNVPLLLLGGGGYTIRNVARCWCYETGVALGIELEDKMPQHEYYEYFGPDYTLHVAPSNMENKNSRQILEDIRSKLLDNLSKLQHAPSVQFQERPPDTELPEADEDLEDADSRWDADSDTNGEERKPNPSRVRREHVEPEGKGTDDMKTEEHLREVESTFAESTSLKGGNSSSTLIDGGQIKVEQGNSNKLFDQPTDIIS

>SlHDA2

MDSSVVEGGASLRSTGTDATKRRVSYFFDSSIGEYDYGEGHLMKPHRIRVAHNLILNYNLHRKMEIIEPFPATKEEIGSFHSSDYVEFLSSVSPETINDKYDSYQRRRFNVGLDSESFDCPVFYGLFDFCQTSSGGSIGAAAKLNRNEADIAINWAGGLHHAKKSEASGFCYVNDIVLGILELLKVHKRVLYVDIDVHHGDGVEEAFFTTDRVMTVSFHKFGDFFPGTGHIKDIGASTGKYYALNAPLGNGIDDESFRSLFRPVIQKVMEVYQPEAVVVQCGADSLAGDRLGVFNLSVKGHADCIRFLRSFNVPLMMLGGGGYTVKNVARCWCYETAVAVGVELDNDLPYNEFYEYFAPDYILYHESLHMKNENSPSELERIRNTLLEQLSRLPHVPSVPFQVTPSVTEVPEKEDENMDQRPKPEISQDYDTDDEEKSNNGKFSNYYNL

>SlHDA3

MDSSTVDGGASLPSTGTDARKRRVSYFYEPTIGDYYYGQGHPMKPHRIRMAHNLIVNYYLHRRMEISRPFPAGEDDIRRFHSPDYVDFLATVSPETLHDHTHSRHLKRFNVGEDCPVFDGLFGFCQASAGGSIGAAVKLNRQDADITINWAGGLHHAKKSEASGFCYVNDIVLGILELLKVHKRVLYIDIDIHHGDGVEEAFFTTDRVMTVSFHKFGDFFPGTGHIKDIGANQGKYYALNVPLHDGMDDDSFGRLFRPTIQKVMEVYQPEAVVLQCGADSLAGDRLGCFNLSVKGHAACLRYLRSFNVPLMVLGGGGYTIRNVARCWCYETAVAVGVEPENKLPYNEYYEYFGPDYTLHVEPIPMENLNSPRDLEKMRNILLEQISQLPHAPSVPFQTTPSTTEVPEEKEENMDRRPKPRIWNGDGYESDADEDEKPRQRSSDSNLTPVESSDMRDVDDQANADDMVDDHP

>SlHDA4

MRSKDKISYFYDGDVGSVYFGPNHPMKPHRLCMTHHLVLAYGLHSKMEVYRPHKAYPVELAQFHSADYVEFLNRITPDTQNLFPSEMARYNLGEDCPVFDNLFEFCQIYAGGTIDAARRLNNKLCDIAINWAGGLHHAKKCAASGFCYINDLVLGILELLKYHPRVLYIDIDVHHGDGVEEAFYFTDRVMTVSFHKYGDKFFPGTGDMKDTGERDGRFYSINVPLKDGIDDGSFTRLFKTIISKVVETYLPGAIVLQCGADSLAGDRLGCFNLSIDGHAECVRFVKKFNLPLLVTGGGGYTKENVARCWALETGVLLDTELPNEIPDNDYIKYFAPDYSLKLPGGHIENLNSKSYIGTIKMQVMENLRCLQHAPSVQMQEVPPDFYIPDFDEDEQNPDERVNQHTQDKHIQRDDEYYEGDHDNDNHTDDA

>SlHDA5

MSSAASSSKTDDAEALRRSRILSSPLYYDVPPSKVPLIYSSSYDIAFFGIEKLHPFDSSKWGRICRFLTKEGIMDQKHVVEPVEATKDDLLVVHSESYLKSLNSSLNVSMIIEVPPVAMLPNCLVQKKVLHPFRKQVGGTILAAKLAKERGWAINVGGGFHHCSSEKGGGFCVYADISLCIHFAFVRLNISRVMIIDLDAHQGNGHEMDFSDDRRVFILDMYNPRIYPLDFEARRYIDLKVEVKSGTATDEYLTKLDHALEVAEKRFDPDFIVYNAGTDILDGDPLGRLKISPDGIASRDEKVFRFARERSIPLIMLTSGGYMKSSAKVIADSIVNLSSKSLIDMKS

>SlHDA6

MASSASQSLPSKNNLIHVFWDEGMLKHDTGRGVFDTGMDPGFLDVLEKHPENSDRVKNMLSILKRGPISPFISWHHGRPAHVSELLSFHTQEYVNELIEADRNGGKELCGGTFLNPGSWHATLLAAGTTLSAMKLIIDGHGNVAYALVRPPGHHAQPTQADGYCFLNNAGLAVQLALDGGCRKVVVLDIDVHYGNGTAEGFYHFDKVLTISLHMNHGSWGPSHSQGGTIEELGEGEGFGYNLNIPLPNGTGDKGYGYAMQHLVVPAIEKFEPDMMVLVVGQDSSAFDPNGRQCLTMEGYREIGQTIRGMANKYSNGRLLIVQEGGYHVTYSAYCLHATLEGVLNVSDPLLSDPIAYYPEDESFPAKVIDAIKKYQREVVPFLRDA

>SlHDA7

MQTFQESLLAAGAGISLVDSVVAASRVSKDPPVAFALIRPPGHHAVPKGAMGFCVFGNIAIAARYAQRMHGLQRVFIIDFDVHHGNGTNDAFYEDPDIFFLSTHQAGSYPGTGKIDQIGCGSGEGSTLNLPLPGGSGDTAMRTVFDEVIVPCAQRFKPDIILVSAGYDAHLLDPLASLQFTTGTYYMLASSIKQLAKDLCGGRCVFFLEGGYNLSSLSNSVAESFRAFLGDRSLASELDDPSYLHEEPLKKVKQIIEKVKHIHSF

>SlHDA8

MILVQKCVTNSEGNEISESSKMSNLTGGKTETNLPNQSGVALCQDLNGRLDGVGSAVVCDYTCHNDGQSGEVSQLGTRDSDGISGLNADPAAITKSAKHESDMTLEDMYNARYNFDEDDDDSDWEPSEKQIEVLKWFCVNCSMINVEDVGNCEVCGEHRESGILRHGFFASPYLPVEDINQDELPVTEDSEDSCKQSSLSSSTAVGFDERMLLHTEVVLKSHPHPERPDRLRTIAASLATAGIFPGKCHPIPAREITREELQMIHSEENIEAVDNTKRMHASYFTPDTYANEYSACAARLAAGLCADLASAIYSGCVKNGFALVRPPGHHAGVKQAMGFCLHNNAAIAASAAQAAGAKKVLIVDWDVHHGNGTQEIFERSKSVLYISLHRHEGGRFYPGTGAADEVGSMGAEGYCVNIPWSRGGVSDNDYIFAFEQVVLPIALDFNPDFTIISAGFDAARGDPLGCCDVTPAGYASMTQMLSALSGGKLLVILEGGYNLRSISSSATAVIKVLLGESPVIDIDKAVPSKAGLRSVLDVLKIQMNFWPTLEANFTKLHSQWGSYAFQDTREQSKKRRRTGLPIWWRLGRKRLLYRVLSKQLRAKSSCNFSC

>SlHDA9

MDSGERRRVGLIYDKRMKKHFDLEEVKHPESADRIQKIWNKLKDSGIAKRCTVMGGKEAKDEHISLVHNRKHIDFIKSISSKEEMFWKEMAKKYNSVYFNQGSSEAAYISAGCVIEVAEKVAEGELDSAFAIVRPPGHHAEPNQPMGFCLFNNVAIATKYLLDKRADLGIKKILIVDWDVHHGNGTQKMFWKDPRVLFFSVHRHDFGTYYPCDEAGSHKMTGEGAGKGYNINVPWEHGGCGDADYLAVWDHVLIPVAKEFGPDIILISAGFDAAIGDPLGGCCVSPHGYAVMLNKLLEFAGGKIVMALEGGYDKVSIANSVQACMEVLLKQKPIIGSSEAYPFESSWRVIRAVRKTLSPFWQSLSERLPVKITSMSSPYIQVVSSDSEDEYNTFSNRVSQHLEMDIENVIQPFSDLKVNDDTGQASNNSHTWREELSKIDIWYATFGSNMKLSRFLCYIEGGQVEGMRKPCIGSLDKSKPKEIKWESFPHRLFFAREHTATWGPGGVAFLHPECNSDEKAYLCLYRITLEQFNDVLLQENTSSFDMNYPLFDMTDLQSIEERKCVPVEAVKNGWYHNVLYLGKENGIPILTMTCQLSDVDNFKSGKVGMCKPSKEYANTLIRGLVEGKQLSEEEATTYIQEAASRAL

>SlHDT1

MEFWGAEVKSGEPLTVQPGDGMVLHLSQASLGELKKDKSESACLSVNIDGKKLVLGTLNSEKVPQQQFDLVFDKDFELSHNLKSGSVYFFGYKATNPFDEEEDDEDDYDDSDEDIPLTLANSGKPDPKEAGKSNAGKDSASGKQKVRIVEPTKDDEDESSDDDGSDLGEDEDDSDESEEETPKKAEPAKKRKADSATKTPVTDKKAKLTTPQKTDGKKGGVHVATPHPSKQASKTPKSAGSHHCKPCNRSFGSEGALESHSKAKHSAGK

>SlHDT2

MEFWGAEVKSGQPLSVQPGDDMVLHLSQASLGEVKKDKASEPVCLSVTIDGKKLVLGTLSLDKLPQQQFDLVFDRDFELSHNWKNGSVYFFGYKAANPFEEYPFLFFYFLFVSLTTIVCDTEEDVDEVEDESDEEIPLSIANNGKTEAKASAKDSASTKQKVRVIEFTKVDKAEDQDESTDESADEDDSMMGEDEEGDSDEDQSDESEEETPKKAEPSRKRPADSAAKTPVPDKKTKFLTPQKTDGKKGAVHVATPHPSKQAGKTPGNKPNQTPKSGGSLACKTCNRTFGSENALESHSKAKHSAGK

>SlHDT3

MEFWGVTSLSHELCIGVEVKAGQTLKVKPELFKLIHISQAAIGEVKDVKEAKYVPLRLTVGDNKYVIGTLSAEDRPQLMFDLVFEKEFELSHGWKNGSVHFMGYSADDPSSEEIDSGDDVFSDEENVMEALNGKLEADVKDVKPDAKKSAPAKDEKNAKVAESKKETESDEDDDDSEDDSDDEDDSEDDSDDVPLGMDGPEGMDLSDDSEDDDDSEEDDDSEEETPKKVEQKKRPAPSPKVAPGSGKKAKQATPDNKSGGKKGPATPSAKQNGKPAFNGNNKPKTQSPKSGGQFSGNKSNNKNFSGQKNFKGKQGRK

>SlSRT1

MSLGYAEKLSFIEDVGNVGMTEYFDPPLLLQDKIERLAVMIQKSKHLVVFTGAGISTSCGIPDFRGPKGIWTLQREGKALPEASLPFHRATPSMTHMALVELEKAGFLKFLISQNIDGLHLRSGIPREKLSELHGDSFMERCPSCGIEYMRDFEIETIGLKETARRCSKVGCGARLKDTVLDWEDALPPKEMNPAERHCKMADVVLCLGTSLQITPACNLPLKSLKGGGKIVIVNLQKTPKDKKASLLIHGLVDKVITGVMEFLSLRIPPFIRIDLLQTIFTQASSLDEKYVNWSLAVASVHGNRAPLPFIKSVEVSFSESQNMKAAVLDKQPLYLKRRTVKSTNPFNIMMKLNFSDGCKCSSAEIMIPIDFKISADVFKDDKDSILQNLRESALTDPSCGQTSVIEKKVIMVPKSEVIVHAIVTNIVKFDRSYDGDLSNGSFKRKYECLNGVIPSRKRSNGRKPRTVINVR

>SlSRT2

MSLRLCCRPSISGFKNKRDLLGLELAAYQSSKTMGKWLSGVKKFIPFEGYVKSVKTAARISFPKISADCQDKEPSNFLSHKKMVPYSNPPSTEDVDSLYEFFDRSTKLVVLTGAGMSTESGIPDYRSPNGAYSTGFKPITHQEFLRSIKARRRYWARSYAGWRRFTAAQPSTGHIALSSLEKAGHISFMITQNVDRLHHRAGSNPLELHGTVYIVACTNCGFSLPRDLFQDQVKAHNPKWAEAIENLDYDSRSDKSFGMKQRPDGDIEIDEKFWEEDFYIPECQSCQGVLKPDVVFFGDNVPKSRADAAMEAAKGCDAFLVLGSSLMTMSAFRLIKAAREAGAATAIINIGATRADDIVPLKISARVGEILPRLLNVGSLSIPAP

>AtHDA2

MTHTRVISTWTELTRDLAIYLLFTFFAIKVFKFLFSCNRTSEISSFSMATHPEALRRERILNSKLYFDVPLSKVSIIYSSSYDISFMGIEKLHPFDSSKWGRVCKFLVSDGFLEEKAIVEPLEASKIDLLVVHSENYLNSLKSSATVARITEVAPVAFFPNFLVQQKVLYPFRKQVGGTILAAKLATERGWAINIGGGFHHCTAERGGGFCAFADISLCIHFAFLRLRISRVMIIDLDAHQGNGHETDLGDDNRVYILDMYNPEIYPFDYRARRFIDQKVEVMSGTTTDEYLRKLDEALEVASRNFQPELVIYNAGTDILDGDPLGLLKISPDGITSRDEKVFRFAREKNIPLVMLTSGGYMKSSARVIADSIENLSRQGLIQTRPE

>AtHDA5

MAMAGESSGKKIGDCDGKVAGNRQRKVGLIYDETMCKHDTPDGEDHPECPDRIRVIWEKLQLAGVSQRCVVLGSSKAEDKHLQLVHTKDHVNLVKSISTKQKDYRRNRIASQLNSIYLNGGSSEAAYLAAGSVVKLAEKVAEGELDCGFAIVRPPGHHAEADEAMGFCLFNNVAVAASFLLNERPDLGVKKILIVDWDVHHGNGTQKMFWKDPRVLFFSVHRHEYGGFYPAGDDGDYNMVGEGPGEGFNINVPWDQGRCGDADYLAAWDHILIPVAREFNPDVIFLSAGFDAAINDPLGGCCVTPYGYSVMLKKVGVELMEFAQGKIVLALEGGYNLDSIAKSSLACVQVLLEDKQIQGPPEAYPFESTWRVIQAVRKRLCTYWPSLADELSWKLINQKTPTPIILISSSDSETEDNAQGLLDQMSKLSIENPQGTLLENHQVEPASTSWRADLAKVDVWYASFGSNMWKPRFLCYIQGGQVDGLKKVCVGSMDKSPPKETVWETFPHRLFFGRESSVGWGVGGVAFTNPLANLIDQTHMCLYRITLEQFNDVLSQENGLNVDSDSPVFDLAALQLVDNKGSILEAPLNSWYGNVVCLGKERDIPILTMTCTLSAVEKFKSGEIPIRPPAKAYANTLIRGLVEGGRLSKEEAEAYIDKAVSKPL

>AtHDA6

MEADESGISLPSGPDGRKRRVSYFYEPTIGDYYYGQGHPMKPHRIRMAHSLIIHYHLHRRLEISRPSLADASDIGRFHSPEYVDFLASVSPESMGDPSAARNLRRFNVGEDCPVFDGLFDFCRASAGGSIGAAVKLNRQDADIAINWGGGLHHAKKSEASGFCYVNDIVLGILELLKMFKRVLYIDIDVHHGDGVEEAFYTTDRVMTVSFHKFGDFFPGTGHIRDVGAEKGKYYALNVPLNDGMDDESFRSLFRPLIQKVMEVYQPEAVVLQCGADSLSGDRLGCFNLSVKGHADCLRFLRSYNVPLMVLGGGGYTIRNVARCWCYETAVAVGVEPDNKLPYNEYFEYFGPDYTLHVDPSPMENLNTPKDMERIRNTLLEQLSGLIHAPSVQFQHTPPVNRVLDEPEDDMETRPKPRIWSGTATYESDSDDDDKPLHGYSCRGGATTDRDSTGEDEMDDDNPEPDVNPPSS

>AtHDA7

MASLADGGKRRVSYFYEPMIGDYYYGVNQPTKPQRIRVTHNLILSYNLHRHMEINHPDLADASDFEKFHSLEYINFLKSVTPETVTDPHPSVSENLKRFNVDVDWDGPVFHNLFDYCRAYAGGSISAAAKLNRQEADIAINWAGGMHHVKKDKASGFGYVNDVVLAILELLKSFKRVLYIEIGFPHGDEVEEAFKDTDRVMTVSFHKVGDTGDISDYGEGKGQYYSLNAPLKDGLDDFSLRGLFIPVIHRAMEIYEPEVIVLQCGADSLAGDPFGTFNLSIKGHGDCLQYVRSFNVPLMILGGGGYTLPNVARCWCYETAIAVGEQLDNDLPGNDYMKYFRPDYKLHILPTNRQNLNTRLDIITMRETLLAQLSLVMHAPSVPFQDTPSSSQATEAAEVDMEKRNDPRI

>AtHDA8

MVTNRVDVFWHEGMLRHDAVEGVFDTGYDPGFLDVLEKHPENADRVRNMLSILRRGPIAPHVNWFTGLPAIVSELLMFHTSEYIEKLVEADKSGERCEIAAGTFMSPGSWEAALLAAGTTLSAMQHILDCHGKIAYALVRPPGHHSQPTQADGYCFLNNAALAVKLALNSGSCSRVAVIDIDVHYGNGTAEGFYTSDKVLTVSLHMNHGSWGSSHPQKGSIDELGEDVGLGYNLNVPLPNGTGDRGYEYAMNELVVPAVRRFGPDMVVLVVGQDSSAFDPNGRQSLTMNGYRRIGQIMRGVAEEHSHGRLLMVQEGGYHVTYAAYCLHAMLEGVLKIPEPHLSDPIAYYPEEEANAVAAVESIKTYHTEFVPFLRGT

>AtHDA9

MRSKDKISYFYDGDVGSVYFGPNHPMKPHRLCMTHHLILAYGLHSKMEVYRPHKAYPIEMAQFHSPDYVEFLQRINPENQNLFPNEMARYNLGEDCPVFEDLFEFCQLYAGGTIDAARRLNNKLCDIAINWAGGLHHAKKCDASGFCYINDLVLGILELLKHHPRVLYIDIDVHHGDGVEEAFYFTDRVMTVSFHKFGDKFFPGTGDVKEIGEREGKFYAINVPLKDGIDDSSFNRLFRTIISKVVEIYQPGAIVLQCGADSLARDRLGCFNLSIDGHAECVKFVKKFNLPLLVTGGGGYTKENVARCWTVETGILLDTELPNEIPENDYIKYFAPDFSLKIPGGHIENLNTKSYISSIKVQILENLRYIQHAPSVQMQEVPPDFYIPDFDEDEQNPDVRADQRSRDKQIQRDDEYFDGDNDNDAS

>AtHDA10

MAFSMLFTGHAECGGYTKENVARCWTVETGILLDTELPNEIPENDYIKYFAPDFSLKIPGGHIENLNTKSYISSIKVQILENLRYIQHAPSVQMQEVPPDFYIPDFDEDEQNPDVRVDQRSRDKQIQRDDEYFDGDNDNDAS

>AtHDA14

MSMALIVRPFFVPGSAGISGSRNICKKNQWRKYLLKPSGSSINCSFSTEKNPLLPSIQQLADARLIYSVSAALGHNKESHPECSARVPAIVNALEMNELTPKFRGSQILELANFKTATVEDIANVHDKAYVFGLEKAMDEASDSGLIFIEGSGPTYATSTTFQDSLIAAGAGMALVDSVIAASRNSVDPPIGFALIRPPGHHAVPKGPMGFCVFGNVAIAARHAQRTHGLKRIFIIDFDVHHGNGTNDAFTEDPDIFFLSTHQDGSYPGTGKISDIGKGKGEGTTLNLPLPGGSGDIAMRTVFEEIIVPCAQRFKPDIILVSAGYDAHVLDPLANLQFTTATYYSLAKDIKRLAKEVCGGRCVFFLEGGYNLESLSSSVADSFRALLGEDSLASEFDNPAYLYDEPMRKVRDAIQRAKSIHCL

>AtHDA15

MVVETIERSCEGSKRRHVNGGDIAVPCSGEECSNGDINVAPGVSAKRARVSREMTFEDIYGADALLNDDDDEDDDCDWEPVQAPMEFVKWCCVNCTMSNPGDMVHCCICGEHKESGILRHGYLASPFFKDTGLIEVEEKYGGSSSATSSTAVGFDERMLLHSEFEVKAQPHPERPDRLRAIAASLATAGVFPGRCLPINAREITKQELQMVHTSEHVDAVDTTSQLLYSYFTSDTYANEYSARAARLAAGLCADLATDIFTGRVKNGFALVRPPGHHAGVRHAMGFCLHNNAAVAALVAQAAGAKKVLIVDWDVHHGNGTQEIFEQNKSVLYISLHRHEGGNFYPGTGAADEVGSNGGEGYCVNVPWSCGGVGDKDYIFAFQHVVLPIASAFSPDFVIISAGFDAARGDPLGCCDVTPAGYSRMTQMLGDLCGGKMLVILEGGYNLRSISASATAVIKVLLGENPENELPIATTPSVAGLQTVLDVLNIQLEFWPSLAISYSKLLSELEARLIENKSEKSDEKEGCSGSDMVEMGTKEAFVQLSLGSYDFKIKVILESILPNPS

>AtHDA17

MAFSMLFTGHAECVKFVKKFNLPLLVTGGGGYTKENVARCWTVETGILLDTELPNEISENDYIKYFAPDFSLKIPGGHIENLNTKSYISSIKVQILENLRYIQHAPSVQMQEVPPDFYIPDFDEDEQNPDVRVDQRSRDKQIQRDDEYFDGDNDNDAS

>AtHDA18

MLLKFEASSELRLVDPSVSLTVLRKIRLSHLPDMTMTSESSGKKCGEGDGKVAGKSQRKVGLVYDETMCKHDTPNGKVDVECPDRIRVIWEKLQLAGVTQRCVVLGGSKAEDKHLKLVHTKKHVNLVKSISTKKKDSRRNKIASQLDSIYLNGGSSEAAYLAAGSVVKVAEKVAEGELDCGFAIVRPPGHHAESDEAMGFCLFNNVAVAASFLLNERPDLDVKKILIVDWDIHHGNGTQKMFWKDSRVLIFSVHRHDHGSFYPFGDDGDFNMVGEGPGEGFNINVPWEQGGCGDADYLAVWNHILIPVTKEFKPDIILLSAGFDAAIGDPLGGCCVTPYGYSVMLKKLMEFAHGKIVLALEGGYNLESLGKSSLACVQVLLEDKQIHGSSETYPLESTRRVIQAVRERLCTYWPSLDASMASNENLKNPSAERNSADALLREVEELKSLMAARDGELEARRKELKAKNKELEANEKELEAGLMLIRAREDVICGLHAKIESLQQERDEAVAKAERIDKELQEDRARSQEFKEDTEFCLSTLRREKELAIMAKNKDLEAKEKELEARLMLVHAREDKIHAKIERLQQERDEAVAKAERIDKELQEDRSRSRVGNGSFAFSQEFYEDMDLDELEPLSPEFNEDMDSEELEPFQVIKKNMERSHKKFIKDMECIKFIASERARVL

>AtHDA19

MDTGGNSLASGPDGVKRKVCYFYDPEVGNYYYGQGHPMKPHRIRMTHALLAHYGLLQHMQVLKPFPARDRDLCRFHADDYVSFLRSITPETQQDQIRQLKRFNVGEDCPVFDGLYSFCQTYAGGSVGGSVKLNHGLCDIAINWAGGLHHAKKCEASGFCYVNDIVLAILELLKQHERVLYVDIDIHHGDGVEEAFYATDRVMTVSFHKFGDYFPGTGHIQDIGYGSGKYYSLNVPLDDGIDDESYHLLFKPIMGKVMEIFRPGAVVLQCGADSLSGDRLGCFNLSIKGHAECVKFMRSFNVPLLLLGGGGYTIRNVARCWCYETGVALGVEVEDKMPEHEYYEYFGPDYTLHVAPSNMENKNSRQMLEEIRNDLLHNLSKLQHAPSVPFQERPPDTETPEVDEDQEDGDKRWDPDSDMDVDDDRKPIPSRVKREAVEPDTKDKDGLKGIMERGKGCEVEVDESGSTKVTGVNPVGVEEASVKMEEEGTNKGGAEQAFPPKT

>AtHDT1

MEFWGIEVKSGKPVTVTPEEGILIHVSQASLGECKNKKGEFVPLHVKVGNQNLVLGTLSTENIPQLFCDLVFDKEFELSHTWGKGSVYFVGYKTPNIEPQGYSEEEEEEEEEVPAGNAAKAVAKPKAKPAEVKPAVDDEEDESDSDGMDEDDSDGEDSEEEEPTPKKPASSKKRANETTPKAPVSAKKAKVAVTPQKTDEKKKGGKAANQSPKSASQVSCGSCKKTFNSGNALESHNKAKHAAAK

>AtHDT2

MEFWGVAVTPKNATKVTPEEDSLVHISQASLDCTVKSGESVVLSVTVGGAKLVIGTLSQDKFPQISFDLVFDKEFELSHSGTKANVHFIGYKSPNIEQDDFTSSDDEDVPEAVPAPAPTAVTANGNAGAAVVKADTKPKAKPAEVKPAEEKPESDEEDESDDEDESEEDDDSEKGMDVDEDDSDDDEEEDSEDEEEEETPKKPEPINKKRPNESVSKTPVSGKKAKPAAAPASTPQKTEEKKKGGHTATPHPAKKGGKSPVNANQSPKSGGQSSGGNNNKKPFNSGKQFGGSNNKGSNKGKGKGRA

>AtHDT3

MEFWGVEVKNGKPLHLDPGLDRLVHISQVALGESKNNVTEPIQLYVTVGSDKLLIGTLSHEKFPQLSTEIVLERNFALSHTWKNGSVFFSGYKVDASDPEPEDLIDDQLEAAGFKAAPKSAAKQVNFQLPNEDVKAKQDDDADGSEEDSSDDDDSENSGDEEEEKVTAESDSEEDDSSDDEEDDSSEEETPKKPEEPKKRSAEPNSSKNPASNKKAKFVTPQKTDSKKPHVHVATPHPSKQAGKNSGGGSTGETSKQQQTPKSAGAFGCKSCTRTFTSEMGLQSHTKAKHSAAA

>AtHDT4

MEFWGIEIKPGKPFKVIQKDGFMVHASQVTLGDVEKVKKDETFAVYVKIGDDENGFMIGNLSQKFPQFSIDLYLGHEFEISHNSTSSVYLIGYRTFDAFDELDEEIDSDSELDEYMEQQIAALPQNEINPEEDDESDSDEMGLDEDDDSSDEEDVEAEAPLKVAPPSKKMPNGAFEIAKGGKKNKSSGGKKRCPFPCGPSCKK

>AtSRT1

MSLGYAEKLSFIEDVGQVGMAEFFDPSHLLQCKIEELAKLIQKSKHLVVFTGAGISTSCGIPDFRGPKGIWTLQREGKDLPKASLPFHRAMPSMTHMALVELERAGILKFVISQNVDGLHLRSGIPREKLSELHGDSFMEMCPSCGAEYLRDFEVETIGLKETSRKCSVEKCGAKLKDTVLDWEDALPPKEIDPAEKHCKKADLVLCLGTSLQITPACNLPLKCLKGGGKIVIVNLQKTPKDKKANVVIHGLVDKVVAGVMESLNMKIPPYVRIDLFQIILTQSISGDQRFINWTLRVASVHGLTSQLPFIKSIEVSFSDNHNYKDAVLDKQPFLMKRRTARNETFDIFFKVNYSDGCDCVSTQLSLPFEFKISTEEHVEIIDKEAVLQSLREKAVEESSCGQSGVVERRVVSEPRSEAVVYATVTSLRTYHSQQSLLANGDLKWKLEGSGTSRKRSRTGKRKSKALAEETKA

>AtSRT2

MLSMNMRRVFGGVSTDLFPSRSMYRPLQSGGNLVMLFKGCRRFVRTTCRVSIPGGSLGNESKAPPRFLRDRKIVPDADPPNMEDIHKLYRLFEQSSRLTILTGAGVSTECGIPDYRSPNGAYSSGFKPITHQEFTRSSRARRRYWARSYAGWRRFTAAQPGPAHTALASLEKAGRINFMITQNVDRLHHRAGSDPLELHGTVYTVMCLECGFSFPRDLFQDQLKAINPKWAEAIESIDHGDPGSEKSFGMKQRPDGDIEIDEKFWEEGFHIPVCEKCKGVLKPDVIFFGDNIPKERATQAMEVAKQSDAFLVLGSSLMTMSAFRLCRAAHEAGAMTAIVNIGETRADDIVPLKINARVGEILHRVLDVGSLSVPAL

>OsHDA702 MDASAGGGGNSLPTAGADGAKRRVCYFYDAEVGNYYYGQGHPMKPHRIRMTHALLAHYGLLDQMQVLKPHPARDRDLCRFHADDYVAFLRSVTPETQQDQIRALKRFNVGEDCPVFDGLYSFCQTYAGGSVGGAVKLNHGHDIAINWAGGLHHAKKCEASGFCYVNDIVLAILELLKYHQRVLYVDIDIHHGDGVEEAFYTTDRVMTVSFHKFGDYFPGTGDIRDIGHSKGKYYSLNVPLDDGIDDESYQSLFKPIMGKVMEVFRPGAVVLQCGADSLSGDRLGCFNLSIRGHAECVRFMRSFNVPLLLLGGGGYTIRNVARCWCYETGVALGHELTDKMPPNEYFEYFGPDYTLHVAPSNMENKNTRQQLDDIRSRLLDNLSKLRHAPSVQFQERPPEAELPEQDEDQEDPDERHHADSDVEMDDVKPLDDSGRRSSIQNVRVKRESAETDAADQDGNRVAAENTKGTEPAADGVGSSKQTVPTDASAMAIDEPGSLKVEPDNSNKLQDQPSVHQKT

>OsHDA704

MASDMRSLNSQKGQSCGVSDQACHSKSKSGNDGKPSHAKANGVSSLSGSHNDEKILKENS

GACNLNSDHANPLSVDGTKVSTARSELIDSSGHDGCLHVKNESCMACDDLLQESDKEQPG

GTLEDLFSFNDEEDDDSDWEPSARLALSRWFCLNCTVPNMEGFTHCQNCDELKGSVVVGY

DAFKAHLAQAALLSADAALPSVSTAVGFDERMLLHSEIEIKPNPHPERPDRLRAIAASLA

AAGIFPSKCVMVPPREITKEELLRVHTSDHIDSVEQTKNMLYSYFTSDTYANGHSACAAK

LAAGICADLANLIVSGRVRNGFAMVRPPGHHAGVKQAMGFCLHNNAAVAALAAQRAGAKK

VLIVDWDVHHGNGTQEIFDGDNSVLYISLHRHEDGSFYPGTGAANEVGVMDGQGFSVNIP

WSRGGVGDNDYIFAFKHVVLPIAAEFAPDITIISAGFDAARGDPLGCCDVTPAGYSRMAS

MLTACSQGKLLVILEGGYNLRSISSSATEVVKVLLGDSPVYDTDATEPSEEGIQTVLQVL

SIQQQFWPVLVPSFASVLALQRSVFSRYTTEVNKMKRKHAGGAGPFWWKWGSKRLLYEVL

FEGRCLRKTKDTGKEKLNDEAEP

>OsHDA705

MAASGEGASLASAAGGEDGRRRRVSYFYEPSIGDYYYGQGHPMKPHRIRMAHSLVVHYGL

HRLLELSRPYPASDADIRRFHSDDYVAFLASATGNPALLDARAVKRFNVGEDCPVFDGLF

PFCQASAGGSIGAAVKLNRGDADITVNWAGGLHHAKKGEASGFCYVNDIVLAILELLKFH

RRVLYVDIDVHHGDGVEEAFFTTNRVMTCSFHKYGDFFPGTGHITDVGAGEGKHYALNVP

LSDGIDDDTFRDLFQCIIKKVMEVYQPDVVVLQCGADSLAGDRLGCFNLSVKGHADCLRY

LRSFNIPMMVLGGGGYTIRNVARCWCYETAVAVGVEPDNKLPYNDYYEYFGPDYNLHIQP

RSVENLNSTKDLENIKSMILDHLSKIEHVPSTQFHDRPSDPEAPEQEEEDMDKRPPQRSR

LWSGGAYESDTEDPDNMKTETNDLSASSVMKDESNDDS

>OsHDA706

MASSAPSAAAGATPPDPLRRDRILSSKLYLDVPGSKAPVVYSPAYDIAFLGIEKLHPFDS

SKWGRICKFLTKEGHLEKNRVVEPLEATKDDLLVVHSESYLNSLKSSLKVASIVELPPVA

FIPNWLVQQKLLYPFRKQVGGSILSAKLALERGWAINVGGGFHHCSAEQGGGFCAYADIS

LCIQFAFVRLNISRVMIIDLDAHQGNGHEKDFANDGRVYTLDMYNAGIYPYDHVAKRYID

QKVELVSGTKTEDYLDQLDKALKVAESRFQPQLIVYNAGTDILDGDPLGRLKISPQGVVI

RDEKVFRFAKDQSIPLLMLTSGGYMKSSARVIADSIINLSNKNLIELGSQLG

>OsHDA709

MATGGNSLPSPSCADDKKRRVCYYYDPGIAHIKFSDDHVMVPARVAMAHSLVGVYGMLGD

MRRLRTRPATEAEIRRFHSPEYVDLLRDLTPESYFNDAALRQKAEDDHGIGGDDDCPAFD

RLWKYCRGYAGGSFAAARALVDGASDIAINWSGGMHHASACKATGFCYVNDIVLAINELL

GTFRRVIYVDIDAHHGDGVQNAFLDSNRVMTLSFHRYGKITPHKDFFPGSGAINEIGDGA

GEHYSVNVPLDAGVRDDVYHTLFKPIVGKAMEVFQPEAIVLQCGADSLSGDRLGGMELSV

RGHAECVSFLRGFNLPLLLVGGGGYTINHVASAWCYETAVAVGKERKLPDDIEIPSHGYE

LMYKNQGNKLHYKTSTATAARKRSSSTEVTKGKVLEHLSQVKRAPSVQFQERRGGDNAAG

VGLYYERPPSLEDDEPAQRLHRLCFPGLTKRIRLND

>OsHDA710

MDPSSAGAGGNSLASASCGDAQKRRVCYFYDPEVGNYYYGQGHPMKPHRVRMTHALLAHY

GLLAPAKMQVLRPLPARDRDLCRFHSDDYVAFLRAVTPETQFDQIRSLRRFNVGEDCPVF

DGLYAYCQTYAGASVGAAVKLNHGTHDIAINWSGGLHHAKKSEASGFCYVNDIVLAILEL

LKLHERVLYIDIDIHHGDGVEEAFYTTNRVMTVSFHKFGDYFPGTGDIRDIGYSEGKYYC

LNVPLDDGIDDDSYQSIFKPIISKVMEMYRPGAVVLQCGADSLSGDRLGCFNLSGKGHAE

CVKFMRSFNVPLLLLGGGGYTIRNVARCWCYETGVALGEELREKLPYNEYYEYFGPEYSL

YVAASNMENRNTNKQLEEIKCNILDNLSKLQHAPSVQFEERIPETKLPEPDEDQDDPDER

HDPDSDMLLDDHKPMGHSARSLIHNIGVKREITETETKDQHGKRLTTEHKVPEPMADDLG

SSKQVPVSRRLLYPSANP

>OsHDA711

MLEKDRIAYFYDGDVGNVYFGPNHPMKPHRLCMTHHLVLSYDLHKKMEIYRPHKAYPTEL

AQFHSADYVEFLHRITPDTQHLYENELRRYNLGEDCPVFDNLFEFCQIYAGGTLDAARRL

NHKTCDIAINWAGGLHHAKKCEASGFCYINDLVLGILELLKYHARVLYIDIDVHHGDGVE

EAFYFTDRVMTVSFHKYGDFFFPGTGDIKDIGEREGKYYAINIPLKDGIDDSGFTRLFKT

VIAKVVETYLPGAIVLQCGADSLARDRLGCFNLSIEGHAECVKFVKKFNIPLLVTGGGGY

TKENVARCWAVETGVLLDTELPNEIPDNEYIKYFAPDYTLKVSNVNMDNLNSKSYLSSIK

VQVMESLRAIQHAPGVQMQEVPPDFYIPDIDEDELDPDERVDQHTQDKQIHRDDEYYEGD

NDNDHEDGAR

>OsHDA712

MESSASSSAAVAAEGAPATGEQLAVFWHEGMVAHDAGRGVFDSGRDPGFLDVLDQHPENA

DRVRNMVSILRRGPIARFISWHSGRPAHAAELLSFHSAEYIEELVQANAVGAKKLCEGTF

LNPGSWGAALLAAGTTLSAMKHILDGHAKIAYALVRPPGHHAQPDRADGYCFLNNAGLAV

KLALDSGRRKVAVVDIDVHYGNGTAEGFYHTDSVLTISLHMKHGSWGPSHPQSGSVDEIG

EGRGLGYNLNIPLPNGSGDAGYEYAMNELVVPAIEKFRPELLVFVVGQDSSTFDPNGRQC

LTMDGYRKIGQIMRGMADQHSNGQILIVQEGGYHISYSAYCLHATLEGVLNLQAPLLDDP

IAYYPEDEKYTMKVVDIMKKCWKESIPFLKDI

>OsHDA713

MAAATTAAAAARVGLLYDERMCAHATPDGEEHPENPERLRAIWRKLSADGVASRCMIMKA

KEAEDKYIASVHSQNHIKLMRSISSKEYDSRRNKIARKFNSIYFNKGSSESAFLAAGSVI

EVAEKVAAGELSSAIALVRPPGHHAEHNEAMGFCLFNNVAIAADYLLNERTDLGIKKILI

VDWDVHHGNGTQKMFYSDPRVLFFSVHRFDYGSFYPAEGDASYCFIGEGDGKGYNINVPW

EHGKCGDADYIAAWDHVLLPVAEAFNPDIVLVSAGFDAALGDPLGGCCITPNGYALLLTK

LLGFAQGRIVMALEGGYNLRSIANSVSACAKVLLGDKFRFDTPDMQPFESSWRVIQAVRD

ELKTFWPVLSNRLPENISLRSRPSQIELYSSGSDSEVEDLPDAIASVNIIQITYGIISES

LSKLNLDEDKIATKTTSSNVMVEGPTDSVEPQNDGSAAVSTEGISSLSSTWRSELSKVYV

WYASFGSNMWTPRFLCYIQGGKAEGMNIPCFGSHDTSPPRGSMWKTVPHRLFFGRSSTPC

WGTGGVAFLNPEINHTENSYVCMYKITLEQFNDVLFQENRLVKENGESGKTESPDSPLIG

LSEIEFVSRNKGVHLAPIKDSWYSNVLYLGEEDNLPILTMTCPSSDVERCRSGELPLCPP

SKTYSATLIRGLMEGKHLDADAAASYINTAATRGL

>OsHDA714

MEQLWVPSLPILGGRILPMLRHYCGFGSHHPLTWRSLQITGRKQKHNGCWIAYCLPSHNG

TSISDTNGVRKDLALPDNLLRDAHILYCTSPAMGHNKEAHPETNKRVPAIVDALEKLELT

SKHRGSQVLEIQDFQPASLDDIALVHSRSYITGLEKAMSRASDEGLIFIEGTGPTYATQT

TFQECLLSAGAGITLVDSVVAASKLGPKPPLGFALVRPPGHHAVPEGPMGFCVFGNIAVA

ARYAQNQHGLKRVMIIDFDVHHGNGTCDAFYEDPDIFFLSTHQLGSYPGTGKIHQVGQGN

GEGTTLNLPLPGGSGDYAMRCAFDEVIAPAAQRFKPDIILVSAGYDAHALDPLAGLQFTT

GTFYMLAARIREVAAELCGGRCVFFLEGGYNLESLSSSVADTFRAFLGEPSLAARFDDPA

MLYEEPTRKIREAIDKAKHLHSL

>OsSRT701

MSLGYAEKLSYREDVGNVGMPEIFDSPELLHKKIEELAVMVRESKHLVVFTGAGISTSSG

IPDFRGPKGVWTLQRSGKGVPGASLPFHRAVPTLTHMALVELEKTGRLKFVISQNVDSLH

LRSGLPREKLAELHGNSFKEICPSCKKEYLRDFEIETIGLKDTPRRCSDKNCGARLKDTV

LDWEDALPPEEMDAAKEQCQKADLVLCLGTSLQITPACNMPLLSLKNGGRVAIVNLQATP

KDKKASLVIHGLVDKVIAGVMYMMNLRIPPYIRTDFVQISLRNSVKKKCVRWTLRVTSIH

GLRAPLPFLRSVEVSFPERPDMKPVVLKEQPFSLQRETSMNRPFVMLLTFNFSDGCGCSS

SSIEWPVDFLKQKDSFVRDRSLVLQELQHAAEHRSRAGQHAILEREGVPRAETSIHALVT

NIVRYDTEDSKAAVPMATWMNSNGSLSKRHMDAIGCNPASSKKQKLVATRHRRKGLNPAT

QKV

>OsSRT702

MAAGAHASRASAPIIAGLTGALRAAYKGFSPQLCNFHASVNNGLLHRRKIQLHFICSFRS

IQARYNHSSAVAPKDYCETYIQFLRDKQIVPDSDPPSAKDVDLLYRFIDQSKKLMVLTGA

GMSTESGIPDYRSPNGAYSSGFKPLTHQEFVRSIRARRRYWARSYAGWRRFRRAQPNSAH

YALASLERIGRVHSMVTQNVDRLHHRAGSKPVELHGSVYEVACLDCGTSIDRESFQEQVK

DLNPKWALAIDSLEVGQPGSDKSFGMQQRPDGDIEIDEKFWEQDFDIPSCNQCGGVLKPD

VVMFGDNVPEERAESTKEAARNCDALLVVGSALMTMSAFRLARLAHEANAPIAAITIGET

RADSILSLKINARCGEILPRILQMGSLAVPNVS

>LcHDMA1

MDPATEPPLEPSDNPIDDAVYDDDSSPETDATLSPVPPEPEDPQTLPETNLTQSPTRNTTLDAPVSDSLDDSSDPITEDQQPQNPNPAEPGPPPRKRRRRKRFFTEINGTPSFAKNRRPRLSCLSKEVDTEALIAISVGFPVDSLTEEEIEANVVAKIGGTEQANYIVVRNHILARWRSNVTVWLTREHALESIRSEHKNLVDSAYNFLLEHGYINFGLAPAIKEAKLTSLDGMERANVVIVGAGLAGLVAAKQLVSMGFKVAVLEGRARPGGRVKTLKMKGEGVVAAADLGGSVLTGINGNPLGVLARQLGLPLHKVRDICPLYLPDGRAIDTDIDSRIEVLFNKLLDRVCKLRHDMIEEFKAVDVPLGSALEAFRNVYKVAEDQQERMLLDWHLANLEYANASLMSNLSMAYWDQDDPYEMGGDHCFIPGGNERFVRALAEDLPIFYQRTVESIRYGVDGVMVYAGGQEFRGDMVLCTVPLGVLKRGYIEFVPELPQRKKDAIQRLGYGLLNKVAMLFPYNFWGGEIDTFGHLTEDSTTRGEFFLFYSYSSVSGGPLLVALVAGDAAVKFETMSPVDSVKRVLQILRGIYHPKGIVVPDPVQAVCTRWGKDRFTHGSYSYVAVGSSGDDYDILAESVGDGRVFFAGEATNKQYPATMHGAFLSGMREAANIMRVSKRRSIALTNKAYNDSEENCNLDKLFDTPDLTFGRISALFDPGSNDLESHALLRVKFQGKKLDSDCLSLYGLVSRKQAIQLSELDGDENRMKMLHYNFGVRLVATRGLCNAGESLITSIKAAKLNLN

>LcHDMA2

METPGSDGLVSRRTLRKKVGQRNYDENLMDELIDKHLGGSFKKRSKKKEDLEKETETEAMIAFSVGFPIDALREDEIKAGVVRALGGKEQNDYIVVRNHILARWRSNVRIWLTKGQIRETVSSEYEHLINSAYDFLLYNGYINFGVSPSFVAYMPEEVNEGSVIIVGAGLAGLAAARQLLSFGFRVVVLEGRSRPGGRVYTQKMGEKGKIAAVDLGGSVITGIHANPLGVLARQLSIPLHKVRDNCPLYKPNGAPVDKEMDANIEFIFNKLLDKVMELRQIVGGFANDVSLGSVLDKLRQLYVVARNPEERKLLDWHLANLEYANAGCLSDLSAVYWDQDDPYEMGGDHCFLAGGNGRLIKALCEGVPIFYNQTVNTIRYGNEGVEVIAGNQRFQADVVLCTVPLGVLKKKKMNFEPELPQRKLAAIERLGFGLLNKVAMIFPYVFWGEDLDTFGCLNEQSNKRGEFFLFYSYHTVSGGPALIALVAGEAAKMFECTDPTFLLHRVLNVLGGIYNPKGIDVPNPIQSICTRWGSDPFTYGSYSHVRVQSSGSDYDILAESVGGRLFFAGEATTKQYPATMHGAYLSGLREASCILQATRGLKNNWRRSVLRNVGSSNDILTDLFRRPDLAFGKFMVVFNPLMEDSKSMGLMRVTFGSDDDGKKVLENNYKHPSNLPLQLYTLLSREQAHQLQQLTGGNECKLSYLTNNLGLKLMGSAALGTLASSLISNIANARRSGRGRNRISAIQQITI

>SlHDMA1

MDPSDNNPSSQQSLPYITSNNPLQFTIHLPNSTPNFTSIPNSNPSRDPNPNPNPNLNPNPHSDSISDQLLSLSIPTKRRRGRPRSTATSSLDQFSKTLVENSNLVRNLSARKNTASFDASDEIIVINKEATTEALIALTAGFPADSLTDEEIEAGVVSVVGGIEQVNYILIRNHILMKWRVNVWTWITKEMFYDVIPKHCYALLDSAHNYLVSRGYINFGVVPAIKDRIPAEPSKPSVIIIGAGLAGLAAARQLMLFGFKVTVLEGRKRAGGRVYSKKMEGGNKVAAADLGGSVLTGTLGNPLGILARQLSCTLHKVRDKCPLYRVDGKPVDQDLDHKVETAYNLLLEKASKLRQLMGEVSQDVSLGAALETFRQDYEDAVNEEEMSLFNWHLANLEYANAGLISKLSLAFWDQDDPFDMGGDHCFLPGGNGKLVHALSENVPILYEKIVHTIRYGTDGVQVGAGAQVFEGDMVLCTVPLGVLKGGSIKFMPELPQRKLDGIKRLGFGLLNKVAMLFPYVFWGTDLDTFGHLTDNSSSRGEFFLFYSYATVAGGPLLLALVAGEAAHKFETMPPTDAVTKVLQILKGIYEPQGIEVPEPIQTVCTRWGSDPFSLGSYSNVAVGSSGDDYDILAESVGDGRLFFAGEATNRRYPATMHGAFLSGLREAANIVHHAKARTMSLKIEKKPSKSTHYYASVLDDLFREPDLEFGSFSIIFARKSSDLESPAILRVTFCGPQTRNHDGIRPGRHLSNKLLFQQLQSQFNNQHELHVYTLLSKQQALDLREVRGGNEMRLNFLSEKLGVKLVGRKGLGPSVDSIIASVKAERGRRKPGTLKTGVMKSKDTTLRRKIVRKAKVVSGGNRTTSFPASSSRIKAVGSSTTTIPLTNLDLEPKPVCAIGSAASPSLNVRVNDDMESKSVGSSVHLLHNASIGDKFEGNFGSSTAPLLNVGGNTGSNSDGPMYPRNTYDDSTDTCVPPITGNLASQHTSGDGDMESMMLDGECRM

>SlHDMA2

MNRRGGRSESKQCLNHVSSDFVGNTNEERSALVATSLSGKISKTRGNSLNFAHRNVHSRMVKSRISKKKLVADEIIFINNESYSEALVAITAGFPSDSLKDEEIEAGVVSEDGGTEVYNYILLRNHIITKWRGNVSIWLTKDMFVDVIPEQYSGLLDSAYNYLLSYGYVNFGVTLAIKDKIPTRPSKGRVIVIGAGLAGLAAARQLMLFGFEVIVLEGRKRAGGRVYTKKMEGGNKIAAADLGGSVLTSTLGNPLGVLARQLSYTLHTVRDRCPLYHADGTPVDEYLDKKVEVAYNELLEKASKVRQDLSPIISLGEALETLRKDSSVAMNDEEMNLFNWHLANLEYANASLLSKLSLRFWDQDDPYDMGGDHCFLPGGNGRLIDALAENVPISFEKTVHTIRYGRDRVKVITAGQVFEGDMVLCTVPLGVLKRGSISFFPELPQRKLDTIRRLGFGLLNKVALLFPYVFWDSNVDTFGHVADDSKHRGEFFLFYNYATVSGGPLLLALVAGKAAHRFERMTPTDAVTKVLQILKGIYEPQGINVPKPIQIVFTSWGSDPFSYGSYSSVAVGASGDDYDILAETVGDGRLFFAGEATTRHYPATMHGAFLTGLREAAKMAHHASVRTSHLQAEKK

>SlHDMA3

METPAGHPSETSHTVNSDENLRQDNHHNANSSPPQTTPEAAVSDSQLDDLPKSSETKISSDLPPPTGVTPPQVKRRRRRKRFFTDLIPPSVSAAAVNGLRVLRPNPKPSTAYSYSETELATGDDPSKNHNRRRRMSDLAKEVDVEALIAISVGFPVDSLTEEEIEANVVSQIGGIEQANYIVVRNHILARWRSNVTVWLTKEHALESIRAEHKNLVHSAFSFLLHHGYINFGVAPAIKEVKFKPPEGASKGNVIVIGAGLSGLVAARQLISLGLKVVVLEGRGRPGGRVRSKKMTGRQNGVVAAADLGGSVLTGINGNPLGVLARQLGVPLHKVYYVLKQLSETPMLTPGNEFYGTNVQFNFTSSYNLFLFYLSLKNSDSYQSS

>SlHDMA4

MENQNSGGLISERPVRKRIASRNYDENLMDNFIDEQLGGPVGKKIRTKKDLEKETEKEALIALSLGFPIDDLLEEEKKAGVVSELDGKEQNDYIVVRNHILVKWRENVHIWLNKGRIRETVSVEYEHLVAIAYDFLLSNGYINFGVSPSFVSNLPEEPREGSVIIVGAGLAGLAAARQLMSFGFKVSILEGRNRPGGRVYTEKMGWKGKFAAVDLGGSVITGIHANPLGVLARQLSIPLHKVRDKCPLYKPDGAPVDAVVDSRVELIFNKLLDKVTELRKIVSGLANDVSLGSVLEKLRRIYTVAKTKEEKQLLHWHFANLEYANAGCLSELSAAYWDQDDPYEMDGDHCFLAGGNWGMIRALCKGVPIFYGKTVETIKYGNEGVEVIAGDQLFQADMVLCTVPLGVLKRRLIRFEPELPEKKLEAIDRLGFGLLNKVAMVFPHVFWGEDLDTFGCLNNYSHRRGEYFLFYSYHTVSGGPVLIALVAGDAAQLFESTEPSTLVNRVMNILKGIYEPKGISVPDPIQSICTKWGSDPFSFGSYSHVRVQSSGSDYDILAENLGGRLFFAGEATIRQHPATMHGAYLSGLREASHIYRSMKARLNNPRKTVSKNVRPSNDVLEELFKNPDLAFGKFLFVFDPLTCDSKSLGLMRVTFGKSNNEFNTEEADNMPQHLLNPSLQLYTVVSREQARELQLVKEGNHCKLSDLLKGLGLKLVGANGLGVQGHSLSAKIVNARKGRKRSRSCTAKHKAGNTTPEVKQMSFHGNIELPDWASNITSVLWIPVRATCDELLKGAPNLVEGLRTIALKLEFGVSQNQIFERTIAVHFTDPFSVSIRVTDKSTDKKLLLQVILQSQVQATLTIYDSWLDLQDDFAPTRNGDKKPISGFFPLVISPKSRAGILFSVCLASAPIEEET

>SlHDMA5

MEIPNSGGSVSRRPVRRRFASRNYDETVMDKIIDEQLGSPVGKKIRTKKDLEKETEKEALIALSLGFPIDDLLEEEKKAGVVSELDGKEQNDYIVVRNHILAKWRENVHIWLNKGRIREIISVEYEHLVAIAYDFLLSNGYINFGVSSSFESNLPEEPREGSVIIIGAGLAGLAAARQLMAFGFKVTVLEGRNRPGGRVYSEKMGWKGKFAAVDLGGSVITGIHANPLGVLARQLSIPLHSVRDKCPLYKPDGAPVDSVVDSTVELIFNKLLDKVAELRKIVSGLATDVSLGSVLETLRRLYCVAKTKEEKQLLHWHFANLEYANAGCLSELSAAYWDQDDPYEMDGDHCFLVGGNRAMIRALCKGVPIFYGKTVQTIKYGNEGVEVIAGDQLFQADMVLCTVPLGVLKRRSIRFEPELPEKKLEAIDRLGFGLLNKVAMVFPHIFWGEDLDTFGCLNHHSHRRGEYFLFYSYHTVSGGPVLIALVAGDAAQLFESTDPSTLINRVINILKGIYEQKGISVPDPIQSICTKWGSDPFSFGSYSHVRVQSSGSDYDILAENLGGRLFFAGEATIRQHPATMHGAYLSGLREASHISQSMKARQNNPRRTVSKNVRPSNDTLEELFEKPDLAFGKLLFVFDPLTCDSNSLGLMRVTFGKSNDELNSEEVDNMPQHLLNPSLQLYTVVSREQAHELQLVKEGNNCKLLDLLEGLGLKLVGANGLGVQGHTLAAKIVKGRRSRSYTAKQKAGESSSQLP

>SlHDMA6

MGEEENVELRLERGTKKRIEMKFDSGDDEPIGSLLKLKSKKQSKKAKVDLGGSKDVVQKTAVKDEHLVGMDDTLASFRKKLRGPKKDSGSVSTIGKSSSSNASKLTVESPDGSVKAVAKIVENGLSDVECLSEGIIDKGFEKGNKRKGKRPKVSSELKKVEISEDMSLQNDKESGKSPPNCMDGILEDSLSAFLKKAQSGMFKKSHSSLQLKRGKESEVLCDVLNSCPTATEIFPSISKNMCQKLVEGMPESNENVHVALDRGSVDMHLSENKEFVQLIQLQSDSRPELLSSALNNVELLKSSIAIDDTSSIEGSQLDLPACFNKIAGVVDGEVKCHSKLSEEETATTYNIVRGNCKDLHDEDVLKNCSIYYEQSASKDGFSDRPMATGRDVLSAGIITPENVEMLEHPVIETKFNADMDVNAILSSRETHVDDQMCSSNRVDDSGSCRSVQLLNKLDHTSEGSTCNVFSRTLISSTFRLEGLTAAKEKTDMEGSGYAQVRLAPDFIAVEKCSSDFDDQQRISGDSVNEQACSPVSLPKEDGQVFAGGLSLVSIGRSQQVNASQMKQEDQIMENDDDLYDSSKQLTIDNAATSLRKCSLVFHQSELADENCEGAHHQSRVFVSGDDEADDTSSPSITPECDESVAEETEAKLAAEEKEQRIFSGQRASRKTKKRRHGDMAYEGDVDWDVLVHGQDLFSSHQDGEGRHAFKTREKLESSLTVMDTENGGIAAVSVGLKAREVGPVERIKFKELLKRRVGLLEFLECRNQILSLWNKDISRVLPLSECGVSETPLADESPRASLIRQIYSFLDQCGYINFGIASEKDKAENGAEHSLKILKEEKTIEKSGASVADRDDGVSFILGRSKSSEIIMPEKNDVLSDEGKKTEKCGADCQLIDRRAIELSTLAEPRECPIDDCRVNGYLDIQSPRQPFDLGLVAQVPSSEVKDSELQNIVDPGLLPPNNTEIDGRAADKHIVISEDSCGFTSDSLGCQSLNTCCDAKGKKEIIVVGAGPAGLTAARHLKRQGFHVTVLEARSRIGGRVFTDRSSLSVPVDLGASIITGIEADVATERRPDPSSLICAQLGLELTVLNSDCPLYDVATGQKVPVDLDEALEAEFNSLLDDMVLLVAQKGEHAMRMSLEDGLEYALKKRQKARFARNHMGNESQKLSVTAVESMALSDVGVPQNNNSKVEILSPPERRVMDWHFANLEYGCAALLKEVSLPYWNQDDTYGGFGGAHCMIKGGYSSVVEALGEELCVHLNHIVTDISYCKKDVLSNNDLFNKVKVSTTNGREFSGDAVLITVPLGCLKAETIKFSPPLPQWKDLSIQRLGFGVLNKVVLEFPEVFWDDSIDYFGATAEDTDERGRCFMFWNVKKTVGAPVLIALVVGKAAIDGQEMSSDDHVKHSLLVLRKLYGEKMVPDPVASVVTNWGKDPYSYGAYSYVAVGSSGEDYDILGRPVENCLFFAGEATCKEHPDTVGGAMMSGLREAVRIIDILTTGTDYTAEVEAMEDAMRHSDVERSEIRDIIKRLEAVELSSVLCKESLDGVKIVTRENLLRDMFCKANTTAGRLHLAKELLKLPVEVLRSFAGTKEGLSTLNLWMLDSMGKDGTQLLRHCVRLLVLVSTDLLAVRLSGIGKTVKEKVCVHTSRDIRAVASQLVNVWIELFRKEKAANGGLKLLRQSTATDTSKSKHIAAPGKPPIRSHPSAVDSKRSSKVSSSAGNHLAVSVNNKKLNVRPATIGAIPVVEPSTSQASVGRQNDTSEETQNFPMSEEEKAAFAAAEAARLAALAAAEAYASSGAKCNMPLQLPKIPSFHKFARREQYANMDESDIRKNWPGGVVGRQDCLSEIDSRNCRVRDWSVDFSAAGVNLDSSKMSVDNRSQRSLSNDNASQLNYKEHSAECAPVDSSIFTKAWVDSSNSVGIKDYNAIEMWQCQAAAANSDFYDPVMHVTDEEDSNVSSKMDMRKHDVLVCESSASQITVNKEALDNQPRGAKRIKQAVVDYVASLLMPLYKARKLDRDGYKSIMKKTATKVMEHATDAKKAMHVYEFLDFKRKNKIRDFVDKLVERHIQMNPGAKS

>AtHDMA1/FLD

MVSFSAPKKRRRGRSQRSMSSLNSLPVPNVGLLPGNSNFVSSSASSSGRFNVEVVNGSNQTVKSYPGIGDEIITINKEATTEALLALTAGFPADSLTEEEIEFGVVPIVGGIEQVNYILIRNHIISKWRENISSWVTKEMFLNSIPKHCSSLLDSAYNYLVTHGYINFGIAQAIKDKFPAQSSKSSVIIVGAGLSGLAAARQLMRFGFKVTVLEGRKRPGGRVYTKKMEANRVGAAADLGGSVLTGTLGNPLGIIARQLGSSLYKVRDKCPLYRVDGKPVDPDVDIKVEVAFNQLLDKASKLRQLMGDVSMDVSLGAALETFRQVSGNDVATEEMGLFNWHLANLEYANAGLVSKLSLAFWDQDDPYDMGGDHCFLPGGNGRLVQALAENVPILYEKTVQTIRYGSNGVKVTAGNQVYEGDMVLCTVPLGVLKNGSIKFVPELPQRKLDCIKRLGFGLLNKVAMLFPYVFWSTDLDTFGHLTEDPNYRGEFFLFYSYAPVAGGALLIALVAGEAAHKFETMPPTDAVTRVLHILRGIYEPQGINVPDPLQTVCTRWGGDPFSLGSYSNVAVGASGDDYDILAESVGDGRLFFAGEATTRRYPATMHGAFVTGLREAANMAQSAKARGIRKRIDRNPSRNAHSCAILLADLFRDPDLEFGSFCIIFSRRNPDPKSPAILRVTLSEPRKRNEDPKADQHSNKILFQQLQSHFNQQQQIQVYTLLTRQQALDLREVRGGDEKRLYYLCETLGVKLVGRKGLGVGADSVIASIKAERTGRKLPSSSTSGTKSGILKAKSGALKRKMIRRIKGPLPLKQSNNNGVSESIKSESLGNGKSLEQQQQPRIVESIGGGSSMQGESKSLSTGVASSSGGSLHVGGETMKKIEQ

>AtHDMA2/LDL2

MNSPASDETAPRRNRRKVSRKNYDENAMDELIEKQLGGKAKKKYRTKQDLEKETETEALIALSVGFPIDELLEEEIRAGVVRELGGKEQNDYIVVRNHIVARWRGNVGIWLLKDQIRETVSSDFEHLISAAYDFLLFNGYINFGVSPLFAPYIPEEGTEGSVIVVGAGLAGLAAARQLLSFGFKVLVLEGRSRPGGRVYTQKMGGKDRFAAVELGGSVITGLHANPLGVLARQLSIPLHKVRDNCPLYNSEGVLVDKVADSNVEFGFNKLLDKVTEVREMMEGAAKKISLGEVLETLRVLYGVAKDSEERKLFDWHLANLEYANAGCLSNLSAAYWDQDDPYEMGGDHCFLAGGNWRLINALAEGLPIIYGKSVDTIKYGDGGVEVISGSQIFQADMILCTVPLGVLKKRSIKFEPELPRRKQAAIDRLGFGLLNKVAMLFPSVFWGDELDTFGCLNESSINRGEFFLFYAYHTVSGGPALVALVAGEAAQRFECTEPSVLLHRVLKKLRGIYGPKGVVVPDPIQTVCTRWGSDPLSYGSYSHVRVGSSGVDYDILAESVSNRLFFAGEATTRQHPATMHGAYLSGLREASKILHVANYLRSNLKKPVQRYSGVNINVLEDMFKRPDIAIGKLSFVFNPLTDDPKSFGLVRVCFDNFEEDPTNRLQLYTILSREQANKIKELDENSNESKLSCLMNTLGLKLMGANSVLDTGGALISVIANARRGRSRSHVVAGQCNLPLNPLHFN

>AtHDMA3/LDL1

MSTETKETRPETKPEDLGTHTTVDVPGEEPLGELIADDVNEVVSDASATETDFSLSPSQSEQNIEEDGQNSLDDQSPLTELQPLPLPPPLPVEARISESLGEEESSDLVTEQQSQNPNAAEPGPRARKRRRRKRFFTEINANPAFSRNRRTSVGKEVDSEALIAMSVGFPVYSLTEEEIEANVVSIIGGKDQANYIVVRNHIIALWRSNVSNWLTRDHALESIRAEHKTLVDTAYNFLLEHGYINFGLAPVIKEAKLRSFDGVEPPNVVVVGAGLAGLVAARQLLSMGFRVLVLEGRDRPGGRVKTRKMKGGDGVEAMADVGGSVLTGINGNPLGVLARQLGLPLHKVRDICPLYLPNGELADASVDSKIEASFNKLLDRVCKLRQSMIEENKSVDVPLGEALETFRLVYGVAEDQQERMLLDWHLANLEYANATLLGNLSMAYWDQDDPYEMGGDHCFIPGGNEIFVHALAENLPIFYGSTVESIRYGSNGVLVYTGNKEFHCDMALCTVPLGVLKKGSIEFYPELPHKKKEAIQRLGFGLLNKVAMLFPCNFWGEEIDTFGRLTEDPSTRGEFFLFYSYSSVSGGPLLVALVAGDAAERFETLSPTDSVKRVLQILRGIYHPKGIVVPDPVQALCSRWGQDKFSYGSYSYVAVGSSGDDYDILAESVGDGRVFFAGEATNRQYPATMHGAFLSGMREAANILRVARRRASSSALNPNQICIDKEEEVDEEEDRCLDQLFETPDLTFGNFSVLFTPNSDEPESMSLLRVRIQMEKPESGLWLYGLVTRKQAIELGEMDGDELRNEYLREKLGLVPVERKSLSQEGESMISSLKAARLNRQIFD

>AtHDMA4/LDL3

MDGKEKKSGSKRGSKVFQFDDDADDDEPIGSLLEIMKHKSSKKDKVETESTGKQRQKQVVEKKLSALGKDSEDMDDTLASFRKRLKGNKKGVESGTSRVRNHEGVDTVTNSNLKPIEEANKNEVQSVLLRENGASNSIQKCASETGTLLHKFSGKDKAASPSHEKVETVSSEKEADVFHQITKEESEIPMSEKAVELSRVSVPMPDVHGEVNCTIAPDKHIHLGEPTSESGYYREKNLVMCDCGTQFNFEDRSFESNTQVTLCQKCKYSSHHNASNGGGIQVNTLEDGTAQASPVSIIPCEDENFRGDAISLPNSGKPSTLQRPERIAKKRKLGNMVYEGDVKWENEQGFLDCQSDKSFKGSDKCGFVPSISKEIEIGRAAAVTAGLKAQSVSPIEKIILKEVLKRKGSNQEYLVCRNSILGLWSKNVSRILPVTECGVTGGPSESELPSASLIREVYKFLDQRGYINAGISSVNGKAASSTNQDYDLLQGRQLEESSMASVADSEEGVAFILGQVKAVESTSEGKKCALQNDERDLVGCATSEMLESISKKCEASIIDDNKRSVSMNALQDSTASNVEKHPETFSVAKPALSSTLSSAHSNQMRGRDCVPCEVIDEKKVIVIGAGPAGLTAARHLQRQGFSVTVLEARSRVGGRVFTDRSSLSVPVDLGASIITGIEADVPSERMPDPSVLVCNQLGLELSVLHGFCPLYDTVTGKKVPAELDDALQAEFNSLIDDVDLLVEEIGKERANKMSLEDGLEYGLQRLRMPHDKVNIDKFGLLNSSSKTGIRGPFMQDESWKDDFLNPLERRVMNWHFAHTEYGCAAVLKEVSLPHWNQDEFYGGFGGPHAMIKGGYSRVVESLAEGLDIHLNKIVSDVSYVSDVSAMDNSKHKVRVSTSNGCEYLGDAVLVTVPLGCLKAETIKFSPPLPDWKYASIKQLGFGVLNKVVLEFPTVFWDDSVDYFGATAEETDLRGECFMFWNVKKTVGAPVLIALVVGKAAFEYTNKSKSEHVNHAMMVLRKLFGGDLVPDPVASVVTDWGTDPYSYGAYSYVAIGASGEDYDVLGRPVQNCLFFAGEATCKEHPDTVGGAMMTGVREAVRIIDILRSGNDYTAEIETLEKAQRKSVPVRDEVRDLIKRLEVVELSNVLARQSLLRNMFFSAKTTVGRLHLAKELLNLPGETLKSFAGTKEGLAVLNSWILDSMGKNGTQLLRHCVHILVRVTSDLFALRLSGIGKTVKEKVCAHTSRDIRAIASQLVNVWLDLYRKEKANSGKKSLRQANTTNTSRIRRKLNSPDTDSKGKLSNGNDVKTDEEFEDNQLPMSEEEKAVFAEAEAARAAAEAAAKAFSEAYHNTSLQLPKIPSFHKFARREQYAKMDESDFRKKFPGNVLGRQDCMSEIDSRNCKVRDWYDFPASCLDLDSARIPVDNYSQPSHSNELVSHSKFRECSGESVAADTSFLTGAWVDTGGSSDGFKDSQAIDRWQSQAAAADPEFFNRTLHIKDEEDSIACSTGPPSWKHDQRANECSVSQVTVNKEPHKNHIRSADRLKQGVVDFVASLLMAPYRAKKIDRDVYKSIMKKTATKVMQHTTDVEKAMAVTQFLDSKRKNKIRDFVDKQVDKYMVIPQVPKP

>OsHDMA701

MSDQPPPYTPLPLLSSFPPNPYPDQTPDPASTPTLVLPNPAFPNKRKRTGFRRKLPSGSP

AAPVAVAASPSAQPPPRASAADDIIVINREPTAEAVTALTAGFPADSLTDEEIEAGVVSD

VGGIEQVNYILIRNHLLTRWRETFNSWLAKESFATLIPPHCDHLLNAAYSFLVSHGHINF

GVAPAIKERIPKEPTRHNTVIVVGAGLAGLAAARQLVAFGFKVVVLEGRKRCGGRVYTKK

MEGGGRSAAGDLGGSVLTGTFGNPLGIVAKQLGLPMHKIRDKCPLYRPDGSPVDPEVDKK

VEGTFNKLLDKSSLLRASMGDVAMDVSLGAALETLRQTDGDLSTDQEMNLFNWHLANLEY

ANAGLLSKLSLAFWDQDDPYDMGGDHCFLPGGNGRLVQALAENVPIVYERTVHTIRYGGD

GVQVVVNGGQVYEGDMALCTVPLGVLKNGGVKFVPELPQRKLDSIKRLGFGLLNKVAMLF

PHVFWSTDLDTFGHLTEDPSHRGEFFLFYSYATVAGGPLLMALVAGEAAHNFETTPPTDA

VSSVLKILRGIYEPQGIEVPDPLQSVCTRWGTDSFSLGSYSHVAVGASGDDYDILAESVG

DGRLFFAGEATTRRYPATMHGAFISGLREAANITLHANARAAKSKVEKGPSTNTQACAAL

LMDLFRQPDLEFGSFSVIFGGQASDPKSPAILKVELGGPRKKGATEGGKADQHHSNKLLF

QQLQSHFNQQQQLYVYTLLSRQQAMELREVRGGDEMRLHYLCEKLGVKLVGRKGLGPGAD

AVIASIKAERNSSRTKTRPSKLKIGIPKSKS

>OsHDMA702

MEEGSEAQPPLQPEAVSAEASEPPPPVPMDQDEGQAAAAEAMEGEAEGAAAAAGTIEGEA

GYAAADADPMEDEAADEAGAAEPMEDDPPTSSAPSATAAVDDSTIARKRRRRKKQFPGMI

PTAGVRVLRAAASAPSAAHLNGVPRRRGRPPTSSSLRLARELDAEALIALAAGFPADSLS

EDEVAAAVLPRIGGVDQTNYLVVRNHVLALWRSNPLSPVASNAALASIRAEHAHLVAAAH

SFLSDHAYINFGLAPSVISLPPCPPPSLPPPSVLIVGAGFAGLAAARHLMSLGFKVAIVE

GRLRPGGRVFTKSMRSTAAEYPDIAAAADLGGSVLTGINGNPLGVIARQLGFPLHKVRDK

CPLYLPDGRPVDPDMDARVEAAFNQLLDKVCQLRQVVADSIPHGVDVSLGMALEAFRAAH

GVAAEREERMLLDWHLANLEYANAAPLVDLSMAFWDQDDPYEMGGDHCFIPGGNSRFVRA

LADGIPIFYGQNVRRIQYGCDGAMVYTDKQTFRGDMVLCTVPLGVLKKGNIQFVPELPAQ

KREAIERLGFGLLNKVVLLFPYDFWDGRIDTFGHLTEDSGQRGEFFLFYSYSSVSGGPLL

IALVAGESAIEFEKTSPAENVEKVLETLRKIFSPKGIEVPKPLQAICTRWGTDKFTYGSY

SYVAIGSSGDDYDILAESVCDRVFFAGEATNRRYPATMHGALLSGYREAANIVRAARRRA

KKVDSPKKMDVNNEVKYEVKVDNIDLDDLFRTPDAAFGGFSVLHDPSTSEPDSISLLRVG

IGARKLGSGSLFLYGLIMRKNVANLAAMEGDEQRLSTLYRDFGTKLVGLDGLGDSGSSLI

SRIKAAARK

>OsHDMA703

MSSSSRRPARRAALTARSSYDESLVDAELESYLGNARSRRISRLRRLSADERQRETETEA

LIALSLGFPIDELLPAERPLLPAPVAAAPNDYIVVRNHILASWRADPRVPLPRSRVQETV

AASYDNLVAVAHGFLAREGHINFGVSAAFPASPPPDAPQRLAASVLVVGAGLAGLAAARQ

LLRFGLRVLVLEGRARPGGRVYTTHLGGDQAAVELGGSVITGIHTNPLGVLARQLGIPLH

KVRDSCPLYHHDGRTVDMKLDRSMDLVFNTLLEHATRLREYLKKAAEGISLGEGIERLRR

FYKVAKSVEEREVLDWHLANLEFSNAGCLSELSLAHWDQDDQYEMGGDHCFLAGGNARLV

HALCDGVPVLYEKTVKRIEHGEDGVSITVEGGQVFKADMALCTAPLGVLKSRSIIFEPEL

PERKLEAIQRLGFGLLNKVAMVFPHVFWDEEIDTFGCLNKERSKRGEFFLFYSYHTVSGG

AVLIALVAGEAALEFEKVDPAVALHRVLGILKGIYGPKGVTVPDPIQSCCTRWGSDPLCS

GSYSHIRVGSSGTDYDILAESVNDRLFFAGEATNRAYPATMHGALLSGLREASKILHASE

SRLNSDYKKYALQKSIRLINNVLDDLFMEPDLECGRFSFVFSYITPEEEQAPGLARITLE

KPLLLPSKKRKVKGNQKDQDPVAEKIDQEVFYLYATVSQEQATELLECDNDKSRIAVLCK

DLGVKLMGYDSTYDVCSHLISSISRAQKARKRLQGPKSLKTGL

>OsHDMA704

MPDIRSAPGRGKSDEDERRPIGSLFKLKRKRRAPGSAEAKGDSNPSVESEAPDGVVPGEMDDTLAIIKRKLRKPKKGKEGGDAVVVGSGAEGELLVEEEDVQGGVNVGDGVAEDKSNLEGVKVEVDEVIGGELKDSGGLGLEDSLSTLFKRSGRKSRQVSVKEEEGVEVAGSHGEEILEKGSGLVSDRVAKGTKRRRRRTKEEMKNAAAKSESAMAHEGSPNRKVGTSLPRKAKAEAKVKISNSNRRSKKSDEKPKASDDVLCHRSLGETIEQDAETRTVLDDGSRNSSDGASHRIEVSACLSNQPCLKPCSGELAEEVSLSAANAATDGVSNEHTYSETLLKERNDDAGCSHGKPPTLAIKSIPGKKPIEMPKKPVRQKDQLLSTDVDNKCVVGSGDTKDVNIENQPAFGIPESHVTGKGLHPHKMATSVKELDVVDVVAPTDFEDMENASKSKRVTRSSRKRKHGDMAYEGDIDWETLMQEQGLFSNLSAALVDYPLKSKDKIKISEVLDNGDGSGVAAVRAGLKAKAVTPIEKIKFKDILKRRGGLQEYLECRNMILSRWCKDVKHILDLAECGVSDVCLDDESPRQTLIRDVYLFLDQNGYINAGIASDKVKTDHESPPEDVEVSKLNESHERKSVSIQDCIVTEAVQDKKAVVKQTDCVLTEASNEESSSAAIHCDAQDLLPPLKSEELIFKEKNQGVLTEGRDESALPSNSDIHSKSDLDGFILKVEGGSLHQAEAADIEHSENKHEASDRVESGGYGKKIIVVGAGPAGLTAARHLQRQGFSVTVLEARNRIGGRVYTDRVSLSVPVDLGASIITGVEADIATERRADPSSLICSQLGLELTVLNSACPLYDVVTGDKVPDDLDTDLESEYNGLLDEMAQLFAQNGESAVGLSLEDGLEYALRKNRVTRSEQDDQLRNVSSAGAVDISESASTEKEIAHCGKEDKTDVLSPLERRVMNWHFAHLEYGCAAMLKSVSLPYWNQDDVYGGFGGAHCMIKGGYDTVLESLAKGLDVQLNHVVTEVLYGSEELGASGNSRKFVKISTSNGNEFVGDAVLITVPLGCLKAQTIKFSPSLPDWKLSSIDRLGFGLLNKIVLEFPEVFWDDNVDYFGATAEQTDLRGQCFMFWNLKKTVGVPVLIALLVGKAAIDGQSISSDDHVKNAIVVLRKLFKDASVPDPVASVVTNWGLDPFSRGAYSYVAVGASGRDYDILGRPVSDCLFFAGEATCKEHPDTVGGAILSGLREAVRIIDLVHSGKDYVAEVEALQTYQMQSDSERNEVKDMSNKLDACELSTALCKTSSDASYPLFSKETLLQEMFFSAKTTSGRLHLAKELLKLPPDVLKSFAGSKDGLSTLNSWILDSLGKNATQLLRHCVRLLLLVSTDLLAVRLSGIGRTVKEKVCVHTSRDIRAIARQLVSVWVEVFRKEKASNGGLKLLRRMPSTESSKPRSKDLLSGKPIVRAPNQVSFNPKVASKNARSAGNHSPHTAIKKPENKAAKLEAMTATRSDGSSLRSQKQQHALEPKVDNGLAMSEEEAAAFAAAEAARAAAIAAAQAYASVEAEINAPRELPKIPSFHTFAMRDHYLDESDTRKKVLSDNLVRLECISEIDSRNDKAKNPSVDHANCADVDSSKMTGDNCTQRSYSNENACLINIRDHSTDSGAVDSRFTRAWVDTDTIFIDGVKDPLAIERWQQQAMEADKEFYSRIRIPDEEDSSSQKQTCRSSASQVAESKPASDGQSRGVEHLRQGLINFISTVLMPLYRNKKVDREGYKGIMRKAVTKIIETCTEGEKMMTVHEFLDSKRKNKIQTFVDKLVERHCHMNRPPNS

>SlJMJ20

MQNKRRKLDVPIVIIDISSSSSEEEENDDDVDYIDNYASSNDDDDSCDNTNSKRKKMSFSNSRKKRESKMCHQCQRSDKERVVCCSKCKVKRYCLACISRWYPGMLEEDFLKACPVCRDFCNCISCLRLDGTAKHLMNVEVKFSDEEKLEYSKHIVRALLPALEQLNTEQMMEKQIEYQIQALPNSEVKIAKAEYQKDECIYCNYCSAVIVDFHRRCSSCSFELCVTCCKELRNGNLQADVSEVMMQYIDNGPDYLHGKGCSVTSVKNGTCAGTTKVAMTSKWKPVENGAIPCPPKDMGGCCNGTLNLRCIFSENWISQLLLKAKEISQKCKVKEMYNDSELHYSCSKSKGENGTSGSKLRKAAARESSDDNYVFCPAAVDTRRANLRHFRVYLAKGEPVVVTNVHDNALGLSWEPMVICRVCRQTKKATDVLNCLNWCKLEKNIHQFFLGYTEGRFDSYGWPQLLKLNDWPPSGLFDEQLPRHGAEFSSCLPFMEYTHPQYGYLNLALRLPDNCGKPDLGPKAYIAYGFPEELGRGDSVTKLHYVMTDTVNMLMNTQAVVPTDEQLSVIKKLKQVHKEQDQREFAADNANRTHESIKDYVPNVNEKPVLKGMNFSQEKQKCDGLKVENKKYCLRSVKAACETKKDGEDSSSLFGQDKPEGFEDADGGGALWDVFRRQDVPKLEEYLRKHFREFRHIYGSPLPQVVHPILDETFYLSTEHKRRLKEEYGIEPWTFVQKLGEAVIVPAGCPHQVRNLKKSFSSVLRTKSCINVAVDFISPENVNESIRLTEELRKLPRNHEAREDKLGVKKIIVHAMSQAVNQLEKTLLNSEAGIDTGLLLSSSISRSINSMPGPSTPKPSSSVSEDLSSSKTDKREEYVVPTENEKPKTAEVNLRSPLPVLDHLSSSNKSMPGPSMPKSSSFSGKTDKREQYVAPSEDEKPRTTEVNVHCLLPVLSANKAQERTSVPSQELKQFIDIVDVESTFHTVQSFLKSLPDQYPSQQSGSLQSNSTSSAQTLAKLIFECSIRLPLEALAHDPINEKEMCGAIAALNENPSSLLFSDEQAKQLVKLKYEFPVMVKKWRELARAELSYQEFLNNFEEDRKKLDNWIRSEAKLKSEYDKKEEQARELEALLQDIRTRQKEIMDERQEGSQEAQKLVSLAQEKAGKIESTSNELVTTKMQMDGLRKNWSNFQSTFP

>LcJMJ19

MDQPWSSSGNREDNVGIPDDLHCKRSDGKQWRCTAKSMPGKTVCEKHYIQAKKRAANSALRASLKKASRKSLGESDIYLESKSDDLDMPLMNIKSGDYPFVSGKKYTDKVSKTHFRYSPETPPTRARNSLKANDDSRREGAEFDENLRSYKTPTPSGMDTSRNRSQRSFDASAMTVSHNIADDAIIVVVLVSRQICLYISDSIGGQTCHHCRRNDIERVIWCLKCDRRGYCDSCISTWYSDIPMEEIEKICPVCHGSCSCKACLRGDNMIKVRIREIPVLDKLQYLSCLLSAVLPVIRQIHQIQCSEVELEKRLHGNEIDLFRAKLNANEQMCRNICRIPIIDYHWHYVKCSYDLCLSCCHDLRGPSMNGVKGESIENHSGERCQDRENASEHISTHRLNLSDKFPGWKANSDGRIPCPPREYGGCGYRSLNLSRIFKMNWVAKLMIPDSAYVPAERRGRIISCIANHLKILTEGIGNFRKHWAKGEPVIIKQDIWRGIRETADEKMKAENRTVKAKDCFDCSAVDIELSQFIKGYSEARIREDGPKIFMSYGTFERDMVYLLVHTCEMKLQTNKVESTQGSIVECEMNESTRNPRKDSDEEILRDVALSGHDEKNKYEATSIADENEMDDQGIENVAPKEKNVNSEQFTRDGGDMPEKCHPGAYWDVFRRQDVPKLIEYLRKHWKDFGKSENLMNDFVMRPLYDEAVYLNGDNKENLKEEFGNRNIFSSAIIKYFISCEETFFSYIGKISLYAASSAIKEVQKLVLDPKFDAELGFEDPNLTAAVSKILEIAKSKQITCS

>AtJMJ24

MQVNFDETCDSVIRMNANEQTRSANGIGNGNGESIPGIPDDLRCKRSDGKQWRCTAMSMADKTVCEKHYIQAKKRAANSAFRANQKKAKRRSSLGETDTYSEGKMDDFELPVTSIDHYNNGLASASKSNGRLEKRHNKSLMRYSPETPMMRSFSPRVAVDLNDDLGRDVVMFEEGYRSYRTPPSVAVMDPTRNRSHQSTSPMEYSAASTDVSAESLGEICHQCQRKDRERIISCLKCNQRAFCHNCLSARYSEISLEEVEKVCPACRGLCDCKSCLRSDNTIKVRIREIPVLDKLQYLYRLLSAVLPVIKQIHLEQCMEVELEKRLREVEIDLVRARLKADEQMCCNVCRIPVVDYYRHCPNCSYDLCLRCCQDLREESSVTISGTNQNVQDRKGAPKLKLNFSYKFPEWEANGDGSIPCPPKEYGGCGSHSLNLARIFKMNWVAKLVKNAEEIVSGCKLSDLLNPDMCDSRFCKFAEREESGDNYVYSPSLETIKTDGVAKFEQQWAEGRLVTVKMVLDDSSCSRWDPETIWRDIDELSDEKLREHDPFLKAINCLDGLEVDVRLGEFTRAYKDGKNQETGLPLLWKLKDWPSPSASEEFIFYQRPEFIRSFPFLEYIHPRLGLLNVAAKLPHYSLQNDSGPKIYVSCGTYQEISAGDSLTGIHYNMRDMVYLLVHTSEETTFERVRKTKPVPEEPDQKMSENESLLSPEQKLRDGELHDLSLGEASMEKNEPELALTVNPENLTENGDNMESSCTSSCAGGAQWDVFRRQDVPKLSGYLQRTFQKPDNIQTDFVSRPLYEGLFLNEHHKRQLRDEFGVEPWTFEQHRGEAIFIPAGCPFQITNLQSNIQVALDFLCPESVGESARLAEEIRCLPNDHEAKLQILEIGKISLYAASSAIKEVQKLVLDPKFGAELGFEDSNLTKAVSHNLDEATKRPQQNSCT

>SlJMJ14

MGLLAAVQHLLTELERRRFSLLLLLLLLLAFCILQPHELQLHVFTRYHNIGIPDDLRCKRSDGKQWRCTALSMPDKTVCEKHYIQAKKRAANSAMRASMKKGKRKSMDENDVYSESRSDDMDITAENQKLGDYSGSFSEKKHKEKYDESRRGYRTPPPSGMESSRSRSLKMFDSSPTAGTSEGSSNSSDNTGGQPCHQCRRNDHRVTWCLRCDRRGYCESCISTWYSNMPVEEIQRICPACRGSCNCKVCMRGDNLLKARIREIPAQNKLQYLYSLLSAVLPVVKHIHNQQCFEVELEKRLRGNGMDLCRTKLNADEQMCCNFCRIPIVDYHRHCSNCSYDLCLSCCKDLRDATKLVQDDRDGNGSIPCPPKQYGGCSSSVLSLKRIFKMNWVAKLVKNVEEMVSGCKVCDSGDLENMSEGKLFQAAHRENGDDNILYHPLSEDIRSEGIEDFRKQWSRGKPVIIKDIYDVSSMSNWDPIEIWRGVRETTEEKTKDDNRTVKAIDCFDGSEIDIQIGQFIRGYSEGRIHENGWPEMLKLKDWPSPSASEEFLLYQRPEFISKLPLLEFIHSKWGLLNVAAKLPHYSLQNDVGPKIFLSYGMYEELGKGDSVNNLHTNMRDLVFLLVHISEVKLKGWQKTKIGKMQKIFAESDHKGISGDALNVSSEGDFSKFSPVGDRGDGQYADTDSNANEMLVDPESRVTSQIGVDNLSHEDLNGSSLNSSDSSHSGALWDVFRRQDVPMLIEYLRFHWKKHGDSDHVTDDSVPSPLYDGIVYLNEHHKRKLKELFGIEPWSFEQHLGEAIFVPAGCPFQVRNLQSTVQLGLDFLSPESLGEAVRMAEEIRGLPNTHDAKLQMLEVGKISLYAASSAIKEVQKLVLDPKVGPELGFEDPNLTALVSENLEKMMKRRQVP

>OsJMJ716

MEAAAAAAAVPEELRCKRSDGKQWRCSAPSMPDKTVCEKHYVQAKKRAASSALRASLRRS

SASASAARGTTPPARMAVARPIYGRVAGEPVYVAEPALPPPPPPPRRRQPVHGLPMGNAA

GARTAAELVGRGSAGLVACSSAAGAAAAATCHQCRRVANTICCTSCDRRGYCTNCISRWY

SDIPIDDVRKVCPACRGICNCRVCLLGDNVIKARVQEISAVDKLEYLHSILASVLPVLKQ

IYSDQCFEIGVDTKAYGLRTDIIRAKVNPDEQMCCDFCKVPVFDYHRHCPRCLYDLCLDC

CRDIRRSRTSVARGEYAEGRVVDRSKDTSNKRARMEPSAESANDKSVPQRRDIKNIDIRS

LFPTWRVNNDGSITCGPHEAGGCGSSKLVLRRIFKINWISKLVKNSEEMVNGCKVHVLEN

GCSSCNDGRTLELTGHRNFGVSTCSNNGGIDRFCVFSPVLEDLKSEGIIHFRKHWIKGEP

VVIRNAFEPSLSSSWDPLNIWRGIQEIMDEEVDDDVIVKAVDCSNQAEVDIELKQFIKGY

SDGHKGEDGELMMLKLKEWPPPSVLEEFLLCQRPEFIVNFPLVDFIHSRWGLLNLSAKLP

PDTLQPEVGLKLLIAYGRHQEAGKGDSVTNLMINMADVVHMLMHTAKGHDVCPKRLQPER

SEKIANGMTMHVNAHAPVQNLNVDMGEQSPDHVSSKFDERAHASALRLQEKSSDAKLNCG

FEGSSTELSCSSHSEEPKVNGSERSQAGSVWDVFRRQDISKLNEYLTANWEELAASSQVK

NPIYEQSIYLNKYHKRILKDQYGIEPWTFQQHIGEAVFVPAGCPFQVKNLQSTVQLALDF

LSPESLGESARMAQEIRCLPNDHDAKLKMLEIGKISLYAASSAVREIQRITLDPKFNLDL

KFEDQNLTQAVSENLARVTKQRNVPCS

>SlJMJ13

MEENEAVPDDLRCNRTDGRQWRCKRRVEEGKKLCEIHYVQGRHRQMKQKVPESLKIVRNTKSKNQRKIKNPKGSLEIGFSKSERALRILKKRKPLKHKPCVSEALDEALRRMELKRGDLPLELIRVFLKRQLEKKNEKESKNASAEVMREFPNALMAIPVIPAENFNNAGSVLDVKLGLDSSSNPFSLRHFRSKNIEPLPISTMQALPFARNGKNSSKVKRRRLCHWCRRSSYRVLIKCSSCKKQYFCLDCIKERRLEQQEIKVKCPICRRDCSCRICKRSELKPNIHKESLRHKRKVPKVQLLNYLVHLLLPVLEKINEEQRIEVEIEANISGKGESDIQIQQASAGDGKLYHCSNCNTSILDYHRICSKCSYRLCLNCCRDSRHGSLTEDCKSEGSNEEQACSSNFERQSRMNHTSTSRQSFSGIHYPSSRSCSNYQACADGSISCPPAEYGGCSDSFLNLRCVFPYTWIKELEISADAILCSYNIQETEHEFSSCSLCRGSDHKDADVDSFINVAERRNSRDKFLYSPSINNLREENLEHFQKHWGEGHPIIVRNVLRNSSNLSWDPVVMFCTYLEKRSKCSLDKETAKAQNHSDWCEVEIARKQIFMGSLEWQTHATMQREIVKFKAWLSSHLFQEQFPGHHAEILQAIPLQEYMNPKSGLLNLAVKLPPEMPQTDLGPSIYISYGGPEELSQAEFISNLCCESYDMVNILASATDVLASKEQVRKIKCLMKNKKPQDHKEITSHSSDQKGKSSLHSGDTEESDLQDATGEQLPDGIAEVPFYSSDSQKGQRYEDRDGNISSDNENDSESESDVSLFCSGSVERSEDSDSDHFFEDVDGAKKEAKTSGAQWDVFSREDVPKLLEYLKRHSSEFTSMRGYSKQVVHPILDQSFFFDAFHKMRLKEEFDVQPWTFEQHLGEAIIIPAGCPYQVKQLKSCINVVLHFISPENVAECINVTDEIRLLPEHHKARGKVLEVKKMVICGMKNAIAEIRNLTHSKQSS

>LcJMJ18

MKLKRGDLQLELIRMVLKREVEKRKTCHFEDDEVADDCSDTYTDGDDTETEFTRELPNGLMAVSSVCSGANSDNAGISSAVKIGAEPPVVIKRRFRSKNIEPTPIGAVQVVPFKKEMMSFRRWKRCHWCRKRSRSLIQCSSCRKLFYCTDCVKERYFDMQEDVKKACPVCRGTCSCKACSSSRYRGSDYKDLLREKTEVEKVLQFHYLICMLLPVLRQLNEDQNAEIEIEAKIKGQKSTEVEIQQAEYNCNRPCFCNSCKTLIVDFHRSCASCSYTLCISCSREILRGSLSASIRAPFCNCPDGRKACTAGVLLSEKKSEITFKQNNDRKYCDSSALLPNWTAPDGSSDIPCPPVEFGGCGNSILHLRCFFPLSWTKDLETSAEQIVGCYELPETIDTLSSCSLCLESDHEANGIKQLQEAAMRENSNDNFLYYPTLMDIHGDKLEHFQKHWGKSHPVIVRNVLPGTSELSWDPIVLFCTYLKNNVDKSKKDEEAAKETRCLEWFEVEIGIRQLFLGSFRGRKHVDTCDEKLKLKGWLSSHLFQEQFPTHYTEIIRGLPLPEYMDPKSGILNIASKMPQQIPGPDLGPCIYMSYSSGEELGQADSVTNLCYDLCDVVNILAHATDIPVSTKQLNKIRELMKMHQVQDQRESIEAALEQKMANKVKGKSSLDGENMEEAGLCDLVGEEVTTCEAHDIHLEDRTKCQDGDDDSDSDSDCSIPCCGTTQSSRPSDRRKLDTNNSSKFTKRRLAESCGAQWDVFRRQDVPKLIEYLRRHSNEFTQANGFRKHVVHPILDQNFFLDSTHKTKLKEEFEIEPWTFEQHVGEAVIIPTGCPSCVNVVLDFVSPENVTECIKLVDELRLLPNGHEAKANKLEVKKMALYSISTAVKEIRELTCAGLQVPDQKCMNHIA

>AtJMJ28

MSENEIVPDEFRCNRSDGKQWRCKRRALEGKKMCESHHSQQSLKRSKQKVAESSKLVRSRRGGGDEVASSEIEPNESRIRSKRLGKSKRKRVMGEAEAMDEAVKKMKLKRGDLQLDLIRMVLKREVEKRKRLPNSNNKKKSNGGFSEFVGEELTRVLPNGIMAISPPSPTTSNVSSPCDVKVGEEPISMIKRRFRSKNIEPLPIGKMQVVPFKGDLVNGRKEKKMRCHWCGTRGFGDLISCLSCEREFFCIDCIEKRNKGSKEEVEKKCPVCRGSCRCKVCSVTNSGVTECKDSQSVRSDIDRVLHLHYAVCMLLPVLKEINAEHKVEVENDAEKKEGNPAEPQIHSSELTSDDRQPCSNGRDFAVVDLQRMCTRSSSVLRLNSDQDQSQESLSRKVGSVKCSNGIKSPKVCKRKEVKGCSNNLFLSLFPLELTSKLEISAEEVVSCYELPEILDKYSGCPFCIGMETQSSSSDSHLKEASKTREDGTGNFLYYPTVLDFHQNNLEHFQTHWSKGHPVIVRSVIKSGSSLNWDPVALFCHYLMNRNNKTGNTTDCMDWFEVEIGVKQFFLGSLRGKAETNTCQERLKLEGWLSSSLFKEQFPNHYAEILNILPISHYMDPKRGLLNIAANLPDTVQPPDFGPCLNISYRSGEEYAQPDSVKKLGFETCDMVDILLYVTETPVSTNQICRIRKLMKNIGRVRSKNPAKGRESRFDKGKKRDRLDDYSSSDSESSQHCLGAKCRGSEFEGEERESCNYSCEEESLSNTYGAQWDVFQKQDVSKLLEYIKNHSLELESMDSSKKKVSHPLLEQSYYLDEYHKARLKEEFDVEPWSFDQCVGEAVILPAGCPYQIRKNKSCVNAVLKFLSPEHVSESIKRVKELNQLPQSVKSKANKIEVKKMAIHKISEAVKEIRELTSSDSTGALRLYN

>AtJMJ27

MEKMRGKRIRPRDSGELVEDGRSESERKTRKKENDVVSKGRIGRGRGRGEVSKRSIEIDISNPEKDIKPDGSRKCLGSTCHHCKILTSESDLIFCSKCNKKCYCFDCIKRSYSERTHEEVRAACPFCMMTCICRACLRLPLVIKPPSEKDTDVKLKQLQYLLVKVLPVLKDIYTEQNRELEIESTIRGHPVTEANIKRCKLDPSERIYCDLCRTSIANFHRSCPNKNCSVDICLSCCKELSEGFHQERDGKKNAEGKGYECRIPAGQGKDSDAYVPLHFSTWKLNSDSSIPCPPKECGGCGTSTLELRRLWKRDWVEKLITNAEKCTLNFRPTDVDIVHECSSCSTNSDSIRRQAAFRKNAHDNFLYSPNAVDLAEDDIAHFQFHWMKAEPVIVRNVLEKTSGLSWEPMVMWRACREMDPKRKGTEEETTKVKALDCLDWCEVEINLHQFFEGYLEGRMHKNGWPEMLKLKDWPPSDLFEKRLPRHNAEFIAALPFFDYTDPKSGILNLATRFPEGSLKPDLGPKTYIAYGFHEELNRGDSVTKLHCDISDAVNVLTHTAKVEIPPVKYQNIKVHQKKYAEAMLQKQQYSGQVKEASELENKSMKEVDESKKDLKDKAANEEQSNNSSRPSGSGEAEKVIISKEDNPTQPAVSTSVESIQEQKLDAPKETDGNTNERSKAVHGGAVWDIFRREDVPKLIQFLKRHEHEFRHFNNEPLESVIHPIHDQTMFLSDSQKKQLKEEFDIEPWTFEQHLGEAVFIPAGCPHQVRNRQSCIKVALDFVAPESVEECLRLTQEFRRLPKDHSSSEDKLELKKIALYAASSAIREVKGLMQSSRRSDT

>LcJMJ8

MDSLPYRRRRTARKNRFSSQEPKMSSTTNNNNSPSRRREASRERMDVGRTRDVSKSKQMRDESDAETSKEDEWPPSKKKKVPRGANGDVAGYVEGKSEKSVSKTGVKDKSKKVKRWSFMCHQCQRSDKNGVVFCSNCNKRFCYDCITNWYHPKTREEIQKACPYCCGNCNCKACLREVPVKHCYGKVENTFKLQQLQYLLYKSLPVLRHIHGELSSELEFESKLQGIQLIEEDIMRIKLAKGERLYCDNCRTSIVDLHRSCPNPDCSFDVCLTCCQELRKIIQPGEAETSSPWILKSNIQKEKPGWQSQVALVNDLIAETSSHSFGWKANADGSIPCPHKEHGDGGTTKLELRRLYNANWVTKLIKSAEDITSCYKPPDVDFSERCCTCHRSGSEGNRNTNPEVRQAAYRNEGDDNFLYSPNAVNLTDHEIEHFRKHWMKGEPVIVRNSLDKTSGLSWEPLVMWRAMREMGANVRFEEETRSVRAIDCLDWCEVEINIRQFFTGYAKGRMHRNGWPEMLKLKDWPSSTSFEERLPRHCAEFFAALPYIDYTDPKSGLLNLAAKLPDNVLKPDLGPKTYIAYGFSEELGRGDSVTKLHCDISDAVNILTHTTKIKSDPWHHDKIKALQKIYVAEDFHEQYGGSDNAKGGTTRPMIKITYERRLKNAKCRKDVNSLENDHGGAVWDIFRRQDVPKLIEFLKKHKNEFRHINGLTVTSVVHPVHDQTIFLSDRHKKQLKKEFNVEPWTFEQHLGEAVFIPAGCPHQVRNRQSCIKLALDFVSPENVGECVRLTEEFRLLPAEHRAKEDKLEVKKMTLYAVNAAVEEARCLMRNLNWLRQQLALPKTAYGRPVGGKPPIQP

>LcJMJ9

MEEGRGGGEVGMGSKKEKGDGEGDSKAVVGFFGHQEFEVGNGCENGGLGFSSDLIEGLFGEVSGGGGDGGLESLGGGGEGDWGLSFDSGVGFGSVGIQGLFGEEMGSGNGGQGLCIEGNNSHGWNFVNGDETVKKKLGRPKGSKNKKKSYGGLEGSLVVDQVGGGDREQNESVSAKKRGRPKGSKNREKTGLEGQIGNGEDGGGDREQNENVSAKKRGRPKGAKNRKKTGLEGQIGNGEDGGGDREENESVSAKKGGRPKGSKNRKKKGLEGQIGNGEDGGGDREGNESVSAEKRGRPKGSKNRKKKGLEGRIGNSEDGGGDRSGVTTGEVNGGRVCENEVLLIKSKRGRPKGSKSRKKIVVSGEENQGMGCEENVGGDGVCSLEIVRVLGSENGEILVGGEGDRGNGFETSGGEVCGSKNGSPKKKRGRRKGSGRKKEIGGDGIVQPKDVPCMSSVAEGMAGDIAGGNEGILWPVVVGEDYGGMPMEANGGYEVESESVRQKKKPGRPKGSKRRKQNIGDGIREKDGHIDWNVLPKGLETGMQIIVEAIIDGGEDECTKAGPKNGGNEIASSKKKRGRPKGSRTSRKSHTGEEENNRTLGRPKGSKNKRVVLFGEALKRILLQKDQNRMDPAKMEEENGSNLKRDVGVPNDNRRDVGHIDTKTSTLTTNSGSVQKRPRGRPRKICKVSENSGSLDATGCQTEQRSLTCHQCLRHDRSGVVICSSCKKKRYCYECIAKCDNCRTSIVNFHRSCPNPDCSYDLCLTCCWELRKDFQPGGNEAESSKQQFVEEVRGQVTDFNGQISMDRKECGWESQRTLLADEWTADMPCSVSDWRAKADYSIPCPPIARGGCGTQTLALRRIFDANWVDNLIKAAEDLTINYRSLDIDFPQGCSLCHPANTAEDGLNGFEVRQAAYRDNDQDNFLYCPNAIQLGSNELEHFQMHWMRGEPVIVRNVFEKSCGLSWDPMVMWRAFIGAKKILKEEAYRVKAIDCLDWCEVEINIFQFFKGYLEGRRYRNGWPEMLKLKDWPPSNSFEECLPRHGAEFIAMLPFSDYTHPKSGLLNLATRLPSVLKPDLGPKTYIAYGSLKELGRGDSVTKLHCDISDAVNVLTHTAEVKISSWQQKIIENLQKKYGEEDSHDLYGLVPKASGKFRGRPRKRLRKGKYFKEVNASESDPLMKHINVEEEHEGDIQNMSKVFVDRMDVSEFSLPDEWDGDPFCSDNSHNVAVNILSLPNRVDARSICPAVEEFQSSHELEAKHETSERSMSYQNTYPYIVAEEVKLLDGKDSLEATSSGNEVDCPRTINPDTSVNIDSIEDNHSTEVEYGGAVWDIFRRQDVPKLVEYLNKHQKEFRHIDNLPVNSVAHPIHDQTLFLNERHKKQLKEEFNVEPWTFEQHLGEAVFIPAGCPHQVRNRQSCIKVALDFVSPDNIQECIRLTEEFRLLPKGHRAKEDKLEVKKMALYAANVAVNEAKNLASKVK

>SlJMJ15

MTKRNHGDGEKRSSVPADDLRCHRSDGKKWRCKGFKVEGKSMCEQHLHRASASVSVVKRVEKRRKRYVARSDTSDSDDLSEPEDDKPVPVSLGDPREVVEGEGDKKKKRGNEEKPCSKWIKSPEKSFSKGIKMVSQEDGERRVSRVVKKGVECDQNDCTNPKDKKKPDPRRKHFSTDDPYDDCQMCHQCMKSDRKVARCGKRCGKRYCSPCIKRWYPHLSEEAIAEECPVCRGNCNCKDCLRKNIIPKEAKYLGIPQENNERINCLKYLVDALYPFLKTFIHDQTMEKEMETSIRGSSLKRLRIPSAFLYKDERVYCNNCNTSIVDLHRNCTTCSYDLCLTCCQEIREGCFLRDEDRRLPEWKAKETGEIPCPPKERGGCGNNRLELKCLIDEKQVEQIMREVENLVKANSSASEAHSTEEQCTCNSNNRRKAASRSDSDDNYLFCPSSDIQEGHLEHFQKHWRMGEPVIVSNVLELTSGLSWEPMVMWRAFRNIAIKKGSSDLMVTAVDCCDWCEVDINIRQFFRGYVEGRAHPDSWPEMLKLKDWPPSTEFEKRLPRHGAEFIRALPYKEYTHPLSGILNVASKLPDGILKPDLGPKTYIAYGFAQELGHGDSVTKLHCDMSDAVNILMHTADVTITKWQLSKIDELKKKKASASDDQKELNNTDTDDHLVRKNDFASAKQEKASDVFSSDENVQLEGSLSSDQVVDLENKFDGPEEENGGAVWDIFRRQDVPKLEDYLKEHQKEFKHTLGSPVDQVVHPIHDQVFYLTTYHKEKLKQDFGIEPWTFVQKLGEAVLIPAGCPHQVRNLKSCIKVALDFVSPENVGECIRLTKEFRMLPQKHRAKEDKLEVKKMALYALERAVADLKELECNDRAQVQQPHSSSEHNPVQTKEVVE

>AtJMJ29

MDSGVKLEHMNCFQLSYQYSWTTRKKRTLKPFMSKGSSPSSSSDSRKRKLSRAEDSDDSAVKRNAKRRRKICKVEEYYEDDDCILSDWVQRNTAKRIDKRNEEVEVMVKIESGDDCTIGKWFSDVSSKRKDKRQVEVDEDEEWEEEVTLCSKIKATSSRSRTHSLSANSPENVTDVISPCRSRSPASNVSDSIQKNDCTSSRKQSGPICHQCLKGERITLLICSECEKTMFCLQCIRKWYPNLSEDDVVEKCPLCRQNCNCSKCLHLNGLIETSKRELAKSERRHHLQYLITLMLPFLNKLSIFQKLEIEFEATVQGKLPSEVEITAAISYTDERVYCDHCATSIVDLHRSCPKCSYELCLKCCQEIREGSLSERPEMKFHYVDRGHRYMHGLDAAEPSLSSTFEDEEANPSDAKWSLGENGSITCAPEKLGGCGERMLELRRILPLTWMSDLEHKAETFLSSYNISPRMLNCRCSSLETELTRKSASRTTSSDNYLFCPESLGVLKEEELLHFQEHWAKGEPVIVRNALDNTPGLSWEPMVMWRALCENVNSTSSSEMSQVKAIDCLANCEVEINTRQFFEGYSKGRTYENFWPEMLKLKDWPPSDKFEDLLPRHCDEFISALPFQEYSDPRTGILNIATKLPEGFIKPDLGPKTYIAYGIPDELGRGDSVTKLHCDMSDAVNILTHTAEVTLSQEQISSVKALKQKHKLQNKVDKQSTEDCNEKEEEEEEELNMPEISSNENEETGSALWDIFRREDVPKLEEYLRKHCKEFRHTYCSPVTKVYHPIHDQSCYLTLEHKRKLKAEYGIEPWTFVQKLGEAVFIPAGCPHQVRNLKSCTKVAVDFVSPENIHECLRLTEEFRQLPKNHKAREDKLEASLLSL

>AtJMJ26

MEGEVATNGVILKHNGVKDISLETCWPEKKKPVEATSLSSGSSDIEEEISVECPKRVANQRRKRSKADEIKTKSSRKRKCDDENKCEENEKKQRSSVKKRATTWKEEEVVVDDEKKCEQQLQLVPSSKATSRSRSKKSVSVDTWLVNNEIDVSALSSRSESELSDSYLKTEYFNDCRSMTRSLKANLGELAICHQCSKGERRYLFICTFCEVRLYCFPCIKKWYPHLSTDDILEKCPFCRGTCNCCTCLHSSGLIETSKRKLDKYERFYHLRFLIVAMLPFLKKLCKAQDQEIETEAKVQDSMASQVDISESLCSNEERVFCNHCATSIVDLHRSCPKCSYELCLNCCQEIRGGWLSDRPECQLQFEYRGTRYIHGEAAEPSSSSVSEDETKTPSIKWNADENGSIRCAPKELGGCGDSVLELKRILPVTWMSDLEQKAETFLASYSIKPPMSYCRCSSDMSSMKRKAASRDGSSDNYLYSPDSLDVLKQEELLHFQEHWSKGEPVIVRNALNNTAGLSWEPMVMWRALCENVDSAISSNMSDVKAIDCLANCEVKINTLCFFEGYSKGRTYENFWPEMLKLKDWPPSDKFENLLPRHCDEFISALPFQEYSDPRSGILNIATKLPEGLLKPDLGPKTYVAYGTSDELGRGDSVTKLHCDMSDAVNILMHTAEVTLSEEQRSAIADLKQKHKQQNEKELQEQNGLEEEEVVSDEIVVYDETSGALWDIFKREDVPKLEEYLRKHCIEFRHTYCSRVTKVYHPIHDQSYFLTVEHKRKLKAEFGIEPWTFVQKLGEAVFIPAGCPHQVRNLKSCTKVAVDFVSPENIDECLRLTDEFRQLPKNHKAREDKLEIKKMVIYAVEQALKEVETLLLDRS

>LcJMJ7

MDMVCDSGRMAAMIAKQERFSGYSDDKMQRIQGASNVSRLATNRRVRGVLNSDGKKQIEHQNKRENVGSSQMRLEGLCRKRQGGVSKSKRNPKKRRRVLIIDSSSEDEVLDEKISSDDVNLGEDEVDSDDEAIALVKIREHRRTRSLDAEHRRSRSLDAAMVKRNPNNKIKINDLMSNSSCSSSSSSSSGSSISGMKSDGNSRDKCTARNLKANKFECFRCHQCAKSERRVVVPCTKCKSKSYCIHCIKQWYPQMSEEDIAMQCPFCRRNCNCSVCLHTSGLIETSKRDVSDQEKVQHLHYLIKLLLPFIRQMCEEQTQETEIEAKIQGVLSSEVEILATLCSNDERVYCNHCATSIVDLHRSCPNCSYELCLDCCQELRMGSLSGRAELKFRYTNRGFGYMHGEDPLPESCVSGTYVDRIESQVQWNANGDGSVTCPPFEMGGCGNCLLELKRILPDTWISDLEREARNVLELFRTKLSNLKQDCSEEHLGLEAAYRKGSDDNYLYCPDSTQIQEEKVLLCFQKHWVKGEPVIVRNVLENVAGLSWEPKVMWRALCENVDSTISSKMSEVKAIDCLAGCEVEINTRQFFKGYMEGRRYENFWPEMLKLKDWPPSDKFEDLLPRHCDEFIRALPFQEYSDPRSGFLNLAVKIPSGVLKPDLGPKTYIAYGFVEELGRGDSVTKLHCDMSDAVNILTHTAEVVLTEEQHKAVKRLKRKHRAQDVKEGLVTEMTDYSSDEEIEISINEHEEFGGALWDIFRREDVPKLEQYLRNHFKEFRHTYSAPVEQSCTKVAVDFVSPENIHECLRLTKEFRLLPKNHRAREDKLEVNTSLCLSFSLMICMS

>SlJMJ17

MDNMDENDDDWNMCKEKSNMSKDGKKKQRIYEKDAVDKRTSVRRAAASVKRYDHDYHIDEWEDDIEEYEVWHHTPSDSRSQRSDVSHESKPKDSLRDIVYNSVKLSACSSLPSSTSSSGSTISRNGIDRSKNVKVNCHQCRRSDRRTVVPCTKCKEKFYCIKCIREWYSELEEEEVSEACPYCRGKCNCNFCLHSSGMLKTSKRDLPDREKIKHLQYLIIKLLPFLKEIHQEQIQEIETESSIRGVSSSSVDIKQSLCHNEERVYCDNCSTSIVDLHRSCPDCSYELCISCCQELREGKCLGNSKKAVVKYPNIGYDYMHGGDAEPERYDDMEIPQDQNKPITWVTNYDGNIMCAPEAIGGCGNFVLELKHLLPKNWISTLEAKAERILIQCNFSEIISQPICRTDDPEQLHRAASRVGSDDNYLYFPTAKDAIEDDALLHFRRHWAKGEPVIVQNVLAHTSGLSWEPMVMWRALCEGTDSKILTSMSEVKAIDCLANCQVPINTRKFFKGYTEGRRYENLWPEMLKLKDWPPSDKFEKVLPRHCDEFISALPFQEYTDPRIGILNLAVKLPAGVIKPDLGPKTYIAYGLSEELGRGDSVTKLHCDMSDAINILTHTAEMAITDEQRSAIEIVKQMHRAQDERERIECEADKYPMKMSSDISREEKTFDDSETTGGALWDIFRREDVPKLSEYLLKHAKEFRHTFCCPVDQVFHPIHDQSFYLTLEHKRKLKEEFGIEPWTFEQRLGESVFIPAGCPHQVRNLKSCTKVAADFVSPENIRECFRLTAEFRTLPKGHKAREDKLEV

>OsJMJ720

MAAEEVEAAAIDGSGEEAKRKSGKQRGSGAKGRRRNGDRAFRPPAMRPEEEGRGVATRPG

ALRERKPPPNAFNAPDDDEDVEKTDQLLEPLNKPKRRDAGKKRGPRKKKVDQENIKTHRH

NANAVKGKMLVNDKVSKTEKKRKRGDTGAAENNGKGKKMLTGENALMCHQCQRNDKGRVV

WCKTCNNKRFCVPCINQWYPDLPENEFAAKCPYCRKNCNCKACLRMRGVEEPPRKEISKE

NQIRYACHVLRLLRPWLIELRQEQMAEKELEAKIQGVSVDQIKVEQAVCDLDERVYCNRC

STSIVDFHRSCKHCFYDLCLTCCQELRKGEIPGGEEVEILDPEERDKDYAFGKILSDGEN

QRDSLKCRSDTQNSESNKGMASDENQKKALLLWKANSNGSIPCPRKEKEDCSFSSLDLKC

LFPEKLLPELEDRSEKVFWSETFAKELGRTSELCPCFDHSGKIRSDSKKLRQAANREDSS

DNYLYCPVATDIQDADLLHFQMHWAKGEPVVVSDTLKLTSGLSWEPMVMWRAVRERTKGK

AEDEQFAVRAVDCLDWCEVEINIHMFFMGYTRGRTHPRTYWPEMLKLKDWPPSSSFDQRL

PRHGAEFISALPFPEYTDPRYGPLNLAVKLPGGVLKPDLGPKTYIAYGFSEELGRGDSVT

KLHCDMSDAVNILTHTAEVPCETYDAVQIKNTQKKMKMQDDMEIYGMIESGSELKPSACP

VELGNKAVGEAPKASCSKENVHTLKDKSNGLDINASPPDDAGGDARDEALSYESVVHSDV

AQCPNHNHETNNSDDARIGAQRCQKKAKGRPPKTGSGVSEHQESGGALWDIFRREDSEKL

QDFLRKHAPEFRHIHCNPVKQVIHPIHDQAFYLTAEHKRKLKEEYGVEPWTFEQKLGEAV

LIPAGCPHQVRNLKSCIKVALDFVSPENVGECVRLTKEFRRLPSSHRAKEDKLEIKKMAF

HALNEVLNFLDPPSSEGSKEAAEKPRRGRGRPRKH

>SlJMJ19

MEETIENKVKFDSLEETEDDIQVKFETLEETVESMEETVELKLESLEDLKVVEEEGMVFTEVVKPKKKRGRPPNKNKGKLKLEEDTGKETCIKKNTNLGRKKNHVQKKNHVNEKEDEIDSMREEEKIDFDRVSATHRPVRSSRKKAIEKITEFSLQMNEWDEEDLSAHKKRRGQGRKSGVKTEEGNADSGQKIASKKRGIMSLKVNGGDSNSKEEEGNGSKKHRAEEQDKVERSESARQSKDNASNPRARKRKDENGNEILSNMCHQCQRNDKGRVVRCTSCKTKRYCIPCITTWYPGMPEEAFAESCPVCRQNCNCKACLRLDGPIRALKDSQCQISEEEKFEHSKFILQILLPFLRRFNAEQVMEKEIEAKTRGPSVSELVLKKAKCQKNERMYCNNCKTSIFDFHRNCSSCSYDLCLTCCRELRDGHLKGGDEEVIVEFVDKGVDYMHGDVRPGSSSDTRTSRRSKSSKKMVENDSVDDARLAFEMEPGDNGGHLQDNSGGPAGEWKSNEDGSIPCPPQKFGGCGKGNLDLKCLLNKTEGLSELLARAEDIAKRFELEYMPEISQGPCCCRNSVNEDDIQKSKMCKTVSRDGCDDNYLYCPAAKDLQQEDLKHFQCHWLKGEPVIVRNVLETASGLSWEPMVMWRACRQIKNLNHPLLLDVVAINCLDWCEVEVNIHQFFKGYLEGRTDSAGWPQILKLKDWPPSDLFDERLPRHGAEFVRCLPFQEYTNPQNGFLNLAVKLPPNSLKPDMGPKTYIAYGVRQELGRGDSVTKLHCDMSDAVNVLTHTQAINLTPEQLSVMEKMKKKHAEQDKTELQMAEDEKKCKNEASSELIDDYCVHSDRSSRRDEEKTEHSEVQSLSCEPDCGNPSIIPSASCVEPEGDTDVDLVINGAINSTSYSEASGGIRIDNDKNDECKDDPVFGKNEVFEDMEGGALWDIFRRQDVAKLEEYLLKHFKEFRHIYCCPVPQVIHPIHDQTFYLTEDHKRKLKEEYGVEPWTFVQKLGDAIFIPAGCPHQVRNLKSCIKVALDFVSPENLHECIRLTEEFRTLPQNHRAKEDKLEVKKMSICAVRDAVIELEKLSKESTGNNEKKRHTIMDDDREHSGDD

>AtJMJ25/IBM1

MDSVEEEGVVRVEEENGRGGLRRHRRVSTKLANYVDPPTDDEEDGGPKRKGKRGGNRAPKKTPKKDEEMQKNEIDEANRVTGLVKEKRAATKILNRKDSIIEVGEASGSMPKEVKGIRIGKRKGEIDGEIPTKPGKKPKTTVDPRIIGYRPDNMCHQCQKSDRIVERCQTCNSKRYCHPCLDTWYPLIAKEDVAKKCMFCSSTCNCRACLRLDTKLKGINSNLIVSEEEKVQASKFILQSLLPHLKGINDEQVAEKEVEAKIYGLKFEEVRPQDAKAFPDERLYCDICKTSIYDLHRNCKSCSFDICLSCCLEIRNGKALACKEDVSWNYINRGLEYEHGQEGKVIEKPANKLDDKLKDKLDGKPDDKPKGKPKGRPKGKPDDKPKGKLKGKQDDKPDDKPDEKPVNTDHMKYPSLWKANEAGIITCCCGAGELVLKRLLPDGWISELVNRVEKTAEAGELLNLPETVLERCPCSNSDRHIDIDSCNLLKAACREGSEDNYLYSPSVWDVQQDDLKHFQHHWVKGEPVIVRNVLEATSGLSWEPMVMHRACRQISHVQHGSLKDVVAVDCLDFCEVKVNLHEFFTGYTDGRYDRMGWPLVLKLKDWPPAKVFKDNLPRHAEEFLCSLPLKHYTHPVNGPLNLAVKLPQNCLKPDMGPKTYVASGFAQELGRGDSVTKLHCDMSDADLWTKLYPFNVSPSIAFLVLHRPGIRFLVLLMPNMQPGIGNLKKKHAEQDLKELYSSVANKEEMMEILENSRQQVQNVETDDGALWDIFRREDIPKLESYIEKHHKEFRHLYCCPVSQVVHPIHDQNFYLTRYHIMKLKEEYGIEPWTFNQKLGDAVLIPVGCPHQVRNLKSCNKVALDFVSPENVSECLRLTKQYRLLPPNHFAKEDKLGVKKMIVHAVDKALRDLSGEKSPEPEEKKQNMRGPKKGAAKAVAKALKDLSPSEKKSSEAAEEEISNGIVNAIDKGLKDLPPSEEKSSEAKVEISNGIVSAMDKDLEHISSSEKKSTEEEGVKRPNIVRTYERRKKLGSEVTNAYIDRLEMEKM

>LcJMJ6

MVGLGKKKRRGRPPGIKNKKPKDVKDENGVEEKVENLGEDKQPSFDGVKVEKEEENNGVVVEEKEEVTNLLKKRLGDGRRRVYYNEDQMFKGLLDDDFEKKKRRGPRKGSLKNKQLLTPENGNNQAEEMETETGGGVLRRSTRPKKMNAAAIEMDTKKKRKRETEDSNMCHQCQRNDKGRVVNCKSCSGRKRFCIPCIATWYPKMTEDEIAECCPFCRKLKKEGKVEFNRDLLKPETIERMLKIKKSDIMFGEDEKVSHSKRILQALLHYMKTFSQEQTKELIMEASIRGISASEIKLQNAGISLDERIYCDNCRTSIVDFHRSCPNCKYDLCLSCCLEICDGSSEGRCEPVIIEYISRGLDYLHGGKPLSVNPSEGKQSDSAVEAEKAVKSSISVWKVNENGSIPCPTIELGGCGNGLLELVCMFSENWVADLVEKAENIAKSLHVEDMPESQEERCPCFNSEGQVNMNNGRLRKAASREDSVDNYLYNPTADEIQPGDLKHFQRHWANGEPVIVSNVLESACGCGLSWEPMVMWRAFRQITNLNHDRHLAVTAIDCLDCCEIFCVPASNAWANYILRFVSCMEGPINIHQFFKGYTEGRYDKKGWPQILKLKDWPPSTLFEERLARHNAEFLCILPFKEYTHPQLGLLNMATKLPKKSLKPDLGPKTYIAYGIAQELGRADSVTKLHCDVSDAVNVLTHTAEVQVKPESLDSIKKLKQKHKKQDDEELFGKSDFPEKELEAADCGESGELPDISENKSEAADSRDTGEPSDISENKLEAADGGAVWDIFRREDVPKLEDYLKKHYREFRHFHCCPIQQVVHPIHDQIFYLSSEHKVKLKQEYGVRLGVRCFKVQVPCLESLETRLDRGVEFAPVLCSRALVKLPTSGGVLLGLYIFLPETFSESWYPIAGMVLIGIPWLFWLFAYIYRCFVSPSGNRVYNRPSSAATTPSSRTPAMAETAATAPASSSEEESPVQSPGVSPGRHVHFGAVVVMGSEGNNKNRNGDQNGTGEGHLRSSIPESDHQGESADKEIVSKKASVHPRESEIPLAYSSGC

>LcJMJ4

MATKLPKKSLKPDLRPKTYITYGIAQELERGDSVTKLHCDVSDVVNVLTHTAEVQVKHESLANIKKLKQKHEKQDEEELLGKSDVPEKKLEAADCGESGELSGISENKSEAADSRDTGEPSDTSENKLDAADGGALWDICRREDVPKLADYLKKHYREFRHFHCCPIQQVVHPINDQILYLSSECKVKLKQEYGWFLGMLIRLMGATKLLKRIRRRSSSFTFGASVVTRGFRGLYARLSPTLVEIVPHAGLQISLLACSPLAMGIFSGKYFSADGGPGDALLNLFKDICYCSSTSLQKLSEKFPGMGTNISSKGNFSFSRRKSDVSECVAFSFFLDKELRF

>LcJMJ5

MIFASVAAWKYVMAARREDRFDYLHGGKPLSVHPRGGKQSDSTVETEKAVKSSISVWKATENGSIPCPTKELGGRGNGLLELVCMFSEDWVADLVEKAENIAKSLRVEDMPESQEERCPCFNSEAQIFCVPASNAWANYTCRFVSCMEEYTHPQCALLNMATKLPKKSLKPDLRPKTYITYGIAQELGRGDSVTKLHCDVSDAVNVLTHTVEVQVKPESLARIKKLKQKHEKQDEEELLGKRDTGEPSDTSENKLDAADGGALWDICRGEDVVHPMHDQIFYLSSEHKVKLKQEYDYIYTCACTMVFGYAYPAYECFKTVEKNKPEIEQLHFWISLLACSPLAMGIFSGKYFSADGGPGDALLNLFKG

>OsJMJ715 MEMEEAVDGKHPKRGRGRPRGRRGRGRGRGRGGRSLASPAAGPGDQGPRRRRGVVPAAAAAAGGRALRERRPAPGAYRESGADNDDDGGGDDEHDEQNDDGAEKSDNQVVDSLNEPNRSNTGKKRGRPKKVKAEQEDSNQLSNGKHLGENNGNDEAIMMKPSKESKKRGAGKKQEEEENNTISIEDEMCDANNKKGKKMLTGENALMCHQCQRNDKGRVIWCKSCNNKRFCEPCMKRWYPGLSEVDFAAKCPYCRKNCNCKACLRMIGVEKPPEKKISEENQRRYAFRIVDLLLPWLKELQQEQMKEKELEGRLQGVSMDEVKLEQADCDMDERVYCDRCKTSIVDFHRSCKACSYDLCLACCWELRKGEIPGGEEAKSVQWEERGQKYVFGNISKDEKKRVSSKRHMETPSTETCNDMAVAGDPNNPLLLWKANSDGSIPCPPKEIGGCGASSLVLRCLLPEIMLSELEHRANKVIKREAFDKAINETSDQCPCFYHTSKIRTNATREAANRKGSSDNYLYCPDANNIQEDDLSHFQMHWSKGEPVIVSDALRLTSGLSWEPLVMWRALREKKTNGDVEDEHFAVKAVDCLDWNEVEINIHMFFMGYMRGRRHPMTFWPEMLKLKDWPPSSMFDQRLPRHGAEFITALPFPEYTDPRYGPLNLAVRLPAGVLKPDLGPKTYIAYGCYEELGRGDSVTKLHCDMSDAVNILMHTAEVSYDTEQLDKIAKIKMKMREQDLHELFGVSESGAKGKADDEASKISCNMENKHTSNQSTKGLDINALPPDDSGSDIGDKPSFCQSEVESELTQCSKHNHEVNSSVKMHAGAHCTSDNQGYIDRSGFKRKDSDCSDQQKTGGALWDIFRREDSEKLQDYLRKHASEFRHIHCNPVKNVSHPIHDQTFYLTVEHKRKLKEEHGVEPWTFEQKLGDAVFIPAGCPHQVRNLKSCIKVALDFVSPENVGECVKLTGEFRRLPSDHRAKEDKLEIKKIALNALKEVVNFLDPLPKGDEVVEVTKPKRKYGNRRGDLKSGEDQPIDESIEERKPKKRGRSKR

>LcJMJ13

MGALLLLVPDFLEDFDVADAIRGLRSHGVDLIERIYIVEGRVCLAKEARNGLEFLKCKRLQRMKSETLTETIGVSNMMSRSGGDALRASSSCGVRLQGNVDSFSQSNAVSNRKDVFLKRKVNKFDTNDLEWTEKIPECPVYCPTKEEFEDPLVYLQKIAPEASRYGICKIVSPLSASVPAGFVLMKEKAGFKFTTRVQPLRLAEWDTDDKVTFFMSGRNYTFRDFEKMANKVFARRYCSAGCLPATYLEKEFWNEIACGKTEFVEYACDVDGSAFSSSSSDPLGSSKWNLKNLSRLSKSILRLLDTAIPGVTDPMLYIGMLFSMFAWHVEDHYLYSINYHHCGASKTWYGIPGHAALEFEKVVREHVYTRDILSTDGEDGAFDVLLGKTTLFPPNVLLEHDVPVYKAVQKPGEFIITFPRAYHAGFSHGFNCGEAVNFAIGDWFPLGARASWRYAHLNRMPLLPHEELLCKEAMLLYTSLVLEDSDCSSADSVSHHCIKVSFVNLMRFQHRARWLVMKSRTCTAISPNYHWTILCSICKRDCYVAYLNCNCSLHPICLRHDLMSLKFPCGSNFTLFLREDIAEMEAAAKKFEQDGVLQEIQHEAEGGDDLYSYPLSNMFQSAVEDGYSPYCEIIMELNPETAGKTLVQFTKLEHSCHSPPTSSRSIKDCSPELAEASLSSAASTLCSFVKNVQSSTTYNGKPDFRVVDIKKSSEEVSRIRQESSLFSQTRNECHSAHENSTQGSEVRAIVDQPSDDSDSEIFRVKRRSSAKVEKRTTNDSISSKNFEHQGLKRLKKHQPEGRCGQLMPSECCRTDLSNRNISHYSNHKEEASDSGLVEDRSARVSAIPLTIKYKKLSDEEASSRQRDNQRNDRFRHELGKAMREPPPLEIGPKRLKVRGPSFLGPDNRYD

>LcJMJ11

MGNNVEIPCWLEGLPLAPEFRPTDTEFADPIAYISRIEKEASAFGICKIIPPLPKPSKKYVFSNLNKSLSKCPDLGSDVDLLNVSNSSRMGSRDVSNDGGEARGFFTTRHQELGHSVKRTKGMVSNDSLQLGVHKQVWQSGEVYTLEQFESKSKVFARGLLSLIKEVSPLVIEALFWKAASEKPTYVEYANDVPGSGFGEPEGQFRYFHKRRRKMKSWKSYRCRGRDDGKNNEIDTIRSSHNGEATDASVKDDTKSCLETSNSSTTSSSIPLDDNSQSLRRKSVHGSADVEGTAGWKLSNSPWNLQVIARSPGSLTRFMPDDIPGVTSPMVYIGMLFSWFAWHVEDHELHSMNFLHTGAAKTWYAVPGDYAFAFEEVIRSEAYGGNIDRLAALALLGEKTTLITPEVIVASGIPCCRLVQNPGEFVVTFPRAYHAGFSHGFNCGEAANFGTPQWLKVAKEAAVRRAAMNYLPMLSHQQLLYLLTMSFVSRVPRSLLPGARSSRLRDRQKEEREFLVKKAFIEDILIENNTLSILLGQESSVYAVLWHPDLLPCPSKDIQLTTETDAVSTEPGENVSHSQSERNNNHSNLLDEMSLYMETLNDLYLDDHDMSCDFQIDSGTLECVACGILGFPFMSVVQLSEQASMELLLTNHDLVHEEPGILESKVAHPFIGLVGTVSSSVSEDLSPVPETSVPPKDLPVASVTKFTKRWNTSSKFLRPRIFCLEHALQVKELLQSKGGANILVICHSDYQKMKAHAVVIAEEIGTPFNYTDVQLDSASQEDLHLIDLAIDDGQNDECNEDWTTKLGINLQHCVKVRKNSPSKRVQHALSLGGLFAERSPSSDFSKVTWQSRRCRSKTKLNPPAQSKLCQSIEIKTDEVLGDKSNDVVKRKEQIIQYSRRKCKLKPDFSVGESSGDGRSRKLLLEEASAATLDNHNRINSNISSCNIADDGSISSGLALLPIGMSERLHEIQVLEASRDLSLNYSPSRVADSLATASIVVDSIEQIEIETSKEVKMEGDCMAICNSAEMGENIEDACNSIEKIEISYGETCSSRSIASGDIFVIQKDRVTDNDIMNGTCDLASEGQNDIVTVRDISMNEVCGAVNYATFYVTSPSVECFDVLIEDTVVDNSSMNNKVCDLMNDNEVHQDMQVTNEKSNDEPLSCYNKLINQPNPASKEESCEDLNNACPGNRMPLHDHVLQESDSAKVINKEHVMSSVRLMNQSGSVSVESSGIPGNECAAADLCGAETSDDGELGLRMCTVRGRKEELVSGSVTCMELDRSSPRSVEGFSDIPRRNCGEEDLCVDVIDPEMQPKFHTTTRTSVDELVSGLQIEGNAVSIGACSELPNGICAADKLGRDVSRDTESLEDAQTSNAANEKPNPSSLAATNQPIVIKTYSRTRRESCSRGKLFHGNEVCSKGNREQETNKSTMEDAVSNAGKGRKRNREMELKTEDQVDYSGFIRSPCEGLRPRAASKTEADISKMPEEKPVTKMARRESNVLVPRQGKKKTMKGCYKCDLDGCCMRFETKRELSLHKSNRCPHEGCGKRFSSHKYAIIHQRVHEDDRPLKCPWEGCSMSFKWAWARTEHIRVHTGERPYQCKVEGCGLSFRFVSDISRHRRKTGHYENISS

>LcJMJ12

MKGEMGEAIQQQEVLPWLKTLPLAPEFHPTLAEFQDPIAYIFKIEKEASQYGICKIVPPLPPAPKKTAITNLNRSLADRAAVAASSSSAATSSGPTFTTRQQQIGFCPRKPRPVQKPVWQSGEYYTFQQFETKAKNFERTYLKKCPNKKGGPLSALEIESLYWKATVDKPFSVEYANDMPGSAFVPVRKNRDAVGEGATVGETAWNMRGVSRAKGSLLRFMKEEIPGVTSPMVYIAMMFSWFAWHAEDHDLHSLNYLHMGASKTWYGVPMEAANAFEEVVRVNGYGGEINPLVTFSTLGEKTTMMSPEVFVTAGVPCCRLVQNAGEFVVTFPRAYHMGFSHGFNCGEAANIATPEWLSVAKDAAIRRASINYPPMVSHFQLLYDLAIALCSRIPTAISAKPRSSRLKDKKKDEGETLVKERFVQDVVQNNELLHILGKGSPIVLLPQSSSDISVCSDLRVGSQPGVNPGMSIGLCSYKEAIEHPRGLVCNDIMVGRSNGMMPVKRFYSAKGKFTSLCERNSKLSGTDNLCTWTSQILPTDTEREGTVHTDRLSDQRLFSCVTCGILSFACVAIVQPKEPTARYLMSADCSFLNDWIGGSGVPNTGFNVAAGDGIMSEQNSSPRWMGKSGLNGLCDVHVCAQIQKADQTNELVLAKQKQRDTSALSLLASTYGNSSDSEEEHVEPDVSVCGDETNLTKCSPERKHQLGFDGSATETDNLSLARLDCGDEAALHVIDCQGETKLGRANFKDKSDQNSDSSIEFETDNPDSSKTIGLDGSFGDPVAVLHSTNSYQKASHTANCCPSIQGAESVEFINAIIPSENTDMSFAPRSDEDSSRLHVFCLEHAVEVEQQLRPIGGVNILLLCHPEYPKIVAEAKLVAEEMGTDSIWNDISFRNATKEDEERIQSALDSEEAIPGNGDWAVKLGINLFYSANLIHSPLYSKQMPYNSVIYNAFGRSSPGTSPTQSNHGRRTGRQRKVVVGKWCGKIWMSNQVHSFLAQKDPEEPEQERSCRTWATPDENFERKSETTRKRRMPAESRSRKKAKCIDAEDAHSDDSLEDDTHVQQRRIVKSKPAKFMESEDASSYDSLEDNSHQQKRSISRKKKAKCIEREVGVSNVLPDNNPIKQYRRMRKGQQAKSIAREYAVLDDLLESSSLKQYRRIRRTKPAKNSEGEDEVSENSLGDNNYRSQHRRIPRSKQARCNEREDAVSDDSLEDNLRQLQHKQLLKSNKAKCFVKTDAESDDSLEDNSRRLQHRRIPKSKRAKIVNREDAISDDSLDENSSQLHRRFAESKQYKWIERDTVSDDSLEDNSHKLHRRIPKSKQTKWIEREDAVSDGSLEDNFLRQHRKILRSKLAKSIEREDSVPLEDDTRQRRRIPVNQKAKLFGREGAVSDDSIEDNSQRQHNRILRNKQGKTVQTKKQETLWNSKSGRRSSTKQQTPRQMKKGALRQVKQVTPRQRNVKTEQKLDSFVEDEQEGGPSTRLRKRIPKPSKELKAKSKEKKPTKNKKPKNASAVKGLGTSSNSKIKNEDAEYQCDMDGCTMGFGSKQELVLHKKNICPVKGCGKKFFSHKYLVQHRRVHLDDRPLKCPWKGCTMTFKWAWARTEHMRVHTGARPYVCNEAGCGQTFRFVSDFSRHKRKTGHSAKKARG

>SlJMJ1

MAEASGNIEVFSWLKTLPVAPEYHPTLEEFQDPIAYIFKIEKEASKYGICKIVPPVPAPPKKTALANLNRSLSARAGSNGPTFTTRQQQIGFCPRKHRPVKKPVWQSGETYTVQQFQVKAKAFEKNYLRKNSKRALTPLEVETLYWKATVDKPFSVEYANDMPGSAFAPKKASLAAGGIGEVSTLADTEWNMRGVSRSKGSLLKFMKEEIPGVTSPMVYLAMMFSWFAWHVEDHDLHSLNYLHMGSGKTWYGVPRDAAVAFEEVIRVQGYAGETNPLVTFATLGEKTTVMSPEVLLSAGIPCCRLVQNAGEFVVTFPRAYHSGFSHGFNCGEASNIATPEWLRVAKDAAIRRASINCPPMVSHFQLLYDLALSLCSRVPKNIRIEPRSSRLKDKKKSEGDMLVKELFVEDLNANNYLLHILGEGSPVVLLPQNSPGISICSNLVAGSQSKVNSRLFPSSSNSDHEVKSKKDSAYDDRKLGRKQGMKQYAGISLEKGKYSSWHTGNSLPDSGRKDDAQSSPETEKVNLDAARGMTYKCDTLSEQGLFSCATCGILCYTCVAIIRPTEAAARHLMSSDYSDFNGWTGSVSGITATGRDPNAAESDSSSGRFVKRAPALIDDPVESSDRIQKLNNGSVEELSRTNTRKETSSLGLLALAYANSSDSDEDEIEVDIPVEACESRHTESEDEVFLRVIDPYGNHRQKRAVSQGRNCQKFDNSVQLENESYPSGESNTLFGRSSHQPRSHQVPAKCISNIREIAQNNAVAPFDNARMQFTSTSDEDSFRIHVFCLQHAVQVEEQLRRIGGAHISLLCHPDYPKLEAQAKQVAEELGSDHFWREISFREASKEDEEMIQSALEIEEAIHGNGDWTVKLDINLFYSANLSRSPLYSKQMPYNFIIYNAFGRDSPDNTPEKSEYTGRGLGKQRRAIVAGKWCGKVWMSSQVHPLLAERTIDEEQEQNKSISALIKIEVKSERPRERTPTSKTVATTCKTGKKRSSTAASRNASNAQLIIADDHDDSLLSSILQQHRRKTNLRSKRIKYETPEPQKDVDKKKIFGSLIDDDPDGGPSTRLRKRIPKPSNESPAKSVKAKPAPTKQHESKKGPKVKLPFANSIAKKEPVTKGPRSNIGKRMREEGEYHCDLEGCSMSFSSKQELTLHKKNVCPVEGCKKKFFSHKYLVQHRRVHMDDRPLKCPWKGCKMTFKWAWARTEHIRVHTGARPYACSEIGCGQTFRFVSDFSRHKRKTGHISKKGRS

>SlJMJ2

MDDIPEWLKGLPLAPEFRPTDTEFADPIAYISKIEKEASAFGICKVIPPLPKPSKKYVLHNLNNSLSKCPDLNSAGAPVFTTRHQELGHTEKKKFPFGAQKQVWQSGQLYTLDQFETKSKNFARTQFGIVKDISPFLVEAMFWKTAFDHPIYVEYANDVPGSAFGEPEENFCRTKRPRNRKILDRTSSTTSVDKGRSHHSVDTPSSSLLTPLSNSSPFRPKGCSNAAEMEGSAGWKLANSPWNLQVIARSPGSLTRFMPDDIPGVTSPMVYIGMLFSWFAWHVEDHELHSLNFLHTGSPKTWYAVPGDYAFSFEEVIRCHAYGETTDRLGMNTYFFVNNNVKFTKE

>SlJMJ3

MAEAGDRLSCEKRVPEKFKRKKDVLNVVEKVTKKGSARRLKKPYLHGPSHGKVSQKTKAAAQISMDEFDWTDTIKECPVYRPSKEEFEDPLVYVQKIAPEASKYGICKIVSPLGSSAPAGVVLMKEQKRFKFTTKLQPLRLAEWNNNDMITFFMRGRNYTIRDFEIMANKATARRYCISGCLPPAYVEKEFWKEMTVAKRGTVEYGINIDGSAFSSTFSDPLGSSKWNFKILPRLQRSTLRLLVNEIPGVTDPMLYIGMLFSMFAWHVEDHYLYRQVILSTFSLMFQLFSFCTFSLLLFLSALTIITVGLPKLGMEFLVMKLFSLRILSGIAFIMRKSYQKMGSMELLIFFKREQLWFLQRFCYNMVSRFTRLCKCQESLSLPSLEHTIQDLVMAVNFAIGEWFPFGAAASERYALLGKVPIIPYEELLCAEAMLLSKSLASRPYCTSDLIDVRCVMTSFSCLLRSYHRARWCLEKLRTSLKMCSKPRGSFTCILCKRICYVAYLECKCFAGPICLFHDFETFNCLCGSSCSLFVTEDISTMEAVAQMFEAEEGMRYEVEQKMKSLPYLWIQTLFPRIQGKYRPYCEIMSSSIQNVDTGIKMSMIRRSTAQGKQMRNMKKKRINGA

>SlJMJ4

MSRSGGDALRSTASCGVRIRVNADMHSGSGTSLNERNVFPKHKVAKFDTSDLEWTDKIPECPVYYPSKEEFEDPIVYLQKITPEASKYGICKIVSPIMASVPAGVVLMKEKVGFKFTTRVQPLRLAEWDRDDRVTFFMSGRNYTFRDFEKMANKVYARRYCSAGCLPPTYMEKEFWHEIASGKTESVEYACDVDGSAFSSSPNDELGKCKWNMKRFSCLPKSVLRLLEKAIPGVTEPMLYIGMLFSMFAWHVEDHYLYSINYHHCGAAKTWYGIPGHAALDFEKVVRENVYNNDILTADGEDGAFDVLLQKTTFFPPNILSEHDVPVYKAVQKPGEFIVTFPRAYHAGFSHGFNCGEAVNFATGDWFPIGSIASRRYALLNRVPLLPNEELLCKEAMLLLTDLELEYSAISSADLITHHTIKVSFINLMRFHHRARWCFLKLKAFSGISSFSHSTILCSICKRDSYVAYLNCSCYSHAACLRHDPRSLHFPCGSSRTLCLREDILDIETTARKFELDDNVLHDVAHYQEGDDLALLLNMFPQAEEEGYVPYCEINFEWTVKAEDRVEQTFDEQASNAPASSIELVPNTGSTMERNDCLPTSINVQENAYNSQEGNNISVKPLRDISRCRSERLACSPSADYLKVHEKIAHVSDVRTVIDQDDDESDSEIFRVKRRFRAENGSRRDSTSVNIEHQVCGYWGLSLGAIFCSFWVFKLKFLVKQFYDKGKRCWFLFGFSMCIFCIHSCSPFRVLHKLA

>SlJMJ5

MVEGRVCMSREAKLEFLKRKRLQRMKTESLNDLSCVSNMLSRSGGDALRSSASCGVRIQVNTDSYPGSGASFNGKDNFSKHKVAKFDTSNLDWIDKIPECPVYCPTKEEFEDPLVYLQKLAPEASKYGICKIVAPITASVPAGVVLMKEKAGFKFTTRVQPLRLAEWDTDDKVTFFMSGRNYSFRDFEKMANKVFSRRYYSAGCLPPTYLEKEFWHEIACGKTDSVEYACDVDGSAFSSSPNDQLGKSKWNLKELSRLPKSVLRLLEKSIPGVTEPMLYIGMLFSMFAWHVEDHYLYSINYQHCGAAKTWYGIPGHAALDFEKVVREHVYTNDILSADGEDGAFDVLLGKTTLFPPNILSEHGVPVYRAVQKPGEFVVTFPRAYHAGFSHGFNCGEAVNFATGDWFPMGSISSRRYALLNRVPLLPHEELLCKEAMLLCTSLKLEDPDYSSSDLITHRSIKVSFLNLMRFQHRARWCLARLKAFSCISLFTHGTILCSICKRDCYVAYLNCNCYAHAVCLRHEPRSLDFPCGSNRTLCLREDILDMETAARQFEQDKVVLHEVQQQHRKTDDFSKLLKMFPRAEDDGYVPYCEINFEWPEDSVEQTIYEEAPNGSGPVVSDLNSSMEPKDYLSTGVNVQGNANCNLGDSSSMKLHGDVFSCGSERSEISSSASSKVHQKVAQETDCRTIIDQDSDESDTEVFRVKRRPRAEHRSVHDSMSINVENQSFKRLKKHQSGRLGSLCLPEHSSTCDINHRSVAISSQSKEALDFHPRDKSVRGGTVPVCIKLKKGVGYEQDEHKRDDRLPFELGQSKRREPGRTESGSKRLKVRGPSVLGFGGRMDGLNR

>AtJMJ11/ELF6

MGNVEIPNWLKALPLAPVFRPTDTEFADPIAYISKIEKEASAFGICKIIPPLPKPSKKYVFYNLNKSLLKCPELVSDVDISKVCKEDRAVFTTRQQELGQTVKKNKGEKGKSNSQRSGVKQVWQSGGVYTLDQFEAKSKAFYKTQLGTVKELAPVVIEALFWKAALEKPIYIEYANDVPGSAFGEPEDHFRHFRQRKRRGRGFYQRKTENNDPSGKNGEKSSPEVEKAPLASTSLSSQDSSKQKNMDIVDEMEGTAGWKLSNSSWNLQMIARSPGSVTRFMPDDIPGVTSPMVYIGMLFSWFAWHVEDHELHSMNYLHTGSPKTWYAVPCDYALDFEEVIRKNSYGRNIDQLAALTQLGEKTTLVSPEMIVASGIPCCRLVQNPGEFVVTFPRSYHVGFSHGFNCGEAANFGTPQWLNVAKEAAVRRAAMNYLPMLSHQQLLYLLTMSFVSRVPRSLLPGGRSSRLRDRQREEREFLVKRAFVEDILNENKNLSVLLREPGSRLVMWDPDLLPRHSALALAAAGVAGASAVSPPAVAKKELEEGHSELQNKEKTSLLEELSLFMEKLNDVYYDDDDGLLNDFQVDTGTLPCVACGVLGFPFMSVVQPSEKALKDLSERQGETDAQEIMTLSSEKSDCEWKTSSRYIRPRIFCLEHTIELQRLLQSRGGLKFLVICHKDFQKFKAHAAIVAEEVKVPFSYDDVLLESASQEELSLIDLAIEDEEKYEHSVDWTSELGINLRYCVKVRKNSPTKKIQHALSLGGLFSDTSQMLDFTTIRWLQRKSRSKAKPSSTSSFTPCEHLEVKADGKLRDNLDSQTGKKEEKIIQYSRKKKLNPKPSAEQVQELATLAKSKDFDKTCKNFSSRSHLDSAIRSEMNSEIGDSGRVIGVSFSINPCSSSFTVGHGQEHPEITVKFGSDLDGNVTNSLSMVNGDSADLTLTSISREQHQGHSMTSNNNGSNSGSHVVASQTILVSTGDNHDGPRKLSGDYVCSDVSVRGIQEAVEMSDQEFGEPRSTVTNIEDEQQSQIVKPTQREAVFGDHEQVEGAEAVSTRENLCSEIILHTEHSSAHVGMEIPDINTASENLVVDMTHDGEPLESSDILSSSNGDEASSNGLQVLNDELSMESEVSSSENTEVIEAPNSMGEAKKKRKIESESETNDNPESSIGFIRSPCEGLRSRGKRKATCETSLKHTETSDEEKKPIAKRLKKTPKACSGSRQQEVPTTTHPNRCYLEGCKMTFESKAKLQTHKRNRCTHEGCGKKFRAHKYLVLHQRVHKDERPFECSWKGCSMTFKWQWARTEHLRLHTGERPYICKVDGCGLSFRFVSDYSRHRRKTMHYVT

>AtJMJ12/REF6

MAVSEQSQDVFPWLKSLPVAPEFRPTLAEFQDPIAYILKIEEEASRYGICKILPPLPPPSKKTSISNLNRSLAARAAARVRDGGFGACDYDGGPTFATRQQQIGFCPRKQRPVQRPVWQSGEEYSFGEFEFKAKNFEKNYLKKCGKKSQLSALEIETLYWRATVDKPFSVEYANDMPGSAFIPLSLAAARRRESGGEGGTVGETAWNMRAMSRAEGSLLKFMKEEIPGVTSPMVYVAMMFSWFAWHVEDHDLHSLNYLHMGAGKTWYGVPKDAALAFEEVVRVHGYGEELNPLVTFSTLGEKTTVMSPEVFVKAGIPCCRLVQNPGEFVVTFPGAYHSGFSHGFNFGEASNIATPEWLRMAKDAAIRRAAINYPPMVSHLQLLYDFVLALGSRVPTSINPKPRSSRLKDKARSEGERLTKKLFVQNIIHNNELLSSLGKGSPVALLPQSSSDISVCSDLRIGSHLITNQENPIQLKCEDLSSDSVVVDLSNGLKDTVSVKEKFTSLCERSRNHLASTEKDTQETLSDAERRKNDAAVALSDQRLFSCVTCGVLSFDCVAIVQPKEAAARYLMSADCSFFNDWTAASGSANLGQAARSLHPQSKEKHDVNYFYNVPVQTMDHSVKTGDQKTSTTSPTIAHKDNDVLGMLASAYGDSSDSEEEDQKGLVTPSSKGETKTYDQEGSDGHEEARDGRTSDFNCQRLTSEQNGLSKGGKSSLLEIALPFIPRSDDDSCRLHVFCLEHAAEVEQQLRPFGGINLMLLCHPEYPRIEAEAKIVAEELVINHEWNDTEFRNVTREDEETIQAALDNVEAKGGNSDWTVKLGVNLSYSAILSRSPLYSKQMPYNSIIYKAFGRSSPVASSPSKPKVSGKRSSRQRKYVVGKWCGKVWMSHQVHPFLLEQDLEGEESERSCHLRVAMDEDATGKRSFPNNVSRDSTTMFGRKYCRKRKIRAKAVPRKKLTSFKREDGVSDDTSEDHSYKQQWRASGNEEESYFETGNTASGDSSNQMSDPHKGIIRHKGYKEFESDDEVSDRSLGEEYTVRACAASESSMENGSQHSMYDHDDDDDDIDRQPRGIPRSQQTRVFRNPVSYESEDNGVYQQSGRISISNRQANRMVGEYDSAENSLEERGFCSTGKRQTRSTAKRIAKTKTVQSSRDTKGRFLQEFASGKKNEELDSYMEGPSTRLRVRHQKPSRGSLETKPKKIGKKRSGNASFSRVATEKDVEEKEEEEEEEENEEEECAAYQCNMEGCTMSFSSEKQLMLHKRNICPIKGCGKNFFSHKYLVQHQRVHSDDRPLKCPWKGCKMTFKWAWSRTEHIRVHTGARPYVCAEPDCGQTFRFVSDFSRHKRKTGHSVKKTNKR

>AtJMJ13

MAERRICLSKEAKDGLEFLKRKKLQKMRSDSVNETVGFSTMARSGGDALRPTSASCGMRLRVTSSDTVSKVHGASTVRGGLMKEKVEKLETDDLKWTERLPECPVYRPTKEEFEDPLTYLQKIFPEASKYGICKIVSPLTATVPAGAVLMKEKSNFKFTTRVQPLRLAEWDSDDKVTFFMSGRTYTFRDYEKMANKVFARRYCSGGSLPDSFLEKEFWKEIACGKTETVEYACDVDGSAFSSAPGDPLGSSKWNLNKVSRLPKSTLRLLETSIPGVTEPMLYIGMLFSMFAWHVEDHYLYSINYQHCGASKTWYGIPGSAALKFEKVVKECVYNDDILSTNGEDGAFDVLLGKTTIFPPKTLLDHNVPVYKAVQKPGEFVVTFPRAYHAGFSHGFNCGEAVNFAMGDWFPFGAIASCRYAHLNRVPLLPHEELICKEAMLLNSSSKSENLDLTPTELSGQRSIKTAFVHLIRFLHLARWSLMKSGLCTGLVSNTYGTIVCSLCKRDCYLAFINCECYSHPVCLRHDVKKLDLPCGTTHTLYLRDNIEDMEAAAMKFEKEDGVSDLITTDEDLYKYPSSITLPAAKEDGYTPYSTIYFDFYTEVEMTSHDQLQSGNPVMSYEANASCISSVADDYECSDYVNRRANCSSSSDSKLSEEVACSSSKKTRFFPVVQDEQLVADQESDGSDSECFRVKRRSSLKFENRTVVLDTRESDHHQELKRLKKSHHHEGRYSSSSSVSRQEEEEDELVISNRKETQQQSDVKMQKKRIENHFGGFKRLKVKGLIKP

>OsJMJ701

MPPQPPPAASASASAPDPAVPAWLRGLPRAPEYRPTESEFADPIAFLSRVEREAAAYGIC

KVIPPHPRPSRRFVFAHLNRSLVSSCDAPAPSPAAASDSSIPPSSSSPPPVSAAVFTTRH

QELGNPRRGRPTPQVLKQVWQSGERYTLDQFESKSRAFSKTHLAGLHEPTALAVESLFWK

ASADRPIYIEYANDVPGSGFAAPVQLQRKKKQKRETAPMDEWEKSSGWRLSNSPWNLQAI

ARAPGSLTRFMPDDVPGVTSPMVYIGMLFSWFAWHVEDHDLHSLNFLHTGAPKTWYAVPG

DRAVELEEVIRVHGYGGNTDRIASLAVLGEKTTLMSPEVLIDNDWCNILVSLW

>OsJMJ702

MSLQPPAVEPPEWLRTLPVAPEYHPTLAEFADPIAYILRIEPEASRYGICKIVPPLPRPP

EDDTFRRLQAAFAAAASSNGDPSPTFPTRLQQVGLSARNRRAASRRVWESGERYTLEAFR

AKAAEFEPPRHAAPPRNPTHLQLEALFWAACASRPFSVEYGNDMPGSGFASPDELPDAAN

ATDVGETEWNMRVAPRARGSLLRAMARDVAGVTTPMLYVAMLYSWFAWHVEDHELHSLNF

LHFGKAKTWYGVPRDAMLAFEETVRVHGYADDLNAIMAFQTLNEKTTVLSPEVLLSAGVP

CCRLVQKAGEFVITFPGAYHSGFSHGFNCGEASNIATPHWLQVAKEAAIRRASTNCGPMV

SHYQLLYELALSLRPREPKNFYSVPRSSRLRDKNKNEGDIMVKENFVGSVTENNNLLSAL

LDKNSCIIVPNADFFVPSFPVALESEVTVKQRFTAGPCSISQQGAENMAADHVAVDKVTE

IQDMSGSLYPCETSLVGCSNRKLYETKYGQRDAAALCLSTSEIQSRGIDTARSHPAGGIL

DQGRLPCVQCGILSFACVAIIQPREAAVQFIMSKECISSSAKQGGIGASDDTSNWIDQSH

EISPPPGPASGTDDNVKHAVSLAHVSDRCRELYASNTDGCTSALGLLASAYDSSDSDDET

TEDVSKHSKKNDSVNQSTDPQILETSASCSSTVQCQKTNSHLHEEECEARATSLMKPVSH

NSRPISQSNRDTDIDHFIELGKSGTQCSGYLDLVDDLTTSVLKSSSDTCVSAAKASMDPD

VLTMLRYNKDSCRMHVFCLEHALETWTQLQQIGGANIMLLCHPEYPRAESAAKVIAEELG

IKHDWKDITFKEATEEDVKKIQLALQDEDAEPTGSDWAVKMGINIYYSAKQSKSPLYSKQ

IPYNSIIYKAFGQENPDSLTDYGCQKSGSTKKKVAGWWCGKVWMSNQVHPLLAREREEQN

SSVVYGKAMFTTISHGKVQDEASTRCNTSNRTPSRRTSRRKKGVSAEKSKPKNKRSTASD

EASMLCSGLGMNSGVIHDQTENSDDYDKHGNGDEIEEGTNPQKYQQRKLQNVTRKSSSKK

RKDEKRTDSFHELYDEDNGVDYWLNMGSGDDATLGNSRQQSPDPVKVKSGGKLQGKRKSS

KYKSNDDLLNEENKLQKMNKKSSSKKQKNDKINRQLQEDQTEDDHMDHLVDVAVADEVTL

DNEDKITEDKIDDVKVKSRGKSQNGKRKGSKHQATDGLRAGNKVAKFPCDIEGCDMSFST

QQDLLLHKRDICPVKGCKKKFFCHKYLLQHRKVHIDERPLKCTWKGCKKAFKWPWARTEH

MRVHTGVRPYECQEPGCGQTFRFVSDFSRHKRKTGHSSDKRRKNST

>OsJMJ705

MRPSPPPAAPAAEPVPPWLRSLPVAPEFRPTAAEFADPVSYILKIEPAAAPYGICKVVPP

LPPPPKKATFSNLSRSFAALHPDDRSPSFPTRHQQVGLCPRRTRPGLKPVWRSSHRYTLP

QFESKAGATRKSLLAGLNFPASRQLTPLDHEVLFWRASADRPIVVEYGSDMSGSGFSPCA

AQPQPPPQQQPTARAAAHLGETAWNMRGVARSPGSLLRFMPEDVPGVTTPMLYVGMMFSW

FAWHVEDHDLHSLNYMHLGAAKTWYGVPRDAALAFEDVVREHGYGGEVNPLETFATLGQK

TTVMSPEVLVESGIPCCRLVQNAGEFVVTFPGSYHCGFSHGFNCGEASNIATPEWLRIAK

EAAIRRASINRPPMVSHYQLLYDLALSMRFREPSNGEMETRSSRIKEKKKCEGEQLVKKM

FIQNVIEDNELLSHLLNDGSSCIILPANAHDGPGLSTLRSTDQSNMNSRISHNLCSREEA

PEASGCLSPNRNGDTRNCISSDTHNMEGDKGDIMSATGLLDQGLLSCVTCGILSFSCVAV

LKPRDSTARYLMSADSNSINNQLSISGGSILADAPTNERNGVISRPYSEHCCNEIMADDA

EIDKNSALDLLAFAHGGQPDPEEDPLEKILKIAHGINKSQPNSSNNVGCVGTKLSSSSTE

RQERPSSQNAHCNGSSVISNGPKGVRTRNKYQLKMVLSEGFQAKDIYSAKEKKVQSEPSS

SKGDVKETIDVSGTENDVGCKSTTISVSEHRGSTKNMYSVKEKKVQSKPSSLKGTVKETV

DVSGTENDARCKSITISVSEHRGSTPMTNSLAASIVKPDKDSSRMHVFCLEHAIEVEKQL

HAIGGSNIMLICRPEYPKIEAEARLLGEEMGLVYDWKGIHFKEANMEDRQKIQEVLRDEE

AIPTSSDWAVKLGINLYYSANLAKSPLYNKQMPYNRVIYRAFGCDSPNDSPVMFNTCERK

QSHQKKIVVAGRWCGKVWMSKQVHPYLAHRVESQEAEEADRICSYHFDEKHKAEPVGNSS

RVEASKRKSSSLTDVTESSNRRGEIPGEETNTKRPKHSQENNLRALETAAEVVVPSPAGT

GLRVSSRIANRANKLKSKMEKEDVPSSRPKSNIKEKSSHASGQKSNVQEANANSASHLRA

MPPKQKAEAEAKKQIRTPKPPKQAVEYSCDIEGCSMSFRTKRDLSLHKSDICPVKGCGKK

FFSHKYLLQHRKVHTDDRPLTCPWKGCNMAFKWPWARTEHLRVHTGDRPYVCHEPGCAQT

FRFVSDFSRHKRKTGHSVKKKKKAKS

>OsJMJ706

MQQVEGRNCLPAEVRIGLETLKRRRLERMRLTAQNNAGDGPPVPARSGGDALRTPANCGV

RLHANNGTALPSRTTQNKDPFAKRRVDKFDMSSLEWIDKIEECPVYYPTKEEFEDPIGYI

QKIAPVASKYGICKIVSPVSASVPAGVVLMKEQPGFKFMTRVQPLRLAKWAEDDTVTFFM

SERKYTFRDYEKMANKVFAKKYSSASCLPAKYVEEEFWREIAFGKMDFVEYACDVDGSAF

SSSPHDQLGKSNWNLKNFSRLSNSVLRLLQTPIPGVTDPMLYIGMLFSMFAWHVEDHYLY

SINYHHCGAFKTWYGIPGDAAPGFEKVASQFVYNKDILVGEGEDAAFDVLLGKTTMFPPN

VLLDHNVPVYKAVQKPGEFVITFPRSYHAGFSHGFNCGEAVNFAISDWFPLGSVASRRYA

LLNRTPLLAHEELLCRSAVLLSHKLLNSDPKSLNKSEHPHSQRCLKSCFVQLMRFQRNTR

GLLAKMGSQIHYKPKTYPNLSCSMCRRDCYITHVLCGCNFDPVCLHHEQELRSCPCKSNQ

VVYVREDIQELEALSRKFEKDICLDKEISGFDSYKQAEKNEPFFEITRNLRNTEVNLIED

AFSGATAADAAKSSPATSTLTSFAQHDVPVLAEAIVCANQADQLYSTTEQTISSPLVKGT

DAVGANSSSMADANNGTGSCNASAVEYSGNSDSESEIFRVKRRSGVSVKPASDAKTSNLS

DQQVLRRLKKVRPEIQQHNKRPEDYGHCSVPSGRMSMKNLNSSSSCGEEHWRMKRRQLET

QQDESSYSAKQKSYSYPSTSYSFRGEFVEMSRDAAAEVRPKRLKIRLPSSSTNRVVEQGS

SGQRFTRDDKSLGCWPAI

>OsJMJ707

MAGNDEVALKPVSCGARLRRSCDASLRFGGSMRDPFLKHKVKKFDLSSLDWIDEIPECPV

FSPSIEEFEDPLVYLNKIAPIAAKYGICKIVSPLCASVPIGPVLMKEQGGLKFTTRVQPL

RLAEWSKDDKFAFFMSGRKYTFRDFEKMANKEFVRRYSSAACLPPRYMEEEFWHEIAFGK

MQSVEYACDIDGSAFSSSPNDQLGTSKWNLKRLSRLPKSTLRLLRAAIPGITDPMLYIGM

LFSMFAWHVEDHYLYSINYHHCGASKTWYGIPGKAAPDFEKVVCEHVYDHEILSGEGENA

AFDVILGKTTMFPPNILLRHHVPVYRAIQKPGEFVITFPRAYHSGFSHGFNCGEAVNFAI

GEWFPLGALASQRYALLKRTPLLPYEELLCKEAALLDHEFSTCDYKDTTTLAGETHSQRC

MKVPFVQLMRVQHRIRWSLMKMGARTHYKADIDATVLCGICRRDCYVAHIMCNCRIDAIC

LCHEEEIRRCPCSCDRVVFVRKDIFELETLSKKFEEESGILDAVKKQMARHDGTSQHSNF

FDCTDHEAEYYPYCNIHIDPSPEIYSISETNFVGYDLNNPHPAASTVTFSFVPHEYSTQS

DECTSSNRRALSSSCLENTITPENAISNACQLSTPDQTCLSDKLAAHDTDDSDCEIFRVK

RRSGLTPEKRHMEDGTTNFTGNQVLKRLKKINAHDRQEHKLPELSCGARSEPVHTDDCIH

CVDFISENGDDFIAPTKLKMIHQLDANIVEDEVASSQKYNSCNYQSPSIELGPKRLKIRG

PSFPSRISELEVSCRFQDNDLGSQHAR

>AtJMJ17

MYGNDLDTSVYGSGFPRIGDQRPESVEADIWDEYCGSPWNLNNMPKLKGSMLQAIRHNINGVTVPWLYLGMLFSSFCWHFEDHCFYSVNYLHWGEAKCWYGIPGSAASAFEKVMRKTLPDLFDAQPDLLFQLVTMLSPTVLQENKVPVYTVLQEPGNFVITFPKSFHAGFNFGLNCAEAVNFATADWLPYGGSGAELYRLYRKPSVISHEELLCVVAKGNCCNNEGSIHLKKELLRIYSKEKTWREQLWKSGILRSSPMFVPECADSVGIEEDPTCIICQQFLHLSAIVCNCRPSVFACLEHWKHLCECEPTKLRLEYRYTLAELDMMVQEVEKFGGCKTQETKISQRPSSGTKRSIALNKKQEGMQVSQARPADKWLLRASKVLDAAFSSVEYATLLKESEQFLWAGSEMDRVRDVTKSLNKAKIWAEAVSDCLSKVEGEVNDDSMKVHLEFIDELLRVNPVPCFNSGYLKLKDYAEEARKLSEKIDSALSSSPTITQLELLHSEVSRSPISLKKHEILSKKISSAKMLAKRAKRYLTDAKPPGIEMDALFKLNSEMLELHVQLPETEGILDLVKKSESARDKSNKVLTGSLSLENVEELLHEFDSFSINVPELNILRQYHVDTLSWISRFNDVMVDVREGKDQRKLISDLSSLLRDGASLGIQVEGLPLVEVELKKASCREKARTVYTARKSLDFIEQLLSEAVILHIEEEEIFVEISGILSTARCWEERASTILENETQMYELKDLVRMSVNIDAVLPTLQGIENTISSAETWLQKSEPFLSATSSMASSPCSMLELPVLKDLVTQAKLLNVQLQEPRILETLLLNCERWQCDNHQLLQETEDLLDNAKIDDGTHSNILPKIMDLITRVDSARRSGLALGLNFDELPKLRTASLKLGWCCKTITLSSSSPTSELLEDVGKPSLQHIQQHLKEGQTLEILPEEYYLGKRLMELKDTGLEWAKRARKVVTDSGALALEDVFELISEGENLPVHAEQELQSLRARSMLHCICLKPYNSRSMVSCSQCGEWYHTYCLKLHWRPKAYVCSACCPLAETTPQIDPARATEPERPSLNQRRTRMVATDAAVNDLKWKTRKHIKRTTKRSPQVHILPWFFT

>OsJMJ708

MDNACMLYSDCNQKGLCAICSCDVGDHITPRCMICQARYHSSCVEPLPASTQVTREWTCP

FCFHLESGDPLQNRLQEKISKGNRPALPALIGLRSFAKGFYSGIEELDLLEEIAEKAHKF

KSYLMQILHDADSYHGEDLSVMHRSLLIALKATSAAGLYDHQISCRIESMLSRYSWKKRI

HILLCGGKKIPIQQVLMLDNEGSSLEICGEDFFKLEINKIKETSLQWLAKAEKTTLDSGK

LALDLVYGLIIEGESLTVHVEKELKLLRDRSVLYCICRKPYDNRAMIACDQCDEWYHFDC

IKLHGPPPKTFYCPACRPNNGGEYISLPCLAHEDDRSTTEAGPHTPPASCEAAGRVGAIQ

CNSSSQWEKTHVRVDLIKLLRCHSETDSSWRESKRVLHRTARRRSNFLGL

>LcJMJ17

MGKGRTRVVSGQNLGVLSTVKSGLLNIPSGPVYYPSEEEFKDPLEYVYKIRPEAEPYGICK

IVPPKSWRPPFALDLDAFTFPTKTQAIHQLQARPAACDSKTFELEYGRFLEEHCGKKLKKK

VLFEGEELDLCKLFNAVKRFGGYDKVVKEKKWGEAFRRVADRANKRWFGSGSASRVHLEKK

FWEIVEGSAGRVEVMYGSDLDTSVYGSGFPRVNDQRPESITANVWDEYCASPWNLNNLPKL

KGSMLQTVHHNITGVMVPWLYVGMLFSAFCWHFEDHCFYSMNYHHWGEPKCWYSVPGSEAT

AFEKVMRNSLPDLFDAQPDLLFQLVTMLNPSVLQENGVPVYSVLQEPGNFVITFPRSYHAG

FNFGLNCAEAVNFAPADWLPHGGFGAELYQLYRKPAVLSHEELLCVVAKGDCDSRASPYLK

KELRRISSKERMWRERLWKKGIIRSRPLCPRKCPEYVGTEEDPTCIICRQYLYLSAVICRC

RPSAFVCLEIKGGRVSLSRLAEEWLQRSCKIFQNPFTSDAYVTLLKEAEQFLWAGSEMDPV

RHMVRNLLEAQRWAEGIRNCLSKVENWSNHHGCDVEKVRLGYLTELLGFDPVPCSEPGHLK

LKKYAEEASLLHQEINAALSKCSEISELELLYTRACGLPIYVEESDKLSQRISSAKVWIES

VRKCISGKCPAGNEIEILYTLKSEILDLQVQLPETEILLSLIRQAESCRAKCSQMLNGSIN

LKDVAALLQELDGFTINVPELMLLKQYHSDATSWIARFNNIMVNIHERRDQYNVIDELDCI

LKDGTSLRIRVDELPVVEVELKKAHCRAKAFKACDTKMSLDFIQHLMADAVVLQIDKEKLF

LDMSEVVDTAMHWEERARHTLACKAQMSEFEDIIRTSEDIFVVLPSLVDVQDAVSAAKSWL

KDSKPFLASAFSLEPFSGSLNLEALKELVSQSKFLKISLEEKGILENILQNCEKWQLNATS

LLQDVWCLLDTTGIGDRLSNGLMPKIEHLIMSMESAIKVGLSLGFHFHEIPELENACSTLR

WCNKALSFCSFAPSFQDVKSLMEVAESLSISCTSSILLNSLYGGVRWLRRASEVISAPSKF

KICKLSDVEEVLAGSQDISVSFPAMVGELTTAVQEHKRWQEQVFQFFNLKDEQRSWSLMLQ

LKEHGKAAAFQCSELERVVSEVDKVENWKQCCIDIVGASAGDIHPLLGALQKLKQSLDRSL

NIYDKSRGFSPINSCVCCASGSEGQESLTCSTCNDCYHLRCLRPAVDTNRPELYECHYCQL

LKCGSISKNGNGPLKFGGKCCDLRKLIELLSGSENFCVGIEERGVLQAIVDEALACKTFLT

DIVKVALCYMDKDLSVISSKLTTALKAIEVAGVYDHQSNRALDLALARTSWRLKVDKLLEG

STKPSIRQIQHRLTEGLAMNISPEDYFRQKLMELNQICLQWTDQAKKVATDSGSSSLDEVF

ELIADGETLPVSMEKELKLLRARSMLYCICRKPYDGRAMIACDRCDEWYHMACIGLRSSLK

IFICAACKPEAEELLSTSTVMDDGRSSPKFVEPKTPSPTHTKLRKKEKKVVPSLTQKMLAF

TNSSRMFNYSSGIDGLWWRNRKPFRRAARKRTVLENFSPFIHRQ

>SlJMJ6

MGRGRPRAVEKGVLGQNTSASPSGLLNIPPGPVYYPTEDEFKDPLEFIYKIRPEAEKYGICKIVPPKSWKPPYALDLNTFTFPTKTQAIHQLQARCASCDPKTFELEYNRFLEEHCGKKAKKRIVFEGEDLDLCKLYNFVKRFGGYDKVVKEKKWGEVFRFVRPAGKISECAKHVLFQLYLEHLYDYEEYYSKLNKLGHRSCRRGNQSERKRESDSPSSSSKRRRKNSEGDRTETRKTKEEEEHDQICEQCKSGLHGEVMLLCDRCNKGWHMFCLSPPLEQVPPGNWYCLQCLNSEKDSFGFAPGRELPLDAFRRIADRAKKRWFGSTSISQVQLEKKFWEIVEGSAGEVEVKYGSDLDTSIYGSGFPRLTDEKPSSVEPGTWDEYCASPWNLNNLPKLPGSMLRAVHHSIAGVMVPWLYIGMLFSSFCWHFEDHCFYSMNYHHWGEPKCWYSVPGSEAQAFEKVMRNSLPDLFDAQPDLLFQLVTMLNPRVLQENGVPVYKVLQEPGDFIITFPRSYHGGFNCGLNCAEAVNFAPADWLPHGGFGAELYQLYRKAAVLSHEELLCAVARSEFDSNAAPYLKTELVRVYSKEKSWRERLWKNGIVNSSPMPPRLKPEYVGTEEDPTCIICQQYLYLSAVACSCAPSSFVCLEHWEHLCECKPQKRRLLFRHTLAELNDMVLITDKSNHEEAAKKIRGQLLSSNDPSALSKKIKGGCITHMQLAEEWLIKSSKLFQNPYSSDAYRRAIKEAEQFMWADHEMDPVRDLVKRLIDAQSWAQNVRDSLSKVKSWMSDHNSVVKVQMEVVDNLLSLNPVPCNEPALVRLKDFQKEASELTLEIDSVLSSCSNILVSDLETLYSKTVDCPIYIKGSEELLCKLSSAKAWAERVRKCVSETSARVEADILYKLEKENLSLQVQLPEGEMLLDLIRQVECCQSQCCDMLKCSLSVKELESLLNKWDGFAVNIPELELLRRYHKDAVSWIKRVNNILLGISEREDQETVAHELTCIQKDASLLRVEVEELPCVDIELKKARCRVKALKALRCRTSMDYIEKLLMEASILQIEKEKLFTDVYEVKEIAVSLEERAKRVLENKEEISEFEDVIRASEEIFVILPSLDEVKDAVSMAKSWLSRSQPFLSRDSMTLGSSPSLEIDTLKILVSESKLLKLSLRELLMIQTLLDTCTRWEQDACSVLHDTECLLNGANTDDEILSRFGKIEKQIQAIESVVEAGQGLGFKFDMVPKLEDACSTLRWCFRALSFATAIPTLEEVKTNLEIATHLPIMYTTCSLCISLLDWVNWLNRALEVSILSTAGRSNLSDAEEVLRQYQNICVSSPAMISQLQKAIEKHNSWMDQVHSFFVLNFRDRSWDLLLQLKEKGNNDAFSCSELDMVFSEVHKTDEWKRRCEEVLHPSIRDANLLAALLQTKNALERSINICEKSNQTNASALCIFCSHDGVNQKLLTCSTCNDSFHLKCIGWSPGDANDSKVFICPYCHFMNSGKISRNGSDPLNIGRKSFKLHKLVELLSDAEDLCLWIQERAVLHQIGQKALDFKARIEEIVKFVLAYLDEDLSIIAKKFCVALKAVHIVGAYDSEANSKLELALARTSWKIRAQRLLDGSQKPSIQVLQRHLKEGLAVGIPSEDYFRQSLIEVKNLGLQWADIAKKVSTDGGALGLDKVFELITEGENLPMSCEKELKLLRDRSMLYCICRRPYDQRPMIACDKCDEWYHFDCIKLSSLPKIYICPACCCMEGEDFASMSTSGEEKVVGGKHEVPQTPSPRHRESRRRSRKTKWERTDVAADISRSSSNIEQLFWKNRKPYRRVARKRSHFESLSPFIFVQNS

>OsJMJ704

MVSSRDPGEEASAPPPPPPRRGEKRRMRGRTPSPEPASAPQDLCPSGACGDNVAGATTTN

GKWHPHESYRPEIDDAPVFTPTEEEFKDPIRYITSIRPQAEKYGICRIVPPSSWRPPCSL

KEKNFWECTEFNTRVQQVDKLQNREPTKKKSQPRVQKKRKRRKRLRFGMTHRRPSANTSE

DCADADEKFGFQSGSDFTLDEFQKYADEFKQQYFGIKGSDEIPLSEIKKKKKNWQPSVDE

IEGEYWRIVVCPTDEVEVDYGADLDTSMFSSGFSKLSSDSNRRDPYGLSCWNLNNLPRIP

GSVLSFETEDISGVVVPWLYVGMCFSSFCWHVEDHFLYSMNYMHFGEPKVWYGVPGADAV

KLEEAMRKNLPRLFEEQPDLLHELVTQLSPSVLKSEGVPVYRVVQNPGEFVLTLPRAYHS

GFNCGFNCAEAVNVAPVDWLPHGQCAVELYREQRRKTSISHDKLLLKTANEAVRQLWMNL

SDCKSEQGVYRWQDTCGKDGMLTSAIKTRVKMEKAARGGNMALRYKKMDGDYDSADRECF

SCFYDLHLSAVSCQCSPNRFACLNHANILCSCEMDRKTALLRYTIEELHTLVAALEGDPT

AVYQWGQNDLGLVCPSGSTQYKKMDLGENTEFPDSATNVNHGCSLGSQDQYHYDPAKPAG

YQQEKGIQIASEKHDKNKMVVNLESPATASNPSRSKSDCSGSLSLNHSSELPSSRIQTGN

STLASITTEKLFGVDIKSNLAQSSDGQVSQLAKPSSSQTDEVSKPAIAKYTVELLDSGTM

MIGKKWCNQQAIFPKGFKSRVTFHSVLDPTRTCCYISEVLDAGLLGPLFRVTVEGLPEVS

FTHTSPMQCWDSVRDRVNEEIAKQISFGKSGLPDFLSCNSLNGLEMFGFLSSPIIKEIEA

LDPCHQCLDYWLSRVSSVGTELPSESVMAAMVNDSTNPPIKLLGIEINRRESEQSSSFNN

SCVRRSHLAGC

AtJM>J15/MEE27/PKDM7C

MEPFSAAQNKEDKDTSVEPPRRRCHRKNKGTNVEPPSSPYHPKVLARWDPANEKRPDIGEAPVFHPTSEEFEDTLAYIEKIRPLAESFGICRIVPPSNWSPPCRLKGDSIWKNKNFPTRVQFVDLLQNRGPVKKKTPKGRKRKRGKYSRTVAPKKRNGSVSKSVSTPKATEEENFGFESGPEFTLEKFEKYAQDFKDSYFERKDNVGDPSVEEIEGEYWRIIEKETNEVKVLYGTDLENPILGSGFSKGVKIPTRRNDMDKYISSGWNLNNLARLQGSLLSFEDCEISGVQVPWLYVGMCFSTFCWHVEDNHLYSLNYHHFGEPKVWYGVPGSHATGLEKAMRKHLPDLFDEQPDLLHELVTQFSPTILKNEGVPVYRAVQNAGEYVLTFPRAYHSGFNCGFNCAEAVNVAPVDWLAHGQNAVEIYSQETRKTSLSHDKILLGAAFEAVKSLSAHGEDNTKRFSWKRFCGKDGIITKAIEARLRMEEKRIEALGNGFSLVKMDKDFDSNCERECISCFSDLHLSATGCKNCSSLEEYGCTKHDICSCEGKDRFIFLRYTIDELSSLVRALEGESDDLKAWLSKVMEGCSETQKGESSGIIVKEKQVQEECFDLNGECNKSSEICEDASIMDLAAYHVEPINLGFLVVGKLWCNKHAIFPKGFKSRVKFYNVQDPMRISYYVSEIVDAGLLGPLFKVTLEESQDESFSYASPQKCWEMVLLRVKEEIMRRSNQKQDVHMLESIDGLKMFGFRSPFIVQATEALDPNHGQVEYWNHKNEKDSLEMKDCFMSNSSQSLSKARLFGVDLN

>AtJMJ18

MENPPLESEIKEDMSLKNHPPDKDKDKDTIMEQPSSPRHRKVVARWLPDEAQRPIINDAPVFTPSLEEFVDPLAYIEKIRPLAEPYGICRIIPPSTWKPPCRLKEKSIWEQTKFPTRIQTVDLLQNREPMKKKPKSRKRKRRRNSRMGSSKRRSGSSPAESTSSPEAEEKFGFNSGSDFTLDEFEKYALHFKDSYFEKKDSGGDIVKWTPSVDDIEGEYWRIVEQPTDEVEVYYGADLENGVLGSGFYKRAEKFTGSDMEQYTLSGWNLNNLPRLPGSVLSFEDCDISGVLVPWLYVGMCFSSFCWHVEDHHLYSLNYHHFGEPKVWYGVPGSNATALEKAMRKHLPDLFEEQPDLLHGLVTQFSPSILKDEGVQAYRVVQNSGEYVLTFPRAYHAGFNCGFNCAEAVNVAPVDWLAHGQNAVELYSKETRKTSLSHDKLLLGAAYEAVKALWELSASEGKENTTNLRWKSFCGKNGTLTNAIQARLQMEEGRITALGRDSSSLKKMEKDFDSNCERECFSCFYDLHLSASGCKCSPEEYACLKHADDLCSCDVKDGFILLRYTMDELSSLVRALEGESDDLKIWASKVLGIEHSDEDQTKTSSVISEEKKLKEGSFDLNIDLEMDYQEDVKEEASTSGGELTASENLGVSVEPINLGFLIFGKLWCNKYAIFPKGFRSRVKFYNVLDPTRMSNYISEVLDAGLMGPLFRVTLEESPDESFFNVSAQQCWEMVMRRVKDTSTSLGLPILPQFESINGLQMFGFLSPSIVQAIEALDPNHRLVEYWNHKNQTSSDSKDHFISSNCSASLTKGKLFGVDLM

>AtJMJ1B/PKDM7B

MDQLASLAESVAMEEDSEKQSIKGESSLEPDSTPSSPKITARWNPSEACRPLVDDAPIFYPTNEDFDDPLGYIEKLRSKAESYGICRIVPPVAWRPPCPLKEKKIWENSKFPTRIQFIDLLQNREPIKKSTKTKKRKRRRISKIGYTRRKRDSGCDTASSGSSDSEGKFGFQTGPDFTLEEFQKYDEYFKECYFQSEDHPGSKASENKKFKPKVKDLEGEYWRIVEQATDEVEVYYGADLETKKFGSGFPKYKPGYPISEADQYSQCGWNLNNLSRLPGSVLAFESCDISGVIVPWLYVGMCFSTFCWHVEDHHLYSMNYLHTGDPKVWYGIPGNHAESFENVMKKRLPDLFEEQPDLLHQLVTQLSPRILKEEGVPVYRAVQRSGEFILTFPKAYHSGFNCGFNCAEAVNVAPVDWLVHGQNAVEGYSKQRRKSSLSHDKLLLGAAMEATYCLWELSLSKKKTPVIARWKRVCSEDGLLTKAVKKRVQMEEERLNHLQDGFSLRKMEGDFDNKRERECFLCFYDLHMSASSCKCSPNRFACLIHAKDLCSCESKDRYILIRHTLDELWALVRALEGDLDAIDLWASKCRDQYPSQHPRAREYAYLKSAPCIKSRGSSKVQQREQNNLQLVSERLQSDLTSNKEVQLKQDGDSDVNRHGHESERNHVHGITDKSAVTDVKLGVGGKFDEKKISVESQNPHSVSDVGCSELAKKVDGCLGGKDQNAATNRLSLSVELLSSGSLVVKKLWCSKQAIYPKGFKSRVKFLSVLDPTNLTNYISEVLDAGLLGPLFRVSVEDYPTENFSNVSAEKCWQMVTQRLKLEIIKKCDQPVSSLTSLQPLESINGLEMFGFLSPHVIKVVEALDPKHQLEEYWNQKAVKLFGAEPIKEGEKDDTEKGGASDPSLDRDTRLLRGLLKKATPEELVMMHGLLCGETRNTELKEELSTLVDKMEISP

>LcJMJ14

MEQFKLVAQSHDKEDHSLKRSSNSDNTIEFSGSPQNGKVSARWYPTEACRPIIDEAPVFYPTVEEFEDTLGYIAKIRSEAESFGICRIVPPSSWIPPCPLKAKDIWGNAKFSTRSQQVELLQNREPMKRKNRSRKRKRRRQSRMGSTRRRASSGSQANTASETDEKFGFLTGPDFTLEEFQKYADSFKECYFGMKDSKEYLKSDGFEHKRWEPTVEDIEGEYWRIVEQSTDEVEVYYGADLETGAFGSGFPKASSMVAERDSHPYLTSGWNLNNLPRLPGSVLCFEGCDISGVLVPWLYVGMCFSSFCWHVEDHHLYSLNYLHWGDPKVWYGIPGSHACTLENTMRKHLPDLFEEQPDLLHELVTQLSPSVLKAEGVPVYRAVQHSGEFVLTFPRAYHSGFNCGFNCAEAVNVAPVDWLAHGQQAVELYSKQHRKTSLSHDKLLFGSAQAAMLAHWELLMLQRETPGNLSWKSVCGKDGLLAKALKTRVQMEKERIGQLPSGLKLQKMEKDFDLNCERECFSCFYDLHSAAAGCKCSPDRFACLKHADDFCSCEIDNRFVLLRYTVDELNTLIEALEGGIEALRIWASENFGFSLSSKTDCSGAKVDLESELSPMDCGEQKRSLYSSPRREEIVDVNDPCCSHSHVSSEVVELDSQHGTLSLSGSHASIESHNDFKDETHVMIKETMADRRGCIDLNIYVTSDDRECKLQHVPDACDKEAIADVKIPVSMCSQEKVYSLGAVKEPDKKRLRSDCNSHEFLNKGQPSCIEDTGFFGSKKLFGIDLILQHSHANAPSDHFLKTEIMSSPEMGVPVTDQTYQGEQMDHCVEAVNYGSVVFGKLWCSKRAIFPKGFRSRVNFYSLLNPEQVCSYISEVLDAGLLGPRFKVTLEDCPSESFVNVSAEKCWEMVLQRLKQEILRRSTLGERRLPPLQSLQNIDGLELFGFLSPPIIQAIEALDPNHQCTEYWNHKHNTSEVNKGSFGLSCSPGDTKMKGCDVALTEEDENNPYAGGNNSVDEEVEVVLRGLLKEASPKELKIMHRILCSEAQSAERRLALRTIEEMHKTCR

>LcJMJ15

MEKDFDSNSERECFSCFYDLHPAAAGCKCSPDRFACLKHANDFCSCEIDNRFVLLHYTIDELNTLIEAFEGGVKALRIWASANIGFSPRGKTDCSGTKVDLESELSPMDCGEQKRSCYPSPRREEIMDVNDPCCSHSHVSSEVVQLASQRRTLSLSGSHASIESHNDFNDETYVMIKEAKSQHVPDACDKEAIADVKIPMSMGNQEKVHSLGAVKEPDKKRLRSDYNSPEFLNKGQPSCAGLIEDTGSRSRVNFYCLLDAEKVCSYISEVLDAGLLRPLLKALDLNHQCTGYWNHKHNTGEINKDSFGLSCSQRETKMRGCGTAMTEQDENNPHVGGNIIQLPKR

>SlJMJ8

MKGHPSRQAPKGENNIECSGSSRRRKESAIWSPGEARRPILEEAPVYYPNDEEFKDPLGYIASIRHNAQKYGICKIIPPASWSPPCPLREKNVWECAKFSTRIQQVDLLQNREPMKKKKTRKRKRRLHSKTGSTRRQPRSLGSESNTHSDSSDDKFGFQSGSDFTFEEFQTFSKDFKELYFRMKDTEVWKPSIEEIEGEYWRIVENPTDEVEVLYGADLETGVFGSGFPLESSSPKSSTLDQYATSGWNLNNLPRLPCSVLCFEENNISGVLVPWLYIGMCFSSFCWHVEDHHLYSLNYMHWGEPKIWYGVPGSHAAALEDAMRKHLPDLFEEQPDLLHELVTQLSPSVLKSEGVPVYRAVQNAGEFVLTFPRAYHSGFNCGFNCAEAVNLGPVDWLEHGLTAVELYSKQCRKTSLSHDKLLIGAASEAIRALWELSAVENINSINLRWRSFCGKDGMLTKAIKGRIEMEEERLKRLLPLVQLQTMDKDFGLKDEQECFSCFYDLHLSAVKCQCSPGQFSCLKHSNLMCSCEPENKTVLVRYNRDELNTLVQALEGKLDAIEQWTSKDPDNFSLNRRQHNSVKQDSERDGFETDPSMKNDSLSGLLREQTHNPKKQCSSCSDDATTSYASNHSSGKKLFGVDLSRGSPSVQQNGTFDSEIDPLSTKVSERTLLYHVDPLKLGSIASGKLWCSKQAIFPIGFRSRVKFFDASSPEITSSYICEILNGGLIGPLFKVSLEECPDTNLVSSSAQKCWEMISHRVFEELATKLNPGRQDLPPLQPDTECINGLGMFGLLSPPIVQSIEALDTNYQCLEYWKNKLKLKDECVTVKGPSGSSESTVDMARSAVMERGQCSGTKVATEEEHANYSSSNTELQLVLRRLLNKADPEELRIMHKILCSGSTSPEWRIAFATLSQEILRKV

>AtJMJ19

MGIEGVSTYLKSGNMDTISAPPGFVSQTSFVLRNVPRDKESPRSVSRQEQTTGFGTDDKDSCNMFLKSRPWIVHGHTIPSSEALRPKKTEVRRRRPLKVSETKVLEEAPVFNPTEEEFRDTLSYISSLRDRAEPYGICCVVPPPSWKPPCLLKEKQIWEASTFFPQVQLFGIQTENRKIKKEVDADSNDAASEGVQLCRVERGPGYTLKSFKNFADTYKKSHFGMKDEVLGSENSSPSLKPNELIVADIEKEYRQIVESPLIEIGVLYGNDLDTATFGSGFPLSAPSESSKYSSGWNLNSTAKLPGSLLSLEDCESVCVPRLSVGMCLSSQFWKSEKERLYSLCYLHVGAPRVWYSVAGCHRSKFKAAMKSFILEMSGEQPKKSHNPVMMMSPYQLSVEGIPVTRCVQHPGQYVIIFPGSYYSAFDCGFNCLEKANFAPLDWLPHGDIAVQVNQEMSKTSLISYDKLLFSAAREAVKCLKEYGLSKKNTACYTRWNDSCGTDGLFSNIIKSRIKLEKNRREFLISSLESQRMDKSYDAVNKRECCVCLGDLYLSAVNCSCSANRYSCLNHMRKLCACPCDRKSFLYRYTMDELNLLVEALEGKKLSSMFRWAGIDQKFCASPATTSSKPEEDKGKETDEVTPCNITRKDVAAGTKDQTRVKARSLADILNVKDGNNDAKETLESCSKKSNRPCDNDSSEANAPKKQKQ

>SlJMJ16

MGAKRIRLQGECDDRKRFSVPPGFESLTSFTLQKVENNEEACNSVAVGNESEQGPVQVASTATIISTGKLKSSVRRRPWILDDHVDHMEENFECESDKGSSSHAYLPRGVIRGCSSCHNCQKVIARSRPESARIPSLDEAPVLHPTEEEFKDTLKYVASILPHVKQYGVCRIVPPSSWRPPCRIEEEDTRCGVNTRIQRISDLQSLFLKMRLEGAHKKTNNRRQKIPSMKPEFGHSVERKEFGCCDEHFEFESGPKFKLKSFKNYADHFKRQYFVKEDQITASNFNSDAMQMLSEPSIPDIEGEYWRIIENPTEEIEVLHGNTTETSASQSGFPLKTNPRDVTACPEYVESGWNLNNTPKLQDSLLRFESCNSSSILLPRLSFGMCFSSNQWRIEEHHLYLLSYIHFGAPRIFYGVPGSHRCKFEEAVKKHLPPLSAHPCLLHNLATQFSPSILTSEGIPVYRCVQNPKEFVLILPGAYHAEFDSGFNCYEAVNFSPFDWLPHGQNAVELYREQDRKTSISHDKLLLEAAAEAIRTLGELALRNNNSFDDSKWRTVCRNYGYLTKALKTRVATEARRRKYLFASLESRKMEDDFCATTKQECVACFCDLYLSAIGCKCSAHKYTCLLHAKQLCDCAWSERYLLIRYEIDELNIMVEDLDRKVSAVHNRAKEKLGLPVSDVSKDAINEVGMETMKHKPVIPNVELSESTSHRSTSRQASDIQQYRNVDVFFAPSVVPSSTTMNLNHRSQLKENVHDENKVLLPKVSQNTAVGENIATSSSTVLKKHLAQGSSSTVRDVIILSDDED

>OsJMJ703

MMGVTTTLNEDTEPSIPPGFGPFATLPLWGIHNDAKPAVTHSTPVQALQSIRKDSEECQP

SAAVSRSDTPCSTSGTQTCRKSLRNRPPIDYSRFEHISDEDSDVEIVEKDVSSTRRRQQL

PKGVLRGCAECSDCQKVIAKWNPAGARRPVLDEAPVFYPTEEEFEDTLKYIESIRPMAEP

YGICRIVPPSSWKPPCLLKDKSIWEGSKFSTRVQKVDKLQNRKSSKKGRRGGMMKRRKLA

ESEENSATAHTQTGMQQSPERFGFEPGPEFTLQTFQKYADDFSKQYFRKDTSMDSVPSVE

DIEGEYWRIVEVPTEEIEVIYGADLETGTFGSGFPKLSPETKSDAEDKYAQSGWNLNNLP

RLQGSVLSFEGGDISGVLVPWVYVGMCFSSFCWHVEDHHLYSLNYMHWGAPKLWYGVPGK

DAVNLESAMRKHLPELFEEQPDLLHNLVTQFSPSLLKSEGVHVYRCVQHEGEFVLTFPRA

YHAGFNCGFNCAEAVNVAPIDWLPIGHNAVELYREQARKITISHDKLLLGAAREAIRAQW

DILFLKRNTADNMRWKSICGADSTIFKALKARIETELVQRKTLGVPAQSRKMDAEFDSID

RECALCYYDLHLSASGCPCCPEKYACLVHAKQLCSCDWDKRFFLFRYDVNELNILADALG

GKLSAIHRWGVSDLGLSLSSCVKREKVQDSKTVRRLTDGPRRSYMSQASAVSLVSSSTSN

EQKDEGNKIMKIASPQTNNVCPSVEQRKSENISPLKEPCVRNELSCTTNSDSNGLQYNGG

LGGHKGSAPGLPVSSSPSFSSNVATRPISTSSVSMKIVQGLVASKSCIQASSRTGDSRSL

LGEHHNRSPAMIHDGTNMKSSLESSNNSCRLIASDYNATPCHSSKDQVLVTPGTNASVVT

LKDSSQVHSASSQQFVRTGPWTQSASHEASSPSTSALKPSLDPPAMKNLYGGFTQGSAHP

GPPSFSNQQPNDGRLQRTSESLPGVEARARGHPTVTAQPALEIHSRNGGAQKGPRIANVV

HRFKCSVEPLEIGVVLSGRLWSSSQAIFPKGFRSRVKYFSIVDPIQMAYYISEILDAGMQ

GPLFMVKLENCPGEVFINLSPTKCWNMVRERLNMEIRRQLNMGKSNLPTLQPPGSVDGLE

MFGLLSPPIVQAIWARDRDHICTEYWRSRPHVLIEDPNNRHMLSQGPPLLALRGLIQRAN

RDELQVLRSLMTNSNNLDDSSRQQAAHIIEEEIAKQLC

>SlJMJ7

MDISAIPPGFESLAPFTLKQVENNRLRINQSSTASESKSHRSQVETNIEGNEDVKMMKSLRRKPGVNYGKYEKSSEDESGSDQNPSVRSSLPKGVIRGCEGCLNCQRVTARWRPEEASRPDLGDAPVFYPTEKEFEDTLTYMASIRSKAETYGICRIVPPVSWKPPCPLEEKNVWEKSKFATRIQRIDKLQNRDSMRRMWEANIHKKKKRRRCLKPGVDLGNGSVDNRNLGDAERFGFEPGPEFTLEAFQKYADDFKAQYFRQNEGQCPSLENIEGEYWRMVEKPTEEIEVLYGADLETGVFGSGFPKHDHQVGSSDTKYLNSGWNLNNFPRLTGSVLTYESSDISGVLVPWLYIGMCFSSFCWHVEDHHLYSLNYMHFGAPKMWYGVPGADASKLEAAMRKHLPDLFEEQPDLLHKLVTQLSPSILKSEGVPVYRCVQNPGEFVLTFPRAYHAGFNCGFNCAEAVNVAPVDWLPHGQNAIEHYREQGRKTSISHDKLLLGAARDAVKAHWELNLLRKNTSNNLRWKDVCGKDGVLSKALKNRVEMERVRREFLCNSSQALKMESTFDATNERECSVCFFDLHLSAAGCHNCSPDKYACLNHAKQLCTCSWGAKFFLFRYDINELNVLVDALEGKLSAIYRWARQDLGLALSSYVNKERQVAGIAGKLSLKPEESVLKEASAGPSIASVKKEKDDGTSALLMKASSSAFSPHKDKLSREPLASESIKASSMPDNASHGIEGAQNGFQGRSESLKVGPVYRTPVTQLSVEGGLCHKKLSTDKREVKGTSSLNDVVILLSDDEGDEMDNSIPSKDTAGKQTVNMGNNDKPVPTTSIDSARVTKDGINCSPSSESMKVEDNSKDEIHRGPNQDTHSFIGGSSVNMDIDRHAQAPQVADTCPQSRQPFDCKPNKEGSQNKTMECAQPLSGDSPVSQNNLDRGFRQKGPRIAKVVRRLACNVEPLDYGVIQPGKLWCDNRVIYPKGFRSRVRYIDVLDPTNMSHYISEVIDAGRDGPLFMVTLERCPNEVFVHLSPVKCWDMVRERVNQEILKQHKLGKPKLLPLQPPGSVEGMEMFGFSTTEIVQAIQDMDINRVCSEFWKSKPLMQTVQSSLVVDRSKLNIKSEISNDPTRADIVLSGLLKKANCEELHALNNLLKTNNLTPNQGLMTRLLNEEIDKRGR

>LcJMJ16

MGTELMRVCIKEDNDEIPSVPPGFESYTSFTLNRVQENEKNESDVKSSCSATVSASESQPVQIKTETGVSDAAKTTRSLRRRPCINYGQYENSSEDENDSGKLGQNFTLMPQLPKGVIRGCPGCGDCQKVIERWRPDNACRPELVDAPVFYPTEEEFEDTLKYIASIRPKAEPYGICRIVPPSSWKPPCPLKGKHIWENSTFATRVQRVDKLQNRHSMRKMSRVQNPTRRKRRRCMRMAVDCGTDSGNILGSGDSGCHEAERFGFEPGPEFTLNKFQKYADFFKDQYFSRNMNDVKDMGSNKAMLQEPWEPSVDNIEGEYWRIVEKATAEIEVLYGADLETGVFGSGFPKSPIQLNSALDERYIKSGWNLNNFPRLPGSLLAYESGDISGVLHVEDHHLYSLNYMHWGAPKIWYGVPGKDALKLEEAMKKYLPALFEEQPDLLHKLVTQLSPSILKSEGVPVYRCVQKAGEFILTFPRAYHSGFNCGFNCAEAVNVAPVDWLPHGQIAIELYREQQRKTSISHDKLLLGAAREAVKAHWELNLLKKNTVDNLKWKDFCGKDGILAKALKTRVEMERKKREYLCSSLQALKMASNFDATTERECNLCLFDLHLSAVGCRCSLDRYACLNHAKHLCPCAWGAKFFLFRYDISELNILVEALEGKLSAVYRWAKLDLGLALSSYVDSKKIDNLSFPLDRTVVKDMKPQPSDNSIEDLQGKEISGDFSLNLNVVASETSFQQKNSQSEAVLPLKDRKASSTSHRSFHDVEIKNHNVKSRKEEPVCSSSNLSIPVCQSSQEDMSYDLDSTEEKCVLKKPLNLEKANVILLSDDEDDEPEKPVSERAPANSAQHAESSERLGYSEEKARSSNHNKGAITNAAMSSRRDLSSSADAQKDNCSSHVQLKGAHEPAGGTLLRSSLPDLCSHGGTAREESGSIIADSSNNRDPSDHIMTNVEGNLQHPPCDGGISNNENNHEKVGTTSTSHFASNMRTTTGSASSAQNNLDRYFRQKGPRMAKVVRRINCSVEPLEYGVVLSGKLWCNSKAIFPKGFKSRVRYISVLDPTNMCYYFSEILDAGRDEPLFMVSLENCSSEVFVHVSAARCWELVRDRVNQEITRQHKLGRINLPPLQPPGSLDGFEMFGFSSPAIVQAIEAMDRNRVCTEYWDSRPYSRPQIQISQPSQNGENSQKLSGEQTNQESSGSRLLPVGVDTMIRSLFKKATPTELNSLYSILTDNKPTADRSLVTRLLNEEIHSRPV

>AtJMJ16/PKDM7D

MGTELMRICVKEDSDDLPSVPPGFESYATFTLKRVVPATTSDKAKTPAIESVSATEQAKMEVESDEAKAARALRRRPWINHSGCDDDGDCAANNDNAASQNPDQNCDVKPALPKGVVRGCEECKDCQKVTARWHPDEARRPDLEDAPVFYPSEEEFEDTLNYIAKIRPEAEKYGICRIVPPPSWKPPCPLKEKQVWEGSKFTTRVQRVDKLQNRSSMKKISKLPNQMRKKKRKCMKMGMDSVTNGMGDPCSASTGMNELETFGFEPGPGFTLKDFQKYADEFKAQYFKKSETSTDDKCKVDNSIDCWEPALEDVEGEYWRIVDKATEEIEVLYGADLETGVFGSGFPKISSSHNASSSEDKYAKSGWNLNNFPRLPGSLLKYEGSDISGVLVPWLYIGMCFSSFCWHVEDHHLYSLNYMHWGAPKLWYGVGGKDAVKLEEAMRKHLPDLFEEQPDLLHKLVTQLSPSKLKTAGVPVHRCVQHAGEFVLTFPRAYHAGFNSGFNCAEAVNVAPVDWLPHGQIAIELYCQQGRKTSISHDKLLLGAAREVVKADWELNLLRKNTVDNLRWKAFSAKDGILAKTLKARIDMERTRREFLCNSSLALKMHSNFDATNERECCICFFDLHLSAAGCRCSPEKYSCLTHVKELCSCPWVTKYFLFRYDIDELNVLVEAVEGKLSSVYRWARQDLGLALSTDVSGSKMEIDEEGKVHKDPTPQTTALSGKDLQLKVTSKEVSKELEKTSKLSHVNLLLKEKEEQITSSHCMKPVKEETVCDSSDPNVSACQPSEGGIICMTAVKSASGKKNSQSLPNDVILLSDDEYDIPRKRGSVRRDAISSGKKLEIRERPTHVLALEASAKIAAPICQREGDSLRDTRNTISLPTNDQKTMRRDVPSSTSHAEVNAEATGLTQDICNRMATNSHGGGKPTSCKSKNSGGLAIVDVVDGTRSSSGTPSCSQNNSPDRFIRQKGPRIAKVVRRINCNVEPLSYGCVLSGKSWCSRRAIFPKGFRSRVKYINILDPTNMCFYISEILDAGRNSPLFMVYLESNPSEVFVHMSPTRCWEMVRERVNQEITKQHKAGKSDLPPLQPSGSPDGFEMFGYSSPAIVQAIEALDVNRVCTDYWDSRPYSRPQVQFPANPLLREANTSGRSNVGNLQLNPGHHISPTGINSILKVLFKKASMEELSSLQEVLSETNSDMVTELVKEEIQNRR

>OsJMJ713

MRAEDDDAGGGEFVGARLDAGLRAARFASPPSADEFAAAVEPRNAPAVFRGVVKDWTASS

RWDPRRGGLDYLRVWIDPSFYLYLAHMRRQWERYLVSVPFYFALCNLRYSIYFRKRLDLM

LMWKL

>SlJMJ18

MPKLGSRPRLVHTHDIDGGALWDIFRRQDIPKLEEYLRKHFKEFRHINCCRIPKVIHPIHDQTFYLTKDHKNKLKEECGIEPWTFVKKLGDAVFIPAGCPYQVRNLKPK

>LcJMJ10

MKEVEKLWEEVRELSLGNNSSIECLDSPPSPLQFLRQFVSQNKPCVIKNAIEHWPALSLWAQPQLSYLSETLSSSLVSLHLTPNGRADSVVKHPSLDLSCFASAHVARLPFDQALRLVLSNSTDSNVVAYLQQQNDCFRSEYSVLGGDCDDHIPWATHALGCCPEAVNLWIGNHFSETSFHKDHYENLYAVVSGQKHFLLLPPTDVHRMYIRQYPAAHYSYSQDVGEFTLELDEPTTYVPWCSVNPYPSPETRESEMAKFPLFFNGPKPFLCTVNAGDILYLPSMWFHHVRQSPDENGLTIAVNYWYDMQFDIKYAYFNFLQSIHYKSPHDSALSEIGCGDLGCDRSVHNLRDEVQADTLALNDTDANKDNEDE

>OsJMJ714

MERAVRELWAESRDLLGLHSPDDAAAADAAMPRAEMPPTPLAFLRDHVSPGRPLLVSSAA

TSHWPAASLWPTDSYLTDALRSTAVSLHLTPDGRADALAPHPRPSHPGAKCFASAHVRQV

DFPTAVRLIRSSDPASGLVAYAQQQDDCLRGEYAAVAGDVDAHVPWASDALGCLPEAVNL

WIGSACSQTSFHKDHYDNIYVVVSGEKHFLLLPPTEHHRLYVRDYPAAHYAAEDEAELRL

KLELEEPERIVPWSSVDPYPPSPEEAAAQASSFPLYFEGPRPIRCTVRAGEMLYLPSMWF

HHVSQSPGPNGLTIAVNYWYDMQFDIKYAYFNFLRSLEIDGSSSKKTDALEDDLEETND

>SlJMJ10

MVEENLRIHTFTEIPSPEIFSSQIEPKNVPAVFKRCIKDWKAFSKWNPSDGGLIYLQERVGSVAVEAMLSRSGPVFYGDIRSHERVPLSFSTFIRYCLGLLKNRDGRRDDFLESQKHSLAVSDTEQTDLHFEEAPQQFYLAQVPILNFEKKEHMQLECLQEDIQTPVPLETKSLSSVNLWMNSMKARSSTHYDPHHNLLCIVSGCKEVTLWPPSATPYLYPLPLYGEASNHSSVTLEEPDLSLCPRATCLSDFSQKVVLHAGDALFIPEGWFHQVDSEVLTIAVNFWWRSMTISGMLEHMDAYYLRRILKRMTDKEMNKMLQFPSSSMDKTITCTTSQPSNAYRDHVHQGISTNCGYRSSKDELKSKVMLQDLEPCASQSLSELISLVHNRLNPSKLTESTDNSSAGENDETNKRKEDSCSTSNDPVANLILTLHPLRIHSVFLAMANHFPRTLEALVLHALTPVGSEILTRKFEEMDQLISGDDQNQFYQIFYGVFDDQSAAMDVLLNGKELFARQAFENVLGQYLGNNPDGPKQQTK

>AtJMJ31

MPTGCEFPMIKTFENALTAADFESTVELTNFPAVFRGCASVWDAYSKWNPFNSGLDYLEERAGSVEVEAMLSRTAPVFNGDIRSHERVSLPFSDFIRFCKQHMRGKGNGSGVDAKSADLNPMCEDYRPGQIYLAQFPILNDEKEEKVLLKILRQDIQTPTFLDAKSLSSINFWMNSAEARSSTHYDPHHNLLCVVSGRKKVVLWPPSASPSLYPMPIYGEASNHSSVGLENPNLSDYPRAEHSLKQSQEITLNAGDAVFIPEGWFHQVDSDELTVAVNFWWQSNYMSNMPEHMDSYYLRRITRSLLVSKPSSTDLRHLSEHIDQSRIEMAEGGNDNIGNESIKKGLSTLHEKASLHDLDPSASQALHDLISLVHDHVNAVDTSKGLQHTSPSCSEGGEKSKFLVNAMSCLEDDRVAHLLWNLEASRLRDVLLAMALELSYLKLLVKMEIFVLVLHKIFETLEALILHMLSPIAAEVLTQKFDEIDQQTGEEDRTQFFREFYSAFDDEAAAMDIILSRKEAFAFQVCSLASLCRLRTYHKLKGEKFSASY

>AtJMJ20

MGIQIIGQIERINGKELSYGDFAERYLAKNQPVVISDLTEDWRAREDWVSENGNPNLHVFATHFGKSRVQVADCDTREFTDQKRLEMSVTEFVEQWTNKDSIEESVLYLKDWHFVKEYPDYTAYQTPPLFSDDWLNVYLDNYQMHEDRDSFQKYDQISCSDYRFVYMGGKGSWTPLHADVFRSYSWSANVCGKKRWLFLPPPQSHLVYDRYMKNCVYDIFEEVNETKFPGFKKTTWLECIQEPGEIIFVPSGWHHQVYNLEDTISINHNWLNAYNLSWVWDLLWKDYKDTEESIEDIRDICDDFEAICQRNLAANTGMNLNDFFLFMSRFSLGNMVLLQSYSDKHKNLNSCSLAMAQNLLMNLSTILKVMMKMISAGGVTAEEVYLDLRETLEDPQFLRFVRDMGRTYARIHMEEEDQFLSSKELLQKLSGLAGPNMQICSPKDLVEMINHHNTFSSQIYFI

>LcJMJ3

MVHLPTPILDEESPRLLQAISEHGGYAYVGMVTQAAAGDIRAAEAAREMAWEQLHSGPWHSVLPVWRDAYSLACLHVAKYHSGKGERGEALRVLDMGLIMGGSLLRNDLDSAIQTLSDSDSDSHSQNPPSNNTKLVSSDNNFDKAESLRVLPVRSLSSKLVVKRSALSLEAFLQDYFLSASPVIITDCMGHWPATLKWNDIDYLKRVAGHRTVPVEINELRNDICIPDHCYAGGGELRSLNAWFGPAGTVTPLHHDPHHNILAQVVGRKYIRLYPASLSEELHPYSETMLSNSSQIQAIVPYLKVDLDHIDETKFPNVPDLDFLDCILDEGEMLYIPPKWWHYVRSLTISFSVSFWWSESGSSTES

>AtJMJ30

MSGATTASSGDHNNLRLPTPTLDAESQTLLQSISAEGGYAYARMAVLAVAGDQSAAEAARDMAWEQLHSGPWHSVLPVWRDAYSMACLHVAKIHFAAGEFGEALGALDMGLIMGGMLLRKDLHDSVLLVSSEARKMTKSLEEASGDFKGERLVPEVPVDVNEVRHVLANLQLLVLKILPCRSLTCKRVEKRSGLSLEGFLRDYYLPGTPVVITNSMAHWPARTKWNHLDYLNAVAGNRTVPVEVGKNYLCSDWKQELVTFSKFLERMRTNKSSPMEPTYLAQHPLFDQINELRDDICIPDYCFVGGGELQSLNAWFGPAGTVTPLHHDPHHNILAQVVGKKYIRLYPSFLQDELYPYSETMLCNSSQVDLDNIDETEFPKAMELEFMDCILEEGEMLYIPPKWWHYVRSLTMSLSVSFWWSNEAESSSS

>OsJMJ712

MAGGEPPAPATEAEEGRRAALLREITEEGGFAFVASAEKAACGDLRAAEAAREMAWEQLH

SGPWSEVGAAWRDAYALACLHVARLRRLGAAAADRRAALRALDMGLIMGGNLLRADLEAA

IARIVADPGGGGDAEAVDEETRRWREGLERNRDVADALNILPAKSLSCKKVERRSCISLE

EFICDYFLRESPVIISGSIDHWPARTKWKDIQYLKKIAGDRTVPVEVGKNYVCSEWKQEL

ITFSQFLERMWSAGCPSNLTYLAQHPLFEQIKELHEDIMVPDYCYAGGGELQSLNAWFGP

HGTVTPLHHDPHHNILAQVLGRKYIRLYPASISEDLYPHTETMLSNTSQVDLDNVDLKEF

PRVENLDFLDCILEEGDLLYIPPKWWHYVRSLSISFSVSFWWRSTVVPSKGS

>OsJMJ717

MLSCQIVHTNSNGENGSTPTPTTTQAPPSRSTLSLFSGTFQNDLPRENISHHKYLTTLDS

SSSLAYATDHLITPENISSSFALFLPAPMATGGEPRAAAAAEGGEKRAALLREITEEGGF

AFVASAEKAAADGDLRAAEAAREMAWEQLHACPRSEVGRAWRDAYALACLHVAALRVAGG

GGDGRRAALRALDMGLIMGGDLLRAELEEAIARVVADRSRGCGGGGGDGAGENGADVEKW

MEGLTRKRDLADVLKVLPVKSLSCKQIERRSCISLEAFIRDYFLCESPVILSGYIDHWPA

RTKWKDIRYLERIAGDRTVPVEVGKKYLSSEWKQELITFSQFLEMMWSSDCSANLTYLAQ

HPLFDQIKELREDIMVPEYCNAGGGELQKLNAWFGPEGTVTPLHHDLYHNLFAQVLGRKY

FRLYSASISNDLYPHRETMLSNISQVDLDNINVNEFPRTGDVEFMDGILEEGDLLYIPPK

WWHYVRSLSTSFSVSFWWRTSIPPPQGS

>LcJMJ20

MGVKIGGQIEKVNGKEMSYSEFEERYLAKNEPLVLSGLMDDWGASKDWVTEDGQPNLYFFSTHFGNSKVQVADCDTRDFTDQKRVEMSVSEFVKHWLENSAPENSDCSNNEANGTSVLYLKDWHFVKEYPEYLAYRTPQIFCDDWLNLYLDSFRMHTDPDTYQKDNEITCSDYRFVYMGAKGSWTPLHADVFRSYSWSANVCGKKKWLFLPPSQHHLVFDRHAHFYFASECIAVWSECIQEQNEIIFVPSGWYHQVHNLAVWSECIQEQNEIIFVPSGWYHQVHNLEDTISINHNWLNAYNLSWVWDLLLRDYKEAKELIEDIRDICDDFESLCQRNLAANTGMNFYDFFTFISRFALANVIQLFHLLSDHENSVRNSSPTAQHLVLNLVSIRKISLKMKAVGVPAADCGFFKDLQETLDDPTFLKLCTSLCHTYGMIRKQIKWTCDMKKALMVDFGDYGSLISTPEDLIKFIDFAAQKLGGTCSDLLSKFDTC

>SlJMJ11

MGLKIGGRIEKVNGRELTYSEFAEKYMSQNQPVLLTGLMDDWRACKDWVSPNGKPNLHFFSTHFGKSKVQVADCGTREFTDQKRIEMTVSEFVDRWLHDGGAGGGSLLYLKDWHFVKEYPEYIAYRTPMDFSDDWLNFYLDKFHMHNDPDTYSERNDITCSDYRFVYMGSKGTWTPLHADVFRSYSWSANVCGKKQWYFLSPSQQHLVFDRNMKSSVYDIFAEVSQSKFPGFEKAVWWECTQEENEVIFVPSGWYHQVHNLEDTISINHNWFNGYNLFWVITTRLVNTLTTSRDVMTLRSFANEILPLIQE

>LcJMJ1

MESFHGGFAMRDRRLDALGDLRVLPDEIICSILEQLTPRDLGRLACVSSVMYIFCNEEPLWMSQCLKRSIGQFQYKGSWKKTTLHFEEPPVEYKESCKRPLHFDGFYSTFLYRRVYRCHMSLSGFSFDDGNVERKKVLTREEFDRQCGGMEPVLLTELADTWPARTAWTIDQLLQKYGDTAFKISQKSAHKISMKFKDYVSYMNVQHDEDPLYIFDPKFGEAAPELLKDYNVPQLFQEDWFELLDEEDRPPYRWLIIGPERSGASWHVDPALTSAWNTLLCGRKRWALYPPGRVPLGVTVHVSDEDGDVHIETPSSLQWWLDFYPLLTDEEKPIECTQLPGETIFVPSGWWHCVLNLEATVAVTQNFVDSQNFPFVCLDFAPGYLHKGVCRAGMLALDEDSLEDVKKNISSDDYDFSYPDLGRNKKRLQTRKSGKNCNDEYATNGACKSYHLRRKGFPYDINILEKFLDEERDHYNFPWSPGNCIGQREMREWLSTLWVRKPGMRELIWKGACLTLNASKWLECLEAICAFHNFPPLTAEERLPIGSGSNPASSVWTWGAVKLFREGEKAIHMFVYGGRGIVGHVRTGKYNMRPKAELLEKVYLMADCVVKIFAENGLESSLYALGTELEFYGLLCKVNSPLKKYIPDVLASGILYLENGSYTIVPWGGKGVPDVIEKCNLITSKCAEDDFPFGIWNKKQFEYRRVGMNLGKLTTSDGSTRVWPYIITKRCKGKILGHLRDTLKWEDMLNLASLLGEQLHNLHLLCYPPFSESTLSGIEQEMDLTFTDCGMVDVLSKSNLPAEWEIFVRTLTRKKKDTARCLTNWFGPRSKPAGLAREGGDPIPKTLIEKVDEYIPDDFAKLIGIYKDENGLNKVCKPCSWIHSDIMDDNIHMESCCPCSCSCGVSADTDLRDNDIMNGYDHHAEKKTWRLSHILDFSNLSLGDPIYDLIPIYLDVFKGNSSLLKQFLERYKLPLVRGKPKEESGGDKFARLSYHAMCYCIMHDENVLGAIFSLWKDLRMAKSWEEVELTVWGELNNYEGFS

>AtJMJ21

MTTLGQRDRRPDALGSLSVLPDETICVLLEYLAPRDIAHLACVSSVMYILCNEEPLWMSLCLRRAKGPLEYKGSWKKTTLHLEGVTQENDAYRKCFHFDGFMSLYLYKRFYRCNTSLDGFSFDNGNVERRRNISLDEFSKEYDAKKPVLLSGLADSWPASNTWTIDQLSEKYGEVPFRISQRSPNKISMKFKDYIAYMKTQRDEDPLYVFDDKFGEAAPELLKDYSVPHLFQEDWFEILDKESRPPYRWLIVGPERSGASWHVDPALTSAWNTLLCGRKRWALYPPGKVPLGVTVHVNEDDGDVSIDTPSSLQWWLDYYPLLADEDKPIECTLLPGETIYVPSGWWHCILNLEPTVAVTQNFVNKENFGFVCLDMAPGYHHKGVCRAGLLALDDENSEDLEEETHDEEDNTLSYSDLTRKEKRTRMNGGGETENREEDVNGVSKRYNMWKNGFSYDIDFLASFLDKERDHYNFPWSMGNSVGQREMRAWLSKLWVLKPEMRELIWKGACIALNAEKWLRCLEEVCTFHNLPLVTEDEKLPVGTGSNPVYLLSDYAIKLFVEGGLEQSMYGLGTELEFYDILGRADSPLKTHIPEVLASGILFFEKGSYKVVPWDGKRIPDIISSSSFDFDASMLNSEFPFGIWNKTLREHKNQGKPAPDSFGSLSSHVWPYIITKRCKGKIFAQLRDDLTWNDAQNLAFFLGQQLRNLHLLPYPPVTRPELLNVNAVHEELNIPAEWKVFVDALCQKKKDVTSRLENWGNPIPRALMTKIDEYIPDDFFVDLLHVFKETNGGDEIKPCTWIHSDVMDDNIHMEPYADDSVDGQHNSWRPSHILDFSDLTIGDPICDLIPIYLDVFRGDADLLKKLLENYGLPLIRSRSSENGTTKTADSTRKKVLSPSYRTMCYCILHEENVLGSIFSIWDELRTAESWEQVEQTVWSLLNTY

>SlJMJ12

MRLESSPMEIDQIDRRPAALGDLRILPDEILCSILTYLTLRDVARLSCVSSVMYILCNEEPLWMSLCIDIADRQLQYKGSWKRTALDQLNVTFENKESCQKPLYFNGFNSLFLYRRLYRCHTSLNGFYYDSGNVERAKNLSVDEFRDKYDGHKPVLIGGLADTWPARTTWTTEELLKNYGDTAFKLSQRSRHKIRMKLKDYVAYMKVQHDEDPLYIFDEKFGEAAPELLKEYTVPNMFKEDFFDVLDMDQRPSFRWLIMGPERSGASWHVDPSLTSAWNTLLYGRKRWALYPPGRVPLGVTVHVNEEDGDVNIDSPSSLQWWLDFYPLLAEEDKPIECTQLPGETIFVPSGWWHCVLNLETTVAVTQNFVNSKNFEFVCLDMAPGYRHKGVVRAGLLALDDISIEDVRKNMLSLESGLSYSDLSRKDKRIRVDQPRSSEDGSTIDWVSKGINSTEVEFSYDINFLAMFLDKEQDHYTSLWSSSNSIGQREMREWLSKLWVEKPETRDLIWKGACLALNADRWYARATEICTFHGLPLPTDDERLPVGTGSNPVYLVGDNVIKILVEEGLEVCLHSLGTELEFYSSLQKMNSPLRNHIPNVLSSGILYIENGLCKVQCWDGKGIPEVIANFRPIVEHGEADYPFGLWSKRQLDYTKAGMSLAELVSTGSGTTIWPYVITQRCKGKIYAQIRDTISWEDTLNLASFLGEQMRNLHLVPCPALNDLTLLETQQKVVPTANGNLEDHEDKICVPAEWNLFLKTLNRKKKDVCDRLTKWGDPIPRELIEKVEEYIPDDLQKVDMGVRSCTWIHSDVMDDNIHMEPCSLPSRSGGTTDDPESIDNVSANGSNLSEPIRAWRPTHILDFSGLSVGDPIVDLIPIHLDIFRGDPHLLKQFLDSYQLPFIKTGVNASAKSNGFQRLSYRAMCYCILHDENVLGAIFSTWKKLKMAKSWEEVEEAVWGDLNSYTGSC

>OsJMJ711

MAAGNGRMEAALGCLAALPDEVLCAVVDLLPPTDVGRLACVSSVMYILCNEEPLWMSKCL

SVGGLLVYRGSWKKTALSRLNLCSENDEIYQKPRHFDGFNSMHLYRRWYRCFTNLSSFSF

DNGHVERKDDLSLDQFRAQYDGKCPVLLTKLAETWPARTKWTAQQLTHDYGEVPFRISQR

SPQKIKMKLKDYVFYMELQHDEDPLYIFDDKFGESAPTLLEDYSVPHLFQEDFFEIMDYD

QRPAFRWLIIGPERSGASWHVDPGLTSAWNTLLCGRKRWAMYPPGRVPGGVTVHVSDEDG

DVDIETPTSLQWWLDIYPNLAEHEKPLECTQLPGETIFVPSGWWHCVLNLDMTIAVTQNF

VNQSNFKHVCLDMAPGYCHKGVCRAGLLAAPDKSIRDIENLPSITSRLNHSDMACKEKRL

KSSEPIRTSNNANQCSAFEFSDVHENLGDQVFSYDIDFLSQFLEKEKDHYSSVWSPTNSI

GQREAREWLRRLWVLKPELRELIWKGACLAINVDKWYSCLEEISACHSLPPPSEDEKLPV

GTGSNPVFIVSGNVIKIYAEGGLGYSIHGLGTELEFYDLLQKLGSPLINHVPEIIASGFL

VYLDGVYKTVPWDGNGIPDVLAKYYSLEVSYANGSFPLGLWSKQLFGLSNSTDAPDRPIC

PYMVTRKCKGDIFARIRDKLTKTDVLNLASSLGVQMRNIHQLPLPHVEHISKSGNEDIKA

KENSISDVTHVPPEWKQVVSTLDRRKKSIKKHLSNWGGSIPQVLIEKAEEYLPDDIRFLI

KFVKDDDGDSVYVVPSWIHSDIMDDNILIEGTTEPGTSTDCIAVEDLNKMDAIHIIDFSD

LSIGDPLCDLIPLHLDVFRGDIDLLRQFLRSYQLPFLRAESNKDIYKSIQNSKFSRASYR

AMCYCILHEDNVLGAIFSLWKDLGTATSWEDVEHLVWGELNQYQQSCSVGEIN

>OsJMJ710

MPSAFHSLLLPAIRNPKPSRRRGRGRGGSKRPKKTTKSKNRLADAAAGDATAFHLKTSAR

AGPGGAGSGRRGDGGCLVQPLGNLLLLGGGGNLRDAGLGALRPLPDDVLLDVLGLLAARD

LARLSAASRALYVVASHDPLWRALVLDELGGDFAFSGSWRATYIAAASGGRAHLPPRGLE

IRGFYSDYLFQSWLCANMEMRPEWLHRDTIDRRRGMSVEQFVSEFEEPNRPVLLEGCLES

WPALQKWTREHLLKVSAGKEFAVGPVSMTLDRYLQYADNVQEERPLYLFDAKFTEKVPEM

GRDYEVPAYFREDLFGVLGEERPDHRWVIIGPAGSGSSFHVDPNSTSAWNAVIKGAKKWV

MFPPEVVPPGVHPSADGAEVTSPVSIMEWFMNFYGACKTWEKRPVECICRAGEVVFVPNG

WWHLVINLEESIAITQNYVSRRNLLNVLDFLKRPNASELVSGTTDRVNLHDKFRNAIDMT

YPGMIKQLELEAQQKAAARKKKVSFWESAVDANTGGFKFSF

>SlJMJ9

MLGSKSLLFKQQKRKRKNGKIKKSKRISVSAKEETVAEPCQIAPEEEEEEEEEGFSLKSTAQSDSYGVQPLGNLYFNPSSHNSRNTGLGNLQTLTDELVLDILGLLEGTHLGILSTVSKGFYIFCNHEPLWRNLVLETCKGGFLFKGCWRSTFISAYRPSFPVLSFGLKVRDFYSDYLFQSWLCANLEMKPEWLERDNIVRRKGISLDEFVMDFEEPNKPVLLEGCLENWPGLEKWNRDYLVKKCGDVKFSVGPVEMKLEDYFKYSDQVREERPLYLFDPKFAEKIPQLGKDYDVPMYFNEDLFSVLGNERPDYRWIIIGPAGSGSSFHIDPNSTSAWNAVTKGSKKWILFPPDVVPPGVHPSPDGAEVASPVSIIEWFMNFYNATKNWKKRPIECICKAGEVIFVPNGWWHLVINLEDSIAITQNFVSRRNLVNVLEFLKRPNACTLVSGTSDRVNLHDKFKNAIEAHLPGTIDELTLKAEEKKAQQNKPSFWESVTDSNAGVFRFSF

>LcJMJ2

MLGCRSLLSQVMQKTKKSKIKNKRRTRNKKSVSRKHESPPDQLQDIEEEEDTEEGFSLRASAQSDSHGVQPLGNLFFNPGSVNSRNTGLGSLQVLSDELVLDVLRFLDATHLGVLATVSKSFYVFSNHEPIWRNLVLDNLNGQFLFNGSWKSTYIATCYPSFDVQTVGVSGSLRIRDFYSDYLFQSWLCANLEMKPEWLERDNIIRKKGISVEEFVLNFEEPNKPVLLEGCLDSWVALKKWDRDYLVKVCGNVQFLVGPVEMRLEDYFRYSDLVKEERPLYLFDPKFAEKVPVLGSEYEVPVYFREDLFSFLGNERPDYRWVIIGPAGSGSSFHIDPNSTSAWNAVIKGSKKWVLFPPNVVPPGVHPSPDGAEVACPVSIMEWFMNFYGATKNWKKRPIECVCKAGEVIFVPNGWWHLVINLEESIAITQNYVSSIAYVLMILVCILKRVKEKFVEGKVHSFTMGDKAHPEIEKIERMWEEILRKIRLAALELLIS

>AtJMJ22

MPKCKNLLLTSKRRKSKSKRLKLHQHEPESLFPEKEVEEEDEDEGGFKLKIAAPSQEHGVQPLGNLYFNPGAVNVRNTGLGNLQILSDELVLDILGLLGANHLGVLATVTKSFYIFANHEPLWRNLVLEELKGDFLFNGSWRSTYVAAYHPKFKFAGDGESNLKIIDFYSDYLFQSWLCANLEMKPKWLRRDNITRVRGISVEDFITKFEEPNKPVLLEGCLDGWPAIEKWSRDYLTKVVGDVEFAVGPVEMKLEKYFRYSDGAREERPLYLFDPKFAEKVPVLDSEYDVPVYFREDLFGVLGNERPDYRWIIIGPAGSGSSFHIDPNSTSAWNAVITGSKKWVLFPPDVVPPGVHPSPDGAEVACPVSIIEWFMNFYDDTKDWEKKPIECICKAGEVMFVPNGWWHLVINLEESIAITQNYASRSNLLNVLEFLKKPNAKELVSGTTDRENLHDKFKKAIEEAYPGTIQELEKKAEEAKRAEEQRVSFWDSAKTDTFKFSF

>AtSDG5

MEKENHEDDGEGLPPELNQIKEQIEKERFLHIKRKFELRYIPSVATHASHHQSFDLNQPAAEDDNGGDNKSLLSRMQNPLRHFSASSDYNSYEDQGYVLDEDQDYALEEDVPLFLDEDVPLLPSVKLPIVEKLPRSITWVFTKSSQLMAESDSVIGKRQIYYLNGEALELSSEEDEEDEEEDEEEIKKEKCEFSEDVDRFIWTVGQDYGLDDLVVRRALAKYLEVDVSDILERYNELKLKNDGTAGEASDLTSKTITTAFQDFADRRHCRRCMIFDCHMHEKYEPESRSSEDKSSLFEDEDRQPCSEHCYLKVRSVTEADHVMDNDNSISNKIVVSDPNNTMWTPVEKDLYLKGIEIFGRNSCDVALNILRGLKTCLEIYNYMREQDQCTMSLDLNKTTQRHNQVTKKVSRKSSRSVRKKSRLRKYARYPPALKKTTSGEAKFYKHYTPCTCKSKCGQQCPCLTHENCCEKYCGCSKDCNNRFGGCNCAIGQCTNRQCPCFAANRECDPDLCRSCPLSCGDGTLGETPVQIQCKNMQFLLQTNKKILIGKSDVHGWGAFTWDSLKKNEYLGEYTGELITHDEANERGRIEDRIGSSYLFTLNDQLEIDARRKGNEFKFLNHSARPNCYAKLMIVRGDQRIGLFAERAIEEGEELFFDYCYGPEHADWSRGREPRKTGASKRSKEARPAR

>AtSDG10

MVTDDSNSSGRIKSHVDDDDDGEEEEDRLEGLENRLSELKRKIQGERVRSIKEKFEANRKKVDAHVSPFSSAASSRATAEDNGNSNMLSSRMRMPLCKLNGFSHGVGDRDYVPTKDVISASVKLPIAERIPPYTTWIFLDRNQRMAEDQSVVGRRQIYYEQHGGETLICSDSEEEPEPEEEKREFSEGEDSIIWLIGQEYGMGEEVQDALCQLLSVDASDILERYNELKLKDKQNTEEFSNSGFKLGISLEKGLGAALDSFDNLFCRRCLVFDCRLHGCSQPLISASEKQPYWSDYEGDRKPCSKHCYLQLKAVREVPETCSNFASKAEEKASEEECSKAVSSDVPHAAASGVSLQVEKTDIGIKNVDSSSGVEQEHGIRGKREVPILKDSNDLPNLSNKKQKTAASDTKMSFVNSVPSLDQALDSTKGDQGGTTDNKVNRDSEADAKEVGEPIPDNSVHDGGSSICQPHHGSGNGAIIIAEMSETSRPSTEWNPIEKDLYLKGVEIFGRNSCLIARNLLSGLKTCLDVSNYMRENEVSVFRRSSTPNLLLDDGRTDPGNDNDEVPPRTRLFRRKGKTRKLKYSTKSAGHPSVWKRIAGGKNQSCKQYTPCGCLSMCGKDCPCLTNETCCEKYCGCSKSCKNRFRGCHCAKSQCRSRQCPCFAAGRECDPDVCRNCWVSCGDGSLGEAPRRGEGQCGNMRLLLRQQQRILLGKSDVAGWGAFLKNSVSKNEYLGEYTGELISHHEADKRGKIYDRANSSFLFDLNDQYVLDAQRKGDKLKFANHSAKPNCYAKVMFVAGDHRVGIFANERIEASEELFYDYRYGPDQAPVWARKPEGSKKDDSAITHRRARKHQSH

>SlSDG22

MISSTSISAESAPTPTKFDGENEEDSSASLKYRINQLKRQIQTDRVLSVRDKLEENKRKLEIHVSELLMLATSRSDTMKNSGTGKMLSLRISSPLCKVVGLVQGSGDRDYANGEEVVSSVTARLPFIQNIPPYTTWIFLDKNQRMAEDQSVVGRRRIYYDQHGSEALICSDSEEDIAEPEEEKRHFSEGEDKILRMASREFGLNEEVLDILTQYVGGTTSEILEHCNVLEEKHQDTDGKSLKDSRESGFGGSMFLDKSLTAALDSFDNLFCRRCLVFDCRLHGCSQILIDAIEKQPYSSDSEDDRKPCGDRCYLKVKGVANQTKYSNVDPVEGLEKHTSEAGGSTMDIKRTRDPDEHIDSKMKHGVSDSINTTLEKSNLVLDDQQDSSGKRRKLSLPTAVSVAAEDGSESNGMSISTNDYVSHSQAPDQSGYNHGTSLHETGDNVSNEGEDTIKETVKHASYSKNLPEWKPLEKELYLKGIEIFGRNSCLIARNLLPGLKTCMEVSSYMDNRAAAQRGGSSSLFSEDNGKADMDYMELDIPTKSRFLRRRGRTRKLKYSSKSSGHPSIWRRMADGKNQSCIQYNPCGCQPMCGKHCPCLQNGTCCEKYCGCSKSCKNRFRGCHCAKSQCRSRQCPCFAAGRECDPDVCRNCWVSCGDGSLGEPPRQGEGQCGNMRLLLRQQQRILLSKSEVAGWGAFLKNPVYKNDYLGEYTGELISHREADKRGKIYDRANSSFLFDLNDQYVLDAYRKGDKLKFANHSSNPNCFAKVMLVAGDHRVGIFAKERIEASEELFYDYRYGPDQAPIWARKPEGTKRDDSPAPLGRPKKHQ

>LcSDG5

MPLCKYSGFPQGLGANDYDICHDVESLTSTELPFVEKIPPYTTWAFIDRNQKMFEHQSAVGSIHAYGQGQHKKEAPICNGSEEDNEEPEEEKHEFIEGEDRILWTILQEYGLGEQVLNIVSQFIGVAISDIKDRYSTLLKEKYHDKNCKAASGSGDSGYHERGISLETTLSAALDTFDSLFCRRCYVFDCRLHGCPPTSILASEKQPAWSESEDARKPCGDHCYLQVRADQDAVGGSTVNVPQRDIDTDLVLDERCNSGKVPSVTSKALYVSEVSTGASNTDTSFMQNMGKGKAVQHSTLVPYGIQHASKKRKKLYPWDIIRALSENRPSPVTKSRHHVGASNALDPQRTTKDSQTKSNESRKLVVSSSEWKPIEKELYLKGVEIFGRNSCLIARNLLSGLKTCIEVFNYMRDSAGGSSLTQKSVASSSFSEENGNANTDHTEQEIPKRRRLFRKRGKARKLKYTWRKSSRQPSIRKKAADDKTMSRAPRVAKTGSGDATVQRNNAEPSNAHALPLNVNVTQIFAAIAGCGDGTLGEPPKQGHSQCGNMKLLLRQKQRILLSKSEVAGWGAFLQNSVKKDDYLGEYAGEMVCYPEADERGTIEKCGPNSFLLVCLQYVLDAHRKGNKLKFANHSATPNCYAQAMMVAGDHRVGIYAKERIEAGEELFFDYMIGREQAPPWARKPGDSEAASSSAAEKHQSH

>LcSDG10

GESNDGLANLSNKLNQLKKQIQAERVVSIKKFHGELNDGLANLSNKLNQLKKQIQAERVVSIKEKLENNKTKIQIDVSQLLPETSRKNVVFMAGNEFSSVPLCKYSGIPQGLGDKEYVNCHEVVFSTSSKLPYVEKIPPYTTWIFLDRNQRMAEDQSVVGRRRIYYDQHGSEALICSDSEEDIVEPEEEKHEFTEGEDRILWTVFQEHGLGEEVLNSVSQFIGVATLEIQDRYSTLKEKYHDKNFKDSRDSGYERGISLEKSLSAALDSFDNLFCRRCLLFDCRLHGCSQNLINPSEKQPYWSEYEDDRKPCSDHCYLQLRADQDMLGGSTNNVPHKMNEAKEGTVVPHEIEEPCGDSGTVLVLDERCNSGKVPSVTSKAVYLSELSSGAPNTDTSSMQSLGKRKVVQESNIVLDDSIVPYDIQDSSKKQKKLLSLDVISASNDDISSPDAKSWHHVGASNEPDPQRTTKDSRSESIECISEKFVGSVSVTDDKFEHDTRDRSKDVEKEPELNQSSSKEKLREGVLSSSEWKPIEKELYLKGVEIFGRNSCLIARNLLSGLKTCIEVSTYMRDSGSSLPHKSVAPSLFLEENGKADTDYTEQEVATRPRLLRRRGRTRKLKYSWKSAGHPSIWKRIADGKNQSCKQYTPCGCQSMCGKQCPCLHNGTCCEKYCGCSKSCKNRFRGCHCAKSQCRSRQCPCFAAGRECDPDVCRNCWVSCGDGSLGEPPKRGDGQCGNMRLLLRQQQRILLSKSEVAGWGAFLKYVLDAYRKGDKLKFANHSSNPNCYAKVMLVAGDHRVGIFAKEHIEASQELFYDYRYGPDQAPVWARKPEGSKRDDSSISQGRAKKHQSH

>OsSDG718

MASSSSKASDSSSQRPKRPDQGPSGKDAAGLVALHGKLAQLKRQVQSTRLAAIKERVEAN

RKALQVHTCALFDVAAAAEVASRGAEGGNALSRGAAEGHRRFVGWDSASGPGERELVHVQ

EENLVAGTLVLSSSGGSGASHRTVVQLVKLPVVDKIPPYTTWIFLDKNQRMADDQSVGRR

RIYYDPIVNEALICSESDDDVPEPEEEKHVFTEGEDQLIWKATQDHGLSREVLNVLCQFV

DATPSEIEERSEVLFEKYEKQSQSSYKTDLQLFLDKTMDVALDSFDNLFCRRCLVFDCRL

HGCSQNLVFPSEKQPYGHELDENKRPCGDQCYLRRREVYQDTCNDDRNACTTYNMDSRSS

SLKVSATILSESEDSNRDEDNIKSTSIVETSRSKITNSEYADKSVTPPPGDASETENVSP

DMPLRTLGRRKISKHASKSNDHSPDKRQKIYSSPFPFAMSVLNKQSVPEIGETCPDSIES

AVDQLPSLDDPNKKISTKDMCAGSTTNTTENTLRDNNNNLFISNKEHSISHWSALERDLY

LKGIEIFGKNSCLIARNLLSGLKTCMEVASYMYNNGAAMAKRPLSGKSILGDFAEAEQGY

MEQDLVARTRICRRKGRARKLKYTWKSAGHPTVRKRIGDGKQWYTQYNPCGCQQMCGKDC

ACVENGTCCEKYCGCSKSCKNRFRGCHCAKSQCRSRQCPCFAASRECDPDVCRNCWVSCG

DGSLGEPLARGDGYQCGNMKLLLKQQQRILLGKSDVAGWGAFIKNPVNRNDYLGEYTGEL

ISHREADKRGKIYDRANSSFLFDLNEQYVLDAYRKGDKLKFANHSSNPNCYAKVMLVAGD

HRVGIYAKDRIEASEELFYDYRYGPDQAPAWARRPEGSKKDEASVSHHRAHKVAR

>AtSDG1

MASEASPSSSATRSEPPKDSPAEERGPASKEVSEVIESLKKKLAADRCISIKKRIDENKKNLFAITQSFMRSSMERGGSCKDGSDLLVKRQRDSPGMKSGIDESNNNRYVEDGPASSGMVQGSSVPVKISLRPIKMPDIKRLSPYTTWVFLDRNQRMTEDQSVVGRRRIYYDQTGGEALICSDSEEEAIDDEEEKRDFLEPEDYIIRMTLEQLGLSDSVLAELASFLSRSTSEIKARHGVLMKEKEVSESGDNQAESSLLNKDMEGALDSFDNLFCRRCLVFDCRLHGCSQDLIFPAEKPAPWCPPVDENLTCGANCYKTLLKSGRFPGYGTIEGKTGTSSDGAGTKTTPTKFSSKLNGRKPKTFPSESASSNEKCALETSDSENGLQQDTNSDKVSSSPKVKGSGRRVGRKRNKNRVAERVPRKTQKRQKKTEASDSDSIASGSCSPSDAKHKDNEDATSSSQKHVKSGNSGKSRKNGTPAEVSNNSVKDDVPVCQSNEVASELDAPGSDESLRKEEFMGETVSRGRLATNKLWRPLEKSLFDKGVEIFGMNSCLIARNLLSGFKSCWEVFQYMTCSENKASFFGGDGLNPDGSSKFDINGNMVNNQVRRRSRFLRRRGKVRRLKYTWKSAAYHSIRKRITEKKDQPCRQFNPCNCKIACGKECPCLLNGTCCEKYCGCPKSCKNRFRGCHCAKSQCRSRQCPCFAADRECDPDVCRNCWVIGGDGSLGVPSQRGDNYECRNMKLLLKQQQRVLLGISDVSGWGAFLKNSVSKHEYLGEYTGELISHKEADKRGKIYDRENCSFLFNLNDQFVLDAYRKGDKLKFANHSPEPNCYAKVIMVAGDHRVGIFAKERILAGEELFYDYRYEPDRAPAWAKKPEAPGSKKDENVTPSVGRPKKLA

>LcSDG1

MTIKEVGLSDAVLESLAQSFSRSPGEVKARYETLVKEENASGISKNGNNGVQTMNTFLQKDLEAALDSFDNLFCRRCLVFDCRLHGCSQDLVFPAEKQLPWYHSDVENVPCGPHCYRSVLKSERNANMGSPLNGNIEEKSLSSSDCAGAQTSSRKKSSGPVRRRLKSYQSESASSNTKNISESSDSEVGPKQETTSTRHLSPSKSKLVGKCGIRKRNSKRVAERVLVCMQKRQKKMAAIDSDSVVSGGPLPSDMKLRSTTRKENEDANSTSHKNVKSPTTGRTRKKELQILDSRSLALSEVPDGQSNEMNSDPPTTSSNGTLRKEEFVDENICKQEISDDKSWKAVEKGLFDKGVEIFGRNSCLIARNLLHGLKTCWEVFQYMTSSGNKLFGQAGDGANSLLEGYAKFDFNGNVVSIIFCLAKCIGQGSHFVLLDVPRAARIDLEAVIVLKVNAEVVSVHVLQQTGNVIQMFVGIVGCGDGTLGVPDQKGDNYECRNMKLLLKQQQRVLLGRSDVSGWGAFLKFVLDAYRKGDKLKFANHSPDPNCYAKVIMVAGDHRVGIFAKERISAGEELFYDYRYEPDRAPAWARKPEASGSKKEDGAPSSGRAKKLA

>OsSDG711

MAGDSRNEPMFCEEGSSESGYVLCVIDSLKKKITSDRFVYIQKRVEENSIKLSPITLHSH

NLSKNRQTSTSNSTDLVSNLLTKRKEDALCAVNSRESSPDESEGANCQDECSSTVIVGGN

LSARNSVRPIRLPEVATLPPYTTWIFLDRNQRMQEDQSVLGRRRIYYDTNCGEALICSDS

EDEAVEDEEEKKEFKDSEDCIIRMTIQECGMSDAVLETLARDIERAPDDIKARYEILQGE

KPEGSSKKVSELNVKMEDVYGDKDLDAALDSFDNLFCRRCLVFDCKLHGCSQDLVFPTEK

QAPLCSSDEGTPCGIHCYKLVSKPDAIMEIDSHLLVDVEEPTSDNLKDQIGSNKKKLGSS

GQKTKSQQSESSSTARVSSESSESEVQLLSNKSPQHSPGLSKNKLGAKGGIKKSTNRRIA

ERILMSVKKGQQEMSPDSNSIVNGCHWPRDMKLRSDTRSGIKDSVVSSQCNSPSTRSFRK

KGTLQMENNSSFVDAQSDSMEDTNNEHSATDGCDSSRKEECVDESICRQEAHGRSWKVIE

QGLLLKGLEIFGKNSCLIARNLLGGMKTCTDVFQYMNYIENSSASGALSGVDSLVKGYMK

GNELRTRSRFVRRRGRVRRLKYTWKTAGYHFIRKRITERKDQPCRQYTPCGCQSACGKQC

PCLTNGTCCEKYCGCPKMCKNRFRGCHCAKSQCRSRQCPCFAADRECDPDVCRNCWVGCG

DGTLGVPNQRGDNYECRNMKLLLKQQQRVLLGRSDVSGWGAFLKNSVGKHEYLGEYTGEL

ISHKEADKRGKIYDRENSSFLFNLNNEYVLDAYRMGDKLKFANHSPDPNCYAKVIMVAGD

HRVGIFAKERISAGEELFYDYRYEPDRAPAWARKPEGPGAKDDAQPSTGRAKKLAH

>SlSDG23

MEKTMSNHLKLLIILRCLVGNIGSCSLESQQPQCGRTSRREVSPVLGSKNYFQGEGFGCQYKEAACAKNGMNYDDTLRENEFGDENNCNQEIDGEKSWRPLEKALFEKGLEMFGRSSCMIARNLLNGLKTCWEVFQYMNNFENKLSLVSDGMNGIFQGSFKGDGHTIVGNQPQRKSRFLHRRGRVRRLKYTGKSAGYNALRKRISERKDKLRRHYNPCNCQVPCGKECPCIVNGTLCEKYCQCRSRQCPCFAVDRECDPDVCRNCWISCGDGTLDIPPQRSDSNDCENMKLLLKQRQRVLLGRSDVSGWGAFLKNSVGKHEYLGEYTGEIISHHEADRRGKIYDLINSSFLFNLDDQCVLDAYRKGDKLKFANHSPDPNCYPKVIMAGGDHKVGIFAKQRICAGEELFYDYCYAPDTPHVWARKPEAPTTRKFFLEDLMCLAGVISCRMVMESMRTLGSILVK

>SlSDG21

MSPASDNSLSDSQTQRLNDLSIVSPEEATVEPDEVLSVIESLKRKIASERADYIKKRVEGNTQKLENLTKDLYNLATERKCLEIFDAGGKIDLLSKRQKDALDMQNGIDTSNGDDDSNSSEDDGYATSAILLGSSIAVKNAVRPIKLPEVKRIPPYTSWIFLDRNQRMTEDQSVVGRRRIYYDQNGGETLICSDSDEEVLEEEEEKKVFAESEDYMLRMTIKEVGLSDIVLDLLGHCLSRKPSEVKARYEALVKADDVGTSKNEFTESSLDLYLAKDLDAALDSFDNLFCRRCLVFDCRLHGCSQDLIFPAEKQSPWYCSNADMEPCGPNCFSLAKKFESNATVISPQCASHGEKSILPSDVANNTQMPGRKHVSRRSKSSKGEGAPNAKNISESSDSDIRPVNDITSNERSSSPSKSKSDNKDGSNKRNSKRIAEHVLVAIKKRQKKMAVLESDTVASESLGFKDLNLHSISRKENEDASPSSQKAQCHSTKRSRRKNSPVLDSKNSLQGKAFGCKVMEVNSEKPVANCDDTLGKNEKVGENNCKQEVDGTKSWRPIEKALFEKGLEMFGRSSCLIARNLMNGLKTCWEVFQYMNNSGNKLFSGTGDGMDDILEGGCNGDGQEIMGEPRRRSRFLRRRGRVRRLKYTWKSTGYHAIRKRISERKDQPCRQFNPCGCQGPCGKECPCIVNGTCCEKYCGCPKGCKNRFRGCHCAKSQCRSRQCPCFAAGRECDPDVCRNCWISCGDGTLGVPPQRGDSHECRNMKLLLKQQQKVLLGRSDVSGWGAFLKNTVGKHEYLGEYTGELISHREADKRGKIYDRENSSFLFNLNDQFVLDAHRKGDKLKFANHSPVPNCYAKVMMVAGDHRVGIFANERICAGEELFYDYRYEPDSAPAWARKPEASGTRKEDAAPSSGRARKHT

>SlSDG35

MPAMKTAIHGGIGHVFSKLIKEIGDPVDFELPDWLNKWQSMPYTFIKRNIYLTKKVKRRLEDDGIFCSCSSTAETSVVCGKDCLCGIMLSSCSSGCKCGSSCLNKPFHQRPVKKMKLVKTEKCGSGIVADEDIKRGDFVIEYVGEVIDDKTCEERLWKMKHSGETNFYLCEINRDMVIDATYKGNKSRYINHSCCPNTEMQKWMIDGETRIGIFATRDIKRGEHLTYDYQFVQFGADQDCHCGAVRCRRKLGVKPNKPKLPASDTALKIVACQVAATSPKLKALLSTRHVYQTGVPRIGSSVYDSDIKIRRPRSCIGQVIRIIRSSKTRSFGIVKRFDAITKKHFVRKSCLKMAVFSTLTCQKKIGNSVTFLSNRVLAVRTRRCSRGCHCVQIRNIECPCCTKCISFILQLFSNNAAISDDISIKQKIETFFTM

>SlSDG15

MKTIYTLCVIEKTTLNNILSLPLFSIAFCCSEPFFSPAIAGDDDHFPPIKATVNECCYLIEPMPATPMKKSATRGGIGNVFNKLTSKIGDPVDFELPDWLSKWQPTPYPYTSIRRNIYLTNKGKRRLEDDGISCTCSSTAESSDVCGMDCLCSMLWSSCTSGCKCGSSCLNKPFHQRPVKKMKIVKTEKCGTGIVADEDIKTKEFVVEYVGEVIDDKTCEERLWKLKHSGETNFYLCEINRDMVIDATYKGNKSRYINHSCCPNTEMQRWMIDGENRIGIFATRDIKRGEHLTYDYQFVQFGADQDCHCGAINCKRKLGIRPNKLKLPSSDAAALKLVACQVAAPFPKEKVLLSAKHDSQTEVPPKGNWSSDSARKIQHPRNCTGQIIRIIRYSDQSPVDSLESRIPDVSSSFGIIKQFDRITKKHLIMFEDGSTEHLDLSKEDWRFCNFA

>LcSDG24

MPATKKNCDNGRIGLTFNKLLKQIGNPVEFQLPDWFIKWKPMPYTFIKRNLYLTKKIKRRLEDDGIFCSCAPSPGSSGVCDRDCHCGMLLSSCSSGCKCGDSCLNRPFQHRPVKKMKLVKTEKCGSGIVADEDIKKGEFVIEYVGEDQDCHCGAVGCRRKLGVKPSKPKMSSDAALKLVACQVAVSSPKLKALLSGKDPCPLLIDPLLQHYFEKLGLICVIHGQVYQNGGLHIVVGEGHGCIRKALGVIEVGWTAFGIIKRFDKYSGQLAVMFEDGGVEFLDMTKEAWELATL

>LcSDG7

MPYTFIKRNIYLTKKIKRRLEDDGIFCSCAPSPGSSGVCDRDCHCGMLLSSCSLGCKCGDSCLNRPFQHRPVKKMKLVKTEKCGSGIVAAEDIKKGEFVIEYVGEVIDDKTCEERLWKMKHRGETNFYLCEINRDMVIDATYKGNKSRYINHSCCPNTEMQKWIIDGETRIGIFATRDIKKGEHLTYDYQFVQFGADQDCHCGAVTCRQKLGVKPSKPKMSSDAALKLVACQVAVSSPKLKALLSGKDVYQNGGLHIGSSQPAHNQQEICPQNCIGKVIRIHQRPTKRAFGIIKRFDKYSGKLTVMFEDGGVEFLDMTKEAWELATL

>AtSDG24

MSSSKKGSDRNQIRKSLRKLKKQIGELEKLESPDRLNNVKPIFIKRNIYLKKKLKKKVKDHGIFCSCSLDPGSSTLCGSDCNCGILLSSCSSSCKCSSECTNKPFQQRHIKKMKLVQTEKCGYGIVADEDINSGEFIIEYVGEVIDDKICEERLWKLNHKVETNFYLCQINWNMVIDATHKGNKSRYINHSCSPNTEMQKWIIDGETRIGIFATRFINKGEQLTYDYQFVQFGADQDCYCGAVCCRKKLGAKPCKTKNTTLEEAVKPVACKVTWKTPKLLNSEVRETNLDASGQAWNNHSQRKICCRDCIGAYYTAQMKVLTLVVDIFQVMYEDGVTEIIDMCREVWKVVTA

>AtSDG7

MPASKKISDRNHLGQVFDKLLNQIGESEEFELPEWLNKGKPTPYIFIRRNIYLTKKVKRRVEDDGIFCSCSSSSPGSSSTVCGSNCHCGMLFSSCSSSCKCGSECNNKPFQQRHVKKMKLIQTEKCGSGIVAEEEIEAGEFIIEYVGEVIDDKTCEERLWKMKHRGETNFYLCEITRDMVIDATHKGNKSRYINHSCNPNTQMQKWIIDGETRIGIFATRGIKKGEHLTYDYQFVQFGADQDCHCGAVGCRRKLGVKPSKPKIASDEAFNLVAHELAQTLPKVHQNGLVNRHIDAGKSWNNLSQRDTCSRNCIGVVIRLSRPTSDRCFGLVRHFDEYSRKHSVMFEDGVTEFVDMSREDWEIV

>OsSDG724

MPRPAKIRKKHENVFDQLIKAIKAPVDFDLPPVLKEWKSNYYVPIKRNAYITRKRVEDDG

IFCSCTPSGSSATCDKDCQCGMLFSCCSSTCKCENKCANKPFQHRTLRKTKLIKTEKCGN

GVVAEEDIKKGEFVIEYVGEVIDDRTCEQRLWKMKRQGDTNFYLCEVSSNMVIDATNKGN

MSRFINHSCEPNTEMQKWTVEGETRVGIFALRDIKTGEELTYDYKFVQFGADQDCHCGSS

NCRKMLGITKPVNSIVLHNGNLSQDQHVRKKRKTYLENCIGEIVRLWHRRHSMYLAASIY

DFNERNGIHTLLFTDATIEEFDLREEDWDFLPDPDGPEEV

>SlSDG16

MIDNRSEPPPYVHIKRNAYLIKKKRDGVIADIGCTHCKSTECSDNCVCRVQCISCSKACRCSDMCSNRPFRRDRKMQVVKTELCGWGVVASESINKGDFIIEYIGEVIDDALCEKRLWDMKYKGVQNFYMCELRKDFTIDATFKGNLSRFLNHSCDPNCKLEKWQVEGETRVGVFAARYIEVGEPLTYDYRFVQFGSEVKCHCGASKCQGYLGSKKKITSKLDISWGSKRKRTSTSCLAIVKSNSF

>AtSDG4

MLDLGNMSMSASVALTCCPSFLPAASGPELAKSINSPENLAGECNGKHLPMIPPEEEVKDIKIANGVTAFTRKQNPSDRVKKGFVLDDHVKDWVKRRVASGVSESTCFLPFLVGAKKMVDCLVCHKPVYPGEDLSCSVRGCQGAYHSLCAKESLGFSKSSKFKCPQHECFVCKQRTQWRCVKCPMAAHDKHSPWSKEILHLKDQPGRAVCWRHPTDWRLDTKHAVAQSEIEEVFCQLPLPYVEEEFKIDLAWKDSVVKEDPPSYVHIRRNIYLVKKKRDNANDGVGCTNCGPNCDRSCVCRVQCISCSKGCSCPESCGNRPFRKEKKIKIVKTEHCGWGVEAAESINKEDFIVEYIGEVISDAQCEQRLWDMKHKGMKDFYMCEIQKDFTIDATFKGNASRFLNHSCNPNCVLEKWQVEGETRVGVFAARQIEAGEPLTYDYRFVQFGPEVKCNCGSENCQGYLGTKRKEPNCLVVSWGAKRRRLFHRPIARKPQQD

>LcSDG4

MPDLANLDLASLPALTRCPMTLKQICPDSASASAAQSTRADDCFCEGGRGGCEKEFRVDGNSNTAVRVKRQSRGSAKKASCSEEDAAARPRKKVELKVPQSNCFLPFLVGAKKMAECLLCHKYIYPGEEVICSVRGCQGICHLLCVKERLGLSNPMKFKCPQHHAVATCDIEKKRDDADDDIGCTTCTSECREDCVCRVQCVSCSKACRCSETCNNRPFRKEKKIKLVKTEFCGWGVEAAERKGEFIIEYIGEVIDDATCEQRLWDMKYRGTQNFYMCEIRKDFTIDATFKGNLSRFLNHSCDPNCMLEKWQVEGETRVGVFAAQSIKVGEPLTYDYRFVQFGPEVKCYCGASNCQGFLGTKRKICKSDLTCWGSKRRRTSAASVAVITV

>SlSDG33

MVQAVEHNSVLEIAQSEHKKSKTTKKGKGKGRQGKNHTGQNSQRSKGKSSIPTGPISLKVKFGSRCLMDVVPLIDDHMDKQCTTGKEFKELPNVARNFDDRLEAGLPSLQFSSCNRNLDNVYVSVSELCLSGKNISQEPVDKHLDFHHESPSQEGTSIDNRCSDSGTSPDSEVINLVPDNQIIEGEPEELNDLIPSRPSVAPGDVLSLRVYDRSKKGRKKDRLPKFASSGSKDLLSSDSMSNSQIFGPLMQGDKVQGGSCYADTSALTIGRISSGNISSTEIISGELLPCSGVPEFNISCAASKLGSGIEGNVCSSFGTESPETEFAEKVVSCHDGQNITKSGRSNLSGKGRSQVPTQKLSKSRESASKKKGNKEKQDNKLEVRHENNQVKSLSEVKNHPGTENEAPYGFGEVGSRNETLSGGISDLDIMRSEVSQPYLQPRNAWVQCDDCQKWRRIASVLADKIEETNCKWTCKDNLDRDLADCSIAQEKSNSEINAELEISDASGEEDVLRTRLNSNRSGQKKAPVSLQSSWTLIKRNSFLHRSRKSQTIDEIMVCHCKPSERRMGCGEGCLNRMLNVECVRGSCPCGERCSNQQFQKRNYAKLKCFKCGKKGYGLQLLEDVSKGQFLIEYVGEVLDLHAYDARQKEYALKGHKHFYFMTLNGSEVIDACAKGNLGRFINHSCDPNCCTEKWMVNGEVCIGLFALRDIKKGEEVTFDYNYVRVFGAAAKKCVCGSPRCLGYIGGDLQNAEVIVQADSDDDYPEPVVLCEDGDMGDELNKILSARSSFDVTEIRTPGETPKNKYKLDEPFTGNLETTTQTHTQNIMKQENSNMDSVAAFGLKIKEESNKWHNVSPSLSLKKKESSEAMEGLESLLHSSVRPVGNSLQSEDITAKTISEVKRECLDAVKISSALPSPNAMLSKSLRKKSGNGETSDESLKSSRRSSSVKKGKSKNSAVNMTSAPDVNNKLQIPQPKFKKPTHDSANGRFEAVEEKLNELLDHDGGISKRRDASRCYLKLLLLTAASGDNCNGEAIQSNRDLSMILDALLKTKSRTVLVDIIDKNGLQMLHNIMKRSQREFNKIPILRKLLKVLEYLAARGILSHEHINGGPSRPGVESFRVSILGLTEHIDKQVHQIARNFRDRWIRRPLRKSSCIDRDDSQIDLRPSPRYNRCSPLQDHCGVKPSETEECTSHLMVESTRIDAGVLDGSSTSCVDGATNGARKRKRKSRWDQEAELDVDQRIETNAVDDRTQDIDDAPPGFSIPKKASRISCGASSSADCSLQEPSCKKHPHPVVTGHLQQRFISRLPVSYGIPLSVVQQFGSPQKERCDAWSVAPGVPFHPFPPLPTYPHDRRDPISPADNAAGIFSKPPQNPQHGLSTHNPPRLSGASLRKIL

>SlSDG34

MLSQISRADNVCDFGQSTYAMDPNCSTENLSVSLQSPQPFEIRSADVSQRAATLDVSGTDVLENVSDSISITELSQKEDDKGKDTVETDNASESEYPDNACIAPRRRSGRNSKLSQSLATVPARKGRRIAIKKTSIDFSSLQITRKRRSYFSKQARSSAWGLLENTVQYLEHNVRLEIALGKQKNLRIAKKGGSRNEKHGKKQIDRKPRKSKGKSSIPRGPISLKVKFGSHCLMDGIPVIDNDTNKNSNAREELKEMTKVASEVDNRIGEEVLSVQFHGCNGNLDNDHVSLSEGCQPGKSAVQDLAAKTLVCHVESPSQDGRSINNRFSDPGTSPDSEVINLIPDTPIDVPEEFHDLTLSKPCAVPVDASILRMHEKSCKKGRKKERLPKIPNSGVKDLPTPESMSNTEVFGDLMHGEKQRNGLFCSDTSVLTTAGNGTGNMFSTVIFSGELLRCSGVSSLGMSCASSNPESDPEGNHCASVGTESPESGLSEKLVSSHDEQNVSKEGRPKESGKCRPEVPNLSKGRGSKKKGNKEKEDIMHDMKHKSDPVKCLGEGIQHSVTENGIASELGQVVSEKRSLDGGISNMDILQSEIGERLLPPRNAWVQCDDCLKWRRIPSLLADQIEETNCRWICKDNLDRAFADCSFPQEKSNSEINAELEISDVSGEEDVSRAHLSLNGSGQKNLLGAHQSSWNRIKSNLFLHRHRKNQPIDEIMVCLCKPPADGRMGCGDGCLNRILNIECAKGTCPCGEFCSNQQFQKRNYAKLKCFKYGKKGYGLQLLENVSEGQFLIEYVGEVLDMHVYEARQKEYALKCHKHFYFMTLNGSEVIDACAKGNLGRFINHSCDPNCRTEKWIVNGEVCIGLFAIRDIKKGEEVTFDYNFVRIFGAAVKKCVCGSPNCRGYIGGDPLDAEVIVQEDSDDEYPEPVLLPKYAKMDQKEDNITCATSSIKCAKIKIQRKRPNKKNTLDGLIAENQETSCQTDINSFVGQEKVNLGNSVAVVSLNVREESENFPGVSPASALKAETCATFKASECLSHSSTEPVETSLSLKDTCETVSGVRKGFTVAGDVAKYSISSAQALDITSPDAVVSKSLKKSKSSNGKETPESCLFVKTSRESSLVKKGKQRNYAVNSRSSPDVDSKLQVPQPKLKKPPDGSLHGHFEAVEEKLNELLDHDGGISKRKDASRCYLKLLLLTAASGDGCNGEAIQSNRDLSMILDAILKTKSRTVLMDIINKNGLQMLHNIMKRYRREFNKIPILRKLLKVLEHLAVRDILSPEHINGGTSRAGVQSLRSSILGLTEHEDKQVHQIARNFRDRILRPLRKRICIDKDDCRINTHSGSQYNRCLASQNQWCDLGCKTSEGADYTCHSTVASVQADGGVLDGSSASCSDIGEACMAKKRKRKSRWDQEAEAKSDPRNESDVAEDQKQVLDDDVPPGYEFPPGFSVPIKACKVLSDDSSTAIYSTEEGNWGEHPQPVVMGHLQQRFVSRLPVSYGIPFSEVQQFGSHQKGRFDAWTVSPGIPFHPFPPLPPYPCDRRGFVPTASELPQNAGEDWGACSPSHLAQNPPSVSGADQPQDGNGNQLGCERASESHNLGRKNFRKQKFNNSKLVPPWLRIRSGWEYTGNSMCIPGASRENEFRSTHNNHLGMQNLGHALRPNTFHRY

>LcSDG8

MGSCENLTAFDESLCDSVVVQSSGGEDCQELVSGHSACPGICCNMIGASVDGNDSLDGCSCFSNDEKTCSLSSDDGKTIGVGDRDGLLGESANVGGVGLEKVLDDECGVRKVSVNENRFAVGVCSSSARCLEFPQDVKDVSVSTNDVIKSVVGNGDLLVSDCQNFAALVSEKFADDECGIGLTESRSEVDVSSVKIDGLCSDVRGFQGEKVCVKSLHGCEMLLGEMPGATSLRNNDLQDEQMDDKEVDAPSVEGTVEALQKKTDVLPQVHVVQEDLNDNKGICCPSLEGVMEIMEEKTVSLAGLEKDRSDLMCPLQSCGVPSDLLPLINCVHEDSQKNDNSIHCSPSEEVMEEKADTCHQVLPSQGCVMPSELTLRTCVRLDKQQDDESVSCPSSEEVMGHKSDVLAGTEKDICKPVLSLQVCDTCLESTAMNGSSEGFVGERDVLAGTKAELLVESISSTGLSSNFVQVNEHKNTESISPYAVEGKDDASAGIEANMCNRISWSQDGEMPSKRSCKGDLVSSCGSIEGPFAESNTQFIAEQSDVTTDIKVEACSQVLLVEEGSVEKPVLLPCHPFGSDEKDSSKLDVKDPLQNNVFGDVVSIDVVDGGKQACHEEQDNVAVDCFSKTENPDIVPPFSKRSNKPSRLSRKTKAKRAARNNRNTVKVQEPQSNIDVIFKVARRKRSCFSKPARTSIWGLLDNITNFFDTSDVNRCSQGRNVRSQKAKGGQGSRKRNKNRTSGNSLGSSKKSHSHTRCLRLKVKVGKEVCQGSLNIMVPALVDTTGSDDAAVSVYRTDSLDISKLVLGGKLGEEGTERHSECFNKNMEEAETDPNDSVLDFGLSNKDLKGTVMSNKSPVNVSDECLSVPAHKGVELLGGAIDNCYMDPGTSPDSEVINLVPDVQVGARSREELHKTVLTSPKACVATEYVVSPRGGEKKDDLVLADNCMLEDRSLVAASKKKAKLSRKCDRRQKMVDNFGSGETLVASSNGEVSLERLPSSRETELGIFREEIVCSIKPKGYRVSKSSKSVGMRKGKSKVSDKARNRRKNTDKQAGNQRKSINKSKLEEISVTKRREKYACDQVEDKTEGQLEIGSNMIDEVGKSDGVDGPADVADVDIVSSVVPQQDCPAESAWVRCDDCYKWRRIPVVLANSIDENCRWVCKNNMDKAYADCSTSQEKTNADINAELGLSDCEEDVSNGLFNYNGSGKDMDCRGLAAVPGSAFRRIDSNVFLHRSRKTQTIDEIMVCHCKPPPDGRLGCGDECLNRMLNIECVQGTCPCGDQCSNQQFQKRKYADMHWLPCGKKGFGLHSLENISTGQFIIEYVGEVLDMQTYEARQKEYAAIGHKHFYFMTLSNSEVIDACAKGNLGRFINHSCDPNCRTEKWMVNGEICIGLFALRDIKEGEELTFDYNFVRVVGAAAKKCYCGSSQCQGYIGGDPQQAEVIYQGDSDDEYPEPVMLEDGETGDGLDKITSKMCSSDGARTQIAETIVKEIGNLDSSTIAMRLLETATDVEDSMSQFASAASQLQSLPETENSKGVQPVELSQPAEDGTRKLISAVQPGTSMEEKVVNKSSSFVQEVGSSSLTLMSRKSKSDTVEDKRGLSRSLPRIKTSRSSSSVKKGKATPSSSNGSKIKMMANKALHLPIKPKKSMEGSSNGRFEAVQEKLNELLDADGGISKRKDAAKGYLKLLLLTAASGNSGNGEAIQSNRDLSMILDALLKTKSRMVLKDIIDKNGLQMLHNIMKQYRRDFKKIPILRKLLKVLEYLASRKILTLDHIIGGPPRPGIESFRESILSLTEHDDKQVHQIARNFRDTWIPKSCRKSSYMDRDDSRMDFHRGVSCNRFLTSHNNWHDQSLRPSEAIDCVKQSSFATTSVDSTANEVGSEPCMGGFQTNGIKTRKRKSRWDQPAETNVDSRSLNHKEQKLESTVLQQHESGPVSKGDQVVLEHTDKVSREDSSCPSCVHNNCNKAETISSEDGRLITQEDLPPGFSSPRNPPLGSSDALTSDLPQQNVSHPPFDVAIGFQQGKFISRLPVSYGIPLHIVEQFGSPLAETVDSWVIAAAMPFQPFPPLPPFPRDNKDTLPASTVSSKTVNGSVEECQVNSHCLSACHVENNASTTRVNQAGVDYPDADTQQTFKRMKGSSNDLGKRFFRQQKLNKGPPWLWRRNELRSPYCSQEIGFRVDKPCSNFYQRPPQQNHH

>AtSDG8

MDCKENGVGDASGCNIDANSLASNLAMNTNEDFYEKLSSRGQNLDSVSSLEIPQTASSVNHTIEGQRKCFTEIEQMGYGNSNSQEDAGNTDDDLYVCYNADDTQEQGVVSGELEQSQELICDTDLLVNCNKLDDGKESQDTNVSLVSIFSGSMQEKEAPQAKEDEGYGGTTLPIGGSGIDTESTFVNDAPEQFESLETTKHIKPDEVESDGISYRFDDGGKEGRNGPSSDLDTGSSDDISLSQSFSFPDSLLDSSVFGCSATESYLEDAIDIEGNGTIVVSPSLAITEMLNNDDGGLCSHDLNKITVTETINPDLKLVREDRLDTDLSVMNEKMLKNHVGDSSSESAVAALSMNNGMAADLRAENFSQSSPIDEKTLDMEANSPITDSSLIWNFPLNFGSGGIEVCNPENAVEPLRIVDDNGRIGGEVASASGSDFCEAGMSSSRRKARDGKQCKVVQTKTSARHLRKSSRKKQSERDIESIFKCSKQKRSSLLKTSRSSEWGLPSKTTEIFLQSNNIPYDGPPHHEPQRSQGNLNNGEHNRSSHNGNVEGSNRNIQASSGSCLRLKVKFGKSGGQNPLNITVSKVSGNSLPGNGIVKAGTCLELPGSAHFGEDKMQTVETKEDLVEKSNPVEKVSYLQSSDSMRDKKYNQDAGGLCRKVGGDVLDDDPHLSSIRMVEECERATGTQSLDAETSPDSEVINSVPDSIVNIEHKEGLHHGFFSTPEDVVKKNRVLEKEDELRASKSPSENGSHLIPNAKKAKHPKSKSNGTKKGKSKFSESAKDGRKNESHEGVEQRKSLNTSMGRDDSDYPEVGRIESHKTTGALLDADIGKTSATYGTISSDVTHGEMVVDVTIEDSYSTESAWVRCDDCFKWRRIPASVVGSIDESSRWICMNNSDKRFADCSKSQEMSNEEINEELGIGQDEADAYDCDAAKRGKEKEQKSKRLTGKQKACFKAIKTNQFLHRNRKSQTIDEIMVCHCKPSPDGRLGCGEECLNRMLNIECLQGTCPAGDLCSNQQFQKRKYVKFERFQSGKKGYGLRLLEDVREGQFLIEYVGEVLDMQSYETRQKEYAFKGQKHFYFMTLNGNEVIDAGAKGNLGRFINHSCEPNCRTEKWMVNGEICVGIFSMQDLKKGQELTFDYNYVRVFGAAAKKCYCGSSHCRGYIGGDPLNGDVIIQSDSDEEYPELVILDDDESGEGILGATSRTFTDDADEQMPQSFEKVNGYKDLAPDNTQTQSSVSVKLPEREIPPPLLQPTEVLKELSSGISITAVQQEVPAEKKTKSTSPTSSSLSRMSPGGTNSDKTTKHGSGEDKKILPRPRPRMKTSRSSESSKRDKGGIYPGVNKAQVIPVNKLQQQPIKSKGSEKVSPSIETFEGKLNELLDAVGGISKRRDSAKGYLKLLLLTAASRGTDEEGIYSNRDLSMILDALLKTKSKSVLVDIINKNGLQMLHNIMKQYRGDFKRIPIIRKLLKVLEYLATRKILALEHIIRRPPFAGMESFKDSVLSFTEHDDYTVHNIARSFRDRWIPKHFRKPWRINREERSESMRSPINRRFRASQEPRYDHQSPRPAEPAASVTSSKAATPETASVSEGYSEPNSGLPETNGRKRKSRWDQPSKTKEQRIMTILSQQTDETNGNQDVQDDLPPGFSSPCTDVPDAITAQPQQKFLSRLPVSYGIPLSIVHQFGSPGKEDPTTWSVAPGMPFYPFPPLPPVSHGEFFAKRNVRACSSSMGNLTYSNEILPATPVTDSTAPTRKRELFSSDIGTTYFRQQKQSVPPWLRNNGGEKTANSPIPGNLTLEKKLNS

>OsSDG725

MEEPDGEARGREDHAAVGRLGGEEGAVGGGGLALLAVPEIGGELGDGGKVCGGQERRLPT

EEDGVRDNGGGSAAELVESAVNVSTPFEGRGQIGGEKESSMQEGSMNMAGEKHGSYHVES

AEPSNLQTCHAPNGGVSNKTLFAPFSEVFSSDNSHMRYLLDKATEGSICEHGDLADSKDD

LGGATDVKTNTEDLQMVCTKPHCDSEGLSDLHNDSERWPQVVDGVGFTIKGNNELKQVDL

IPKIEAEVSRSVEDDSIPSFSGGIDDSLRKAGCACETLNDMGMSHMANGDLWCNVLYAPL

SEGCQSKDARHIAVMGNKVTQGSQCGQGDLACDGIVLRGGVDVEKSLDDLQMCSKEPQCD

NKGFPYLTEFGVQQPSYGMNVICSKTDPNHQLEKDELLTNTRGEFSSSIHEDSVPSISVS

SVDFTFDGNAGQIGKTSEHRAIMEKVSHGSQRGGVLSCESRSLKESHADENQSSTLEVKT

CEEGLQTVDEASIPSNYNSPIDVLLYKEDGLVGEISENRIGVEKLAHDLLGEVMLSFDSR

PQTEASGDENQHFWMDVPKGSTASVCEVENTGTRRSCDPCAEIEFPLQQSREKHVISESP

PERDLTSSSHNLPCENEPCYSGRETPAFCLGHQDSAGLGLESSDCLVQELNTCTSTDDKA

CSVDFVENGNGSHNQKEVPVIFFRRRNPVRAASSRNSNFEKCDQINKSGNSTRKSKKVDS

VSSLLKSTMIKFPNKTTKGRSGINRPLNSSAWGSLQKLMDGFNQNCGPSTSRSHQTCLGK

EISNRGSSEKKQLSIRKIRTSRCSKYKNTSLSDIGYLAGELNGQPTCSVRIDTNVSSDAL

FNSPNGAHKAAQCVEGNHTLKLTSSLTDTQQFGLENVTQETCPGYIHGECGTSTSERSLN

NIVGFSPDSVLDIASVTCESNTSATLDVIVHENPSCPGGLIGGGLRASALSTSHCENHHA

SSLMDLEQQVKTVRENDMGEEDVIPSHAMMYNDIGEGKQTLAKSNTMRKGRNVGKQECRK

KDGKKGKNINKNRSSTKISSSEASKLVSFSNDSPSLDPSELLLHTRPPKFGSCSKVVTSA

IHDVGMHGYDNMRPFGIDNDDEGSAFDNVKSLRRKKKDSHGGKKGKVRDPHGKGRSKKKN

IADNTYGDLIPAAELVFKNSSAVSVELPAVVACKTDGASVPPAPAWVCCDDCEKWRCIPT

ELADKISKENLRWTCKENEDKTFANCSIPQEKTDDEINAELGLSDASADEANGDGSNSKA

SGEPNFALLRSNLFLHRNRRTQSIDEFQRRTYAKLGKFHTGKKGYGLQLKEDVSEGRFLI

EYVGEVLDITAYESRQRYYASKGQKHFYFMALNGGEVIDACTKGNLGRFINHSCSPNCRT

EKWMVNGEVCIGIFAMRNIKKGEELTFDYNYVRVSGAAPQKCFCGTAKCRGYIGGDISGA

DMITQDDAEAGTFEPMAVQEDAEEVLGANGLSSHGTHLDIVDHEASTKTEDSNDCPSVNP

PELESEQQTSGTLFDTSEPENSLEALSPQDDEDVVRTPVHVSRTVESTSRQFPEYGLHMT

VNDRYLTCTVEGRLNSLLDVNGGISKRKDATNGYLKLLLVTAAEGDNAGGTSKSVRDLSL

ILDALLKTRSNSVLLDIINKNGLQMLHNILKQNKSDFHRIPIIRKLVKVLEFLASKGILT

SEHINGGPRCAGTESFRESMLGLLRHNDMQVQQIARNFRDRWIQWAPRNISRNEPTEYSR

ASISAHDIHVISTAGGSFPTSANTMDWKSIRRKRKSRWDYQPDDHYKMGGLKIQKVCPVQ

SEFRTGSVGNKLHGNWGTNSSHNDVPVVGSSADGADDEAPPGFESQQESRPGQACLESGV

SPGLYLERYQHNLTISYGIPIAFVEHFGTPEVEGGPCRKNWKVAPGLQIETGEYIGIGEM

GREQNFHTIIKDGDFLTIIKDFESCLLPPTSQEQGDPGPRGRE

>SlSDG36

MVTHLFKDLVVERRDSGKRFQKCEYARTKLFRTEGRGWGLLADENIKAGQFIMEYCGEVLSSEVAKKRSLSYEAHKVKDAYIMSLNANYFIDATKKGSLARFINHSCQPNCETRKWIVLGKTRVGIFAKKDISVGMELLYNYNFEWYGGARVRCLCGAANCSLFLGAESQGFKLAQECSDVSEEEGNRYIMDNILLYDTTDDDESSPVISGTGEGNKHTKVLNDSEASTFKVEPTKSRTKKKSQPKPKLKLLHSSKGQVQRPELQQPHSSSTSSPLHQLSISKDKNSI

>AtSDG26

MQFSCDPDQEGDELPQYEHIYQNDFSYRKHKKQKEEDISICECKFDFGDPDSACGERCLNVITNTECTPGYCPCGVYCKNQKFQKCEYAKTKLIKCEGRGWGLVALEEIKAGQFIMEYCGEVISWKEAKKRAQTYETHGVKDAYIISLNASEAIDATKKGSLARFINHSCRPNCETRKWNVLGEVRVGIFAKESISPRTELAYDYNFEWYGGAKVRCLCGAVACSGFLGAKSRGFQEDTYVWEDGDDRYSVDKIPVYDSAEDELTSEPSKNGESNTNEEKEKDISTENHLESTALNIQQQSDSTPTPMEEDVVTETVKTETSEDMKLLSQNSQEDSSPKTAIVSRVHGNISKIKSESLPKKRGRPFSGGKTKNVAQKHVDIANVVQLLATKEAQDEVLKYEEVKKEAAVRLSSLYDEIRPAIEEHERDSQDSVATSVAEKWIQASCNKLKAEFDLYSSVIKNIASTPIKPQDTKTKVAEAGNEDHIKLLEAK

>LcSDG26

MSSSEQQIEEQQIDELPPYQHIRENEFLSRKEQKEEDIAVCTCKFDADDPESACGERCLNVITSTECTLGYCPCGAHCRNQRFQKCEYAKTKLFRTEGRGWGLLADENIKAGQFIIEYCGEVISWKEARRRSHTYEVQGLKDAYIICLNASLSIDATRKGSHARFINHSCQPNCETRKWNVLGEIRVGIFAKQDISAGTELGYDYNFEWYGGAKVRCLCGAATCSGFLGAKSRGFQEDTYVWEDDDDRYSVEKIPLYDSAEDEPSNKLITTIDSIKSEYDGNAEKDYPMVMNGSVKCEHQLESTSLVVHPLESVPMDGVVMNGGKTEESEERKLYSKDTEQAFPQSNAMISRIRSNSACRNYRIGPSSTPRKRAQHYSNGKLRHIVQKQVDVKHVAQLLASKEAQEELLINEEMRNDATAQLSSLYNEIRPAIEEHERDNQDSVATSVAEKWIEASCLKMKTEFDLYSSLIKNLACTPRRTLDQANGCGDSEVKYLEF

>SlSDG37

MLHSEEDKCNVVQLPEGVTPFIYITQNEFLGRKHKKLKEDDIAICECKYDASVPESACVERCLNVITNTECTPGYCQCGATCRNQRFQKCEYAKTKLFRTEGRGWGLLADENIKAGQFIIEYCGEVISSEEAKKRSQAYEAHGLKDAYIISLDANHFIDATRKGSFARFINHSCWPNCETRKWTVLGETRVGIFAKQDISIGMELAYDYNFEWYGGATVQCLCGAANCSIFLGAKSQGFQEYNHVWEDGDVRYTVEEVPLYDSAEDDSLPVIAGTGGGNEQTKILNDSEGSTLKLEPSNTTCKSFNIGSGSTPKKTAQRLPKRKVKSSSRKQVNDGDFAKLFASKEAREEVTMYEGLKNEATSKLNSVYEEIRPTIEEHGRDNQDSVPTSVAETWIEAHCSKYKADFDLYFSVIKNVMHPRPATYTTAAAPSEGGAVPQMTNAEPKLSQGAK

>SlSDG19

MVHSVEDKSNVMSCYRHKKLKEDDIAICQCKYDTSDPKSACVDRCLNVLTNTECTPGYCQCGDSCNNQMFQQREYAKTKLFRTNERGWGLFADENIKAGQFIIEYCGEVISSEAAKKRSYVYEAHEVKDTYMITLDTNYVIDSTRKGNFSRFLNHSCRPNCETRKWTVLGETRLGIFAMKDISVGKELTINYYFEWYAGATVRCLCGAANCCIFLGAKSQRFKEYNHVWKDGNDSHQRKHEKKLPSTRGYENKLTLKLNSAYEETRPTIEEHGYCAHQ

>SlSDG18

MVVNSFSRCLLCRCLYKEGKFCNIPKSFIISVESMIVNLMKSEIIKIDMLVSLWHIMMVNWCRLILYVVEFPSRPNCEKRKWTVLGETTLEIFVLKNIPLGKELTINYYFEWYAGATVRCLCGGAKCCIFLVAKSQSFMVT

>SlSDG29

MGDGGVACVPVQHIMEPFSVCAPKTNSSTFSTSSLNSTTATVKKKKKKMNGKMKAKREKKVVNLSSKSVVKEIESNGDAAKDEVEEGELGTLPVDNGQLVQEKSFSRKYEIKSEIEKGEITPDVKRGEFLKGRWRKGEWEKANYISDKSDRKGEFDKNDTGYEPGEFVPDRWRKGEGSARDDFNYSRTRRYDFAKDKGWKGDLDWTPPLVKDKGWRDDREWTPPSVKDKGWRNDREWTPPLVKDKGWRNDLEWTPPSAKDKGWRNDREWTPPSAKDKGWRNDHEWTPPSSGKHSGQKDGGRSGGIQHVKRLSRYEPSIPERNPRISSKIVGEEGPSKSELRNGNNPARDYFSGNRLKRHGTDSDKNDRKFRGEYDDFSSSKSRKLSDDGSRAVYTVDHSLRRSTEKLHKNAPSNRNIPPDRYSSRHYETSKVPYDRLNSSPRHLERSPRDRARHLDNWDRSPARREKSPYDRGRHFDHSRSPYDRSRHYDHRSRSPSYSEWSPQDQGRHHHRRDRTPNFMEPSPRDRSRTTYHRDTGRKSGPSDKKDSHFEGKKHEGKFNNQKDVSMKDAKDSEVRSCPENSNCSIVKSGNHPVNNDGLPQCPAVNALEPSEENGAVEEAASMEEDMDICNTPPHVTTVAEGAIGKWYYVDQFGVEQGPSRLCKLKSLVEEGYIVADHFVKHADSERWVTVENAVSPMATVNFPSVVSDVVTQMVSPPEASGNVLEDKCDLAQLNDQVAVDTFPPPSEIVPCHGDNLTAAEPSSEHHIDERVGALLEGFSVTPGRELEIIGEVLQVTLEHVEWEKWGSAEGEHWNQSSDELSLSSEVQKESTEPRTSDKETDFFCSDPAELFSGLWSCKGGDWKRIDEATQDRLWKKKLVLNDGYPLCLMSKSGIEDPRWPQKDELYNPSHSRKLDLPSWAFTPDEWNDSNVVGRPNQSKPPVLRGTKGMMLPVIRINACVVKEHGSFVSEPHTKVRGKDRHPQRSSRPYVVTGDTKRSSEEAVYRSKSRQDQELHGSSKSIMPLIIPKDRLCSADELQLHLGEWYYLDGAGHERGPFSFIELQVLVDQGVIPENSSAFRRVDRIWVPVASSSKTSDLSKMCQTPNETLGASESELENSLLSAPSGAPCTFHGMHPQFIGHTQGKLHELVMKSYKSRELAAAINEVLDPWINARQPKKESNPDFRASKKARCHGSEEEYEMEEDISVFQNDECQFDDLCSDETFNRETITTYGIKNGSWDLLNDRVLGRVFHFLKADVKSLVYASLTCKHWRSIVKIYKGISPQVDLLSVASSCTDSMMQTIMSGYNKEKITSLVLRDCTSITPRMLEDVLFSFSCLSYIDIRGCSQLDDLAVKFPNINWIRSRSSNLKVKSLKNFSDRTASSYRTYNSQENQMDDSIGLRDYLESSDKREFANQLFRRSLYKRSKAFDARKSSSMLSRDAQLRHLAMRKSRNCFKRMKEFLASSLREIMKENTFEFFVPKVGEIEEKIRSGFYASRGLKSAKEDISRMCRDALKSKNRGDAKDMNRIIALFIRLATRLEEDPKSFRTRDEMMKTSKDESPPGFSSSTTKYKKNPARMSEKKYFNRSNGSSYVNGVSDYGEFASDREIKRRLSKLRLKSLDSGSETSDDLSGSSGDTSSDNESTASETESDMDLRSECGAAESKDYFTPDDGFDSFADDREWGARMTKASLVPPVTRKYEVIDHYVIVADEKEVKRKMLVSLPEDYAGKLSVQKNGTEESDMEIPEVKDYKPRKTLGEEVIEQEVYGIDPYTHNLLLDSMPDESDWSLLDKHLFIEDVLLRTLNKQVRRFTGSHTPMIYSLKPVFEEILETADKDQDKRTIRLCQFMLNAIDTRPEDNYVAYRKGLGVVCNKEGGFSEEDFVVEFLGEVYPAWKWFEKQDGIRSLQRNNNDPAPEFYNIYLERPKGDADGYDLVVVDAMHKANYASRICHSCRPNCEAKVTAVDGQYQIGIYSTRPIAYGEEVTFDYNSVTESKEEYEASVCLCGSQVCRGSYLNLTGEGAFLKVLQEYHGLLNRHQLMLEACELNSVSEEDYIDLGKAGLGSCLLAGLPHWLIAYSARLVRFINFERTKLPDEILKHNLEEKKKYFSDVCLEVEKNESEIQAEGVYNQRLQNLALTLDKVRYVMRCVFGDPEKAPPPLERLNPEEAVSFIWRGEGSLVEELLQCMAPHLEDSMLNDLKAKIRAHDPSRSDDLETGLRKSLIWLRDEVRDLPCTYKSRHDAAADLIHLYAYTKCFFRIREYKTVTSPPVYISPLDLGPKYTDKLGPGTHEYRKTYGENYCLGQLFYWYNQANADPENCLFKASRGCLSLPEAGSFYAKVQKPSRQRVYGPRTVKFMLSRMEKQPQRAWPKDRIWSFKNSPNVFGSPMLDGILNKSPLEREMVHWLKHRPAIFQAKWDR

>AtSDG2

MSDGGVACMPLLNIMEKLPIVEKTTLCGGNESKTAATTENGHTSIATKVPESQPANKPSASSQPVKKKRIVKVIRKVVKRRPKQPQKQADEQLKDQPPSQVVQLPAESQLQIKEQDKKSEFKGGTSGVKEVENGGDSGFKDEVEEGELGTLKLHEDLENGEISPVKSLQKSEIEKGEIVGESWKKDEPTKGEFSHLKYHKGYVERRDFSADKNWKGGKEEREFRSWRDPSDEIEKGEFIPDRWQKMDTGKDDHSYIRSRRNGVDREKTWKYEYEYERTPPGGRFVNEDIYHQREFRSGLDRTTRISSKIVIEENLHKNEYNNSSNFVKEYSSTGNRLKRHGAEPDSIERKHSYADYGDYGSSKCRKLSDDCSRSLHSDHYSQHSAERLYRDSYPSKNSSLEKYPRKHQDASFPAKAFSDKHGHSPSRSDWSPHDRSRYHENRDRSPYARERSPYIFEKSSHARKRSPRDRRHHDYRRSPSYSEWSPHDRSRPSDRRDYIPNFMEDTQSDRNRRNGHREISRKSGVRERRDCQTGTELEIKHKYKESNGKESTSSSKELQGKNILYNNSLLVEKNSVCDSSKIPVPCATGKEPVQVGEAPTEELPSMEVDMDICDTPPHEPMASDSSLGKWFYLDYYGTEHGPARLSDLKALMEQGILFSDHMIKHSDNNRWLVNPPEAPGNLLEDIADTTEAVCIEQGAGDSLPELVSVRTLPDGKEIFVENREDFQIDMRVENLLDGRTITPGREFETLGEALKVNVEFEETRRCVTSEGVVGMFRPMKRAIEEFKSDDAYGSESDEIGSWFSGRWSCKGGDWIRQDEASQDRYYKKKIVLNDGFPLCLMQKSGHEDPRWHHKDDLYYPLSSSRLELPLWAFSVVDERNQTRGVKASLLSVVRLNSLVVNDQVPPIPDPRAKVRSKERCPSRPARPSPASSDSKRESVESHSQSTASTGQDSQGLWKTDTSVNTPRDRLCTVDDLQLHIGDWFYTDGAGQEQGPLSFSELQKLVEKGFIKSHSSVFRKSDKIWVPVTSITKSPETIAMLRGKTPALPSACQGLVVSETQDFKYSEMDTSLNSFHGVHPQFLGYFRGKLHQLVMKTFKSRDFSAAINDVVDSWIHARQPKKESEKYMYQSSELNSCYTKRARLMAGESGEDSEMEDTQMFQKDELTFEDLCGDLTFNIEGNRSAGTVGIYWGLLDGHALARVFHMLRYDVKSLAFASMTCRHWKATINSYKDISRQVDLSSLGPSCTDSRLRSIMNTYNKEKIDSIILVGCTNVTASMLEEILRLHPRISSVDITGCSQFGDLTVNYKNVSWLRCQNTRSGELHSRIRSLKQTTDVAKSKGLGGDTDDFGNLKDYFDRVEKRDSANQLFRRSLYKRSKLYDARRSSAILSRDARIRRWAIKKSEHGYKRVEEFLASSLRGIMKQNTFDFFALKVSQIEEKMKNGYYVSHGLRSVKEDISRMCREAIKDELMKSWQDGSGLSSATKYNKKLSKTVAEKKYMSRTSDTFGVNGASDYGEYASDREIKRRLSKLNRKSFSSESDTSSELSDNGKSDNYSSASASESESDIRSEGRSQDLRIEKYFTADDSFDSVTEEREWGARMTKASLVPPVTRKYEVIEKYAIVADEEEVQRKMRVSLPEDYGEKLNAQRNGIEELDMELPEVKEYKPRKLLGDEVLEQEVYGIDPYTHNLLLDSMPGELDWSLQDKHSFIEDVVLRTLNRQVRLFTGSGSTPMVFPLRPVIEELKESAREECDIRTMKMCQGVLKEIESRSDDKYVSYRKGLGVVCNKEGGFGEEDFVVEFLGEVYPVWKWFEKQDGIRSLQENKTDPAPEFYNIYLERPKGDADGYDLVVVDAMHMANYASRICHSCRPNCEAKVTAVDGHYQIGIYSVRAIEYGEEITFDYNSVTESKEEYEASVCLCGSQVCRGSYLNLTGEGAFQKVLKDWHGLLERHRLMLEACVLNSVSEEDYLELGRAGLGSCLLGGLPDWMIAYSARLVRFINFERTKLPEEILKHNLEEKRKYFSDIHLDVEKSDAEVQAEGVYNQRLQNLAVTLDKVRYVMRHVFGDPKNAPPPLERLTPEETVSFVWNGDGSLVDELLQSLSPHLEEGPLNELRSKIHGHDPSGSADVLKELQRSLLWLRDEIRDLPCTYKCRNDAAADLIHIYAYTKCFFKVREYQSFISSPVHISPLDLGAKYADKLGESIKEYRKTYGENYCLGQLIYWYNQTNTDPDLTLVKATRGCLSLPDVASFYAKAQKPSKHRVYGPKTVKTMVSQMSKQPQRPWPKDKIWTFKSTPRVFGSPMFDAVLNNSSSLDRELLQWLRNRRHVFQATWDS

>LcSDG2

MWAARWPPQMDSKSLGFDKFHVSQPNGNRHDLGICDEHRQPRERGELGFGGVVSIIGRKGATRRFPWAMERIVKVKKIITVKKGEGQKSGVKQTNKVAENGGGGDGVSVDKDKVKNEEVEEGELGTLKWENGEFVQPEKSQSQLQPQPQLQQQPPPQPQSQPHPQSQSQLQSMSQSQSRRSEIEKGEIVVSSSSWRRGEAEKGEFGLWRGGKDDIEKGEFIPDRWHKGEVVKDEYGYSRSRRYDYKLDRTPPSGKYVSDDLYRMKDFNRSGSQHSKSASRWESCQERNVRINSKVLDDEGLYRSDYSNGKTQGRDYSAGNRLKRHGTDSDSGERKYYGDYGDFGGSKSRRLVDDNNTRAVHSEHYSRHSVERFYRNSSSSRISSLDKYSSRHHESSLSSRVVYDRHGRSPDHSERSPRERGRYYDHRDRSPTRRERSPYSRERSPYSWERSPYARDRSPYGRDRSPYARDRSPYARDRSPHARDRSPHTREKSPYYRSRHYDHRNRSPIKADRSPQDRARVYDRRDRTPNYVERSPLDRSKAINSRETSRKSGAGEKRNSQSDSKGQEDKLGQRDGNVKHSHSSTKESQDRNSLQDFNVSEEKNIKSESHKEEPSHSSIANCRESPQVDSGPLEELVSMEEDMDICDTPPHVPVVTESSIGKWFYLDYYGMECGPSKLCELKALVGEGVLTSDHLIKHLDSDRWVAVENAVSPLITVNFPSIASDSITQLVNPPEAPGNLLVDTGDTLQYSGDETQVSLPQLMCPGDSAAASESLEDLRIDDRVGALLEGFTVIPGKEIETLAEILRTTFEPVEWQNHAGYIGEQEQYDKKAVELSRNSEIKLMESTEVRFEKDHDLASLESDDWFSARWSGKGGDWKRNDEATQERSSRKKFVLNDGFPLCQMSKSGYEDPRWHQKDDLYYPSHSRKLELPSWAFSCPDERNDGGGINRPTLNKPTIVRGVKGSMRPVVRINACVVNDHGSLVSEPRSKIRGKERHSSRSARPYSSAGDVRRSLAETDSHSKAINDQDSRLFWKSMASINMPKDRLCSADDLQLQLGEWYYLDGAGHERGPSSFSELQLLVDQGVIQKHMSVFRKFDKVWVPITSTTEASDSTVRNQSENVASSGDSLQSLVQSQGATLGESENNVNSNLFHSMHPQFIAYTRWKLHELVMKSYKREFAAAINEALDPWISTKQPKKETEKLIYRISDGDARAGKRARVHVDESDEEEEMEEDMNTIQDESTFEELCGDAFFHGEESAGSETNLGSWGFLDGHVLARVFHFLRPDIKSLTIASLTCKHWRAAVSFYKGISRQVDLSYMGPNCSDFVIRNIMNGYNKEKLNSMVLVGCTNVTSAMLEEILRLFPCIVSVDIRGCGQLGELANKFPNLNWLRSQSSRGNKVFNESYSKIRSLKQITDKSSLVSKSKGLGGDMDDYGELKHYFDSVDKRDSANQAFRRSFYQRSKVFDARRSSSILSRDARMRRWAIKKSENGYKRMEEFLASSLKDIMKENTFDFFIPKVAEIEERMKNGYYIGHGLSSVKDDISRMCRDAIKAKSRGGAGDMNRIVTLFIQLATRLEQGAKSSYYEREEMMKFWKDESPAASKYKKKLVKTVSERKYMNRSNCASLSNGVFDHGEYASDRELRKRLSKLNRKSLDSGTETSDDVDRSSEDGKSDSESTVSDTDTDLEFRSDNRFGESRGDGYFTADEGLDSMTDEREWGARMTKAGLVPPVTRKYEVIDQYAIVADEEDVKQKMRVSLPEDYAEKLNAQKNGNEELDMELPEVKEYQPRKQLGDQVIEQEVYGIDPYTHNLLLDSMPEELDWTLMEKHLFIEDVLLCTLNQQVRHFTGTGNTPMMYSLQPVIEEIRRAAEEDCDVRTIKVCQGILKAIDNRPDDKYVAYRKGLGVVCNKERGFGKDDFVVEFLGEVYPVWKWYEKQDGIRSLQKNSKDPAPEFYNIYLERPKGDADGYDLVVVDAMHKANYASRICHSCRPNCEAKVTAVDGQYQIGIYTVREIQYAEEITFDYNSVTESKEEYEASVCLCGSQVCRGSYLNLTGEGAFDKVLKEWHGLLDRHQLMIEACELNSVSEEDYLDLGRAGLGSCLLGGLPNWVVAYAARLVRFINLERTKLPAEILKHNLEEKRKYFSDICLEVEESDAEVQAEGVYNQRLQNLAVTLDKVRYVMRCVFGDPKKAPPPLEKLSPEETVSFLWKAEGSLVKELLQCMAPHVEEDVLNDLKFKIQSHDPSDSDDVQKELQRSLLWLRDELRNLPCTYKCRNDAAADLIHIYAYTKCFFRLQEYKSFTSPAVYISPLDLGPKYSDKLGAEYCEYRKTYGENYCLGQLIFWHIQTYTDPDCTLARASRGCLSLPEIGSFYAKPIFQEKQPQRPWPKDRIWSFKNSPKVFGSPMLDAVLNGSTLDRDMVYWLKHRPIVFQAMWDRFNKSFLTHNHELTPLPPSDFTTPCHHYHQLPSSSSAFNFPTQLFHLPESSSTSSSAKRARFDWHPPPVQSRSRMKLSDKTRSLQKLLPSDKKMDIATMLEEAHKYVRFLQAQVRAIQSMPLDSSFVVQTDCDWPSFGVPSSLGMLNRQQLLQIIVNSPAAQTMLYSQGFCVYSLEQLLFFNTAAQPNLFIKN

>AtSDG29

MIIKRKLKTLKRCNSTNEEDDIVRKKRKVNLNGGGSGGDYYYPLNLLGEIGAGIVPGKNGFSVSLCKQVSCSPKVEVVEEEEEEEEIKSTRLVSRPPLVKTSRGRVQVLPSRFNDSVIENWRKDNKSSGEEREEEIEEEACRKEKVKVSSNHSLKIKQQETKFTPRNYKYSSSSALCGEIDDEDKCEEIVRYGNSFEMKKQRYVDDEPRPKKEGVYGPEDFYSGDLVWGKSGRNEPFWPAIVIDPMTQAPELVLRSCIPDAACVMFFGHSGTENERDYAWVRRGMIFPFVDYVERLQEQSELRGCNPRDFQMALEEALLADQGFTEKLMQDIHMAAGNQTFDDSVYRWVEEAAGSSQYLDHVAPSQDMKKYRNPRACVGCGMVLSFKMAQKMKALIPGDQLLCQPCSKLTKPKHVCGICKRIWNHLDSQSWVRCDGCKVWIHSACDQISHKHFKDLGETDYYCPTCRTKFDFELSDSEKPDSKSKLGKNNAPMVLPDKVIVVCSGVEGIYFPSLHLVVCKCGSCGPERKALSEWERHTGSKAKNWRTSVKVKSSKLPLEEWMMKLAEFHANATAAKPPKRPSIKQRKQRLLSFLREKYEPVNVKWTTERCAVCRWVEDWDYNKIIICNRCQIAVHQECYGTRNVRDFTSWVCKACETPEIKRECCLCPVKGGALKPTDVETLWVHVTCAWFQPEVCFASEEKMEPALGILSIPSSNFVKICVICKQIHGSCTQCCKCSTYYHAMCASRAGYRMELHCLEKNGRQITKMVSYCSYHRAPNPDTVLIIQTPSGVFSAKSLVQNKKKSGTRLILANREEIEESAAEDTIPIDPFSSARCRLYKRTVNSKKRTKEEGIPHYTGGLRHHPSAAIQTLNAFRHVAEEPKSFSSFRERLHHLQRTEMERVCFGRSGIHGWGLFARRNIQEGEMVLEYRGEQVRGIIADLREARYRREGKDCYLFKISEEVVVDATEKGNIARLINHSCMPNCYARIMSVGDDESRIVLIAKTTVASCEELTYDYLFDPDEPDEFKVPCLCKSPNCRKFMN

>AtSDG16

MIIKRKFKTQIPSLERCKLGNESRKKKRKLNLGGGGYYYPLNLLGEIAAGIVPGNGRNGFSASWCTEVTKPVEVEESLSKRRSDSGTVRDSPPAEVSRPPLVRTSRGRIQVLPSRFNDSVLDNWRKDSKSDCDLEEEEIECRNEKVVSFRVPKATNLKSKELDRKSKYSALCKEERFHEQHNDEARARVDEKLPNKKGTFGPENFYSGDLVWAKSGRNEPFWPAIVIDPMTQAPELVLRSCIPDAACVVFFGHSGNENERDYAWVRRGMIFPFVDYVARFQEQPELQGCKPGNFQMALEEAFLADQGFTEKLMHDIHLAAGNSTFDDSFYRWIQETAVSNQELNNNAPRQGLLKKHRNPLACAGCETVISFEMAKKMKDLIPGDQLLCKPCSRLTKSKHICGICKKIRNHLDNKSWVRCDGCKVRIHAECDQISDRHLKDLRETDYYCPTCRAKFNFDLSDSEKQNSKSKVAKGDGQMVLPDKVIVVCAGVEGVYFPRLHLVVCKCGSCGPKKKALSEWERHTGSKSKNWKTSVKVKSSKLALEDWMMNLAELHANATAAKVPKRPSIKQRKQRLLAFLSETYEPVNAKWTTERCAVCRWVEDWDYNKIIICNRCQIAVHQECYGARHVRDFTSWVCKACERPDIKRECCLCPVKGGALKPTDVETLWVHVTCAWFQPEVCFASEEKMEPAVGILSIPSTNFVKICVICKQIHGSCTQCCKCSTYYHAMCASRAGYRMELHCLEKNGQQITKMVSYCAYHRAPNPDNVLIIQTPSGAFSAKSLVQNKKKGGSRLISLIREDDEAPAENTITCDPFSAARCRVFKRKINSKKRIEEEAIPHHTRGPRHHASAAIQTLNTFRHVPEEPKSFSSFRERLHHLQRTEMDRVCFGRSGIHGWGLFARRNIQEGEMVLEYRGEQVRGSIADLREARYRRVGKDCYLFKISEEVVVDATDKGNIARLINHSCTPNCYARIMSVGDEESRIVLIAKANVAVGEELTYDYLFDPDEAEELKVPCLCKAPNCRKFMN

>LcSDG16

MLSLKRCKLGESGDDDGGNWARRRRKRRKTSNGYYPLNLLGDVAAGVLPMGFDGILGFGWCGERSEPPKRPSIKEGGGGVSAAACEDFARAGSARSSNHNHNNVRNKSKVDRMGYRRSKYATLCEEEGEEEDDVKFRRSFDFGKYMGARRSSVMSVVHEGGLVDSDDEKSLREENLIMNVEREDDGLYGPEDFYSGDIVWAKPGRKDPYWPAIVIDPMTQAPELVLRSCIADAACVMFFGYSEDGNQRDYAWVKRGMIFPFVDYVDRFQEQSELSDCKPSDFQMALEEAFLADQGFIEKLMQDINMAAGNPTYDESVLKWVQEATGSNAELEYHFLNQGLCRKNKSTRPCDGCGMSLPSKSLKRQEASATGGQYFCKTCARLTKSKHFCGICKKVSNYSDSGSWVRCDGCKVWVHAECDKISSNRFKDLGGMDYYCPTCKVKFNFELSDSEKWQPKVKCNKNSGQMVLPNKVVVLCSGVEGVYYPSLHTVVCKCGYCGTEKLALSEWERHTGSKLRNWRTSVRVKDSMLPLEQWMLQLAEYHTNAHVLAKPPKRPSIKERKQKLLAFLQEKYEPVCAKWTTERCAVCRWVEDWDYNKIIICNRCQIAVHQECYGARNVRDFTSWVCKACETPDIKRECCLCPVKGKLLDRGALKPTDVDTLWVHVTCAWFQPEVSFASDEKMEPALGILSIPSNSFVKICVICKQIHGSCTQCCKCSTYYHAMCASRAGYRMELHSLEKNGRQITKMVSYCAYHRAPNPDTVLIIQTPLGIFSAKSLMQNKKRAGSRLISSGRTKLEEESIVESSEIDPFAIFCCKVQSIQERTEHDRVCFGRSGIHGWGLFARRNIQEGEMVLEYRGEQVRRSIADLREARYRLEGKDCYLFKISEEVVVDATDKGNIARLINHSCMPNCYARIMSVGDDESQIVLIAKTNVSAGDELTYDYLFDPDEPEEFKVPCLCKAPNCRKFMN

>SlSDG26

MIIKKSLKTVMPSLKRCRVSDSGADEDDFSGNNNRKKRKSSGGYYPLHLLGEVAAGIIPFNGYRIQTILAAGGDGGAAAAAAASWCTEVSRCAGEAEMNSPPKQRSNPVNEASRPPLVRTSRGRVQFDEAIDLSGTDAMVMQEGGRRAYRYGHGGFNSGDIVWAISGRHCPAWPAIVLDSETQAPQQVLNYRVAGTVCVMFFGYSGNGTQRDYAWIRRGMLFPFQEHVDRFQGQTDLNDSTPADLRSAIEEAFLAENGVVEMLMVEINAAAGNLDYLRSLPRGVFEACDSNQDQECNSPSQARFKGLLKKKELDSCDACGSRLSSKPSRKLNDSTLRSHRLCTACARLKKSKHYCGVCKKIRNPSDSGTWVRCDGCKVWVHAQCDKISSRNLKELSTSDYYCPECRARFNFELSDSENMNSKAKNNKNDTQTVALPDKVSVICSNVEGIYFPRLHLVVCKCGYCGAQKQALSEWERHTGSKIKNWKTSVRVKGSLLPLEQWMLQMAEYHAQNVVSTKSVKRPSLKVRRQKLLSFLQEKYEPVYAKWTTERCAVCRWVEDWDYNKIIICIRCQIAVHQECYGARNVRDFTSWVCRSCETPEIERECCLCPVKGGALKPTDIQQLWVHITCAWFQPEVCFASDEKMEPAVGILRIPSNSFVKICVICKQIHGSCTQCCKCSTYYHAMCASRAGYRMELHCSEKNGKQVTRMVSYCAYHRAPNPDTVLIIQTPKGVFSARSLLQNNKRTGSRLISTSRLKLEEAPAAEIEEIEPFSAAKCRVYNRLRDKGTGETAIAHHVRGPCHHSSSSMRSLSIIREVRGSKTFSTFRERLRELQRTENDRVCFGRSGIHRWGLFARRNIPEGEMVLEYRGEQVRRSVADLREARYRVEGKDCYLFKISEEVVVDATDKGNIARLINHSCMPNCYARIMSVGADESRIVLIAKANVAAGDELTYDYLFDPDECEDFKVPCLCKAPNCRKFMN

>SlSDG25

MVKRTVKVEMPKLKRCKAEGNDSGGEGESCSASPKKLKTDELFTVPIRELEDYRTSLVDSFCREALSYAGEVESSLVLAGASRSLDKALEVSNNKPPLLKSSRGRIQVLPSKFNDSVLPSWRKEENQEEQELLCLNEKDEEAVLPRKKRFKLERSNVDIHFFKNQLIHLPSSIKIQDREFSSMQSKDCSRSSVTSIGDGGSSVVVESGECKLRVKRGTVRADNFTKEKVGKKKDFFEPADFVSGDIVWAKCGKNYPAWPAVVIDPLCEAPEAVLRACVPGTICVMFYGYSRSGQRDYGWVKAGMIFPFQEYMDRFQEQTKLYGSRPSDFQMAIEEAILAEHGYTNKCPEMEQEASPATNDSGVEEATGSNQELEFCFSDQDGYDKRKDTRPCDSCGLVVLRRTLKKVKDKMSKAQFSCEHCTKLKKSKQYCGICKKIWHHSDGGNWVCCDGCDVWVHVECTDISSNALKNLQNTDYFCPKCKGISNKKLLGSVQGGPKARLRESSGSVMPDKITVVCTGVEGIYYPDIHLVQCKCGSCGIRKQTLSEWEKHTGCRAKKWKCSVKVKGSMITLEQWLSDNNAHNVSYQKLDQQQLFAFLREKYEPVHAKWTTERCAICRWVEDWDYNKIIICNRCQIAVHQECYGVSNGQDFASWVCRACETPEIERECCLCPVKGGALKPTDIDSLWVHVTCAWFRPEVAFHNADKMEPAAGLLRIPPNTFLKACVICKQVHGSCTQCCKCATSFHAMCALRAGYHMELNCSEKNGIQITRWLSYCAFHRTPDTDNVLVMRTPFGVFSTKSLVERQSQEHCSGGKRLISSKTLELPDASDAGRSSFEPLSAARCRVFQRSSYKRAGQEAVFHRLMGPRRHSLEAIDCLSAQELTRDVKAFSTLKERLIHLQMMENRRVCFGKSGIHGWGLFARRSIQEGEMVLEYRGEKVRRSVADLREARYRLEGKDCYLFKVSEEVVIDATNKGNIARLINHSCMPSCYARILSLGEEESRIVLIAKRNVSAGDELTYDYLFDPDEHDDVKVPCLCGAPNCRKFMN

>SlSDG24

MVVKRKTNEKEIVDEIYRENWFFTSKKQKFDEVFGAGFKDFSFQFKVEECKSPNFQPQVQDYCFSAESPRSHLDREVKVELRKINICCSACKKVCSDLNENSLCPDCGVNSDFIGVICNGMEGIYFPELHMVECRCGSCRAKKLTVGEWERHAGSRAKKWKVSIKVMMTMQPLGEWVANNNGHGIITPLKIDRRQQLMSVLQEKYNPVYAKWTVERCAICSWIEDWDFNKIIICSRCQIAVHQECYGAREVQDLASWVCRACETPEVERECCLCPVKGGALKPTDVDPFWIHVTCGWFRPEIAFVDYEKMEPATGLLAIPSKSFHQACSICQQTHGSCIQCSKCTISYHSTCASRAGYYMEMQCSEKNGTQTTKWLSYCASHKAPSEDNILVMRTPGGVYSNQKLLQRRNGGRVLKGLRLMPSDTSSAEANQPNAFSAGRCRVFRPSTDKKAKPEPIIHRVTMPHHHSLTVIQSLTSEQPQEDKNFPTLRERLHHLSKTINHRVCFGKSGIHGWGLFAKRKLQEGEMVAEYVGEKIRGSVADLRERKYKSQGKNCYFFRITEEVVIDATMKGSIARLINHSCMPNCFARIMSLGENEERIVLFAKKDVSAGNELTFDYRFEPDQNDEVKVPCHCGAPNCSKFMN

>AtSDG14

MILKRTLTTFENQNLKRCKIDSEIEYGRKKGEIIVYKKRQRATVDQPCSKEPELLTSSSSSLTSKEESQQVCSDQSKSSRGRVRAVPSRFKDSIVGTWKSSRRKGESTESSHDDDDVSLGKKVKGFSGSSKLHRSKDSKVFPRKDNGDSSEVDCDYWDVQISYDDANFGMPKKSDASRKGVYKPEEFTVGDLVWAKCGKRFPAWPAVVIDPISQAPDGVLKHCVPGAICVMFFGYSKDGTQRDYAWVRQGMVYPFTEFMDKFQDQTNLFNYKASEFNKALEEAVLAENGNFGDAEIISPDSSATESDQDYGPASRFQGSYHEDIRTCDGCGSVMPLKSLKRTKDSQPEELLCKHCSKLRKSNQYCGICKRIWHPSDDGDWVCCDGCDVWVHAECDNITNERFKELEHNNYYCPDCKVQHELTPTILEEQNSVFKSTEKTTETGLPDAITVVCNGMEGTYIRKFHAIECKCGSCGSRKQSPSEWERHTGCRAKKWKYSVRVKDTMLPLEKWIAEFSTYTLETQMLDKQKMLSLLEEKYEPVRAKWTTERCAVCRWVEDWEENKMIICNRCQVAVHQECYGVSKSQDLTSWVCRACETPDIERDCCLCPVKGGALKPSDVEGLWVHVTCAWFRPEVGFLNHENMEPAVGLFKIPANSFLKVCTICKQTHGSCVHCCKCATHFHAMCASRAGYNMELHCLEKNGVQRTRKSVYCSFHRKPDPDSVVVVHTPSGVFGSRNLLQNQYGRAKGSRLVLTKKMKLPGFQTQTQAEQSRVFDSLSAARCRIYSRSNTKIDLEAISHRLKGPSHHSLSAIENLNSFKASFSFRAPFMSVFCFLGATFSEYLRKILISIYLVTHQEADFTSFRERLKHLQRTENFRVCFGKSGIHGWGLFARKSIQEGEMIIEYRGVKVRRSVADLREANYRSQGKDCYLFKISEEIVIDATDSGNIARLINHSCMPNCYARIVSMGDGEDNRIVLIAKTNVAAGEELTYDYLFEVDESEEIKVPCLCKAPNCRKFMN

>LcSDG14

MIVKRHLKLETQDPRQCKIGEVTCYEDDEDYEYTYAANPRKRRRKDGDVCRPNSMHVEVDEFSSGSGSWAACWALELESNSKSFKFNRRDKNRSSKKFRRPTLKPLKGRTQMLPSKYDDSVLIVGDTDSSIGDDEDDNDGSLLQLKEKKFNGMRYVRDKLRFGSSNYYSLGEKGLGYLDYNDFDPREYLVSRSSVMPAHSKLNSLPLVDNKQYMSAFSSRSVERLTKEKGKKRKDVYKPEDFALGDLVWAKCGRTYPAWPAIVIDPILQAPEAVLRCCVPGALCVMFFGYSKNGTQRDYGWVKQGMIFPFAEFMDRFQEPTQLYKSKLSGFQVALEEAILAENGFLDASLGIEQVGNPEAHSSISACYKDAGACDGCGLIRPCKVRKLNDSISETQFLCDHCAKLNKSRQYCGICKKIWHHSDGGNWVCCDGCNVWVHAECDKIFGKHFKDLEKSDYYCPDCKANLRFESSAEQWHPNVKPTESNGQMVLPDKVIVVCNNVEGAYIPKLHLVVCKCGSCGPKQQTLSEWERHTGCRAKKWKYSVKVKGTMLTLEKWIAEFNAHGIDPVKLDKQKLLAFTKEKYEPVYAKWTTERCAICRWVEDWDYNKIIICNRCQIAVHQECYGANDVQDYTSWVCRACETPDVEKQCCLCPVKGGALKPTDVETLWVHVTCAWFRPEVTFMNHEVMEPASGILRIPANSFLKSCIICKQAHGSCTQCCKCATYFHAMCASRAGYGMEVQSFEKNGKQIIRKLMYCAIHRTPAPDAAVVVLAPTGVFSGGSMLRNQRGCFRGSRLISAKRTESPESSTAETNEFDPLSAARCRVFKRSKNKGSGGEPVFHRPMGPIHHSLDAINSLTANKELDDHKVFTSFKERLCHLQRTEKQRICFGKSGIHEWGLFARRNIQEGEMVVEYLGENVTQKVADLREARYRKEGKDCYLFKISEEVVIDATDKGNIARLINHSCMPNCYARIMSMDGRECRIVLIAKTNVRAGDELTYDYLFENDEKDELKVPCLCKAPNCRLFMN

>LcSDG30

MAFLHQKKPLQDDDDNNNTSGTPLRYASLDRVYSASCVTGAMSTKKVKARKLLLDDDRDQPPPPDRDHLKRPPIINVYSRHSKRPRNASSFFDSLLADCGESRVSKKRRRRFGSSELVKLGVASSAVLLRSFEGPRLRDCRNHKISSPISRTKKPHCKRTTPTALSSSTKRWCRLSFNGVDPRTFIGLQCKVYWPLDADWYSGHVVGYDAESNRHNVKYQDGDKEDLILSNENVKFHISLDEMDRLKLSPSVDNMDNHGSDFNEMVVLAASLDDCQELEPGDIIWAKLTGHAMWPAVVVDESLIGGRKGLNKLSGGKSVSVQFFGTHDFARINVKQVISFLKGLLSSYHLKCKKPRFTRSLEEAKVYLSEQKLPRRMIQLQNGIGVDDSENACSEDEGSAASNVDCIKDKRIQGSPGSLGTSPVVLGDLQIISLGKIVKDSEYFQDDRSIWPEGYTAIRKFTSITDPNGYSSYKMEVLRDAESKIRPLFRVTLDNTEQFEGSTPSACWNKIFRKIRKRQLNSSDCISAEGGLERIYGSGSDMFGFSNPEVVKLLQGLSKSRLASRYSISKFTSGRYRDLPVGYRPVRVDWKDLDKCSVCHMDEEYENNLFLQCDKCRMMVHARCYGELEPVDDVLWLCNLCCPGAPESPPPCCLCPVIGGAMKPTTDGRWAHLACAIWIPETCLSDVKRMEPIDGLNRVSKDRWKLLCSICGVAYGACIQCSNNTCRVAYHPLCARAAGLCVELEDEDRLHLLSLDEDDEDQCIRLLSYCKKHRQPSNERLAIDERIGQNIRHCSDYAPPLNPSGCARSEPYNYFGRRGRKEPEALAAASLKRLFVENQPYLVAGYCQHGLSGNALPSIGVIGSKFSFNIRKDAPNDILSMADKYKYMRETFRKRLAFGKSGIHGFGIFAKHPHRAGDMVIEYTGELVRPAIADRREHFIYNSLVGAGTYMFRIYDERVIDATRAGSIAHLINHSCEPNCYSRVITVDGDEHIIIFAKRDIKQWEELTYDYRFFSIDEQLPCYCGFPRCRGIVNETESEEQAAKLYVPRKKLYRANIMPIWDPPEDHLPSYNLLMIACLAYSQSTDGLLVLWSHVKLYCFLIFDLTRRTQ

>AtSDG30

MISMSCVPKEEEGEDTQIKTELHDHAADNPVRYASLESVYSVSSSSSSLCCKTAAGSHKKVNALKLPMSDSFELQPHRRPEIVHVYCRRKRRRRRRRESFLELAILQNEGVERDDRIVKIESAELDDEKEEENKKKKQKKRRIGNGELMKLGVDSTTLSVSATPPLRGCRIKAVCSGNKQDGSSRSKRNTVKNQEKVVTASATAKKWVRLSYDGVDPKHFIGLQCKVFWPLDAVWYPGSIVGYNVETKHHIVKYGDGDGEELALRREKIKFLISRDDMELLNMKFGTNDVVVDGQDYDELVILAASFEECQDFEPRDIIWAKLTGHAMWPAIIVDESVIVKRKGLNNKISGGRSVLVQFFGTHDFARIQVKQAVSFLKGLLSRSPLKCKQPRFEEAMEEAKMYLKEYKLPGRMDQLQKVADTDCSERINSGEEDSSNSGDDYTKDGEVWLRPTELGDCLHRIGDLQIINLGRIVTDSEFFKDSKHTWPEGYTATRKFISLKDPNASAMYKMEVLRDAESKTRPVFRVTTNSGEQFKGDTPSACWNKIYNRIKKIQIASDNPDVLGEGLHESGTDMFGFSNPEVDKLIQGLLQSRPPSKVSQRKYSSGKYQDHPTGYRPVRVEWKDLDKCNVCHMDEEYENNLFLQCDKCRMMVHTRCYGQLEPHNGILWLCNLCRPVALDIPPRCCLCPVVGGAMKPTTDGRWAHLACAIWIPETCLLDVKKMEPIDGVKKVSKDRWKLLCSICGVSYGACIQCSNNTCRVAYHPLCARAAGLCVELADEDRLFLLSMDDDEADQCIRLLSFCKRHRQTSNYHLETEYMIKPAHNIAEYLPPPNPSGCARTEPYNYLGRRGRKEPEALAGASSKRLFVENQPYIVGGYSRHEFSTYERIYGSKMSQITTPSNILSMAEKYTFMKETYRKRLAFGKSGIHGFGIFAKLPHRAGDMVIEYTGELVRPPIADKREHLIYNSMVGAGTYMFRIDNERVIDATRTGSIAHLINHSCEPNCYSRVISVNGDEHIIIFAKRDVAKWEELTYDYRFFSIDERLACYCGFPRCRGVVNDTEAEERQANIHASRCELKEWTES

>AtSDG27

MACFSNETQIEIDVHDLVEAPIRYDSIESIYSIPSSALCCVNAVGSHSLMSKKVKAQKLPMIEQFEIEGSGVSASDDCCRSDDYKLRIQRPEIVRVYYRRRKRPLRECLLDQAVAVKTESVELDEIDCFEEKKRRKIGNCELVKSGMESIGLRRCKENNAFSGNKQNGSSRRKGSSSKNQDKATLASRSAKKWVRLSYDGVDPTSFIGLQCKVFWPLDALWYEGSIVGYSAERKRYTVKYRDGCDEDIVFDREMIKFLVSREEMELLHLKFCTSNVTVDGRDYDEMVVLAATLDECQDFEPGDIVWAKLAGHAMWPAVIVDESIIGERKGLNNKVSGGGSLLVQFFGTHDFARIKVKQAISFIKGLLSPSHLKCKQPRFEEGMQEAKMYLKAHRLPERMSQLQKGADSVDSDMANSTEEGNSGGDLLNDGEVWLRPTEHVDFRHIIGDLLIINLGKVVTDSQFFKDENHIWPEGYTAMRKFTSLTDHSASALYKMEVLRDAETKTHPLFIVTADSGEQFKGPTPSACWNKIYNRIKKVQNSDSPNILGEELNGSGTDMFGLSNPEVIKLVQDLSKSRPSSHVSMCKNSLGRHQNQPTGYRPVRVDWKDLDKCNVCHMDEEYENNLFLQCDKCRMMVHAKCYGELEPCDGALWLCNLCRPGAPDMPPRCCLCPVVGGAMKPTTDGRWAHLACAIWIPETCLSDVKKMEPIDGVNKVSKDRWKLMCTICGVSYGACIQCSNNSCRVAYHPLCARAAGLCVELENDMSVEGEEADQCIRMLSFCKRHRQTSTACLGSEDRIKSATHKTSEYLPPPNPSGCARTEPYNCFGRRGRKEPEALAAASSKRLFVENQPYVIGGYSRLEFSTYKSIHGSKVSQMNTPSNILSMAEKYRYMRETYRKRLAFGKSGIHGFGIFAKLPHRAGDMMIEYTGELVRPSIADKREQLIYNSMVGAGTYMFRIDDERVIDATRTGSIAHLINHSCVPNCYSRVITVNGDEHIIIFAKRHIPKWEELTYDYRFFSIGERLSCSCGFPGCRGVVNDTEAEEQHAKICVPRCDLIDWTAE

>OsSDG732

MVPLAWQQPQTRLRLQTVGQPVARAVASASASACSGAGDGDGDTAAEPDAHMASFYCVRV

QIDSASLRHILRPFAAQGAAPPEQLAASHPSSQYGQPARAAPSTSLLAQVAGNHPHATHV

SDRKALFGMLNAGNAANVIDLTRASPLGGAEPLPKHPRHGLEASSSVEQPSCLGPLFQNT

SANVQGISQGAIQFQDSSTCAVQKLPSQSTPRHHPALLGDQICVSCLNVGGFNLQQYLQP

RSTTLCHGLMREVVIEWLVTHSGGPAEKAGEIVIMDNGMTIVQWLKYCMGVGASISDTKW

DWPEWAYMRYSSEEYWTKSLLTTNNNMEKTGLFSGHGKSTGHINNPVYSSDIHNEVGRFT

SVEKLVNKPDETFYRKSVGLHEAFSKNPAIQQSSKINLANHMIHDMNMNSISRPSERTYS

TANMGITYSRNHLAHDYANFLEKNLNNLSRSPGPSSTRVLSNDSRACMPDVPHKIIQDGS

GRASNTELKLGQSSYHQSMATLFPSVQSTIIEFQKPQHHLQFTTPKSLGADAYPKQTTKA

NKTIENIEPSFGTGNRKRSLDVSNGTSHSELNEITDDAAKNSFISLFLSHLERNSTSESI

DDVLNSNEHYLLKAPDVAYSSDRLKTASTQVETRANDNQLKLAPAIIHTKRISDSRSLPV

PVASKGYVHQDVLHANSQEPLINGDCLPHLLPSQPNAGISKICAEVSSPVNCSGTLSHIS

GLDEVRTRSTFIPRSSLCSRELMLQSCCHACPIDGYYRSSMGHTANSLTKNTLLDAPNNT

ECSPYRDGKCCCSLAPKCLAGYGFTKHCVARIDQTDHTVQKSKDDGMQAAARCCTLGESE

KLICQCSSEIIARKSDSKASFQNEVSTEVLNRPCVPTLQQLKNVTEASAVGGHWPYETVK

EKASACRDSGIFKELKSGFSSGFSSDVVTKFSASPELNKYGLEHKNLVFDEGSRIEKCSS

SSYLPISTGCEEAQNSFSRFHLEPSLVKHKNNQISEGSTRKEHENEGQCSEMSKKTRTLR

CCANKSESDDCTRKIDLSSREGDSQPQHKAGPFSRRVSKTKRKHPPTHLNKHVKRLHSNC

KVLNVDNERSDDEGIYVGESNSSDRKKQEDNMTTLDRTKCQQQGSRLLVRKLPKYVSLNC

IVNETNSEDACSGSASIDSSLIATGITNDNRKSPKIVPLNLILKKAKRCHAIKPLSKTEN

IHFSEEKSSDGSADKSSSGDRSFSPQDELWSPKKNRYSSNVSRPHVKTDCQSPCCVLEED

EPLSLADMGTSQLSASRSRGSKNQRACISLNRMERYIQRPSLDASCCVCGISNLEPSNQL

IECSKCFIKVHQACYGVLKVPRGQWFCKPCKINTQDTVCVLCGYGGGAMTRALKAQNILK

SLLRGIATAKRSDKYVYSSGNVNSECTSKLHGEYVRHDSFNGHRSRSFNAISSFGIKEAS

IGSARGDIISKSWTSNRNSSLLGPRTRQWVHVVCGLWTPGTKCPNTITMSAFDISGASPA

KRNTECSMCNRTGGSFMGCRDVNCSVLFHPWCAHQRGLLQSEPEGEHNENVGFYGRCLDH

AMLDPNHVNPKKECLRSNDWTCARTEVFRGRKGDSFGANRSRKPEEKFGECSVSQEQINA

WIRINGSKSCMRGQKEYVHYKQLKGWKHLVVYKSSIHGLGLYTSEFIPRGSMVVQYVGEI

VGQCVADKREIEYQSGKRQQYKSACYFFKIGKEHIIDATRKGGIARFINHSCQPNCVAKI

ISVRNEKKVVFFAERHINPGEEITYDYHFNREDEGQRIPCFCRSRGCRRYLN

>SlSDG20

MSCQSNGESENVSTPCDAGGSSTIDKSSMVYPQAVLATGWMYVNEQGQMCGPYIKEQLYEGLSTGFLPEELHVYPVLNGAISNAVPLKYFNQFPEHVATGFAYVMVSSSGANGPTDKSMGVAKDSGGNEMDLQTTSPYSNSVAQHGTHLLNQQMATTGSAGTLAPSTTSVNEESCWFFEDHEGRKHGPHSLMELYSWCHYGYIVDSVMVHHVAGKYRPFSLKSLISSWTTATPGALFLSNPDGHETASLQDFVSEISQEVCSQLHMVIMKAARRTLLDEIVSHAISECISEKKDLKKAANQKKVTNQKKVINQSVKMSSPGTRMSAGCGGSKALIDPERSAEAPNLLNWESAAAEIPSKSSGSSKSVGSFENYCDSYTVVCRKVFDSCMHSIWNAVFYDHVSEYSSAWRKRKLWSPPCLMVESSIQAVSYANCTTKLSTEVLQGEEESFPPGFEKKNVTVDLPPVSSSKDFTVELSTEVLQVEEESFGCDPDYPPGFEEKNMTADIPSVSSSKDCTAELSTEVLQVEQESFACDVDFPPGFEEKNLTADLPLVLPSKDCTVELSTEVLQVEQESFGCYLDFPPGFEEKNMTVNLPLVSSPFNDERVLSRSSHATDPEANDCIQPIVERVLHELHLSAKMSLGKYFTSLLHEEAMGKVDLLKDGMIIKVAEDPNTFSGAACQNDSSEAILVSENLAHVDIQNTSSCKSSLHQNSIDPYVIRVSDWFSSAFQKSASLDSASSNEMTDELQPPECEAVPVQTSKVRLARSDDSILRIIWYATLSNCRQRVHEKALRELKSFLVDDIIRNFLTTSSSARRCSKSEDSQVTRSKAGNETRDKSPVALSKSGDGSPKVPTAVGKYTYYRKKKMVKRKLGSSSQPLLGGDIGYEKSSINKSRKKDLSGEATAKTKGDSATSSEKEIGLKDCRRELFTNASLVVPPSSLTSCNTSSEKDASVYKAGKSNASRKKLKATFVAEVCSDNGEVSPDIVFRKRSIRKKSRKQDLLVEATGSTKVDNADLNGIEIRPKDCRRELFTNASLVVPPSSVTNCNTISEKISSASKARGSSASRKKLKDAFVAEVSSDNGKVGEDVGFKKRSIDKSRKQDLLGEATESTKGDNAALNVKEFGLKDCSRELLTNKSLIVPPSSVINCDIISEKVASYSQARRRNASRTKLKAANVTEVSSDNGMVDGNIGIKKRTINKSTKQDPLGEETEINKGDNAALHVEEIGLKDCHKELFTNAALVVPPSSVINFNTISEKVASVSRGRSNTGHSKLKATFVAADSSGDGKVSEVANRELGTQEMQPPSCSKKTPKSAKLPDLKKRKLEDNLTASRSRKIQKQSTGVGNQAATKVATPEKNQKGKSRIAKHCSQSVGCARTSINGWEWRKWSLRASPAERARVRGTKVVHIQSASSDANGSQMLNAKGISARTNRVKLRNLLAAAEGADLLKATQLKARKKRLRFQQSKIHDWGLVALEPIDAEDFVIEYVGQLIRRRVSDIREHYYEKIGIGSSYLFRLDDDYVVDATKRGGIARFVNHSCEPNCYTKVISVEGQKKIFIYAKRHIAAGEEITYNYKFPFEEKKIPCNCGSKRCRGSMN

>LcSDG12

MVSSASLVHDYDDCFLSWKRQRVSDMIHRDFVSSISEGVCAEVDSSIQPSVKECFWFGCINTGRISSSCCNLDEKFIMETSCQSNGTSGDMPQSSSSGGASCPDNRYYVPPTTVSGWMYVNENGQMCGPYIQHQLFEGLSTGFLPDELLVYPVVNGTLINPIPLKYFKQFPDHVSTGFAYLNAGSLSTTVTPNRSFSHSGNMISPRQEGLVQHSAPVTVSPDSLLQSQSLVKYNSYFSNLSKSEEEFPPLSGEDACWLFEDNGGAKHGPHSLLELYSWYQYGYLQDLIMIHHVENKFGPITLLSAINLWRTNRPETFPASDVKINEAGSFVNFLSEISEELSSQLHAGIMKATRRVLVDEIISNNISEFVSTKKAQRHLKVDLVNQHAKSCFSGGKVPEIPHEVNSHATAECEAVACHSITDRVCSYEMHTKSPASKKCVGSIENFWGSYTVVCGALSNYCMQVVWNAVFYDHIVECSSAWRKRKLWSDHPKIALPACNNRDYDNTNDIGSGGHLPSGEDSSVCTNDCPPGFEMMAIETDNDLQSSKLSSSTLLEEKSCKQKNVSCNNHLLYGDRKCIIEAVENELYLSIRMILAEYVETLVEKVARQVVNSSKDDKKGENAIGSSSHCLHDCEFGFGGMHGELRIDSTEMSAEKISCEDSENLLDVGKPLDKDLLTNTLACVFKKSRDYCVDDVVDEPEIDEPAPPGFEENTLSVVLSGINKIQPSSSEDGTVGKIQSSSSEDSTAKIRLYIATAICRQKLHDNVLREWKSLFASGALDQFFTSWHTSRKECEPDGSEQGASGAYNEHCGDVPNKVDKLREVSTSTSVERYTYQRKKKLMRKKIGLSSLPSTPVDYGIQTQPAEKSRKQRVLRDASDYAEAEASAVSSKKIRKNKVETKSSHKARSSKITVKSNLSSDHTSTKKRSDQKVMKVSHTVKKENVDVIKPLRERVSAVTEDCTDVEKVVRAKGRNIRIEKNPTLDSSKNKLNVTKASTTKRKRLVDSVPSLHSKVQKVANGSKQAASGQVAVQKMKMGTCYVHSKSLGSEVNASQWANGKGLSARTNRVKLRNLLAAAEGAELLKASQLKARKKRLRFQRSKIHDWGLVALEPIEAEDFVIEYVGELIRPRISDIRESHYEKMGIGSSYLFRLDDGYVVDATKRGGVARFINHSCEPNCYTKVISVEGQKKIFIYAKRHIAAGEEITYNYKFPLEEKKIPCNCGSKKCRGSLN

>AtSDG25

MVAVDSTFPSHGSSYSSRRKKVSALEPNYFGSMCMGVYSDDVSISAREVAQDYSCDSCGDLATVSSACCNFDELCGLDSALEMGCRSNEDCRAGQEASGSGIASGLDKSVPGYTMYASGWMYGNQQGQMCGPYTQQQLYDGLSTNFLPEDLLVYPIINGYTANSVPLKYFKQFPDHVATGFAYLQNGIISVAPSVTSFPPSSSNATVHQDEIQTEHATSATHLISHQTMPPQTSSNGSVLDQLTLNHEESNMLASFLSLGNEHACWFLVDGEGRNHGPHSILELFSWQQHGYVSDAALIRDGENKLRPITLASLIGVWRVKCGDANCDEPVTGVNFISEVSEELSVHLQSGIMKIARRALLDEIISSVISDFLKAKKSDEHLKSYPPTSAVESISSRVINAEKSVVSNTESAGCKNTMNEGGHSSIAAESSKYTKSVGSIENFQTSCSAVCRTLHHHCMQIMWNAVFYDTVATHSSCWRKNKIWFRSSDISTVNYCKGSHTKYSDKPESFESFTCRVDSSSSKTAYSDEFDLATNGARVRGLSSDTYGTESVIASISEHVENELFLSLKTHLTDYTSILIKDGANNTTSSARDGKMHEGSFREQYNLEGSSKKKNGLNVVPAKLRFSNDFSDSQRLLQEGESSEQITSEDIIANIFSTALETSDIPVNDELDALAIHEPPPPGCESNINMPCLRYKYQPVRSKESIPEIKAYVSMALCRQKLHNDVMRDWKSLFLKCYLNEFLASLKGSHQVSRKETLALKKRKTVTRNKKLVQSNISNQTAEKLRKPCVGASEKVLVKRSKKLSDSHSMKEVLKVDTPSIDLSVRKPSQQKMRNTDRRDHCIIKDATKLHKEKVGKDAFSKVICDKSQDLEMEDEFDDALLITRLRRISRNKTKELRECRNAAKSCEEISVTAEESEETVDCKDHEESLSNKPSQKVKKAHTSKLKRKNLSDARDEGTKSCNGAVKSFTEISGKEGDTESLGLAISDKVSHQNLSKRRKSKIALFLFPGFENTSRKCFTKLLSPEDAAKNGQDMSNPTGNPPRLAEGKKFVEKSACSISQKGRKSSQSSILKRKHQLDEKISNVPSRRRLSLSSTDSEDAVIKEDYDVRNEEKLPCHTSDKLQKGPNKLIRRRKPLAKHTTERSPIKDLSVDDGRPKPIALKPLEKLSSKPSKKKLFLSIPKSDGCARTSINGWHWHAWSLKASAEERARVRGSSCVHMQHFGSKSSLTQNVLSARTNRAKLRNLLAAADGADVLKMSQLKARKKHLRFQQSKIHDWGLVALEPIEAEDFVIEYVGELIRSSISEIRERQYEKMGIGSSYLFRLDDGYVLDATKRGGIARFINHSCEPNCYTKIISVEGKKKIFIYAKRHIDAGEEISYNYKFPLEDDKIPCNCGAPNVYCFCEQVPWIAKLKRRTWFSRRN

>AtSDG15

MATWNASSPAASPCSSRRRTKAPARRPSSESPPPRKMKSMAEIMAKSVPVVEQEEEEDEDSYSNVTCEKCGSGEGDDELLLCDKCDRGFHMKCLRPIVVRVPIGTWLCVDCSDQRPVRRLSQKKILHFFRIEKHTHQTDKLELSQEETRKRRRSCSLTVKKRRRKLLPLVPSEDPDQRLAQMGTLASALTALGIKYSDGLNYVPGMAPRSANQSKLEKGGMQVLCKEDLETLEQCQSMYRRGECPPLVVVFDPLEGYTVEADGPIKDLTFIAEYTGDVDYLKNREKDDCDSIMTLLLSEDPSKTLVICPDKFGNISRFINGINNHNPVAKKKQNCKCVRYSINGECRVLLVATRDISKGERLYYDYNGYEHEYPTHHFL

>LcSDG15

DYSNISCEQCGSGERPDELLLCDKCDRGFHMKCLRPIVARVPIGSWLCPSCSGQRRVRSFSQKKIIDFFKIQKCNAMEEKCASSQDAKKRRRRSASLVVQKKRRKLLPFIPSEDPVQRLKQMGTLASALTALQMEFSDDLTYMPGMAPRSANRAKFEDGGMQVLSKEDTETLEHCRAMSKRGECPPLVVVYDSCEGFTVEADGQIKDMTFIAEYNGDVDFLINREHDDCDGIMTLLLATDPSKSLVICPDKRGNIARFINGINNYTPESKKKQNCKCVRYSVNGECRVFLVATRDIAKGERLYYDYNGYEHEYPTHHFV

>SlSDG28

MAPSSSASPVAGLSRPVAQRKVHPSADYRRRPRMSVSPPPKKFRSMVEIMKVATRVELPEESEESEEEDDYEEVVCEQCGSGERPDELLLCDECNKGFHMLCLSPIVVRVPMKLWHCPHCSADQHRVIKSFSQKKIVDFFRIQKESQMVVKCSSAQDIKKRRKRSLVFHKRRRRLSLYIPTEDPHRKLVQMASLASALTALDMEFSDELTYMPGMARKSANSANFESGGMQVLSKEDTETLEQCRAMYKRGECPPLMVVFDSREGYTVEADGPIKDLTILAEYTGDVDYIRNRQEDDCDSMMTLLLARDPSKSLVICPDKRGNISRFINGINNHSPEGKKKQNLKCVRYSVKGACHVLLVTIRDIAKGERLYYDYNGYEHEYPTHHFV

>OsSDG730

MGPATPLRRRTRARPAATRAEGGSGGDGDDDDVRCEACGSGESAAELLLCDGCDRGLHIF

CLRPILPRVPAGDWFCPSCASPSPHSKKSHAAKKPKQFPLVQTKIVDFFKIQRGPAAALA

AAAESSEGKKRKRKVLPKEDVETLNLCKRMMARGEWPPLLVVYDPVEGFTVEADRFIKDL

TIITEYVGDVDYLTRREHDDGDSMMTLLSAATPSRSLVICPDKRSNIARFINGINNHTPD

GRKKQNLKCVRFDVGGECRVLLVANRDISKGERLYYDYNGSEHEYPTHHFV

>SlSDG27

MGSSTTVVLRRRTEAPKPGRRILRNRLNSRKMVEEDEYSDTSCVKCGSGEYPAQLLLCDKCDRGFHLFCLRPILASVPKGSWFCSSCDDNKNPTKLSLVQTKIVDFFRIERPSNSINECGPGKDCQKKRKRGSGLVMSKKRRRLLPFNPTKDPTRRLEQMTSLATALLAAGAEFSNELTYVPGMAPRSANHAALEREGMQVLSKDDTETLQLCKNMMKQGEWPPLMVVFDPKEGFTVEADAFIKDWTIITEYVGDVDYLNNREADDGDSMMTLLTTNDPSKDLVICPDKHSNIARFINGINNHTRAGKKKQNVKCVRFDVDGECRVLLVANRDIRKGERLYYDYNGYENEYPTAHFV

>AtSDG34

MVAVRRRRTQASNPRSEPPQHMSDHDSDSDWDTVCEECSSGKQPAKLLLCDKCDKGFHLFCLRPILVSVPKGSWFCPSCSKHQIPKSFPLIQTKIIDFFRIKRSPDSSQISSSSDSIGKKRKKTSLVMSKKKRRLLPYNPSNDPQRRLEQMASLATALRASNTKFSNELTYVSGKAPRSANQAAFEKGGMQVLSKEGVETLALCKKMMDLGECPPLMVVFDPYEGFTVEADRFIKDWTIITEYVGDVDYLSNREDDYDGDSMMTLLHASDPSQCLVICPDRRSNIARFISGINNHSPEGRKKQNLKCVRFNINGEARVLLVANRDISKGERLYYDYNGYEHEYPTEHFV

>LcSDG34

MLLKQRRTSAPNPDSGEYDDVCCEKCGSGDSPSELILCDKCDKGYHLFCLRPILVSVPKGFWFCPSCSTHKMPKSFPLVQTKIIDFFRIQRSTDLTQKPSQDHLRKRRRASGLVMSKKKRKLLAFNPTEDPKRRLQEMASLATALRASGSEFSNELTYVPGMAPKSANRATLERGGMQVLSKEDTETLNLCKSMMNRGQWPPLMVVYDPKEGFTVQADKFIRDLTIITEYVGDVDYLRNRENDDGDSTMTLLHAADPSQTLVICPYKHSNIARFISGINNHTTEGKKKQNLKCVRYNVNGECHVLLIANRDIAKGERLYYDYNGYEHEYPTEHFV

>LcSDG22

MGSIVPFQDLNLLPSPAITTTTLLTPKIEPKTEPFDEPIRTHHLQFHHNTPETFSSSSSSAAAAASTIPFFPNSQSTPVSQTPSSDQDNLYSEYYRISELFRSAFAKRLQHQYGDVEAALDSDSGAIVSVNQDNNQLSTNVVVPRRKYEKRSSELVRVQDLGADDTRYFRDLVRRTRMLYDSLRIMSVDDEEKRRGLGQGRRARGDLTAATVMRDHGLWLNRDKRIVGSIPGIQIGDLFLFRMELCVVGLHGQAQAGIDYLPGSQSSSGEPIATSIIVSGGYEDDEDGGDVLVYTGHGGQDKFNRQCEHQRLEGGNLALERSMHYGIEIRVIRGIKKYEGSVSSKVYVYDGLYKIHDCWFDVGKSGFGVYKYKLLRIDGQPEMGSAILKFAETLRTRPLSVRPKGYLSLDVSMKKEPIPVLVFNDIDNDHDPLYYEYLAKTVFPSFVYSQGSSGVGCGCVTGCTYGCLCAMKNGGEFAYDHNGSLLRGKPVVFECGPFCHCPPNCRNRVSQKGLRHRLEVFRSRETSWGLRSLDLIHAGDFICEYTGVVLTMEQAQIFAMNGDNLVYPNRFSARWAEWGDLSQVLSDYMPPSYPSIPPLDFAMDVSRMRNAACYISHSATPNVLVQFVLYDHNNLMFPHIMLFASENIPPLRELSLDYGVADEWTGKLAICN

>LcSDG6

MVKDLTVARRYIMQKLAVGMLNIIDQFHSEALMESARSVTVWKDFAMEASRCIGYSDLGMMLLKLQRMILQKYINSDWLQHSFESWVQQCQNVHSAESIELLKEMSASVVYSTFFGDSCILVLVCTFSTSMCSSYCWCDVESVAWLEETCSCGNQKAVAMVKILQELYDSILWNEVNSLWDAPMQPTLGSEWKTWKHEVMKWFSTSHPLPNGGDVEHRSGDGTMTISLQASRKRPKLEVRRAESHASQVENNDSNQPLAVEVDCELFSSQDTVNAAMFSSELAKQDDMKEVTTGTDTLSGVADRWDEIVVDAGNSDVIQTKDVELTPVSGEAKQSMDPLNQTKEVALIPVSEVGTKKSVDFGSKNRQCIAFIESKGRQCVRWANDGDVYCCVHLASRFMGSATKAEGTLSTDSPMCEGTTVLGTRCKHRALYGSSFCKKHRPRDDAEKSLYSPEITLKRKHKETISSLETSCRDIVLVGESESPLQVDPVSVMDGDSFQGRNILTGNPETEAQHCIGLYAQNNSNPCEESPRRHSLYCDKHLPSWLKRARNELGTHWMDNHKKEAQWFFRGYACAICLDSFTNKKVLESHVQERHHVQFVEQCMLLQCIPCGSHFGNSEALWLHVLSFHPVDFRMSKAAQQNNLSGGEDSPLKLEPGNSASLENNTGNLGSIRKFICRFCGLKFDLLPDLGRHHQAAHMGPNLVTSRAPKKGIRYYAYKLKSGRLSRPRFKKGLGAVSYRMRNRSAVSMKKRIQASKSLVMGAPIVQPQVTEGGTLGTLAESQCLVVSKLLFSKIRKTKPWPNNHDILSIARLACCKVSLKASLEEKYGVLPECLYLKAAKLCSENNIQVEWHQDGFICPNGCKIFKDPHLLSPLVPLPSDFTGKHSTDSHNHEKDEWEVDEGHYIIDSHHLGFRPLQNATLLSDDISSGKESVPVACIVDDGLLDTLCISADKSHSQSSRHSMPWESFTYVTKMLLDQHLNLDSEHSTCSPEMCDHVYLFDNDYEDAKDIYGRPMHGRFPYDQKGRIILEEGYVVYECNRMCSCNRTCPNRVLQNGVRVKLEVFKTENKGWAVRAGEMILRGTFVCEFIGEVLDEQEANKRRSRFTLHRMLRYGGDGCGYMFNVDAHINDMGRLIEGQVRYVIDATKYGSVSRFINHSCLPNLVNHQVLVDSMDCQRAHIGLYANRDKDVLAIVGLQIVGDAFTKSSIGTTIVTAAESIRNLKMPNISDDLVERISFDHLYS

>LcSDG9

MGLMDNFLHTESTRLVSLISGNHSEGRSGKLRMENGHCSLKIKRRKVSAVRDFPPGCGRCASWINSSANEEAVVSTVCLDAEKPVISSNDVNGANLMSAYPEGTLLPQTDILTALEPVISDVANILNGVNVDAPKEEMVPQKTSLPHDCPTAVLDVYNLENGVPKNYPPRRRVSAVRDFPPFCGRNASCLSKEECGKALISSKSLGKEASSIGNKSVEETLVTNVKQMEENFNDGDTYKSKLEGDVSGITGGNVQAEFEGHATVEMRKDDDYGTSSTNVIKVVQEDMRENIKSSCETKQYRFNSKTGAVIRSSNQDVGILEEDPLRDIVLYTDKKHRDRNCPIFSGTNDRFDKRDSEGLECQSNPIIVQGLMASPSCPWRQGKGNNLKSAGCTVEKHRKKRDLLPLCKSSLKSMNEAESSEGPLIKKKKSLTENTSLCMGQVFLRDNEDYYGHDGELGELMIKDTLEQDEEGISPLAIMDVDGVCKRQRSHSYNVNLPPSHPSTVKGPDSNATGSRSKVRETLRLFHAVCRKLLQEEEAKLKEQSSSRRIDLLAARILKDKGKHVNVGKQIMGSVPGVEVGDEFQYRIELNIIGLHRPTQGGIDYLKQNGKLLAMSIVASGGYDDDLDSSDVLTYTGQGGNVTKVGGKEPEDQKLERGNLALVNSMHAKNPVRVIRGEAKASDAARKYVYDGLYLVDRYWQEMGAHQKLVFKFKLVRIPGQPELAWKVVKKCKKSKVREGVCVDDISLGKELIPISAVNTIDDEKPPPFTYITGMMYPDWCCPIPPKGCDCTKRCSESGTCSCVIKNGGEIPYNHNGAIVEAKSLVYECGPSCKCPLSCYNRVSQHGIKFQLEIFKTESRGWGLRSLNSIPSGSFICEYVGELLEEKEAEKRTGNDEYLFDIGNNYNDSSLWNGLSSLMPDVHSSSCGVVEDVGFTIDAAQYGNVGRFINHSCSPNLYAQNVLFDHEDKRIPHIMLFAVENIPPLQELTYHYNYEVDKVYDSDGNIKKKNCYCGSPECTGRLY

>LcSDG19

MDQTFGHDSVPASGSFDKSRVLDVRPLRRLVPIFPSPSNMSSFPTPGAPFVCAPPSGPFPPGVAPFYPFFGSVDSSRPPNVHSNTISDAVPINSFRTPPAATAGARVNGDTGPSRSTRSRFQSQSQSGFTEDDGYDSQSLNMNDVEGTSNTRRSKKKPHKRTRGSGSGDIILASPEVDTDAILNNILSSFNLAEIDASRKTDGDKESVGFILLIFDLFRRKISQAEDTREVHGVSRRPDLRGGAILMNKGIRTNAKKRIGVVPGVEVGDIFFFRMELCLVGLHAPSMAGIDYMSLKVSQEEEPLAVSIVASGGYEDNAEDTDVLIYSGSGGNANKNNKEVMDQKLERGNLALEKSLHRGNEIRVTRGMRDAATPGYLSNQKHTRYGNRFSSGRTVSRIGVILPDLTSGAENIPVSLVNDVDGEKGPQYFTYLSSLKYSKPVNTVDSAAGCTCSGGCLPGNSNCPCIQKNDGYLPYTANGVIVDQKSLTHECGSSCLCPSNCRNRVSQGGLKVHLEVFKTSNKGWGLRSWDPIRGGAFVCEYAGEVIDMSKIEELRRENEDDYVFDATRIYQPVEPVPGGSEELPKIQSPLAISAKSVGNVARFMNHSCSPNVFWKPVLRENNKGYELHIAFYAIRHIPPMTELTYDYGRPDKAERRKHCQCGSSKCRGYFF

>LcSDG32

MEAIFPTLSWDFIRAGSFICEYAGEVVDMLKIGKLGGESEDYYVFDATRTRQLSEPLPVDSNEAPKIPFPLIINATAVRNVAHFMNHSCSPNVFWQPILRESNGDSDLHIAFQAIKQIPPVTELTYDYGLPDKAGRRKNCLCGSSKCRGYFY

>LcSDG13

MAPNPRVTSAFKAMKAIGISETKVKPVLKKLLKLYEKNWELIEAENYRVLADAIFDEEDEAKCSGQKKPQIDEENFEEEDQEEQPLLPLKRLRRGQEQRVPPSPSSSRPSLDGSSLKRPKVEGDELPATSSQNRSRDNVNFPRVGAGNGRTEIQQRGSKGKELVSAHVASTENRGIPKRSSQALSIREPAAERGNLLLPMKDSSKSSPKMSLLLMTYSINKGDSSSGNVSLEQVAQGLPASPHLDGNNRVDGVQASSSERRSSCELVAIPEENPSCLEIASSPKGEVKIFLSCNSAVGRSNFHMPSLDELRESMEERCLRSYKIIDPNFSVMNLLKNVCECFLELTTNSSCESQEKLGTVTPCDSLRKSTAQDVLDGRGKGNIYMSSGILRESVDNQCFADVVPPQVPRRLQFLNGRDKLVGTTEEMVANGSTISDEGNLESSGSHSLILVPQCQLTSDELRAINNLRDVTKGEEKVQIPWINEINTEHLPPFYYIAQNLVYQSASVNISLSQIGAEQCCSDCLGNCLAPTSAIACACATVRQAGKFVYTPDGLLDEAFLAECVSITRDPQQHSCLHCRDCPLERSENEGVLKPCKGHLERKIIRECWSKCGCCKQCGNRVVQRGINSKLQVFYTAEGKGWGLRTLEKLQKGAFVCEFVGEIVTIKELYDRDTRKHNCPVLLDAFWVPEGVSKEEAFCLDATNFGNVARFLNHRCFDANLIEIPVEIETPEHHYYHLAFFTTREVEALEELTWVRRFVIITF

>LcSDG31

MGKERALKALRATRALDLPDEEVCEVLQDLLEVYDWNWEPIEAEDYRALIDAYFEFKENQGVEDEKEGKMVHDGSKRPSKKLQIGVQADHASSAIVKSSKKLALEKAKMPLSTSAQGSMELSKLCSMDRRIESSSKLPDIQLNRELKDPTSTRLLSTDRKSTHNRVSHAVYFKQPLDAHGSSSTSKENSPASHCNRELIKSKTEKHCGDMARCSVPMSVVVTKNLLNIASSASGEVNLSLNCTPALANPNFQCPNFDAVLEYLEEYLRTEKLVSSQFSVKKLLKDLCDRYLELGKSYGGSIIKSSSPEVVIPDGESFSSRRLKKRASGIANSEMDLHKKGKNCCGILDSSNLVKIQQRTVTSDQKRLFHSIGDITRGTENVKISLVDEFGNEDLPKFRYMAQNTIYQNAYIHISLTRISDEDCCSSCLGDCLSSSIPCGCARETGGEFAYTQQGLLKEEFLDACMSMESGRNDQHLVYCQDCPLERSKNEHFPEPCQGHIIRKFIKECWTKCSCDVDCGNRIVQRGITCELQVFCTHEGKGWGLRTLRDLPKGTFVCEYVGEILTNTELYERNMQSGGRGRHTYPVTLDADWGSEKVLRDEEALCLDATFVGNVARFINHRCFDANLIDIPVEIETPDHHYYHLALFTTRKVNALEELTWDYGIDFSDHNHPIDAFKCCCGSAFCRDMMQNSKLAFYLVWKLVEFCIYILEKLSDGGAVKLRRPELFRFRDW

>LcSDG20

MSLVDEFGETVSGCDCEECVEVGDGCPCFSGMGEIVSECGPSCRCGFGCKKRLTRRGISVRLKIVRDVNKGWCLFADQLIPQGQFVCEYAGELLTTKEARRRQQIYDERASHVGVSSALLVIREHLPSGKACLRMNIDATRVGNVARFINHSCDGGNLSTMLVRSSGALLPRLCFFASKDIKEGEELTFSYGEIRPPATPPNVPGGVVEPVSAPAASPCGACKFLRRKCISGCIFAPHFGSDQGAARFAAVHKVFGASNVSKLLLHIPVNRRHDAVVTISYEAQARLSDPVYGCVSTILALQQQVASLQAELAMVETQLMNSRFAMANALQNSQQHLQQPQHPQHVAVLQPAYSNSSSASTSFMNNMSSFPSNFDLVADTAVPSSQSLEPLQMSQPAQDDEDDEEESRIPVGFANHIIHRR

>LcSDG18

MLERSSGWAQQHKARALEMKEYGKSSTGESVETLKFETTDNIRGKNLEIVGPQNRFGISSRRDVEVGQNNRHELPRAVLHSEAETPLIRRLRNRESRSLNTVENSNPSMLERSSGWAQQHKARALEMKEYGKSSTSESVETLKFETTDNIRGKNLEIVGPQNRFGISSRRDVEVGQNNRHELPRAVLHSDGSKILESKSEYMEDQVADNVSDSSQLDLVSSDNGEVKLTLVCDSSRQFDFRRLNVDAVLNLVEEKFRNQYAIENPEFSIRNVLKEFCQSLLEISNSVNNEMILLPMYDISKKSDSKVPMDNQKRETPTKDSKKNPTFIQDITLGEEKYPISLVNEDGSLELPKFRYIPKNTVYKNAYVNFSLARISEDNCCSKCFGDCLSDPANCACTRETRGEFAYRPGGLLQEKFLDECIAMNMRKTKKEHFYYCENCPLEHVVTSSSQKKRKRSSKKYMCKGHLMRKFIKECWVKCGCDYDCGNRIVQGGIKVKLQVFATPEGKGWGVRTLQDLEKGTFVCEYAGEIVTNMELDERNERRSLSDEQHTYPVLLDADWASERHLKDEDALCLDATFFGNVARFINHRCYDANLVEIPVEVETPDHHYYHIAFFTTRRVEAYEELTWDYGIDFDDETHPIKAFNCMCGSPFCGMKN

>LcSDG17

MVVQGQRRVSARIQEKQEEKQRLVKRRVELIGGVVEGGAGNDKENNVYARKRRKNGVDSKKEEDCKKQDESRNGDKEEERNDVVSVQTTSSPSPAVNELPNGSVVNLAEKSDTAKVKETLRLFNKHYLHLVQEEEKRCAKPAVDPKASKSKSKNAKAKKKDVPKEDAKAKAKRPDLKAITKMMENNEILFPEKRIGDLPGINVGYQFYSRAEMVAVGFHSHWLNGIDYMGTSYKRGDYTNYKFPIATAIVLSGMYEDDLDNAEDIIYTGQGGHNLTGNKRQRQDQVLARGNLALKNCVEQSVPVRVIRGHKSSSSYTKKVYTYDGLYKVIQYWAEKGISGFTVFKYRLRRLEGQPLLTTNQVFFTYGRVPQSVSEIRGLVCEDITGGFTYRKTLQVASNIKVPTNAVGCDCKGLCVDPRTCACAKLNGSDFPYVHRDGGRLIEPKDVVFECGPKCGCGPDCFNRTSQKGLMYRLEVFRTPKKGWAVRSWDFIPAGAPVCEYIGVLMRTEDVDNVSENDNNYIFDIDCLQTMKGLGGRERRLRDVSIPTIDCPGDQRSESLPEFCIDAGSTGNIARFINHSCEPNLFVQCILSSHHDLKLARVVLFAADNIPPLQELTYDYGYALDSVCGPDGKVKKMPCFCGAADCRTRLF

>LcSDG21

MTEVIPVTAGTDSKIPDHIHTDPTRFICIKGVTKVIQYWAEKGISGFTVFKYRLRRLEGQPLLTTNQVFFTYGRVPQSVSEIRGLVCEDITGGQENFPIPATNLVDDPPVAPTGKMNFQNLVPYWHLLLFVREFVYEMKRADLLLVSIFGFTYRKTLQVASNIKVPTNAVGCDCKGLCVDPRTCACAKLNGSDFPYVHRDGGRLIEPKDVVFECGPKCGCGPDCFNRTSQKGLMYRLEVFRTPKKGWAVRSWDFIPAGAPVCEYIGVLMRTEDVDNVSENDNNYIFDIDCLQTMKGLGGRERRLRDVSIPTIDCPEDQRSESLPEFCIDAGSTGNIARFINHSCEPNLFVQCILSSHHDLKLARVVLFAADNIPPLQELTYDYGYTLDSVCGPDGKVKKMPCFCGAADCRTRLF

>LcSDG33

MLVEFGTKDLMCFGRLIDFKERSADSGAAMHSGDGIGVNDISDGKRPGSSNDQKRPLATLNDQGRRVSPRLQNIPDCKRPYYGSSEKKPLGGFLQMYRDNVLRQFEEDENSNGMDMGPLPVEHGEKEIVNSEDDGLRCISYGNALVDKIGVGTFLKVKETLRLLNLHHLYHAKEEKRCENVEVLDLQIVNGSQFNISEKNKIFNPDKEFGPLPGIEVGRQFLSRAQISFAGFHEHWLFGNDCKRKSYSKLEEYDGNTFPIAMTILLSGQYEDVDDLLEVVYASQGGNDLSFIKTQVSDQAMHLGNLALKNNVDHHVSVRVIRRQDCAHSYTGKVYTYDGLFKVVKCWADEDVSEFTVFKYHLKRLQPRPKLALRDQVHTIRGKVSKVLYSLPGLVCKDISNGQEDICIPATNVIDNPPVAPSGKFNIAKILSQSNLCVTGFTYIKSVKVAANVVLPQISHGCNCKGSCTNRRSCSCAERNGSDFPYVRQDGGRLTEARDVVFECGPSCGCGPGCINRASQRGLKYQLEVYRTKDKGWAVRSLDFIPSGAPICEYTGVLRRTTELDDVSENDFIFEIDCLQTINEIGGRERRMCNVSVPKTDLAVMTTDDESENAPEFCIDAGQYGNVARFINHSCEPNLFVQCVLSSHHDVRLARVVLFASDDIPPMQELTYDYGYALDSVIGPDGKVKKMPCNCGFADLVLCEFEYRSVT

>LcSDG11

MDQNLGEDSVPASGSIDKTRVLDVVQLRRLVPVFPANAPFVFAPPSRPFLSGVAPFYPFFGSVGSQGQNEQNSQTPIGFPYKSVSSGNNNPISDAVTLNSFRSPPALTGGGNNVKVGVSKSKSQKRTKGTDDNKLTLSEEEVDKILNGILSSLNVMEFDQARKTDGDKDLVGYVLLILDFTDGDNEIRVIRGVKDVATSKGKIYVYDGLYKIQELWLDKGKSGVNVYNSKLVRLPGQPEAFMTWRLIDRWKNGVTPRMGVILPDLAFGAENIPVSLVNDVDDEKGPKHFTYFPSLKYSNMENSVERSVGCTC

>SlSDG1

MEQGFGSDPSGSTIDKTRVLDVKPLRCLSPVFPSASEMSSITTPQPSPFLCITPTGPFPSGVTPIFPFLSPDEPVRMGESSQQTPNQVPNQGTFGFGQPISPIPVNSFGNQTANGSSGHVNNVGDSGSGKKKGGPKKPRKVPPENAKKADGDKEVVRRILLVFDLFRRRMTQIDEPRYGAGSGRRPDLKASKMMMLKGMRTNQTKRIGNVPGIEVGDIFFFRMELCVVGLHAPTMSGIDYMSLKLTKDEEPLAVSIVSAGGYDDDGGDGDLLIYTGQGGVQRKDGQMFDQKLEKGNLALEKSVHRANDVRVIRGVKDVANPTGKIYIFDGLYKIQGSWEEKIKTGCNVFKYKLLRVPGQPEAFKVWKSIQQWRDGVVSRVGVILPDLTSGAESQAVCLVNDVDDEKGPAYFTYIPSLKYSKPFLTPRPSLGCQCIGGCQPDDTNCPCIQRNQGLLPYNSLGVLMTYKNLIHECGSACSCPANCRNRMSQGGPKVRMEVFKTKNKGWGLRSWDPIRGGCFICEYAGEVRDIGYDRDDNYIFDATRIYEPLEAVHDYNDESRKVPFPLVISSKNGGNIARFMNHSCSPNVYWQLVVRESNNEAYYHIAFFAIRHIPPLQELTFDYGMDKADHRRKKCLCGSFKCRGYFY

>SlSDG2

MEQGFGSDSVPPAGPIDKSKVLDVKPLRCLVPVFPSPNGMASGTTPQPSPFVCVPPSGPFPPGVSPFYPFLSPNESGRSAENQDGLGFGTPISPVPLNSFRTPAANGDTGPRRPGRPRASNGLAAEDDDSQNHSDQFGSGYSGHANDVEDTSTGKKRGRPRKTRLGQPSSGNPATPPIEVDVDPLLNQLLASFKLVEIDQVKKADGDKELSGRILLVYDLFRRRMTQIEERRGETPGSARRPDLKGANLLMTRGARTNQTKRIGNVPGVEVGDIFFFRMELCLVGLHAPSMAGIDYMSVRLTGDEEPIAVSIVSSGGYDDEGDDGEVLIYTGQGGVQRRDGQMFDQKLERGNLALEKSMHRGNEVRVIRGVVDVQNGGRGKIYMYDGLYRVQESWAEKSKLGNCSIFRYKLIRVPGQPEAYTLWKSVQQWREGTATRVGVILPDLTSGAESQPVCLVNDVDDEKGPAYFTYIPSLKYSKPFMKSNPSVGCQCLGGCQPGGTSCPCIQKNGGYLPFNPLGVLMSYKTLVYECGSACSCPPNCRNRITQAGPKARVEVFKTKNRGWGLRSWDPIRGGGFVCEYAGEVIEESRVGEFGNDGDDDYIFDATRMYEPLEAVRDYNDESKKVPYPLVISAKKGGNVARFMNHSCSPNVYWQLVVREINNETFYHVAFFAIRHIPPMQELTFDYGMVPPDKADRRRKKCLCGSLNCRGYFY

>SlSDG3

MEMGSVVGLGDVNFSTEPKTPTPTMIFPKIEPKLEPLDEFTPQSMNPNSNFSYNSGFRNTTTPQQQQLNATSSQTPSSIEAGVHSEYNRISELFQTAFAQSVQRDGDVEANEDLGCRAIVPVSNGSQVSDIVITRRKYEKRSSELVRVTDLKPEDVRYFRDLIRKTRMLYDSLRIFVNLEDENSQHLGSGRQTRARGDLKASQMMREHGLWLNRDKRTVGPIPGVLVGDLFLYRMELCVVGLHGTPQAGIDYLPANQSSNGEPIATSIIASGGYEDDEDAGDVIIYTGQGGQDKNSRQVVHQKLEGGNLALERSMYYGVEVRVIRGFKYVGSSSGKVYVYDGLYRITESWFDVGKSGFGVYKYKLVRIENQPDMGSAILRFAESLRTRPLEVRPMGYISLDISRKKENVPVFLFNDIDNERDPACYDYLLKTVFPPYVYQHVGNGSGCECTDGCGNGTNCFCAMKNGGQFAYDTNGILLRGKPIIFECGPHCSCPPTCLNRVSQKGVRNRFEVFRSRETDWGVRSLDLLQAGSFICEYTGVVLTQEQAQIFTMNGDSLIYPSHFAERWAEWGDLSRIDSNYARPAYPSIPPLDFAMDVSRMRNLACYMSHSSSPNVLVQPVLYDHNNVSFPHLMLFAMENIPPLRELSIDYGMPDDCTGKLAICN

>SlSDG4

MGSLVPFQDLNLQPESTNFTSSTTPNPRIIPKIEPKLEPLDEYTQADLQTPAFFSNPSPNFNTSSGSAFRRNPQLATHEADSQSPSSIIPEVPPGCDRNNVYVYSEYNRISEMFKEAFTEKMQRYGDVEVVGNQNQDSVDVVMEDADARAIVPVSNNDTQVAEVVVARRKYQQRSSELVRVTDLKVEDQLYFREAVRKTRMLYDSLRILAMVEDDGSQHLGPYRKPRGDLKACQILREHGLWMNRDKRIVGPIPGVLIGDVFFFRMELLVVGLHGQAQAGIDYVPASQSSNREPIATSVIVSGGYEDDQDGGDVIIYTGHGGQDKHSRQCVHQKLECGNLALERSMHYGIEVRVIRGFKYEGSGSASGKVYVYDGLYRIVECWFDVGKSGFGVYKYKLVRIENQEEMGSAILRFAQNLRIRPLEARPTGYVTLDISRKKENVPVFLFNDIDDNHDPAYFEYLVKPIYPPHVSLNVHSGNGCQCIDGCADNCFCAMRNGGQFAYDYNGILLRGKPLVFECGPHCRCPPTCRNRVTQKGLRNRFEVFRSRETGWGVRSLDLIQAGSFICEYTGVVLTREQAQIFTMNGDSLVYPSRFPDRWAEWGDLSQIYPNYERPAYPSIPPLDFAMDVSRMRNVACYISHSSSPNALVQPVLYDHNHVAFPHMMLFAMENIPPLKEISIDYGVADEWTGKLAICD

>SlSDG5

MVVPCVAELSDPVNDAMVPRRCSARIKKLKSEQEAQRERESQRVRCRSNDDSVLGKKTKVYKKSKLVTPSQAQTQAPNNDVTVATVDNDDVTITNVGAPIDCTDHPVPENSLNPQLSGNGTEKSSHARVTETLRIFNKHYLHFVQEEEIRCGRAQADQKTKKHSKSKEAEDDGKRSSKRPDLKAISKMISEKEVLNRERIGSLPGIDVGHQFFSRAEMVVAGFHNHWLNGIDCVGQSAGKKGEYKGYSLPLAVSIVVSGQYEDDQDNYEEVVYTGQGGNDLLGNKRQIKDQVMERGNLGLKNCMEQSVPVRVTRGHRCVNSYVGKVYTYDGLYKVVNYWAEKGISGFTVYKFRLKRIEGQPVLTTNQVHFTRGCTPNSISEIRGLVCEDISGGLEDIPIPATNLVDDPPAAPSGFTYSRDIVCAKGIKFPSAPTGCNCHGSCLDPRVCSCAKLNGSEFPYVHKDGGRLIEPKAVVFECGPNCGCGPACVNRTSQKGLRYRLEVFRTPNKGWGVRSWDYIPSGATICEYTGLLKKTDQIDPAADNNYVFDIDCLQTMKGLDGRERRLREVSLPGYWHNDSEKMSDGGPEYCIDAVSVGNVARFINHSCQPNLFVQCVLSTHHDIGLARVVLMAADNIPPLQELTYDYGYVLDSVMDREGKVKQMACYCGAADCRKRLF

>SlSDG6

MVSFSNDGLSDQCVKKRSSVNGYHLLDSGTMSKHKVRIVCGEQDLPPGCSRNAPKVDLNQNENAMVSISENMADTLVAHGDNGPNTGVEFCSVEVASARTTNVIENGLEEPTSHDKSLRFELSKDHKNSEMSLLKKAKVIGYDELGTEVDVARHFFLVENVIGMYKDHVLHPGSMTDRVIPVCDSKTLSLPQCQIKNGSVEDNISPLPKKKYCRRGVFAVRDFPPFCGRNAPKSTKLDLLGGNEASKRAILLNKGVTENEVIETSKNVMDTGTLSLGLTASREADSWSKTEVTGSKCSLIERATVRVEDPEDVQDNYVRRSQLERTVMLPETMTKKERDDTGKFLLKESIVYSRNEREKATTARHGFGSGDKITKPVVHGLMDERCSPWRQKKQTPRQIVQGLMAETNKDWRQKEQTRLDGLMSRNQVPKPSMYRQRMSVVVARKSIPKPKFPETLFGRSRSGFVGEAVPEYPSSPFSKNDGIRNLNCEAQPKDSPIGQKKCEFDETRPPFGPKSSSRCDARSKVLETLRLFQSHFRKILQGEESMSRSAGVNAKQKDKIRRIDLQAAKLVKDKGKQVNTGTQILGEVPGVEVGDAFQYRVELSLVGVHRLYQAGIDSMYIKGGLLVATSIVASGAYDDDLGDADELIYSGQGGNVVGKVKIPEDQKLVKGNLALKNSIRERNSVRVIRGSKEIRTPESGGRPNVVTTYVYDGLYTVENYWKEKGPHGKMVFMFKLVRIPGQPELTWKEVQSSKNSKARHGVCVPDITEGKESLPIAAVNTIDGEKPPPFKYIKNMMYPVGFRPAPPRGCDCIGRCSDAERCSCAVKNGGEIPYNRNGAIVEVKPLVYECGPHCKCPPSCYNRVSQHGIKIPLEIFKTDTRGWGVRALTSISSGTFICEYTGQLLEDTEAERRIGMDEYLFDIGQNYGGYTANSSGQANQNELVEEGGYTIDAARYGNVGRFINHSCSPNLYAQNVVYDHKDKRVPHIMLFAADNIPPLKELSYHYNYVVDQVYDSDGKIKVKRCFCGSSDCSGRMY

>SlSDG7

MASVSKDGLSNKSVKKRLLENGCHSSYLGIIPKYKIRKVSAVRDFPPGCGRTSLKVDLNHVQNAEVSTNIEDMTNIILVDGVKETNIEVKSQSVEVVNDLINLENQENVDRLAGEVMATNMSAIANGVGEKISDEKSTGFELPKDLKTSEMELSKETEDIQNDTSVKEVDEQGLPLVESINGGHMTQKLISVMEHTSTSPKNKYRKRRVSAVRDFPPFCGTKVPKSTEQNCFGVTEESKDVAGFGKAVTRNEVIETLREVTETGALPEKLIGSEDADSLKDRDVSSPKDRQLEQITMVRTEEQEGVQCDYDGRSQVERTVVMPEIMTKKGSDAGPVGKETLVYSENEREKLTSASSALGSGNEKQITKGAKPSGARKQGKQKSLDDPVSGNEIVVSQVESHLTKTAVNAFGSGHEIVKPIVQGLMAKPCCPWRQGEPTSLDCGNQVEKDDFSGRKKAKAVTRKSNPRGKKKSVTLGEATDGLSSALVVFNDKGPGLWATSNDGACSLNREAVHEDSPVRRGQCDFDVTLPPFGPNSSSHGDARTKVRETLRLFQGICRKLLQGEESKSKPEEAKSKQGPNRIDLHAAKIIKEKGKEVNTGQHILGEVPGVEVGDEFQYRVELAIVGVHRLYQAGIDYMKQGGMLIAISIVSSGVYDDGLEDADVLIYSGQGGNVVGKSKTPEDQKLERGNLALKNSISVKNPVRVIRGSKETKNSDSVDGKGKLVTTYVYDGLYTVENYWTEQGTKGKMVFMFKLVRVPGQPELAWKEVKSSRKSKVRHGVCVHDITDGKETFAISAVNTIDGEKPPPFNYIQKIIYPDWFQPSPFKGCDCIGRCSDSKKCSCAVKNGGEIPYNRNGAIVEVKPLVYECGPHCKCPPSCYNRVSQHGIKVPLEIFKTNSRGWGVRALTSIPSGTFICEYVGELLEDKEAEQRIGSDEYLFDIGQNYSDCSVNSSRQAEVSEVVEEGYTIDAAQYGNIGRFINHSCSPNLYAQSVLYDHEDKKMPHIMLFAADNIPPLAELSYHYNYSVDQVHDSKGNIKVKKCFCGSSECSGRMY

>SlSDG8

MVDTESPSTFKRLKIHATRNFPENCGPFVCQNNGSRKIYPEFPSNTKRVKVDSRRSFPENCGPQKRDGSDTQCSVDADNNSCSEVESAESCNFEATGNQPLKLKEENVIYDESTQHHQVQKQSTDTFDWFIKDEPIENGPAIVSQENLIDCQNDEPSKETCQSVHREEVSDDESRSWVDDDDISILTCSEWNSLTSALKDGKKGGKEGEIIHKCSDILEDFKPLPDIIRPEQQYESVFMKKQMDLGVPQENSRNSAVMCGVSGHGFSTEYEHIHEVKQVRKTLKLFDDVYTKLLQEDKAENPEGRSKRKIHIEAAMTLKNQKKWVNCEWTFGHVPGVQIGDRFRFRAELVMIGLHHQFMNGINYVNIGRKYVATSIVDSGRYDNEAISSETFIYVGQGGNPKVSINARVEDQKLKGGNLALKNSMDMGCPVRVICGRKRVNGEKSDIRYIYDGLYTVTKCWEEIAPTGKYVFKFELKRNPGQPKLNREVVSRPTSLGKVDHFHVNKATKSIMESEFVVDNDVSQGKEKIPICVVNAIDDERLPSFTYITSIRYPDWYYISKPQGCNCTSGCSDSEQCSCASRNGGEIPFNTRGSIIRAQPLVYECGPSCKCPPSCKNRVSQHGPRDHLEVFKTESRGWGLRSRDRVSSGSFICEYVGELLDEKEAESRIDNDEYLFDVGNYDEEIPKRNPMRNNNLKVESDSLGRKDEDGFALDAVRYGNVGRFINHSCSPNLYAQNVMYYHGDRRVPHIMFFASKSIAPFEEFTYHYNYGHVYDKNSNMKRKNCICGSQKCEGRMY

>SlSDG9

MLGKKKLHQMVDTKSPSTVKRLKVDATRNFPENCGPFVGENDGTGDKYPEFPSATKPVKVETTRNYPENCGPCVLQKKNGCDTQSSANVDIGSCSEVEMDVVELGDPLSVFVPKDMQFDLDATGVCEEEGGDSSHLNTSCQPVTNGNQVLTTKEVNLMYDDSTQLNEVLVNQILQKTSTDTGNTCDWFINGDPIENGPELPSEETNKGFQYKEVADDESTSRVDNSSCSQSNSQNSGLKTPSASKKGGKGEIVQEEAVKCPEPLHKCKVIFEHESVVRKKQIDIGVSPEDLRNSDVFCGASGNGLLMEHENIQKVKEVKETLKLFDDEYTKLLQEDKAKKHEGRSKRRIHIEAAMNLKKQKKWVNCEWTFGHVPGVQIGDQFRFRAELVAIGLHHQFIKGINYVTIGRKNVASSVVDSSRYDNEAISSETFIYVGQGGNPMVSLNGRVEDQKLEGGNLALKNSMDLGYPVRVICGRQRLNGEKSDTRYIYDGLYTVTKCWEERASTEKYIFKFELKRNLGQPKLNRELVSRPAKLVKVTHSCVNKSTKSVMQSEFVVDYDVSQGKEKIPIRVVNAIDDERLPPFTYITNMQYPDWYYISRPQGCNCTSGCSDSEQCSCASRNGGEIPFNTRGSIVRAQPLVYECGPSCKCPPSCKNRVSQHGPRYHLEVFKTESRGWGLRSRDHVSSGSFICEYVGELLDEKEAENRIDNDEYLFDIGNYDEEIPKRNVARNNNLKVDSNSSMRKDEDGFTLDAIRYGNVGRFINHSCSPNLYAQNVMYYHGDKKVPHIMFFASESIAPLKELTYHYNYHIDHVYDKNGDVKRKNCRCGSRKCEGRMY

>SlSDG10

MEVLPCSNLHYVPESDCPQQGSGTTLMYGGKPNHLEHAEQVQSGDVKVDDVLLNTKECQEEEADGRQFSVEGLPTADVIPTKEAYYDFGGDCQILSSDFHDSVDDNVVEHDHVTKSDLVRECLRPVVDTNEIGLPYSNQVVGSSSCESKWLDEDGPLAVWVKWRGLWQAGIRCARADWPLSTLKAKPTHERKKYLVIFFPRTRNYSWADVLLVRPISDFPHPIAYKTHKVGVKTVKDLTLGHRFIMQRLAISILNIIDQLHAEALEETARSVMVWKEFAMEVSRCKGYPDLGRMLLKFNDMILPLYKKSFSMESWIQHCQNADSAESIEMLKEELADSVRWDELNSLPNEGLHLDLNSQWKNCKSEVMKWFSVSHPVSDSGDVEQPNNDSPLKMELQQSRKRPKLEVRRAEAHALPVEFQVSHQAVPVGFDAGGLGGHDISKNVLLESEPTKDDISLGEAPRNGSPGSVADRWGEIIVQADNSDVIQMKDVELTPINGVSSNSFDHGSKNRQCMAFIESKGRQCVRWANDGDVYCCVHLASRFASTSIKVDASPHVDTPMCGGTTVLGTKCKHRALCGSPFCKKHRPRDENGLGSILPESKHKRKHEDNVLGLDTSNCKDIVLAGAFDAPLQVDPISVLRGESCYRNNLLEVPQYLQNRPSGSEMHCIGLWPHGSELCIESPKRHSLYCEKHLPSWLKRARNGKSRIISKEVFIELLKDCQSRDQRLYLHQACELFYRLLKSLLSLRNPVPKEVQFQWVISEASKDPMVGEFLMKLVCTEKQRLKSVWGFSASENAQASSYVKEPIPLLRITDNDQDHCDVIKCKICSETFPDEQVLGTHWMDSHKKEAQWLFRGYACAICLDSFTNKKVLETHVQERHHSQFVENCMLFQCIPCTSNFGNSEELWSHVLTAHPSSFRWSHTAQENHFPASEVASEKPDIGNSLSTQNFNSENQSGFRKFICRFCGLKFDLLPDLGRHHQAAHMGPNPVGSHISKKGIRLYAHKLKSGRLSRPKFKKGLGSVAYRIRNRNAQNMKRRILSSNSIISGKPSIQPSATEAAGLGRLGDPHCLDIAKILFAEIKRTKPRPSNSDILSIARITCCKVSLQASLEATYGILPERMYLKAAKLCSEHNILVSWHQDGFICPKGCRPVHDPFIVSSLLPLPGQVNRTGSIPPNSAISEWTMDECHYVIDSQQFKHEPSDKTILLCDDISFGQESVPITCVVEENLFASLHILADGSNGQITTSSLPWESFTYATKPLIDQSLDLAIGSSQLGCACPNSACSSQTCDHIYLFDNDYDDAKDIYGKPMRGRFPYDERGRIMLEEGYLIYECNQWCSCSKSCQNRVLQSGVRVKLEIYKTETRGWAVRAREAILRGTFVCEYVGEVLDEQEANKRRNRLSATEGCGYFLEIDAHINDMSRLIEGQSPYVIDATNYGNISRYINHSCSPNLVNYQVLVESMDHQLAHVGFYARRDILAGEELTYNYRYKLLPGEGSPCLCGSSNCRGRLY

>SlSDG11

MNKQQRTGESEKDDNGAGIFCRVAHLVLPYLEPAGLASVSATCNVLHVVSKAITSTRISDASRNLENYPIPFFNSVDSELYANFIYSPVQTLPTFPTIPWGGGSGRVKPDPFLVRVEGAYGCDCESCDLDSGSNCACVDFSELPTRECGPSCGCGLECGNRLTQKGISVKLKVVKDRRKGWSLCAAEFIPKGKFICEYTGELLTTEEARNRQWLYDKRTKSGHFSPALLVVKEHLPSGNACMRINIDATRIGNIARFINHSCDGGNLSTLIVRNSGALLPRVCFFSSRVILENEELAFSYGDTTVNSTGSQCFCSSACCSGILPADRGITSPIPKPHTYAI

>SlSDG12

MAPNSKSRVTKAFEAMKVFGYSETVVKPVLRNLLNLYNKNWKLIEDENYSVLLESIIDSEESKEKQKSSMEDEPEENEPPLKRSRLYSQGNHSSAAKHDAGPSVDTCTSELQPYGKQKMADITTESCETQDVEMKPRFLLNHHQRKGKKQISSEASPVSEEDNDIVVLSDDDKQETRILSSHLKLKKRGDTSRLYSAVKPKRRLAYSSSLEEPNVMGSTDVSKEGALVEYSFSDAMPLSDTLPGFDVPLAVVPSDLERLNSEHLGTEGNKEDATNSSRLNIASTPNGEVKLSFVYKIYSSSDFCPPSLDAVFKRMEEKYMKSYRFSQPGFLLSLMENLCKCYLTAGTRTRTANEPSAGIWSQKLHPVGVRYDATNHELHFAPDTSNGSFKLSNLIKILPQIPTFTASGNRDIMCYMVDFNGTRINGAEKDNTNKLLKLLASSTMNNSVLVQSEHSSPGLRNSVYYIEDISNGQEEHKISLINAFSHVLPVFKYIPKNVIFQNAYVKFLLARISDDSCCSNCSGDCLSQDIPCACAGETGGEFAYTSGGLLKEKFLESCISMSCEPQKHGLVYCQDCPLERSKNNSVSGLCKGHLVRKFIKECWHKCGCSRGCGNRVIQRGIAVPLQVFMTADGKGWGLRALEDLPRGAFVCEYVGEIVTNTELYERNTQTASERHTYPVLLDADWGSEGVLKDEEALCLDATYYGNIARFINHRCYEGNLIEIPVEVETPDHHYYHIAFFTTRKVNALEELTWDYGIDFTDHTHPVKAFKCCCGSKSCRDTGARKYTLMKITPH

>SlSDG13

MPVNPRVKKAFRAMKSIGISEEKVKPILKSLLKLYDKNWELIEEENYRALADAIFENEDAEVAEHKQPENNEVRALPLVQREEVLEEEAVYEEPERPLKRLRLRFQEGQASPSSNNSSAGTSLKRPRREEEGELSGPRYQNQLQGEANPSSVRKNLRLNETQTSPITSRGQSSVSAKSSHASKLKEPKTEPGGELSSKQKMSGSLALIKPKDEPYTDDMPLFEVPIAVIHPEPSNKGDTSSGNTSRSEPSAIDLRSVRDSGIMTSLNVMTTSRELIEVQDRCHVDGDIASSPSGEVKISISCDPALCRSSDFHMPSVESVLRMVELKCLKSYRIMDPNFSLMKLMKDMCECVLELGTQHSPELQSTKDVAAENDFGSRSMTVNSLNEGMNFEIDAGDAQPKIPPRSPPRIGEDCIQAGQIASMGNCGSTTGTDQNGIEQTNPWSMDAPCGLILGEIGSFDSLNELLNSDLGAGEAQPEIPHLNSYFGGDSTQADHTASTSNCGIAPDTSQSRLEEMVSCEATPRDVVSVEVIDITKGQENVVISLVNEVNSNQPPSFHYIASNVVFQNAYVNFSLARIGDDNSCSTCSGDCLSLSTPCACAHITGGDFAYTKEGLIKEEFLKECISMNRDPKKHCQLFCKVCPLERSKNEDIIEACKGHLVRNFIKECWWKCGCSKQCGNRVVQRGISHKLQVFMTPEGKGWGLRTLEDLPRGAFVCEYVGEVLTNIELFDRVARSPNGEEHSYPALLDADWGSEGVLKDEEALCLDATFYGNVARFINHRCFDSNLVEIPVEIETPDHHYYHLAFFTTRKIKAMEELTWVRLWY

>SlSDG14

MPSNPKVAKAFRAMKNIGISQEKVKPVLKDLLKLYDKNWELIEEENYRVLADAIFEKEEATESQKPENIDQEEVLEEEAADEEPERPLKRLRSRHQEVHSSSISAGTSFKKVEEQAELPGTNSQGCSLGPELNNRNAAAESQSVPCLTYVRKEGKQPVSPNSADRLENNANSRKNRLKGKETQTPQIISKEKGLVLGKASRASILKKPKTEPDEPHTVDMPQLEVPLAVIHPEPSNDKGSSNGNASRKQPDTSETSAAELRGGREADKDIPTFSNGLVTSHELVKPQNQCYSNIDVASSTFGEVKLSINCDAALGRSDFHLPSLEAVVKLVEDKCLKPFKTLDPNFSVPKLMKDMCECFLELGTQYNHELQETAKVDAENDIGYRSMALVSSNGSINLELDSGEDQPEKSQLPLPCNGHTNSAQTDQTTSVRNCGSVPEIDQNILEHLMSESPVALCGSKNLELDAGEAQPEKPQLHPCNSHNNSASTDQIASVENCGSAPEIDQNILDHVTFQSPVPLCESTQDETGSCVVTDITRGQEEVMISLVNEVNDKIPPSFNYIAHNVVFQNAYLNFSLARIGDDNSCSTCSGDCLSLSTPCACAYETGGNFAYTKEGLVIEELLKESISMNRDPKKHCQFFCKECPLERSKNEDIIEPCKGHLVRNFIKECWWKCRCDKQCGNRVVQRGISRKLQVFMTPDGKGWGLRTLEDLPRGAFICEYVGEVLTNAELFDRVSQSHNREEHSYPVLLDADWGSEGVLKDEDALCLDATFFGNVARFINHRCFDSNMVEIPVEIETPDHHYYHLAFFTTRKVKALEELTWDYGIDFDDHEHPVKAFKCQCGSKFCRNMKRPRRNRARKGW

>OsSDG717

MPHSRSDSGSGSRGADPCRGRKRGRLLMLEEEEEEEESGMEGCSAPACGDVRGDFVGWCS

DRHQVASCSGDQTQSASMFAAMQENACSIDSKGVVCPQSGLGYSAGQNGTHGGGGSMLHQ

NLEGCMYMNQLGQMCGPYPPEQLYDGLSTGFLHRDLAIYAVFGGKMANPVSLGSLKQFLS

QWSSDSVVATRDESVENKKMAPVNKLILPDNLSSEESCWMFEDAEGRRHGPHSLAELSYW

HHSSYLHDLSMIYHVDSKFGPFTLVSLIDWWSGGTEHSESSANDSGSLNALMDDVVEDIS

HQLHAGIMKSARKVFIDEIFSSVLPEMIACRKTEKQMAAKRKSQAAKTDNVSNKNALVLK

GKGDGTSTRPKSLNSYNNKVPEDPSVAVQSTAMQYEFADILSAVWETIYNKSMKSIWDEV

LYDPVMDYCDAWLKRKNESNLLSTVVPGASDNQKMQDTDEMSPKAICDSDAPESDMDFPP

GFGPNQESAEHSHSACVEYVTEKTDGRSGSSITLFSGPLGRVQVMLANELYVAAKEALFQ

HFEEVISEEITNCLCIGFEDDINQERIRTPVHAPEPSSPPGISVHETPSPAEMPRDEISD

MAEMARDEISDMAVDTIPCPADMAASGTSTVPEVTTDKLIIPYVEHQSPSASHASIFEKL

DAHEEAELDDSFDEVPPGTEAGLASLVIMEKNKYQPSKSVDSVLDIYRYTSWAFFRQILH

ESVMKEWASLFSGALSNCFDSWYARKNIVAKTMDDTLRPKEYTYYRKRKLRKNCEASSSE

KPMDEQLSRPLRDLVECKVNMKNIHRSSKAGISQRVSVVEKPSKKRAKPSHNDNINLNIQ

QDLKLLSDKVPKRNRSSHPTSKPLVSSKVPTEDRTTSAMPAKKRKQKNLATESNLKTKAV

ILSPESHGCEAPTEKRTTAIMP

>LcSDG28

MATLLSVSPSSSSSTFLSSTKTLKTLSFCTYKPLLPLKNKPFSATSLHSTTYSSPPQPEVQTFWEWLCDQGVVSAKSPVRPATVLEGLGLVARRDIRKNEVVLEIPKKFWINPDAVAASEIGNVCSGLKPWISVALFLIREKKREDSPWRVYLDILPQSTDSSVFWSEEELVELQGTQLLSTTLGVKEYVQNEFLKLDEEILRPHRELFPYHITLDDFLWAFGILKSRAFSRLRGQNLVLIPLADLINHSPNITTEDYAYEIKGAGLFSRDLLFSLRAPVAVKAGEQVFIQYDLNKSNAELALDYGFIESTSDRNTYTLTLEISESDPFFGDKLDIAESNGLGETAHFDIVLGRPLPPTMLPYLRLVALGGTDAFLLESIFRNTVWGHLDLPVSRANEELICRVVRDACKSALSGYHTTIEEDEKLLEGGNLQPRLKIAVGIRAGEKRVLQQIDGIFKDRELELDELEYYQERRLKDLGLVGEQGEIIFWE

>LcSDG3

MLLASRVTNLWCYKHPLSTVSRRVAVKLKFSSLSESKALNCVNDDDFLAWLESKAGAEISSVLSIGKSAYGRSLFASRKIQAGDCILKVPYNVQLAPDNLLPEIKPCIGDEVGNVAKLAIVVLVEQKMGQDSEWAPYISRLPQPGEMHNTIFWSEDELNMIRQSSLYQETVVKKVQIEQEFLKIMPVLERFPELFQDVTLKEFMHAYALVESRAWDSTKGLSLIPFADFLNHDKVSEAIVIYDEGKQLSKVIADRSYARNEEVLISYGELPNAALMLDFGFALPYNNHGEVQIQITVPHDDPLREMKLELLKGHRLPITKDSNDFKLSEDYFIIKEVKSARGKGKGLPQALRAFARVLCCTSPQELNDLAMEAAKSDGRLARRPLRNISQEVLAHQILLSKFTQLTEEYNASIESLEPVNSPLASKRLTLRKQMARDLLIGELRMLKSATAWLKNYCESLTPRVRHWRL

>LcSDG29

MEEEEESNKLKKLLQWAANLGVIDSSSSTTNQNPCCLSHSLTVSNFPVNGGRGLSALHDLTKGDLLLRVPKIALFTTQRLMEDDHHLSFVLLKYPSLSSTQRLSVCLLYEVGKGRSSWWYVYLVNLPRSYDVLAGFGEFEKQALQVDDAIWAAEKAVSKAKLEWKEAKELMGELKFKPQLLSFKAWIWASATVSSRTMHIPWDDAGCLCPVGDLFNYAAPGEQSNGSEDVEGRAPLSSSLPEVDTEDTLDSETCSADLQRLTDGRFEEDVNSYCFYARENYKKGEQVLLSYGTYTNLELLEHYGFLLDENPNDKVFISLEPGMYSFSSWPKESQYIHQNGKPSFALISTLRLWATPQHQRRSVGHLAYSGCQLSVDNEIFVMKWISNKCHAMLKNLPTSIDEDQLLLSTINKIQDFNTLNELEKVLSPFGSEVCTFLEANGLQRGKNSCELSLPWKTKMSIRRWKLAVQWRLSPYLKTQVHLLPQGHFRQSQKHQERHYK

>LcSDG27

MAASKVVVSSLTQIRPIKCAASYHTRVVPHPPDLLKWVKREGGFIHQAVKIAPQQPDNWGLGLLASHDIPKGSLLISLPLHIPLKFEADAVNRADSVLLNLARQVPEELWAMKLGLKLLQERANVGSFWWPYISNLPETYSVPIFFQGEDIKNLQYAPLLYQVNKRCRFLLDFDQVVRRTLECLESNDHPFGGQEVNASSLGWAMSAVSSRAFRLYGKKSADGTRNDIPMMLPLIDMCNHSFKPNAQILQEEDLGNENSQIKAETEIKQDNPLLLNYGCLSNDLFLLDYGFVIPSNPYDTIELKYDGVLLDAASMAAGISQLNFSSPAAWQQQILSQLNLVGEAPILKVTLGGPEIIEGRLLAALRVLLANDAETVQKHDLNTLKSLSAEAPLGIANEVASFRIIIALCAIALEHFPTKIMEDESLLKQGVSAASELAIQFRIQKKSLIIDVMRNLSRRMKLLSSKEAASAQG

>LcSDG36

MAAVDEKWSSHIASLLRPPSPVQDYFHQLVLDRNCSRIKVIQHDDSGKGVYADDDFEEGELVLKDPILVGMQHSSNKFDCLVCSFCFRFIGSIELQIGRKLYFQSIGVSPIAGGGCYDAGLSHMEEHSHLKIHEKLEDCVVSSSSSKDNVSLPKDVVDSLMNGEFVLPHSAEFPLPSPVSCPGGCKEAYYCSKLCAEADWNLSHSLLCTGERSESLSREALLKFIQHANGTNDIFLLAAKAISFTILRYRKLKADYLNEQQKCPSATALDCCVLSLLLEAWKPISFGHKRRWWDCIALPEAVDSSDEDSFRMEIREVANMSLQLLKAAIYDKECEPLFSLEIYGHIIGMFELNNLDLVVASPVEDYFLYIDDLPELQKKEAEIITRPILDALGDDYSACCEGMILICFAKAFKREKDRDGQATIIALRPIRKGEEVTISYIDEDLSYEERQASLADYGFKCRCPKCLEEQPEPV

>AtSDG36

MDSVYKTDENFAADVAALLAPLPTPQLQEYFNKLITSRRCNGIEVKNNGTIGKGVYANSEFDEDELILKDEILVGIQHSSNKVDCLVCSFCFRFIGSIEKQIGRKLYFKNLGVSGCCDDDSSEEDECVKYNGNEEQCGGSSSSHNTLPEGVVSSLMNGEMALPHTDKFPLPSPLSCPGGCQEAFYCSESCAAADWESSHSLLCTGERSESISREALGEFIKHANDTNDIFLLAAKAIAFTILRYRKLKAEHVDKKAKQSEPKQSLLLEAWKPVSIGYKRRWWDCIALPDDVDPTDEGAFRMQIKNLACTSLELLKIAIFDKECEALFSLEIYGNIIGMFELNNLDLVVASPVEDYFLYIDDLPDAEKEETEEITRPFLDALGDEYSDCCQGTAFFPLQSCMNHSCCPNAKAFKREEDRDGQAVIIALRRISKNEEVTISYIDEELPYKERQALLADYGFSCKCSKCLEDSSSI

>SlSDG39

MEIICPIDAQYSDQIAALLKPPPPLEVQKYFEELLATRQCDGIKVKPTPRYGKGVYAETDFKEEDLVLKDQMLAGAQHPSNKVDCLVCSYCFCFVGSIELQIGRKLYLEQLGVSPIDECHMQKDCYNSDSSVGEDDSDVEDQQVSGECASSPSKDKISLPKDVVESLFNGEMRLPYSEKFSMPPIVSCPGGCKENYYCSKSCAEADWESFHSLLCTGEGSKSLSTKALQKFIEHANDTNDIFLLAAKVISFTILRHKNLKESRHEGKGKQVISESIDFSLLGEAWKPVSMGYKRRWWDCIALPADVDGSDEASFRMQIKELALTSLQLLKEAIFDEECQPLFSLEIYGNIIGMFELNNLDLVVESPVEDYFLYIDDLPLSEKGEVEQTTKPILDALGDDYSICCQGTAFFPLQSCMNHSCRPNAKAFKREEDRDGQATIIALQPIAKGEEITISYIDEDLPFEERQALLADYGFRCGCSKCLEET

>OsSDG722

MAVSVFNTRRRTSLVSAFIDRTRMFISTPLPQVRDKVSRTKPPKPHGGGGERRRKKQPQE

AAARAGGGMGGSSASPCDLDRDFAPQIAQLLATPPLQPAQEYYNGLIQSRKHDGIRVNFS

SKHGKGVCANKEFAEGDLILKDQILVGAQHSLNKIDCAVCSYCFRFIGSIEFQIGRRLYW

QSVGSSSDCTNRRHCHESDVGSSASSSGATKENSSTLPEEVLGSLITGDMSLPFTDHFSL

PQVVPCRGCEEERYCSQSCADSDWETYHSLLCTGSKTEPSQRSALQKFIEHANGSNDIFL

VAAKAITFTLLRYKKLKTQPEFQNNTDESNFSLLMEAWKPLSMGYKKRWWDSVALPEDVD

SCDEDTFRQQIRDLALTIIIFCVSQSLQLLKDAIFDSDGLVVASPVEDYFIHIDDLPDDE

KCNISQEEAEKVTRPFLDALGEDYAAPCEGTAFFPLQSCMNHSCCPNAKAYKRDEDTDGN

AVIIALEPIKKDDEITISYIDEDVSYEERQAELADYGFICTCPRCQEEKPN

>SlSDG38

MEMRAKEAISIGQDLTPPIPPLSLCLHHSTLLSHCSSCFSPLPPPPSLHYPPFFSPKNPNSNHSIRYCSLQCSSLDSPIHFSSSEFHFFHLFPQPLYTNFPTSSDLRLSLRLLHLFQTLHLIQESNGSLLNLERIGGLMTNFRKVMFLEEHCNDNDLSGRIRDGAKALAASRRMRVGLETNGEYTVEAAVLCLVLTNAVEVYDKDGRSLGVGVYDVPFSWVNHSCSPNASYRFCTASDSGGILESRICPAATETGAAGIGHESISSNTELQKSMSVIGGSEACGPKIILRSIKGIQRSEEVLISYTDLLQPKVMRQSELWSKYRFSCCCKRCRSMPMTYMDHCLQEILILNLDSSNMATGDNFYEEHVMEKLIDCLDDAIDDFLSFNNPKNCCEKLEILLTQDHVNVLLKPDGEKLHQLFRLHPLHHVSLHAILTLASAYKVSVSELLALDPEGHEHQTKAFSLSRKSAAYSLLLAGATQHLLESESSLIVPVSNFWMTAGETLLSLVRSSTWNLLSMERHVEEFSFSSHQICGKCTLLDRFRDKFADCHDENAEFADVTSQFLSCVTDTTSKIWDFLTKEGGYLKVVEDPINFRWLGSRMPSFSQFATHATSPSADKTDSGLEAEDNHNEIRSS

>AtSDG41

MEIRAAEDIEIRTDLFPPLSPLASSLYDSFLSSHCSSCFSLLPPSPPQPLYCSAACSLTDSFTNSPQFPPEITPILPSDIRTSLHLLNSTAVDTSSSPHRLNNLLTNHHLLMADPSISVAIHHAANFIATVIRSNRKNTELEEAAICAVLTNAVEVHDSNGLALGIALYNSSFSWINHSCSPNSCYRFVNNRTSYHDVHVTNTETSSNLELQEQVCGTSLNSGNGNGPKLIVRSIKRIKSGEEITVSYIDLLQPTGLRQSDLWSKYRFMCNCGRCAASPPAYVDSILEGVLTLESEKTTVGHFDGSTNKDEAVGKMNDYIQEAIDDFLSDNIDPKTCCEMIESVLHHGIQFKEDSQPHCLRLHACHYVALNAYITLATAYRIRSIDSETGIVCDMSRISAAYSLFLAGVSHHLFCAERSFAISAAKFWKNAGELLFDLAPKLLMELSVESDVKCTKCLMLETSNSHRDIKEKSRQILSCVRDISQVTWSFLTRGCPYLEKFRSPVDFSLTRTNGEREESSKDQTVNVLLLSSHCLLYADLLTDLCYGQKSHLVSRFRL

>SlSDG31

MEELEEALSDKGLTVSSVPEKGRCLFTTRDFSPGEVIISEEPYVSVPNKSAKCEWCFTSSNLKRCSACQVVNYCGNTCQKSDWKLHRVECQVLSKVDKERVKSITPSIRLMVKLYLRRKLQDEKVIPITVMDNYNLVESLFVSIDMTGIDEKQLVLYAQMANLVNLILQCPKINVKEIAENFSKFSCNAHTICDAELKPLGTGLYPVVSIINHSCLPNSVLIFEGRMAVVRALHHIPKGTEVSISYIEMAGTTATRQKALKEQYLFSCTCIRCIKLGQNDDIQESAVLEGYRCKDKRCTGFMLRDSGNIGFTCQLCGLVRDKEEIKNTVHEIQSLSEKASISLPCGHNKDASVMYKMIEKLQLELYHASSINLMRTRENILKILMELQDWKEALKYCRLTIPAYRRVYPECHPLLGLQYYTCGKLEWWLGETEEAYRSLAKAAEVLRITHGTYTTFMKELFVKLEEARAELSYKISSKEE

>OsSDG716

MNLFLFFFSNLLERRGVGAGGMASWEEQLRDELAGRDLAVASVPGKGRGLFAARSFFPGE

VVISQEPYASTPNKISVGSNCDNCFASRNLRKCSVCRVAWYCGSACQREEWKLHQLECRA

IAALTEDRKKMLTPTIRLMVRLVLRRKLQDDKAIPSSGTDNYNLVDALESHRII

>LcSDG37

MEELQRSLEEGGLSVTNLPEKGRSLFTMRDFNSGEVIVSQEPYVCVPNNSHSQSRCDGCFASTSIKKCSGCQVVWYCGSACQKLDWKLHRLECQALSRLDKHQRKSITPSIRLMVKLNLRRKLQNEKVISTTAMDNYDLVEALVAHMSDIDENQLVLYAQMSNLVNLILQWPEINVKEITENFSKLACNAHTICDSELRPLGTGLYPVISIINHSCLPNAVLVFEGRLAVVSAVQHIPKGSEVLISYIETAGSTMTRQKALKQQYLFTCTCPRCIKVGQYDDIEESAVLEGYRCKDDACSGFLLRNSDDKGFTCQKCCLVRSKEEITTKASEIKALSDETLESASRGNQQEVLAVYKMIEKLQRTLCHPFSISLMQTREKLIKVKSLFHFFFLIGNKTLLIKQRSCKCESEVHKQSKQK

>AtSDG37

MADLQRFLQDRCLGVSNLPQKGRSLFTARDFRPGEVILSQKPYICVPNNTSSESRCDGCFKTNNLKKCSACQVVWYCGSSCQKSEWKLHRDECKALTRLEKEKRKFVTPTIRLMVRLYIKRNLQNEKVLPITTTDNYSLVEALVSHMSEIDEKQMLLYAQMANLVNLILQFPSVDLREIAENFSKFSCNAHSICDSELRPQGIGLFPLVSIINHSCSPNAVLVFEEQMAVVRAMDNISKDSEITISYIETAGSTLTRQKSLKEQYLFHCQCARCSNFGKPHDIEESAILEGYRCANEKCTGFLLRDPEEKGFVCQKCLLLRSKEEVKKLASDLKTVSEKAPTSPSAEDKQAAIELYKTIEKLQVKLYHSFSIPLMRTREKLLKMLMDVEIWREALNYCRLIVPVYQRVYPATHPLIGLQFYTQGKLEWLLGETKEAVSSLIKAFDILRISHGISTPFMKELSAKLEEARAEASYKQLALH

>LcSDG23

MCRWIGRLQLQKKQHQHVSFFSSTATHHHNQQPSPPPPPPIRVSITESAGRGVFATRRIGPGQLIHTAKPLLTHPSLSKLNTVCSFCLRNISPNTWNSQVHPHPLLFCSQLCKDDSQSFYDVESRADWSAFHDYCRSQGLKYPLLVKRLACMIISGAASADFVDILQPASLSTEMILQMEEGFGLLRNALTKAHIMDEQMTFLSKQWYASVLSRIHINAFRIELAGGLYEDLLSSAAASIEAEIAVGNAVYMLPSFYNHDCDPNAHIVWIENADARLKALRDVEEGEELRICYIDVSMGRNARQRLLAQGFGFQCNCARCLSGD

>AtSDG38

MSRLALNRYSRCFSRLKTLTTPLFFSSSAASNRDGDYQIGPPPIRVGLTESAGRAVFATRKIGAGDLIHTAKPVVACPSLLKLDSVCYLCLKKLMGSAKFEDRGVSYCSQECQENSKGFLDVETRADWSSFDDYCRTHNFKYPLMVKRLCCMIISGARPADCLDILQPAVLSSEMISKIEDGYGLLWNAFRKANFKDDDVAFLTKQWYTAILARIRINAFRIDLVGGSCGEDLLSLAAASVEGEGAVGHAVYMLPSFYNHDCDPNAHIIWLHNADARLNTLRDVEEGEELRICYIDASMGYEARQTILSQGFGFLCNCLRCQSTD

>AtSDG40

MDLEHQTMETFLRWAAEIGISDSIDSSRFRDSCLGHSLSVSDFPDAGGRGLGAARELKKGELVLKVPRKALMTTESIIAKDLKLSDAVNLHNSLSSTQILSVCLLYEMSKEKKSFWYPYLFHIPRDYDLLATFGNFEKQALQVEDAVWATEKATAKCQSEWKEAGSLMKELELKPKFRSFQAWLWASATISSRTLHVPWDSAGCLCPVGDLFNYDAPGDYSNTPQGPESANNVEEAGLVVETHSERLTDGGFEEDVNAYCLYARRNYQLGEQVLLCYGTYTNLELLEHYGFMLEENSNDKVFIPLETSLFSLASSWPKDSLYIHQDGKLSFALISTLRLWLIPQSQRDKSVMRLVYAGSQISVKNEILVMKWMSEKCGSVLRDLPTSVTEDTVLLHNIDKLQDPELRLEQKETEAFGSEVRAFLDANCLWDVTVLSGKPIEFSRKTSRMLSKWRWSVQWRLSYKRTLADCISYCNEKMNNLLGTQDRLRDL

>AtSDG42

MAISEEEAKLERFLDWLQVNGGELRGCNIKYSDSLKGFGIFASTSTQASDEVLLVVPLDLAITPMRVLQDPLLGPECQKMFEQGQVDDRFLMILFLTLERLRINSSWKPYLDMLPTRFGNPLWFSDDDILELKGTNLYHATELQKKKLLSLYHDKVEVLVTKLLILDGDSESKVSFEHFLWANSVFWSRALNIPLPHSFVFPQSQDDTGECTSTSESPETAPVNSNEEKGKSLTSAQPAPSVGSGDTIWVEGLVPGIDFCNHDLKPVATWEVDGIGSVSRVPFSMYLLSVAQRPIPKKEISISYGNKGNEELLYLYGFVIDNNPDDYLMIKEMLVNFVLTSVVTFNNGFIQVHYPVEAIPSIPFSDSKGQLLEAQNAQLRCLLPKSVLNHGFFPRTTSVIRESDEKETVRSCNFSWSGKRKMPTYMNKLVFPEDFMTGLRTIAMQEEEIYKVSAMLEELVESRQGEQPSETEVRMAVWEACGDSGALQLLVDLLNSKMMKLEENSGTEEQDARLLEEACVLESHEESRDLDGRRMSRNKWSSVVYRRGQKQLTRLLLKEAEHALHLALSSDH

>AtSDG43

MSASVAVVSGFLRIPSIQKSQNPSFLFSRPKKSLVRPISASSSELPENVRNFWKWLRDQGVVSGKSVAEPAVVPEGLGLVARRDIGRNEVVLEIPKRLWINPETVTASKIGPLCGGLKPWVSVALFLIREKYEEESSWRVYLDMLPQSTDSTVFWSEEELAELKGTQLLSTTLGVKEYVENEFLKLEQEILLPNKDLFSSRITLDDFIWAFGILKSRAFSRLRGQNLVLIPLADLINHNPAIKTEDYAYEIKGAGLFSRDLLFSLKSPVYVKAGEQVYIQYDLNKSNAELALDYGFVESNPKRNSYTLTIEIPESDPFFGDKLDIAESNKMGETGYFDIVDGQTLPAGMLQYLRLVALGGPDAFLLESIFNNTIWGHLELPVSRTNEELICRVVRDACKSALSGFDTTIEEDEKLLDKGKLEPRLEMALKIRIGEKRVLQQIDQIFKDRELELDILEYYQERRLKDLGLVGEQGDIIFWETK

>OsSDG741

MFHHLRRRLLCTAAAPPIRVALTESSGRGVFATRPISAGEVLHSAQPLVSHPSPPLIHEV

CYSCLRRKSGSGGGSSGSCYFCSDACREHAKGFHGVEKKADWSLFDDHCSSRGLKYPYMA

KRLACMVISGAVSADCLDILQPARLHQGTLTEMEEEFALLDSTFRKAGFQEEITTFLTKE

WYINVLARIRINAFRIELVASSYENLLSSAVASVSCDAAVGNAVYMLPSFYNHDCDPNTH

IVWLASADARLKALRNIEEGEELRICYIDASMDVDARQRILAEGFGFECRCQRCLSGD

>OsSDG731

MEALLRWAAELGVSDSPSAPSPSSCLGRSVLIADFPDAGGRGLAAARDLRRGELVLRAPR

AALLTSGRVMDDDPRIASSVASHLPRLSSVQTLIICLLSEVGKGKSSNWYLYLSQLPSYY

TILATFNDFETEALQVDEAIWVAQKALRGIRSDWEEATPLMKGLGFKPKLLMFKSWIWAF

ATVSSRTLHIAWDDAGCLCPIGDLFNYAAPNDDNSSTDEDRDDMMHQETNKMLDQTDFDS

SEKLTDGGYEDVNEYRLYARKRYRKGEQVLLAYGTYTNLELLEHYGFLLGENPNEKIYIP

LDLDLCMIGSWPRDSLYILPNGHPSFALLCALRLWTTPRNRRKALSHQIYSGSLLSVENE

LEILKWLVKKCKETLQQLPTTIEFDDNLLVLLCKLQNSTSCITEMNRSIFEQEFAPFFRF

HGFKLDCSIHSKLPVRLLRSLERWGLAVQWRCNYKRTLTKCIVHCKSLVHELSLQQNQQ

>SlSDG40

MSAPRFWIRDFKMEKAYQLLRSTFEDAGFTDEQIAFLSKKWYIDVLARIRINSFRIELALGSYEDILLSAAASVEAEAAVGNAIYMLTSFYNHDCDPNAHILWIESVNAKLKALRDIEAGEELRICYIDASMDHDARRATLSEGFGFDCRCARCMSND

>SlSDG43

MSSKMMLMANSLTHVRPLTCAAAAVYPSRLVAQPPDLIKWVKTEGGFVHKSIKVAQGDTFGLGLVASEDIPKGSDLIALPQHIPLKFDGSTSESENSHSALIKLAQHVPEELWAMKLGLKLLQERARKGSFWWPYISNLPETYSVPIFFPGEDIKNLQYAPLLYQVNKRCRFLLDFEKILKHELENLKPDDHPFSGQDVDSSALGWAMSAVSSRAFRLYGGKRPDGTRSNVPMMLPLIDMCNHSFDPNAEIVQEEANTNRNMLVKMVAGREIKQNDPLLLNYGCLSSDLFLLDYGFVIPSNPYDCIELKYDAALLDAASMAAGFTSPNFSSPSPWQQQILSHLNLDGPNSDLKVTLGGGELVEGRLLAALRVVLSNDEEAVKQHDLETLKSLTVEAPLGISTEVSALRTVVALCVIALGHFPTKIMEDKSLLKQNVSPTTELALQFRIQKKSLIVDVMRDLSKRVKLLLAK

>AtSDG39

MINDGGAKPETLLRVAEIGGRGRSLVAAQSLRAGQVILRESPLLLYSAFPFLSSSVSPYCDHCFRLLASSAHQKCQSCSLVSFCSPNCFASHTPWLCESLRRLHQSSSSAFSDQPSDRQVQARFLLSAYNLAAASPSDFQILLSLQGSGSSNGDPSCSAGDSAAAGFLHSLLSSVCPSLPVSISPDLTAALLSKDKVNAFGLMEPCSVSNEKRSVRAYGIYPKTSFFNHDCLPNACRFDYVDSASDGNTDIIIRMIHDVPEGREVCLSYFPVNMNYSSRQKRLLEDYGFKCDCDRCKVEFSWSEGEEDENEIMEEMEDQDEQEEMEDSVGENEEEVCGNGVDDESNFPHAYFFVRYMCEKENCFGTLAPLPPKTHDASRVLECNVCGSVKEDEVGVNQ

>OsSDG740

MAGDALRAADLPGRGRGLLAARSIREGEVILTEQPLLLYPASLASLPSFCSACFRSLSAA

ASPCPSCRAAGFCSPSCAAASHPRLLCTALSGGGGNGNLASAAEPHQEPLLFLLSAYSLP

EPSLRVLLSLSSAATPPPSDQDPGSLHAMVAALVPPQMLPPGFSPDLTAALLSKDRTNSF

SIMEPYRPEVPQPLRKARAYAVYPRASLLNHDCLPNACHFDYADRPGPGNTDIVVRALHD

ITEGREVCLSYFAANWQYKDRQQRLLEDYGFRCECERCQVESKWKQDDDSDGGDGDDTME

EEEEDGNGGEGGDDGMEQEEGDGGSDSDDDFPHSYFFVRYLCNHGECYGMLAPLPPLPNG

EPSHVFECNVCGNLKNEDEIDAPDGGDSSMAD

>SlSDG41

MENNQLLKVSEIEGRGRGIVATQPLKPGQIILKDSPLLLYSASVKNSTFCSNCFRVILQSPIPCSWCTSSFFCTSNCQSVALSSSHTPWVCQSLTHLKNTFSSHSLNVDQQIQAFFLISAYNLAVISPSSFRVLLSLQGDSSFVSESDVLLLHSLVATCPSLNLGEFGFSKELTAALLAKDKVNAFGLMEPFEVDRERGVRAYGIYPMASFFNHDCLPNACRFEYVDTDVNSRSNTDIVVRVIHDVPEGREICLSYFPVNFKYAERQQRLKEDYGFTCNCDRCVVEANWSDGEDDAMDKEGEESEEEEEEDEDMEEDMDDEVNVNGEVEERDQDFPHAYFFLRYMCNRENCGGTLAPLPSPSSVMECNVCGNLSKSDEL

>OsSDG739

MATPGLDDDSLQQLRSRATQLLLKENWTEYIAVCSLIIEAFDAAAACKDRRVLCSTLAHR

ADARARLGDAPGALADCDAALAADPAHPGALLSKGAVLRGLGRYSRAAECFRAALAVSGT

DEVREMVEQCKRLDAQARSGAVDLSEWVLAGFSGKCPDLAEHVGAVEVRRSAHGGRGVFA

VKNIEAGANLVISKAVAIGRGVIPDAADSGEKMVVWKDLVDKVLDAAEKCPRTASLIYTL

STGEEPEDELPIPDMAHFKQETEELDDGTAMAPKASLDVDKILKVLDVNCLTEDAAPSAN

LLGSNGVVNCGVGLWILPAFINHSCHPNARRTHVGDHAIVHASRDIKAGEEITFAYFDVL

TPASKRREAARAWGLECQCDRCRFEASDAIVGQELTKLENELVNGRGGDMGALVVRLEER

MRKSMVKERRKAFLRASFWSAYSALFDSDKLVRKWGRRVPGEAAVAESVAGAIGGNESVL

RAMLRGADNGNGCGNRLEVEDKVVRIGRATYGRVVKRQAMRALFRLTLDADSNKSL

>LcSDG35

MREVEQEPPVAEDAMQQLRSKAAELLLTEEWQESVQVYSQFINLCQAELLRTSNEADHVSKLHKSLCLAFSNRAEARFRLRDFNEALQDCEQALRIETTHFKALLCKGKILLSLNRYSMALDCFKATLLDAHASGNLEIVNGFVEKCKKLECQSRTGAFDFSDWVLSGFRGKCPELGEYVGAVQIKKSEISGRGLFATKNIDAGTLVLVTKAIASERGILSGGGDSSERAQLVMWKNFIDKIVECTSKCRRTRHLISTLSSGENEDELKVPDISLFRPEMEESECSNKKLDMDRILNILDVNSLVEEAVSAKVLGKNRGFHGVGLWLLASFINHSCHPNARRVHVGDFVVVHASRDVKAGEEITFAYFDMLSLLEKRKEMSQTWGFSCNCKRCKFEEGMSSKPELREIEMGLERGIDAGCAVYKLEENMKRWTVRGREKGYMRASIWSAYSEAYGSERLIKRWGRRIPAMEAVVDSVVEAVGSDERVLKVLMGGLKRSGCGVAEMERAMKFKRGLYGKVDVSFQSFSLLLNSLAILQAVVDSVVEAVGSDERVLKVLMGGLKRSGCGVAEMERAMKFKRGLYGKVVKKQALKSLLEMDIC

>SlSDG30

MEEEEDRIQNLRSKATELLLRKEWKDSIEVYTELISLCHDQISKPHQNLDPNNLPKLKKSLCLALCNRAEARLNLQDYPQALLDCNEASQIGNTHFKTLLCKGKILLSLNQYGLALDCFKKASLDPNELENSEMLNGYLEKCRKFEFLSRTGAFDISDWVLNKFQGKPPELAEYIGSIEIKKSDISGRGLFATKNLDCGSLLLVTKAVAVERAIVPESVFQDSKEQAQLDMWRNFIDRILESIKKCNRTRDLISKLSNGENEDDLEVPDIDLFRPEGEDSSTLHDKKIDKEKLLNILDVNSLVEELISAKVLGKNSDVHGIGLWILSSFINHSCDPNVRRSHVGDHLMIHASRDIKAGEELTFAYFDVFSPFRDREEKAKNWGFVCTCKRCNLEKGVCSNQEMMEIEMFLGKGLDNGGVVYRLEENMRRWMVRGKGKGYLRSSFWRVYSEVYESERLLRKWGSKVPLMDNVLDSVVDAVGSDERIVKLLMRKNGHKGNGIVEMEKAMKLGRGLYGKIMKKQTLRTILIQLGN

>AtSDG35

MRGEQFELEEDRDGPLELLQSLRSKATELLLREEWEESIKVYTEFIDLSRRQVSSTGGSDPDPDSIAKLRKSLCLALCNRAEARARLRDFLEAMRDCDQALEIEKTHFKTLLCKGKVLLGLSKYSLALECFKTALLDPQASDNLETVTVYIEKCKKLEFQAKTGAFDLSDWILSEFRGKCPELAEFIGSIEIKKSELSGRGLFATKNIVAGTLVLVTKAVAIERGILGNGECGEKAQLIMWKNFVEEVTESVRKCGRTRRVVSALSTGQGEDSLEIPEIALFRPDEAFETCGDWKQSLDTEKLLSILDVNSLVEDAVSGKVMGKNKEYYGVGLWTLASFINHSCIPNARRLHVGDYVIVHASRDIKTGEEISFAYFDVLSPLEKRKEMAESWGFCCGCSRCKFESVLYATNQEVREFEMGLERGVDAGNAVYMVEEGMKRWKVKGKDKGLLRASYWGVYDEIYNSERLMKRWGRKIPTMEVVVDSVSDVVGSDERLMKMAVEGMMKKHGGFSNIVEMEKIMKLGKGVYGKVVSKKKAMKTLLGIE

>SlSDG32

MFCVDDVSEASLVHKEDPYAAVILKKCRETHCHFCFNELPADAISCVSCSIPLYCSDWCQKQAGAPKFDRSSNSFSDLEGLADDLKNYMSDVVLAGASTLDTGHIAEHRHECQGFHWPLILPSEVVLAGRILVKVIEQNRHASADSNLVGFLDLSHNYVKLPPESKLEMHIYSIILLHCLQHVYRTELPISGIMVSKLVILLSQIQVNSMAVVRMQAPKVKGPIYEPGNALTSNLEQVKVGQAVYVAGSFFNHSCQPNIHTYFLSRTLHIQATEYVLAGSELELSYGPQVGQWDCKDRQRLLEDRYSFTCQCTGCSELNVSDLVINAYRCTKLNCLGVILDRTVTTCEKQKLKLLIDAPTVYSKQIVDAYAKETIKTIIKDMDDYYFGVLVDELKDISHKEQMTLVLRYINKNGELIENFLVLFMRKSGLKSLILQDAPSAYCIQSFVYQLQLTLLSRYKKHLGPFGVAQWFVSYCTDAEFYLVHTQRVEKLKDDNIAEVACHVFESDYKLEPQHCLVCDSYRDLESSCAAISQTENCCKRLQDAIASNEVPTNILRDAVKYTDLLRTIFHPYNKIIAEVEDNLAQAFCLVGELQAAIDHCKASIQILEKLYGANHIAIGNELIKLVSLQILVGDTAASGSISRITAILSRYYGSHADEIYPYLRHLKGRQDAD

>OsSDG723

MAAPRHHHLALAVALALLVVTAAAADEGGPRGRRVLVLVDDLAVRSSHSAFFASLQGRGF

DLDFRLADDPKLSLHRYGQYLYDGLVLFAPSTPRFGGSVDQNSILEFIDAGHDMILAADS

SASDLIRGIATECGVDFDEDPEAMVIDHINYAATDAEGDHTLIAGDDLIQSDVILGSKKI

EAPVLFRGIGHAVNPSNSLVLKVLSASPSAYSANPKSKLASPPSLTGSAISLVSVMQARN

NARVLISGSLDLFSNRFLKSGVQKAGSKIRHEKAGNEQFVTETSKWVFHERGHLKAVNVK

HNKVGETNEPGMYRINDDLEYSVEIYEWSGTSWKPYVADDVQVQFYMMSPYVLKTLSTDK

KGVFSTSFKVPDVYGVFQFKVEYQRLGYTGLSLSKQIPVRPYRHNEYERFITSAYPYYAA

SFSTMGAFFIFSFVYLYHK

>OsSDG738

MAMGMRARESVNMSEDLTQAIAPYATALHDASLQSHCSSCFHRIPAQSPHDMSCTMCGSV

RYCCSDCLISDCEVHSSSGECCFFVKHLREASPSTLTEETSDIRAALRLLYSLETRGLVS

SDSVSSSNRIGGLSASGIREVLEEGGEIAEGVLEGSLLMLSARKSRMKNYVGLSNGLTIE

KVALWAVMTNSVEVQISEEQSLGIAVYGPSFSWFNHSCCPSASYRFVLVPQNEGCTSNKP

ESCVVPVSKGAAPDAWHAWQNEEAGFAHAQCKYGPRVVVRCTKPINKGDEVFITYIDLLQ

TREARLSDLWSKYKFICSCELCTALPKPYVDLILNCDARNLKSPHNAVTDPAIEDLDNNL

QQAISEYSFLDDSKACCDVIESMLSENLMNDLQQEELSPRKYILHPLHHISVSSFMILAS

AYRCSAFKSSTDNLHGENCDFIFRMTKAAAAYSIVLAGATHHLFLSECSFVTLLSHFLLS

TGQSILDFAECIKGETRKNMPEAIFSFASCSTNSAKHDSVRYNQFRSTCEKFGKPLLSLS

LQCWPFLAQGLPCLEKIKNPIDFSWLGPAIFQAFQLSEEDSFNLSGKHAPATLIEQQKEC

ILSLAVCCITYSKYLASICYGPEHYLANRAKDLLECINHVQ

>LcSDG25

MEGLFGYSIGEASFVIMIGEIRVPDDRSGWDWPEALSAAAGLVKSSVSTSSMPSNSDLAKLVGSSGGLIQSGQPLEKIVFSKNTRTDQSFVVDALHELEQRYTQDSSNVLKGFVNSLQSNLGPSRCSTVSKFVDKGTNSSLQSVSGYIDSLVQSRNSSITHTSQNSRTVGKNYDVTKIENAYDWVVADRTEAPSNIELRLGQPYQHSQSSGNSVPSVIGPKLFETLVSSSKSLFLQQMIHNATNGEEKGESRQYFQCSTGLSDLSVRNEQSQLDLGSRVCGLSDAMDAATLEKFEGNVIKSSVVPLLSHLNSPPERAANCKADHNTVTGNRIVPKPLNSESHNPTGSGHNGSERQFNVSEFGFLRFADKGKSVGCTADGSFALPDSDFKIHKQLEKRGTSIVAMAASSSDPCFSAAHEKSHYFHQPSGVPPDAFDTRNHFHHSERVPYLGSGGHIDHTALKSMITPADSEKNLQSQVVSMAFPSAISTCVTGMLPAISKTEGIGASPYLLDDDMRLLALRQIVELSKQQHALSSVGMDIEKGRFGGLSNVNIQQPFSDDPLTFGEQRRGLNLSSQQNVSEVPMVSAPSGCFTRLGVNIEKAAPVPGKSENVTQSSECAKCCHGVPCRYFQGHCNYENYTKCLGCSESRVGSSPKSFNEQVQNVNEASVIVSSDFVKDQAGAQGTSTLLDQRGKLKGQLPKNVVCHSPQWRDVPSKFKSMSDVICLDQSPDMLDRRGHIESEVGDAAANGSCGTTKIPDSLKEQEMSNVSSGCSSAAAVTQASGQVNNMDSSTANTGNARYVNNHVLDEGSGIDKCWSSDDALESERSSEFLGADCKTKFSKEGSSKVTDDQPSRSLLDELKLINSMTWKKGRNQIHNRLIVHGKINLKKIERGFKTGKRKKARKLKMLEATSPAGDPSVHYECPRGTGTFEPFLSKDVEDPSVQETYTTGALSLHTASKRGRSTLSSSTEASRKRDLRMIYNDRDGDNDCQIELNRNTNLCKVHEFSGSKKVRRAWTSDCIRRSRVEELTHSDTRDTLRCKSVSSMKASSSHELNIGCSRARPVVCGKYGEICSGKLDEDVSRPAKIVPLSRVLKTAGSCTLPNSCKPKHSILNESMDFSDREADFDGSSYLKKEEKFGLRHASFCSEMNIDTTVKGGMLCSNGHKLSADELTILEKESDHKSRKGCSVLNGNVHSKLKLKTRKRSLFELTVKGKESCSGSSTLVKISNCMSKVKQQKTSKNAEGSKHQDHELCKVHIEKSNQEHRSLSVMDSEAFCCVCGGSNKDEINCLLECNRCFIKVHQACYGVSKVPKGHWYCRPCKTSSKDIVLCAVLSRLWVSCSDLSHTSPGRPNSDCLAICSHLRRPRWRSPLGQLTGQVICHWRCVNFKSQTQQVCVLCGYGGGAMTRALRSHMIVKSFLKAWNVESECKLKNAVSSAEIMESDLNVLHSSGSIHEGKFPVLRPFGIEPSTSVVCRVDSQKQADICQNSSFRVNSSRVHNSIMAGLLNSTVKQWVHMVCGLWTPGTRCPNVDTMSAFDVSGASLPKSNVVCSICDRPGGSCIQCRVVNCRVMFHPWCAHQKGLLQSEVEGVENENVGFYGRCLFHATYPLRESGSGPIDSKLESSGEKELTCARTEGYMGRKRDGFWYNLYGQSNAKSGCLVPQEQLNAWIHINGQKACTQGLPKLPPSDIESDCRKEYTRYRQSKGWKHLVVYKSGIHALGLYTSRFISRGEMVVEYVGEIVGQRVVDKRENEYQSGRKLQYKSACYFFRIDKEHIIDATCKGGIARFVNHSCLPNCVAKVITVRNEKKFELDTALE

>SlSDG42

MVSSTILIQQPTNFFHQPELHHQLWRGLQHGCVASLQKQPILVCNSNKRNRPLRVSSSANGAVTSSTLEAYDSSPSPSAFPLFTPPSQPQDTPASQLELADPDFYKIGYVRSFRAYGIEFREGPDGYGVFASKDVEPLRRARVIMEIPLELMLTISKKLPWMFFPDIIPVGHPVFDIINSTNPETDSDLRLACLLLYAFDCKDNFWQLYGDFLPSADECTSFLLATEEDLLELQDEKLASTMREQQNRALEFWEKNWHSAVPLKIKRLAQDPERFIWAMSIAQSRCISMQTRIGSLVQEANMLVPYADMMNHSFQPNCFFHWRFKDRMLEVMINAGQKIRKGDEMTVNYMAGQKNDLFMQRYGFSSPVNPWDVIHFTGDAKIHLDTFLSVFNISGLPGEYYHNSKLSNDGDRFVDGAIIAAARTLPTWSDGDLPPIPSLERKAVKELQEECHQMLAEFPTTSDEDQKILDSMPECRRTFEAAIKYRLHRKLLIEKVIQALDIYQDRILF

>LcPRMT6

MGSNTNGVVPAKHTATTTTTVDKGVDFAQYFCTYAFLYHQKEMLSDRVRMDAYFNSIFKNKHHFQGKTVLDVGTGSGILAIWSAQAGARKVYAVEATKMAEHARTLVAANNLQHVVEVIEGSMEEVTLPEKVDVIISEWMGYFLLRESMFDSVICARDRWLKPTGVMYPSHARMWMAPIRSGLGDQKMNDYEGSMDDWYGFVKETKTVYGVDMSVLTKPFLEEQKKYYLQTSMWSNLHPHQVIGTAAVVKEIDCSTATVNDILKIRSTIFSSITGESSRLCGFSGWFDVHFRGSREDPAQQEIELTTAPSIDNGTHWGQQVFLLHPPIRVSEGDDLNVSFSMNRSKENHRLMEIELSCEIRQSSGVVLPPFRNSVCIWKQDRYPYVLDSEIEGGLANLVAASVEDQMAAGR

>LcPRMT1

MGRRNNDNKKSNNSITPMNTITRFEDAEEETAVSSNPLTANDIVIEEDESMCDVDRSFVDGGDDDKTSADYYFDSYSHFGIHEEMLKDVVRTKTYQNVIYQNKFLFKDKVVLDVGAGTGILSLFCAKAGAAHVYAVECSDMADMAKQIVETNGFSDVVTVLKGKMEEIELPVAKVDLIISEWMGYFLLFENMLNTVLFARDKWLVDDGAVLPDKASLFLTAIEDAEYKDDKIEFWNNVYGFDMSCIKKQAMMEPLVDTVDQNQIVTNCQLLKTMDICNMAPGDASFTAPFKLVAQRNDYIHALVAYFDVSFTKCHKLMGFSTGPKSRATHWKQTVLYLEDVLTICEGETISGNMTVAPNKKNPRDIDIMLKYSLQGRRCAVSRTQYYKMR

>LcPRMT4

MEQKQKQAEFALASVTELSSSSSSSSSFSSPVFARFSVGSGVAELRFRHESESVDGFTADLRTAQLFKLGPVRSVCISEGSDTDKEKSYSKGVAIQFRDEEESMAFHCAFEQWKEESSVQGENLPNGVVTVCKKSKFDDKVEESSAKMYFHYYGQLLHQQNMLQDYVRTGTYYAAVIENRADFTGRVVVDVGAGSGILSLFAAQAGAKHVYAVEASEMAEYARKLIAGNPSLGQRITIIRGKIEEVELPEKADILISEPMGTLLVNERMLESYVIARDQFLVPNGKMFPTVGRIHMAPFSDEYLFVEIANKALFWQQQNYYGVDLTPLYGSAFQGYFSQPVVDAFDPRLLVAPAISHLIDFTKTKEEDLYEIDIPLRFISSVGTRVHGLACWFDVLFDGSTVQRWLTTAPGAPTTHWYQLRCVLSQPLYVMAGQEITGQLRMVAHSSQSYTIYLTLSAKMWGPGAEQGGIIQTSSCKLDLKEPYYRMSQPQAYTIAQDQQPHQLMAAQDIQIQADDLDETELMQQQPQNSGAQLQ

>LcPRMT3

MAINSETETLETRKIVVVVEEEEEEEEESSSSGGGDWGDWRDDNDGGEEAEDMDSESGFQCLFCDTKYSSCDALFEHCRSSHHFDFHTIKAALNLDFYGSFKLINYIRSQVADNRCWSCRLTYQSNQDLLHLHETINFKGVQLPWDDEKYLKPFMQEDKLLYSFGDDDEGEDDYITSFDKEELMKDLRHFEDICIDDENIVKFRLDTCTTYGNGKEISSTSKGLLNIASSSRKAIVNGLDSRGVGASEKKPNEIDSRVSLMNLAAKDIKRVNENYFGSYSSFGIHREMISDKVRTDAYRQAILENPSCLKGAVVMDVGCGTGILSLFAAQAGASRVIAVEASEKMAAVATQNAKDNGFWRTGPEGDGNEHSTGVMEVVHGMVEEVGKSLQIQPHSVDVLLSEWMGYCLLYESMLSSVLFARDQWLKPGGAILPDTATMFVAGFGRGATSLPFWEDVYGFNMSCIGKELVQDAARFPIVDIVADNDLVTDAVLLQTFDLATMKPDEVDFTASAELEPKLGGLESNSVESNSTTWCYGVVLWFETGFTSRFCKEKPVVLSTSPYGPKTHWSQTIFTFREPIAIASGKFRANKTAAIGTDTCPSGRIHLRVSIARAAEHRSIDISLETTGVGSDGRKCSWPVQIFNLS

>LcPRMT2

MYSNMGYTNGYHQGVSSHRDRVKRGNRRSRESLRVLEHQQQQQTERKPTTPCTDFDVAYFNSYAHVGIHEEMIKDRVRTETYRAAIMQHQSFIEGKVVVDVGCGTGILSIFCAQAGARRVYAVDASDIAVQANEVVKANNLSDKVIVLHGRVEDVEIDEEVDVIISEWMGYMLLYESMLGSVITARDRWLRRGGLILPSTATLYMAPVTHPDRYSESIDFWRNVYGIDMSAMMPLAKQCAFEEPCVETITGESILTWPHVVKHVDCYSIQIEELESVSTSFNFKSMMRAPLHGFAFWFDVEFIGPTISPSNNQLSVMTSSNNQPMDSCQKKKRANPNEALVLSTAPEDPPTHWQQTLIYFYDPIEVEQDQQIEGSVILSQSKENPRFMNIHLKYSSGGRSFVKESVMR

>LcPRMT7

MSTQRVFQLKFEPLTGNSEWIVIDENDDVPESSQEPLLATTSYLDMLNDSARNRAYRLAIDKTVTKPCHVLDIGAGTGLLSMMAARAMGSTDSAAQSNAKGTVTACESYLPMVKLLRKVLHVNGMGRNINVINKRSDEVEFGVDISSRADVLVSEILDSELLGEGLIPTLQHAHDFLLVKNPLVVPFRATTYGQLVESTVLWKLHDLHTNEAKASDGIHLAPTGLHTILHVKSQQYAMHCDAITKEIKLLSEPFKIFEFDFWKRLDSHGKIELQIKATNDGRVHAVVSWWVLQLDQEGTIFYSTAPRWITSSITTSTRDWCDHWKQCVWFVQGEGMSICKDEEVLLNACHTETSISYNLKPQVPRTKVTKDKLNAGDFQLLLPPERIAIYGDCEWRRCMLTAIRNALSQRAEPLCVVADDSVFLTICVAHLSKAAHVLSLFPGLGDKVVQYLEAVADANGFSMDRVEILQKRKMCLTIDDTHQKKVDLLIGEPYYYGNDGMLPWQNLRFWKERTMLDPILTKDVLIMPFKGMLKACAMSLPELWNSRRSLSKIEGFDHAVVNTTLGACGYLSAPKDGPCLPFFIWQCGEIKEVSEVFTVMEFDFSKPISQCHGKALVEFTKPGICHGFALWIDWVMDVENSIVISTGPDKKYWKQGVKLLASPVAVGTKASVQMGECCSTLIEASFDPTSGELNIQHTFS

>LcPRMT10

MPLGERGGWDKSESRYCGVETEFNDDMANVLSLNLSTGGFDFVVASLMDPSYRPSQSNKGDVLPFASSDLVLSPSQWSSHVVGKISSWIDLDSEDDILRMDSETTLKQEIAWATHLSLACLLPFPKGASCANYARCVNQILQGLNNMQLWLRIPLVKTDDDSVDSDQMIDSWEVWNSFRLLCEHHSQLSVALNVLSSLPSANSLGRWFGEPVRAAIINTDSFLTNARGYPCLSRRHQKLVTGFFNHSVQMVISGKSVHSLPGASSDSSANHTGSNANNAQRHPLRPYLDYVAYLYQRMDPLPEQERVELGYRDFLQSPLQPLMDNLEAQTYETFEKDCVKYIQYQRAISKALLDRVPDEEAFSLTTVLMVVGAGRGPLVRASLQAAEETGRKLKVYAVEKNPNAVVTLHSLVKLESWENIVTVISCDMRHWDAPEKADILVSELLGSFGDNELSPECLDGAQRFLKEDGISIPSSKACTKVNLWFTERLRNQPAEITEPTEHKNRTPFAVCPGPLLPDSGEQTPFVAKIELRRRASRYTSFIQPVTASKLYNDIKSHNDAVHFETAYVVKMHSVAKLAPSLPVFTFIHPNFSTKINNQRLRKLKFEIPSDTGSAIVHGFAGYFNAILYKDVHLGIEPSVATPNMFSWFAIFFPLRTPVCMQPGSSLEVNFWRCCGSTKVWYEWCVASPCPSPIHNSNGRSYWVGL

>LcPRMT11

MSLGERGRWDKSESIYCGVEMEFNDDMPNVLSLNLSTGGFDFMVASLMDPSYRPNQSNEGDVLPFASSNLVLSPSQWSSHVVVANNILIWSRKISSWIDLDSEYDILCMDSETTLKQEIAWATHLSLQACLLPSPKGASYANYARIPLVKTDDDSVDSDQMIDSWEVWNSFCLLCEHHSQLSVALNVLSSLPSANSLGRWFGEPVRAAIVNTDSFLTNARSYPCLLRRHQMLVTRFSNHSVQEKVYRVFSEQAQIHLPIILVVMPTLLMDNLEAQTYETFEKDCVKYIQYQRAISKALLDWVPDEEASSLTINHGLEKEKLRSELESTENKCSATDSFSIESSAFRGYIVLVASRLMVRSEFSNGVP

>LcPRMT5

MPLGEKGGWDKSKSRYCGVETEFNDDMPNILSLNLSTSGFDFVVASLMDPSYRPSQSNGGDVLPFASSDLVLSPSQWSSHIDSWEVWNSFCLLCEHHSQLSVALNVLSSLPSTNSLGRWFGEHVRAAIINTYIFVFVILSDLCQCS

>LcPRMT8

MDILNGLRGTKVQLNRECLAVRDEISLKDGIDKENALKVRTESCSPLSLEEDNDDDTGPNALENGSECCEDKNGRLVQVREVDRQDDISNTCMMNSVSVLGLSPDDIVGVIGEKQFWKARRTIINTIMTRFYVHFFINTIMTILSHPLMDNLEAQTYETFEKDCVKYIQCQRAISKALLDRVPDEEASSLTIISFPCPAYVIYTLS

>LcPRMT9

MRGGKDDENVTASPMFPRLHVNDTEKGGPTRLMDNLEAQTYETFEKDCVKYIQYQRAISKALLDQVPDEKASSLTIVLMVVGAGRGPLIRASLQLMRVENEEGFAWKYEAMGSGGGQDEDEDINIVDENVNDSLQYEAVGSGGGRDEDEDISMVNSNSFLYTKSEMVVDYKIDAEEFYKIGLMDCLPRKAVSGCDCRGVLQDMEEEEVEELDNKKDYKLQYEAVGSGGAREVVTTELLGRVRKRVAARQS

>SlPRMT1

MAMEELNTVYMEVEDDNSDDDKDQNWDDWENGDEEDEEEAMSSKLLCLFCDSTYDSSNALFEHCASEHRFHFNTLKNSLALDFYGCFKLINYVRSKVAGNKCWSCGIVCRSKEDLLNHLHEVASFDNGKLPWNDDEFLKPFLNEDALLYSFDEDDEGEDDMDNMPIDKEELIKDLEQISIDEDDFTLETEENKPTAFSLSGGKSTSLTNTTLSNGVVSAEGGVSSYKNNHDLDSSLYIAKVAANKIKDVNKNYFGGYSSYGIHRDMISDKVRTDAYRQAILENPSLLKGAVVMDVGCGTGILSLFAAQAGASRVIAVEASEKMAAVAAKIAKDNNLLRIGSKNEGSDQGNGVMEVVQGMVEELKSAQKVQPHSVDVLVSEWMGYCLLYESMLSSVLYARDQFLKPGGAVLPDMATMFAAGFGRGGTSIPFWENVYGFNMSCIGEEIVKDASRIPIVDVVDSRDIITNSKVLQNFDLVTMKLEEMDFTGMVELELKGETSANGSTGSKPVTNWCYGVVVWFETGFTERFCKEKPTNLSTSPHTTSTHWSQTILTFSEPIAMVSPGTLNVDKMAAVGSDACPAVKIQCRISVVRAAQHRSIDISMELSGIGPFGRKRNWPAQMFNV

>SlPRMT2

MLQPTDGGYTNGYHHDGAVAGGISGGHRVRRGSRGRPRGRRGGGGSSDGDARVSLGQQHNEDEEEREKPAPPCTEFDTAYFNSYAHVGIHEEMIKDRVRTDTYRNAIFQHQHFIAGKVVVDVGCGTGILSIFCAQAGARRVYAVDASDIAVQANEIVKANNLCDKVIVLHGRVEDVEIDEEVDIIVSEWMGYMLLYESMLGSVITARDRWLKPGGLILPSHATLYMAPVTHPDRYSESVDFWRNVYGIDMSAIMPLAKQCAFEEPSVETITVENVLTWPQVVKHVDCYKVTTQELESITSRFRFESMMRSPFHGFAFWFDVEFTGPRVFPLDNGMRPSFVEPSNSNMMEDNQRKKRPNPNDALVLSTAPEDPPTHWQQTLVYFYEPLDVEQDQVIEGSLTLSQSKENARFMNIHLAYSSGGRSFVKESVMR

>SlPRMT3

MEVSVKENEFVVLSISEVSSSSSTSFSKSEPVFARFRSGSGAPELRFGQESQSGADIVINLRGSQLFRLGPAESLFISEVFEDNKETGYSRGISIQFKNKEESRSFHCAFEQWKTEMVVQGPPLPNGAVSTSTSKFDEKIEASSAKMYFHYYGQLLHQQNMLQDFVRTGTYYAAVIENRADFLGRVVVDVGAGSGILSLFAAQAGAKHVYAIEASEMAEHAKKLIAGNPSLNERITVIKGKVEEVELPVKADILISEPMGTLLVNERMLESYVIARDRFLVPNGKMFPAVGRIHVAPFSDEYLYLEIANKAIFWQQQSYFGVDLTPLQRSAYEGYFSQPVVDAFDPRLLVAPAISHAINFTLIKEEDLYEIEIPLKFACSVSTRIHGLACWFDVLFNGSTVPRWLTTAPGAPTTHWYQLRCVLSQPLYVMPGQEITGNFRLVAHRAQSYTIYLTLSATVGDMLQTSSGKLDLKEPYYRMSQPQAYSSAQEPNQLLQTQDVLTQSWVEDGLVPVQQPSPNHGVELQTL

>SlPRMT4

MQCLFPKTLNPQFFLRSFNNTRSKAFTVRSMSSGSRMFQLKVDPLTGNSEWVVIEEDEASGDATKQLLANTSYLDMLNDTRRNKAYREAIDKTITKPCHVLDIGAGTGLLSMMAARAMDLGDSVESSGSKGMVTACESYLPMVKLMRKVLHANGMQRKIRIINKRSDELEVGVDMPSRADVLVSEILDSELLGEGLIPTLQHAHDQLLVDNPKTVPYRATVYGQVFDHGFIFKYSFADYLWKLHDLYNTEKEVLDEICLVPEGMDSALCVKRQQFSMHCDALEEDIKLLSEPFKVFDFDFWRRPDSHRVTKLSVQATDTGAVHAVISWWLLQLDEKGTIFYSTAPKWISCPSSVEGFNSSISCEWRSQNWCDHWKQCVWFIPKKGLSLLKDEEVSLLAVHTDTSISYEMKTLSQNLELEQSEVSAQKYQITLLPEKIALYSDVNWRCSMLKAIKNAMKQKTPSLCVVVDDSIFLAVALAHLAKGSHVLSLFPGLQEKGALYLQAVATANGYSKDHVEVQKMSELLTSQSSQEKIDLLVGEPFYYGNNSVLPWQNLRFWKDRSLLDSILSEGAVIMPCKGLLKACAMSLPDLWQSHQCLQHVEGFDHSVVNSTLGACGGLPPGQENPTLPFSVWQCGESKKMSDIVTIMEFNFLKTISPCSGKAKVEFISHGKCHGFVLWIDWVMDAEESIVLSTGPEQRYWKQGVKLMKEPVAVGSHRSATTDCHSADIETSFDPSTGDLIVEYAFL

>SlPRMT5

MDCRSNYENQNSGSTSSKMKFEYEDESSNIEVEEDTSMCDPIDESIVGSNKTSADYYFDSYSHFGIHEEMLKDVVRTKTYQNVIYKNSFLIKDKVVLDVGAGTGILSLFCAKVGAKHVYAIECSSMADMAQEIVKLNGFSDVITVIKGKVEEIDFPVRKVDIIISEWMGYFLLYENMLDTVLYARDKWLVKDGLVLPDKASLYLTAIEDADYKDEKIEFWNSVYGFDMSCLQKQTIMEPLVDNVDQKQIVTNCQLLKTMDISKMASGDASFTVPFKLVAERDDYIHALVAYFDVSFTKCHKLTGFSTGPRSRGTHWKQTVLYLEDVLTVCQGEAVVGSMTVASNKKNPRDLDIMLKYSVNGQRCSVSRTQCYRMR

>SlPRMT6

MTLGERQGDEKNDSKYCGVETEFNDDMPQLLSLNIHGGFDFVVAPLMDPAYRPSLLVSSNGGSGVLPFAGSDLVLSPSQWSSHVVGKISSWLDLDSEDEMFRRDSEITLKQEIAWASHLSLQACLLPAPKGVTCANYARCVNQILQNLSNMQLWLRIPLEKSDDDEDRSPNSMGEEHRDSWEMWNSFRTLGEHHSQLSVALDILSSLPSVNSLARWFGEPVRAAIINSNSFLTNARGYPCLSKRHQNLLTDFFNHSIQIVISGQQMQNFPTGTSVSNSHSSNNQSEGVEGMQRHPLRSYLDYIAYLYQKMDPLPEQERFELGYRDYLQSPLQPLMDNLEAQTYETFEKDTTKYIQYQRAVAKALVDRVPDEKASTITTVLMVVGAGRGPLVRASLQAAEETGRKLKVYAVEKNPNAIVTLHSLLKIEGWEKLVTIVSSDMRCWDAPEKADILVSELLGSFGDNELSPECLDGAQRFLKEDGISIPSSYTSFFQPVTASKLYNDIKSHKDLVHFETAYVVKFHRVARLTSPQPVFTFNHPEDSNRKSNHRYTKLRFEIPTDTGSALVHGFAGYFDAVLYKDVHLGIEPSTATPNMFSWFPIFFPLRTPMCVQPGIPLEVHIWRCCGISKVWYEWGVTSPSSSPIHNCNGRSYWVGL

>SlPRMT7

MGSSSNAAAPINNVDKGVDYANYFCTYAFLYHQKEMLSDRVRMDAYYNAIFQNKHHFAGKAVLDVGTGSGILALWSAQAGARKVYAVEATKMAEHARELVKTNGFEHVVEVIEGSMEDITLPEKVDVIISEWMGYFLLRESMFDSVICARDRWLNPDGVMYPSHARMWVAPIRSGLVDQKKIDYDRAMDDWSHFVNETKTFYGVDMGSLTKPFTDEQRKYYLQTSLWNNLHPNQVIGKPAVIKEIDCLTSSVNDLLSLQANISSIITAENTRFCGFGGWFDVHFRGRKENPAKNEIELTTAPSEDLGTHWGQQVFLFYPSTRVSQGDNMTMNFSMNRSKENHRLLEVEFDCELRQSSGKSLPSFSKKFYIE

>SlPRMT8

MDSVSNNEIENSASYGKNQSKIKFQYEDDDEQVVQDEAVTESSNLEVDEDISMCDPTTTAAAVDESMVGGDKTSADYYFDSYSHFGIHEEMLKDVVRTKTYQNVIYKNSFLFKDKVVLDVGAGTGILSLFCAKVGAKHVYAIECSSMADTAQEIVKLNGFSDVITVIKGKVEEIDLPVPQVDIIISEWMGYFLLYENMLDTVLYARDKWLVKDGVVLPDKASLYLTAIEDADYKEDKIEFWNSVYGFDMSCIRKQAMMEPIVDTVDQNQIVTNCQLLKTMDISKMTSGDASFTAPFKLIAERDDYIHALVAYFDVSFTKCHKLLGFSTGPKSRSTHWKQTVLYLEDVITVCHGEAVVGSMTVAPNKKNPRDVDIMLKYSVNGKHCRVSRTQYYRMR

>SlPRMT9

MEENQKQQQFLVSSVSELTSSSSSLFSKTEPVFARFSLDSGLPELRFGQGAELSDAVVFNVKISQLFKLGPVESLCVSEANKEKSHSRGISIQFRNEEESRAFHCAFEQWKKEVVVEECSLNNGAVSTSKSKFDDKIEASSAKMYFHYYGQLLHQQNMLQDFVRTGTYYSAVIENRADFLGRIVVDVGAGSGILSLFAAQAGAKHVYAIEASEMADYARQLIAGNPSLNDRITVIKGKVEDVELPEKADILISEPMGTLLVNERMLESYVIARDRFLVQNGKMFPGVGRIHMAPFSDEYLYMEIANKATFWQQQNYFGVDLTPLHGSAYQGYFSQPVVDAFDSRLLVAPAVSHVINFSSVKEEDLYEIDVPLRFLSTVSTRIHGLACWFDVLFNGSTVQRWLTTAPGAPTTHWYQLRCVLPQPLYVMPGQEITGRLHLVAHKAQSYTIYLTLSALVGDMLQTSSVKLDLKEPYYRMSQPQSYSAAQDQNPSQLLQSDMQFPSRDDDGSILMQPPSPNELHSL

>AtPRMT1B

MTKNSNHDENEFISFEPNQNTKIRFEDADEDEVAEGSGVAGEETPQDESMFDAGESADTAEVTDDTTSADYYFDSYSHFGIHEEMLKDVVRTKTYQNVIYQNKFLIKDKIVLDVGAGTGILSLFCAKAGAAHVYAVECSQMADMAKEIVKANGFSDVITVLKGKIEEIELPTPKVDVIISEWMGYFLLFENMLDSVLYARDKWLVEGGVVLPDKASLHLTAIEDSEYKEDKIEFWNSVYGFDMSCIKKKAMMEPLVDTVDQNQIVTDSRLLKTMDISKMSSGDASFTAPFKLVAQRNDYIHALVAYFDVSFTMCHKLLGFSTGPKSRATHWKQTVLYLEDVLTICEGETITGTMSVSPNKKNPRDIDIKLSYSLNGQHCKISRTQHYKMR

>AtPRMT1A

MTSTENNNNGSDETQTTKLHFEDADESMHDGDDNNADVADDITSADYYFDSYSHFGIHEEMLKDVVRTKSYQDVIYKNKFLIKDKIVLDVGAGTGILSLFCAKAGAAHVYAVECSQMADTAKEIVKSNGFSDVITVLKGKIEEIELPVPKVDVIISEWMGYFLLYENMLDTVLYARNKWLVDGGIVLPDKASLYVTAIEDAHYKDDKVEFWDDVYGFDMSCIKRRAITEPLVDTVDGNQIVTDSKLLKTMDISKMAAGDASFTAPFKLVAQRNDHIHALVAYFDVSFTMCHKKMGFSTGPKSRATHWKQTVLYLEDVLTICEGETITGSMTIAQNKKNPRDVDIKLSYSLNGQHCNISRTHFYKMR

>AtPRMT4B

MEVSSVKKLEQLEYSLESVTDLSSSSVSSSSPAVATFSYVDGVTELRFLQSDSTHCFNFDLASAQLFKLGPVHFICVSDGSSSSEEKSFSKGVNIKFKNEKDSKDFCESFEEWRNDSVVQGSSLQNGTVSANKSKFDNKIEASSAKMYFHYYGQLLHQQNMLQDYVRTGTYYAAVMENHSDFAGRVVVDVGAGSGILSMFAAQAGAKHVYAVEASEMAEYARKLIAGNPLFADRITVIKGKVEDIELPEKADILISEPMGTLLVNERMLESYVIARDRFMTPKGKMFPTVGRIHMAPFSDEFLFIEMANKAMFWQQQNYYGVDLTPLYGSAHQGYFSQPVVDAFDPRLLVASPMFHMIDFTQMKEEDFYEIDIPLKFTASMCTRMHGLACWFDVLFDGSTVQRWLTTAPGAPTTHWYQIRCVLSQPIYVMAGQEITGRLHLIAHSAQSYTIDLTLSAKMWGPGASQGGILQSSTCKFDLKEPYYRMSQPQAYPVAQEPPLQPQPELSTQQDIQTPNDELEEELLQQLPQNPSAQL

>AtPRMT4A

MEIPSLNKQQEFTLASVTDLTSPSSSLSSSPVVATFSCVNEVKELRFQESKSSDGFSFDLSSTQLFKLGPLQFTCVSDGSISSAKEKSSFSRGVVIKFRDEKDSKEFCDSFEECKKDDAVKQGSALPNGTVVSANKSKFDDKIEAASAKMYFHYYGQLLHQQNMLQDYVRTGTYHAAVMENRSDFSGRVVVDVGAGSGILSMFAALAGAKHVYAVEASEMAEYARKLIAGNPLLAERITVIKGKIEDIELPEKADVLISEPMGTLLVNERMLETYVIARDRFLSPNGKMFPTVGRIHMAPFADEFLFVEMANKALFWQQQNYYGVDLTPLYVSAHQGYFSQPVVDAFDPRLLVAPSMFHVIDFTMMTEEQFYEIDIPLKFTASVCTRIHGLACWFDVLFDGSTVQRWFTTAPGAPTTHWYQIRCVLSQPIHVMAGQEITGRLHLIAHSAQSYTINLTLSAKMWGPGANQGGILQTSSCKLDLKEPYYRMSQPQVYPTQEPPAQSQDIHIHSDDLEELELLQQNANAQL

>AtPRMT5

MPLGERGGWERTESRYCGVETDFSNDVTHLLNFNISTGGFDYVLAPLVDPSYRPSLVEGNGVDTQVLPVCGSDLVLSPSQWSSHVVGKISSWIDLDSEDEVLRMDSETTLKQEIAWATHLSLQACLLPTPKGKSCANYARCVNQILQGLTTLQLWLRVPLVKSEGDSMDDTSEGLNDSWELWNSFRLLCEHDSKLSVALDVLSTLPSETSLGRWMGESVRAAILSTDAFLTNARGYPCLSKRHQKLIAGFFDHAAQVVICGKPVHNLQKPLDSSSEGTEKNPLRIYLDYVAYLFQKMESLSEQERIELGYRDFLQAPLQPLMDNLEAQTYETFERDSVKYIQYQRAVEKALVDRVPDEKASELTTVLMVVGAGRGPLVRASLQAAEETDRKLKVYAVEKNPNAVVTLHNLVKMEGWEDVVTIISCDMRFWNAPEQADILVSELLGSFGDNELSPECLDGAQRFLKPDGISIPSSYTSFIQPITASKLYNDVKAHKDLAHFETAYVVKLHSVAKLAPSQSVFTFTHPNFSTKVNNQRYKKLQFSLPSDAGSALVHGFAGYFDSVLYKDVHLGIEPTTATPNMFSWFPIFFPLRKPVEVHPDTPLEVHFWRCCGSSKVWYEWSVSSPTPSPMHNTNGRSYWVGL

>AtPRMT7

MSPLSSLPPKTFISSFHCHSVTRLRRSVTARTMSSQSSQRVFQLRQDPLTGNSEWIVIEDNDQPGTSTDGLLATTSYLDMLNDSRRNIAYRLAIEKTITEPCHVLDIGAGTGLLSMMAVRAMRGDSKGMVTACESYLPMVKLMRKVMHKNGMTKNINLINKRSDELKVGSEDIASRADVLVSEILDSELLGEGLIPSLQHAHDMLLVDNPKTVPYRATTYCQLVESTFLCNLQDLRNNEAKTSDGVRLVPPGLESLFGIKSQQYSMHVDAIEKEIKLLSEPVKIFEFDFWKRPESNGELDVHIEAKTTGSVHAIISWWVLQLDSEGTIFYSTAPRWIDSNSEIGVRDWCDHWKQCVWFTPGTGVSISKGEKVHLHASHTCTNILYNLKKTQSLTHERTHFPLSTGDLHLTLPPERVAIYGDSIYRQSLFEATRKALQGKSYPQCLVIDDSLLLPLMALHISNRSRVLSLSPGLQENAARYFEAIADSNGFSKDRFEYFRDGKTNLAKAYPGKIDLLIGEPYYSGLENGLPWQNLRFWKDRTLLDSVLSEDAVVMPYKGVLRGCAMYLPDLWKSRCCLGSVEGFDHTLVNTTLGGCGDLPSGKDSPCLPFFIWQCGETKILSKEFTVMEFDFSKPITGPCSGEVQIEFIKPGVCHGIALWMDWVMDEENSTVISTGPDDKYWKQGVKLLGKPVTVRMEGPSSSIGIQASLDLSSNSELIVTHTIS

>AtPRMT3

MAATMVKHEILNYSEDEEENYSDEGDWGDWKADDNGIEGGEEEEEDDGDDSESDFLCLFCDSHFVSCDLLFEHCRLSHGFDFHGVRKELKLDFYSSFKLINYIRSQVAENMCFSWKIEADDYKDVKFPWDEEKYLKPFWQEDSLLYSFADDEEDEEVTFDREEVMEELQKLGDLSIDVEALGESSMSNSDKCNINGSKDVTSLSNCNGLKQSSADDLIVNGKDAEPKVCDGRLVNRNIRKVNENYFGSYSSFGIHREMLSDKVRTEAYRDALLKNPTLLNGSVVMDVGCGTGILSLFAAKAGASRVVAVEASEKMAKVATKIAKDNKVFNDNEHNGVLEVAHSMVEELDKSIQIQPHSVDVLVSEWMGYCLLYESMLSSVLYARDRWLKPGGAILPDTATMFVAGFGKGATSLPFWEDVYGFDMSSIGKEIHDDTTRLPIVDVIAERDLVTQPTLLQTFDLATMKPDEVDFTATATLEPTESEAKTRLCHGVVLWFDTGFTSRFCKENPTVLSTSPYTPPTHWAQTILTFQEPISVAPASVLSGNDRREAIGTEECPASSIHLRVSVARAHEHRSIDISLEATGLSSKGQKRHWPVQIFNL

>OsPRMT702

MASPDLFPNVSFSHVSVPAAAGASTEVTGGATAVFGGDASTGAPRLSLVWSGETQAKHTL

EIDLSDAQIFKLGPTEWLCVSGESEAKDGVEEKSYSRAIKVVLRTEAESKAFYLAFQQWK

HRVISGKAGEPLENGLIIGSKSKFDTKIEASSAKMYFHYYGQLLHQQNMLQDFVRTGTYY

AAVMENRSDFEGRVVVDVGAGSGILSLFAAQAGARHVYAVEASEMAEHAQRLISGNPSLG

QRITVIKGKVEEVELPEKADILISEPMGTLLVNERMLESYVIARDRFLVPGGKMFPTTGR

IHMAPFSDEYLYVEMANKALFWQQHNFFGVDLTPLHGSAFQGYFSQPVVDAFDPRLLVSP

PTFHTLDFTTMKEEELYEIDIPLNFVASVGTRVHGLACWFDVLFNGSTVQRWLTTAPGSP

TTHWYQLRCILSQPLYVMAGQEITGRLHLVAHSAQSYTIYLTMSAKMWGEGAEQGGILQT

STAKLELKEPYYRLSQPQPYVMQQDQQQQQLPSLQPQSPLWDYHYGQD

>OsPRMT703

MDQRKGSGSDANGGLAEATASRLRFEDPDEVMEENPAAAAATVGAEEEGGEGGGGEEVIG

SDKTSADYYFDSYSHFGIHEEMLKDVVRTKSYQNVITQNSFLFKDKIVLDVGAGTGILSL

FCAKAGAKHVYAIECSQMADMAKEIVKTNGYSNVITVIKGKVEEIELPVPKVDVIISEWM

GYFLLFENMLNTVLYARDKWLADGGVVLPDKASLHLTAIEDAEYKEDKIEFWNNVYGFDM

RCIKKQAMMEPLVDTVDANQIVTNCQLLKTMDISKMTPGDASFTVPFKLVAERNDYIHAL

VAYFNVSFTKCHKMMGFSTGPRSKATHWKQTVLYLEDVLTICEGETITGSMTVTPNKKNP

RDIDIKLCYALSGHRCQVSRTQHYKMR

>OsPRMT708

MPLGQRAGDKSESRYCGVEVLDFPAGEELPAVLSHSLSSSFDFLLAPLVDPDYRPTPGSV

LPVAASDLVLGPAQWSSHIVGKISEWIDLDAEDEQLRLDSEITLKQEIAWASHLSLQACV

LPPPKRSSCANYARVVNHILQGLTNLQLWLRIPLEKSEPMDEDHDGAKDNSDMSDTVDSW

EWWNSFRLLCEHSSQLCVALDVLSTLPSMNSLGRWFGEPVRAAILQTNAFLTNARGYPCL

SKRHQKLLTGFFNHSVQVIISGRSNHNVSQGGVLSGDENHTEDTAVRHALSPYLDYIAYI

YQRMDPLPEQERFEINYRDFLQSPLQPLMDNLEAQTYETFEKDTVKYTQYQRAIAKALVD

RVSDDDVSTTKTVLMVVGAGRGPLVRASLQAAEETGRKLKVYAVEKNPNAVITLHSLIKL

EGWESLVTIISSDMRCWEAPEKADILVSELLGSFGDNELSPECLDGAQRFLKPDGISIPS

SYTSFIEPITASKLHNDIKAHKDIAHFETAYVVKLHRIARLAPTQSVFTFDHPNPSPNAS

NQRYTKLKFEIPQETGSCLVHGFAGYFDAVLYKDVHLGIEPNTATPNMFSWFPIFFPLRK

PIYVPSKTPIEVHFWRCCGATKVWYEWAVTAPSPSPIHNSNGRSYWVGL

>OsPRMT709

MPSCCCLLGLGFPSPPSALRILRRRMASRAFQLRLNPLTGDSEWLVVEEEEEEDHHPTPP

PKQLLATTSYLDMLNDSARNRAYRRAIEAAVTDPSSRVLDIGAGTGLLSMMAARALAAVG

GETRGGSVSACESYLPMGKLMRRVLRANGMENRVKVFHKRSDELKVRDDLDSPADILVSE

ILDSELLGEGLIPTLQQAYDMLLAKNPKIVPYRATTYGQLVESTFLWKLHDLHNNEANAA

DGVWLTPGEMERIVSVKPQQHAMQCDALEDEIRLLSEPFKVFEFDFWKRPDSHREANIKI

RTTRDGYVHAIISWWVLQLDSAGSIFYSTAPRWARQSSSEGPQRDMKDWCDHWKQCVWFM

QGKGIPATEDQVLSLRARHNQTSISYQLNINDEACDRSSKGDHLTLLPERIALYGDKDWR

SALINTIKNALTVKSSPTCVVADDSMFLALLISSMSPTSKVIAMYPGLRDKGAAYLRSVA

DANNFSIDQIQVIGKRASSITADDLKHKKVNLLVGEPFYLGSEGMLPWQNLRFWSVRTLL

DSMLSEDAFIMPCKGILKLCAMSLPDLWRSRSSLKDVEGFDHSVVNETLGACGCLPGDQQ

GPCLPYYVWQCGYTKKLSKVYSLMDFNFSEPIHSCFGKTKIEFSHDGTCHGFAVWIDWVL

DERKSVVLTTGPDNRYWKQGVQLFSKPVEVNPGKSVMHVEASFDPSTGEITFSSSSTTCS

>OsPRMT710 MAATMVKHEILNYSEDEEENYSDEGDWGDWKADDNGIEGGEEEEEDDGDDSESDFLCLFCDSHFVSCDLLFEHCRLSHGFDFHGVRKELKLDFYSSFKLINYIRSQVAENMCFSWKIEADDYKDVKFPWDEEKYLKPFWQEDSLLYSFADDEEDEEVTFDREEVMEELQKLGDLSIDVEALGESSMSNSDKCNINGSKDVTSLSNCNGLKQSSADDLIVNGKDAEPKVCDGRLVNRNIRKVNENYFGSYSSFGIHREMLSDKVRTEAYRDALLKNPTLLNGSVVMDVGCGTGILSLFAAKAGASRVVAVEASEKMAKVATKIAKDNKVFNDNEHNGVLEVAHSMVEELDKSIQIQPHSVDVLVSEWMGYCLLYESMLSSVLYARDRWLKPGGAILPDTATMFVAGFGKGATSLPFWEDVYGFDMSSIGKEIHDDTTRLPIVDVIAERDLVTQPTLLQTFDLATMKPDEVDFTATATLEPTESEAKTRLCHGVVLWFDTGFTSRFCKENPTVLSTSPYTPPTHWAQTILTFQEPISVAPASVLSGNDRREAIGTEECPASSIHLRVSVARAHEHRSIDISLEATGLSSKGQKRHWPVQIFNL

>AtSDG3/SUVH2

MSTLLPFPDLNLMPDSQSSTAGTTAGDTVVTGKLEVKSEPIEEWQTPPSSTSDQSANTDLIAEFIRISELFRSAFKPLQVKGLDGVSVYGLDSGAIVAVPEKENRELIEPPPGFKDNRVSTVVVSPKFERPRELARIAILGHEQRKELRQVMKRTRMTYESLRIHLMAESMKNHVLGQGRRRRSDMAAAYIMRDRGLWLNYDKHIVGPVTGVEVGDIFFYRMELCVLGLHGQTQAGIDCLTAERSATGEPIATSIVVSGGYEDDEDTGDVLVYTGHGGQDHQHKQCDNQRLVGGNLGMERSMHYGIEVRVIRGIKYENSISSKVYVYDGLYKIVDWWFAVGKSGFGVFKFRLVRIEGQPMMGSAVMRFAQTLRNKPSMVRPTGYVSFDLSNKKENVPVFLYNDVDGDQEPRHYEYIAKAVFPPGIFGQGGISRTGCECKLSCTDDCLCARKNGGEFAYDDNGHLLKGKHVVFECGEFCTCGPSCKSRVTQKGLRNRLEVFRSKETGWGVRTLDLIEAGAFICEYAGVVVTRLQAEILSMNGDVMVYPGRFTDQWRNWGDLSQVYPDFVRPNYPSLPPLDFSMDVSRMRNVACYISHSKEPNVMVQFVLHDHNHLMFPRVMLFALENISPLAELSLDYGLADEVNGKLAICN

>AtSDG6/SUVR5

MEVKMDELVLDVDVEEATGSELLVKSEPEADLNAVKSSTDLVTVTGPIGKNGEGESSPSEPKWLQQDEPIALWVKWRGKWQAGIRCAKADWPLTTLRGKPTHDRKKYCVIFFPHTKNYSWADMQLVRSINEFPDPIAYKSHKIGLKLVKDLTAARRYIMRKLTVGMFNIVDQFPSEVVSEAARDIIIWKEFAMEATRSTSYHDLGIMLVKLHSMILQRYMDPIWLENSFPLWVQKCNNAVNAESIELLNEEFDNCIKWNEVKSLSESPMQPMLLSEWKTWKHDIAKWFSISRRGVGEIAQPDSKSVFNSDVQASRKRPKLEIRRAETTNATHMESDTSPQGLSAIDSEFFSSRGNTNSPETMKEENPVMNTPENGLDLWDGIVVEAGGSQFMKTKETNGLSHPQDQHINESVLKKPFGSGNKSQQCIAFIESKGRQCVRWANEGDVYCCVHLASRFTTKSMKNEGSPAVEAPMCGGVTVLGTKCKHRSLPGFLYCKKHRPHTGMVKPDDSSSFLVKRKVSEIMSTLETNQCQDLVPFGEPEGPSFEKQEPHGATSFTEMFEHCSQEDNLCIGSCSENSYISCSEFSTKHSLYCEQHLPNWLKRARNGKSRIISKEVFVDLLRGCLSREEKLALHQACDIFYKLFKSVLSLRNSVPMEVQIDWAKTEASRNADAGVGEFLMKLVSNERERLTRIWGFATGADEEDVSLSEYPNRLLAITNTCDDDDDKEKWSFSGFACAICLDSFVRRKLLEIHVEERHHVQFAEKCMLLQCIPCGSHFGDKEQLLVHVQAVHPSECKSLTVASECNLTNGEFSQKPEAGSSQIVVSQNNENTSGVHKFVCKFCGLKFNLLPDLGRHHQAEHMGPSLVGSRGPKKGIRFNTYRMKSGRLSRPNKFKKSLGAVSYRIRNRAGVNMKRRMQGSKSLGTEGNTEAGVSPPLDDSRNFDGVTDAHCSVVSDILLSKVQKAKHRPNNLDILSAARSACCRVSVETSLEAKFGDLPDRIYLKAAKLCGEQGVQVQWHQEGYICSNGCKPVKDPNLLHPLIPRQENDRFGIAVDAGQHSNIELEVDECHCIMEAHHFSKRPFGNTAVLCKDISFGKESVPICVVDDDLWNSEKPYEMPWECFTYVTNSILHPSMDLVKENLQLRCSCRSSVCSPVTCDHVYLFGNDFEDARDIYGKSMRCRFPYDGKQRIILEEGYPVYECNKFCGCSRTCQNRVLQNGIRAKLEVFRTESKGWGLRACEHILRGTFVCEYIGEVLDQQEANKRRNQYGNGDCSYILDIDANINDIGRLMEEELDYAIDATTHGNISRFINHSCSPNLVNHQVIVESMESPLAHIGLYASMDIAAGEEITRDYGRRPVPSEQENEHPCHCKATNCRGLLS

>AtSDG9/SUVH5

MVHSESSILSSLRGGDGGGIPCSKDELAINGSYTDPMGRRKSKRFKVAAESEFSPDFGSITRQLRSRRMQKEFTVETYETRNVSDVCVLSSQADVELIPGEIVAERDSFKSVDCNDMSVGLTEGAESLGVNMQEPMKDRNMPENTSEQNMVEVHPPSISLPEEDMMGSVCRKSITGTKELHGRTISVGRDLSPNMGSKFSKNGKTAKRSISVEEENLVLEKSDSGDHLGPSPEVLELEKSEVWIITDKGVVMPSPVKPSEKRNGDYGEGSMRKNSERVALDKKRLASKFRLSNGGLPSCSSSGDSARYKVKETMRLFHETCKKIMQEEEARPRKRDGGNFKVVCEASKILKSKGKNLYSGTQIIGTVPGVEVGDEFQYRMELNLLGIHRPSQSGIDYMKDDGGELVATSIVSSGGYNDVLDNSDVLIYTGQGGNVGKKKNNEPPKDQQLVTGNLALKNSINKKNPVRVIRGIKNTTLQSSVVAKNYVYDGLYLVEEYWEETGSHGKLVFKFKLRRIPGQPELPWKEVAKSKKSEFRDGLCNVDITEGKETLPICAVNNLDDEKPPPFIYTAKMIYPDWCRPIPPKSCGCTNGCSKSKNCACIVKNGGKIPYYDGAIVEIKPLVYECGPHCKCPPSCNMRVSQHGIKIKLEIFKTESRGWGVRSLESIPIGSFICEYAGELLEDKQAESLTGKDEYLFDLGDEDDPFTINAAQKGNIGRFINHSCSPNLYAQDVLYDHEEIRIPHIMFFALDNIPPLQELSYDYNYKIDQVYDSNGNIKKKFCYCGSAECSGRLY

>AtSDG11/SUVH10

MGLVGLHSGTIDMEFIGVEDHGDEEGKQIAVSVISSGKNADKTEDPDSLIFTGFGGTDMYHGQPCNQKLERLNIPLEAAFRKKSIVRVVRCMKDEKRTNGNIYIYDGTYMITNRWEEEGQNGFIVFKFKLVREPDQKPAFGIWKSIQNWRNGLSIRPGLILEDLSNGAENLKVCLVNEVDKENGPALFRYVTSLIHEVINNIPSMVDRCACGRRSCGSKHVFREKLSVSSSLVISAKKSGNVARFMNHSCSPNVFWQSIAREQNGLWCLYIGFFAMKHIPPLTELRYDYGKSRGGGKKMCLCRTKKCCGSFG

>AtSDG13/SUVR1

MAPNLRIKKACDAMKLLGISETKTRAFLRKLLKTYENNWDFIEEDAYKVLLDAIFDEADAQSTEKNKKEEEKKKKEEEKKSRSVATSRGRRKAPEPLVQDEEDDMDEDEFPLKRRLRSRRGRASSSSSSSSSYNNEDLKTQPEEEDEDDGVTELPPLKRYVRRNGERGLAMTVYNNASPSSSSRLSMEPEEVPPMVLLPAHPMETKVSEASALVILNDEPNIDHKPVISDTGNCSAPMLEMGKSNIHVQEWDWETKDILNDTTAMDVSPSSAIGESSEHKVAAASVELASSTSGEAKICLSFAPATGETTNLHLPSMEDLRRAMEEKCLKSYKIVHPEFSVLGFMKDMCSCYIDLAKNSTSQLLETETVCDMSKAGDESGAVGISMPLVVVPECEISGDGWKAISNMKDITAGEENVEIPWVNEINEKVPSRFRYMPHSFVFQDAPVIFSLSSFSDEQSCSTSCIEDCLASEMSCNCAIGVDNGFAYTLDGLLKEEFLEARISEARDQRKQVLRFCEECPLERAKKVEILEPCKGHLKRGAIKECWFKCGCTKRCGNRVVQRGMHNKLQVFFTPNGKGWGLRTLEKLPKGAFICEYIGEILTIPELYQRSFEDKPTLPVILDAHWGSEERLEGDKALCLDGMFYGNISRFLNHRCLDANLIEIPVQVETPDQHYYHLAFFTTRDIEAMEELAWDYGIDFNDNDSLMKPFDCLCGSRFCRNKKRSTKTMQILNKA

>AtSDG17/SUVH7

MDKSIPIKAIPVACVRPDLVDDVTKNTSTIPTMVSPVLTNMPSATSPLLMVPPLRTIWPSNKEWYDGDAGPSSTGPIKREASDNTNDTAHNTFAPPPEMVIPLITIRPSDDSSNYSCDAGAGPSTGPVKRGRGRPKGSKNSTPTEPKKPKVYDPNSLKVTSRGNFDSEITEAETETGNQEIVDSVMMRFDAVRRRLCQINHPEDILTTASGNCTKMGVKTNTRRRIGAVPGIHVGDIFYYWGEMCLVGLHKSNYGGIDFFTAAESAVEGHAAMCVVTAGQYDGETEGLDTLIYSGQGGTDVYGNARDQEMKGGNLALEASVSKGNDVRVVRGVIHPHENNQKIYIYDGMYLVSKFWTVTGKSGFKEFRFKLVRKPNQPPAYAIWKTVENLRNHDLIDSRQGFILEDLSFGAELLRVPLVNEVDEDDKTIPEDFDYIPSQCHSGMMTHEFHFDRQSLGCQNCRHQPCMHQNCTCVQRNGDLLPYHNNILVCRKPLIYECGGSCPCPDHCPTRLVQTGLKLHLEVFKTRNCGWGLRSWDPIRAGTFICEFAGLRKTKEEVEEDDDYLFDTSKIYQRFRWNYEPELLLEDSWEQVSEFINLPTQVLISAKEKGNVGRFMNHSCSPNVFWQPIEYENRGDVYLLIGLFAMKHIPPMTELTYDYGVSCVERSEEDEVLLYKGKKTCLCGSVKCRGSFT

>AtSDG18/SUVR2

MRSYNLFHESNPTISFSFVGAETMAPNLHIKKAFMAMRAMGIEDARVKPVLKNLLALYEKNWELIAEDNYRVLADAIFDSHEDQAIQESEEKKADEVKEDEGCAAEVDRGKKKLHESIEDDEDVMAESDRPLKRLRRRGEGGSALASPSLGSPTLEGPSINDEENAPILLPYHPVPIENDHDAGELILTKVEPITNMPLSSIPDSVDRGDSSMLEIDKSNGHVEEKAGETVSTADGTTNDISPTTVARFSDHKLAATIEEPPALELASSASGEVKINLSFAPATGGSNPHLPSMEELRRAMEEKCLRSYKILDPNFSVLGFMNDICSCYLDLATNGRDSANQLPKNLPFVTTNIDALKKSAARMAYTSQASNDVVEICSNEHMRDAENGAVGDSMALVVVPECQLSADEWRLISSVGDISLGKETVEIPWVNEVNDKVPPVFHYIAQSLVYQDAAVKFSLGNIRDDQCCSSCCGDCLAPSMACRCATAFNGFAYTVDGLLQEDFLEQCISEARDPRKQMLLYCKECPLEKAKKEVILEPCKGHLKRKAIKECWSKCGCMKNCGNRVVQQGIHNKLQVFFTPNGRGWGLRTLEKLPKGAFVCELAGEILTIPELFQRISDRPTSPVILDAYWGSEDISGDDKALSLEGTHYGNISRFINHRCLDANLIEIPVHAETTDSHYYHLAFFTTREIDAMEELTWDYGVPFNQDVFPTSPFHCQCGSDFCRVRKQISKGKNVKKRA

>AtSDG19/SUVH3

MQGVPGFNTVPNPNHYDKSIVLDIKPLRSLKPVFPNGNQGPPFVGCPPFGPSSSEYSSFFPFGAQQPTHDTPDLNQTQNTPIPSFVPPLRSYRTPTKTNGPSSSSGTKRGVGRPKGTTSVKKKEKKTVANEPNLDVQVVKKFSSDFDSGISAAEREDGNAYLVSSVLMRFDAVRRRLSQVEFTKSATSKAAGTLMSNGVRTNMKKRVGTVPGIEVGDIFFSRIEMCLVGLHMQTMAGIDYIISKAGSDEESLATSIVSSGRYEGEAQDPESLIYSGQGGNADKNRQASDQKLERGNLALENSLRKGNGVRVVRGEEDAASKTGKIYIYDGLYSISESWVEKGKSGCNTFKYKLVRQPGQPPAFGFWKSVQKWKEGLTTRPGLILPDLTSGAESKPVSLVNDVDEDKGPAYFTYTSSLKYSETFKLTQPVIGCSCSGSCSPGNHNCSCIRKNDGDLPYLNGVILVSRRPVIYECGPTCPCHASCKNRVIQTGLKSRLEVFKTRNRGWGLRSWDSLRAGSFICEYAGEVKDNGNLRGNQEEDAYVFDTSRVFNSFKWNYEPELVDEDPSTEVPEEFNLPSPLLISAKKFGNVARFMNHSCSPNVFWQPVIREGNGESVIHIAFFAMRHIPPMAELTYDYGISPTSEARDESLLHGQRTCLCGSEQCRGSFG

>AtSDG20/SUVR3

MQRLRESPPPKTRCLGEASDIIPAADRFLRCANLILPWLNPRELAVVAQTCKTLSLISKSLTIHRSLDAARSLENISIPFHNSIDSQRYAYFIYTPFQIPASSPPPPRQWWGAAANECGSESRPCFDSVSESGRFGVSLVDESGCECERCEEGYCKCLAFAGMEEIANECGSGCGCGSDCSNRVTQKGVSVSLKIVRDEKKGWCLYADQLIKQGQFICEYAGELLTTDEARRRQNIYDKLRSTQSFASALLVVREHLPSGQACLRINIDATRIGNVARFINHSCDGGNLSTVLLRSSGALLPRLCFFAAKDIIAEEELSFSYGDVSVAGENRDDKLNCSCGSSCCLGTLPCENT

>AtSDG21/SUVH8

MVSTPPTLLMLFDDGDAGPSTGLVHREKSDAVNEEAHATSVPPHAPPQTLWLLDNFNIEDSYDRDAGPSTGPVHRERSDAVNEEAHATSIPPHAPPQTLWLLDNFNIEDSYDRDAGPSTSPIDREASHEVNEDAHATSAPPHVMVSPLQNRRPFDQFNNQPYDASAGPSTGPGKRGRGRPKGSKNGSRKPKKPKAYDNNSTDASAGPSSGLGKRRCGRPKGLKNRSRKPKKPKADDPNSKMVISCPDFDSRITEAERESGNQEIVDSILMRFDAVRRRLCQLNYRKDKILTASTNCMNLGVRTNMTRRIGPIPGVQVGDIFYYWCEMCLVGLHRNTAGGIDSLLAKESGVDGPAATSVVTSGKYDNETEDLETLIYSGHGGKPCDQVLQRGNRALEASVRRRNEVRVIRGELYNNEKVYIYDGLYLVSDCWQVTGKSGFKEYRFKLLRKPGQPPGYAIWKLVENLRNHELIDPRQGFILGDLSFGEEGLRVPLVNEVDEEDKTIPDDFDYIRSQCYSGMTNDVNVDSQSLVQSYIHQNCTCILKNCGQLPYHDNILVCRKPLIYECGGSCPTRMVETGLKLHLEVFKTSNCGWGLRSWDPIRAGTFICEFTGVSKTKEEVEEDDDYLFDTSRIYHSFRWNYEPELLCEDACEQVSEDANLPTQVLISAKEKGNVGRFMNHNCWPNVFWQPIEYDDNNGHIYVRIGLFAMKHIPPMTELTYDYGISCVEKTGEDEVIYKGKKICLCGSVKCRGSFG

>AtSDG22/SUVH9

MGSSHIPLDPSLNPSPSLIPKLEPVTESTQNLAFQLPNTNPQALISSAVSDFNEATDFSSDYNTVAESARSAFAQRLQRHDDVAVLDSLTGAIVPVEENPEPEPNPYSTSDSSPSVATQRPRPQPRSSELVRITDVGPESERQFREHVRKTRMIYDSLRMFLMMEEAKRNGVGGRRARADGKAGKAGSMMRDCMLWMNRDKRIVGSIPGVQVGDIFFFRFELCVMGLHGHPQSGIDFLTGSLSSNGEPIATSVIVSGGYEDDDDQGDVIMYTGQGGQDRLGRQAEHQRLEGGNLAMERSMYYGIEVRVIRGLKYENEVSSRVYVYDGLFRIVDSWFDVGKSGFGVFKYRLERIEGQAEMGSSVLKFARTLKTNPLSVRPRGYINFDISNGKENVPVYLFNDIDSDQEPLYYEYLAQTSFPPGLFVQQSGNASGCDCVNGCGSGCLCEAKNSGEIAYDYNGTLIRQKPLIHECGSACQCPPSCRNRVTQKGLRNRLEVFRSLETGWGVRSLDVLHAGAFICEYAGVALTREQANILTMNGDTLVYPARFSSARWEDWGDLSQVLADFERPSYPDIPPVDFAMDVSKMRNVACYISHSTDPNVIVQFVLHDHNSLMFPRVMLFAAENIPPMTELSLDYGVVDDWNAKLAICN

>AtSDG23/SUVH6

MEMGVMENLMVHTEISKVKSQSNGEVEKRGVSVLENGGVCKLDRMSGLKFKRRKVFAVRDFPPGCGSRAMEVKIACENGNVVEDVKVVESLVKEEESLGQRDASENVSDIRMAEPVEVQPLRICLPGGDVVRDLSVTAGDECSNSEQIVAGSGVSSSSGTENIVRDIVVYADESSLGMDNLDQTQPLEIEMSDVAVAKPRLVAGRKKAKKGIACHSSLKVVSREFGEGSRKKKSKKNLYWRDRESLDSPEQLRILGVGTSSGSSSGDSSRNKVKETLRLFHGVCRKILQEDEAKPEDQRRKGKGLRIDFEASTILKRNGKFLNSGVHILGEVPGVEVGDEFQYRMELNILGIHKPSQAGIDYMKYGKAKVATSIVASGGYDDHLDNSDVLTYTGQGGNVMQVKKKGEELKEPEDQKLITGNLALATSIEKQTPVRVIRGKHKSTHDKSKGGNYVYDGLYLVEKYWQQVGSHGMNVFKFQLRRIPGQPELSWVEVKKSKSKYREGLCKLDISEGKEQSPISAVNEIDDEKPPLFTYTVKLIYPDWCRPVPPKSCCCTTRCTEAEARVCACVEKNGGEIPYNFDGAIVGAKPTIYECGPLCKCPSSCYLRVTQHGIKLPLEIFKTKSRGWGVRCLKSIPIGSFICEYVGELLEDSEAERRIGNDEYLFDIGNRYDNSLAQGMSELMLGTQAGRSMAEGDESSGFTIDAASKGNVGRFINHSCSPNLYAQNVLYDHEDSRIPHVMFFAQDNIPPLQELCYDYNYALDQVRDSKGNIKQKPCFCGAAVCRRRLY

>AtSDG31/SUVR4

MISLSGLTSSVESDLDMQQAMLTNKDEKVLKALERTRQLDIPDEKTMPVLMKLLEEAGGNWSYIKLDNYTALVDAIYSVEDENKQSEGSSNGNRGKNLKVIDSPATLKKTYETRSASSGSSIQVVQKQPQLSNGDRKRKYKSRIADITKGSESVKIPLVDDVGSEAVPKFTYIPHNIVYQSAYLHVSLARISDEDCCANCKGNCLSADFPCTCARETSGEYAYTKEGLLKEKFLDTCLKMKKEPDSFPKVYCKDCPLERDHDKGTYGKCDGHLIRKFIKECWRKCGCDMQCGNRVVQRGIRCQLQVYFTQEGKGWGLRTLQDLPKGTFICEYIGEILTNTELYDRNVRSSSERHTYPVTLDADWGSEKDLKDEEALCLDATICGNVARFINHRCEDANMIDIPIEIETPDRHYYHIAFFTLRDVKAMDELTWDYMIDFNDKSHPVKAFRCCCGSESCRDRKIKGSQGKSIERRKIVSAKKQQGSKEVSKKRK

>AtSDG32/SUVH1

MERNGGHYTDKTRVLDIKPLRTLRPVFPSGNQAPPFVCAPPFGPFPPGFSSFYPFSSSQANQHTPDLNQAQYPPQHQQPQNPPPVYQQQPPQHASEPSLVTPLRSFRSPDVSNGNAELEGSTVKRRIPKKRPISRPENMNFESGINVADRENGNRELVLSVLMRFDALRRRFAQLEDAKEAVSGIIKRPDLKSGSTCMGRGVRTNTKKRPGIVPGVEIGDVFFFRFEMCLVGLHSPSMAGIDYLVVKGETEEEPIATSIVSSGYYDNDEGNPDVLIYTGQGGNADKDKQSSDQKLERGNLALEKSLRRDSAVRVIRGLKEASHNAKIYIYDGLYEIKESWVEKGKSGHNTFKYKLVRAPGQPPAFASWTAIQKWKTGVPSRQGLILPDMTSGVESIPVSLVNEVDTDNGPAYFTYSTTVKYSESFKLMQPSFGCDCANLCKPGNLDCHCIRKNGGDFPYTGNGILVSRKPMIYECSPSCPCSTCKNKVTQMGVKVRLEVFKTANRGWGLRSWDAIRAGSFICIYVGEAKDKSKVQQTMANDDYTFDTTNVYNPFKWNYEPGLADEDACEEMSEESEIPLPLIISAKNVGNVARFMNHSCSPNVFWQPVSYENNSQLFVHVAFFAISHIPPMTELTYDYGVSRPSGTQNGNPLYGKRKCFCGSAYCRGSFG

>AtSDG33

MAGKRKRANAPDQTERRSSVRVQKVRQKALDEKARLVQERVKLLSDRKSEICVDDTELHEKEEENVDGSPKRRSPPKLTAMQKGKQKLSVSLNGKDVNLEPHLKVTKCLRLFNKQYLLCVQAKLSRPDLKGVTEMIKAKAILYPRKIIGDLPGIDVGHRFFSRAEMCAVGFHNHWLNGIDYMSMEYEKEYSNYKLPLAVSIVMSGQYEDDLDNADTVTYTGQGGHNLTGNKRQIKDQLLERGNLALKHCCEYNVPVRVTRGHNCKSSYTKRVYTYDGLYKVEKFWAQKGVSGFTVYKYRLKRLEGQPELTTDQVNFVAGRIPTSTSEIEGLVCEDISGGLEFKGIPATNRVDDSPVSPTSGFTYIKSLIIEPNVIIPKSSTGCNCRGSCTDSKKCACAKLNGGNFPYVDLNDGRLIESRDVVFECGPHCGCGPKCVNRTSQKRLRFNLEVFRSAKKGWAVRSWEYIPAGSPVCEYIGVVRRTADVDTISDNEYIFEIDCQQTMQGLGGRQRRLRDVAVPMNNGVSQSSEDENAPEFCIDAGSTGNFARFINHSCEPNLFVQCVLSSHQDIRLARVVLFAADNISPMQELTYDYGYALDSVHGPDGKVKQLACYCGALNCRKRLY

>OsSDG703

MGIPEVVVPPRAAGPRRYKGLVPWRFQPGFVRPPPVKPPAAAAAVAGGGVAGTPGGKGRG

LGASGEGVGSSGGRGDPQSRRCTRSASAKGSGDARSVEEGGPRVAGDDGGSGKSGVAAEG

SGFEGLRNGRGGGVGTAAAEDCGLEKSNPDGIVGDADVHLESGSDARDGECVSEGLKKPC

VNNSNGSSAADCAPKVKKGNDSGNGGADECNAAAKSSNLACPGNNGDETNRKGRKVVLPW

RFQVGFKRSFSKAFCSDSESSGPSGTQFYRAQDSSTPCTPATRSSVRCYASAHSGVRVSA

MRDFSVKGEKETSTPYKKSKTGMDGPSQGMPKNGVVLARENIMGSLQNFRLIYRDLLDEE

EEKSTEAVIRPDLQAYRIFRERFITDCDEKKYIGNVPGIKVGDIFHLRVELCVVGLHRPH

RVGVDHIKQEDGTCIAVSIVSYAQSSDIKNNLDVLVYSGAMTAIANQKIEGTNLALKKSM

DTNTPVRVIHGFVTHLNGNCQRKKIPTYIYGGLYIVEKYWREKEGNDRYVYMFRLRRMAG

QKHIDIQDILNSGQAESYGGIIIKDISRGLEKIPVSVVNSISDEYPMPYRYIAHLQYPRN

YQPAPPAGCGCVGGCSDSKRCACAVKNGGEIPFNDKGRILEAKPLVYECGPSCKCPPTCH

NRVGQHGLRFRLQVFKTKLMGWGVRTLDFIPSGSFVCEYIGEVLEDEEAQKRSTDEYLFA

IGHNYYDEALWEGLSRSIPSLQKGPDKDEEAGFAVDASKMGNFAKFINHSCTPNLYAQNV

LYDHDDKSVPHIMFFACEDIPPRQELSYHYNYTIDQVHDANGNIKKKKCLCGSIECDGWL

Y

>OsSDG704

MAGTRQTTSVPMDNAAVVDAKPLRTLTPMFPAALGLHTFTAKENSSSIVCITPFGPYAGG

TEQAMPASIPPMFASPAAPADPNQRQPYAVHLNGAAPANGTANNTGVIPDLQIAVAGTVE

SAKRKRGRPKRVQDSSVPSAHLVPSAPGGNITAVQTPPSATTDESGKKKRGRPKRVQDVP

VLSTPSAPQVDSTVFQTPASAVNESVTRKRGRPRRVQDGADTSAPPIQSKYNEPVLQTPS

AVTLPEDGKRKRGRPKRVPDGALIPLSHSGVSIDDDSGEIITGKRGRPRKIDVNLLNLPS

LFSDDPRESVDNVLMMFDALRRRLMQLDEVKQGAKQQHNLKAGSIMMSAELRANKNKRIG

EVPGVEVGDMFYFRIEMCLVGLNSQSMSGIDYMSAKFGNEEDPVAISIVSAGVYENTEDD

PDVLVYTGQGMSGKDDQKLERGNLALERSLHRGNQIRVVRSVRDLTCPTGKIYIYDGLYK

IREAWVEKGKTGFNVFKHKLLREPGQPDGIAVWKKTEKWRENPSSRDHVILRDISYGAES

KPVCLVNEVDDEKGPSHFNYTTKLNYRNSLSSMRKMQGCNCASVCLPGDNNCSCTHRNAG

DLPYSASGILVSRMPMLYECNDSCTCSHNCRNRVVQKGSQIHFEVFKTGDRGWGLRSWDP

IRAGTFICEYAGEVIDRNSIIGEDDYIFETPSSEQNLRWNYAPELLGEPSLSDSSETPKQ

LPIIISAKRTGNIARFMNHSCSPNVFWQPVLYDHGDEGYPHIAFFAIKHIPPMTELTYDY

GQSQGNVQLGINSGCRKSKNCLCWSRKCRGSFG

>OsSDG705 MSELDENGDDDYEEGHNFVASRKYSMSLSTVTSVHGEPYSYYHRKGLMKRQYDDDDDDDDDDDDDDDDEDEEAEEEEEEEEEEEEELSYWRNDFVYGDIVWARLGKRQPVWPGVVVDPAQPAAAQALPPQPRSGAVLCVMLFGWAAEFGDEKKFIWVRQGGIFPFVDYMDRFQGQTELSSCKPGDFQRALEEAFLADQGFFEVPMDGNTTGQPAVCQSFPADLEEVTGSNELECQSQIKAS

>OsSDG709

MNRESNFMPTPDQDVLEVKPLRTLAPMFPAPLGIDVLNRLTAPPLVFVAPAGQFPGGFGS

LNIPAVRSFAAFGGQDASGGKTAGGGDQDASGGKTAAGGDQDAGRGETAAFGGQETVRGE

FVANGTPNVGASATGPIDATPISACKSTQPSVISLDDDDNDDDEPYGGNQTSASGRKIKR

PSHLKGYNVSDGLGTDSSNGTKKRPKTSNRKAATDNEISLMPPSSDPREVVEVLLMTFEA

LRRRHLQLDETQETSKRADLKAGAIMLASNLRANIGKRIGAVPGVEVGDIFYFRMELCII

GLHAPSMGGIDYMNKFGDEDDSVAICIVAAGVYENDDDDTDTLVYSGSGGISRNSEEKQD

QKLERGNLALERSLSRKNVIRVVRGYKDPACLTGKVYIYDGLYKIHESWKERTKTGINCF

KYKLQREPGQPDAVAIWKMCQRWVENPAARGKVLHPDLSSGAENLPVCLINDVNSEKGPG

HFNYITQVKYLKPLRSMKPFQGCRCTSVCLPGDTSCDCAQHNGGDLPYSSSGLLVCRKLM

VYECGESCRCSINCRNRVAQKGVRIHLEVFRTTNRGWGLRSWDPIRAGSFICEYVGEVVD

DTKVNLDGEDDYLFRTVCPGEKTLKWNYGPELIGEHSINISADTFEPLPIKISAMKMGNV

ARFMNHSCNPNTFWQPVQFDHGEDGYPHIMFFALKHIPPMTELTYDYGDIGCESRGVGSR

AKNCLCGSSNCRGFFS

>OsSDG710

MDGPTLELSEMMLHAAQPWRSRCTQRDVRPGAVPPRPVAADGRGEGTSTVRGRVLEGTTR

GGGRGGGMEREREVVAPARNAVAVAGDLATHGGERVAGPLVAKEKRNGGGELGTKRGLEK

RAPLPPPKRRVVSAKRQFPPDFGRDSAVPLGRGRGRGGGVRPSDGAPARAVLGEKVASAG

NGDSMANVHHHAVMDTVLMKSSHASDENLVAFKVGSPENGAEGAARGKGAHNGELLGKRE

VLAQAVNLLPMRRTVSATHRFTAGCGRDAAAPLARREEGKVGSGLEVMPVDVGGGVSKEV

MATDGSKHSVNQCTANIVGAVGVLDGTVQYQELEEGEVADEAYCDVESQKVVGCDSFDDS

AGERHEGVVPVTFAVTEVLTSHAYDEMMQIKALQEGGSDAAQETEHDLPMGGKCETILPD

ASPKCSFGGPSNEIVHGKRVLGSHGMKGEVPSLAIEDHGGIAQIDQELEDVEMTTGEYRV

QDAQIATHVIPHESTTGRHEGGLCASAAAEDVKVMNKYKGTLPKGAAKSSMNIATGVFGD

GIMRSKILSTARKVVKPPVRASHKPPLNTLHRPFSTNSASFGHKKLKVKRPDQSKDIPMK

IASTSGLAGKDNLIDEKALSLEDDDILKALVVHDGKLEVYLNVPSCVQLHRQHGSGNADD

RSKIRMLCRRFQFICRALLHAVEQGSLKIRRVDLAADKIIRKLPGFTKPGPTVGNVNGVE

VGDEFMYRVELALVGLHRPYQGGIDTTDYNGVLVAISIVCSGGYPDELSSSGELIYTGSG

GKPAGKKKDEDQKLERGNLALKNCIETKTPVRVIHGFKGQNREDNSHSRAKQILTFTYDG

LYLVVDCWTEGLKGSRIFKYKLQRIPGQPELPLHIAKGLRRSLSRPGLCIADISQGKEMD

PICVINDVSNVHPTSFQYISRIKYPSWLTKRHPQHHGCDCSDGCIDSTKCFCAVKNGGKI

PFNSNGAIVHDKPLIFECGPSCRCHSSCHNRVSQKGMKIHLEVFRTANKGWGVRSLRSIS

SGSFICEYVGILLTDKEADKRTNDEYLFDISHNCDDEDCSKGRPSTISSLNSSGGCSQTM

EDVCFTIDASEYGNIGRFINHSCSPNLYAQNVLWDHDDQRVPHIMFFAAENIPPLQELTY

DYNYKIGEVRDLNGRVKVKDCHCGSPQCCGRLY

>OsSDG712

MAKPNGKEKTGDTGLSMAPPKISKDRFDAAIRAMADIGILKETAAPVLNNLLNLFDYNWV

HIEADNYLALADAIFCDSDPKEGQKRQANETNLDADQSNKKLKTKKRSQNPTSKMHGNDN

REFVEAPPQQGRGTLSARTVNGKKVTRAHLELPSSQLLIKEPHTCPSIAKNTTIVENNSA

VLCHGQDLQTFEVPVATTCPQVVAPSTRKDARRTSGARHDQKHEGVSGAHERNRAVACSN

QEIVSSKDSPSNIEVVLSNYGAGKLSFTYNSSLANRSDFHLPDIKLICKKMEARCLRKYK

SLEPNFSFKNLIKDTCQCIVESSGPRHEGIIQTVPALDILSKPSVPQILQSNQANSAFMP

PNNVMSLGGTSSSCTVAGVSQNSSNMPVVPHQLHIGANRPPHDVNDITKGEERLRIPIIN

EYGNGILPPPFHYIPHNITLQEAYVNISLARIGDDNCCSDCFRDCLAQSLPCACAAETGG

EFAYTTDGLLKGAFLDSCISMIREPLKHPHFYCKICPNERMKIEVNSDSSNTEMNPGPCK

GHLTRKFIKECWRKCGCTRNCGNRVVQRGITRHLQVFLTPEKKGWGLRSTEKLPRGAFVC

EYVGEILTNIELYDRTIQKTGKAKHTYPLLLDADWGTEGVLKDEEALCLDATFYGNVARF

INHRCFDANIIGIPVEIETPDHHYYHLAFFTTRIIEPFEELTWDYGIDFDDVDHPVKAFK

CHCGSEFCRDKTRRSKSRARV

>OsSDG713

MESNQHKASDPQDSMVHLDLDEDKIMVTSALPCPSMSVGKSVMRKRGRPSRHARGTSLSS

VTPEGCKKMEGRSYNLRSDSTILLRNSCLLIADGSTKQKRSWGLDKDDLHIPFFQISDNP

REAVDDILMTFGGLHRRIMQLIDVKMASKQLVFQALNLMRKVGYHVNKDKRVGEVPGVKI

GDIFYSRIEMLLVGLHSNINRGIEFMSGAFINKEDKIATCIVSSGMYENGDDDPYTLVYN

GQGKVHHKLERGNYSLNQSFIRRNHIRLIRSEPNPLVRLGSKEKIYIYDGLYKIEEKYRQ

TTKSRSNLKFNKLVRELGQPNGIVVWKNTQKWRENPSCRDHVIMPDMSNGAEIARVCVVN

NIDSEDAPNNFTYSTKLDNGNHMVSANKMCVCKCTSSCLGEDNCSCLKTNGSYLPYNSSG

ILVCRKTMIYECNDSCACTINCSNRVVQRGSYLHFEVFKTMDRGWGLRSWDPIPAGAFVC

EYVGVVIDKDSLVEEDEYIFEVTRPEHNLKWNYLPELIGEPSFYDMNDTFKKLC

>OsSDG714

MEVMDSVAVMEVSPVPKPPLEAALALRRSVRCLNRTRRPTYVEQEEPKESAGRRRGGKRK

REEEKKEPVAQHAMKPVRMGDAASERKPSSEGKPMPAIAAEPVSCAGFARPAAEDDVLGN

GKSAKLRVKETLRAFTSHYLHLVQEEQKRAQAVLQEGQKRPSKRPDLKAITKMQESNAVL

YPEKIIGELPGVDVGDQFYSRAEMVVLGIHSHWLNGIDYMGMKYQGKEEYANLTFPLATC

IVMSGIYEDDLDKADEIIYTGQGGNDLLGNHRQIGSQQLQRGNLALKNSKDNGNPIRVIR

GHISKNSYTGKVYTYDGLYKVVDDWVQNGVQGHVVFKYKLKRLEGQPSLTTSEVRFTRAE

APTTISELPGLVCDDISGGQENLPIPATNLVDDPPVPPTGFVYSKSLKIPKGIKIPSYCN

GCDCEGDCANNKNCSCAQRNGSDLPYVSHKNIGRLVEPKAIVFECGANCSCNNNCVNRTS

QKGLQYRLEVFKTASKGWGVRTWDTILPGAPICEYTGVLRRTEEVDGLLQNNYIFDIDCL

QTMKGLDGREKRAGSDMHLPSLHAENDSDPPAPEYCIDAGSIGNFARFINHSCEPNLFVQ

CVLSSHNDVKLAKVTLFAADTILPLQELSYDYGYVLDSVVGPDGNIVKLPCFCGAPYCRK

RLY

>OsSDG715

MASPPPPPRLLTPKPDPDAPLPPLPYPDPNLVQSMLFSAQSPQAQPQAPPPHIQPPASAS

AEAPSGDEKNKKKKKRARASQEMVRITNLSIADHLHYRSLVRRARLTFEALRAIYQRQDL

ATAGGIRNRFDLRASSKMLSKGLWMHRDIRTVGSIPGLLVGDSFFYRAELCVLGLHTAPQ

AGIGYIPASIVDQGHPVATSIVSSGGYLDDEDSGDVLVYSGSGGRLRNRLDHSADQTLQR

GNLALHYSCHYGIEVRVIRGHACDHSPSSKVYVYDGLYRVVTSTFGPGKSGRDVCKFKLV

RIPGQDDLGSKAWHTAAELKDALDSKIRPPKYISLDIAKGKEPFRVPLYNKLDDDRSPLF

YDYIACPDFPTTQQLLKRQTQRGCHCAELCGSRCSCERKNRGADGPVYTSDGILLRGRPL

VYECGPLCGCPMTCPNRVTQQGMKHRLEVFRSKETGWGVRTLDLIQPGAFICEYAGDVLS

LDSHSGDAPLPPMEDGSSIIDPTKFPERWREWGDASVVYPDRVPHFPLFAGARYRLDVSQ

RRNVACYISHSCSPNVFLQYVIRGNEDESYPHMMVFAMETIPPMRDLSIDYGLD

>OsSDG726

MEMDTSPSSSAPSSPAASSDSIDLNFLPFLKREPKSEPASPERGPLPLPAAAPPPPPPPP

PPPPPPQVQAATVATPVPATPDLSAAAVMTPLQSLPPNPEEETLLAEYYRLATLYLSSAG

AAGVIVPAAAPEASAGAVAQPGSGSGAKKRRPRSSELVRVSSLSVQDQIYFRDLVRRARI

TFESLRGILLRDDERAEVLGLTGVPGFGAVDRRRVRADLRAAALMGDRDLWLNRDRRIVG

PIPGISVGDAFFFRMELCVLGLHGQVQAGIDFVTAGQSSSGEPIATSIIVSGGYEDDDDR

GDVLVYTGHGGRDPNLHKHCVDQKLEGGNLALERSMAYGIEIRVIRAVKSKRSPVGKVYF

YDGLYKVVDYWLDRGKSGFGVYKYKMLRIEGQESMGSVNFRLAEQLKVNALTFRPTGYLG

FDISMGREIMPVALYNDVDDDRDPLLFEYLARPIFPSSAVQGKFAEGGGGCECTENCSIG

CYCAQRNGGEFAYDKLGALLRGKPLVYECGPYCRCPPSCPNRVSQKGLRNRLEVFRSRET

GWGVRSLDLIKAGTFICEFSGIVLTHQQSEIMAANGDCLVRPSRFPPRWLDWGDVSDVYP

EYVAPNNPAVPDLKFSIDVSRARNVACYFSHSCSPNVFVQFVLFDHYNAAYPHLMIFAME

NIPPLRELSIDYGMIDEWVGKLTM

>OsSDG727

MSVLNPPPKRRAISAIRKFPRDCGRAASTLAESGASMEELPLEATPISVATGGASMEDSL

ARTPISVQGASLVCGLDHSSEAIDGKTIEDDESSKVENRIQEFQVATNVALDDFEGAKNG

STHPNDSIAKPSPSHGFVERVNGKGSQQEKKLVARSAGDGKMVSKYEERLQKGTPETRMR

DLVDVKAKKKILKSDKMNGALQNDARSSGDGKMKTKASSTQRGVVRSDMSLKQGDIARKV

DATGKCKGGVNSLIKEATSGKHATTNGIEENDDRDLVSDRIIVQALMAPDKCPWTRRRKS

IGGSSESRTPKLKKKFGRPRKELKDTTPREEVSPEVASCKAIKHEAIEDKEDSYFEDEGN

SKASYSDVEGNSKELVRGGKALVVCGGKKELCVTLPPSAPFGTDPRSKIRNLLIKFHAAC

RKLVQVEEQHKGNIGRIDIEAGKALKQNGFIKPGPIVGNVAGVEVGDEFNFRIELSFVGL

HRPYQGGIDSTKVNGILVAISIVASGGYHDELSSSDELIYTGSGGKAIGNKAAGDQKLER

GNLALKNSIETKTPVRVIHGFKGHSKGEASHSKSKQISTYIYDGLYMVVDYWKEGPEGSM

VYKYKLQRIPGQPELALHIIKATRKSKVREGVCVPDISQGRERIPIPAINTIDDTQPTAF

KYTTEVIYPHSYAKEPLKGCDCTNGCSDSNRCACAVKNGGEIPFNSNGAIVEAKPLVYEC

GPSCRCPPTCHNRVSQHGIKIPLEIFKTGNKGWGVRSLSSISSGSFVCEYAGEVLQENGD

EHVETDEYLFDIGHHYHDEVWEDPKFEGILGLESSTSKTTEDTEGSKTTEDTEGSTIDAS

KCSNVGRFINHSCSPNLYAQNVLWDHDDMKKPHIMFFATENIPPLQELTYDYNYGKVEDK

NGKEKVKPCFCGSPDCSRRLY

>OsSDG729

MRNSATPGAVGELAELVLPWLPPQDLAAAASASRALRAAASSVSAGRAADAAHGLEPHPI

PFDNLVDGKPYAYFLYTPFSLTPSSASASPRRAQPWGRTWARPPGPTWPRSDLGGFPSSG

CACAQGACGGARGCPCADPEAEAVGLGSEAGMGSLRECGDGCACGPSCGNRRTQLGVTVR

LRVVRHREKGWGLHAAEVLRRGQFVCEYAGELLTTEEARRRQGLYDELASVGKLSPALIV

IREHLPSGKACLRVNIDATKVGNVARFINHSCDGGNLHPVLVRSSGSLLPRLCFFAARDI

IEGEELTFSYGDARLRPNGLPCFCGSLCCSGLLPSEET

>OsSDG733

MENSEDEAESDKLPLDLEPLRSLAPKFPTILGYDVETQSTDPLLVYATPSIPCSSSEQPQ

EAPASFSLPLPKSPVPIKATPISAAFPTPQHEDESSDQDYKPFCKNKKPAMPKRAKRPQQ

AEKSNDANIKRRSIRRNLDNEFNLCSSSSDNPKESVEGILMMFDSLRRRVLQLDEKEDAS

RRADLKAGTLMMQNNLRINNHKMIGHVPGVEVGDIFFFRIEMCIVGLHAPAMGGIDYISS

KNKDETLAVCIISSGGYENDDDDTDILVYTGQGGNSRHKEKHDQKLERGNLALMNSKSKK

NQIRVVRSAQDPFCNSGKIYIYDGLYRIEDTWTDTAKNGFNVFKYKLRRDPGQPDGISLW

KMTEKWKANPATREKAILLDLSSKVEHLPVCLVNDVDDEKGPSHFNYVAGVKYLRPLRKT

KPLQCCKCPSVCLPGDPNCSCAQQNGGDLPYSATGLLAKHTPMVYECSSNCQCSHNCRNR

ITQKGIKLNFEVFWTGDRGWGLRSWDPIRAGTFICEYAGEVIDETKMDIDVEEDKYTFRA

SCPGNKALSWNLGEELLEEKSTAVITKNFKKLPIIIRANNEGNVARFLNHSCSPNLLWQA

VQYDHGDDSYPHIMFFAMEHIPPMTELTYDYGTRGAPPGFEGKPFKACKLKSCLCGSKHC

RGLF

>FvJmjC2
MENNSVPPGFAPRTSFKLKKAEVSVVGVEGTDRSNAVVDSSKQGSTQMDSTSEMTALEKVKTFLQQRPWLLFDQTDHTSVQPDPQQFQMEPPPKACLSNGVTRGSPESTYCVKEPRLPKGVTHGCPESGYCVKVTERWRPEGGIIEKLDKAPVFYPTHEEFKDTLTYITSISARAAQ
FGICRISPPPSWIPPPPIEENSIWRSSTFAåSHIQRIDGLRSKLDGSDESRKKKRRISTVGLDCGFEFTSSMGEAGQSDVNCFESERGPEFTLDSFKRYAD
DFKRQYFRKSDVIGNQEQWEPSVENIEAEYTRIVENPTEGIEVLCGNYLDTKGLGSGFPIVSPDTPECLQSGWNLNNLPRLPGSLLSFESHDTCHILVPQ
TRVGMCFSSSLWRVEEHHLYSLCYNHLGAPKVWYGVPGSSRIRFEAAKTKSFPDLLKQHRLVKQVSPSALKSEGTPVFCCIQRPGEFVLVFPGAYHSEFD
CGFNFSESACFAPLDWLPYGQNAVELYSERERKTSISYDKLLLGAAREAVRAQWEISLLKNKKTSEGLRWKSACFKDGILTELLKTRLKSEEVRRKYFCT
SLKSQKMNSDFDATIKRECSICFCDLHFSAVGCPCSVDRYSCLFHAKQRCFCDWSNKFVLYRHEIKELNLLVEALEGKLSAVIKWAKDDLGLYLHEHLPI
SLTDKTKLKEYTSEDASSPVSTCDTASSIKAELKARMLQSTISNMLKANRDPIASTDAASSIRAELKARLLQSSISKTLTGNDNPLDAAAANGNGRNFVS
SQSTISDEHKGMCTPQQPVAMVGTPILPIEVASDVSSVTSSESSSESDDIIPHLGKSSESSHQAESRAPQDCVHVLSDDSDG

> FvJmjC5
MARLFSLPSILSLVLAIALDGSGCSNGGGGMGMLARGGSIPCVIRMFWWVYLKAAAWAQRRCCVLASFVAGPTSDNL
WRSPLSVKTACDFDQDRIVLPEWGGRLGLGWGYMQLRLLGLLGYQEVLVLIDLDEVIERVFLILVLQAMEETGFAAESQPKEVHSSNNKQRSDNTVECSD
SLLSQKAGNILHVIHLFFTVIIAARWVPDEACRPVIDDAPVFYPTAEEFADTLGYIAKIWTQAESYGICRIVPPHSWIPPCPLKEKDMWENAKFSTRIQQ
VDLLQNRESMKKKTRGRKRKRRRHSKRRAEANAASDTDEKFGFQSGSDFTFAEFQRHAFTFKESYFGTQDCKEGSNSGGNNKKRWEPSAEDIEGEYWRIV
EQPTDEVEVYYGADLETGVFGSGFPKASSVVTKSDPDLYAMSGWNLNNLPRLPGSVLCFEESDISGVLVPWLYVGMCFSSFCWHVEDHHLYSLNYLHFGD
PKVWYGVSGSRATSLEQAMRKHLPDLFEEQPDLLNELVTQLSPSVLKSEGVPVHRVVQHAGEFVLTFPRAYHAGFNCGFNCAEAVNVAPVDWLQHGQTAV
ELYSKQCRKTSLSHDKLLLRSALDAVQVLGQTSLGTKFISNRSWQKVCGKDGMLTKAVKRRVEMEEERLDRLPICWKSQKMDRDFDSNTERECFSCFYDL
HLSAASCNCSPDRFSCLKHAKHFCSCEMTQRYVLLRYTVEELNLLVKALEGELDAIHVWASKDSGVVSIDYTHKCAAKKPKLDGASKSCDPMEIMPDCPI
SEDKVNMNGSCSSSSHVSSAVVQSGSPDDHNGHESLVVNAAPKVEHDCSFDLNLNCASDEHESKVIDVSDGCDNKTSTIEEETSTSMSNQEKASMSEGNK
LFGVDLGLSRPASNIPPISSSKTEIVDTAAVNASMRQKSYQSRSLSLVEPLNFGSLMAGNYWCTKQVIYPKGFRSRIKYYSVLDPTKLCSYISEVLDAGL
LGPLFKVSLEEYPEESFANVSADKCWEMVLNRLNNEISRRSSLAERGLPPLQYSQSINGFAMFGFLSQPIVEAIEALDPDHQCTEYWNHRRKQQHSSVPS
SLGLPQTKLFGINMTNKEQNEGEHSINETQLVLRRLIEKANPTPEEFRTLHRIFSSQSVESRVACADLIEEMQRNVDNMKP

>FvJmjC15
MGTELMRVCLKEDNDEFPSVPPGFESFTSFSLKRVNDSEKQDSENLITCNATTSTSGSQSVPMETGNDVADVAKRSQ
RRRPGINNRRYNNYAEDESDYESLDQSFLQRSLLPKGVIRGCPECSTCQKVSARWHPGDGQRPDIQDAPVFRPTEEEFKDTLKYIASIRPKAEPYGICRI
VPPSSWRPPCPLKEKNVWEASKFATRIQRVDKLQNRNSMRKIPKSQNHARKKRRRCTRMGADCPGGGRGFGDDGNCEAEIFGFEPGPMFTLGAFEKYADD
FKTQYFSKNEHVTDIGSHLSEVKERWEPSVENIEGEYWRMVEKPTEEIEVLYGADLETGVFGSGFPKMSRQDGSTSEEQYITSGWNLNNFPRLPGSVLSY
ESSDISGVLHVEDHHLYSLNYMHWGAPKLWYGIPGSEACRFEEVMRKHLPDLFEEQPDLLHKLVTQLSPSILKSNGVPVYRCCQNPGEFVLTFPRAYHSG
FNCGFNCAEAVNVAPVDWLPHGQVAIELYQEQGRKTSISHDKLLLGAAREAVRAHWELNLLKKNTFDNLRWKNVCGKDGVLAKVLKARVEMERVRREFLC
NSSQALKMESNFDATSERECSICFFDLHLSAAGCHQCSPDRYACLNHAKQFCSCAWSSKFFLFRYDIDELNILLEALEGKLSAVYRWARLDLGLALSSYI
GKDNMKIGKLSHASKSTMLEGVSSHPQSNCFKDQLGKEISKDDPGRSTGREESFLSTANSLQVCQLSREDTSYALNSAEKESGMKMTSVETIILLSDDES
DEPKKDDGSDEPTKLHSDNLTAISSANELEPSNSLVAPDGKVSPCNVEKVAVLNLPVTDADVMVKRVISPSASGDEKSHIINVKDEQESEGQSRSNSPNL
PSALDSVGAEHGPDTCHIGGPKVAISRSDPKDSQPCGNIKPENEDRHEKIVRNADANIVDNVRTATGNPSPSQNNLDRYYRQKGPRIAKVVRRITCIVEP
LEFGVVISGKSWCNSQAIFPKGFRSRVKYISVLDPTVRCYYVSEVLDARQAGPLFMVSLEECPGEVFVHNSVGRCWDMVRDRVNQEITRHHKLGRSNLPP
LQPPGSLDGFEMFGFTSPVIVQAIEAMDRNRVCSEYWDSRPYSRPQVQIPQKAPSEETRENLNDQEAAGVSLLSSGVDAILGGLFKKANLEELNSLYSIL
SDNQQTVGRGLVTRLLNEEIQTRRPT

> FvJmjC10
MAAPPEQPPEVLPWLRALPVAPEYHPTWAEFQDPIAYIFKIEKEASQYGICKIVPPVPPAPKKTAIANLNKSLILRN
GPVTGKGPKAQPTFTTRQQQIGFCPRKARPVQRPVWQSGEHYTFSQFEAKAKSFEKSYLKKQRKKGGLSALDIETLYWKATVDKPFSVEYANDMPGSAFV
PLSSKKSGGSTSREAGDGVTLGETAWNMRGVSRSRGSLLRFMKEEIPGVTCPMVYVAMMFSWFAWHVEDHDLHSLNYLHMGAGKTWYGVPREAAVAFEEV
VRVQGYGGEINPLVTFATLGEKTTVMSPEVFISSGIPCCRLVQNAGEFVVTFPRAYHTGFSHGFNCGEAANIATPEWLRVANDAAVRRASINYPPMVSHF
QLLYDLALALCSRTPVHSSAEPRSSRLKDKKKGEGETVVKGLFVKNVIQNNELLHVLGKGSSIVLLPQSSSDISVCSKLRVGSQLRVNPDDLIIDGNRGI
KQVSVKGKLASLCESSRHLSLNGNDSAATPSKMLNMSAKRESNVEGEGLSDQRLFSCVTCGILSFSCVAIIQPREAAARYLMSADCSFFNDWAVDCEPIQ
GANGDPNSSKKGPCTETGLKQKSAPDSLYDAPFQSADNQNQITDPSNEVDSNTENQRDTNALGLLALTYGVSSDSEEDQANQDVPVCGDKSNLSDCSLEG
RYEYQSASPPLRASYGGTAGVRSPTSPGFDCGIGLPTIDGNGLPTIDVYVENRPEATNFKDKGHQYSVDLDTNNLALTKTNGLVGTSIDPMKVSYSGSPD
AFDVQPTGFGQVTLRKDSTGTSFAPGFDHDSSRMHVFCLEHAVEVEQQLRSFGGAHILLLCHPDYPRIVDEAKEIAEELGVNYPWNDLVFRNATRADEQR
IQSALDSEEAIAGNGDWAVKMGINLFYSASLSRSHLYSKQMPYNSVIYNAFGRSSPATSPAGPEVCGRRPAKQKKVVVGKWCGKVWMSNQVHPFLIKREH
EEKKVEQERRRFQESPIPDEKLHGNTESTHKTEKTVVTKQYSRKRKMTVDGETTKKAKRTDAVSAQSVDDDSHLQQMRFLKNKQGKHIESGPTKKSKIEK
EDAVSSDSMEDDFRQQNRRTLRSKQAKHSVGDDDVSDDSMGVDSQQQQTRIAKSKQAKHSAKDFSVVSDDSVGVDSDHQQKRVAESNTREFSAVSDDSLD
ESIHQLHRRSLRRNKGKSIGRENFTSQNLYGVSSRQKQKKTSKSKQAKIVEREEAALDETTDDNAALQHKIVRGKQIKPETLQQMKRETPHRVRQGSRRL
QESQQQTPRIRNTTDVHAEEPEGGPSTRLRKRPPKEQPETSRKKAKVQPETGRKKAKEQQQTGRIKVNTASAVKTKNASARKTKNASGARVEEAEFVCDI
EGCTMSFGTKHELNLHKKNVCPVKGCGKKFFSHKYLVQHRRVHEDDRPLRCPWKGCKMTFKWAWARTEHIRVHTGARPYVCAEPGCGQTFRFVSDFSRHK
RKTGHSVKKGKGRSR

>FvJmjC18
MGDVKIPNWIEGLPLAPEFYPTHTEFADPIAYISKIEKEASEFGICKIIPPLPRPSKRYVFNNLNKSLARRPELGCD
LVPGSDGAVTKMGAADGSNDGEVRAVFTTRHQELGQSVQRGKEPTVQDLTSSGVAKQVWQSGEVYTLEQFESKARTFARSILGTIRDVSPLVIEAMFWKA
ASEKPIYIEYANDVPGSAFEEPEGVLFYSRRRRRKRNSYHRSGPNSDSKKSEVIRSCEKNSQNGEVEEATPKNVSPTCLEVPKSSASPGIVSTDETSQSS
RKRSQNSSCDMEGTAGWMLSNSPWNLQVIARSPGSLTRFMPDDIPGVTSPMVYIGMLFSWFAWHVEDHELHSMNFLHNGSAKTWYSVPGNYAFAFEELIR
TEAYGGTADRLVVNGSTNMFRMVQFLFLAAALSLLGNKTTLMSPEVVIASGIPCCRLIQNPGEFVVTFPRAYHVGFSHGFNCGEAANFGTPQWLKVAKEA
AVRRAAMNYLPMLSHQQLLYLSTMSFISRVPRALLPGVRSSRMRDRQKEDRELSVKKAFIEDILNENDVLSALLGKESSCRAVLWNPDLLPYTSKESPIP
TAGAPVDTNSKENATDTQGGKSTNDQNSLVDEMSLYMENLNDLYLGSDDLSSDFQVDSGTLACVACGILGFPFMSVMQPSEKASTELQPEYILSEELPGN
SHFSPELHEAFKDSATEILSPISNPCTTRFDNHWNIVNKFLRPRSFCLEHAVETLELLQCKGGANMLVICHSDYQKIKAPAGAIAEEIGCPFNYKEVPLD
TASKEDLNLIDLAVDDGRDECGEDWTSKLGINLRYCVKVRKSSSSKRVQHALALGGALSTQSCSAEFLTVDWKSRRSRTKKVYLDATYKQCQTIEKKKEE
VVEAKSAAAASFKSEATIIQYSRRNKRRPSTSTGAGRVVEQPATSEEFDKHGRRASDSSTHNNGKSTSLCARLDSYASKSMSEVHPDVQMLEATRDICLN
SLPQVADRVALTAGSADKQIENHSLEERQMNSRGSSLAASESDMQHEIKILEEARVNDPITPLAIACDERSENREKQENRKLNKNDGNCNLVSERQSQLW
AEEDVLMDVGPVFTELTNVCTADSIVTSSEEQMGNAVIDKSCVNSEICESITTATDRSNNKAPASYNSTLVSDPTSAASEGHVEFERETCIAEDFSNVVS
SEFKPEEDIEIPQGRNEDSSFSHARRIRQPSPACTDRLSGVPSTICAEADFHRGPTSHIQEFQAADRSSEGEYISTSVIQMEETHPSISLEESPEVPMGS
SLEEGTSNGVILATVVQQDAQTTNKAVEAPSQNFVIRVKNHPIPVYVEDDVPRVSCATEATLDDKEQWTNSYSNKELIASHDTSKCELSRVTIKTYFRVK
RGSRAAEKLCNGSEDCISQPERETGNIEPSLVDHRPGPETGSKRKRGLEQKMDDNFNINGYIRGPCEGLRRRAGKGVTVSEIDIHDEVEEKPVAKKVKRP
SDASVHLKDKKKEQVKKTHSCNLGSCSMSFHTKEELMVHKRNRCPHEGCGKKFSCHKYAMVHSRVHENARPFKCPWKGCSMSFKWAWAQTEHIRVHTGEK
PYKCKVDGCGLSFRFVSDFSRHRRKTGHYVS

> FvJmjC8
MGITIGGEIERVNGKEVSYSEFVERYMEKNQPVVLTGLMEDWRACRDWVQDDGKPNLQFFATHFGKSKVQVADCGTR
EFTDQKRLEMTVGIFVEQWPGDSEEEEEGGSAPSQAAGSKMLLYLKDWHFVKEYPEYTAYTTPMFFCDDWLNMYLDNHRMHNDPDIYSENNDISCSDYRF
VYMGAKGLSIFAHGLLFMLMFSGHIAGQQMNMKSSVYNILDDVSETKFPGFAKEDTISINHNWFNANNLRWVWDLLLRDYNESKEYIEDIKDICDDFEGL
CQRNLAANTGMNFNDFLIFLARFSFANLIRLQYVSRKNGNQTRNLSPITQHFTFNLVSIQKIASRISSLQDPGSHGFCLDFRETLKDPGFIRLCAVLGKT
YGMIHVRENWNFDMEKAWIDDMKDLDVIGTCNSQVCTPKDLVTFIEYALAESNGG

> FvJmjC6
MALPAASTALSAADSDLHLPTPTLDSESSALLHTVSEHGGYAYVSMASRAAAGDVRAAEAAREMAWEQLHSGPWHSV
LPVWRDAYAMACLHVAKLHFAAGEFSDALRALDMGLLMGGPLFKSDLHSAVSKVSAKSRARVSEENGPNLDRRLVRDDLKASEMVGVLPPKSLSCKMVVK
KSGLSLEGFLREHFLPGSPVILTDCMGHWPARTKWNDMDYLLRVAGDRTVPVEVGKNYLYPEWKQELITFSQFLERIRGTDSSSGVPTYLAQHSLFDQIN
ELREDICIPDYCCAGGGELRSTNAWFGPAGTITPLHHDPHHNVFAQVVGKKYIRLYRASLSEELYPHTETMLCNSSQVDLDNIDEKEFPKVQDLEFLDCI
LEEGDMLYIPPKWWHYVRSLTTSMSRKSQSGGYFTTSAWQLAWAHLLLKSDLDGAIQPDPDPFLCLIRNTNREAINLGLVKPSHFSVPKSQTPSKLRVFT
LRFSSATANAVSVYCEVDLLVIRVTRKPIGCHAKVDNHNYAVDPKSLPDGVEVIHVNLKDGTCAGLAYPARNIMSVQYHPEASPRPHDSDYAFGEFIELM
NRVKEFLERIRGNDSGSGSPMYLAQPSLFDKVCYNCDTHVCSGIHCAGELRSFNARFGPVGTVAPLHHDLHHNVFAQVDLDNMDDKEFPEVHLEYLDCIF
EEGDMLYIPPKHYVWSMTTSMSVSFWWSDNDSSAVLIACGLTEQFSVAGLYILMYISCCVIFTIRLAEV

> FvJmjC3
MEGKIETLWNEVRELNLGTSGDLDRLDSPPTPLQFLRDFVSQNKPCIISNATNHWPALTRWTSDSYLTSALSSSLVS
LHLTPHGRADSLVPSPSSSLCFASAHVQRTPFPEALRLIKNDASFVAYAQQQNNCFQSEYSPLAADCDPHIPFATEALGCRPEAVNMWIGEERSTTSFHK
DHYENLYAVVTGQKHFLLLPPTDVHRMYIREYPAAQYRHSPVTGEFELEMESPARPVPWSSVDPYPAPEDREEQLKKFPLFFNGPRPFRCTINPGEMLYL
PSMWFHHVRQSPDSRGRTIAVNYWYDMQFDIKYAYFNFLQSIQCASIESQMLAETVQEDFDSDSPEKTEDE

> FvJmjC16
MDLRLGPGNGEDNLGIPDDMRCKRSDGKQWRCTARSMPDKTVCEKHYIQAKKRAANSALRANMKKAKRKPSGEGDLF
LESKSDDFDVPLATKSDDVDVPLASIKSEEKSRPSHGKKFTKISKNQFRYSPDPPPMRSVPRRNLSNEERKSDEHEDDWSSYKSPPVSALDSPRNRPQRS
FDANAMPVSESADGSSESSEDAGGQTCHQCRRKDDTVIWCHRCDRRGYSNTPPEDIQSSCPACSGTCNCKVCLRRDNLVKVRIREIPALDKLQYLHCLLS
SVLPVVKQIHQEQCFEVELEKKLRGSDIDLARTKLNADEQMCCNFCRIPIIDYHWHCPGCAYDVCLNCCLDLREASKQVVKGEVTEEIDDESQEKETMLE
QFAKVRLNFSEKFPDWKANSNGSIPCPPKEYGGCGYSALSLSRIFKMNWVAKLVKNVEEMVSGCRVNDAASLALTEVNDKRLCQYAHREDSDNFLYCPRS
EDIKCDGIATFKRHWLRGEPIIVKRVFDSSTVSSWDPAVIWRGIQETTDEKSKDQNRMVKAIDCYDWSEVDIELGHFIEGYSEGQIYENGRPKILKLRDW
PSPSASEEFLLYQRPEFIRKLPLLEYIHSKFGLLNVAAKLPHYSLQNDVGPKIFISYGTHEELDKGNSVTNLHFNMRDMVYLLVHACVVKQKGQQKTKIE
NVQKPFEASEVKESHEDLVMGAGDSTFPDLSIDQSEENPYEARLDTDKVDSAVNHGLETTHVEMNTISCEHSEKEGDDISQKTHPGVLWDVFRRKDVPKL
TEYIRIHGEEFGKLKSETNDLVTRPLYDETCFLNEHHKRKLKEEFGVEPWSFEQNLGQAVFIPAGCPFQVRNLQSTVQLGLDFLSPESLGDAARLAEEIR
CLPNDHEAKQQVSEVGKISLYAASSAIKEIQRLVLDPKFSGELGFEDPNLTAAVSENLEKITKRRQIACS

> FvJmjC14
MCQVFVGGTYWAQGKMGTGDMLPCRDTPGVSVQHSTNLNMAPLFQKLPRGFAPTVFQLSLVSTIEEERGRGSKTQKP
KRKKLLVENGDGDVNRVAVRTLRKRPDRNKKVGDMEAEENGGKSVKKRGGGGRKKVVIVKEEEKKGELEVKGDKKRGRKRAKTEEEEEEGGKNKVRCSFR
RLREQGHLSVEERKKWIEEVSMMCHQCQRSDKGSVVRCKKCKTKRYCVPCIENWYPHISEDAIAEACPFCCGNCNCKACLRLDIPVKNLKNQELVLDKDA
RVEHSKYLIHALLPSLKRIHDEPKSEMAEEARSKGISDSELQIPKSDCAADVRGWFDLKHWPQILKLKDWPPTNLFEERLPRHGAEFISCLPYKEYTHPR
NGPLNLFAKSPSKCVKPDMGPKTYIAYGFSQELGRGDSVTKLHCDMSDAVNILTHTTEVNLTPEQLTVIEKLKKKNALQDQREIFGNCNIVDDNIDSDKA
GGDSFFSAADDKQPFDEVESNSIGVAVSELVNPIVPHVGDASMESESGSEKNEEAGLESDISGDIPVTNYGNKLKESDVTEGGALWDIFRRQDVPKLQVY
LMKHFKEFRHIHCCPVQQVVHPIHDQTFYLTVQHKKMLKEEYGIEPWTFIQKLGDAVFIPAGCPHQVRNLKSCIKVAMDFVSPENVGECFHLTEVFRTLP
EDHRAKEDKLEVKKMILHSVTDALKELDQILRLVNNFT

> FvJmjC13
MARRGKRKRKQDPDTHLPNQTEHVAVKAGDEVLSEIKGEDSLGNTQKEEKAEGVGVGVKQKKKPGRKSKKGLVGNGD
GGLKGVVGFGGGEIAVRTLRKRPERNKKVGDMEAAGNGEKGKENGAKSVKKRGGEGRKKVEIAKEEGKKGELEVKGDTKRGRKRAKTEEGGNKVKVRCSF
RRLREQGHLSAEERKKWIEEVSMMCHQCQRSDKGSVVRCKKCKTKRYCVPCIENWYPHTSEDAIAGACPFCCGNCNCKACLRLDIPVKNLKNQELVLDKN
VRVEHSKYLIHALLPSLKIILDEQKSEMAEEARSKGISDSELQIPKSDCTADVRVYCNNCRTSIFDYHRSCPKCSYDLCLLCCLEIREGKLQLGTEEVTL
EYVSQGLPYLHGGEEAKNKNGKEKRNRNAVEPPTETGPKTSGRRIFEWKSDEDGIILCPPEDMGGCGVGKLELKCIFSKNYVEELVKKAEVIDETYKLVY
TSGTSAERCPCLNSVDDVNININTSRKAASRDNSEDNYLYCARAGDIKVEDVKHFQWHWIKGEPVIISNALETGSGLSWEPLVMWRACRQMQHTKHGKHL
NVKAIDCLDWCEAEVNIHWFFTGYLKGWFDQEHWPQILKLKDWPPTNLFEERLPRHGAEFISCLPFKEYTHPRNGPLNLFAKLPSECVKPDMGPKTYIAY
GFSQELGRGDSVTKLHCDMSDAVNILTHTTEVNLTPEQLTIIEKLKKKHAIQDRREIFGNCQIVGDNIDSDKAGGDSFFSAADDKQTFNEVESNSIGVAV
SKLVDAIVPQVGDASMGFESESEMNEEAGLHRDIGSEMPARNYGNKLKESDVTEGGALWDIFRRQDVPKLQEYLMKHFKEFRHIHCCPVQQVVHPIYDQT
FYLTVEHKKKLKEEYGIEPWTFIQKLGDAVFIPAGCPHQVRNLKKVLLGTHHFSFFLLVIPLSLLVPLNSFKHNADHYLWPAIAIAI

> FvJmjC12
MVGRKRGRGGGRGGKRPRKEENGGEGVLGIENEEVKQVAVEEKPKPGRGRKRRGQKEEEEDCGGGQRRYTLRERALK
AETRKKRQRKVAAAAKEERPDVSTMCHQCQRNDKGEVVRCTGCWKRRERKRYCLHCIQTWYPNASVKSFAEACPVCRGNCNCKSCLRLDVPVRCLDNVDP
KIDDEETKLEHCKYLVHSLLPYLKRIKDEQVSEMVVEAKRQGLSHVSQLQVQKSNCLEDERMFCNHCNTSIFDFHRTCPDCEYDLCLVCCREIRDGKWKK
REGEAGHKYVDLGREYLHGEGEFNSLLKKNRENVRTGRREQKKRNVDVEVKPDKLESAEEEDEKFVNLGEHLDGEGELKRNDDVGVEQDDSESAEVATFE
WRASEDGSILCPPKHMKGCGKHTLELRCNFSQNHVIELVEKAGKIDESYNLLHASSETPAQKCSCVKPVDDAVSISSSSCKIRKAAYRDGSDDNYLFCPR
VGQIQDEDFGHFQWHWMRGEPVIVSNALENGSGLSWEPSVMWRAFRQIKNLNHDRELAVDAIDCLDWNFIEINNCDFFTGYSKGRFDKLDWPEILKLKDF
PPSTEFDNRLPRHGKEFLCCLPFKEYTHPKGSLNLAMFLPKESVKPDMGPKTYIAYGVVQELGRGDSVTKLHCDMSDAVNILTHIAEVTLAPEKLATIDE
LKKKHREQDRREMFGSCGTIAENGSVPQEDSAEVYEDGGALWDIFRCQDVPQLEKYIQKHINEFRHLHCNPLKSVTHAIHDQTVYLTAEHKRKLKEEFGI
QPWTFVQKLGDAVFIPAGCPHQVRNLKSCIKVALDFVSPENVGECFRLTEEFRTLPTNHRASEDKLEVKKMIVHTLCNAVMEITGEKKSEDDVKPKKRR

> FvJmjC21
MAPICRDELFSRVYYRKRRRQENGGEGILGIGDQDSTVAVKQLRQIKEEKDLIIGDQLKGQNGKDKSQENGGFDGIE
EGLAKKKKKRCGEKGMKTERFEENGDEDGLVKSRRWSKADVSTMCHQCQRNDNGRVVRCGGCSRRGDRKRYCIPCIKKWYPNSSEEDFAEACPVCLGNCN
CKACLRLDVPLRCSKNRDLEIGEDERVEQCKYLVNRLLPYLNRINDEQVREMNFEAEKQGLVEFEGMEIKKSDCRVGERMYCNNCKTSIFDFHRSCPGCQ
YDLCLNCCREIRGEEMVQKLGSECFDGEGKSGVGTRRLRSLFTKTKFEWKADKDGRIRCPPEHMEGCGKHHLELRCLFPGNKVMELVDKAENINENYKNS
YASSETCEESCSCKNPVDDVKCRKAACRGDSKDNYLFCPRADCIQPEDFVHFQRHWMRCEPVIVSNALENGSGLSWEPLVMWRAFRQVNKTKHEKDLEVQ
AIDCSDWSFIDVNMHKFFTGYSEGIFNRKDCPQILKLKDWPPSTEFDKRLPRHGKEFVCCLPFKEYTHPSSSTLNLTCHLPKSAVKPDLGPKTYIAYGVA
QELGHGDSVTKLHCDMSDAVNILTHATEVTLKPKQLAAIQELKRKHREQDQKEIFGNFGPKRLTCNEDIDDAGEGGALWDIFRVQDVPKLEQYIKKHCRE
FRHIQCRPLKQVVHPIHDQTIYLTVEHKRNLKAECGIEPWTFIQKLGDAVFIPAGCPHQVRNLKSCIKVAVDFVSPETVGQCFRLTEEFRTLPPDHRASE
DKLEVKKMIVHAVSDAVKVISKVKCKGSSVLPLRGFVCNPRKPSANTCLGMMSSNTDLPRSMALKAIPGSTPSAETSGAEVKEEKSEIYSNTMTEAMGAV
LTYRHELGMNYNFIRPDVIVGSCLQTPKDVDKLRSIGVKTIFCLQQNSDLEYPDSSRYFGVDIGAIQEYAKEFDDIEHLRAEIRDFDSYDLRLRLPAVVS
KLYKAINRNGGVSYIHCTAGLGRAPATVLAYMFWLQGYKLDDAVNLLLSKRSCFPKLDAIKNATADILTGMKKKAVTLTWRGHNCSTVEISGLDIGWGQE
EEKPLSNEKSKPRFPPPLSSLAFINVEFTYPVKFQHLIDHTASATKEIDTCLAFRSFLLSRIPLEFDDKLGSWILKRELPEGRYEYKYIVDGEWTINKNE
PVTSANKDGHVNNYVEASFSFDWCPILALFVIVEDDANSPNAIIRKRLTGDDPDLTTDERLKIRQFLEACPTDE

> FvJmjC22
MEKGKGLREAGNVGNEGGGAEMGEQRRKRIGGKSDEILGSGDLGIGGDEWFGAGNGGGGIGGGDEIARLFGEVGGGG
GVSFWGGGNGGGAEVGGGQGFGFEEIQGGFGEKFQFGGGLGGLDGGGGIHLWGNEGLGGEGMQGLFDRSGQFWGGEASGNVDSQGVQGCLSVNASGNGGE
IAEARDGSGEDRNGGGAQGSKGKRGRPKGSKNKTKPIGAEEVIVGLSGTGSFDVGGEESVTPKRKLGRPKGSKNKMKALANDENGGEESKVLVGNESVGR
KRKVNQPKGAKTKRKQDGGMPMEASMAIGVGNGSVQVKKKRGRPKGSTRKQDQAVKEDGRAKLAGYQAVGMSSETSFGNDVQTKDDDRKLQEIQEKFGGM
VVDNDAPMMSSKKERPINEGKEEKGMSAACGSNEGQNEVVQQKRPGQGANERPIASVGEDQSGKGENVRKKILDMRVQREDLSFPATQLKNSEDTEKKLR
KRPRGRPRKINSQPMNHSEFRSEQLKNSEDTEKKFLNRPRGKRPRGRPRMSKNLEMNHSEFHSEQLKNSEHTEQNRPRGKRPRGRPRKFNNLVMNHSTFR
AQRLKNCEHTEQKLCKRPRGRPRKFNNLQVHPRDIKPGISTDTLERKERLWCHQCLRNDRNGVVICLKCRKKRYCYDCLAKWYPGKTKQDIESSCPYCHG
NCNCRICLKEDIVVMVGDEETDTNIKLEKLRYLLCKTLPLLKHIQQEQRSELDAESTIRGVQLIEENLTRSILEDDDRVSCPNPDCSYDLCLTCCWELRK
GCQLGGSEAESSVKQFCEGNGKSTISDCQSPENVNENKPQTLLVNEFTNDIASEFPHWKAEADGRIPCPPKARGGCGTQLLELRRTFEADWVEKLILSSE
DLTIHYQPSDVDFSQGCSACHSISSACNGVNASEVRHAADRMNCHDNFIYCPDSVHLGNNDIEHFQLHWRRGEPVVVRNVLEKATGLSWEPMVMWRAFMG
AKKVLKEEAAKVKAIDCLDWCEVEINIFQFFKGYIEGRRYPNEWPEMLKLKDWPPSNLFEECLPRHGAEYMAMLPFSDYTHPKSGVLNLATRLPTALKPD
LGPKTYIAYGTMEELGRGDSVTKLHCDISDAVNVLTHATEVKIPPEQGKIIDRLQKEYEAEDEIIEGTSCNDKRFEHSNVTEDMNFVLGTDPSQQKTSGN
LISRKESMEFGSDSNRSARSDYKSDLVYGGAVWDIFRRQDVPKLIEYLLKHQTEFRHISNLPVIHPIHDQTLYLDEKHKRKLKEEFGVEPWTFEQHLGEA
VFIPAGCPHQSCIKVALDFVSPENVQECIRLTEEFRLLPENHRSREDKLEVKKMALYAASDAISEAEILKSRIRYCLLLFTITIRGYTMSRSRSGELSSA
GDSGNKADAMNDDRVELEKSNILVMGPTGSGKTLLAKTLARFVNVPFVIADATTLTQESFPVYFVAHNAGLHGLKETLNITPFFLMVVSISAAAGYVGED
VESILYKLLSVADYNVAAAQQGIVYIDEVDKITKKAESLNISRDVSGEGVQQALLKMLEGTVVNVPEKGARKHPRGENIQIDTKDILFICGGAFIDLEKT
ISERRQDSSIGFGAPVRANMRTGGVTDAVVTSSLLEIVESSDLIRYGLIPEFVGRFPILVSLSALTEDQLVQVLTEPKNALGKQYKKMFQMNDVRLHFTK
SALRLIAKKAISKNTGARGLRAILETILMDAMYEIPDVRTGDDIIDAVVIDEEAVGTDGQGCGAKILYGKGALDRYLSQNKEKDVQIATTEGSDVEPEGE
AELSSVVRSLGKGMDLESIECVSSSDGLDEDEIHQHLHNNINNPSHQHHHDFSSSKPRSNAAGNNNAAGIPGATAIAPATSVHELLECPVCTNSMYPPIH
QGWGCYDLDDVSNALLVFKEVEFSCKSMTLQGGLFLGAHIFWNVLILQELGDIRCLALEKVAESLELPCKFYSLGCPEIFPYYSKLKHESVCNFRPYNCP
YAGSECSVVGDIPFLVAHLRDDHKVDMHTGCTFNHRYVKSNPREVENATWMLTVFHCFGQYFCLHFEAFQLGMAPVYMAFLRFMGDENEARNYSYSLEVG
ANGRKLIWEGTPRSVRDSHRKVRDSHDGLIIQRNMALFFSGGDRKELKLRVTGRIWKEQQNPDAGVCIPNLCS

> FvJmjC19
MDSPPHDRWSKRGAGPKSRSSDAAQMKSGADKKIKRDSEEVAEEDRRCTRRAGAWRCGEEVVVGKPMCEHHLAQRKS
YQKKRRGGGADSDSGEEDGASRKVNAGESVNNKRRRRTGSESESDSESEKANNRTVKPKVNGKSGDSGNVMKKSKLKEEKPMEKSKSNRSKGSLMCHQCQ
RNDKNGVVHCSLCKAKRFCYECIERWYPGKSREDFENACPFCCGNCNCKACLREFLVKPCREVDPSVKLQRLRYLLYKALPVLRHIYSEQSSELEIEAKI
RGVHLTEMDIKRTKVDRNERIDNCYTSIVDFHRSCPNPNCSYDLCLTCCKELRNGRQPGGSEAETSHQQALDRAHKEVKGHCWESKGASTSDDSKVDPSI
SFPNWRADSHGSIPCPPKERGGCGNVKLELRRKFKANWVMKLLKNAEDFTTDFKWQEADISKGCSWCQPNDSEGTNDSQPERRQAAFRKNSHDNFLYCPN
AIDISDDEIEHFQRHWMKGEPVIVRNVLDKTSGLSWEPMVMWRAFRETGANVKFKEETKSVKAIDCWDWNEVEINIHQFFTGYLAGRMHKTKWPEMLKLK
DWPSSTLFEERLPRHCAEFIAALPYCDYTDPKDSNAGILNLATRLPEKSLKPDMGPKTYIAYGFSEELGRGDSVTKLHCDMSDAVNVLTHTTTVKIHSWQ
QNAIKALKSKHVAEDLCELYNERTHEKGKGGEGNNLDRAQSCRTSPLSDSVNPGILRSDETEYVPEPVATTELNKAKVASVDHQRNDVLKSTSPHANGVE
TKQECVQCSSDTISGRLEGKDASRNASEVNSRATKDFKSSDKLDVVHGGAVWDIFRIEDTSKLIEYLKKHKKEFRHLNNHPVESVVHPIHDQTLYLNERH
KKQLKQEYDVEPWTFEQHLGEAVFIPAGCPHQVRNRQSCIKVALDFVSPENLEVCLRLTEEFRLLPKTHRAKEDKLEVKKMTLYAVSSALREAKSLMPEL
GIVIVEYGIEEMFASGSGLKSRILKEYDGFKLLHLVACSNRTSVATRKNCGPYGQGFELLCKNLSCERGSHTTLLTTTPMRRLSCAWSLFQVHVGGVATM
LRDLT

> FvJmjC4
MRLAPKRIVKRVKNRTAFSRKMSQSSPSQVQNQKGGEFRPHQSPIMSPIIVTVLIIVVVVVVVVVVVVDIMSVLCEW
LRLKQQLHVFQPCNDRVYFAKWYLKGERGSPTSSGTFGSKFVNLDYRSYVRFLNWVPEALKMPEPELIDHAGLDSAVYLRIYLMGLKIFVPIAFVAWVIL
IPINWTDNTLDIAGKQANITSTNIDKLSISNIPYRSQRLWTHVVMAYAFTFWTCCVLLREYEKVATMRLHFLATEKRRPDKFTALVRNVPPDPEESVSEV
VEHFFLANHPDHYLTHQVVYDANKLAKLVKKKKKMDNWLVYYENKLERSKTSVRPFIKTGFLGLWGKKVDAIEHYQSEIVKMKKEIPEERERVKNDPKSI
MPAAFVSFKSRWGAAVCAQTQQSRNPTTWLTEWAPEPRDVYWPNLAIPYVKLTVKRLIMGVAFVFLTLFSLIPIAFVQSLASVEGLKKEAPFLNPIVKLK
FIKSVMTGFLPGIALKLFLIFLPMILMMMAKFEGYPSRSSLERRVAIPETIGFAIPMKATFFMTYIMVDGWAGIAAEILMLLPLILYHFKNTFLVKTDKD
REEAMAPGSIGFHVGEPRIQLYILLGFVYSTITPVLLLFIIVFFGLAYAVFRHQIVNVYSQEYESGAAFWPDVHGRIVCALIISQLILLGLLSTKRAALA
TPFLLVLPVSTIVFYKYCADRFEPAFVRYPLQEAKMKDDLEHARDPDLNVRDYLQSAYVHPVFKVCDEDDEDKEHNSIVVTMELLGRPEQYTDEDIGSGE
KGFSELLEHEKEELLQDQSDEDGKDNGGLQRIGAKSLSKKRRCTSENGSSNGKRIRKKQLVLNYYEDEDELMDEMERDTEIAFMIKSKTRKRGSPGRGRI
NRSSSVDSGVKDESKNTISYTSSSSPPSSPSSSVSVLRWERGRCTSRHRKNNGDVCFKCHQCMQESKTIVSCSKCKTNTYCIRCIKEWYPHMKVQDVKEF
CPFCRQNCNCNACLHSTGMIKTSKRDLSDYEKTQHLTHLILSVLPFLKEFSDEQKEEIKIEANIQQVKFQYRNRGCEYIHGGDPSRECSSVQAPDNHIEP
LTGWNANDDNSITCAPKEMGGCGERKLALKRILPCDWIANLEAKAKTVIDICETKNCTFKHEYGETRTDMLRKAASREDSCDNYLYCPDSWDTLKEGGLL
HFRKHWINGEPVIVQNVLEQANGLSWEPMVMWRALSENMDPNSTSQFSEVKTIDCLAGCEVEINTRQFFEGYTEGRMYGNSWPEMLKLKDWPPSDAFENL
LPRHCDEFISALPFQEYTDPRAGLLNLAVKLPPGVLKPDMGPKTYIAYGLVEELGRGDSVTKLHCDMSDAVNILTHTAEIKLGDEQKSAISDLKKLHRAQ
DERELRNWVNSQSGRSGSQIGDDEIIEKNYNREFKVPEDHAEGPTLSGSPCMEAVDETGGGALWDIFRREDVPKLEAYLKKHVKEFRHTYCSLVERVIHP
IHDQSFYLTVDHKRKLKEEFGIEPWTFVQSLGEAVFIPAGCPHQVRNLKSCTKVAVDFVSPENVHECLRLTKDFRQLPKNHRAREDKLEIKKMILYAVDH
AVKDLEALVATQV

> FvJmjC7
MGKGRPRAVEKGVVGPNLSCASSSSLTIPSAPVYYPSEDEFRDPLEYICKIRAEAEPYGICRIVPPESWKPPFALDL
EKFTFPTKTQAIHQLQVRPASCDSKTFELEYNRFLEDHCGKRLRRKVVFEGEELDLCKLFNAAKRYGGYDKVVKEKKWGEVVRFVRSARKVSECSKHVLH
QLYLEHLFEYEEYYNKLNKEGARGCKRGLQEEKNGECSSSKRRRTSNNDGERAKVRKVKKEEEEHDQICEQCRSGLHGEVMLLCDRCDKGWHIYCLSPPL
KQIPSGNWYCLDCLNSDEDCFGFVPGKRFSLEAFRRVADRAKKKWFGSGPASRVQIEKKFWEIVEGSIGEVEVMYGSDLDTSIYGSGFPRVNDLKQESVD
AKIWDEYCGSPWNLNNLPKLKGSVLRAVHNNITGVMVPWLYMGMLFSSFCWHFEDHCFYSMNYHHWGEPKCWYSVPGSEAGAFEKVMRNSLPDLFDAQPD
LLFQLVTMLNPSVLQENGVPVYSVLQEPGNFVITFPRSYHGGFNLGLNCAEAVNFAPADWLPHGGFGAGLYQLYHKTAVLSHEELVCVLAKSDCDSRVSP
YLKKELIRIYNKEKTWRERLWRKGIVKSSLMSSRKFPEYVGTEEDPTCIICQQYLYLSGVVCRCRPSTFVCLEHSERLCECKSSRLRLHYRHTLAELHDM
VKGGHASFAQLADQWLLRACKIFKSLFSREDYVNVLKEAEQFVWAGSEMNNVRETANNLKEARKWAEGVRKSVSKIESWSSNHDKDIEKVRVEYINELLS
FDSLPCDEPGHLILKGYAEKARMLIEEINTAMSSCSKVPELELLYNRVCEFPVYVTESEGLQQKILSAKVWIEGITKCISEKQPAAIELDVLYKLKLEIP
EVEVQLPQIEVLSDLVRKAESCQAQCVEILKGPITLKDVEALLLEWDTFSVNVPELKLLRQYHTDVVSWNARLKAVLTKIHEREDQDTVVDELEHILKDG
ASLKIQVNQMPAVEFELKKARCRERALRMRETIVSLDFIQEVMVDAQGLHIDGEQIFVNMSKVLDAAIQWEERAKYILAHGAQISDFEDVLRSSENIHVT
LPSLLDVKEALSKAMAWLSRSEPFLLHCSSLESASSSLLKVDTLKALISESKDLKVSMKEIKILETVLRNCEEWKHDACSLLQDTRCLLDMATNGEGISE
GLISKIEHVLARIGSMENTGLSLTFDFVELAKLKDACSLLQWCKKAISFCFAVPTLEDIESLISDAETSCCTDSSGALFDSLFEGVKWLKQATKIISAPS
NSTSCKLSEAEEVLADCQSINISFPLTVTQIEDFGPIVPWLIMVTLVSAYAIHACYKSKLLQNALMIPLELHNEAIASWLEQVHQFFSLRVAERSWSLIL
QLKELGIAGAFNCAELDSIISEVERVQKWKRQCMDIFRIAEENSLLCALEKLQQTLDRSMQIYDKANGLSEKGSYACCSVGSLDQEFVTCSSCKECYHLR
CLGSLTVYGKHSEYVCLCCQYLVSGTLQNEGNPRGFGGVRLALQKIVELLSEEDFCVCMEERDILKEVLKKARVCKTHLEALVDFALAYLDKDLSVIFAK
LATALKAVELEGLYDDEGYCNLTLALSRYSWKVRVERLLEGSKKPTIYQIQQHLKERVAVNIPPEDYFKQKLTELKCSGLQWADKAKKVAADSGALPLDK
VFELISEGENLPVLVEKELKLLKDRSMLYCICRKPYDQRAMIACDKCDEWYHFGCMKLRSTPKVYICPACEPLAETLPTSSVVPCTDAKFVEPKTPSPKH
TKPRMSPNKEEFIATQKVASTDDANVFRCSSGIDRLWWRNRKPFRRVAKKRAELDCLSLFSHVQQ

> FvJmjC9
MGSCESAKSESWGRLILLHVALAFRLYTHVSGARKSSPEVASARAFEVRKGLTNWKVGGADPPPSLSRNRSASPARR
LSSVGEPLSLTRSLKALDLRVSHRTVPFSTFCENVGFRGLAQGLCVICRGWDRDLGFGFSDLDSVEGRVCLSKEAKSGSESLKRRRLQPMRSESIPEPVS
FTNTMARSGGDALKAPAPCGVRIHGGANSVFRSNGASQGNDVFSKRKVDKFETNDLGWTEKIPECPVYYPAKEEFEDPLVYLQKIAPEASKYGICKIISP
VSASTPAGIVLMREKAGFKFTTRVQPLRLAEWDNDDKVTFFMSGRNYTFRDFEKMANKVFARRYCSSGSLPPTYLEKEFWKEIGSGKTESVEYACDVDGS
AFSSSPNDPLGSSKWNLKNLSRLPKSILRLLENAIPGVTDPMLYIGMIFSMFAWHVEDHYLYSINYHHCGASKTWYGIPGHAALQFEKVVKEHVYTHDIV
QSDGADGAFDVLLGKTTLFPPSILLEHDVPVYKAVQKPGEFVVTFPRAYHAGFSHGFNCGEAVNFAIGDWFPLGAIASRRYTLLDRMPLLPHEELLCKEA
MLLHASLELEDSERSSADLVSHNRVKVSFVKLMRFQHRARWILMKSGACNGVLPNTYGTVLCSICKRDCYLAYISCNCYMHHVCLRHDVRSLDLSCQRNP
TLFLREEIAEMEAVASKFEEEEEVLEEIQGQGENGDDVYSYPLNWSQIAEEKGYSPYCNIEFELNHELPDTTELTGTTLDQSQEPAAPCSHGGKYSSPAV
SEGSISYIASTLCSLSEPLESLCAANHGNANSKTVTPTSKRSAYESSRSSPSYDECSSVHPGSSNALELRPTDQGSDDSDSEIFRVKRRSSLKMEKRTIS
DASPSNVSENKGFKRLKKLQPDIRCGRSVPLQSSSTSNNKGAPEIASRDDRFARGNTISRGSTVPLSIKFKKLTNEDSVRRQRDHHRSDLRQLELGKSRR
EPPPIEIGLKRFKVKGPSFLASENRRTVLPAKLQTKKRSRNWVSEFHQWVLHVDYMNKMQRNFFILIICFSPSLSPAMKITHFACPLSEGIWGQCGDPCS
SSAPGKANNSLIPVLCPFAITLTI

> FvJmjC17
MHGQPQNREAKNTHDGNKNAENHLGNRRPGKKAMQELPKRNQKWKKETNVRLCQFRFEISGSEKSGGLVELGGVRGR
GIERGEGQDSEAKPSSMLSFKSLLSKRTKAITKKKKNKSRNKIRSKTVPTKQNPICEKCPDEEEMEEAEDGDEVEEGFNLKASAPSHTHGVKPLGNLYLN
PGSVNSRNTGLGNLQTLTDELVLEILAFLAGTHLGVLASVSKSFYIFANHEPLWRNLVLENLNGRLLYNCTWKSTYIAAHYPSFDVSNVSVSGLRVRDFY
SDYLFQSWLCANLEMKPEWLERDNIIRRRGISVEEFISDFEEPNKPVLLEGCMDNWVALEKWDRDYLVKLCGDVQFAAGPVEMNLEDYFRYADQAREERP
LYVFDPKFGEKVARLGSEYEVPVYFREDLFNVLGNERPDYRWIIIGPAGSGSSFHIDPNSTSAWNAVIKGSKKWVLFPPDVVPPGVHPSPDGAEVACPVS
IIEWFMNFYGATKTWKRKPIECICKAGEVLFVPNGWWHLVFNLEESIAITQNYASRSNLLNVLDFLKRPNASTLVSGTRDRVNLYDKFRKAIETSLPGTI
DKLLQKAEEKKAQQSKPSFWDTVTDSKAGAFKFSF

> FvJmjC11
MEIYEAPPFRPRDRRPNALGNLLVLPDELICAVLELLSPRDVARLSCVSSVMYIFCNEEPLWLSICLNTLNGPLQYKGSWKKTVLHLENVPYERDKDGRKPLSFDGFDSLFLYRRLYRCHTTLDGFSFDNGNVERKDKITVEEFSCDYDGKKPVLLAGLADAWPARRTWTLDHLLQNYGDTAFKISQRSSRKVSMTFKDYVSYMKAQHDEDPLYIFDHKFGEVEPGLLKDYSVPYLFQEDYFDVLDKDKRPPFRWLIIGPQRSGASWHVDPALTSAWNTLLCGRKRWALYPPGRVPIGVTVHVNEDDGDVNIETPTSLQWWLDFYPLLADEDKPIECTQLPGETIFVPSGWWHCVLNLEPSIAVTQNFVNSKNFEFVCLDMAPGYRHKGVCRAGLLADDEGIIEDSTHIPYDKDDYNSSDMTRKVKRVRTLKPGEYPSSERTSNGAQGFSYDVNFLAMYLDEERDHYNAPWSSGNCIGQREMREWLFKLWVGKPGMRDLIWKGACLALNAGKWSKSLAEICAFHKLPSPTDDERLPVGTGSNPVYLMSNCVIKIFVEEGLETSLYGLGAELEFYSLLGNVNSPLKNHIPDILASGIIYLENGTYKIIPWDGKRVPDVIAKCNFIPEKVKEDVSPFGVWRKKQYEYRKAGLSTDKSINSVEYTRIWPYLITKRCKGKIYAELRDAVSREDELNLASFLGEQLRNLHLLPPPPLNISTSSDIEQESDRPFTNGSVEAVPDQSDIPAEWDMFIRTLSKKKNDVSSRLIKWGDPIPSTLIEIVHKYIPDDFAKFLYIFKDENGRSKVSKSCSWIHSDIMDDNIHMEPCGVNSCFIGNAKTTCLVKNGSLNVDGDSAQRKTWCPSHILDFSNLSIGDPIYDLIPLYLDIFRGDRNLLKRFLDSYKLPFVRQASPSDYIDGGDKFKRLSYHAMCYCILHEENVLGAIFSLWDELKMAKSWEEVEHVVWGELNNYKGFPSWIEPDTPPIL

> FvSET29
MEFCQSYALETWFCCNLGQSNYARHLLSHILDLLNGKPTEIVTPTRGIRQGDPLSPYIFILCAEALSSLISRAIDTN
MVQGLKMSPTAPVLHHGGLPYIRRRLPPASTPSPAASAPGLLRPWTRRATIPAALWHPPHAQPPPHAQQQATPCLAPPRQRVHQASFGLGPGLLRRRRKGGECTRPPPASTCVAWAEVDRTHLGWITPLAWKSHGDVPGDTVGLAQKMYQLKKQIQAERVMSVKEKIEQNRKKLEGYVSEISSIISREVELNGSSSLLNSRIQHPPCKFSGFSKGFGDKDYSNNEEELLSSSIELPKAHKIDPYTTWIWLDRNQRMAEDQSVVGRRQIYYDKEHGVEALVYSDTDDEMTEPEEVKRAFSGGEDRILLMAFQEHGTGEEVTKAVSQFIGATTSEIQERYITIKERVCEKRESKDSGESGSNRHIFLDKSLSAALDSFDNLFCRRCLSEKQMHWSEHDEDEKPCSDQCYLRLKAVENLVEGSHNNTLPRTNVTNSQRESAPTSSVTEIIRDENYIPGKDEAVTSERIHRSDVFTGALGLDTDMMMTHNENTGKRKVVEYTDKEAHDQTILPDDLEGSSKRQKRLDVLHLVTGTSTPIAVHDHSTSSEHGTSDAGLPNKNELELANKKSTGHTSKELVCFGSSSCDESMDDVKDEPKDVIEVLKQPSKSTEVQVEKKCGSSSEWKAVEKELYMKGLEIFGRNSCLIARNLLSGFKTCLEVSIFMHDAEASMPNRSVGFMEDNGKADTDQSEHEMRTKSRSFRRRGKARRLKYSWNLHVEGNVLVCKMEPAVKNIVDAQRAAKIGSGDATVQKVNAEVGSAHALPQIVNVTRMSVAIVGCGDSSLGEPPKQGDSQCGNMRLLLR
QQQRILLGKSDVAGWGAFLKNPVNKNDYLGEYTGELISHEEADRRGKIYDRADSSFLFDLNDQARDKLKFANHSKKPNCHAKVMLVAGDHRVGIFAKEHIDAGEEIFYDYCYPPETEIPWAQAEGTKRDDSSVSQQLRILCKKVCQLLPSGLNWLYMLI

> FvSET4
MASKASPSASANRSEPHQDPPMTGTEETAMTRDQKEISLIIDSLQKQVISNRGVSIKKRMEENRQKLAAVTNNFYNLSLERRNNSLLNPGKSVDLLTKRQQDAFKLQNGDESNGSNDSSQEDGRGSTAVLLESNVAVKNAVRPIKLPEVKRLPPYTTWVFLDRNQRMTEDQSILGRRRIYYDQNGGEALICSDSEEEAIDEEEEKREFVESEDYILRLVQLIMKTSMTIKEAGVSDPVLESLAHYFSRSPTEVKARYEALIKEEESMGISKNTDDEDLSKNDNSFLDKDLDAALDSFDNLFCRRCLVFDCRLHGCSQDLVFPAEKHAPRSPPDDENLPCGPNCYRSVRKSEGTVSSPMNDYLEEKGSKRSSGLSRKKGKSFQSESASSNAKNISESSDSENETRQDDTCSHDPISSKTKVAGKIGTPKRNSKRVAERVLVCMQKRQKKTMASDSDSIVDGGLCASDTKLRSNSCKDNEDTSSSSQKNLKSSTSGRSRRESPLKDSNKVVQGEVVDGSLNEMITDPPATSSDDNLRKEEHVDENIYKQECSDDKTWKTIETSLFEKGIEIFGKNSCLIARNLLNGMKTCWEVFQYMNYSDSKVSCQAGDAANSLVEGYSKGNNEARRRSKFLRRKGRVRRLKYTWKSAAYHSIRKRITDKKDQPCRQYNPCTCQTACGKQCSCLLNGTCCEKYCGCPKSCKNRFRGCHCAKSQCRSRQCPCFAADRECDPDVCRNCWVSCGDGSLGVPNQRGDNYECRNMKLLLKQQQRVLLGRSDVSGWGAFLKFVLDAYRKGDKLKFANHSPDPNCYAKVIMVAGDHRVGIFAKERIASGEELFYDYRYEPDRAPAWARKPEASGSKRDDTAPSSGRAKKLA

> FvSET38
MEAETVVCTTDQLIEEEPQYTHIHQNEFSYRRHKKQEEEDIVICECKYDEKDPESACGERCLNVLTSTECTPGYCPCGVYCKNQRFQKREYAKIKLFKTEGCGWGLLANENIKAGQFIIEYCGEVISWKEAKKRSHAYEIEGIRDAFIISLNASESIDATVKGSLARFINHSCQPNCETRKWNVLGEIRVGIFAKQDILVGTELAYDYNFEWYGGVKVRCLCGAPSCSGFLGAKSRGFQGDTSSRGSGVGSDTAMDSMIQSSTHEVDTSTRGSEVDSECVMDFTSEFTTYEIFKSRESLMLWAREAGKRNGFVVVTVRSDLGGKSLKPRVTLGCERGGKFKVHKERCDKGGKQRCGTGSKKCSCPFTLKGEKLTTGDGWMLNVVCGVHNHALTETLEGHSYAGRLSEEETSLLIDLSKSLVRPKEILNKLKQRDSLNVTTIRTIYNARQRQRVKDKAGKSQMEQLLSKLNEHNYLEWHRSCPISESMLDLFWAHPVSLELLRVFPTILMMDCTFKTNRYRYPLLEIVGVTSTNMTFSIAFVYLDSEKEDNYVWALNRLRDLLDGCAMPNVIVTDKDLELMRAVETVFPSARHMLCKWHINKHVFAKCRNLFESKDKWDRFIMKWNVLVTSPTEEDYIQELAVLHSEFSTTEVLDYVTNTWLNPFKDRFVSAWTDMIMHFGNVASNRAESSHSKLKRQLGLSQGNFEGSFEKIHALLELQHSDIKASFEKCLTVVRHSSWPSEFKHLRGVVSMFALDMVLSQFKLSTSIEIDDLHCGCVIRRTHGLPCAHEIAKYKKDCRPVPLESVEPHWRKLHMSPPSDLINNTMDVNEFPELELLQQYFFKTSPDNRLTLRKRVRELVPSLATLVEPKVKSRTRGQLKDIIDMSIQKKASAIELIQSASVDSLSPSVTRDTMEVLEKSTHKQTGPMKEKVYRTLSAKIMSYIDSFPAGLGPYIDEVKDVCDDGNCGFRAVADLVGLGENAYLQVRNDLLTELSSYSAHYGELYGSAERVRELAQSLSFFNEGRAPFDRCMTMPDMGHLIASCYKIVLFYLSATQCLTFLPLRSTPVPLPMRRHIAVGLVNDSHYVEVFLKPGHPVPPIECNWIRYRTQAAQGWDTIYTDCIAHFQSGVNGAGEGPELSFKMLKNRPKTKAFSIDIPDVTGPSSSEFLWRGPLSRLLLLLDVFAISYLNPHIAIFSQQEDTYVWEDDDERYSVEKIPLYDSAEDEPSMLFKTVNSNSEFDVKRMMINVNANSEHQLMSTALVVQSLDSDPMEGVVMEVKNEASEETKLYSQNNQPAFLKKNAMISRIRSNTACRNYHIESGSVSNKRLKQGSSGKLKHYAQKQVDAKSVALLLASNEAQEEILKHEEMRSDAATHLESLYNEIRPAIEEHERDSQDSVATSVAEKWIEACCLKLKMEFDLYSSIVKQVACTTRKASSESKYADSNIENEIKLLPN

>FvSET15

MASCEDLISVEEPLCESRGTEQQMGSEILVEQQSCLEAQPVLDGNVDPTSAADRSLGFWRNDNAGCVSCSEGTELGDVEREGLAGGLGSTCVLEAEDFLNSEYVSSRCGDQNDVDGGCDTESGRMQLKEGNWCREDGFAGSFADCEVPTEVLPSAGSPGNGIQLDDKSGNGIQLDECRDDKSGNGIQLDERRDDESANCLSSEGMAEVMEEQGQLPLEFVDVDVCRDYSIFSGNSLEAPYEKSVVSTGVEDVGLCNGISPARDGQMPSEVSNTGDIVTNFNWNNKHFDDNGVSGASSLSTEKSTEVFEACNQLLPSLRFQRSLEDLQMPNSPSNFGQQSDPKSNTTVDGSLADATEFMEEKSNVMSDKEAEIHTQISPVKVNLSCLKDNSSNVAPNCIIENSVPLQLCDTFSIANNSVDGLYADTVAEVVDMNSNIVDACNQTLSPDLLSVCTQENDQSSDKVKECMEQNVDGMTDMRVETGTQILLKEEKAFNLTEGSAGLVPNSTIDKSVALQLHQPFDIVSNDSSKTVSVPDVNYSPGHVESSTSFDHSGLMDHGGNEYFRVEHLPKTNFSEIIALPAQRSGRSRKTPTKKAPRKRRNASKVLQPLGSVESVFKGPGRKRSCHSKPPRLSTWGLLGSVTQSFEESNGLQVHQICQGQNEGSQIQRGGQRSGKQKQSGASGNLQWSKGPSTNHVRLKVKFGKEFNKNSLFTKAPEVVDTSTSANSIQIVNVVEDNWRQEATVRKCQYTNKKLEEETCQNGELANKDLECVSVTENSAEDEIQNCAGVHSHAIAVSSGGSVGSSYRDPGTSPDSEVINLIPEAHVEARPQEDCHGTVFTSDKVLSASGDFISSKREKKKHKLPSAGNCVQEDGSLSPCPASTMKAKPSKHDGCRRNCIQDFCLGETFTFSPCAKASSNSSSDKEFYVEPLCLSGESDHGVSREALTVERGAEAETDCNLDVVLGLQCSKNMLPSSNTKGQKPPKGKTRGFDSVSKRSSTRKPRENDQNSVNKRKVKEDKQLTCKVESLPESGDLFGDANSSHVAECIGVPNLDAVPVGLDKQYIPPRNAWVLCDACNKWRRIPAELADFIDETKCTWTCRENQDRDFADCSIPQEKSNAEINAELEISDASGEEDASGTRLHYKTLECRRPSVSQQNVASIKTNQFLHRNRKNQSIDEIMVCHCKPPKEGQLGCGEDCLNRMLNIECVRGTCPCRDLCSNQQFQKRRYSKLEKFRCGKKGFGLRSLEYIRKGQFLIEYVGEVLDTHAYEARQKEYAVKGHRHFYFMTLNTSEVIDACAKGNLGRFINHSCDPNCRTEKWMVNGEVCIGLFALRDIKKGEEVTFDYNFVRVIGAAAKKCHCGSPQCQGYIGGDPLNTEIIVQDDSDEEYVEPVMIPEDGVAEDSRGSAEARLDSLDHQYGAIIQHEESASTNKEIDRSTISVCKLDITMQRKESENQYSLELQHPLPSFVQPVEVFQPTEDVTSRSTPVIQQQVFREIGTAEKSSNSCERPEITSPIKVISKPLSDDIDAPASDSNKNSKVNTFEDEQLLSKVHRNVKTSHSSSFVKKGKVRSTPLNTNKIQVVANKSHVLPFKPKRSIEGSVEEKLNELLDTDGGISKRKDSAKGYLKLLFLTAQSGDSGSGEAIKSNRDLSIILDALLKTKSRTVLIDIINKNGLRMLHNIMKMCRRDFNKIPILRKLLKVLEYLAEKPQILTQEHITGGPPCPGMERLLLVHDIARNFRNRWIPKALRRHCFVDRDDGKMEFNRSSNYNRFPTSHDNWRDQTGRSTEVADSAKQSVVKTPPSASTVTQDGASTPCTGGCTTTETKVRKRKSRWDQPAVTVPDSKSRWDQPAVTCPDSSLHPNKEQKINCKQLEGDATLLPENQSREGGNCSSTVLHICEQVGADVVYAGKQNILDDAPPGFSSCLNTPVVSYLSTSSVIGHPQAKFVSRLPVSYGIPLSIMQQYGTPHAETADTWVVAPGMPFHPFPPLPPCPRHKKDPSHDVRHASVNQASEGQQASCDTTNCHSEESTPSTTGVTQADSGTPCANNQSGIKRERESSYEAPLGRRYFKQQKWNHPKLRPPWMRDRTGWGCNGNNF

> FvSET1
MKIRLKSESKVEICTPPYGTARGRVSTPADSSSFPDHFPSPASPLNSSLTARSPKTSNNVNLAGKLEFGGLTGDEKW
SGKLLESAGVEALPRQRCTVLGWSEMHSCDDNAVKRCGSVVEQGGSGVTEERVGSNYGGLQCWAWEKGGWAESLLVGLKKKSKMSRRVADDDEEVDEVGE
EEEYEVQDLRDQIKSSRGSRFNLITNELGLDQGSSTANSRRRRMRYYLSKESVINGVRGLSKGIGVIHPDNRWYRAWTKVILVWAIYSSFFTPFEFGFFR
GLQEKLFILDIVGQVAFLVDIILQFFVAYRDSQTYRMVYKRTPIALRYLKSGFILDLLGCMPWDNIYKACGRREEVRYLLWLRLCRVRKVTKFFQDLEKD
IRISYEFTRIVKLLVVELYCTHTAACIFYYLATTLPPVEEGYTWIGSLKLGDYSYSSFREIDLWKRYTTSLYFAIVTMATVGYGDIHAVNMREMIFIMVY
VSFDMVLGAYLIGNMTALIVKGSKTEKFRDKMTDLTKYMNRNRLGRDIRNQIKGHLRLQYESSYTEAAVLQEIPASIRAKISQTLYFPYIVSVPLFKGCS
TEFINQIVIKLHEEFFLPGEVIMEPGNVVDQLYFVCHGILEEVGLGEDGSEETVSLLQPSSSFGEVSILCNIPQPYTVRVCELCRLLRLDKESFTSILDI
YFYDGRKILNNLLEAKGPHVKQLESDISFHIGKQEAELSLKVNSAAYHGDLYQLKGLIRAGADPNKTDYDGRSPLHLAALRGHEDITLFLIQQGVDINIK
DNFGNTPLLEAIKNAHDRVSSLLIKEGASLNIDNAGSFLCTAIAKGDSDFLKKLLSNGIDPNSKGYDQRTPLHIAASEGLYLMAKLLLEAGASVFSKDRW
GNTPLDEGRMCGNKNLIKLLEEAKAAQLSEFPYRAQEIADKMHPKKCTVFPFHPWDSKEHRRSGIVLWVPPTIEELINTASEKLEFLGGVCILSEDAVSP
QAAVLSAMPAMRKHSERSHFGNVFNKLLKQIGSPVDFELPDWFNKWKPMNYTFIKRNIYLTKKIKRRLEDDGIFCSCAPSSPGSPTVCGRDCHCGMLLSS
CSSGCKCGSSCLNKPFQHRPVKKMKLVKTEKCGSGIVADEDIKQGEFVIEYVGEDQDCHCGAVGCREKLGVRPTKPKMSSVAALKLVACQLAVSSPKVKG
MFSAKDVYPNGGFPVGSSQHIRKDHSQGGTACNCIDEVIRIRRPINERSFGVIKRFDNHSKKHAIMFEDGGIEILDMSKEEWEFVTL

> FvSET25
MPNLANLSTSDSLAVAHCPNLKSLSVTDPPEHSCSVKTLGVDGDWDQRRMGLATLPNGNGAGSSNCKSLEEHVRDWV
CRRLELGISESRSSLPFLYGAKKLAECQICHHFVYPGEEILCSVRGCQGPYHQGCVKERLRISNMKKFTCPQHVCFICRQRLHWRCVHCIIASHDKCAAW
PDKVIHLVDQPGRAVCWRHPTDWREDRKGLLIWPSDDRCSISGWLTGMVYLLNEQHAVSASNTEEVFFRLPLPYTEEEFKIDLMWKHRENNVEPPPYTHI
RHIYLVKKKRDDDDDDDVGCSGCTSVCSVDCVCRLQCISCSKVCRCSENCTNRPFLEEKKIRIVKTAHCGWGAEAAECIKKGDFIIEYIGEVIDDALCEQ
RLWDMKYKEVKNFYMCEIRKDFTIDATFKGNPSRFLNHSCDPNCVLEKWEVEGETRVGVFAARSIEVGEALTYDYRFVQFGPEVKCYCGASNCQGYLGSK
KKICKMDLFWGKKRKRTPTACIAILRESLSTIS

> FvSET32
MEMSCQSNGNSSDIQPVCNSGGTSYQDKSYSGYMPPSPSFVSGWMYVNEQGQMCGPYIQQQLYEGLSTGFLPDELPV
YPVVNGALINPIPLKYFKLFPNHVTTGFAYLSLASISSASTPTNSLKSCNGDLATSSIPTPIATSYPDLQNDSTSQANSNTDFSSKLILKSEAPNQDTSY
QSLSSKESCWLYEDEEGKRNGPYSLFELNSWHQYGYLRDTLMIYHVKNKCKPFTLSSVKCSWKLDGSETITKFDTKCNQSGSFVSIISEVAEDVSSQLHY
GILKSARRVVLDEIISNVIAEFVTTTKAQRLNQSMKTCSLDAKRSEIDGENPALSSEAGAADCVAQRTFINQVSPEPLPNTKSVGSIHTYWDSYAVVCGM
LFNHCMEVMWNAVFYDSVAEYSSAWRRRKLWTGSPSFWIPPNRCGDRVEKVTVLPHENLSDGYDDDCPPGFELLGTELGCHAQPSLMSSSVLMVEKPTKK
IGPSYEDMKYIVEHIENELHLSAKSSLTEYVGSIVDEEVSKRVNSSKEENSMKDTVVCSPRNMGGSSDLCHKLKTSNNTSAEIILADSLTPQAEKPFHNS
LPENRMSNFLVSAFKEVCSYVDDAVVDQEVNEPSPHGLIANVKTLGQSPVCKFRPSRSEESIPKIGAYVATAMLRKKLHDDIIRELKSFIDLALNKFLAS
WRTSRKNHICNEEKACNTNKKESKHRLSSVTAEVSPVMDEYTYQRKRLLRKKSDSSGLVTVGLKSGETVEKSKKLHVARDVLNNATLISKKRSLSKPQTE
SSVDAVSVKVNSKRVSSTDKSASKNASIRKPLRGYYEQSCEPINDVIDGISSIRGPQIELSIGAIALQAIDTSCSSADTVAANNASSQKRLKVSLAVEGI
EPMKCMPKPSKRMVSAHVNNDIEKVVNSHGHNGQLKGEPLPKVLKQKRERPVGGSQLPHPKKVLKVADDVLKQAANIPGSVRKARSTKSKTSNPCPKSDG
CARASINGWEWHRWSLNASPAERARVRGIKYVNTQQLTSETNTPQLSNGKGLSARTNRVKMRSLLAAADGADLLKSTQLKARKKLLRFQRSKIHDWGLIA
LEPIEAEDFVIEYVGELIRPQISDIRERHYQKMGIGSSYLFRIDDGYVVDATKRGGIARFINHSCEPNCYTKVISVEGEKKIFIYAKRHIAAGEEITYNY
KFPLEEKKIPCNCGSKSYMMCNYLWLGKLGTALHLLNLTLTGLDCDVSRKRWGGCLGAFSCFGTQKGGKRIVPASRIPDGNASAIQPNGPQAAGLANQAT
GLAPSLLAPPSSPASFTNSALPSTAQSPSCYLSLAANSPGGPSNRMFATGPYANETQLVSPPVFSTFTTEPSTAPLTPPPELAHLTTPSSPDVPFARFLS
SSADIKSTEKTNYAANDLQATYSLYPGSPASLRSPISRASNDCSSSFPERDFPSQWDPSVSPQNGSYPRSGSGRLYGHDTTGPSVASQDSNFFCPATFAQ
FYLDNPPFPHTGGRLSVSKDSDAYSTSGNGNGSQNRHNRSPKQDVEELEAYRASFGFSADEIITTTHYVEITDVMDDSFTIGSHKSPPEECTEPKSAVAG
HSDLPVLTNGYEDNKSRRQPCDVSRSSTPGIHTVTNEEDIFSKMGSSKSSRRYPMGQSCSDAEIDYRRGRSLREGKGNFTWHDKE

> FvSET24
MAFPLTQRNDDVPIDVDTAAVAPIRYLSLDHVYSATSPCVSASGSSNVMSKKVKARKLNHFDSDDVSDHHHHHKPLP
PPPPPPPEHKPEVVLVYSRREKRPRHSFFDALVARAQPKAVKVEAVDEDEYVRLKKKRKESKFGSSELVKLGVDSNVLLALSAPPRLRECRVSNQKPEKS
SSKKRNSSVKAEKVPPSVKRWVGLSFSGVDPKTFIGLQCKASALFTGHWMLIHIAVALWDTTPTVEYEDGDEEDLVISNEKVKFYISREEMESLNLSCSL
KNMDSDVYDYNEMVVLAASLDDCQELEPGDIIWAKLTGYAMWPAIVVDESLIGDREGLSKTSVGGSVPVQFFGTHDFARIKVKQAISFLKGLLSSFHLKC
RKSRFLKSLEEAKMYLSEQKLPRRMLRLQKGINIDDCESESGEDEMRTDSGDGCLDDVMIPRSLDFPGTSVLVMGDLQIIRLGRIVRDSKFFQDERDVWP
EGYTAMEVLRDPESKIRPLFQVSLDNGEQFKGSTPSACWNKIYKRIRKIQNSALDDSNANAEDGFEKIYKSGSEMFGFSEPEVAKLIQGLLKSSHASKVD
KCKSASRRHRDVPVGYRPVRVDWKDLDKCSVCHMDEEYENNLFLQCDKCRMMVHARCYGELEPVGGVLWLCNLCRPGAPEPPPPCCLCPVIGGAMKPTTD
GRWAHLACAIWIPVDDDEEGQCIRFLSFCKKHKQPSNDRSMAGDRIGRTVRRCSDYSPPSNPSGCARTEPYNYSCRRGRKEPEAIAAASLKRLFVENQPY
LVGGYSQHQLSSNPLPPNGVVGSKFSSRLERLKASQLDAPTDILSMAEKYKYMRDTFRKRLAFGKSGIHGFGIFAKHPHRAGDMVIEYTGELVRPPIADR
RERFIYNSLVGAGTYMFRIDDERVIDATRAGSVAHLINHSCEPNCYSRVISVNSDEHIIIFAKRDIKRWEELTYDYRFFSIDEQLACYCGFPRCRGVVND
VEERGTKLYAPRSELIDWTGE

> FvSET16
MENAGYLSCQRGSQDLRPAMLGWLQDPLLSSIQSSGSHRSEHVNLGNSFLSLLSGSPSLLQRGFQDFSNSQPICTSG
KILPVGNNSILNSTQSTIPLSSTGLPSEKLSWTNLQSGTDFCHNGSSKVVPSSICASNSVLHDLQSSDLAKVVICHTGPVNEKLESSYALSREWHCAGPA
SRANIQNSERMPLEANSFISNQAYRVCHSASPADSWKARRESIHTSQTIPLEANSFISYHASSLWHGTNPADNGKACRANIETSPKMPQASSFMNGCPRV
FCSTTSGYLLFSNTGFLGIVCSCHSFRMSAFKFCEHSGLYGVNPGDAIRMDSGETISQWCKLYLPKFGIRIPGDKSEWDWPEELSATASLMKRSVPMPKI
SNSSSDLVFTRGGSVSSKQSFDGVPLSKNLITCQSLVISAVSNKPEGNSQDSNNPFLKALTGTSQSNLQMADNMTMERAMATSKLVGNGAEDSCQFISSY
TGSVPNRTSIAHPPLQERRINGKESDFRRIENTRDGAFRDAAISNIELRLGQPYQLAQTSGNTDLSAVGPPLLGTVVNPMKSLFPQQMNASRANCREEVE
FMQCDRLSANPSNPSRNRNWNQLNHGNNAFVIRNGTDDERAQNSVISLLTNLKSPCKENKPSKANNSMFNVSGNSMRNTLHSEPLSDKNDLATVWRSGGN
SERQLDMSHLGSYKLNDNDKGLSSAAHASQLAKDLGFRIRKEMEVSSSFNRLSGNGDPNFSTAHRNSCYSHQLSGVPLGTPESKIMSNYPEKVNSLANSG
QVDHVYLRPMASSMGSGIPTQAVSKGIPVSASTSLADLIPPFYREEFVGVHTHLPDDTLQVHATRQMQEISKLPSPSKNQGEGRVGCSTYMQQSRVDTSA
SGKQSHKLSLSDKHDVSEAGVNPHPSDVTCRIGTDEGVNCCCQFSQYKQGNAIHFKEVGLKHQTSVVPLCKEQPSPRSEKSKNVPEPSEHERCCHKVPCG
NFRGSSSHAAYRNCLEMNSESRVGSFSAVSKVQMGTVNSEASMILSPQFSNSHLIPKDKTVSLDHKRKLSGEVTKNNAYHTSQWRDVPSKVKGVSDVTRV
DRLANLFDATREDREKLGDTCVKCFNGTVQIADSMKEHEVSNISSGCSAPVVSQPSIEFNNMESSTNDPGDHGCGSNFVVDEGSGIDKAWSSDDALESER
SAKFLASTGSSLKKVGAPKNLNHESSSCLLDDLKLLNSLTWQKGRDQIPAGLALRDKDKHLQNLEQGLKIGKRKRELALELNASCSNSDSSRVRQENHNS
NGTSQFTSQPSKSLMMLSTSRKSGTHVTGNCITQSSSKPRLHISSSAKKLLLRSDLHKLHDDKESEVNNVFQTELNGGANNHELPEVSGGKTCKRDCSSN
AFRQFQIQESSRKDTKRTKYNSVDGFKSTCSQQVKIGHRKARPIVCGIYGELTDGSSTGRMSKPAKLVPLSRVLNSSRKCILPKLCNSKSSSMRKKKLGG
AAICNTYDLKTEKYKCHDAMVKVNDTSMRKKKKECSPGEREIHKELFSMEKQGDVQSEKDHQKLDSITHTQLQMKPKEIRKRSIYEFTEKGDDTGFKSSS
VSKISNFRPANDGKLVNTGEDSGLCQHSAKNAVLLKSTGAIVIATQIQFAASVVAQTKMKSIFYWSVVNVQSEYVAIQSIFNFSFLCSFSCKYSFVIDRL
VILSNVVINRCIRLVMVCPKYLRAVGLADHVCVLCGYGGGAMTQALRSQTIAVSILRAWNIETECGPKNELCSIKTLQKDSTGLHCSGYRHSESSSLFVS
QQSGQPLAAAHCKRGMSYRVDGVENSPSVSKTKVHNSITMGLVDSATKQWVHMVCGLWTPETRCPNVDTMSAFDVSCVPLSTDDASGRRLKVRTSVRSPS
SGAGDGAPPSQKTPAASPTTSPPRRVRFFQFPARSTLFEVLGDLAGKPEKNGLAGEGKWSGTLLESSGVEARRRRHRRVENGRTSALDVRTLSRGLLQTE
VEGVDNENVGFYGRCGLHATHPIYKSEYPVDTEAGCLDEKKLVCARTEGYKGRKRDGFRHNYCDRSKGSDGCLVPQEQLNAWAYINGQKSCTQELPKLAI
SEIEHDSRKEYTRYKQAKLWKHLVVYKSGIHALGLYTSRFISRDEMVVEYVGEIVGQRVSDKRENEYQSAKKLQYKSACYFFRIDKEHIIDATCKGGIAR
FVNHSCSPNCVAKVISVRNEKKVVFLAERDIFPGEEITYDYHFNHEDEGKKIPCFCNSKNCRRYLN

> FvSET42
MPSLKRCKLGDSEEEESSGRKKRKTNGYYPLNLLGEVAAGIIPVSFRGLLGAEKGGFSWCTEVSCSPPPPEEEEEEA
VVESKSKAGKSAKAKAAEVSRPPLVRTSRGRVQVLPSRFNDSVIENWKKESKSNVRDNVEDEKPSLKPQKNGKKVRSNAERIGYGSKKYSGLCEDEEEEE
EEEEEEEEEEEEEVEEEEEEEEGYMPYKSYNMRKYNSGSRSTLTSVHEHLVGNGRCSVVEIVDEHEDDVEEAVRISKQRKDGLYGPEDFYSGDIVWAKPG
KKEPFWPAIVIDPMTQAPELVLRACIPDAACVMFFGYSGNENQRDYAWVKRGSLFPFMDYIGRFQEQSELGNCKPCDFQMATEEAFLVEQGFTEKLLADI
NMAAGNPVYDESLPRGVQEATGSNHDLDYQFVDQASSPKITFFQRDIYRKNKYTRACEGCGSDLKLPKKLKVPTSGGHFLCKSCAKLTKPKHICGICKKW
NHSESGSWVRCDGCRVWVHAECDRINTNYFKNLGGITDYFCPPCKVKFNFELSDSEKEQPKVKSNKNEAQLVLPNKVTVLCNGVEGIYFPSLHSVVCKCG
YCGTEKQALSEWERHTGSKSRNWRTSVRVKGSLLALEQWMLQLAEFHENALVSVKPPKRPSIKERKQKLLTFLQEKYEPVYAKWTTERCAVCRWVEDWDY
NKIIICNRPEVSFASDEKMEPALGILSIPSNSFVKICVICKQIHGSCTQCSRCSTYYHAMCASRAGYRMELHSLEKNGKQITKMVSYCAYHRAPNPDTVL
IIQTPLGVFSAKSLLQTKKKPGSRLISSNRIKLEEVPTVETTEPEPEPLCSARCRIFKRLKDSRKRTEEEAVAHQVMGHSHHPLEAIRSLNKFRRTENDR
VCFGRSGIHGWGLFARRNIQEGEMVLEYRGEQVRGSVADLREARYRSEGKDCYAYIYPCLQLFKISEEVVVDATDKGNIARLINHSCMPNCYARIMSVGD
EESRIVLIAKTNVSADDELTYDYLFDPNEPDEFKVPCLCKAPNCRKFMN

> FvSET7
MTSLHVTTPKPPLLLPLSQKLTISVPSATTKHILSKIPILPLSHPQMPPATTSPAGRPAQRLIRFNRRTSAPRRHLS
PPQSPPPKKFKPLSEVMERARFAVVERIDYSDVRCEQCGSGDRAEELLLCDKCDRGFHMKCLRPIVAILPIGSWHCPKCSGHRRKKIIDFFRIQKCSDDK
DKCASPQDATKRRRRSGSLVLQKRRRRLLPFIPSEDPAQRLKQMGSLASALTALHMEFSDDLTYMPGMAPRSANQSKFEDGGMQILSKEDTETLENCRAM
CKRGECPPLTVVFDACEGFTVEADEQIKDLTLIAEYTGDVDYIKHRANDDCDSMMTLLLALNPSKSLVICPDKRGNIARFINGINNHSLEGKKKQNCKCV
RYSVNDECRVLLVATRDIAKGERLYYDYNGYEHEYPTEHFV

> FvSET41
MVAALRKRTRALNPQLVMETGSESENDDVCCEECGSGHSPAELLLCDGCDKGYHLFCLRPILVNVPKGSWFGPCCSK
QKKPKSFPLIQTKIVDFFRIQRNVETLEKLSQDNRKKRRRGSSLVVSKKRRKMLPYHPSEDPAVRLRQMASLATALTATGTEFSNKLTYRPGMAPREANC
PDYEQGGMQVLSKEDFETLNLCKIMMERGECPPLMVVFDPQEGFTVEADKPIKDWTIITEYAGDVDYLKNRENDDGDSIMTLLSAADPSKSLVICPDKYG
NIARYINGINNHTREGRKKQNLKCVRFDVNGESRVLLIANRDIPKGERLYYDYNAYENEYPTEHFV

> FvSET33
MEPLNGIDKSRVLDVKPLRTLTPVFPTGAQAPNFGCMPPFGHTPNGFSPFFPFSAPPQAQATQHSPDLNHVNPPAMQ
TPAGPMPAPLRSYRAPNPSGGLPHDFPEESNGDRESSMGDEDGYFDGHKDVAGSSSSRKKTTRGSYRKKVRRNGDADAVVLGSSVNFVSVISPAQLEDGN
RDLVNYVLMNFDAIRRRICQIEDTKEKNTDSIKRADLKAGNIAMTKKVRTNMRRRIGPVPGVEIGDIFFFRMEMCVVGLHAPSMAGIDYMTLRGDLEKDP
IALSIVSSGGYEDDTDSSDVLIYSGQGGITNSKDKQVAVTDQKLERGNLALERSLQRGNEVRVIRGLKDDTTPNSKVYIYDGLYKIQESWTERGKSGGNM
FKYKLVRVSGQPDAFSVLQTIRKWKDGFSSRAGLVLPDLTSGSERIPVSLVNEVDNEKGPAYFTYLPSLKYSKSFTLTQPSLGCKCRNACLPGDMNCSCI
QKNEGEFPYTGNGILVSRKQLVYECGASCPCPPNCKNRVSQSGVKVRLEVFRTKDRGWGLRSWDPIRAGAFICEYAGEVIDEAKFKNKGDEGEIDEYVFD
TRRNFDSFKWNYEPGLLDEESPNDSVEAYSIPYPLIISAKNAGNVSRFINHSCSPNVFWQPVLYEQNNQSFLHIGFFAIRHIPPLTELTYDYGVSMSGGA
GNNNGPHRKKKCLCGSSKCRGYFG

> FvSET40
MEHSSGGQDSIPGIGSFDKSRVLDVRPLRRLVPVFPSMPHSAAAPPFVCVSPAGPFPPGVSPFFPFFISNENAVPPS
TAGVTPNRYTGASNGGDTTSRVQSPVNSDDTRTVGRPKGKVQSQKRTRGGQDIDVQIDAIVDNILASFTNTLRQSDGDKELVRYALLSYDLLRRRISQIE
EIKEGVSRIGRSDLRAGTLFMNKGIRTNAKKRIGAVPGVEVGDIFFFRMELCLVGLHAPTMGGIDYMGVKNSSEEEPLALSIVSSGGYEDHMQDGNELIY
SGQGGNANNDNRKDKEIKDQKLERGNLALEKSLHRGNDVRVIRGLKDVSNPLGKVYVYDGLYKINESWVDKGKSGANVFKYKLVRLPGQPEAFSVWKSIE
EWKETNATRVGLILPDLSSGAETFPVSLVNDVDGEKGPVHFTYIPSLKYPEPVKLAEPTAGCNCTGGCLPGNSNCLCIQKNGGYLPYTSNGLLVNQKSLL
HECGSSCKCSPNCRNRVSQGDLKIRLEVFMNKDKGWGLRSWDPIRAGAFLCEYAGEALNVSGIEKLGDDHADDYSFDATRTCQPLAVLPGDSNEAPNVPF
PLHISANTAGNVARFMNHSCSPNVFWQPVLRENKNEADLHVAFYAAAHIPPMTELTYDYGLNPHGKAYQRKKLCLCGSTKCRSFFY

> FvSET34
MGPVEGEKKTPLRFRRWIMVVQSAEGMASVESSIDVEEAADAAAASPHSDEKKRRLSSRIQVKQKPQKAFLVRQRVE
LLDEHEHGPRKKPNVADSEELPKACDSVDKSDRVRAKEALRLFNKHYLYFVQEEEKRALKAEALKKASKAAKKKKGAKNSKGSPETKVAKRPDLKALTKA
SVHHYGIDVGHQFYSRAEMVAVGFHSHWLNGIDYMGQSYSKGKYSNYTMPLAVAIVISGMYEDDLDNAEEVVYTGQGGHNLTGDKRQCRDQVLQRGNLAL
KNCVEQNVPVRVVRGHDCKSSYCGKIYTYDGLYKVVNYWAEKGISGFTVFKYRLKRLEGQPLLTTNQVQFINGRVPQSISEIRGLVCEDITGGLEDIPIP
ATNLVDDPPILLYPSIPGYTYCKSIQVAQDVKLPNDASGCNCKGSCVDSKTCECAKLNGSDFPYVHRDGGRLIEAKDVVFECGPKCGCGPSCVNRTSQRG
LKHRFEVFRTPMKGWAVRSWDFIPSGAPVCEYVGILRKTEDVDSASENYYIFDIDCLQTMKGLDGRERDSLKFEVLGDLAGNWKRTDSPVRESGRGGCWS
LLGWRHATAGAGRRRTEGRPHFKDCADRRSQAVCIPTVNSLERPDDQKSDNVPEYCIDAGSNGNIARFINHSCEPNLFVQCVLSSHHDIKLARVVLFAAD
NIPPLQELTYDYGYALDSVLGPDGKVKKMFCHCGAVGCKKRLF

> FvSET26
MDTRSVGVFDMLDMARHKRFKADVRTRLKVRTSLHLPSDGDGTPPPQRTPAAKLEFGGLAGEGKWSGTLLESAGVEE
RRHRCRSVANGGTSAPERAFATISMGEPRVSPGRQNRRKRMSSPDAKKDGRKKAKVDGDPDTSEVNNLGISWFAPLTVLSNSSITSIKKFRETRRINHLL
RTDEFHLTRKSRRDLVQESSSKVDENDLPGLVCNDLCNGKENLCIPVTNEVDDPPVAPEEFEYISSTRVPKNLVTPASAEGCNCKGKCTDTRTCSCATLN
GSIFPYVGKAGGGRLFEPKAVVFECGPNCGCGPMCLNRTSQQGLKYRLEVYKTPDKGWGVRSWDYIPSGGYVCGYTGVLRKTSDLDNVSGNDYFFEIDCE
HTMNGIGVREKRLGEVPPIVNGHIAGVDGDGKLLEESDPDYCIDAASSGNVTRFINHSCEPNLFVQCVLSSHHDVKQARIVLFASENIPPMQELTYDYGY
ELGSVVDTDGIVKELPCCCGTADCRKRLY

> FvSET45

MATPKDSDSLLERRVSLRLQYSQQKPFYGTRKRTPQQPEATGRNGVVLSKKAKVKLSSPAKKSESKTMKLGRHKVDFDEAGNCESEELENAKVESEEDKDCEGENKSVSVKERDMSVYAPSGARIDDQGKSYVAKLKETLRRFNLHYLHFVQEEEKRCTKLENESECRDDLKKPSNGADKKAVSENGSGSLGVLKKQSKRPDLKAISQMLINKEILYPEKTVGHLPGIEVGQQFFSRAEMVVVGFHSHWLNGIDIIGKSKGKDKFKGYTLPIAVAIVLSGQYEDDVDNSDEIVYTGQGGNDLLGNKRQIQDQVMRAGNLALKNNMEQFIPVRVIRGHKCDTSYTKKVYTYDGLYQVHHYWAEKGVAGFTVFKYRLKRLPGQPKLISNQVLYTNGKGSKAQSELPGLVCKDICNGLENIEVPVTNIVDIPPVAPEGLTYITTTEVAKSVCIPPRARGCDCEGNCTNSRTCSCAQLNGGDFPYVSRDGGRLVEAKAVVFECGPQCGCGPNCVNRTSQRGLKYRLEVFRTHDKGWAVRSWDFIPSGAPVCEYIAVLRRNDEIDNISEKENEYVFEIDCWQTMNGIGRREKRMGTVAIPNSDAISESDPEYCYDAGSRGNVARYINHSCEPNLFVQCVLREHHDVSLARIVLFAADNIPPLQELSYDYGYELNSVVGPDGEIKKLFCRCGAAGCRKRLY

> FvSET18
MGALKGSTGEPRVSPGQQNRLKRMSSADAKKNDRKKAKVDDDTDTSEVNSLGISWFAPLTVLSKSSISSIKNFRESR
RVNHLISTDEFHLTRKSRRDLVQESSKVVENDLPGLVCNDLCNGKENTCIPVTNEVDVPPVAPEEFEYLSTTKVAKNLVIPASAEGCNCKGKCTNTRTCY
CATLNGSDFPYVGKPGGGRLFEAKAVVFECGPNCGCGPKCLNRTSQQGLKYRLEVYKTPDKGWAVRSWDFVPSGGYVCEYTGVLMKTSDLDNVSVNDYFF
EIDCEHTMNGIGVREKRLGEVPPIVNDHITGVDEDGKLLEESDPDYCIDAGSSGNVARFINHSCEPNLFVQCVLSSHHDVKQARIVLFASEDIPPMQELT
YDYGYELGSVVDADGKVKELPCCCGTAECRKRLY

> FvSET30
MGVVEANPHLDSSSRTVVSLHGSHSEARLGKKPMDVGECSYSPSSGKIKRRLVSAVRDFPPGCGRNVLLNNGVAGTS
RTGPMEGSSELEASVGASQSVPTENSVAGDRINDGNEVDDSDMMSVPVETRTSLEDEVSDLQANLCQLSNNSTIVEGASPVGTTDQAEQLIRRDRNDDGQ
KAVSMILSAGQVGGDSDLMNRAVVGTVETDELTALDHEGSDLSLNPYLVRMATQDVQMVSVMSDQNSASISVSNSGQEKNAARRYPPRRHVSAVRDFPPF
CRRNAALEARNFSEEQSDMGDKPSSSKMNTIMQQAGVGDVREEEFHKNELGGNDYEVTGDGVQTERKGHDVEEMERKDECNNGMKLVLEDTRKNEIVPSQ
EESNECKGTREDGIHSEKKVGKQIVVYHEKNSPGGNIQEDRVIVMGLMAASNCPWLKAIEVEEPKPNGGMSEGKQKKPYGMSGSKRKKPDGMSERKQKKP
SAGVSESKQKTLHFECQPEGSNTTPRTKSDSKIGRKPRKTNGAGARETANQGTSQQLVIRGEDAVPISCYTHVSHVCPPPFCQSSSSNEVCDGGAIVTRN
KVRETLRLFQAVSRKLLQEDEAKSKEGGTSRKRYDLQAAKILKEKGKYVNVGKQILGAVPGVEVGDEFHYRVELLMIGLHRQIQGGIDYVKHGGKILATS
IVASGGYADALDDSNSLIYTGQGGNMINTEKEPEDQKLERGNLALKNSLDEKNPVRVIRGSESSDGKSRTYVYDGLYLVEKCWQHLGPHNKLVYKFHLDR
IAGQPELAWKELKKSKKFQVREGICVDDISGGKESIPICAVNTIDDEKPPSFEYITSMIYPYWCRPLPLLGCSCTAACSDSEKCSCAVKNRGEIPYNFNG
AIVEAKPLVYECGPTCKCPPSCHNRVSQHGIKFQLEIFKTKSRGWGVRSLNSIPSGKFICEYIGELLEEKEAEARAGNDEYLFDIGNNYNDNLWDGLSSL
MPDAHSSSYEVVEEGCFTIDAASKGNLGRFINHSCSPNLYAQNVLYDHEDNRIPHIMFFAAENIPPLQELTYDYNYMIDQVRDSNGNIKKKNCYCGSPEC
TGRLY

> FvSET14
MGSLLPILDLNASPNPSQPATAAAAVALKVPKIEPKAEPYDDPMPPPQTTPQPPLDLFPNPQITPPVAPQITPPVVP
AADQDTVYSEFHRIQELFHTAFAKGIQNCDGGGGGGCDEDGVVLDPESGAIVPVEDASQKQQLAEVVQRKKYPQRSNELVRVTDLREEDHRYFREVVRKT
RMLYDSIRINSIAEEDRKNPGQGKRTRGDLRAASVLRDRGLWLNRDKRIVGSIPGVYVGDLFFFRMELCVVGIHGQVQAGIDYLPGSQSSNREPIATSII
VSGGYEDDEDAGDVIIYTGHGGQDKFNKQCAHQKLEGGNLALERSMHYGIEVRVIRGRKIQSVVSQKVYVYDGLYRILETWLDVGKSGFGVYKFKLLRIE
GQPEMGSSILKFAESLRTKPLTVRPRGYLSLDLSQQREKIPVRLFNDIDADQDPLYYEYLRAPAFPTHVYHQSANGTGCECVNGCNQNCICAMKNGGEFP
YDQNGFLLRGKPVVFECGSFCRCPPSCRNRVTQNGMKHRLEVFRSRETGWGVRSLDLIHAGAFICEYAGVILTREQAHIFSMDGDSLIYPHRFADKWAEW
GDLSQIYPDYVRPTYPAIPPLDFAMDVSKMRNVACYMSQSSTPNVMVQFVLYDHNNLMFPHLMLFAMENIPPMRELSLDYGVAVADEWTGKLAICN

> FvSET13
MAPPSPQVAKAFQAMTAIGLTEQQIKPVLKNLLKLYNKDWSLIEAEEYRALADAIFEAEDAKVAAEKKKCNNYDEDI
DAESQPNLEPELPLTRLRSRSQDESSSQKKKSPDEELVDNIEEDFLVPHRPLKRLRLQNQECQVSPSSNTCNPMSGGTLLIKPKVEAEELLDARSAQQPQ
NTSHSPESRPPVSLQSGIKDKGKQPLISKPLALQGKSLSERSSNGVRFKETVVEPRIVLLPKQNVNSLALIEPKDEPFTDDMAQDEVPIAVIHPDESSQV
NPPLSTEGATGIQHAELVASQERESRNDIPALSNEGSMNNELATIPEGTPSNLGESSCLEVASSPSGEVKLSLSCSSAIRRPGFHMPNLDAILKLTEEKC
LHTYRITDPNFSLKNLLGHMCESFLELATNSNDESQDGPINVVPNLDSLPKSPAWDAVTDPEVPRVPFPLNGDCPQVSGSIVSNGFSEDNVEDSLGLVVV
QNSDLTPDDLRTIHDINDIARGEERVKISWVNEWSRSRPPSFFYISENIVSKDADLKICLSSIEDKNCCATCFGDCVSASTPCACAHQTGGKFAYTPEGL
IKDDLLEECISMTRNPQPDHLFYCKTCPLERIKNDDCLESCKGHLRRNFIKECWIKCGCHGQCGNRVFFTREGKGWGLRTLDDLPKGVFVCEFVGEILTN
KERHQRKIQSTRSGKRPYPVLLDANWGTKSNLKDEDALCLDATKYGNVARFINHRCLDANLVLIPVEVETPDRCFYHVAFFTTRKVDALEELTWDYGIDF
DDHDDPVKVFNCQCGSKFCRNMTRSNSKCTENLPML

> FvSET11
MDIHQIQSPPSKRNAPPSLFFQCSDLILPWLTPQELAIASLTCTTLRNLSNSITLRRASDASRSLEPLPIPFRNSLD
QHPYAFFLYQPTSSPSSNLVRQSWGSTHQNPDTETRTETSSLCFVDETGNCASSGCGCEETCEDGCPCVVGFGGFDGVVFECGPSCGCGLSCGNRVTQRG
IRVKLKIVRESRKGWGLFADQFIPKGRFVCEYAGELLTTKESRLRQQMYDELSSGGHFSPALLVVREHLPSKKACLRLNIDATRIGNVSRFINHSCDGGN
LSTALVRSSGALLPRLCFFASDDITKDEELTFSYGETRLNSKGLQCFCGSSCCVGILPSEQT

> FvSET17
MEHAVVYIDTFRAGSFDQLKQNATKEKISLWKQLHGEAAPFKEMCQVAEATKPDLVIFGMDGSIGHWLVHLIKLKHL
SKVLMELELLLLKWMVMHAKGGGALSVVVATKSPDIFIGIREHMDQFDVFDVEVLNLLQLTSILPGVELGDVCFKPHEYYTLRLRWRVAAVANCDPQIGA
PDRWCKFAVKCPNKSNKNRSRRLSEKGWTGIFVGRRGIRPDAVTFLALLSACRHSCLVVLGEQFFQSMTKDYNLLPELEHYACMLANCYAAEGNWKGVGR
LRKKMKGKEMVEWIEDGVLVDDEWNGDEEMRKKMGLDFGRWGWRFVVEEKVKMVIWVHDRMMEEWQPKFEGALERWRFNGGDKLSDHLPWSGGAFSLRVD
GCRPWKNVVGSALGTEVGGPLLMPAEIDGSGAARWWLLAVVHGQHISLTSNPMGLVVVLIVVPPFWVKVLSIGSLVSMVTLVGCKEISRLLDGGRNRLHL
DPFRFMYTDHVLPVAFKNFSPSKRNTPPSLFFQCSDLILPWLTPQELAIASLTCTTLRNLSNSITLRRSSDASRSLEPLPIPFHSSLDQHPYAFFLYQPT
SSPSSNLIRQSWGSTHQNPETETRTETSSLCFVDETGNCASSGCGCEETCEDGCPCVVGFGGFDDGVVFECRPSCGCGLSCGNRVTQRGIRVKLKIVRDS
RKGWGLFADQFIPKGQFVCEYAGELLTTKESRLRQQMYDELSSGGHFSPALLVVREHLPSKMACLRLNIDATRIGNVSRFINHSCDRGNLSTALVRSSGV
LLPRLCFFASDDIKKDEELTFSYGETRLNSEGLRCFCGSSCCLWISLHSLKYLGEEESLAEHIEFCIELQIGSRRGIRRDKTVFGSEMSSKELHNDGDAL
AIAGTILGLQQL

> FvSET35
MAPSRETKEHLANAYKAAKAWGISKEEIKPVLRNLFQAVGGRWEDIEPDDYKIVVETYFESNRKGDDEHIDQKLRKR
RHLADEEDQASPGSGSSSELSLRHSSGNGNDRPTCHLASGDGSVHRERASASAVPCSDHMNYFKKPKDKVSTDDATFSAERTSVRRPGSVNGGARIQRPI
TREDKKPPKYISDITKSTEKVAISLIDEVGSESLPRFNYIPQNIIYENAHLNISMSRIADEDCCLDCLGDCLSPPLPCACAHETGGEFVYTPEGLLKDDF
LSKRMDTEKQEYIYCQDCPSERNKNEAMPERCKGHWVRKFIKECWRKCGCDMRCGNRVVQRGIACKLQVFHTKGKGWGVRTLEVLEKGSFVCEYVGEILT
NNELYYRNMKSKGNGRHTYPVTLDGDWGSEQGLKDEDALCLDATLHGNVARFINHRCFDGNLVDIPVQVETPDRHYYHVAFFTTRKVDAFEELTWDYGLD
FEEVHLIEAFQCICGSQYCRGKNPKGKKVEKLG

> FvSET44
MACQAMKEFGIAEVQTKPAVKQLLQVYDNNWDFIEADNYKELLNFIVPDTNKPDEEQVQPKVQGPLMRESKRVKSKN
KQAPYDTTEVIERPTKRYCTRQQKDQSVANTKYSESSLKRYVTFEEESKESEDSQPLVIKPRQRHINLQEDSESSLKRFVNAGEDVMKYLQVGLLNESNS
VSVPDRSSPVKNALISSSSQNSGFDLPNGEVSQEMMDGKCIKSYRTGECLSMAIATNVMGNKPELITCNEQDQGSKSSGLGSFTELFVDENLINPIRTPS
HIPLGCFEGLRSIVGIKVRDMENICEESAKILRVFEGQNPSCKEGASECYGDCLTSSVPCLCVIANGGECAYTPGGVVKEKFLEECISKKHEPKEHPQFY
CKKCPLEKLENGKSSVACKGHLLRRFIKECWCKCGCMRSCGNRIVQKGIAVKLQVFWTTEGKGWGLRTLEALPRGAFVCEYVGEVLTIGELHERNLPSVG
KEHSYPVLLDADWDSQDMLKDEKALCLDTTVYGNVARFINHRCSDATLVEIPVEVETPDRHYYHVALFTTRDVGAMEELTWDYGIAFNDHDYHVNPFQCV
CGSPFCRDRNDSKREKTYVRRRRKDGKDNQKDGVLGHLAKDLDEATPREEIQVEPDVVMQLEVVPSTGALREEVQVAAPHASRRLEIVPRALNKEIQDVP
NQCMQLQVIPSTPRIEPREAMHCLEREPVRDSSSSSTALLAKEVADLQAQLEAEKAAREKDHINFEAHRAQFQAVMQQLSTLLPGFQSFTSLSVMRISAA
VLPEDTWVDKTPWFLLKRQQSGAGPGAPPPQRTPAASPITFSPRRVRQIPVFRRDHPKLQIKSIYTETGIISPETGIQRTRRGGKVFRDAAGVRWGRGAP
SPAPDGGERSSVRTCARALHIRPHISD

> FvSET22
MKPRDRKTIDACSAMKTFGIKPAETKKALKGLLKLYDNNWELIEDENYKVLFDVLMPEEEGMTSQQKVPAHVKAEAF
ELPIKRLFTGQEEKHASSLSKSPLDSNPSRRAHQNDGDSGPSQPLLRRFRLRCPEKAFIRESKELKSKKNDAPPDNNTEVIELSTKRLYTRQQKKQALSL
SKSCMSDDDLFSDSEEAQPLLKRSRLSGQEGEAMVCSSKGAIYNNIESPSIGKTEVVELPTNTYTRQQKKQASSLPESCVETSLLTMAHQNDGFMFYDSE
ESEEIPLLSKSNQGRHQKKDVLVPGNKLVSGIPPPTKSPIVICLDSSSDSDVCTVDEEILEAQIVEALDVEPLTVDNIACLSDMSEMDDDEDSLEVLKAE
PLIVDSRGCVFDSSRVDIASSLSKGEVDSSLTRNSFPQSDIHFPIMDVVEERGNKSYRDRECLLAIGTDSITDENARFREVPQLEKSKSQDVLAMKDDYQ
GSNCSAPGFPSEVFVFQNLIKIVPHIPRHIDLCRFGCLHSLIGFPIKDIEKVFGTSGKRLQVLPGLKSSKLYKVQTAQSHQNSQVVKFCVYVDDIARGEE
KLKIPLEGGRNAEDLSKFFYIPQNVVYKDAHVNFALRCTSNQACCSHCFGNCLASPEPCACAMQSGGGFAYTPNGTLKITFLEECLSVIHERKQHHYFCC
KMCPLANFRKKKSSNACKGHLLQKFVKECWSKCGCNKGCGNRIIQQGISVKLQVFLTPEGKGWGLRTLEDLPRGAFVCEYVGEIVTSTELFERNMQSAGR
KHTYTVLLDANWSSKGVLKDDDEALFLDATVYGNVARCSDATLVSIPVEVETPDHHYYHVALFTTRNVAAMEELTWDYGIGFDDLDHPVKPFHCLCGSQF
CCGSNHVEEHVCNTEVLEDPIDKNFKMDLSNVKDLSRTEHGTDEELRPSPKPPPPSSSPLTTKTTASKMNLEVSRFEPQHATMSKRGRGGSAGNKFRMSL
GLPVAATVNCADNTGAKNLYIISVKGIKGRLNRLPSACVGDMVMATVKKGKPDLRKKVLPAVIVRQRKPWRRKDGVFMYFEDNAGVIVNPKGEMKGSAIT
GPIGKECADLWPRIASAANAIV

> FvSET31
MEVLPCSNVQCVGQSDCPQQNSGTTPVNGESNCLEHEKQVQVIDRTVEGLLPNVEGPQLGSQGEVKGAVHELHTSEG
CPVGALSLDCQLESQKSSSGSHGSESFDNDDVNAHNYSAEPSLVSDNGGFKLDSSENGLPYNSREGESSHSDSTWLECHESVPLWVKWRGNWQAGIRCAR
ADWPLSTLRAKPTHGRKKYFVIYFPHTRNYSWADMLLVRSIDEIPQPIAYKTHNAGLRMVEDLSVARRFIMQKLAVGMLNIVDQFHTEALIETARNVVVW
KEFAMEASRCNGYSDLGKMLLKLQSRCQNACSAETVELLKEELVESILWNEVQSLRNAALQPTLGSEWRTWKHEVMKWFSTSHPISNSGDFPQHSSDAPV
TPSLQVSRKRPKLEVRRAEAHVSQVESRGSEEAIAIEIDSEFFNNREAVNAATLASEPDKEVNMKDVAALTGDSGVADKWDDVVVATGNSVFIQSKDVEL
TPVNVVSGVKSSVSGAKSRQCIAYIEAKGRQCVRWANDGDVYCCVHLSSRFTGSSTKSEGSHSMDTPMCEGTTVLGTKCKHRSLHGSSFCKKHRPKNEPE
TITNTPENGLKRKYEENMSSLDTMNCREMVLVGDVGAPLEVDPVRIMAGDGFNGRESLSEKSELSAKTSSVTEDMRCIGSGSQDSSNPCLESPKKHSIYC
EKHLPSWLKRARNGKSRIISKEVFVDLLKDCHSHEHKLHIHRACELFYKLFKSILSLRNPVPKDVQFQWALSEASKNLVVGEIFTKLVCSEKERLVRLWG
FTTDEDTREDVCVLNSAMEEPALLPWVVDDNHDDETAIKCKICSQEFMDDQALGTHWMDNHKKEAQWLFRGYACAICLDSFTNKKVLETHVQDRHRVQFV
EQCMLLQCIPCGSHFGNNEELWSHVLVVHPDDFRPSKAVQHTLSADDGSPRKFELCNSASVENTSQNVANVRKFVCRFCGLKFDLLPDLGRHHQAAHMGP
SLVSSRPSKRGIRYYAYRLKSGRLSRPRMKKSLAAASYRIRNRANATLKKRIQASKSLSSGGTDVQNHSTEAVSLGRLADSHCSAVARILFSEMQKTKRR
PHNLDILSVARSACCKISLEVLLQGKYGILPHRLYLKAAKLCSEHNIKVSWHQEGFICPKGCRDFNALLPSPLIPRPIGTMGHRSQPLSDPLEEKWEVDE
SHYVVGSNYLSQRSQKAHILCDDISFGQETVPLVCVADEGFLDSLPANAGSPTHQIAGHSMPWESFTYTARPLLDQSPGLDTESLQLRCTCPHSTCYPEA
CDHVYFFDNDYDDAKDIYGKSMLGRFPYDDRGRIILEEGYLVYECNQMCSCSRTCPNRVLQNGGWGVRAGETILRGTFICEYIGEVLDENEANKRRNRYE
KDGYGYLYEIDAHINDMSRLIEGQAQFVIDSTNYGNVSRFINHSCSPNLVNYQVLVESMDSERAHIGLYANQDVSSLLSIMIFVHMMHGMGVEMVIKVSE
IIFLLEMVSKDVHAIVALPDAEADSIKPSMNAGLGIQFWCLYLSI

> FvSET20
MAMTKELRLTELQGRGRSLVAAQPLKSGQIVLRDSPILLYSALPLLTPSPPPYCDHCFKTLNPQTPISLCPSCSHHR
FCSPNCLSAALSSSHTPWVCQSLAKLRNCPSPLSAHPADRQVQARFLIAAYNLAAVSPSSFQLLLTLQGEPQDSAEAQFLHSLISTLCPPPQLFSVELTA
ALLAKDKLNAFGLMEPPSETGLRSSRAYGIYPKASFFNHDCLPNACRFDYVDEGSTEITVRMIHDVPEGREICLSYFPVNEPYSSRQRTLAEDYGFVCQC
DRCKVEANWSDNEEVAEEKGGSAEGMDEDQDQEMGGDLEAEGGSGSDIEAEAQGEADFPHAYFFVRFMCSRTNCWGTLAPLPPKDDGTSSDAMECNACGS
LKQDEEISGHGQNGISLDG

> FvSET3
MADASKDELLQLIKRFGTYLTVKMSSLFSISSQNLNTRSIGAIAGFAVAIVFTWRLFRTPAGPQRRQPKRQAPASSS
SGVNSPSNSTLTATGVSLSEDTRAQNVVDEFFQPVKPTLGQIVRQKLSEGRKVTCRLLGVILEESTPEELQKQVTVKYSVLEVLLEITKYCDLYLMEKVL
DDETENKVLSALEDAGVFTSGGLVKDKVLFCSTENGRTSFVRQLEPDWHIDTNPEIIFQLSRFIKYQLHISPTRSERAASNVFNSPSLEQFFGCSCEMES
ECPIDAELRREISALLEPPPPAQVEHYLNQLIQTRQCHAITVKHNGHLGKGVYAVSDLEGDELVLKDQMLVGHQHSSNKIECLVCSFCFRFVGSVEQQIG
RRLYLQELGVSSDCCPQSDEEEEEELGQCGSSSSAYKDKVPLPKGLAEALMNGELKLPYSDKFSMPQAVSCPGGCGETYYCSKLCAESDWNSSHCLLCTG
ERSEALSRESLVKFIQHANETNDIFLLAAKAVASTILNYRKLKLTCSEGDNKPNVSGSPYLSLLVEAWKPISVGHKRRWWDCVALPIDVESSDEAAFRMQ
IRELAFTSLQLLKAAIYDEECKPIFSLEIYGHIIGMFELNNLDLVVASPVEDYFLYIDDLPDPEKKEAEEITRPFLDALGDDYSVCCQGTAFYPLQSCMN
HSCTPNAKAFKREEDRDGQATIIALKAISKGEEVTISYVDEDIPFEERQALLADYGFRCRCPRCLEEQ

> FvSET36
MARVAVRCSRWAPRFLPSISKNEPFLSSTFSTAPENANPGRPGPPPIRVALTESSGRGVFATRKIETGELLHTAKPI
LTHPSLSSLHTVCYFCLRKLRTADASQPQSVSYCSQECQQQAQGFHDIEMTADWSAYDNYCRSNGLKYPLLVKRLACMVMSGAAHANLLVILQPASLTPE
MISEMEEGFGLLRNAFINSNIMGEQMSFLTKQWYIGILARIRINAFRIELAGGYNDLLSSLAASIEAEAAVGNAVYMLPSFYNHDCDPNAHIIWIENSDA
RLKALRDVDEGEELRICYIDASMDHDARQSFLSQGFGFRCNCPRCLSGD

> FvSET9
MEMEMEMRAGEEIELGRDLTPPLSPLYSALHDSLLSSHCSSCFSPLPTPPSPNNSHPVLLFCSSLCSSSASVSTAEP
RLLRLLHSHPSTYPHGDSSDLRAALRLLHSLPASSPAPRISGLLTNRRKLDDDLRIRDGARAMFLARTMPDDNDAVLDVAHDDAVSEEAALCLVLTNAVE
VQDHTGRTLGIAVYDSCFSWINHSCSPNACYRFLLSSPSQPTPPQCDETPLRIVPAGQIESGVYSNNLLMKECEKFGPRVIVRSIKRINRGEEVTITYTD
LLQPKAVRRSELWSRYRFMCSCKRCSASPLTYVDRALEDISAVNYNSSRFSSDISFDRDKATERLTDYIDDAIADYLSIGNPESCCERLEQVLTEGLSDK
QPEGNEEKSELTYWLNPLHHLSLNAYTTLASAYKILADDLLTMSSEIDNHVLGAFGMSRTGAAYSLLLAGAAHHLFNSESSLVVYVANFWTSAGDSLLNL
AKSSIWSEIVRWDLPVSDNLELYHIAKYKCPRCSLIDKLETYSLHDPVTHSDFGHASREFVDCVTNLTQKVWYFLVQGCRYLGLCKNPIDFIWLDTSECS
SEGEVFTHSTGTNCGNDRSISGSEAEENTNLLRMYILKLGVHCLLYGEYLARTCYGVHTRPVTPISERACAVVHSDISSQIEHLSE

> FvSET28

MRDTQQQLEMAEELMQQLRSKATELLLREEWSDSIQVYSQFIDLCQHQTPITPKLHKSLCLALSNRAEARSRLRDYANALKDCDEALKIESAHFKTLVCKGKILLNLNRYSMALSCFRAAQLDPQANGSSVGLNEYLQKCKKLELMSKTGVYDLSEWVVSGFRAKPPEPAEYIGAVEIRRSGIRGRGLFATKNIEAGSLVLISKAVATERGILPGHDLDENAQLVMWKTFTEKVLDSVAKCSRTCGLISTLSSGEDEDELEVPEINVFRPVSEEFGNSSEKKVDVSRILSILDVNSIVEDSISSKVLGKNSDYYGVGLWMLASFINHSCVPNARRLHVGDYVVVHASRDVKAGEELTFAYFDVLNPLEKRNEMSKTWGFGCSCRRCKFEAEVCLRQEGIGEIEMGFERGMDAAAAVYRLEEGMRKWKVKEKEKGYLRASFWAAYGSEKLMRSWGRRIPTMESVVDSIVEA

>FvSET10
MEKTNVIDMVSSTTPSLDDTEQAERYLMMEELQSSLGNRGLTVSKLPEKGRCLLTTKDFYPGDVIISQEPYVHVPNN
SADNSKCDACFESSHLMKCSRCQVVYYCSATCQKSEWKLHRLECEALSKVPKEKRRAVTPSLRLMIRLYCRSKLQSQKTIPTSAMDNYGLVEALVAHMSE
VDEKQMVLYAQMANLVNFILERPDINIKEIAENFSKFACNAHTICDSELKPLGTGLYPVISIINHSCLPNSVLLFEGKTAVVRAVQHIPKGAEVLISYID
TAGSTATRQKALKEQYLFTCACPLCIKAGHYEDIKESAILEGYRCNDNKCDGFLLRDSDDNGFICQKCGCLKRKENIIEMESEIRSLQEKGVRAVESAPT
ITYHEVIAILKAMETLQRYLCHNFCVYLLPTWEELIKNLMKVEEWSEALAYCRLTIPVYQMSSVECLNKLIACLRLLGETDDALKSLTKAVDILRITHGT
STPFMKDLFRRLEEARAEAFINGVD

> FvSET12
MYRQAQIYEEECRAYATILPENSTPTHHRLIHLCRLDDRKTATYGHRCYETFDLANHHAVCALLDDRATPHAFHPTN
IGLHRENFPPIWITLSAPHKTPLIRLLIHRHRVHVVLFDDDAVMARQKPTPSVTSPLFGIWKTALNGFHGGILGNRGLAISKLPGKGRCLFTTKDFNPGE
VIITEEPYVCMPKDSVANYLCDACFEESGNLNKCSRCKTVYYCSTACQKSEWKLHRLECEALSKVPLEFHHQITPRIRLMIRLQCRLKLQYENTIPTSAM
DNHNLVNALVSHMSKIDAHRRWHYAMDANLVSMVLQWPDINKQEVAVNFSKMAFNAFTIFDSELQHLGEGLYPVISFMNHR

> FvSET19
MASSLPLHHPSHCLISNPQGFNYGGSPRPRFYFNTKDTDKGIRIQPIKAKAATEASPFPLLQSPQAEDSASELEPAD
PDFYRIGFVRSMRAYGIEFKEGPNGFGVYASKDVEPLRRARVIMEIPLELMLTISQKLPWMFFPDIVPVGHPIFDIINSTDPETGWDLRLACLLLYAFDR
EDNFWQLYGDFLPSADECPSLLLASEEELSELQDPNLASTMREQHRRALEFWEKNWHSGVPLKIKRLARDSERFIWALSMAQSRCIGMQIRIGALVQDSN
MLIPYADMLNHSFQPNCFLHWRFKDRMLEVMINAGQRIKKGDEMTINYMSGQQNHMLMQRYGFSSSVNPWDVIQFSGNAQIHLDSFLSVFNISGLPQEYY
HNTDRLSSGGDSFVDGAVIAAARTLPTWSDGDVPPIPSMERRAIKELQEECRQMLAAFPTNSTEDQNILGKLATQILISGSCFLDIGNSCFAPSISPDSI
PDATRTLEAVIKYRLHRKLYIKKVMQALDFQQLEDGCTLADYNIQKESTLHLLGRQLLQSLLTMSYFLVRMQGLQVYIETDDALKLVQLSHKIVASMAGD
SGQCKCLLNYVYEQIKDKEAADVHEDGRKNGRLGLGYQICFRLLP

> FvSET21

MAASKMVMASLTAIRPFSCAAAASASASYPGRLAPHTPDLIKWVRREGGFVHEAVKIAQDTSFGLGLVATDEIPKGSELVVLPEHVPLRFVPLESDSGDGADAVLANLARQVPDELWAMKLGLKLLQERARVGSFWWPYIGNLPEAFSVPIFFPGEDIKNLQYAPVLYQVNKRCRFLLEFEQEVRRALDNNKPSDHPFGGQAVGASALGWAMSAVSSRAFRLYGKKLADGNYDDVPMMLPLIDMCNHSFKPNARILQDQDDRNKKMLVKVVAETEIKQNDCLELNYGGLSNDLFLLDYGFVVPSNPYDCIELKYDGAFLDAASMAAGESSPSFSAPAPWQKEILCQLKLDGEAPLLKVCLGGSEVVEGRLLAALRVVLASDVETVQKHDLNTLKSISVEAPLGIANEVAAFRTVIALCVIALGHFPTKLMEDESILQKGVSDSTELAIQFRILKKSVIIDVMRDLTRRVKLLSSKETATAQG

>CsJmjC1 orange1.1g000392m
MGNNSNNNVEIPKWLQGLPLAPVFYPTDTEFADPIAYISRIEKEASAFGICKIVPPLPKPSKKYVFGNLNKSLSKCSELGSDV
NLPDAGTVATVGCCERGNEGEARAVFTTRHQELGQSVKRIKGVDNKDNLQLGAQKQVWQSGEVYTLEQFESKSKAFARSLLSVIKEVSPLVIEALFWKAA
SEKPVYVEYANDVPGSGFGEPEGQFRYFHRRRRKVTSWKSYRNRGKADEKNIELESARNCHNDQITHSCDKNDLETPTSSTPSSTLPFDENSRSSRRKSV
TGSNDVEGTAGWKLSNSPWNLQVISRSPGSLTRFMPDDIPGVTSPMVYIGMLFSWFAWHVEDHELHSMNFLHTGAPKTWYAIPGDYAFTFEEVIRNEAYG
GDIDRLAALSLLGEKTTLISPEVIAASGIPCCRLVQNPGEFVVTFPRAYHAGFSHGFNCGEAANFGTPQWLMVAKEAAVRRAAMNYLPMLSHQQLLYLLT
MSFISRVPRSLLPGARSSRLRDRQKEERELLVKKAFVEDILKENNILSVLLGRQSTFNAVLWNADLLPCQSKESQMPSANETVSTTPGETVPNNPYEKHN
DHNNLLDEMNVYMEALNDPYMGDDDISRDFHIDSGALACVACGILGFPFMSVVQLSERASIELLADLVKEGPGVSELKNTHHHTNLDGSVKSSVSDDLCL
VPDISLLQKDLSVPSITKSSRIWNTSNKYLRPRIFCLEHAAQIEEILQSKGGAEILVICHSDYQKIKAHAAAVAEEIGSPFNYIDVPLDAASEEDLHLID
LAIDDGELDECREDWTSKLGINLRHCVKVRKNSPSMRVQHALSLGDLFSEKSLSSDFSKIKWQFRRSRSKIKLYGRAHSKPCQNIEIKKDEVTGRKLDGA
TVKKEEKLIQYSRRKFKQKPDLSTGACGDQVHPRELLPEVSAATCDHLDGHNRSDFEINPDGTGNSGSISAGSIHSPIGMSEGLHDIPVREATSNLSLNY
SPSRVADSLATATLVVDSIVQNDTESMKELNIEGDIFHMATCKSAEMQQNSGTDVTSEKTEISHHTVASNEGSIIMRSDQITESMTIKNEKCNLASEGHC
RKIEDLAPDNSCMISEACDHMISDNEVRQNVQSTNGGNDVEPISCDHKLIDEPPASTGESCEDMREISTAESLQDNLQHERNIGNGSNEELVSSSVTMMI
QPTSAPMEISEVPSKECAAADLLNVGTKQKLISSCVSRMEVDQPSPLKVGGCSEVPIEICTKEDSGADMTLDPRTQLQNHTTAEAIMDELVCNSSAQLEE
NERIPASVAACSEESNGIFAEEKMDFDMTIGTQTKNATSEEPKPTSLIPIDQPIPAVIRKYSRTRRESYSAEKFCNGNEAYSSKDNKERECNESNLEDPS
FSAGKGRKRNRELERLTENKFNGSGFIRSPCEGLRSRAGKDAANTSEVDIRKIAEKRATKTMRNRESVPAPCQDKKKILKGHHRCDLDGCRMSFETKREL
SLHKRNRCPHEGCGKRFSSHKYAIIHQRVHDDERPLKCPWKGCSMSFKWAWARTEHIRVHTGERPYKCKFEGCGLSFRFVSDISRHRRKTGHYENLSA

> CsJmjC2 orange1.1g042974m
MKSETVNETIGISNMMSRSGGDALRASASCGIRLHGNADSFSRPNTAPTGKVVFSKRKVDKFDTNDLDWTEKIPECPVFRPTK
EEFADPLVYLQKIAPEASSYGICKIVSPVSASVPAGVVLTKEKAGFKFTTRVQPLRLAEWDADDKVTFFMSGRNYTFRDFEKMANKVFARRYCSAGCLPA
SYMEKEFWNEIACGKTETVEYACDVDGSAFSSSSGDPLGNSKWNLKNLSRLPKSVLRLLDTVIPGITDPMLYIGMLFSMFAWHVEDHYLYSINYHHCGAS
KTWYGIPGQAALKFEKVVREHVYTRDILSTDGEDGAFDPGEFIITFPRAYHAGFSHGFNCGEAVNFAIGDWFPLGAVASWRYAHLNRIPLLPHEELLCKE
AMLLYTSLVLEDLEYSSADLVSHRCIKVSFVNLMRFQHRARWLVMKSRACTGISPNYHGTVVCSICKRDCYIAYLNCNCYLHPVCLRHDIESLDFSCGST
YTLFLRDDIAEMEAAAKKFEQEEGILKEVQQKAESDDLYSYPFSKMFHSVREDGYSPYCEINMELNHKPAAKTWNRSGKSEYSCHIQPILNQEAANFRSE
HAETSVSDAASTICSFVKPIESSSTANNDVRWQSKFNLGILAVKNSPEEVSRTTYESSQTRNECPSANGSNFHRSEVGAVMNQYSDDSDSEIFRVKRRPS
KVDKRCMNDVTSSTHTEHQGLKRLKKLQPEGRCGQLMLTEFRRTDESNHKSSHTSNYKETSERGSKDRFARVGGAVPISIKFKKLADEEAISRQQENCRK
ERFQHEFGKATREPPPIEMGPKRLKVRGPSFIGSDRSD

> CsJmjC3 orange1.1g043353m
MARPRTARNRSPVSKQESYKFKFSDVDWTHKISDCPAYYPTLQEFDDPFIYLQKIAPEASQFGICKIISPVKASVSAADVLKK
EIKGFEFGTYRQPLRLPKWNANDTGVFFSGERKHTYDTFESEAIKMLKRQSPRLGDLPPSYVEKKFWLEMTHGRKGTVEYGVNIEGSAFSSDPNDQLGKC
KWHLKTLRGLPQSIFRFLEHIIPGITDPMLYIGMLFSMFAWHVEDHYLHSINYHHSGAPKTWYGVPGHHALQFEKVARNQVYSRDILSAAGEDGAFEVIA
EKTTIFPPKILLDNGVSVYKAVQKPGEFVITFPRVYHAGFSNGFNCGEAVNFAIRDWFPFGEEAGKRYARLHKMVILPYQELLFKEVSEHEGTDIPSSVK
ATVLHQIRSLNNALFCLNNLKMPFDYLQNSQGSFVCDLCKRDCYLAFTECKSCQRYTCLFHEFKSRHCSCGYNRVVLLRKDIQEVEVVAKKFEEEEIMSR
HLNIISCIGEGLARNS

> CsJmjC4 orange1.1g002177m
MDHQRSSLGNGEDNGGIPDDLRCKRSDGKQWRCTAMSMPDKTVCEKHYIQAKRRAANSALRASLKKAKRKSLGESDIYLESKS
DDYDMPLVNMKNNDYPSVSGKKTLEKVSKSHFRYSPETPPTRGMSARNPLKANDDSQRDVAEYEENLRSYKTPPHSGMDSSRNRSQRSFDPSPTMEYSEG
SMNSSEDTGGQICHQCRRNDRERVVWCVKCDKRGYCDSCISTWYSDIPLEELEKVCPACRGSCNCKACLRADNMIKVRIREIPVLDKLQHLYCLLSAVLP
VVKQIHQIQCSEVELEKKLRGNEIDLARAKLSADEQMCCNICRIPIIDYHRHCGNCMYDLCLSCCQDLREASTSVGKEEFSENDRIQDTENASEQVKTSK
LRLNLLEKFPGWKANNDGSIPCPPNEYGGCGYRSLNLSRIFKMNWVAKLVKNVEEMVSGCKVCDSETLLNTGSYDHSLCQYAHREDRDGNFLYCPSSHDI
RSEGIGNFRKHWVKGEPVIVKQVCDSSSMSIWDPKDIWRGIRETADEKTKDENRIVKAIDCLDWSEVDIELGEFIKGYSEGRVREDGWPEMLKLKDWPSP
SASEEFLLYHKPEFISKLPLLEYIHSRLGFLNVAAKLPHYSLQNDVGPKIYMSYGTYEELDRGNSVKNLHFNMPDMVYLLVHMGEVKLPTTEDEKIQSSS
RESEVNESVGDPEKVSGEGSFPDLSLGGHDVNNEHVEKSATDEDEIMEDQGVETGTAEEKTVKSERLNGYSDVSEKTHPGAHWDVFRRQDVPKLIEYLRE
HWTDFGRPDGVTNDFVTHPLYGEVVYLNGDHKRKLKEEFGVEPWSFEQHLGEAVFIPAGCPFQVRNLQSTVQLGLDFLFPESVGEAVRLAEEIRCLPNDH
EAKLQVLEVRQRKLFQEVGKISLYAASSAIKEVQKLVLDPKLGAELGFEDPNLTATVSENLENLMKHKQITCA

> CsJmjC5 orange1.1g001595m
MEQSKLAAESHIKEISARWDPAEACRPIIDEAPVFYPTVEEFEDTLGYIAKIRSKAESFGICRIVPPSSWTPPCPLKAKNIWE
NAKFSTRIQQIDLLQNREPMRKKIRSRKRKRRRQSRMGSTRRNANSSSEANAAETDEKFGFQSGPDLTLEGFQKYAQNFKECYFGMNDSKEDVKSDGFEH
KRLEPSVVDIEGEYWRIIERPTDEVEVYYGADLETGAFASGFPKASSLGTESDLDQYAMSGWNLNNLPRLPGSVLAFEGSDISGVLVPWLYVGMCFSSFC
WHVEDHHLYSLNYLHWGDPKIWYGVPGSHASTLEKAMRKHLPDLFEEQPDLLHELVTQLSPSVLKAEGVPVYHVVQHSGEFVLTFPRAYHSGFNCGFNCA
EAVNVAPVDWLAHGQQAVELYSEQHRKTSLSHDKLLFGSVQAAIKALWELSVLQKKTPGNRKWKDACGKDGVLTKAIKTRVQMKKEGLQKLPSYFKLQKM
EIDFDLKTERECFSCFYDLHLSAAGCKCSPDRFACLKHANIFCSCEIDHRFVILRYSTDELNTLVEALEGGLDALKELASKNFKWADCSDTDGGLVKMDM
ESEVFPMDCCEQKESSSSSPRVENIVEGNGPCCSRSHVSSEVVQSEPQRGTSGLSASHVSVNSHNEGNDETQVMNKKAKVKHEVCIDLNMDVIPDGNESK
LLLSDSHGKEAIENLKAHLSACYQEKVLCSGTVKEQDTMQVRSDCNSSNSHKDPNKDQPSCSRVIEGTCSFDVKKLFGVDLSLPHQQSKLPLVDFLKTDT
INGSNVRTSVTDQRFQKKLETCVEPINFGCVMCGKLWCSKQAIFPKGFRSRVNFYSVLNPEKVCNYISEVLDAGLLGPLFKVTLEECPSETFVNVSAQKC
WEMVLQRLNQEIERQGGLHERGLPHPQSLQSIDGLEMFGFLSSPIIQAIEALDPNHLCMEYWNHKLLTFGKTTEVNKNSSSGLSCSEAETKSKIFGVALM
DEDQNSPSGQNSVEEEAQLVLRGLFQKASPKELKVMQRILYSEGRSDEWRVALATLIEEIQKSCR

> CsJmjC6 orange1.1g001517m
MGSKKKNKILTSEENRRMPGNIVCDNGSGHKNVLPVSLERTSMAKGEEKQQVGDVQKNDCGNKKPCKRGRAKDGKNKRAVFYG
KALNRILAKKHQNQRPPTKIGEEKGKYMKVKRGCLVEEGSDIGHGDINTCKLSNDSVKIEKRTRGRPRKICNQSENSESIDATSCKKEQRGLMCHQCLRN
DRSDVVVCANCKRKRYCYQCVAKWYPEKTREDIEIACPFCRGNCNCRVCLKQDLDVLAGHQEEDKNIKLEKLLYLLQKTLPLLRHIQQEQNSELEVESKI
CGIQLTEDHVKRSVLDDDDRVYCDNCSTSIVNFHRSCPNPDCSYDLCLTCCWEIRKDIQSGDKEAKSSQQQVFEKVRGQVAELNGQNSVNFGTDDCVADM
SCKFLDWRAEPHGRIPCPAKARGGCGTQMLTLRRIFDANWVSKLITTAEDLTFSYRSLDVNVSQGCSLCHPVDSAENGTKPLEVRQAAYRENSQDNYLYC
PNAIQLGNSAIEHFQMHWIRGEPVIVRNVLETTCGLSWDPMVMWRAFVGARRILKEEAHKVKAIDCLEWCEVEINIFQFFKGYLEGRRYRNGWPGMLKLK
DWPPSNSFEECLPRHGAEFIAMLPFADYTHPKSGLLNLATKLPAVLKPDLGPKAYIAYGSSEELGRGDSVTKLHCDISDAVNVLTHTAEVKIPPWQQKII
KNLQKKYDAEDLDKLSSRVPNASGRVGRKPQKKPPKEKNPKVNTTGSDSLMEHFNLEEKKQDGLQNTSQEGEYSKGLDALWLTPKRRESALGQSDFHGPK
PDQGERDAASESLPDNRIQSYNNCLDDARANPSFPNGMDTGHSCAAVEEFQPAHALEGNHETVEGSVCNQDHPYDVAGKTELVKGEGSLEATYSDDGVDN
EASIESDVNAERDNFLDNHMTDVVYGGAVWDIFRRQDVPKLIEYLQKHQKEFRHINNLPVTSVIHPIHDQTLFLSERHKKQLKEEFNVEPWTFEQHLGEA
VFIPAGCPHQVRNRKSCIKVALDFVSPENVQECIQLTEEFRLLPKGHRANEDKLEVKKMALYAVSAAVSEAQILTSKSE

> CsJmjC7 orange1.1g039227m
MSEEAIAKSCPFCCRNCNCNACLHNSEIVKVTRDLGAPAKTVENIGHFKYLLRLLYPFLRKFHHDQVKEKKIEAKIKGLELSE
IEVPQVVLRSNERLFCNNFDYYRSCPRCSYDLCLTCCREIRDGCLQGGVNMYTSHFDRGKAYLHGGESLPLPSGKKSGIRFSSKKRMRKISQWKARENGD
IPCPVNKLGGCGHEYLELKCIFANGWLSELKVKAKKLVKVHNLVDRPHHSGQSCSCFKLNGQIDCCSKSLRKAASREGVSDNYLYCPSATDVQHESLEHF
KSHWIKGEPVIITNVLDYSSGLSWEPMVMSRAVRDTSYSKGSQKLVVKTVDCLDLCEVKINTYQFFKAYMEGRTHSNSWPVILKLKDWPPSCLFEERLPC
HGAEFMNILPYKDYTHPYSGILNIATKLPPDFLKPDLGPKAYIAYGVAEELGRGDSVTKLHCDMSDAVNVLMHTAKVDYSSKQVAEIEKLKRKHAIQDRR
EFFNPLYARDETFDMNHSKSEEKLRPISSMQSNTLSLNGKDGGALWDIFRREDVPKLGEYLRNHHKEFRHVYCSPVEQVVHPIHDQTFYLNMYHKKKLKE
EFGVEPWSFVQQLGEAVLIPAGCPHQVRNLMSCTKIALDFVSPENINECIRLTDEFRTLPRNHRAKKDKLQ

> CsJmjC8 orange1.1g000212m
MGKGRTSAVLGQKLSVASTSKSASLSVPSGPVYYPTEDEFKDPLEYICKIRAEAERYGICKIVPPKSWKPPFALDLGSFTFPT
KTQAIHQLQARSAACDSKTFELEYSRFLKEHVGTKLNKKVFFEGEELDLCKLFNAAKRFGGYDKVVKEKKWGEVFRFVRSNRKISDCARHVLCQLYYKHL
YDYEKYYNKLNKEVTKGCKRGLDGDVKSEDKVERSSSKRRRRNNCDQERVKVCHKVDKEDELDQICEQCKSGLHGEVMLLCDRCNKGWHVYCLSPPLKHV
PRGNWYCLECLNSDKDSFGFVPGKRYTVESFRRVADRAKKKRFRSGSASRVQMEKKFWEIVEGAAGNVEVMYGSDLDTSIYGSGFPRVCDHRPESVDANV
WNEYCNSPWNLNNLPKLKGSILRMVHHNITGVMVPWLYLGMLFSAFCWHFEDHCFYSMNYHHWGDPKCWYSVPGSEAGAFEKVMRSSLPDLFDAQPDLLF
QLVTMLNPSVLVENGVPVYSVLQEPGNFVITFPRSYHAGFNFGLNCAEAVNFAPADWLPHGGFGADLYQQYHKAAVLSHEELLCVVAKVSDLDSKVSPYL
KRELLRVYTKERMWRERLWRKGIIKSTPMGPRKCPEYVGTEEDPTCIICRQYLYLSAVACRCRPAAFVCLEHWEHLCECKTRKLHLLYRHTLAELYDLFL
TVDRNSSEETSESNNLRRQISSSNRPTTLTKKVKGVRVTMSQLVEQWLSCSLKVLQGLFSSDAYGTLLREAEQFLWAGFEMDAVRDMVNKLIEGRRWAEG
IRDCLHKAENWSSLPGSDSEKVRLDCVNELLGFDPLPCNEPGHLILQNYAEEARSLIQEINAALSACSKISELELLYSRASGLPICIVESEKLSQRISSA
KVWRDSVRKCISNKCPAAIEIDVLYKLESEALDLKIDVPETDMLLKMIGQAESCRARCSEALRGSMSLKTVELLLQELGDFTVNMPELELLKQYHSDAIF
WIARLNDILVNINGRKDQHNVIDELNCILKEGASLRIQVDDLPLVEVELKKAHCREKALKACDTKMPLDFIRQVTAEAVILQIEREKLFIDLSGVLAAAM
RWEERAADILIHKAQMCEFEDIIRASQDIFVVLPSLDEVQNEISTAKSWLKNSELFLASAFAVAPASCSLLRLESLKDLVSQSKFLKISLKEQTELEKVI
NNCERWQNHASSLLQDARCLLDKDDIGDGLSNSLVSKIEQLITSMESAANCGLSLGFDFHEISELQNACSTLHWCKKALSFLSVSPSLEDVESLMAVAEG
LSTRCFSSMLWNSLIHGVKWLKRALEVISAPCKFKRCKLSDVEEVLAGCKGINFSFPVVIGELTSAIQKHKLWQEQVHQFFNLKCAQQSWSLMLQLKELG
EAAAFDCPELEKVLSKVDKVENWKQRCKEIVGTSVGDKNSLLGLLQKIKQSVHRSLYIYNKPHGSVSMTLCMCCESDSKELEFLICSACKDCYHLQCLRP
TEVDRNHAEAYICPYCQYFESESVSQFGGSPLRFGGKRSDLRMLIELLSDSEFFCRGIEAKDVLQEVVDVALECKTCLTDIVKFESCYLDKDLHVISNKL
TITLKAREAAGVFDRQSNSALDFALARNLWRVRVSKLLEGLTKPTIGQIQNYLKEGLLMNISPKDHYRQKLMELNRIGSQWADVAKKVVLDSGALSLDKV
FELIAEGENLPVYLEKELKSLRARSMLYCICRKPYDEKAMIACYQCDEWYHIDCVKLLSAPEIYICAACKPQAEESSTPQNVDGGRTNAEFLEPKTPSPK
HTNSRKKLRKAEPGLAQKMLAIANNSSVFDCSSGIDNLWWHNRKPFRRAAKKRTVLDSLCPFIYTQQ

> CsJmjC9 orange1.1g039459m
MGTKRMRANLGNEDLDKLSAPPGFMSLTSFLLKKVENSEESCNSVAFASASAQEPVCANAPSDMVDAGTLKRSLRNRPWILYE
QSDNNQKESNFEQPVEELSSRATLPKGVIRGCPDCSNCLKVTARWSPEGAKNDVLEEAPVFYPTEEEFSDTLKYIASVRLKSEEYGICRIVPPPSWKPPC
LVKENDIWKSSKFVTQIQQIDGLQNQYFSSKAAKIYDNVNSNSKRRRSLNTGLQNGVGGNGCTMNLDEARCTEGFESERGPEFTLETFKKYADDFKEQYF
CTKNIDMTVDENPLVFKKQGEPSLENIEGEYRRIIENPTEEIEVLYGENLETGTFGSGFPTVSNPCEASDHQKYLKSGWNLNNLPMLPGSLLSSESCKTC
NLLVPRLHVGMCFTSIYWKVEEHCLCSLYYMHLGAPKIWHSIPQRYAVKFDAAAKKYLPTLSFKQSKWHNRWVASLSPSPLKSEGVPVYRCTQSPGEFVL
VFSGSYYSGFDCGFNCSESVNFAPIEWLPHGQNAIELYREQGRKTSISHDKLLLGAAREVVKTQWEISLVKKHTSDNFMWRHVSGKDGILAKALKSRINS
ESNRRKYLCSSSQSQRMDKNFDYTSKRECNICLYDLHLSAAFCPCSPDIYSCLNHVKQLCSCAWTEKIFLFRYEISELNVLLEAVEGKLSAVYRWAKDDL
KMYLHSYSSRDGLRPNSQAEESKQTEYKPLDSAKFNGVGSDSFS

> CsJmjC10 orange1.1g039624m
MAEPVQQQDILPWLKTLPVAPEFHPTLAEFQDPIAYIFKIEKEASQYGICKIVPPVPPPPKKTAITFLNRSLAQRAAATGGAT
SSSGPTFTTRQQQIGFCPRKPRPVQKPVWQSGEYYTFQEFETKAKNFEKSYLKKCGNKKAALSALEIESLYWKASVDKPFSVEYANDMPGSAFVPVRKIR
EAVGEGVTVGETPWNMRGVSRAKGSLLRFMKEEIPGVTSPMVYIAMLFSWFAWHAEDHDLHSLNYLHMGASKTWYGVPMEAANAFEEVVRVHGYGEEINP
LVTFATLGEKTTMISPEVFVGAGVPCCRLVQNAGEFVVTFPRAYHMGFSHGFNCGEAANIATPEWLNIAKDAAIRRASINYPPMVSHFQLLYDLAIAMHS
SIPVAVSAKPRSSRLKDKNKDEGETLVKELFVQDVAQNNELLHVLGQGSPIVLLPQSSSGALGANPWIPLGLCSYREAIKSSG

> CsJmjC11 orange1.1g042108m
MGKQELPQPEELEAIEEETEEEGGGGGYNLKSSAPSNTHGVQPLGNLYFNPGSVNSRNTGLGNLQTLTDELVIDVLGFLDATQ
LGVLATVSKSFYVFANHEPLWRNLALDNLKGEFMFNGSWKSTFVSACYPSFDVGKVNVDGCLRVRDFYSDYLFQSWLCANLEMKPEWLERDNIARKKCIS
VEEFVSSFEEPNKPVLLEGCLDNWAALKKWDRDYLVNVCGDVRFAVGPVEMKLEEYFRYSDSVREERPLYLFDPKFADKVPTLGGEYEVPVYFREDLFSV
LGNERPDYRWVIIGPAGSGSSFHMDPNSTSAWNAIIKGSKKWILFPPDVVPPGVHPSSDGAEVACPVSIMEWFMNFYGATKNWKKRPIECICKAGEVIFV
PNGWWHLVINLEESIAITQNYVS

> CsJmjC12 orange1.1g016948m
MQEVKKLWDEVRELSLGSNSTIERLESPPTPLQFLRDYVSQNKPCIIKNVSLHHQWPAFSLWPHPSYLSKTLSSSPPVSVHLS
PNGRADSLVTLTHPRSGEISQCFASAHVERLPFDEALQLVSNSKNGDVVAYLQQQNDCFRDEYSVLGSDCDEHIAWATEALGCYPEAVNLWIGNQLSETS
FHKDHYENLYTVVSGQKHFLLLPPTDVHRMYIRQYPAAHYSYSRVNDVERFTLELEEPVRYVPWCSVNPYPSPETRESEMAKFPLYFNGPKPFECTVNAG
EILYLPSMWFHHVRQSPDDNGYTIALNYWYDMQFDIKYAYFNFLQSLHFKAPCDPTLLEIDYEDSRPNASICNSREKLFADASSLNELETTKDSEDA

> CsJmjC13 orange1.1g002333m
MAATIAKRQGLGRYSDDKMRRIRGTSDISWQRRGVSKVLDRENMKDIAQEKEKERHVLQASSGAKKPRMNQDFGFSDSTRIPK
KPRSALNRKVSYENGDEDEVLDKRTSLEVEMSEELDYDAEEIALIRIRERRRSRRLEPDGAMIKTNPHKGRQKIDSANSSSCSSSSTSSGSSDSVLKSNS
NNNGRCTARNVKEKELERIKCHQCMKSERKYVVPCGKCRTKVYCIQCIKQWYPKMSELDVAEICPFCRRNCNCSVCLHTSGFIETSKINMTDCEKVEHLR
YLMVSLLPFIRQICEEQTQEIEFEASIQRVHSSKVGVSETLCGNDERVYCNHCATSIIDLHRSCPKCSYELCLTCCKEICEGRLSGRAEMKFQYVNRGYG
YMQGGDPLPESCLHQTPDVHVEPSVMWSADDNGTISCPPTEMGGCGDCVLELTRILPDRWISDLEKEARDLVLILDNKLTNLRQNRAETGTDMLCKAASR
EGSDDNLLYCPDSTKIQEDEELFRFQKHWIKGEPVIVRNVLDKVTGLSWEPMVMWRALCENVDSEVSSKMSEVKAIDCLASCEVEISTRQFFKGYTQGRT
YDNFWPEMLKLKDWPPSDKFEDLMPRHCDEFISALPFQEYSDPRAGILNLAVKLPSGVLKPDLGPKTYIAYGVAEELGRGDSVTKLHCDMSDAVNILTHT
EEVLLTEEQHSAVERLKKEHRAQDLKENLVQDGMDESIEEPNSDNNKEDTDVSEINDSELLPSGIRGEFKMSRDEMQGTAFTCPHSEGTMVESGGALWDI
FRRQDVPKLEAYLRKHFKEFRHVYCSPVEQVIHPIHDQCFYLSSEHKKKLKEEFGVEPWTFEQKLGEAVFIPAGCPHQVRNLKSCTKVAVDFVSPENVDE
CLRLTKEFRLLPKNHRAREDKLEVYLVFIKRKCYVHEISSSFVFILLTHIFF

> CsJmjC14 orange1.1g006745m
MVLSLKKYLILSLKVFDSKPQKGSRRSASMRDFKVDSTPETDSKFDTTPGTDSKVGSNPEKDSKGREKPISDWKANENGSILC
PSIELGGCGNVLELRCTFDENWVAELLRKAEEIAKAHNLEDTPESSERVCTCYNPLGEIDMTNSELIKAASREDSTDNYLYNPAAKDIRHGDLKHFQWHW
AKGEPVIVSNVLENALGLSWDPMVMWRACRQISNTKHRLYLDVKAIDCLDWCEGEVNIHQFFKGYTDGRFDKESWPQILKLKDWPPSNLFEERLPRHNVE
FLGCLPFKEYTHPCAGALNIATKLPKKSLKPDMGPKTYIAYGVAQELGRADSVTKLHCDMSDAVNVLTHTTDVKLKPEHLAKIEKLKQQHKAQDQMEFFG
CSQFSDENSHANSSAIPVKNEQCGGKPDDGDGVGVVPQDSQICDSMLNDPIPVQRAISEEASEAIADLGKSRESGEPSNIPENEFESADGGAVWDIFRRQ
DISKLQDYLKKHFREFRHIHCCPVQQVIHPIHDQVFYLSSEHKAKLKQEYGIEPWTFIQKLGEAVFVPAGCPHQVRNLKSCIKAALDFVSPENVSQCVRL
TEEFRLLPPNHRAKEDKLEVKKMILYAVSQAVKDISDPGAANKPRILNQ

> CsJmjC15 orange1.1g044388m
MGTELMRVCIKEENDEVPSVPPGFESFASFTLKRVQDTEKHDCDITSCSASASASESLSVHMETEVKVADAAKAARPLRRRPG
INYGLLDHSSEDESDSGKLGQNFTARPCLPKGVIRGCPTCSDCQKVTARWRPEDSCRPDLEDAPVFYPTEEEFQDTLKYIASIRPKAEPYGICRIVPPSS
WKPPCPLKEKPIWDSSTFVTRVQRVDKLQNRNSMRKVSRIHNHSRRKRRRSTRMAVDCGSDSGNVSASGDVGCYEDERFGFEPGPAFTLNTFQKYADLFK
AQYFSRDKNDAKGLGANTAVLEEHWEPLVENIEGEYWRIVEKATEEIEVLYGADLETCVFGSGFPKTLNQVGSASDERYIKSGWNLNNFPRLPGSVLSYE
SGDISGVLVPWLYIGMCFSSFCWHVEDHHLYSLNYMHWGAPKMWYGVPGKDALKLEEAMRKHLHDLFEEQPDLLHKLVTQLSPSILKSEGLPVYRCVQNA
GEFVLTFPRAYHSGFNCGFNCAEAVNVAPVDWLPHGQIAIELYREQGRKTSISHDKLLLGAAREAVRAHWELNLLKKNTSDNLRWKDFCGKDGILAKALK
KRVDMERARREFLSSSSQTMKMESNFDATSERECSVCLFDLHLSAVGCHCSSDRYACLIHAKNFCSCAWGSKFFLYRYDTSELNILVEALEGKLSAVYRW
ARLDLGLALSSFISRDNMDFDKLSHSMDGPVLKM

> CsJmjC16 orange1.1g011537m
MGIRIGGGQIEKLNGKEVSYSEFVEKYMAKNQPVVLTGLMDDWRACKDWVTENGQPNLLFFSTHFGKSKVQVADCGIREFTDQ
KRVEMSVSEFVKNWLENSIMENSNASTNEANDKSVLYLKDWHFAKEYPEYVAYRTPLIFCDDWLNMYLDHFRLHKDPESYQKDNDICCSDYRFVYMGAKG
SWTPLHADVFRSYSWSANVCGKKKWLFLSPSQCHLVFDRNLKGCVYNIFDDVSETDFPGFKKTLWLECTQEQNEIIFVPSGWYHQVHNLEDTISINHNWF
NGYNLSWVWDLLLRDYNEAKEYIEDIRDICDDFEGLCQRNLAANTGMNFYDFFSFLSRFSLVNVVILFHLRRDYENQIWSSSPVARHLALNLVSIQKIAL
KMKSVNDLAGSFGFFMYLKETLDDPEFLKLCMGFCRTYGMIHEEEKWTCEIKKALMLDFEDYDSLISSPEDLVKFIDFAAGKFSGNFSEENILLSRLEDG

> CsJmjC17 orange1.1g015736m
MDELLTPIMDEESPRLLQTISEHGGYAYVGMAAQAAADIRAAEAARDLAWEQLHSGPWHSVLPVWRDAYSMACLHGAKYHYRN
GEFKEALRVLDMGVLMGGPVLRKDLDSAIETLSLKAREGENERFGEREANRLVSEEFNTAKALQVLPNRSLSCKLVVKRSALSLEGFLSEYFLSGSPVII
TDCMAHWPARTNWNDLDYLKRVAGDRTVPVEVGKNYLCQDWKQELIPFSQFLERIQSNGSSASVPTYLAQHQLFDQINELRNDICIPDYCFVGGGELRSL
NAWFGPAGTVTPLHHDPHHNILAQVVGKKYIRLYPASLSEELYPYSETMLCNSSQVDLDNIDETKFPKVRDLEFFDCILDEGEMLYIPPKWWHYVRSLSI
SFSVSFWWSDGGSSTAYS

>CsSET1 orange1.1g048157m
MGVMDSLLQTESARVVSLPNGSHSDGRLGKAPMENGHCASQGGPKHKRRKISAVRDFPPGCGPSASRINWIPNEEAIVGVLRP
DAENVVVSSNHVDMLDLVSADPNGTLLLDTENVNTSGGKMYDGSKNLNMMHIGVSDEEMVLQSGSKALSSPNSRNAVPHLSNLERILTRNYPPRRRVSAI
RDFPPFCGQNASVLGKEECMEAHPSFRSSPQEESDSKGKPLKETVKTDENQIRVNGYDGDVCMNEFGGDVSKITSGKVLADFEEHATMETKNRDGFATSS
KKMMTVAQEDTGEMSVVCPHATKRYRFDGKTGALIKSNERDVGVLEENPVRDIVVYGEHKQLDGTRSDFSVSDNQFQEEDSEGLQLALNRVIVQGLMASL
NCPWRWEKGVCKPNYVSGTGQRERKKHNSLPPSKSPSEEIIKAKGSEGSYCKRNSYSGRNAYENRSALVMRDGKDSLGHDRGQENFHLGQRSHVFDVTLP
PHPRSSSGKGPENDAIGARNKVRETLRLFQAVCRKLLHEEEAKPSRQNSHKRVDYLAARILKDKKKYIPVDKKVIGSVPGVEVGDEFQYRVELNMIGLHL
QIQGGIDYVKHKGKILATSIVASGGYDDNLDNSDVLIYTGQGGNVMNGGKEPEDQKLERGNLALANSIHEQNPRYWQDVGSHGKLVFKFKLARIPGQPEL
SWKVVKKCKKSKVREGLCVDDISQGKELIPICAVNTVDDEKPPSFKYITNIIYPDWCRPVPPKGCDCTNGCSELGKCACVAKNGGELPYNHNGAIVQAKP
LVYECGPSCKCPPSCYNRVSQQGIKFQLEIFKTEARGWGVRSLNSIPSGSFICEYAGELLEEKEAERRTSNDEYLFDIGNNYNDGSLWGGLSNVMPDAPS
SSCGVVEDGGFTIDAVEYGNVGRFVNHSCSPNLYAQNVLYDHEDKRMPHIMLFAAENIPPLQELTYHYNYVIDQVYDSSGNIKKKSCFCGSSECTGRLY

> CsSET2 orange1.1g006009m
MGSIVPFQDLNLMPSPSTAASTAAAATLPLLTPKIEPKTEPFDEPVPTHQLERGQNTPESLLSESAPGFFSNSENTPESQPPD
RDNVYSEFYRISELFRTAFAKRLRKYGDVDVLDPDSRAIVTVTHQDAQLSNAVVPRTKPMKRSGELVRVTDLSAEDERYFRDVVRRTRMLYDSLRVFAVY
EEEKRRGIGQGRRARGDLTASSVMKERQLWLNRDKRIVGSIPGVQIGDVFFFRMELLVVGLHGHSQAGIDYLPGSQSANGEPIATSIIVSGGYEDDEDAG
DVLIYTGHGGQDKLSRQCEHQKLEGGNLAMERSMHYGIEVRVIRGFRYQGSVSSKVYVYDGLYKIHDCWFDVGKSGFGVYKYKLLRIEGQPEMGSAILRF
ADSLRTKPLSVRPKGYLSLDISGKKENVPVLLFNDIDGDYEPLYYEYLVRTVFPPFVFTQGSNGAGCDCVSGCTDRCFCAVKNGGEFAYDHNGYLLRGKP
VIFECGAFCQCPPTCRNRVSQRGLRNRLEVFRSRETGWGVRSLDLIHAGAFICEYAGVVLTMEQAQIFSMNGDSLIYPNRFSARWGEWGDLSQVFSDYMR
PSHPSIPPLDFAMDVSRMRNVACYISHSPTPNVMVQFVLYDHNNLMFPHLMLFALENIPPLRELSIDYGVADEWSGKLAICN

> CsSET3 orange1.1g037821m
MLQVFLTDRHKGWGLRTLQDLPKGSFVCEYVGEILTNTELYERNMQSSGSERHTYPVTLDADWGSERILRDEEALCLDATFCG
NVARFINHRCFDANLIDIPVEIETPDRHYYHLAFFTTRDVSASEELTWDYGIDFSDHDHPIKAFHCCCGSEFCRDVK

> CsSET4 orange1.1g008407m
MHIADAEVNSKGGRRKNKPQKRTRSGRDINVTLPDIDVDSIVNNILSSYNLMEFDTVRRTDGDRDSVGYILLIFDLLRRKLSQ
IEDVREAMPGVARRPDLRVGTILMNKGIRTNVKKRIGAVPGVEVGDIFFFRMELCLVGLHHPIMAGIGYMGLTVNLEEESVAVSVVSSGGYEDNVEDGDV
LIYSGQGGNINRKDKEVTDQKLERGNLALEKSLRRGNEVRVIRGVKDLSTPTGKIYVYDGLYKIQESWTEKGKSGCNVFKYKFIRVHGQPEAFMTWKLIQ
QWKDGISLRVGVILPDLTSGAENIPVSLVNDVDDEKGPAHFTYLASLKYAQPVDSLEIFGGCDCRNGCVPGDQICPCIQKNAGYLPYTSNGVLVTQKSLV
HECGPSCQCPPTCRNRVSQGGLRVHLEVFKTKDKGWGLRSWDPIRAGAFICEYAGQVIDISKIEELGGENVDDYLFDATRTYQPVEPVPSDANGVPKIPF
PLIITAKDVGNVARFMNHSCSPNVFWQPVLRQSDKGYDLHVAFHAIKHIPPMRELTYDYGLPDKAERKKNCLCGSSKCRGYFY

> CsSET5 orange1.1g018070m
MNESVQLLTPRLAQYDFIGHNFVILFIPFLPQVCYFWKMCLGRYSRCLISRLQSLHLQKRQLCSTATHNGKPSQPSPPPIQVA
LTESAGRGVFATRRIRASDLIHTAKPIITHPTLSTLNSVCYFCLRKITSSSQHFQHHNARFCGEVCKDNAKAFYDVERRADWSAFNDYCRSQGLKYPLLV
KRLACMIISGAESADCIDILQPASLSPELILAMEEGFVMLRSAFKKAGIDDEQMKFLNKQWYTNVLAQIRINAFRIELAGGLYEDLLSSAAASIESEIAV
GNAIYMLPSFYNHDCDPNAHIMWIDNADARLMALRDVEEGEELRICYIDASMARDARQAILTQGFGFQCNCLRCSSGD

> CsSET6 orange1.1g046120m
MSAVSETVTVAEIEGRGRGLVSTQSLKAGQIVLRDSPILLYSALPFINSSSFCHNCFRKTMHSSSSICPSCSVAFCSPKCSTA
AASSSHSPYVCQALTRLLNLNSPDAANLPLDRQLQARFLIAAYNLAVVNPSQFQILLAFQGTVTDNDTSAAHYLSSLCPPPASTTIELTAALLAKDRLNA
FGLMEPYIEGQDGRRSVRAYGIYLKASFFNHDCLPNACRFDYVDAAAENNTDIIVRMIHDVPQGREICLSYFPVNYDYSTRQKRLLYDYDFACDCDRCKV
EANWSDNDNDDENNEEVMDEDQDEQMVASDDDAEVHGDTNFPHAYFFMRYMCDRDNCWGTLAPLPPSDATPSTVMECNVCGNLKNDAIGREDTVGMDD

> CsSET7 orange1.1g010495m
MYSNQELMPLFPSENLQCEEFPSYQHIYQNEFLSRKHKKQKEEDIAICVCRVDPNNLESSCGERCLNVLTSTECTPGYCPCGV
FCKNQRFQKCQYAKTKLVKTEGRGWGLLADENIKAGQFIIEYCGEVISWKEARRRSQAYETQGLKDAYIICLNALESIDATVKGSHARFINHSCQPNCET
RKWNVLGEIRVGIFAKQDIPVGTELAYDYNFEWYGGTKVRCLCGAATCSGFLGAKSRGFQEDTYLWEDDDERYSVEKIPLYDSAEDEPSLTLFKTVEATK
TEYVVDGKEEYSMGMNVSVKPENHLDSTSLVVQPLESVPMEGVVVNAIKIEESEETKLYPQDTQQQVFSQNNAMISRIRSNSACRNYHIGPESMPKKRSQ
LKSNGKLKHLAQKHVDAKHVCQLLAFKEAQEEVLRNEEMKNEASSQLASLYNDIRPAIEEHERDSQDSVATSVAEKWIEACCTKLKTEFDLYSSIIKNVA
CTPTRKPNQAKACDANSVTEVKYLGF

> CsSET8 orange1.1g042571m
MRSSATSYQSMLLQRKLRNTLSFTKLIRLLTVAILMAECLKLPVRQIMDVRGAIMLQLALRQQPVTIFLIRCVNMKFIHYLLL
AQKSGGSIEIFWGSYNIVCKMLFDHCMQVMWNAVFGDRVAEYSSAWRKRKLWSGHPKITGPASDYKDDRKRMEQAPSRHDSSVSDDDCPPGFGMVEIRTE
NDVQPYHLSLSVPVGENLSKQKNLSCNDHLLLDDVKCILDGVENELYLSTKATYTEYVEILVEDEVRKVVSASKGINMKEDVVDPSSHDLHTCQCGFADV
NGGMRIDSNETSAEIFSSEDSKSLFQAGKPLSKDLLSNILACAFKRSFSGFVDNVVDELETDEPSPPGFEDSVRKLVPSCNGKFQFSWSDEFTTKMGEYV
AIAMCRQKLHAIVVGEWKSLFVDDALQQFLALWCNMKECCEADGNEKAEGASNAHNEHHGDTSTVVDKLKEGSKRFHSSEASTMVEKYTYHRKKKLLRKK
FGSPSNCSNSVENAFQTEHVEKSRKQGVAGDVFENAKVQPSAVSSKKIGKNKLIDASSKKIGANKFTSVPSKMIGKNKVTAESSASAGSSKVKSKLPSGY
SSAKSTISQKVMKVTSAVQRDKVPVPKPSGEMLSTLSADGNDVGKVVRGKAHNVGIEKDSILDSSKSKPNATKESKQKRKRTMDGLELHATKALKVAKGT
AKQAASRQVAMKKTKASKSRTSNLCPRSDGCARSSISGWEWHKWSLNASPAERARVRGAQYVHTKYLGPEVNASQWANGKGLSARTNRVKLRNLLAAAEG
AELLKASQVKARKKRLRFQRSKIHDWGLVALEPIEAEDFVIEYVGELIRLKISDIRECRYEKMGIGSSYLFRLDDGYVVDATKRGGIARFINHSCNPNCY
TKVISVEGQKKIFIYAKRHIAAGEEITYNYKFPLEEKKIPCYCGSKKCHGSLN

> CsSET9 orange1.1g047072m
MAAGERGLQTNYVSGTSQMERKKHNLLPPPKSASEEIIKAKSSEGSYCKRNSYPGRNAYENRSALVMRDEKDSPGHDRGQENF
HLGQRSRVRETFRLFQAVCRKLLHEKEAKPDRQNSHKRVDYLVARILKDKKKYIPVHKKVIGSVPGVEVGDEFQYRVELNMIGLHLQIQGGIDYVKHEGK
INATSIVASGGYDDKLDNSDVLIYTGQGGNVMNGGKEPEDQKLERGNVALANNIHEQNPVRVIRGDTKAFEYRTCIYDGLYLVERYWQDVGSHGKLVYKF
KLARIPGQPELSWKVGLCVDDISQGKELIPICAVNTVDDEMPPSFKYITNIIYPDWCRPVPPKGCDCTNGCSKLEKCACVAKNGGEIPYNHNRAIVQAKL
LVYECGPSCKCPPSCYNRVSQQGIKVQLEIYKTEARGWGVRSLNSIAPGSFIYEFVGELLEEKEAERRTSNDKYLFNIGNNYNDGSLWGGLSNVMPDAPS
SSCGVYGNVGRFVNHSCSPNLYAQNVLYDHEDKRMPHKMLFAAENISPLQELTYHYSYMIDQVYDSSGNIKKKSCFCGSSECTGWLY

> CsSET10 orange1.1g000416m
MEVLPHSGVQYVGELDAKQSSGTEFVDNGESNCVQHENQVQMTNGKMDDMLSNVEGPVSERRGEGQRTGEELPSSEGHLGGVS
YFDCQLEGQGLSCGSHDFEDDDVNAQNECTGPCQASENSNLIVDTIESEVPNDNKEGESSFSEPKWLEHDESVALWVKWRGKWQAGIRCARADWPLPTLK
AKPTHDRKKYFVIFFPHTRNYSWADMLLVRSINEFPQPIAYRTHKVGLKMVKDLSVARRYIMQKLSVGMLNIVDQFHSEALVETARNVSVWKEFAMEASR
CVGYSDLGRMLVKLQSMILQQYINSDWLQHSFPSWVQRCQNARSAESIELLKEELYDYILWNEVNSLWDAPVQPTLGSEWKTWKHEVMKWFSTSHPLSNG
GDMEPRQSDGSLTTSLQVCRKRPKLEVRRPDSHASPLENSDSNQPLALEIDSEYFNSQDTGNPAIFASELSKGPGLREETAQTNTPSTVSNRWDGMVVGV
GNSVPIHTKDVELTPVNGVSTGPFNQTNMALTPLNELVTKKPLELGQRNRQCTAFIESKGRQCVRWANEGDVYCCVHLASRFTGSTTKAECALSADSPMC
EGTTVLGTRCKHRALYGSSFCKKHRPRTDTGRILDSPDNTLKRKHEETIPSAETTSCRDIVLVGEDISPLQVDPLSVVGSDSFLGRNSLIDKPEHSGKGY
SATEAQHCIGLYSQNSSNPCHESPKRHSLYCDKHLPSWLKRARNGKSRIISKEVFLELLKDCCSLEQKLHLHLACELFYKLLKSILSLRNPVPMEIQFQW
ALSEASKDAGIGEFLMKLVCCEKERLSKTWGFDANENAHVSSSVVEDSAVLPLAIAGRSEDEKTHKCKICSQVFLHDQELGVHWMDNHKKEAQWLFRGYA
CAICLDSFTNKKVLESHVQERHHVQFVEQCMLQQCIPCGSHFGNTEELWLHVQSVHAIDFKMSEVAQQHNQSVGEDSPKKLELGYSASVENHSENLGSIR
KFICRFCGLKFDLLPDLGRHHQAAHMGPNLVNSRPHKKGIRFYAYKLKSGRLSRPRFKKGLGAVSYRIRNRGAAGMKKRIQTLKPLASGEIVEQPKATEV
VTLGTLVESQCSTLSRILIPEIRKTKPRPNSHEILSMARLACCKVSLKASLEEKYGALPENICLKAAKLCSEHNIQVEWHREGFLCSNGCKIFKDPHLPP
HLEPLPSVSAGIRSSDSSDFVNNQWEVDECHCIIDSRHLGRKPLLRGTVLCDDISSGLESVPVACVVDDGLLETLCISADSSDSQKTRCSMPWESFTYVT
KPLLDQSLDLDAESLQLGCACANSTCFPETCDHVYLFDNDYEDAKDIDGKSVHGRFPYDQTGRVILEEGYLIYECNHMCSCDRTCPNRVLQNGVRVKLEV
FKTENKGWAVRAGQAILRGTFVCEYIGEVLDELETNKRRSRYGRDGCGYMLNIGAHINDMGRLIEGQVRYVIDATKYGNVSRFINHSCFPNLVNHQVLVE
SMDYQRAHIGLYASRDIAVGEELTYDYHYELLSGEGYPCHCGASKCRGRLY

> CsSET11 orange1.1g002418m
MASKASPSASPNRSEPLKSSSLTKTENGTLTRKEILSVIDCLKNQVAADHFVSVQRRVEKNRQKLIGVTNHLYRLSLERRNNQ
TINTHGSVDLLTKRQREALGVQNGIDVSSGDRDSHISQEDGYASTAVYGSSNPTKNIIRPIKLNDNKRLPPYTTWIFLDRNQRMTEDQSVMSRRRIYYDQ
NGGEALICSDSEEEVIEEEEKKDFVDSEDYILRMTIKEVGLSDATLESLAQCFSRSPSEVKARYEILSKEESAVGGSNNGNDEHTMNNFLVKDLEAALDS
FDNLFCRRCLVFDCRLHGCSQDLVFPAEKQPLWYHLDEGNVPCGPHCYRSVLKSERNATACSPLNGDIKEKFISSSDGAGAQTSSRKKFSGPARRVKSHQ
SESASSNAKNLSESSDSEVGQRQDTAFTHHSSPSKSKLVGKVGICKRKSKRVAERALVCKQKKQKKMAAFDLDSVASGGVLPSDMKLRSTSRKENEDANS
SSHKHAKSSSSGKTRKKEMQIQDSRNLMHVRVPLGSSQEIVSNPPAISTNDSLRKDEFVAENMCKQELSDEKSWKTIEKGLFDKGVEIFGRNSCLIARNL
LNGLKTCWEVFQYMTCSENKLFCQAGDAATSLLEGYSKFDFNGTTGNNEVRRRSRYLRRRGRVRRLKYTWKSAAYHSIRKRITERKDQPCRQYNPCGCQT
ACGKQCPCLLNGTCCEKYCGCPKSCKNRFRGCHCAKSQCRSRQCPCFAADRECDPDVCRNCWISCGDGSLGVPDQKGDNYECRNMKLLLKQQQRVLLGRS
DVSGWGAFLKNSVGKHEYLGEYTGELISHREADKRGKIYDRENSSFLFNLNDQFVLDAYRKGDKLKFANHSPDPNCYAKVIMVAGDHRVGIFAKERISAG
EELFYDYRYEPDRAPAWARKPEASGSKKEEGGPSSGRAKKLA

> CsSET12 orange1.1g041727m
MLLCGMLRRVRAFKRWMRSQGLEYSDVLDFKDDPEQGISVIALGDLKEGDLVATIPKSACLTVKTSGASDIIESAGLGGSLGL
AFALMYEKSLGEDSPWAGYLQLLPQQECVPCVWSLEEVDSLLSGTELHEIVKEDKGLIFEDWKQNILPLLDLNSANVKLNPDYFGVEHYFAAKSLVASRS
FQIDDFHGSGMVPLADLFNHKTGAEDVHFTSVSSHCQSDSDADNDDHVVANSDDNEPSTEHPHGDGEEFSAPSNGKSPLGGSDLESSALEDVPTVLQMIM
VKDVRAGAEVFNTYGLIGNAALLHRYGFTEPDNQFDIVNIDMELVLKWSSSLFSSRYIRARLSLWRKLGYSACQSQNSEYFELTSDGMPQIELLILLYIM
LLTENVYHKLDLKLSTMESYDEALCIILSEKNNIQLGKGSEMSEEQLLTESVCNAISSLADMRESLYPSNSKEDDIELLRSCSIRDRKLYHSLSLRVSER
KILEKLRTYAATHMLFPFSLYNFVRKITCSEPLFAT

> CsSET13 orange1.1g019353m
MLLRRRTEAPKPNIHRPITYESPDDDDSRCEKCGSGDFPDELLLCDKCDKGFHLFCLRPILVSVPKGSWFCPSCSHHKRPKPF
PLVQTKIIDFFRIQRSADLTQKLTPDNLRKRKRASGLVMSKKRRKLLPFNPTEDPERRLQQMASLATALRASGTEFTDELTYVTGMAPRSANSAVLEKGG
MQVLSKEDIETLNLCKHMMNRGEWPPLMVEYDPKEGFTVQADRFIKDLTIITEYVGDVDYLNNRENDDGDSTMTLLHASNPAQSLVVCPYKHGNIARFVN
GINNHTADGKKKQNLKCVRYNVNGECRVLLVANRDIAKGERLYYDYNGYEKEYPTEHFV

> CsSET14 orange1.1g001263m
MAFPQSLLHLKQQRQHHEEEEHLEEEEENDDDDDVLHKNAGTPIRYASLDRVYSACVTATSSTANGGSSNVMSKKIKASRKLC
RPPIVNVYTRRTKRPRRRQQHSSFLESLLGAREAEAERVDHSLAVKHEICEFENKIVGNDNHHDDHHDLRVLKKRKRFGSSELVKLGIDSISSVFSSFDR
PRLRDCRNNNSSSNNNKINNINLKRKKTDSNSKKILSVSPTAKRWVRLCCDGVDPKAFIGLQCKVYWPLDADWYSGFVVGYDSESNRHHVKYVDGDEEDL
ILSNERIKFYISQEEMDCLKLSFSINNVDNDGYDYDEMVVLAASLDDCQELEPGDIIWAKLTGHAMWPAIVVDESLIGDYKGLNKISGGRSIPVQFFGTH
DFARINVKQVISFLKGLLSSFHLKCKKPRFTQSLEEAKVYLSEQKLPRRMLQLQNAIRADDGENSWSQDEGSLGSGENCFKDERLQGTLGSIGISPYVFG
DLQILSLGKIVKDSEYFQDDRFIWPEGYTAVRKFTSLADPRVCNSYKMEVLRDTESKIRPLFRVTLDNGEQFTGSTPSTCWSKICMKIREGQNNTSDDFS
AEGAAEKISESGSDMFGFSNPEVMKLILGLTKSRPTSKSSLCKLTSKYRDLPGGYRPVRVDWKDLDKCSVCHMDEEYQNNLFLQCDKCRMMVHARCYGEL
EPVNGVLWLCNLCRPGAPEPPPPCCLCPVVGGAMKPTTDGRWAHLACAIWIPETCLTDVKRMEPIDGLNRVSKDRWKLLCSICGVSYGACIQCSNTTCRV
AYHPLCARAAGLCVELEDEDRLNLLSLDEDDEDQCIRLLSFCKKHKQPLNDRLAVDERLVQVTRRCCDYIPPSNPSGCARSEPYNYFGRRGRKEPEALAA
ASLKRLFVENQPYLVGGYCQNGLSGNTLPSIRVIGSKFSFSLHRDAPNFLSMADKYKHMKETFRKRLAFGKSGIHGFGIFAKHPHRAGDMVIEYTGELVR
PSIADRREHFIYNSLVGAGTYMFRIDDERVIDATRAGSIAHLINHSCEPNCYSRVISVNGDEHIIIFAKRDIKQWEELTYDYRFFSIDEQLACYCGFPRC
RGVVNDTEAEEQVAKLYAPRSELIDWRGD

> CsSET15 orange1.1g011626m
MEELQKALQDRGLTVTGLPEKGRCLYTTKDFYPGEVIISQEPYVCVPNNSSSISRCDGCFASSNLKKCSACQVVWYCGSNCQK
LDWKLHRLECQVLSRLDKEKRKSVTPSIRLMLKLYLRRKLQNDNVIPSTTTDNYSLVEALVAHMSDIDEKQLLLYAQIANLVNLILQWPEISINEIAENF
SKLACNAHTICNSELRPLGTGLYPVISIINHSCLPNAVLVFEGRLAVVRAVQHVPKGAEVLISYIETAGSTMTRQKALKEQYLFTCTCPRCIKLGQFDDI
QESAILEGYRCKDDGCSGFLLRDSDDKGFTCQQCGLVRSKEEIKKIASEVNILSKKTLALTSCGNHQEVVSTYKMIEKLQKKLYHPFSVNLMQTREKLIK
ILMELEDWKEALAYCQLTIPVYQRVYPQFHPLLGLQYYTCGKLEWFLGDTENAIKSMTEAVEILRITHGTNSPFMKELILKLEEAQAEASYKLSSKDE

> CsSET16 orange1.1g017646m
MPAAKKNSDNSRIGHAFNKLLKQIGNPVEFELPDWFIKPKAIPYVFIKRNIYLTKRIKRRLEDDGIFCSCTASPGSSGVCDRD
CHCGMLLSSCSSGCKCGNSCLNKPFQNRPVKKMKLVQTEKCGAGIVADEDIKRGEFVIEYVGEVIDDQTCEERLWKMKHLGETNFYLCEINRDMVIDATY
KGNKSRYINHSCCPNTEMQKWIIDGETRIGIFATRDIKKGENLTYDYQFVQFGADQDCHCGAAGCRRKLGAKPSKPKISSDAALKLVACQVAVSSPKLKA
ILSGKDFYQNGDLHIGSSRPPYNQRQICPQCCIGKVIRISHPKNESSFGIIRRFDEYSRKHSVLFEDGESEFIDMAKVDWELVTD

> CsSET17 orange1.1g035535m
ELMQQLRSKATELLLREEWKESVQVYTQFIDLCQSQITETKQEASQLSKLKKSLCLALSNRAEARSRLRDFDNALRDCEQALK
IESSHFKALLCKGKILLSLNRYSMALDCFKETLVDAQASGSLETVNGFLEKSKKLEYQSRTGALDLSDWILNGLRGKCPELAEYIGAVQISKSEISGRGL
FATKNVEAGTLFLVTKAIATERGILSGENSNENEQLVMWKNFIDKVMESISKCQRTRHLISILSSGDNEDEVEVPDVSAFRPEAEERRSSNEKLDMGKIL
SILDVNSLVEDAISAKVLGKNKGLYGLGLWALASFINHSCSPNARRVHVGDYIIVHASRDVKAGEEITFAYFDMLLPLEKRKEMSKTWGFHCKCKRCKFE
EGMSSKQELSEIEIGLERGIDAGNAVFRLEENMKRWIVRGKEKGYLRASIWSAYAETYGSERLMKRWGKRIPAAEAVVDSVVEAVGCDERMLKVLIEGLK
GSSCGMPEMERPIKLGKGLYGKLVKKQALKSLLEI

> CsSET18 orange1.1g010945m
MAALSNFSFSLSTSPPTFVSSSKTLKLLTSLTKKPSFRLKSRAFAATCSLHSASATTNPPTAQVETFWQWLRDQKVVSPKSPI
RPATFPEGLGLVAQRDIAKNEVVLEVPMKFWINPDTVAASEIGSLCSGLKPWISVALFLIREKKKEDSPWRVYLDILPECTDSTVFWSEEELVELQGTQL
LSTTLGVKEYVQNEYLKVEEEIILPNKQLFPRPITLDDFLWAFGILRSRAFSRLRGQNLVLIPLADLINHSPGITTEDYAYEIKGAGLFSRDLLFSLRTP
VPVKAGEQVLIQYDLNKSNAELALDYGFIESKSDRNAYTLTLEISESDPFFGDKLDIAETNGLGESAYFDIVLGRTLPPAMLQYLRLVALGGTDAFLLES
IFRNTIWGHLDLPVSHANEELICRVVRDACKSALSGFHTTIEEDEQLLQGGNLDPRLRIAVGVRAGEKRVLQQIDEIFKERELELDELEYYQERRLKDLG
LVGEQGDIIFWEPK

> CsSET19 orange1.1g036712m
MPNPQFCKIAESCEENEVADEYAYVANPKKRRRRGGDDCHQSLMHVEVDDLSSGSSSFISEEATCWDPEFEPDLNNFNYKGRG
TNRSSDRFRPPALKPSKGRTQILPSRYDDSVLVVGDTDSSFDEEDDVDIIEVNGDFDKLGFTMDKYRFGNSNYRGYNGFDPREYLVSRRPVMPAGNVNSL
PMAGKKQFMPGFSSRNVERITKEKEKKKKRKDVYKPEDFALGDLVWAKCGRSYPAWPAVVIDPILQAPEAVLRCCIPGCLCVMFFGYSKNGTQRDYGWVK
QGMLFPFAEFMDKCEPTQLHKSKISGFQIALEEAVLAENGFLDLNLGIGQIGPEAYSRRGQEATGSGQDLEYCPQNQNACYKVARVCDGCGLFRPCKLKR
MKGLVSETQFLCKHCSKLQKSEQYCGICKNIWHHSDSGNWVCCDGCNVWVHAECDEISGKHFKDLEHIDYYCPNCRVKFKFQSSNIGKWQPGVSAVENDG
QMVLPDKIMVVCNDVEGAYFPKLHLVVCRCRSCGPKKLTLSEWERHTGCRAKKWKYSVKVLGTMLPLGKWTMQITEFNADAMDPVKLDEKKLLAFMKEKY
EPVSVKWTTERCAICRWVEDWDYNKIIICNRCQIAVHQECYGVTDVQDFTSWVCRACEMPNAERKWGALKPTDVQTLWVHVTCAWFRPEIGFLNHEKMEP
ATGILRIPTNLFLKSCIICKQTHGSCTQCCKCATYFHAMCASRAGYCMEIHSLERYGKQITRKLIYCAVHRTPNPDAVVAFHTPTGVFAGRSLLQNQRGC
FRGSRLVSAKRTEDSESPSPDTNDFEPLSASRCRVFKRSKNKSMEREPICHRPMGPRHHSLDAVISLNTYKEVDKPEIFSSFKERLYHLQRTEKHRVCFG
KSGIHGWGLFARRHIQEGEMVVEYRGEQVTQSIADLREKQYRKEGKDCYLFKISEEVVIDATNKGNIARLINHSCMPNCYARIMSVGDCESRIVLIAKTN
VSAGDELTYDYLFDPDEHDELKVPCLCKAPNCRMFMN

> CsSET20 orange1.1g000582m
MASSDDEVEVGQKLVSDYYFEHEGERKQKEIISFSALPIQWNENERTGGCKELIYLRGAADSGLQKIFKPVIAWKFDLTNVIP
EIFVLSKENSWIKLQKPRKCYEEIYRTILIMVHCLSYAKRNPEATAKSIWDFLSRFFCLYEVRPSQNDLVDHMDLIKEALERDDVLAKSKFLVTFLEEKP
TKRKLSDEVVQTKAMSGFIVDDMEEDMVHDTEEDESNEEDELFDSVCSFCDNGGDLLCCEGRCLRSFHATIDAGEESHCASLGLTKDEVEAMLNFFCKNC
EYKQHQCFACGKLGSSDKETGAEVFPCVSATCGHFYHPHCVSKLLLRDDEVAADQLAKSIIAGESFTCPLHKCCICKQGENKADSDLQFAVCRRCPKAYH
RKCLPRKIAFEDKLEEGIITRAWEGLLPNHRILIYCLKHEIDDEIGTPIRDHIIFPGIEENKTIIDRPRKKQSLASPSGKQKVASTKSSLTSKAPPQGKF
SVKALKRVPSKAGQGETMEISERLLVGSDSSRRAKATDVSRKSFKGNVKSLSVQVDRSSSVDSKKTSLGERLYAAFVTEGTEQTKFGKQDNSDRETSRTV
TVKPLRKKLISELPSLDEDSKRRLSSLMKDAASSVRMEEILKRHKIPSTHAYASKSAVDKAITLGKVEGSVEAIRTALKKLDVDGSSIEDAKAVCEPEVL
SQIFKWKNKLKVYLAPFLHGMRYTSFGRHFTKVDKLQAIVDKLHWYVNDGDMIVDFCCGANDFSCLMKKKLDETGKNCLYKNYDILPAKNDFNFEKRDWM
TVEPKELAPGSRLIMGLNPPFGVKAGLANKFINKALEFNPKLLILIVPPETERLDRKESAYELVWEDDQFLSGKSFYLPGSVDENDKQMDQWNMTAPPLY
LWSRHDYAAHHKALAEKHGHISRPQSRTQMERNCYETHAVDHPKEEGQGDASMLIDLPLQINVTKELRNEAREDDKAGFPDNATEGGGESSHGHGDNQSG
KTSRKRKRDRKKHGSGMRENSPLDGQNRGRHLASGIHGMSKHSPANIANVSPLLEGHSSKSIDMPSHVGSGDNDCQHFSNKGMPLSSPTIVIDGTSPLGV
HSSKTIEMLSHDDGCQCDQMPHRSPVNVSSGILEGESSKPIEIPALSGIGDGGYEHFGVGMSHHSAATVIDGASSLQGLSSKSIEMPSYTQFDDNVHQHF
EDKGVPRCSPAKVIAKSSLEDHSSKSIDIPSQTGFGSDYQHHEPTRSSSHIGTTYYGTQAGIPNDMGSYGMSSLNNGLSHGANLDERYTGYVRNTDSLGY
RPSMSTDRELTMWPLARIYGQDFPAPTPGYGQMGSVPSNLYGNLGSSAEASYRMSTSAMDRYAPRLHQLNNTRMNTFRSEPFMPSRFGFYDSRAPQPGFF
ADMDFGPGFHPPFPQQGSGGWLDD

> CsSET21 orange1.1g038692m
MPDLANLALSSSSSSLTLTRCASSLKPLAPPHSATESPGSDSAVVKTLALTGEEENVCANGNGHSVRVMKRCRGAKNISGLED
HVAAWVKKKMELGVPQSNCSLPFLVGAKKMIECRACHRFIYHGEEVFCSVRGCGGVYHFICVKERLGISNPRNFKCPQHACFICRQRLQWRCVRCTIASH
DKCAPWPDRVIHLKDQPGRAVCWRHPAKWLLDKQEVFCRLPLPYADEEFKIDLTWKDLMENKVGPPPVQYISCSKACHCSETCNNRPFRKEKKIKIVKTE
FCGWGVEAAEPINKGEFIIEYIGEVIDDALCEQRLWDMKYRGVQNFYMCEIRKDFTIDATFKGNFSRFLNHSCDPNCILEKWQVEGETRVGVFAARSIKA
GEPLTYDYRFVQFGPEVKCYCGASSCQGYLGTKRKIGKLELCWGSKRKRSSTACLAIITL

> CsSET22 orange1.1g006089m
MDGTVPSAPLDKTKVLDVKPLRSLRPVLPSSPQAPPFVCAPPFGPFPPGFSPFYPFSTPEFTPDNNQNNNTQTPPTSFATPIR
SFRSPDVNFVDGSNGDLGSSDGFLDGKKRRTSSYKQKRPKNAQDSDFSVGISSFERDDGNRQVVNNVLMRFDALRRRISQIEDAKETSTGLIRRADLKAS
NILMSKGVRTNMRKRLGVVPGVEIGDIFFFRMEMCLIGLHSQSMAGIDYMITRSDLDEEPVAVSIISSGGYDDDAEDSDILIYSGQGGNANRKGEQAADQ
KLERGNLALERSLRRASEVRVIRGMKDAINQSSKVYVYDGLYTVQESWTEKGKSGCNIFKYKLVRIPGQPGAFALWKLIQRWKDGMSGRVGLILPDLSSG
AEAIPIALINDVDDEKGPAYFTYLTTVKYSKSFRLTQPSFGCNCYSACGPGNPNCSCVQKNGGDFPYTANGVLVSRKPLIYECGPSCPCNRDCKNRVSQT
GLKVRLDVFKTKDRGWGLRSLDPIRAGTFICEYAGEVVDKFKARQDGEGSNEDYVFDTTRTYDSFKWNYEPGLIEDDDPSDTTEEYDLPYPLVISAKNVG
NVARFMNHSCSPNVFWQPIIFENNNESFVHVAFFAMRHVPPMTELTYDYGISKSDGGNYEPHRKKKCLCGTLKCRGYFG

> CsSET23 orange1.1g002895m
MVSRASDSSSKSRKSYSEQSNDGLGNLTYKLNQLKKQVQAERVVSVKDKIEKNRKKIENDISQLLSTTSRKSVIFAMDNGFGN
MPLCKYSGFPQGLGDRDYVNSHEVVLSTSSKLSHVQKIPPYTTWIFLDKNQRMAEDQSVVGRRRIYYDQHGSEALVCSDSEEDIIEPEEEKHEFSDGEDR
ILWTVFEEHGLGEEVINAVSQFIGIATSEVQDRYSTLKEKYDGKNLKEFEDAGHERGIALEKSLSAALDSFDNLFCRRCLLFDCRLHGCSQTLINPSEKQ
PYWSEYEDDRKPCSNHCYLQSRAVQDTVEGSAGNISSIITNTEGTLLHCNAEVPGAHSDIMAGERCNSKRVLPVTSEAVDSSEVAIGNENTDTSMQSLGK
RKALELNDSVKVFDEIEESLNKKQKKLLPLDVLTASSDGIPRPDTKSGHHVGAINDNELQMTSKNTIKKSVSAKVVSHNNIEHNIMDGAKDVNKEPEMKQ
SFSKGELPEGVLCSSEWKPIEKELYLKGVEIFGRNSCLIARNLLSGLKTCMEVSTYMRDSSSSMPHKSVAPSSFLEETVKVDTDYAEQEMPARPRLLRRR
GRARKLKYSWKSAGHPSIWKRIADGKNQSCKQYTPCGCQSMCGKQCPCLHNGTCCEKYCGYSFLRCSKSCKNRFRGCHCAKSQCRSRQCPCFAAGRECDP
DVCRNCWVSCGDGSLGEPPKRGDGQCGNMRLLLRQQQRILLAKSDVAGWGAFLKNSVSKNDYLGEYTGELISHREADKRGKIYDRANSSFLFDLNDQYVL
DAYRKGDKLKFANHSSNPNCFAKVMLVAGDHRVGIFAKEHIEASEELFYDYRYGPDQAPAWARKPEGSKREDSSVSQGRAKKHQSH

> CsSET24 orange1.1g024518m
MQVAKGVKLPTTAIGCDCRGNCLNSHDCSCAKLNSTDSKHYDFPYVHRDGGRLVEAKAVVFECGPKCGCGPDCINRTSQRGLK
YRLEVYRTPKKGWAVRSWDFIPAGAPVCEYIGVLRRTEDLDNACDNENNFIFDIDCLQTMRGLGGRERRLRDVSISTIYNSDRPDDQKVENTPDYCIDAG
AVGNVARFINHSCEPNLFVQCVLSSHHDLKLARVVLFAADNIPPLQELTYDYGYELDSVHGPDGKVKQMVCYCGAEGCRGRLF

> CsSET25 orange1.1g044651m
NPIFVEDITRGEEKQPISLLNENGTSELPKFLYISKNTVYKNAHVNFSLARIGDENCCLNCSGNCLSAPANCACTSETRGDFA
YTAAGLLDEKFLRESIAIIRRKNDKKHLFYCENCPLENRLVNGNRNHKRKRSVKPCKGHLMRKFIKECWAKCGCSLNCGNRVVQRGITVKLQVFQASEGK
GWGVRTLEALEKGTFVCEYVGEVVTNQELDERNEEFSGDRHTYPVLLDADWASERFLKDEEALCLDATKFGNVARFINHRCYDANLIEIPVEIETPDHHY
YHVAFFTTRKVEVNEELNWDYGIDFSDETHPIKAFDCKCGSFFCSMKSQS

> CsSET26 orange1.1g017938m
MAFSNAPTELINCHKRTIEVDEEQKQNQFLQWARLILPWLTPGELANVSLTCRTLSQISKSITLSRSLDASRSVENFPIPFHN
AADKTPYAYFIYTPSQIIPPPCPAQFPPRQFWASTNAAADAESNSSLSRLGFDSVSLVCESDESESGCDCEECFEVGLGDGVFGCPCFSGLEDVGIVSEC
GPSCGCGSECGNRLTQRGISVRLKIVRSVNKGWGLYADQFIKQGQFICEYAGELLTTKEARRRQQIYDGLASSPRNSSALLVIREHLPSGKACLRMNIDA
TRIGNIARFINHSCDGGNLSTTLVRSSGSILPRLCFFASKDIKEGEELAFSYGEIRARPRGLPCYCGSTSCFGILPSENT

> VvJmjC1 GSVIVT01016246001
MSRKCHVNQIPEAIPISAQLKSYGTENSNSKPCDVSHRRSVIPIWLKGLPLAPEFRPTDTEFADPIAYISKIEKEASAFGICK
VIPPLPKPSKRYVISNLNKSLSKCPELGSDVNASTVCSSAKMGSGDGDADGEARAVFTTRHQELGQNLKRTKGVVQPQAGVHKQVWQSGEIYTLEQFESK
SKAFARNLLGMIKEVSPLVVEAMFWKAASEKPIYVEYANDVPGSGFGEPEDETSRQKNLNGSNEMEGTAGWKLSNSPWNLQVIARSPGSLTRFMPDDIPG
VTSPMVYIGMLFSWFAWHVEDHELHSLNFLHTGSPKTWYAVPGDYAFAFEEVIRSQAYGGNIDRLAALTLLGEKTTLLSPEVVVASGIPCCRLIQNPGEF
VVTFPRAYHVGFSHGFNCGEAANFGTPQWLKIAKEAAVRRAAMSYLPMLSHQQLLYLLTMSFVSRVPRSLIPGARSSRLKDRQKEERELLVKQAFIEDML
NENNLLSVLLGKGSTYRAVLWDPESLPSSTKEPQLSTEITTVSTKPRENISEVENKDDSNQNDLFDKMSLYIENVNDLYLDDDDLLCDFQVDSGTLACVA
CGILGFPFMSVVQPSDRASMEFLHADHPLVEDRAGDTETMKSYCPSAVHGTSKGPVSDETTKEEISSAILMTENLKCRKDLKLIKDGKESSIDANSLSSE
SLQMPLITNFEKGWNKSTELLRPRIFCLEHAVQIKELLQPKGGASMLIICHSDYQKIKAHATTVAEEIGHPFNYNEIPLDTASQEDLNLINLAIDDEEHV
ECGEDWTSKLGINLQYCVKIRKNSPSKQVPHALALGGLFTDTTSSSNFLSLKWQSRKSRSKLKSNLPSHIKPYESNQIKEVEVMEGKSVGSTIRKEDKLI
QYSRRIFKFKSGGAEGASRARGRPRKNLPKDVSATSCDIVKNISRTSNNSPNIEKEGGESAGLDFYASFGKSEMLHEVQVLEATEDLSKNAVPAQVINPL
VTATPVVKSVEARINNQTLEDEACNSVTCDGSEMPLEINITEVTGEKNKILGAENDSTLPIISVPTVEKSGIQMDHQIMEEVNMTNEPGNLTQYNSEGQH
GIQGDGDVLMNEVSDCDNFTSSHGPVGEGFDAQIENVVIEESCTNGEIGECMILDKEASEQGILIADGSGDEEHILSNDAMTNQPPPPSTVESSEIPREI
CPVNPKSTKKAERKRKREGGQKTEDKFYFDSFIRSPCEGLRPRAKKDGSTGADTNKPVVEKPMAKTRKPADTSGPHKDKKENTKGSHRCDLEGCRMSFKT
KAELLLHKRNRCPHEGCGKKFSSHKYAMLHQRVHDDERPLKCPWKGCSMSFKWAWARTEHVRVHTGARPYQCKVEGCGLSFRFVSDFSRHRRKTGHYVNN
TPKRKQWQPPRHLLHQLQRPPHPQHCLRLAPSEIQSSPESESLTHPMFAPSTSSCTSSPSSSASPTFSPPLSPPFHPTSSTLLLSNPSPSSSLKIRIGRR
RRGGRVRAVFPKLFDVLGEARVLYRGFVCQGLLQEERVWEDVVVCGGSSGGENGVWESGVVRCNTSK

> VvJmjC2 GSVIVT01007692001
MGLRIGGEIEKENGDELSYSDFVERYLMKNRPVVLRGLMDGWRACKDWVTHTGQPNLEFFSTHFGKSIVQVADCGTREFTDQK
RMEMSVAEFIDHWLKDSANYHVNATTNEHGKPLVYLKDWHFVKEYPEYLAYTTPLFFRDDWLNLYLDNYSMHNEPDACQEKNEISCSDYRFVYMGAKGTW
TPLHADVFRSYSWSANVCGKKKWLFLPPSQSHLVFDRHEYVFLHIYSVYNICEDVNEAKFPGFKKAVWLECIQEQDEIIFVPSGWYHQVHNLEDTISINH
NWFNAYNLSWVWDLLLKDYNEAKEYIEDVRDICDDFEGLCQRNLAANTGMNFCDFFIFIACFSFANLVQLYHLHRVNENPSWCLFPKAQHLALNLLSAQK
IVLKMKTVDALAGDHALLLDLRKMMDDSKFLELCSGLDRTYRGIHKEQQQNYDKKETLMDGLGDLDFIVKSCSQIYAPEDLVSFIDSAVKKLSRAFNRES
PLLPELDELRSPDAQ

> VvJmjC3 GSVIVT01013083001
MTRSGGDALRPSSSCGVRLHGNTDSFYRSSGALNEKDAFSKRKVDKFDTTDLEWIDKIPECPVYRPTKEDFEDPLVYLQKIAP
EASKYGICKIISPLSASVPAGVVLMKEKMGFKFTTRVQPLRLAEWDSDDKVTFFMSGRNYTFRDFEKMANKVFARRYCSAGCLPSSYLEKEFWHEIACGK
TETVEYACDVDGSAFSSSPNDQLGKSKWNLKKLSRLPKSILRLLESEIPGVTDPMLYIGMLFSMFAWHVEDHYLYSINYHHCGASKTWYGIPGHAALEFE
KVVREHVYTRDILSADGEDGAFDVLLGKTTLFPPNILLEHDVPVYKAVQKPGEFVITFPRAYHAGFSHGFNCGEAVNFAIGDWFPLGAVASRRYALLNRM
PLLPHEELLCKEAMLLYTSLELEDPDYSSTDLASQHSMKLSFVNLMRFQHNARWALMKSRACTAIFPNSGGTVLCSLCKRDCYVAYLNCNCYLHPVCLRH
DVNSLKLPCGSNHNHTLSLREDISEMEAAAKRFEQEEEIFQEIQHAKSDDDLSPLSDMFLISEEDGYYPYCEIDFGLVPGIPVATQDESPELEQSAPSQP
PFNSGREYFRTEMSDASLSCAASTLCSFLKPVESSSIPRNVQGDAKFNLGDHVSRKFSEDISQNIHESCLSSLSCDECLSTHQNFHGSEVKPIIDQDSDD
SDSEIFRVKRRSSVKVEKRNANDASSVKHFDHQGLKRLKKLQPQGRSKH

> VvJmjC4 GSVIVT01025322001
MGLIMGGTLLRQDLHAAVEKVTAKASELRVSEEEQGEVIVGGENAYHEAEVLGVLPMKSLSSKIIGKRSALSLEGFLCDYFMS
GSPVIISDCMGHWPARTRWNDMDYLKRVAGDRTVPVEVGKNYLSSDWKQELITFSQFLERIQSSDCTSTLPTYLAQHPLFDQIHELRKDIFIPDYCYAGG
GELRSLNAWFGPAGTVTPLHHDPHHNILAQVVGKKYIRLYPASLSEELYPYTETMLCNSSKVDLDNIDEKEFPKVKDLEFQDCILEEGEMLYIPPKWWHY
VRSLTTSFSVSFWWSNCGVLSLLQNLTPESFQRLLHLLCPYLFGPRFVFSLVDDAALKNSSMAFTEEPLKPSSSLMASFIISLTSLTFFLMSFLFSVAFI
TNSIFLTISSCLTSHIGPLATSTHTPISSSKNFAL

> VvJmjC5 GSVIVT01027160001
MEASVDFRCKKSAGKWRCSETASKGKSYCEKHCLQIKNQSERKKREREEGKISGSGEFAGGGGGERTGEKRRRRKESDSDGSD
DNSTLVKDLRKRHPITKKDRVNRIVDINSDKIESNCGNGKAESGGGQRSSTEDQSKSGSRISDKLNKNKEHGSLMCHQCQRNDKSGVVHCSSCTRKRYCF
ECIAKWYPEKTRDEIESACPFCCGNCNCKACLREVLFVKANHKELDDSVKLQRLQYLLFKALPVLRHVHQEQKSEVEIEAKIRGVQLMESDITRSKLEKN
ERLYCDNCNTSIVDFHRSCPNPDCSYDLCLICCRELREGRQPGGSEAETSHQQFVERAHGQLAADDSKADVSNQFPDWRATGDGSIPCPPKERGGCGTAI
LELRRNFKANWVMKLIQSSEDLICHYQLPDHNFSQGCSLCWPNVTGRNSEQNSEMRKAAFRKHGHDNFLFCPNAVNITDDEIEHFQRHWMRGEPVIVRNV
LDKTSGLSWEPMVMWRAFRETGAKTKFKEETRTVKAIDCLDWCEVEINIHQFFAGYLEGRMHKGGWPEMLKLKDWPSSTLFEERLPRHGAEFIAALPYCD
YTDPKSGFLNIATKLPTESLKPDLGPKTYIAYGFPLELGRGDSVTKLHCDMSDAVNVLTHTAKVKVAPWQHKRIKTMQKKHAIGDLHELYGGISEAVDES
ENIVEKDHLLPEQKKSKDQLDEDNETMAEEDASNQDGLNSSSDTTTNDSLQNIDDSTVVHGGAVWDIFRRQDVPKLIEYLQKHQKEFHHINNLPIKSVIH
PIHDQTLFLNERHKKQLKEEYNVEPWTFEQNLGEAVFIPAGCPHQVRNRQSCIKVALDFVSPENVQECIRLTDEFRLLPKNHRAKEDKLEVKKMTLYAVS
SAVREAKKIISNLK

> VvJmjC6 GSVIVT01019761001
MYGSDLDTSVYGSGFPRVNDKKPESVEDEIWDKYCASPWNLNNLPKLQGSMLRAVHNNIAGVMVPWLYVGMLFSSFCWHFEDH
CFYSMNYLHWGEPKCWYSVPGSEAIAFEKVMRNCLPDLFDAQPDLLFQLVTMLDPSVLQENGVSVYSVIQEPGNFVITFPRSYHGGFNFGLNCAEAVNFA
PADWLPHGGFGAELYQLYRKAAVLSHEELLCVVAKANDCDSKALPYLKKELHRIYAKEKNCREGLWSNGIIKSSPMSPKKCPEFVGTEEDPTCIICQQYL
FLSAVVCCCRPSAFVCLEHCKHLCECKPNKHRLLYRHTLAELKQLVLLIDKYNFDETPQCRDLQRQLSCSDDSNALTKKVKGGHVSLAKLAEEWILRSSK
IFQIPFSRDAYVNALKETEQFLWAGSEMDAVRAVAKNLIEAQNWAEGIKDCLCKIESWSCNRSHNLEKVDLEHVNNFLNLNPLPCIEPGHLKLKGYAEEA
MILVQEIDSALSTSSKSSIPELEQLYSRACEVPIYVKEMEKLMARISALKMVINIIAWFSDSFFLSNLMILMKFFHPLFVWVDNVKKCILEKCPAAIEVD
VLYRLKSEMLELQVQLPEVEMLMDLLRHVESCQARCNEILNGPINLKNVEVLLQELESITVNIPELKLLRQYHGDAVSWISHFNDVHVNIHEREDQENVV
DELQCILKQGLLLRIQVDELPLVEVELKKAYCRKEALKARRTKMTLFSIQQLMEEAAMLQIEGEQLFVDVSGVLAAAMHWEERAAHIFATEAQMSDFEDV
IRTSKDIHVILPSLDDVKDAISMAKSWLKNSKPFLGSSFPAAHPSCSLLKVEALKELVSQSKLLKISLEERTMIHSVLKNCLEWEHDSCSLLEEVDCLFN
TNNIDNALINGLIPKIEHLVTMIESILETGLSLGFDFDEIPKLQNARSILQWCSKALSFCSVAPALPGIESLMENAEHLPVTCASSALCSSLIDGVKWLK
KASEVIPVSCNGKICKLSDAEEVLSEVQRIKVSFPLMVGQLLKAIEKHKLWKEQILIFFGLKTEERSWSKLLQLKVIICFMYFGISFNVIQIKHTLDRSL
YIYKKSRGCNPRDPCIHCFSDIKDQELLTCSICKDCYHLQCLGATLGHQSDAEAYVCSYCQFIGSGSISRNGGALRFGGKRPELNMLIELLSDAEGLCVG
CVIEERDVVQQLVELAIACKDCLTELTDFTLAYLNRDLSIISEKLTTALKAVEMAGVYYNHGNNRLELALARNSWRVRVNKLLEDSQKPLIQHIQKILKE
GLAISIPPEDHFRQKLTELKCIGLQWAENAKKVSMDSGALGLDEVCELITQGENLPVHFEKELKLLRARSMLYCICRKPYDQRAMIACDQCDEWYHFDCI
KLSSAPKIYICPACKPHTGELSVLLSVNKERSTGAKYGEPQTPSPPHTESRRKNIEAKPSLKQMMPAAMDHGNILRYSDGIDCLFWRNRKPFRRVAKRRA
EVESLSPFFHIQ

> VvJmjC7 GSVIVT01033203001
MAKSKKKDVDLPPPDHLRCNRNDGKGWRCRDYKLEGHNLCQYHHDSAISRSQKSKPDRKPKTKPQPKPKPKTEAEPKPQMEPE
SKPDAKLKISRSKRAPGRSGAKSSDATLRSDAARGSEVGQGREVARGGDGGRRYPSRGKLLRRLLDSDTDQEWRGNGSNAKKRNAQEDECRCCHQCQKSD
REVVRCRKCQRKRFCHPCIERWYPRVSKEAIAEACPFCSGNCNCKACLDRDTKTLEPEMSKDDKIKHSKYLVKVLLPFLEQFDHEQEMEREIEAKIQGLS
PPEIQVQQAVLREDERVYCNNCRTSIVDFHRNCPNCSYDLCLTCCREIQSNFCVSSSSKDPGSTICEWKVKENGDIPCAPKEMGGCGHGRLDLKCMFSET
WVSELKEKAEGLVKTHKLTDVLGIPACSCSCFKLNSEIDFDNKKLRKAAAREDSFDNYLYCPSESDILQGDLVHFQSHWMKGEPVIVSDVLEFTSGLSWE
PMVMWRAFRKVSYTKSSQLAEKAIDCLDWCEVEINIHQFFKGYSEGRAHRNLWPEMLKLKDWPPSNLFQERLPRHGAEFISSLPYLEYTHPRSGLLNLAA
KLPQKSLKPDLGPKTYIAYGVVEELGRGDSVTKLHCDMSDAVNVLMHTAEVTLSSQQLAVIEKLKKCHAAQDQKELFAAIHTEQGEFSDDHMASGNKLVG
FDKEGGGAVWDIFRRQDVPKLQEYLRKHHREFRHTHCSPVEQVVHPIHDQTFYLTLHHKRKLKEEFGVEPWTFVQKLGEAVFIPAGCPHQVRNLKSCIKV
ALDFVSPENIHECVRLTEEFRALPHNHRAKEDKLEVKKMSLHALRQAVDNLEQLTGDEAAVPSPPETTEPPTDSHQSSE

> VvJmjC8 GSVIVT01037956001
MTFHPTTSITNFIPFSIWLIGSIYFFNIKSGLSQLCDLVLLRIVFGIFWKDWVCHQKGPFFQRVVIDKLGFDTDCFWSMEQSS
LEPEFQIKEDHSSKHALKNDSNIEYSGSPQNQKISARWNPTEACRPLIEEAPVFYPTVEEFQDTLNYIASIRPKAEPYGICRIVPPPSWVPPCPLREESI
WKHLKFPTRMQQVDLLQNREPMRKKNRGRKRKRRRYSRMGTTRRHSRSEVSEANIVSDSDEKFGFHSGSDFTLEEFQKHADSFKEFYFGIKDAKDNLNSD
GVECNKRWEPSVEDIEGEYWRIVEKPTDEVEVYYGADLETEAFVSGFPKASSLISENDSDQYVASGWNLNNFPRLPGSVLCFEQNDISGVLVPWLYVGMC
FSSFCWHVEDHHLYSLNYLHWGDSKVWYGVPGSHASALENAMRKHLPDLFEEQPYLLNELVTQLSPSVLKSENVPVYRAIQNSGEFILTFPRAYHSGFNC
GFNCAEAVNVAPVDWLSHGQSAVELYSEQCRKTSISHDKLLLASAQKAVQALRDPSVLGKEDQVNLSWKSVCGKDGTLTKAVKTRVQMEEERLDRLPIGW
RLQKMERDFDLKNERECFSCFYDLHLSAASCECSPDQFACLKHASLICSCEPNRKFVLLRYTMDDLKTLVESLEGGLDAIEVWASEDLGLVSADKDACLT
KGYESKVGQGFCIDLNLDTMSDEHVSGLQQVSYSCDSKATGNMNFCVEPMHFGTVLFGKPWCSKQAIFPKGFTSRVKFFSVCDPTQMCYYISEVLDAGLL
GPLFKVTSEGCPSETFANVSPEKCWEMVLQKLQQEIIRHSSLGKQLLPSLECLQGVNGLEMFGFLSPPIIQVIEALDPNHQCLEYWNQKSRAKLFGFDLT
KQDPDNSSIGRGDHSVGEDIKTTLQGFFKKANREELIMMYKVFCSEYTSAEWGVAFTTLTEEIRKTCK

> VvJmjC9 GSVIVT01026208001
MGDDGSSGSPDGGSERKPPRSEEAPENGGGGEEVLRVEGDGGVVPKKRRGRKPGKKAEVKRKGKGSGVGGCSEGGGGPTRKRG
RSKKDVKTGENVDLAAEKGGENGDKGVGSGGSGDEGGGKAGEDVESGENQDMQIAKEGANGREGLQNCGREDEGSEKAGEDVKSGENEDLLAEKSREKDE
KGKSGSGNESSEAQEDLKHVEIEDVLMEKSGEGGEKGKETNSSENVGVLVRRRGRKPKSVILQEIEQNENGIERGVDENGGVTSRRCSLRPRKEVKSVGN
YDLQIEKDEEDGEENVESGVSDDGVAVKKRGKKKWIEEVSLMCHQCQRNDKGRVVRCRKCKRKRFCIPCLETWYPHMSEEAIAESCPFCSGNCNCKACLR
CDGSLKKMAELDYLKMKLSDEEKFKHSRYLLQAVVPFLKQFNQEQMLEKEIEAKIQGLSPSELKIQRVVCNKNERAYCDNCRTSIVDFHRSCPNCSYDLC
LICCREIRDGHLQGGEEESSRRKRKLNFPANASPKDHAKSMSGWEANKNGSIPCPPKNLGGCGQGLLELRCMLEENFVLGLIMEAEEIASSNKLMDISGN
PQQCCSCLNFADDNDTDNSKLRKGASRDDSSDNNLYCPKATDIQDEDLKHFQWHWLRGEPIIVRDVLENTSGLSWEPMVMWRAFRQITNTNHAQHLEVTA
MDCLDWCEVAVNIHQFFKGYSDGRFDSYKWPQILKLKDWPPSTLFKERLPRHHAEFVSCLPFKDYTHPFDGILNLAVKLPKGSLQPDLGPKTYIAYGVAQ
ELGRGDSVTKLHCDMSDAVNVLTHTAEATLPSDNLAEIEKLKAQHSAQDQEEHLEDKVGQDGKGGALWDIFRRQDVPKLQEYLKKHFRQFRHIHCFPLQQ
VVHPIHDQTFYLTLEHKRKLKDEYGIEPWTFVQNLGDAVFIPAGCPHQVRNLKSCIKVAVDFVSPENVGECVRLTEEFRTLPQNHRAKEDKLEVKKMVIH
AVYNALKTLNPQKTVEIMGPEKKVKKTRRKKSRTRG

> VvJmjC10 GSVIVT01012667001
MRSDRRIVVPCTKCKSKLYCIQCIKQWYPNMSEVEIAELCPFCRRNCNCNLCLHSSGIVKTVKTDISDGEKVQHLLYLIKTLF
PYLKQIYEEQTEEIEVEANIQGIPSSGITIPVSSCPSDERVYCNHCATSIVDLHRSCPKCCYELCLSCCKEIRKGNLLRCTAVDFQYPLTEWNANKDGSI
ICAPKEMGGCGGSLLQLKHILPEDRILDLKERAEQVMMKFGTEQARNCSTNGSEMVKRASSREGTDDNYLYCPASHDILKEEEFLNFQRHWAKGEPVIVC
NVLEQTTGLSWEPMVMWRALCENMDSKMSSKMSEVKAEECLSSCQVDISTRQFFKGYTEGRSYDNLWPEMLKLKDWPPSDKFENLLPRHCDEFISALPFQ
EYTDPRAGFLNLAVKLPNTILKPDLGPKTYIAYGIAEELGRGDSVTKLHCDMSDAVNILTHTAEVVLDDNQRLAVKRLKKKHQAQDKRENLVPPCQQEED
LPISRITTQKTGSALWDIFRREDVPKLQDYLRKHSKEFRHVFCSPVNRVVHPIHDQSFYLTLEHKKKLKEEYGIEPWTFEQRIGEAVFIPAGCPHQVRNL
KSCTKVAVDFVSPENIHECIRLTEEFRQLPKNHRVREDKLEIKKMIVYAVAQSLKDFYLLASG

> VvJmjC11 GSVIVT01023517001
MGTELIRACVKEENLDVPPGFESLTSFTLKRVEDNEITTPCLASTSGSESQSIKMETEFDISDAANISRSLRRRPWINYGQFD
NSSDDESDSEHLNQNLPFRRPCLPKGVIRGCLECIHCQKVTARWCPEDACRPDLEEAPVFYPSEEEFEDTLKYIASIRSRAEPYGICRIVPPSSWKPPCP
LKEKNIWEGSKFATRIQRVDKLQNRDSMRKMPRVQNQTRRKRRRFGSCDGETFGFEPGPEFTLDAFQKYADDFRAQYFSKNGNATDLRVENIEGEYWRIV
EKPTEEIEVLYGADLETGDFGSGFPKVSNPVGSTSDERYTKSGWNLNNFPRLPGSVLAFESGDISGVLVPWLYIGMCFSSFCWHVEDHHLYSLNYMHWGA
PKIWYGVPGQDALKLEAAMRKRLPDLFEEQPDLLHKLVTQLSPSIVKFEGVPVYRCVQNPGEFVLTFPRAYHSGFNCGFNCAEAVNVAPVDWLPHGQNAI
ELYREQGRKTSISHDKLLLGAAREAVRANWELNLLKKNTLDNLRWKQVCGKDGILAKTLKARVETEHTRREYLCGSSRALKMEANFDAINERECIVCLFD
LHLSAAGCHCSPDRYACLNHAKQLCSCAWNTKFFLFRYDISELNILVEALEGKLSAVYRWARLDLGLALSSYISKDNLQIPGLIGKLSQSSEGTVLNEQN
SKPVSSLKKVGGAENATALLDLEGRKVPSSRNRMGNQRFQFTKEESVLSAPSLGTPVCHPSQEDMYNTENLASVKSELERNTFPGHGNVILLSDDEGEEL
KKPVLDIAKETPFAKHSEFFERLTDSDAKVNTCNYVKDSVLTTPATNAAVLGERNAISLLHGEMKNCSTSIDSDRNALYLSTTRENSDFNVVNAGSYLQH
PLPHVGGKPNGEDNNDKVGPAAGPKLIDNARTIAGNPSCSQNNLDRYFRQKGPRIAKVVRRINCIVEPLEFGVVISGKLWCNRQAIFPKGFRSRVKYISV
LDPTNMSYYVSEILDAGLAGPLFMVSLEHYPSEVFVHVSAARCWEMVRERVNQEITKQHKLGRMPLPPLQPPGSLDGLEMFGFSSPTIMQAVEAMDRNRV
CTEYWNSRPLIAQHSQLEGSVGNLHRMPEEQNYQYGQSNHPFPVGVDTILRGLFMKANPEELHSLYSILNDNSRPTGDGGLVTRLLSEEIHKRPR

> VvJmjC12 GSVIVT01028351001
MIKYSIKKWGFKEMMRALGNFSQIVNNLHSKTYLPKGVIRGCSDCSNCQKVSARWRPEDACTDILEEAPVFHPTEEEFKDTLK
YIASLRPRAEPYGVCRIVPPPSWQPPCHIKEKNVWTRSKFPTQIQRIDELRDQSRRYETQGFKFEPGPEFTLETFKNYADDFKGQYFCKKDEVADSDVNS
TVSQKQWEPSLENIEGEYRRIVENPTEEIEVLHGADLETGVFGSGFPKVSNQEQMSDHAQYFESGWNLNNTPKLPGSLLAFENYDIFRILQPRLHVGMCF
SSLCWKVEEHHLYSLCYMHLGAPKIWYSIPGRYRPKFEAAVKKYFPYLSATQPELLPKLVTQLSPSTLKSEGIPTYRCIQYPREFVLIFPGAYHSGFDCG
FNCTEAVNFAPVDWLPHGQNTVELYCLQGRRTSISHDKLLFGAAREAVRAQWEVSLLGKSTLDHLRWKELCGKDGILASALKSRIKSEGRRREYLCTSSQ
SRKMDKDFDSVRKRECWTCFYDLHLSAACCQCSPDKYACLNHAKQLCSCSWSAKTFLFRYEMSKLDLLVQALEGKLSSVYRWAREDLGLALSRCVSNDQL
KACGFVNNPSPTESKQEQKCQDEVLKSQDVVEPNGIIENSTNWISEMKTPAVVQALENLKKREHAVAFAISSSGTADDSYSMQKENPYIVPSESTSSSSL
SSSSESDEDISDGFLFRKKQCLFSAYNSNSPVYHLKKEALSSKLPKDDSSEHNIAQRLIPSSRVGHLTDLASEKQITKRPPSCCRSDIILLSDDEGEDPC
RKLC

> VvJmjC13 GSVIVT01007564001
MRGISRAKGSLLRFMKEEIPGVTSPMVYVAMMFSWFAWHVEDHDLHSLNYLHMGAGKTWYGVPREAAVAFEEVVRVHGYGGEI
NPLVTFAVLGEKTTVMSPEVFVSAGIPCCRLVQNPGEFVVTFPRAYHSGFSHGFNCGEAANIATPEWLRVAKDAAIRRASINYPPMVSHFQLLYDLALAL
CSRIPMSISVEPRSSRLKDKKRGEGETVVKELFVQNIMQNNDLLHILGKGSSIVLLPKRSSDISVCPNLRVGSSSRVKPRLSLGLCNLEEAMKTSKSILH
LSHGNDNGSALTSQTQNMETKIESISHGDGLSDQALFSCVTCGILSFACVALIQPREAAARYLMSADCSFFNDWIVGSGPSGVANEDFTGVSGDVHNSEL
NSCSGWMRKRVPNALFDVPIQSANYQIQTVDQNNEVVSNTGTQKNTSALGLLALTYANSSDSEEDQLEPDIPLEADNLASTESNSSEGIFRDPLAISWAT
SKYSPVGHDAERAKFSNAIVPVENTNMSFAPRSDEDYSRIHVFCLEHAVEVEQQLRPIGGVNMLLLCHPDYPKVEAEAKLVAEDLGIDYLWNDFVYRDAT
KEDGEMIQSALDSEECIPGNGDWAVKLGVNLYYSANLSRSPLYIKQMPYNSVIYNVFGRSSANSPTAPDVYGRGPGKQKKIVVAGKWCGKVWMSNQVHPL
LAQKDPEEQEEDRNFHVWVKKPDEKPERKSESSRKAETSSAPRKSGRKRKMMVENGSTKKANRPEREDPTPRRRNSCEQSAREFDSYVEDELEGGPSTRL
RRRNPKPPKELEAKPVVKKQTARKKAKKAPAAKAPGNHNNAKIQDEEEEYQCDMEGCTMSFSSKPELALHKKNICPVKGCGKKFFSHKYLVQHRRVHIDD
RPLKCPWKGCKMTFKWAWARTEHIRVHTGARPYICTEAGCGQTFRFVSDFSRHKRKTGHSAKKARG

> VvJmjC14 GSVIVT01024382001
MVPIHLIRFSSVVVVVAVVAVVLALIDCLKKTGTAIPKFARISSKDDDEPFNKTSMEDTQKVQDAVPSGYPIISDSQLKTGTM
EIFLSEMEREKPTRFSPQQLARFTSDFSMVLGSGGFGVVYKGEFPNGLPVAVKVINSNSEKKVAEQFMAEVASIGRTYHINLVRLYGFCFDPTMRALVYE
YVENGSLDRLLFGENKATNDQWGKFEEIAVGTAKGIAYLHEECRQRIIHYDIKPANVLLDAVFSPKLADFGLAKLCNRDSTQVPVTGFRGTPGYAAPELW
KPYPVTCKCDVYSFGMLLFEMVGRRRNHDASLSETRQWLPRWTWEMFEKNELPEMLSLCGIKETNIEKAGRMCMVAMWCVQYLPEARPTMGKVVKMLEGE
TEIPPPPYPFQRSMPNLVSSSGNSEDTDPSESTRETSEPSYAAPGEGVLEIEIVQGKASKPEKENVLEISNSKMMKSGIVPEVVDNTSMSRHSNNESVYQ
SSGSVIAKRITSKGKVDNFDTTDLEWTDKIPECPVFKPSKEEFEDPLVYLEKISPEASRYGICKIVSPLNASIPAGAVLAKENTGFKFTTRVQPLWLPDW
NVDDKVIFFMRGRNYTLHDFENMANKEFSSKYCCSGSLPSMYLEKEFWHEIASGRKGTVEYAINIDGSAFSCASNDQLGKSKWNLKTLPQLPKSPLRLCE
TSIPGVTDPMLYIGMLFSMFAWHVEDHYLYSINYHHCGAPKTWYGVPGHAAPDFERVVQNHVYTDHILPSTKREDGAFAVLAEKTTMFAPCTLLQHDVPV
YKAVQMPGEFVITFPKAYHAGFSQGFTCGEAVNFAVGDWFPFGAEASQRYSRLCRMPIIPYEELLCKEAMLLHNSQEQGGLAHSSADLASYHCVKVSFIC
LIQSHHHACQFLKKIKGSPSVSPNSQGTILCSLCKRDCYVAYINCNCYSRPICLFHEIEALNCPCGNNPILFLREDVSKMEKIAKKFEQDKGIMREVHRY
KDDKCLQKNETTLKVSKQQVKFQCFL

> VvJmjC15 GSVIVT01031115001
MPDKTVCEKHYIQAKKRAANSALRASLKKAKRKSLGETDVTKKQVRYSPETPPVRSVSIRSSLKPNDDSQRETQFEENRRSYR
TTPLSVMDSSRTKSQRSLDVSAMADYSDGSTDSSDDENGGQTCHQCRRNDRDRVIWCLRCDKRGYCDSCISTWYSDIPLEEIQKICPACRGTCNCKVCLR
GDNLIKVRIREIPVQDKLQYLHSLLSSVLPEVKQIHHEQCAELELDKRLHGASIKLERQRLNNDEQMCCNFCRVPIIDYHRHCMNCSYDLCLNCCQDLRE
ASMLVFHAHQRIMVAVVSHH

> VvJmjC16 GSVIVT01036861001
MYIRQYPAAHYSFSQDSGQLKLELDNPARNVPWCSVNPYPSPETKDAEISQFPLYFNGPKPLECTVKAGEILYLPSMWFHHVK
QTPDSSGRTIAINYWYDMQFDIKYAYFNFLQSISYPSTCNLKLAGTECEDSGSDVCACLSKYAPFTYSSVLRACGRMKDHGRCGRLIHASTIKLGLESDI
YVQCGLVDMYGKCGLLVEARRVFETVSDTNKTNIVCWNAMLTGYIRHGLLMECRYNQAHRYHVQWIGSISTRHSSLCWMSLQHNFHTCHSSLCWMAQVLG
VW

>VvSET1 GSVIVT01025844001
MEQSLGSDSGPADKSRVLNVKPLRCLVPIFPSPPNFSPFSPGQSAPFVCANPSGPFPSGFAPFYPFFSPTDSQRPPEQNSQTP
FGADDNEYSETPNQNAQYLSSFSMHVTDAERTSKAQRSKSKSQKRGRKGQEVNFSSPEVDVELIIIGYILMVYDLLRRRITQIEDGKEATPGVTRRPDLR
SGTILMNKGIRTNIKKRIGLVPGVEVGDIFFFRMEMCLVGLHAPCMAGIDYMGYEDNVEDGDVLIYSGQAGVILPDLTSGAENLPVSLVNDVDDEKGPAY
FTYFPSLRYSKPVNLTEPSFSCNCQGGCLPGNSNCSCIKKNGGYIPYNVAGVLVNNKSLIYECGPCCSCPINCRNRISQAGLKVRLEVFKTKDKGWGLRS
WDPIRAGAFICEYAGEVINDSRFMNHSCSPNVFWQPVLRESNSESYLHIAFFAIRHIPPMTELTYDYGITQSGKADERKKRCLCGSLKCRGHFY

> VvSET2 GSVIVT01001133001
MTSSASKVGESGLVKTVEIEGRGRALVASQSLRGGQIILTDSPILLYSAHPLSSSSNAYCSNCFRHLQTCSTLVSCSSCPCMA
LPSSDSDAPTFLHSLLSSLSPPQGVAGFSVELTTALLAKDKLNAFGLMEPPALAPGGERSVRAYGIYPKASFFNHDCLPNACRFDYVDTASHHNTDITIR
LIHDVPEGSEICLSYFPVNETYADRQKRLLEDYGFTCYCDRCRVEANWKDDDEQEEEQDDEGQKIAGAHWLHCLHRIQMHLLLISWNVTSVETRRSLMKT
STAMKTGSLWMTELFSLFVFTAEDVIFLIPCKKGEEKKKLNFQS

> VvSET3 GSVIVT01003217001
MATLFTLVSSSSSVFFFPIKTLKNPPIIHSRRPPFSLTCLRSLETNPPPPVQTFWKWLFDQGVVSGKTPVKPGIVPEGLGLVA
QRDIARNEAVLEVPKRFWINPDAVAASEIGSVCGGLKPWVSVALFLIREKLRDESPWRSYLDILPEYTNSTIYWSEEELVEIQGTQLSNTTLGVKEYVQS
EFLKVEEEVILPHSQLFPFPVTLDDFLWAFGILRSRAFSRLRGQNLVLIPLADLINHSPSITTEEYAWEIKGAGLFSRDQLFSLRTPVSVKAGEQVLIQY
DLDKSNAELALDYGFIESRPNRNSYTLTLEISESDPFFGDKLDIAESNGLSEIAYFDIVLGQSLPAAMLPYLRLVALGGPDAFLLESIFRNTIWGHLELP
VSRANEELICQVIQDACKSALSGYLTTIEEDEKLKEEGNLHPRLEIAVGVRTGEKKVLQQIDGIFRERERELDQLEYYQERRLKDLGLCGEQGEIIFWES
K

> VvSET4 GSVIVT01001083001
MFFELTSYKYFNGEILFGAGVSVGQQFFSRAEMVVVGLHGRWMSGIDYMGKSYKKQYNNYTFPLAVAVVLSGNYEDNEDDMEE
VVYSGEGGNDILGTKQQIRDQVMERGNLALKNSMEQLVPVRVIRGHKFRDTYPRKVYTYDGLYMINEYWEEKGISGFIVFKYKLDRFGGQPKASSKVVLF
SNKKSSSRAPSAKDELVCKDIAKGQEKLRIPVINEVDNHRGFTYSNSLKVADNVILPPNAAGCNCKGKCTNPMSCSCAERNGSSFPYVLENGNRLLFEPK
DVVFECGPNCGCGPNCLNRTSQQGIKYHLEVFRTKEKGWGVRTLDFIPSGSPVCEYIGELKRTKDINDVFDNDYIFEIDCWQTMHGIGGREKRLKDVQIP
VHNNVDNIDDMPEYCIDARKTGSVSRFVNHSCEPNLFVQCVLSSHHDLELAQVVLFAAENITPSQELTYDYGYILDGVVGPDGNIKELACRCGAASCSKR
LY

> VvSET5 GSVIVT01029980001
MSECGPGCGCGLNCENRVTQRGVSVGLKIVRDEKKGWGLHAAQFIPKGQFVCEYAGELLTTEQARRRQQIYDELSSGGRFSSA
LLVVREHLPSGKACLRMNIDGTRIGNVARFINHSCDGGNLLTVLLRSSGALLPRLCFFASKNIQEDEELTFSYGDIRIREKGLPCFCGSSCCFGVLPSEN
T

> VvSET6 GSVIVT01006875001
MEMSCRSNGNTDDILQSCNIGGTLNQDRGGSGYAPPPFVGGWMYINEQGQMCGPYIQQQLYEGLSTGFLPDELPVYPVVNGNL
INPVPLKYFKQFPDHVATGFAYLSAGISATIRPTNLTAHRQDGTVEFAALDKGYLQSASQPCVSHSVYGFDGQMPNTEAANCSTSNPHLSGEASCWLFED
SEGRKHGPHSYAELYSWHHYGYLSDSSMIYHAENKCGPFTLLSMLNTWRTDRPETNPLSDGENNETGSSLNLMSEIAEEVSSQLHSGIIKASRRALLDEI
ISNIIAEFVASKKAQRLRKLETANQTFNMCSDGRMSEIIGSRKNSVAPGGGTALSDQTCLINETPKESSEKIKSVGGIENFQHTCMVVCRTIFDSCMQVM
WNAVFYAPVAEYCSTWRKRKRWSGHPRIMHPAVEQAMLFRDNVEKSEKLIDEPLQEEHEYSVCEVDCPPGFGLVMTDQDIHIQSSVGLSSSTVEGIPFKE
KRPSDNVQPYDDMQCIVETVQNELQLSAKMMLVECVEAFIEEEVMNLIDSFKDKKLKEGTSDFSIQCPHANEDASSDMVSGLRIESTVAEMILSVDSCTP
QQSPTDFHLPNNASVSVSEHFMSKLNKLCTTDDVVDDQDIDEPPPPGFEYNSRTFVPSQICRFRPSSSDECTPIIGEYVALALCRQRLHEDVLQEWKDLL
VEGTLDQFFASWWTSKQRCDSTGCEEGVSNSNKEKPCDSSAASDQRRERTKDRHSLGSPELSLVIGKYTYYRKKKLVRKKIGSLSHAAASVDSGSQDQLM
EKSRKQDVPGDVSEITEVEMGILKRRKIGLNTCHAEDNSLQAIVQSTLPGDSSSVRIKPNRRSTKCAHVVRNGEVIEDDLACGREEASPFAEDCDFVDKV
VNSNGNGHDVGNLKELAGDCSKKTKSTKVSKKKRKDLKDVPSSRSAKVLKPANGAAKQDTGRQVAVHKSKFSKFKTLNPCLRSVGCARSSINGWDWRNWS
LNASPTERAHVRGIHKAQFACDQYFRSEVVSSQLSNVDATKRGGIARFINHSCEPNCYTKVISVEGEKKIFIYAKRQITAGEEITYNYKFPLEEKKIPCN
CGSKSFRMPLNCLCSCLVFLAVVTVVTSRDGRVAAELLFGYEIW

> VvSET7 GSVIVT01034571001
MQCGMLLLCSRHIKQKENDIAICECKYKANDPDSACGERCWNVLTSIECTPRYCPCSIHCKNQRFQKREYAKTKLFRAEGRGW
GLLATENIKAGEFVMEYCGEVISRTEARGRSQVYVSQGLKDVYIIPLNARECIDATKKGNLARFINHSCQPNCETMKWSVLGEDRVGIFALRNISVGTEL
TYSYNFEWYSGAKVRCLCGATRCSGFLGGKPCGFQEDSFAWEKNNERYSGGDKPSSSLGPKKRLKHDHNGGSRPLPGKQVDAKYVAQFLASKDAQEEVLK
NEEERKEALSHLASVYREIEPAIEDHDMYGPANVLTDVAEQWIGASCRKLKAEFNLHSSIIRNLICPPQRAPEDAKPSAGDPDHEIK

> VvSET8 GSVIVT01034572001
MITTKYYWIFMFMHLHIKQEEDDITICECKYNTNDPDSACGERCLNVLTSIECTPHYCPCSVHCKNQRFQKHEYAKTKLFRTE
GRGWGLLANEDIKAGRFIIEYCGEVISWNEARERSLAYASQGINDAYIISLNARECIDATKSGSQARFINHSCEPNCETRKWSVLGEVRIGIFAMRDISI
GTELTYDYNFQWYGGAKVHCLCGATSCCGFLGAKSRGFQDTDVWEDIDERCSLEDKPSLSMRPESMPKKRSKNNHNGPSRPLNREQVDAKFVAQFLASKE
AQEEILKYEEQREEASSHLHLLYKDEVEPSIKENEIYGIDGVPASVAEKWIRASCMKLKAEFNLHSSIIRNIACTPQRAPDEAQPSEGEPKSDDVVSFGV
ILPCRRAPRHSQAPLFQSTATVYRRTIDWSRKLDQQIEGLPEYIHINRNDFSYRKHIKQQEEDIAICECKYDANDPDSACGEACLNVLTSTECTPGYCRC
GLFCKNQRFQKCEYAKTKLFRTEGRGWGLLADENIKAGRFVIEYCGEVISWKEARGRSQVYASLGLKDAFIISLNGSECIDATKKGSLGRFINHSCQPNC
ETRKWTVLGEVRVGIFAKQDISIGTELAYNYNFEWYGGAKVRCLCGAISCSGFLGAKSRGFQEDTYLWEDGDDRYSVEKIPLYDSAEDEPSSKLPRVMDY
SKPEFISHGKHQLESTELAFPQKNAMIPHIQSNSASQNNHIGPGHVAKKRSKHFPNGRSKPVAQKQVDAKFVAQFLGSEEAREEVFKYEEEKNQASSRLD
SIYDEIRPAIEEHERDSQDSVPTEVARKWIGANCSKMKADFNLYSSIIRNIVCNPRKPQGEAKASEGGDNENETKDLIT

> VvSET9 GSVIVT01032986001
MVVPSLHCMNNTVANGGVDQELTTDNQHSMASGGSSMDNRRKSKSSVKSEKKTLKVESDDTHPVERRSSARIQKLKSEKASSV
YRLEESPNSRPEQCSRKKTKVYQRRKANTHEGSEEVVGEAVSPTEGSSEAMDSDCVENGESTNPVLMEVAANGIVNDVSTTNVVEKSVYAKVKETLRTFN
KYYLHFVQKGDAPEKDGRQGSRRPDLKAISKMMETNAILYPEKRFGPIPGVDVGHQFFSRAEMVAVGFHSHWLNGIDYMGQSYNRREYSGYTFPLAVAIV
LSGQYEDDLDNSEDVVYTGQGGNNLLGNKRQVQDQVMERGNLALKNCMEQCVPVRVIRGHKSANSYVGKVYTYDGLYKVVQYWAEKGVSGFTVFKYRLKR
LEGQPILTTNQVQYARGRVPNSISEIRGLVCEDISGGQEDIPIPATNLVDDPPFAPTGFTYCNSIKVSKSVKLPSNAIGCNCKGTCTDPRTCSCAMLNGS
DFPYVHRDGGRLIEAKDVVFECGPKCGCGPGCLNRTSQRGLKYRLEVFRTPKKGWAVRSWDYIPSGAPICEYKGILMRTDELDNVSDNNYIFDIDCLQTM
RGLDGRERRFRDVSMPTSTDDQKSESVPEFCIDAGSTGNVARFINHSCEPNLFVQCVLSSHHDAKLARVMLFAADNIPPLQELTYDYGYTLDSVMGPDGK
IKQMLCFCGAADCRKRLL

> VvSET10 GSVIVT01027118001
MIIKRTMKIEMPQIKRCKLEQPGDDVASLIKPKKRRIDGNGPADTPGNVEEDSIVAGSLCTEISYCASEVESNSKGKRKGRNP
KAEGSRPPLLPSSRGRHRALPSRFNDSIIDSWTKEDSKADDMESNLDDFEVVVYEKERIGEAGYVGFRESESKKYSCSHSSLSSLHDGLNPLVEASDYPG
FNSKGREKAGKDKTEKRKDFYRPEEFVLGDIVWAKSGKRYPAWPAIVIDPVFEAPEAVLSSCVADAICVMFFGYSKNGKQRDYAWVKHGMIFPFLEYLDR
FQGQTQLHKSKPSDFREAIEEAFLAENGFFDTNNGSGQLSRTEENPIFPCSYIQGVFNNGEAQPCDGCGCVLPCKSSKKMNNSTGETQLLCKHCAKLRKS
KQFCGVCKKTWHHSDGGNWVCCDGCNVWVHAECEKISTKRLKDLEDIDYYCPDCKAKFNFELSDSDKWQPKVKCIENNGPPVLPDKLAVVCTGMEGIYLP
NLHVVVCKCGSCGTRKQTLSEWERHTGSRAKKWKASVKVKDSLIPLEKWLAEYTTHGINPLKLQKQQLFSFLKEKYEPVHAKWTTERCAICRWVEDWDYN
KMIICNRCQIAVHQECYGARNVKDFTSWVCRACETPDAKRECCLCPVKGGALKPTDVEGLWVHVTCAWFRPEVAFLNDEKMEPAVGILRIPSTSFLKVCV
ICKQTHGSCTQCCKCATYFHAMCASRAGYSMELHCGEKNGRQITKKLSYCAVHRAPNADTVLVVRTPSGVFSARNRQNQKRDQSFRGSRLVSSRRPELPV
SLALETNELEPLSAGRCRVFKRSINNVGAGAIFHRLMGPRHHSLDAIDGLSLYKELEDPQTFSSFKERLYHLQRTENHRVCFGKSGIHGWGLFARRSIQE
GEMVIEYRGEQVRRSVADLREAKYRLEGKDCYLFKISEEVVIDATNKGNIARLINHSCFPNCYARIMSVGDEESRIVLIAKINVSAGDELTYDYLFDPDE
RDESKVPCLCGAPNCRKFMN

> VvSET11 GSVIVT01008928001
MEDLQADLSANGLTVSTTPEKGRCLLSIKDFSPGEVIISQEPYVSVPNNSAVHSRCEGCFRSSNLKKCSACHVVWYCGSTCQK
SDWKLHRLECNALSRLEKERQKSLTPSIRLMVKLYMRRKLQSEKIMPTTARDNYNLVEALVSHITDIDEKQLVLYAQMANLVNLILQWPDINVKEIAENF
SKLACNAHTICDGELRPLGTGLYPVISIINHSCLPNSVLVFEERLAVVRAVQHIPKGTEVLISYIETAGSTITRQKALKEQYLFTCTCPRCRRMGQYDDI
QESAILEGYRCKDDRCDGFLLRDSDDIGFICQQCGLVRNKEEIKRLASELKPLSDKATMSSSSHYVEATSIYKMIEKLQTKLFHPFSINLMRTREAILKI
LMEMKDWRAALTYCKLTIPVYQRVYPGFHPLLGLQYYTCGKLEWLLGETEDAVKSLTKAADILQITHGTNTPFMKELLFKLEEARAEASHKRSSKDK

> VvSET12 GSVIVT01023397001
MYCGNRIVQRGITFKLQVFMTHEGKGWGLRTLEALPKGAFVCEYVGEILTNMELYERNKQSNGNDRHTYPVLLDADWGSEGVL
KDEEALCLDATFYGNVARFINHRCFDANLLEIPVEIESPDHHYYHLAFFTKRKVDALEELTWDYAIDFADENHPIKAFQCCCGSEFCRDMNGKGTSILSC
SLKQLSIFFR

> VvSET13 GSVIVT01013555001
MAFPLKEEEEEVDSGTGTPVRYLPLRHVYSTSAPCVSASGSSNVVTKKVKARRMIADGFDGEGDGVDQKPYPAKPPVVHVYAR
RRKRPRNLTAERPESGALVAVKEERCESDGCEGVGGGDRGVGVLGKKRRSANLEVKNLGDNSRGVGSSVRRRLREARKDSTVDLPHRRKRKSSENLTKVD
SNSACIKRWLWLNFDDVDPEKFIGLQCKVYWPLDGEWYRGCIIGYDLEANRHQVKYNDGDKEELILSSEKIKFYVSREDMQHLNLSLSVRSLDSDDIDYD
EMVVLAASWNDCQDHEPGDIIWAKLTGHAMWPAIVVDESIIHNRKGLNKISKEKSLPVQFFGSHDFARVKTKQVTPFLKGLLSSFHLKCTKPHFHQSLVE
SKAYLSEQKLSKRMLRMQKLTEDDDCESMSGEDEKRTDSGDDCIGDERVKRKLDDLIKFPFEIGDLQVIRLGKIVKDSDRFQVEGFICPEGYTAMRKFTS
ITDPSLCALYKMEVLRDAESKIQPLFRVTLDNGEQFQGSTPSSCWNKIFRRIRKMQNSASDGSSAEGGAEKLNESGFDMFGFSNPEIFRLVQELSTSKIS
SKFSMSKSISRRYQDLSSGYRPVRVDWKDLDKCSVCHMDEEYENNLFLQCDKCRMMVHARCYGELEPVDGVLWLCKLCGPGAPDSPPPCCLCPVTGGAMK
PTTDGRWAHLACAIWIPETCLSDIKTMEPIDGLSRINKDRWKLLCSICGVSYGACIQCSNSTCRVAYHPLCARAAGLCVELEDEDRLHLISVEDDEDDQC
IRLLSFCKKHRQPSNERTAFDERIGQVARECSNYNPPSNPSGCARTEPYNHFGRRGRKEPEALAAASLKRLFVDNRPYLVGGYCSKFSFRNQKIKASQLD
APKSILSMVEKYNYMRETFRKRLAFGKSGIHGFGIFAKQPHRAGDMVIEYTGELVRPSIADRRERLIYNSLVGAGTYMFRIDDERVIDATRAGSIAHLIN
HSCEPNCYSRVISFNGDDHIIIFAKRDIKRWEELTYDYRFFSIDEQLACYCGFPRCRGVVNDIDAEERMAKRYAPRSELIGWIGE

> VvSET14 GSVIVT01026529001
MAPATSSPAAAQKLFPFKGSVRRTEAPRRPSLPARKYKSMAEIMATAKYLVIERADYSDVRCVQCGSGDHDDELLLCDKCDRG
FHMSCLRPIVVRIPIGTWLCPSCSGQRRGRSLSQTKIIDFFGIQKRSDGMGEFASTQDGRKRRRKSASLVLHKKKRRLVSFTPTEDHAQRLKQMGSLASA
LTALHMEFTDELTYMPGMAPRSANQAKFENGGMQVLSKEDIETLEHCRAMSKRGEGPPLIVAFDSFEGYTVEADGLIKDMTFIAEYTGDVDYIRNREHDD
CDSMMTLLLATDPSKSLVICPDKRGNIARFINGINNHTLDGKKKQNLKCVRYSVNGECRVLLVATRDIAKGERLYYDYNGYEHEYPTHHFV

> VvSET15 GSVIVT01019046001
MEVLPCSGVQYVGESDCPQQSPGTTFIYDGDSNCVEHGQQVQLADDKMDKLLLNAERSQKEKKGEVEGRVEELPTSEGHCSGA
LYFDCEVEDQKQPCNSLYFEDGNLNVQNGCTEPCLASDSSHLIVDTIESELPSNTGEGELSVSEPKWLEQDETVALWVKWRGKWQAGIRCSRADWPLSTL
KAKPTHDRKKYVVIFFPHTRIYSWADILLVCPINKFPQPIAHKTHNVGLEMVKDLTIARRFIMQKLAVGMLHISDQLHIEALTENVRNVMSWKEFAMEAS
RCKGYSDLGRMLPRLQSMILMNYISPDWVQHSFRSWVERCHSADSAESVEILKEELFGSILWNEVSSLWDAPVQPELGSEWKTWKHEVMKWFSTSHPISS
SGDIKQQSGDNPLTSSLQINRKRPKLEVRRAETHASVVETGGLHQAVTVDIDSGFFDSRDIVHDAPSASEPYKEEVFGEGAVTTNSPGSATDRWNEIVVV
AKKSLDPGNKNRQCIAFIEAKGRQCVRWANDGDVYCCVHLASRFVGNSAKADVAPPVDMPMCEGTTTLGTRCKHRSLYGSSFCKKHRPQSDTKRTLTSPE
NKLKRKHEENISISETTLCKDIILVGEVENPLQRKHNLIENPEYSSKGYMNAEVLHCIGSRPEDGGDPCLESPKRHSLYCEKHLPSWLKRARNGKSRIIS
KEVFIDLLRNCCSQEQKLHLHQACELFYRLFKSILSLRNPVPREVQLQWALSEASKESGVGEFLTKLVCSEKDKLMRLWGFNADTDVQVSSSEFPDDQAI
GKHWMDNHKKESQWLFRGYACAICLDSFTNRKVLESHVQDRHHVQFVEQCMLFQCIPCGSHFGNTEALWLHVVSVHPVDFRLSTVTQQHNNHTEGQGGFR
KFICRFCGLKFDLLPDLGRHHQAAHMGPNLVSSRPGKKGVRYYAYRLKSGRLSQTHPGIYFNQFWGTKSTFSSLLEGKYGVLPERLYLKAAKLCSEHNIQ
VSWHQDGFVCPNGCKPVSNAHLPSLLMPHSNGSIGHGSASLDPVSEEWEMDECHYVIDSHGSDGQITRYSMPWESFTYVTKPLLDQSLGLDAESWQLGCA
CLHSTCSPERCDHVYLFDNDYSDAKDIYGKPMSGRFPYDEKGRIILEEGYLVYECNGKCSCNRTCQNRVLQNGVRVKLEVFRTEEKGWAVRAGEAILRGT
FICEYIGEVLSEQEADKRGNNRHGEEGCSYFYDIDSHINDMSRLVEGQVPYVIDATRYGNVSRFINHSCSPNLINHQVLVESMDCQLAHIGLFANRDISL
GEELTYDYRYKPLPGEGYPCHCGASKCRGRLH

> VvSET16 GSVIVT01035924001
MGGMNGKPSMLFTTRFHKDHIVQKEKNISFHQNEKSKGQNHKKIDCHASQWKDVPSKVIVSCDMKCVRPSVDGLGGRKNDEDQ
PAMYGRKNDEDQLADTAAKRFNGNLQEINCLKEQEMSNISSGCSAPAVTQASIEVNNMDSCTVDAGDTGCANDLVVDEASGIEKCWSSDDALDSERSAEF
LGFTCKTSFIKEGSSKALANQSSRSLIDELKFRDSFRWKRVRNESHTGLAIHEKNSHSPKIERGLKTRKRKKTMKMKMLNASFPASGFSSGHYEHTECAG
SAEWRSFSYKDVDTLLQCELGTSHTCGACTIGPSFKRRRSTLSSAKNFSRKRDVDKIYADREGEDGYQAQSKGKTEFLSIHEVSGAKRIGPDRTAEAFRQ
FCMQEPSHTKAVKYNSVGCVKESSCLKLDVSNRREKPVVCGKYGVISNGKLAIDVPKPAKIFSLSRVLKTARRCTLSANDEPRLTSMRQLKKARLRGSNG
CVNEISNLMKEKENEIQNATRCDERNPDNSMEEAEKAVISGDTRCADELLMSKQEKAYGSKKDDSYHSTRLKRKYKEIRKRSLYELTGKGKSPSSGNAFV
KIPKHAPQKKSGSVGLENAEDSKHSMSESYKVNSKKSIKEHRFESFISDTDAFCCVCGSSNKDEINCLLECSRCLIRVHQACYGVSRVPKGRWYCRPCRT
SSKNIVCVLCGYGGGAMTRALRTRNIVKSLLKVWNIETESWPKSSVPPEALQDKLGTLDSSRSGLENESFPIHNTITAGILDSTVKQWVHMVCGLWTPGT
RCPNVDTMSAFDVSGASRPRANVICSICNRPGGSCIKCRVLNCLVPFHPWCAHRKGLLQSEVEGVDNENVGFYGRCMLHAAHPSCELDSDPINIETDSTG
EKELTCARTEGYKGRKQEGFRHNLNFQSNGNGGCLVPQEQLNAWLHINGQKSCTKGLPKTPISDVEYDCRKEFARYKQAKGWKHLVVYKSGIHALGLYTS
RFISRGAMVVEYVGEIVGLRVADKRESDYQSGRKLQYKTACYFFRIDKEHIIDATRKGGIARFVNHSCLPNCVAKVISVRNEKKVVFFAERDINPGEEIT
YDYHFNHEDEGKKIPCFCNSRNCRRYLN

> VvSET17 GSVIVT01008805001
MESEVLGAEDPLSNCTGPSEQKGGEGRVRDAVEGRSDHVVDGIKVDIWSQILPSQEGEMQLEFLGMDGFLSDCSELNDQMGAK
LAGDVKDKGGVLGGISVGGFEQASPSQGCETPLELLLTGGSLSDCAEHNECKADMSVNGSCGEVREVVEEKIDGLGGINEQLLPSQGCEMPMELLVSGGS
LGNCGEDSENKVYLSVNGSCVEEVKEVVEEKNEMLGGINEQMLPSQDCEMPLELLVTGGLLCSCDADNECKADTSIEGSYGEEAREVVKEKSEIFDGTDE
QTLPSQGCEMLTGGSLSSCGKDNECKVNTSINGSCWEVVEGKNDALGGINEPILPSQRVETPLESLVAGGSLSTCVKNNDYKVEMSINGSSGKDVQEAVE
ERSDILDGIDEQILPSQGCEMPLESLIKGSLSNCAKDDECKVNLSINASCCKQIREVVEEKSDILRMINEQILPSQGCGRPLESPSNFAEQNKHKDSGVA
GGPSEFVDDILAGSQNNKIRQILPSQDCKIPLEHLSVASSPTDCAEGNVQKVTAGFDGSSAETVTEVVEEKSDIFLGMKGEMCSQISPIEENMYDLRERS
SSMAPDYTLEKSDSPPPCCYSGVVDNGSSEIFAEPGYSGADVLIDAFNSTDADSSGNIGGEEKVDVRWDCVSETKCPEIICLPPRRSARARKSSQKTQTA
NVARKGWKTANKKPHSHGIFEIFLKVVRKKRSSFCKPARASIWGSLENITQVFYHNSDLDCGRVQNQGSRKTKGGRGCGKRNKSRAVGNSQGSKVKGRAS
TSHIRLKVKMGKRVSQSGSKDIVPDVVDTSDPVQTMFSDNGSELCWAMGSELQKFTVGIETQLVEEIPGTGQHLTSHGNLEKEKTSPIDSALDEVHFTDK
DQETIVIPDNSDRNAATNYLSISSKTEVEALEGAIDNGYLDPGTSPDSEVINLIPDGQVGARVQEDLHDVVQASSKDSVAAADVTSSNVPLLDGSSESQN
SKKLLPSTKAKGHKLPKSSKSGRASKSRSQFLDSGRNQRRNACRQKESQQKSARKNVNEEGVCNHVCKVESHQEIAYAVENHVVDDIGEIVTAEKTVSKD
MSNLDMIQNEVVRQYLPPRIAWVRCDDCYKWRRIAAALADSIEETNCKWICKDNMDKAFADCSIPQEKSNGEINAELEISDASCEEDVYDAHLTSKEFGQ
RRSTVTQSSSWMLIRSNLFLHRSRRTQTIDEVMVCHCKRPVEGRFGCGDECLNRMLNIECVQGTCPCGDLCSNQQFQKRGYAKLKWFKCGKKGYGLQLQQ
DISQGQFLIEYVGEVLDLQTYEARQKEYASRGHKHFYFMTLNGSEVIDACAKGNLGRFINHSCDPNCRTEKWMVNGEICIGLFALRDIKKGEEVTFDYNY
VRVFGAAAKKCVCGSPQCRGYIGGDPLSTEVIVQGDSDEEYPEPVMVNEDGETADSFDNTISTTSSFDAAEIQTSSDSADANVSKSETPEEKQVCSKSRL
LMKASRSSSSVKRGKSNSNPVNANKPPGIGNKTQVLSNKPKKLLDGSANARFEAVQEKLNELLDANGGISKRKDSSKGYLKLLLLTVASGDNGNREAIQS
TRDLSMILDALLKTKSRVVLVDILNKNGLRMLHNIMKQYSREFIKIPVLRKLLKVLEYLALRGILTLEHINGGPPCPGMESFRDSMLTLTEHNDKQVHQI
ARSFRDRWIPRPVRKISCMDRDDGRMEFHRGSNCSRFSSQHNYWREQEESSAPGFGGSATNGTNTRKRKSRWDQPIEAHPDPRFHPHKEQKEEEDERQNL
HEDVPPGFAYPLNTPLTFEVAGGHPQKRFNSCLPVSYGIPLSIVQQFGTPQGETMQSWVVAPGMPFHPFPPLPPYPRDRRDPPSRTVNPITRNQPGEEQQ
NCHGSASCHTDQSTPSTSGASPPDVNVPCANNQHVFKRVKNNSYDLGRKYFRQQKWNNSKGIYDIFSSCNLLSWK

> VvSET18 GSVIVT01012615001
MKEDLEMGEELMQQLRSRATELLLREEWNESVQAYSHFISLCQHHISRIHQHADPDHLFKLQKSLCLALSNRAEARSRLRDLA
NALQDCDGALEIEGTHFKTLLCKALEGYLERCKKLEHQSRTGAFDLSDWVVNGFRGKFPELAEYIGAVQIMKSEISGRGLFATKNVDAGTLVLVTKAIAT
ERCILPEQNDDSADNIQLVMWKNFIDKVVESASKCKRLHHLISVLSNGEDEDVLEVPDVNLFRPETEESGLSMGKLDMGKILSILDVNSLVEDATSAKVL
GKNSDYYGVGLWILPAFINHSCNPNARRLHVGDNVIVHTSRDVKAGEEITFAYFDVLSPWRKRKDMAKTWGFQCNCKRCKFEEQICSKMEIQEIQMGLER
GLDMGDAIYRLEEGMRRWTVRGKEKGYLRASFWAAYSEAYESEKTVRRWGRRIPAVEAVVDSVLEAVGSDERVLKAFMAGLKRSGGGGVVEIERAMKLAR
GVYGKVVKKQAMRTLISLGY

> VvSET19 GSVIVT01008928001
MEDLQADLSANGLTVSTTPEKGRCLLSIKDFSPGEVIISQEPYVSVPNNSAVHSRCEGCFRSSNLKKCSACHVVWYCGSTCQK
SDWKLHRLECNALSRLEKERQKSLTPSIRLMVKLYMRRKLQSEKIMPTTARDNYNLVEALVSHITDIDEKQLVLYAQMANLVNLILQWPDINVKEIAENF
SKLACNAHTICDGELRPLGTGLYPVISIINHSCLPNSVLVFEERLAVVRAVQHIPKGTEVLISYIETAGSTITRQKALKEQYLFTCTCPRCRRMGQYDDI
QESAILEGYRCKDDRCDGFLLRDSDDIGFICQQCGLVRNKEEIKRLASELKPLSDKATMSSSSHYVEATSIYKMIEKLQTKLFHPFSINLMRTREAILKI
LMEMKDWRAALTYCKLTIPVYQRVYPGFHPLLGLQYYTCGKLEWLLGETEDAVKSLTKAADILQITHGTNTPFMKELLFKLEEARAEASHKRSSKDK

> VvSET20 GSVIVT01028411001
MARNPRVLAAYRAMRALGIAEATVKPVLKNLLRLYEKNWELIEEENYRALADAIFEYEETKQDNILGGETQLHDEPARPLKRL
RLRNQESQDAEQPQTLAERQPQGIAETPEPSVGNIRPELHPVSSPQAHLRAESDLLHTQQRLRDKGKEPLSPQIAAKEKRSIPVRSFHLNAEPGIILSPK
QKVHDTPALMKPKDEPFTDDILQLEVPIAVIHPDPLHKGNLPENYSTGKLDGPQPPVNSRVDGEDEVNGGPASSSGAGTNCELANISNLEIASSPLGEVK
ISLSCNSALGKPDFRTHTEESHEGSINTTPTGDLLGKSTAPDAVGSCGDEENFSMSSCITNGSFKIQCSTEETNGPNNANSLSLVVVQQRQLTPDDIRFI
HDVDDITKGEEKVRIPLVNETNSEFPTPFHYISQNLVFQNAYMNLSLARIGIENCCSTCFGDCLSSSTPCACACESGGDFAYTLEGLVKEDFLEECISRN
RDPQQHQLAFCQECPLERSKAEDILEPCKGHIVRKFIKECWSKCGCSKQCRNRLVQRGITCNFQVFLTPDGKGWGLRTLEDLPKGSFVCEYVGEILTTVE
LYERNMQSTSRGKQTYPVLLDADWALRGILKDEEALCLDATFYGNVARFINHRCLDANLVEIPVEVESPDHHYYHLALFTTRKVNALEELTWDYGIDFDD
QDHPVKTFRCCCGSKFCRNMKRTRSRSALSLG

> VvSET21 GSVIVT01028124001
MFPLMRLRTILEMNCEWKPFEKELYLKGIEIYGRNSCLIARNLLSGLKTCIEVSSYMYDDGSAMLHRSAVVPSSFLEDNGRGD
ADYTEQEMPTRSRLFRRRGRTRKLKYSWKSAGHPSIWKRIADGKNQSCKQYTPCGCLSMCGKECPCQSNGTCCEKYCGCSKSCKNRFRGCHCAKSQCRSR
QCPCFAAGRECDPDVCRNCWVSCGDGSLGEPPKRGDGQCGNMRLLLRQQQRILLAKSDVAGWGAFLKNSVNKNDYLGEYTGELISHREADKRGKIYDRAN
SSFLFDLNDQYVLDAYRKGDKLKFANHSSNPNCYAKVMLVAGDHRVGIFAKEHIEAGEELFYDYRYGPDQAPAWARKPEASKRDDSAVSQGRAKKHQSH

> VvSET22 GSVIVT01030624001
MEGICPIDSQFSHEISALLKPPPAHQLQEYFDNLIRTRQYLGLKVKHDGEFGKGVYADSDFGEGELVLKDQMLVGAQHSSNKI
NCLVCGFCFRFIGSIELQIGRRLYLQGLGVSTNHDELGECASSSSKDKVPLPKGVVESLMNGELALPYPKEFPLPSAIACSGGCGEAYYCSKLCAEADWE
SSHSLLCTGEKSESICREALSKFIQHANETNDIFLLAAKVICFTILRYKKLKKAHLKEQEKYTSAIVLKNGDLPLLLEAWKPISMGFKKRWWDCIALPDD
VHSCDEAAFRAQIKELAFTSLKLLKEAIFCKGCEPLFSLEIYGHIIGMFELNNLDLVVASPVEDYFLYIDDLPYPQKKKAEEITRQFLDALGDDYSVPCQ
GTAFFPLQSCMNHSCYPNAKAFKREEDRDGQATIIALRPIFKEEEVTISYIDEDLPFDERQALLADYGFRCKCPKCLEEEP

> VvSET23 GSVIVT01013881001
MHPQLERNLLQLDPWCQTFGNKAGRGKSVLRSIGKEVEICSKDQSSMDHQTIVNNSRVQGALNLFQELLEKLTHEASLTMKKV
IISKLHVEAAMTLKRQQKWVNTTKRLGHVPGIEVGDTFRYRVELCIIGLHSHFQNGIDYMEKDGKILAISIVDSGRYANDKESSDILIYSGQGGNPMVGH
KQAEDQKLERGNLALKNSMDAKTPVRVTRGFQATKVTSQGYTYDGLYFVDKYWQEIGQFGTLIFKYQLKRIRGQPKCNLREFNESKKSKVRWKITFNDIS
RGRELNKPKKSKVRMKTILNDISQGKEERPIRVVNTIDDEKPQPFSYIARMVYLESSNWSIPSGCDCTDGCSDSVKCACVLKNGGEIPFNCSGAIIEAKP
WIYECGPLCKCPPSCNNRVSQNGIRFPLEVFKTKSTGWGVRSRNYIPSGSFICEYAGELIQDKEAEQRVGNDEYLFDLAKDYGAFAIDAAKFANVGRFFN
HSCSPNLYAQNVLYDHDDKRMPHIMLFATKNIPPMRELTYDYNYMVGQVRDINGKIKKKRCYCGSRECTGRMY

> VvSET24 GSVIVT01013854001
MTLKRQQKWVNTTKRLGHVSGIEVGDTFHYRVELAIIGLHSHFQNGIDYMEKDGKVLAISVVDSGRYANDKESSDVLIYLGQG
GNPMVGYNKQPEDQKLERGNLALKNSMDAKTPVRVTRGFQAMKVTSNGYTYDGLYFVDKYWQERGQFGKLVFKFQLKRITGEPKFDQRELNQKLKKSKKS
KVCRKNILNDISLGKEERSIHVVNTIDYEKPQPFTYIARMAYLEGSKWSIPSGCDCTDGCSDSVKCACVLKNGGEIPFNCHGAIIETKPWVYECGPLCKC
PPSCNNRVSQNGIRFSLEVFKTKSTGWGVRSRNYISSGSFICEYAGELIQDKEAKRRTANDEYLFDLDNGAFAIDAAKFGNVGRYINHSCSPNLYAQKVL
YDHDDKRLPHIMLFATKNIPPMRELTYHYNYMVGQVLDINGQIKTKRCYCGSQECKGRMC

> VvSET25 GSVIVT01038708001
MIIKRNLKSKMPSMKRCRLGHSAADDDESPAAKKKRKMNGYFPLNLLGDVAAGIIPLSGYGLQRIFGGHVGDDGDGVGAMNRA
AQVHRPPLVRTSRGRVQVLPSRFNDSILDNWRKESKPNAREIILDEDFEPEKEKPCSKTPKQSKYSSSRSSLTSLHEQLAEVERYPTDEVEEKFGLGRVD
RESKGGSRLEEFISGDIVWAKSGKKDPFWPAIVIDPTSQAPGQVLSSCIAGAVCVMFFGYSGNGSRDYGWIKRGMIFSFIDNVERFQGQSDLNDCKPSDF
RTAIEEAFLAENGFIEKLTEDINVASGKPNYLESTRGIQEATGSNQDQECDSQDQASGDVFRKKDTWSCDGCGLRIPLKSTKKMKVLTPKGRFLCKTCDR
LLKSKQYCGICKKMQNQSDSGTWVRCDGCKVWVHAECGKISSKLFKNLGATDYYCPACKAKFNFELSDSERWQPKVKCNKNNSQLVLPNKVTVTCSGVEG
IYFPSIHLVVCKCGSCGMEKQSLTEWERHTGSKGKNWKTSVRVKGSMLSLEQWMLQVAEYHDNSFLAVNPPKRPSIRERRQKLLTFLQEKYEPVHARWTT
ERCAVCRWVEDWDYNKIIICNRCQIAVHQECYGARNVRDFTSWVCRACETPDVERECCLCPVKGGALKPTDIETLWVHVTCAWFQPEVSFSSDEKMEPAV
GILSIPSNSFIKICVICKQIHGSCTQCCKCSTYYHAMCASRAGYRMELHSLVKNGRQITKMVSYCAYHRAPNPDTVLIIQTPLGVFSTKSLIQNKKKSGS
RLISSNRIELQQIPTVETDEFEPFSAARCRIFRRSKSNTKRTVEEAIAHQVKGPFHHSLSAIESLNIFREVEEPKNFSTFRERLYHLQRTENDRVCFGRS
GIHGWGLFARQAIQEGDMVLEYRGEQVRRSIADMREVRYRLEGKDCYLFKISEEVVVDATDKGNIARLINHSCAPNCYARIMSVGDDESRIVLIAKTNVA
AGDELTYDYLFDPDEPDECKVPCLCKAPNCRKFMN

> VvSET26 GSVIVT01038589001
MAPNSLSREPKKKARETLSLALTLKYRPPTLHTQSHTSLSFSLPTAMASSQTRTLRRRRTQASKPISQSPLKSLSKSQIDDYY
SDAVCEECGSGDAADELLLCDKCDRGFHLFCLRPIIVSVPKGPWFCPSCSSQKKLKYFPLVQTKIVDFFRIQRSTESTQKLNYDSQKRRKRSSSLVVSKK
KRKLLPFNPTEDPLRRMEQMASLATALTATRTEFSNELTYIPGMAPKSANRAVLEHGGMQVLSKEDTETLNLCKSMMGRGEWPPLMVVFDPKEGFTVEAD
RFIKDLTIITEYVGDVDYLKNRENDEGDSMMTLISANEPLRSLVICPDKRGNIARFINGINNHMPDGKKKQNVKCVRFEVNGECRVLLIASRDIPKGERL
YYDYNGYENEYPTQHFV

> VvSET27 GSVIVT01023699001
MPDLGNLLNSSSLTLSRCHNLKPLSDSCDSGGPLSPNSVNWEQRLRFPLSPKDEIECEKSGVGIRVLKRTRGGSLDRSKKLSN
GKSLDDHVKAWADKKMESGATKSQCSLPFMSGASRLDECLVCHSFIYPGEEVSCTIDGCQGVYHLKCAKNELGFSTKRKFKCPQHACFVCKRRSYLQCVR
CIIASHQKCAPWPEEMTLLRNRSGQAVCWRHPTDWRLDKKHAAPTSDIEEIFSRLPLPYDEEEFKIDLTWKDTVENKMEPTPYVHIRRNVYLVKKKRDDA
ADGIGCTNCSSVCSENCVCRVQCISCSKSCHCSENCTNRPFRKEKKIKIVKTELCGWGVDAAESINKGDFVIEYIGEVIDDALCERRLWDMKDRGDQNFY
MCEIRKDFTIDATFKGNASRFLNHSCDPNCKLEKWQVEGETRVGVFAARSIKAGEPLTYDYRFVRFGPEVKCHCGAPSCHGYLGTKKKIAKVELCWGSKR
KRSSYFALITI

> VvSET28 GSVIVT01022097001
MSSKPSLSASASASGSRSEHSKDPTTNKLPETSPIPRDISSVIDSLKKQALADRCVSIKERMEENRQRLVGITKHLHKLSEER
RNNRNSDIDKSVDLLTKRQRDALDMQNGNDANNGDKASHCSQEDGHVSSAVLLGSSIAVKNAVRPIKLTEVKRLPPYTTWIFLDRNQRMTEDQSVVGRRR
IYYDQTGGEALICSDSEEEAIEEEEEKKEFADFEDYILRMTIKETGLSDPVLEALGRYLSRKPCEVKARYEILNKGEKSVVGSKNGVIEDISQTLTSYLD
KDLDAALDSFDNLFCRRCLVFDCRLHGCSQDLVSPAEKQLPWNHLDEDNIPCGAHCYRLAVKSESIGMVSSPVCADFEDKTAPSSDGAGPHLSSRKNCGP
SSKRRAKSCQSESASSNGKNISESSDSEIRPKQDTTSTHHSSSPPKTRLVGKCAIRKRNSKRVAERVLVCMRKRQKMVASDSDSILSGRLWPRDMKLRSN
SRKENEDASSSSLKKVKPSITGRSRRKCSPVQDSNKLVEGEVPEGQMNEMINDPPASSSDDTLRKEEFVDESMCKQERSDDKSWKAIEKGFFEKGVEIFG
RNSCLIARNLLNGMKTCLEVFQFMNCSENKPFFRAGDGSNSMVEGYSKVDFNETMGNEVRRRSRFLRRRGRVRRLKYTWKSAGYHSIRKRISERKDQPCR
QYNPCGCQSACGKQCACLLNGTCCEKYCGCPKSCKNRFRGCHCAKSQCRSRQCPCFAADRECDPDVCRNCWISCGDGTLGVPSQRGDNYECRNMKLLLKQ
QQRVLLGRSDVSGWGAFLKNSVGKHEYLGEYTGELISHREADKRGKIYDRENSSFLFNLNDQFVLDAYRKGDKLKFANHSPDPNCYAKVIMVAGDHRVGI
FAKERISAGEELFYDYRYEPDRAPAWARKPEASGVKKEDVAPSSGRAKKLA

> VvSET29 GSVIVT01024266001
MHISLIWLISKRKRRKSVTSIYKLKHLFLSALSLSLVSLVCFALCTLLHLSDLPNPKSAPLLSPRFKIILLSIFCGAKLIEAM
PSAKKLKQNIEQKGVGGAFSKLLKELGNPVDFELPSSFNKWKPTSYTFIRRNIYLTKKIKRRLEDDGIFCSCSSGSGSSGVCGRDCLCGMLQSSCSSGCK
CGTSCLNKPFQSRPVKKMKMVETEKCGSGIVADEDIKQGEFVIEYVGEVIDDKTCEDRLWKMKHLGETNFYLCEINRDMVIDATYKGNKSRYINHSCDPN
TEMQKWRIDGETRIGIFATRDIKRGEHLTYDYQFVQFGADQDCHCGAVGCRRKLGVKPSKPKLASSDAALKLVACQVYQNGGLHIGSSRLTHDQQRIGSR
NCIGEIIMVTRSMGERSFGIIKRFDNNSKKHLIMFEDGDGEFLDMSKEDWEFVTL

> VvSET30 GSVIVT01027942001
MGSLIPFQDLNLLPDPATSPSTAATAITPALIFPKIEPKLEPFDAPTLPLQSFPQNPSPNFFNNVYSEYYRISELFRTAFSKR
MENLGNIEVLDPDSRAIVPVPEETRISNVVVSRRRDQRSSELVRVTDLTIDHVRYFRDLVRRTRMLYDALRIFSMMEEEKRREVGLITRRSRGDLRAAKL
MKDRGLWLNRDKRIVGSIPGINIGDLFLFRMELCVVGLHGQAQAGIDYLPGSRSSNGEPIATSIIVSGIKYEGSVTGKVYVYDGLYKIHDSWFDVGKSGF
GVYKYKLLRNEGQAEMGSAILRFAENLRVSPLTVRPVGYLCDDLSTKKENIPVFLFNDIDGDNEPMYYEYLPRTVFPLHAYNLGGNGSGCDCVAGCTDDC
VCAQRNGGEFAYDQNGFLLRGKPVIFECGSFCRCPPTCRNRLTQKGLRNRFEVFRSRETGWGVRSLDLIQAGAFICEYAGVVLTREQAALFSMNGDTLIY
PNRFTDRWAEWGDFSKVYSDYVRPMHPSIPPLDFAMDVSRMRNLACYMSHSSCPNVLVQFVLYDHHNLLFPRLMLFAMENIPPLRELSLDYGVADEWTGK
LPICN

> VvSET31 GSVIVT01027907001
MVLQLKTYSNLLANASGEDQANGVSDSSQFDIASSPNGEVKISLILNTSQQSGCHIPNLDAVSKALEDKCRGTYGITEPSFSV
MKLMQEFCEYFLAIGADSTDDEKLKTMETSSTLDILKEPAAQDVLGRGDHKGKFCIPSSSSNGSVKCQNLVEVESLNSCGIVAVQKHCFSVDTVKPLQYF
DDITKGEEMVKISLVNGTSSQLPPNFFYIPQNIVFQKAYVNFALARISDEDCCSNCFGDCTSLAIPCACARETGGEFAYQQGGLVKEKFLEECISMNRDP
QNHRLFYCKNCPLERSRNENTSNPCKGHLVRKFIKECWCKCGCSKKCGNRVVQRGITVNLQVFLTPEGKGWGLRTLENLPKGAFVCEYVGEIVTNTELYE
RNLRSTGKERHTYPVLLDADWGSEGVLKDEEALCLDATFYGNVARFINHRCFDANLVEIPVEVETPDHHYYHLAFFTTRKVDALEELTWDYGIDFDDHNH
PVKAFRCCCESKGCRDTRNSKRHGVKRRKMEMKA

> VvSET32 GSVIVT01005913001
MEFEVKREQSIGSPRENNLPRPDQKSQIVEKANEVLEGKVGKEIVIYSKDENSKRKVTSLSGRVNKVPAGDELSQERVTVLCL
MAAQNCPWRRQGKGGLKLDSGMIEACDSIVTRNKVRETLRLFQAIFRKLLQEEEAKTKQGGNPVRRVDYLASRILKDKGKHVNTGKQIIGPVPGVEVGYA
DDLDNSDVLIYSGQGGNLIAPEYMDSRAKVVTTYIYDGLYLVEKYWQEIGPHGKLVFKFQLNRIPGQPELAWKEVKNSKKFKVREGLCVDDISMGKEPIP
IFAVNTIDDEKPPPFTYITSMIYPDWCHRLPPNGCDCSNGCSDSEKCSCAVKNGGEIPYNYNGAIVEAKPLVYECSPSCKCSRSCHNRVSQHGIKFQLEI
FKTVSRGWGVRSLTSIPSGSFICEYIGELLEDKEAEQRTGNDEYFSCEVVEDAGFTIDAAQYGNVGRFINHSCSPNLYAQNVLYDHDNKRIPHIMLFAAE
NIPPLQELTYHYNYTIDQVRDSNGNIKKKSCYCGSDECTGRMY
